# Transcutaneous auricular VNS applied to experimental pain: A paired behavioral and EEG study using thermonociceptive CO_2_ laser

**DOI:** 10.1371/journal.pone.0254480

**Published:** 2021-07-12

**Authors:** Manon Dumoulin, Giulia Liberati, André Mouraux, Susana Ferrao Santos, Riëm El Tahry

**Affiliations:** 1 Institute of Neuroscience, Université Catholique de Louvain (UCLouvain), Brussels, Belgium; 2 Centre for Refractory Epilepsy, Cliniques Universitaires Saint-Luc, Brussels, Belgium; University of Essex, UNITED KINGDOM

## Abstract

**Background:**

Transcutaneous auricular Vagal Nerve Stimulation (taVNS) is a non-invasive neurostimulation technique with potential analgesic effects. Several studies based on subjective behavioral responses suggest that taVNS modulates nociception differently with either pro-nociceptive or anti-nociceptive effects.

**Objective:**

This study aimed to characterize how taVNS alters pain perception, by investigating its effects on event-related potentials (ERPs) elicited by different types of spinothalamic and lemniscal somatosensory stimuli, combined with quantitative sensory testing (detection threshold and intensity ratings).

**Methods:**

We performed 3 experiments designed to study the time-dependent effects of taVNS and compare with standard cervical VNS (cVNS). In Experiment 1, we assessed the effects of taVNS after 3 hours of stimulation. In Experiment 2, we focused on the immediate effects of the duty cycle (OFF vs. ON phases). Experiments 1 and 2 included 22 and 15 healthy participants respectively. Both experiments consisted of a 2-day cross-over protocol, in which subjects received taVNS and sham stimulation sequentially. In addition, subjects received a set of nociceptive (thermonociceptive CO_2_ laser, mechanical pinprick) and non-nociceptive (vibrotactile, cool) stimuli, for which we recorded detection thresholds, intensity of perception and ERPs. Finally, in Experiment 3, we tested 13 epileptic patients with an implanted cVNS by comparing OFF vs. ON cycles, using a similar experimental procedure.

**Results:**

Neither taVNS nor cVNS appeared to modulate the cerebral and behavioral aspects of somatosensory perception.

**Conclusion:**

The potential effect of taVNS on nociception requires a cautious interpretation, as we found no objective change in behavioral and cerebral responses to spinothalamic and lemniscal somatosensory stimulations.

## Introduction

Several studies have suggested that vagus nerve stimulation (VNS), a technique used for the treatment of refractory epilepsy, may also modulate nociception and pain perception [1, 2]. This has led to multiple potential applications to various painful conditions, such as headache [3], trigeminal allodynia [4], chronic pelvic pain [5] and fibromyalgia [6].

The possible mechanisms by which VNS modulates pain perception might result from the common anatomical pathways shared between the nociceptive system and the central projections of the vagal afferents.

Somatosensory information can be divided between mechanosensory and thermonociceptive inputs [7]. While the former is conveyed by encapsulated low thresholds mechanoreceptors of myelinated Aβ-fibers (responsible for vibrotactile and proprioceptive signals), the latter is transduced by high threshold mechanoreceptors and free nerve endings of thinly myelinated Aδ- and unmyelinated C-fibers (responsible for the signaling of sharp mechanical, thermal and chemical stimuli, of potential noxious nature). After a first relay at the dorsal root ganglion of the spinal cord, transmission to higher order neurons follows the lemniscal pathway for mechanosensation, while thermonociception ascends via the spinothalamic tract [8]. Both fascicles relay within the contralateral ventro-postero-lateral nuclei of the thalamus [7], prior to widespread diffusion to cortical areas such as the primary sensory cortex and paracentral lobule (responsible for the perception of sharp localized pain), the insula and/or rostral cingulate gyrus (responsible for the dull and deep pain, as well as the emotional features of the sensations) [9]. Once pain emerges as a conscious experience, top-bottom control of pain perception loops back to the dorsal horn of the spinal cord through noradrenergic, serotoninergic and opioid descending inhibitory pathways [9, 10].

With regards to vagal projections, auricular local anesthetic and transganglionic chemical tracers studies in animals indicate the importance of the nucleus tractus solitarius, raphe magnus, locus coeruleus, and periaqueductal gray in vagal nerve transmission [1, 11–14]. Importantly, each of these structures is part of the inhibitory descending pathways relative to the nociceptive system [9, 10]. In addition, positron emission tomography studies in humans have shown modulation of VNS on the thalamus, hypothalamus and insula [15, 16], all key regions involved in pain processing [7, 9]. Similarly, bilateral fMRI alterations were found under VNS at all levels of central nociceptive processing, from cortical areas (primary and secondary sensory, prefrontal and anterior cingular cortices), to subcortical regions (insula, thalamus, hypothalamus, amygdala), and brainstem nuclei (locus coeruleus, periaqueductal gray, dorsal raphe nuclei) [17–28]. Within these areas, the VNS-induced mood [29] and pain modulations [30] could result from alterations in specific neurotransmitters expression such as serotonin [31–34], noradrenaline [35–37], opioids [38], and GABA [39, 40].

Additionally, the anti-inflammatory effects of VNS might further concur to the observed antinociceptive effects [41], as decreased TNFα serum levels [42–45], decreased oxidative stress [46, 47] and increased cortisol releases [48, 49] were observed under VNS.

Despite the growing evidence, the exact mechanism of how VNS modulates pain perception in humans remains unclear, with both contradictory pro- and anti-nociceptive effects observed at present [50–56]. One study has shown that low intensity invasive cervical VNS (cVNS) leads to a reduction in thermal pain threshold in epileptic patients, corresponding to a pro-nociceptive phenomenon [53]. In addition to cVNS, the effect of transcutaneous auricular VNS (taVNS) on nociception were also investigated by several researchers. In the case of taVNS, the vagus nerve is stimulated through the auricular branch of the vagus nerve (ABVN), responsible of the epidermal innervation of the cymba conchae, tragus and posterior canal of the ear [57–61]. Using quantitative sensory testing (QST) in healthy subjects, two studies observed that taVNS (applied with a commercial device, Nemos) achieved a global analgesic effect on mechanical [62], pressure [62, 63] and tonic heat pain paradigms [62]. On the other hand, using an independent device with customable settings, Janner et al reported that the analgesic effect of taVNS on repetitive noxious heat was equivalent to that observed with active sham and placebo conditions [64]. Moreover, for electrical pain, Laqua et al reported that depending on the individual, taVNS could exert either anti- and pro-nociceptive effects [54]. These contradictory results were further observed in an fMRI study exploring how taVNS modulates pain perception [55]. In this study, Usichenko et al administered tonic heat pain with and without taVNS to healthy volunteers, combined with the quantification of behavioral pain thresholds before and after the fMRI sessions. Although taVNS induced a decreased activity in the anterior cingulate cortex after the application of tonic heat pain at a group-level, a subgroup analysis revealed that taVNS exerted both anti- and pro-nociceptive changes in behavioral responses. Finally, using QST in chronic pain patients, one study observed that taVNS decreased mechanical pain perception [5], while another study reported a lack of effect on pressure pain perception [65].

While reviewing the literature, we noticed that the majority of previous studies mostly relied on behavioral assessments solely, therefore leaving our present state of knowledge at risk of limitations due to subjective variability [66].

Complementary to QST, nociception research has made extensive use of event-related potentials (ERPs) elicited by thermonociceptive laser stimulations, which have proven to be a validated, alternative method to probe the functional integrity of the nociceptive system (from peripheral nociceptors, spinothalamic transmission up to cortical projections) [67, 68]. Although laser-ERPs are not entirely specific to nociception and might also reflect a certain degree of psychological artefacts [68], the combined use of both QST and somatosensory evoked-ERPs might help increase our understanding of the observed subjective responses [69].

Additionally, there is still little information available on the time course effect of taVNS [5, 24, 25, 50], and the question arises whether the effect of VNS on nociception is time-dependent (i.e. short or long lasting).

Hence, in order to gain a better understanding of how VNS affects nociception, we aimed to study how VNS modulates laser-evoked ERPs, which has not been performed to date. Within this frame, we applied a variety of spinothalamic/lemniscal stimuli and recorded the elicited ERPs with regards to their perceptual correlates (noxious, innocuous), to determine whether VNS has differential effects related to Aδ-, C- or Aβ-fibers inputs respectively. Two experiments (Experiments 1 and 2) were designed to address the possible time-dependent effects of taVNS. Furthermore, because auricular nerve topography, as well as sensitivity to stimulation of vagal afferents, might highly vary from one individual to another [61, 59, 70], we expected taVNS to present variable efficacy amongst subjects [71]. A third experiment (Experiment 3) was therefore performed to study the effect of cervical VNS in epileptic patients, which remains the gold standard in terms of vagus stimulation.

## Methods

From August 2018 to September 2019, three experiments were conducted at the NOCIONS laboratory (Institute of Neuroscience, UCLouvain—Brussels, Belgium). The research was approved by our local ethical committee (Hospital and Departmental Ethics Committee, Saint-Luc Hospital, UCLouvain–Brussels, Belgium), and written consent was obtained in all participants (ethical reference: B403201630289).

The first two experiments were conducted in healthy subjects, with aim to determine the time-course effect of taVNS in modulating pain perception. Experiment 1 focused on studying the alterations observed in pain perception after 3 hours of taVNS, while Experiment 2 focused on the immediate effects of its duty cycle on pain perception.

Experiment 3 was implemented to address the potential limitation induced by the unknown efficacy of taVNS in recruiting vagal afferents. We therefore tested epileptic patients treated with an implanted cervical VNS (cVNS).

### 1. Experiment 1

In Experiment 1, each participant underwent a 2-day protocol, during which 3 hours of taVNS and active sham stimulation were administered in a cross-over design, to assess their respective effects on somatosensory perception. Each session was empirically separated by a minimal interval of 48h (to allow sufficient wash out time [62, 64]) and conducted at the same time of the day (to reduce physiological variability).

#### 1.1 Participants

Inclusion criteria for healthy subjects were right-handed participants between 18–65 years old. Exclusion criteria were chronic pain, active neurological or psychological conditions, skin irritation at ear location, pregnancy, as well as the recent/chronic use of medications or recreational drugs. Additional exclusion criteria consisted of medical comorbidities affecting vagal functions (cardiac disease, diabetes, gastro-esophagal reflux, inflammatory diseases of pulmonary, digestive or rhumatismal origins). Participants were asked to refrain from heavy exercise 12h prior to each experimental session, and sleep sufficiently the night before. Caffeine consumption was avoided or kept to a minimal use.

Experiment 1 was completed by 22 subjects (10 males, 12 females), with mean age of 27.32 ± 9.11 years (median: 24.5, min. 20 –max. 55). On day 1, 14 participants received taVNS, while 8 participants received sham stimulation. The mean interval between each experimental session was of 5 ± 2 days (median: 2 days, min. 2 days—max. 14 days). In experiment 1, participant 16 was excluded from the analysis of Cool-evoked ERPs, as the onset of the cool-stimulus delivery did not appear on the EEG recording.

#### 1.2 taVNS

TaVNS was conducted on the left ear with a standard commercial device (Nemos/Vitos, Erlangen, Germany), consisting of a bipolar electrode connected to a generator. Stimulation parameters were predefined as monophasic rectangular pulses at a 25 Hz frequency, 250 μs pulse width, with a duty cycle of 30s ON /30s OFF. Current intensity (mA) was individually titrated to elicit a maximal, but non-painful, tingling sensation [72]. Electrode contact was continuously monitored throughout the stimulation duration.

**Fig 1:**
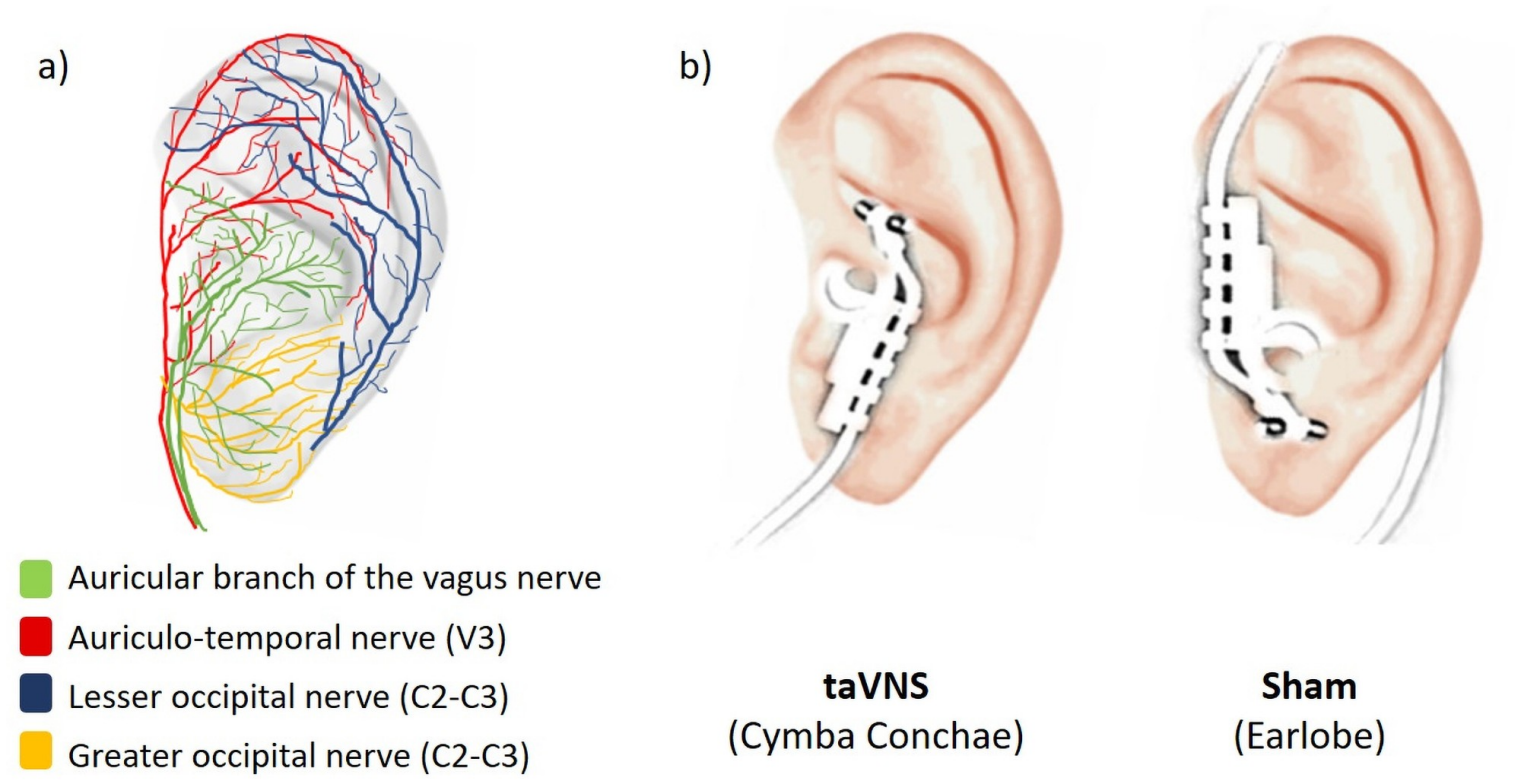
Auricular stimulation. a) Auricular ear anatomy. b) Electrode positions for taVNS and sham conditions, on the left ear. Stimulations parameters were identical between both conditions, consisting of monophasic rectangular impulses at 25Hz and 250μs pulse width, with a duty cycle of 30s ON /30s OFF. Current intensity (mA) was titrated at individual level to elicit a maximal, but non-painful, tingling sensation. *Abbreviations*: *V3*: *Third branch of the trigeminal nerve (mandibular branch)*. *C2 and C3*: *Second and Third cervical roots of the cervical plexus*. *Adapted from Peuker & Filler (2002)*, *He et al (2012)*.

Real taVNS and active sham conditions only differed by the electrode placement on the ear, with taVNS on the cymba conchae and sham on the earlobe [26] (Fig 1). The left side for taVNS was chosen for homogeneity with the left sided cervical VNS performed in Experiment 3. Hereafter, “auricular treatment” refers to both real taVNS and sham stimulation indistinctively.

#### 1.3 Somatosensory stimuli

Somatosensory stimuli were applied on the right hand contralateral to auricular treatment (see details below). When applied on the hand dorsum, the target skin area was randomly shuffled by ± 2 cm between each stimulus, to reduce receptor fatigue and/or sensitization [73–75]. Somatosensory modalities of either spinothalamic or lemniscal transmission were both tested to assess the selectivity of the effects of taVNS.

**Brief, radiant heat pulses** were delivered on the hand dorsum with a CO_2_ laser (SIFEC, Ferrières, Belgium), with a laser probe capable of measuring skin temperature through a built-in radiometer. The laser beam was characterized by a 10.6μm wavelength and a 6 mm diameter beam at target site. Laser-pulses were calibrated to reach 62.5°C at skin location within 10ms, for a total duration of 50ms. Participants were expected to predominantly perceive burning/pricking sensations upon the concomitant activations of heat-sensitive C- and Aδ-fibers nociceptors, although the isolated activation of heat-sensitive C-fibers can produce only warmth [75].

**Cooling of the skin** was induced on the hand dorsum using a micro-Peltier thermode (TCS1, QST La, Strasbourg, France). Using a 125mm^2^ probe, the thermode was calibrated to reach 10°C at skin location, with a cooling ramp of 200°C/s, for a total duration of 250ms. Participants were expected to report a cool/cold sensation upon the selective activation of cool-sensitive free nerve endings of the skin [76].

**Mechanical stimulation** was delivered manually with a 128mN pinprick device (MRC Systems, Heidelberg, Germany) applied on the hand dorsum. Pinprick stimuli were expected to induce a pricking sensation, responsible for the selective activation of Aδ-nociceptors [77].

**Vibrotactile stimulation** was delivered on the fingertips of the index and thumb, using a recoil-type vibrotactile transducer (length: 2.8 cm, width: 1.2 cm, Haptuators; Tactile Labs, Montreal, ON, Canada), and served as a control stimulation of the lemniscal pathway [62]. Vibrotactile-pulses were calibrated with a 250Hz frequency, for a 50ms duration. This stimulation modality was expected to induce a vibrating sensation upon the selective activation of Aβ-mechanoreceptors [75].

Prior to each set of somatosensory testing, skin temperature was measured with the built-in radiometer of the laser probe, and averaged between three subsequent values obtained from distinct areas of the right hand dorsum.

#### 1.4 Detection thresholds and intensity of perception

**Detection thresholds** for heat-sensitive C- and Aδ-fibers, cool-sensitive Aδ-fibers, and Aβ mechanoreceptors were determined using a staircase algorithm based on stimulus detection [78]. All stimuli were conducted on the right hand, contralateral to the auricular treatment. To measure reaction times, participants were tasked to press a button held on their left hand “as soon as they could feel a stimulus”.

*Detection thresholds for heat-sensitive C-fibers* were estimated using the CO_2_ laser, calibrated with a starting value of 41°C at the right hand dorsum, for a total duration of 100ms (10ms heating ramp, 90ms plateau). A staircase reversal was defined as a detected stimulus followed by an undetected one (or vice-versa). Depending on whether the stimulus was detected or not, the following stimulus temperature was decreased or increased respectively by 1°C prior to the first reversal, then by ± 0.5°C. The threshold was computed by averaging the four values at which a staircase reversal occurred.

Similarly, *detection thresholds for heat-sensitive Aδ-fibers* were determined with a starting value of 46°C (100ms, ± 1°C before first reversal, then ± 0.5°C), with an additional criterion of a < 650ms reaction time.

*Detection thresholds for cool-sensitive free nerve endings* were approximated based on sole detection criterion. The thermode was calibrated with a starting temperature of 29°C, with increment/decrement of ± 1°C prior to the first staircase reversal, then of ± 0.5°C.

*Detection thresholds for Aβ-fibers* were determined using the Haptuator device calibrated through an abstract computer scale starting at 0.002, with increment/decrement of ± 0.0005 of abstract unit (A.U) prior to the first reversal, then of ± 0.0001 A.U.

**Intensity of perception** was retrieved for laser, cool, pinprick and vibrotactile modalities, with the suprathreshold calibrations described under *1*.*3 Somatosensory Stimuli*. Following each single stimulus, participant were asked to rate the perceived intensity using a numerical rating scale ranging from 0 (“no sensation”) to 10 (“maximal sensation”), regardless of pain perception. The average of all ratings obtained during one block of 40 stimuli was used to determine the intensity of perception for a specific somatosensory modality.

#### 1.5 Event-related potentials

Event-related potentials to laser, vibrotactile and cool were elicited by blocks of 40 suprathreshold stimuli (one block per modality), with a variable interstimulus interval (self-paced by the experimenter, from 3 to 10s). During each block, participants were quietly seated and asked to keep their gaze fixed on a cross placed in front of them (5x5mm, ~30° below eye level, ~50 cm distance). To help maintain an adequate level of attention, participants had to press a button held on their left hand, as soon as they could feel a stimulus on their right hand.

An electroencephalogram (EEG) was recorded concomitantly using a 32 Ag-AgCl electrode cap according to the international 10–20 system (Waveguard 32-Channel Cap, Advanced Neuro Technologies, The Netherlands). The impedance was kept below 20kΩ in all electrodes, and below 10kΩ at electrodes Cz, M1 and M2. Two surface electrodes were placed on the upper-left and lower-right sides of the right eye, to monitor ocular artifacts (horizontal electrooculogram). To minimize accidental auditory bias, subjects wore headphones and listened to white noise while performing the experimental task. Data acquisition was conducted using a digital amplifier with a 1kHz sampling rate and 26.55x amplification scale (ASA-LAB EEG/ERP system, Advanced Neuro Technologies, The Netherlands). The recorded EEG signals were analyzed offline using Letswave 7 (www.letswave.org). Cerebral responses were recorded for all somatosensory stimuli, with the exception of pinprick stimulations, as the manual device could not be paired with the EEG acquisition system. To obtain somatosensory-evoked ERPs, the raw EEG data was first band-pass filtered (0.5-30Hz), then segmented into 2s-epochs per stimulus modality, ranging from -0.5s to +1.5s relative to the stimulus onset. An Independent Component Analysis (ICA) *(RUNICA algorithm–EEGlab*, *Square method)* was used to identify and remove ICA filters responsible for ocular artifacts in the EEG recordings [79]. Thereafter, a visual inspection was performed to complete the rejection of epochs with an amplitude value > 100μV, susceptible to artifact contamination. In Experiment 1, the mean ± SD of rejected epochs was of 0.19 ± 0.49 for laser stimulations, 0.29 ± 0.86 for vibrotactile stimulations and 0.33 ± 0.86 for cool stimulations. Epoch rejection was completed by baseline substraction with the prestimulus reference interval -0.5s to 0s. In each participant, separate averaged waveforms were computed for all epochs with respect to *one stimulation modality* (laser, vibrotactile or cool), *one auricular condition* (taVNS or sham), and *one specific timepoint* (before or after stimulation). The averaged waveforms were analyzed at the Cz electrode to identify two quantifiable peaks, namely N2 and P2 [75, 76]. Regardless of stimuli modality, N2 was defined as the largest negative deflection between 0.1s to 0.5s after stimulation onset, and P2 as the first positive deflection following N2. At last, to obtain group-level ERPs of an experimental session, individual waveforms relative to one stimulus modality and auricular condition were combined into a grand-average.

#### 1.6 Time course of Experiment 1

**Fig 2:**
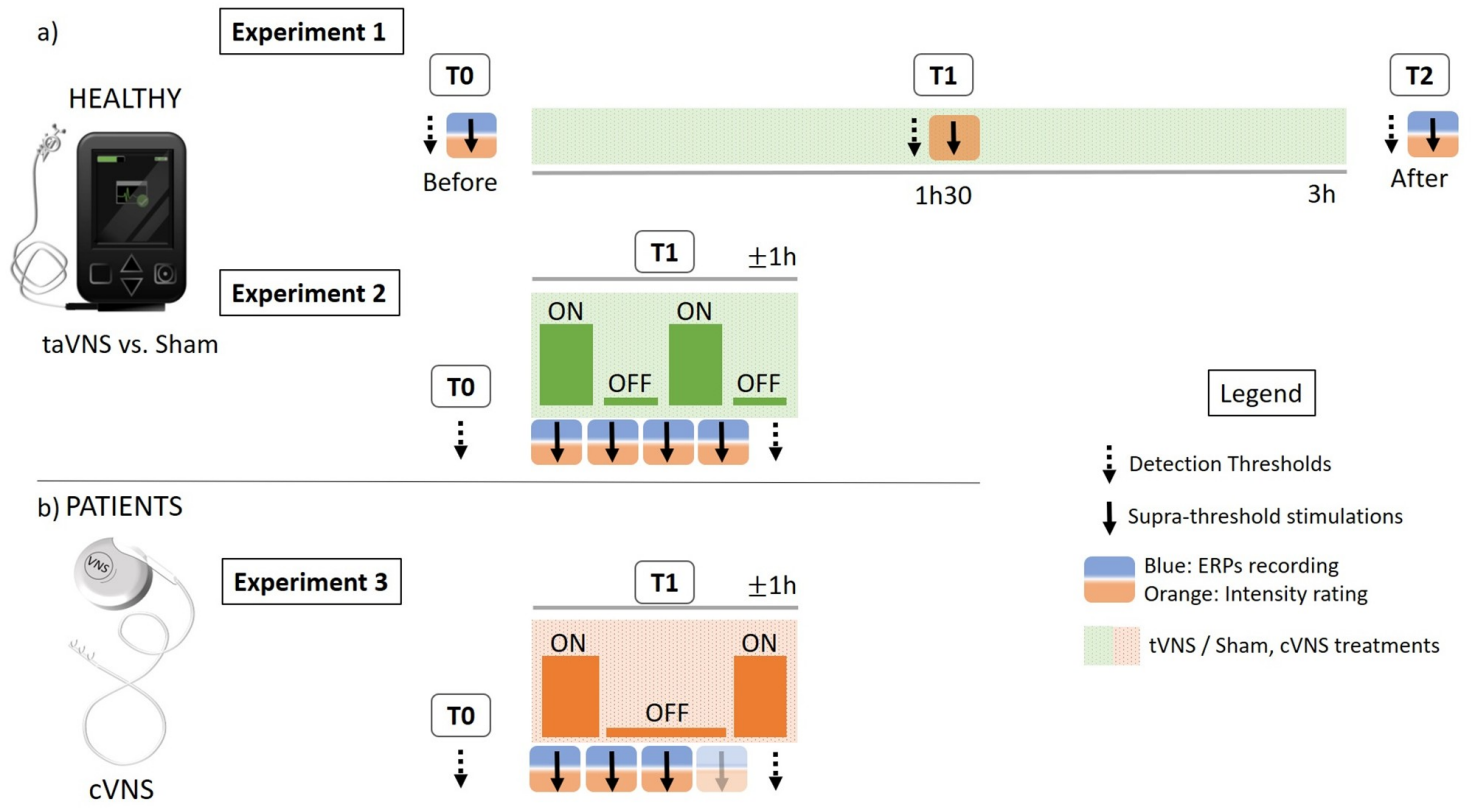
Overview of all experiments. a) Experiments conducted in healthy subjects. Each participant underwent a randomized protocol, during which non-invasive taVNS and sham stimulations were delivered in a 2-day cross-over design. Experiment 1 focused on the somatosensory effects induced after 3h of taVNS/sham stimulation, while Experiment 2 focused on the immediate effects of the OFF/ON phases of the duty cycle (30s ON/30s OFF). b) Experiment 3 was conducted in epileptic patients. The experiment focused on the immediate effects of the OFF/ON phases of the duty cycle (rapid cycling: 30s ON/ 1.1min OFF) of the implanted cervical VNS in a 1-day protocol. Participants completed a minimum of 3 blocks of suprathreshold stimulations, and a fourth depending on tolerability (fading 4th arrow). *Abbreviations*: *ERPs = Event Related Potentials*, *taVNS = transcutaneous auricular vagus nerve stimulation*. *cVNS = cervical vagus nerve stimulation T0 = baseline*. *T1 = during vagus nerve stimulation*. *T2 = after vagus nerve stimulation*.

Laser, vibrotactile and cool stimuli were tested for detection thresholds and suprathreshold stimulations (1 block of 40 stimuli per modality) at baseline (= T0), midway through auricular stimulation (= T1), and after 3h of auricular stimulation (T2). EEG recordings were acquired only at T0 and T2. Pinprick stimuli were tested for intensity ratings only, at all timepoints (T0, T1, T2) (Fig 2).

#### 1.7 Statistical analysis

Power and sample size estimations were based on two studies of n = 48 [62] and n = 49 [64] healthy participants, in which the reported effect sizes (large effect size of *η*_*p*_^*2*^ > 0.2) indicated that a sample size of 10–12 participants should be sufficient to reach a statistical power of 0.80–0.90.

All statistical analysis were performed with IBM SPSS Statistic 25 (Armonk, New York: IBM). For each somatosensory modality (laser, cool, pinprick, vibrotactile), a linear mixed model (LMM) analysis was used to assess the effects of several fixed factors on the recorded behavioral and brain responses. **In Experiment 1**, the fixed factors were ‘conditions’ (2 levels: taVNS and active sham) and ‘time’ (3 levels: T0,T1,T2).

A significant effect of ‘time’ would indicate that 1h30 (T1) and/or 3h (T2) of auricular stimulation successfully altered the observed behavioral and/or cerebral responses evoked by a specific somatosensory modality.

A significant 2-way interaction between ‘condition’ x ‘time’ would demonstrate a differential effect between taVNS vs. sham stimulation on the recorded cerebral and/or behavioral response(s) to a specific somatosensory modality, observable after 1h30 and/or 3h of stimulation as compared to baseline.

When appropriate, post-hoc analysis were conducted using Bonferroni confidence interval adjustment, as well as bilateral paired t-student tests and one-way ANOVAs with factor ‘Condition’, to search for a differential effect of taVNS vs. sham stimulation on the changes induced in each parameter of interest (ΔTime or ΔPhase). For each response, Pearson correlations with skin temperature were performed to rule out bias induced by potential changes in skin temperature. Significance level was set at p = 0.05 for all analyses. Cross-conditions and cross-experiments comparisons (i.e intensity and duration of auricular stimulations) were conducted with paired or independent bilateral t-student tests respectively.

Complementary Bayesian analyses (Bayesian Repeated Measures ANOVA) were conducted using the free software Jasp 0.14.1 (https://jasp-stats.org/download/), with default parameters for the Cauchy distribution (location centered around 0, with width parameter *r* = 0.707) (for details, see S1 Appendix–Bayesian analysis).

### 2. Experiment 2

Experiment 2 focused on the immediate effects of the ON/OFF phases of the duty cycle of taVNS. Similarly to Experiment 1, each participant underwent a 2-day protocol experiment, with taVNS and sham stimulation administered in a cross-over design. Based on the results of the ERPs recorded in Experiment 1, we choose to only reconduct the somatosensory modalities with the best signal to noise ratio (SNR). Thus, in Experiment 2, only laser and vibrotactile stimulations were performed, with significant reduction in experimental duration.

#### 2.1 Participants

Inclusion and exclusion criteria were identical to those of Experiment 1. Experiment 2 was completed by 15 healthy subjects (9 males, 6 females), with mean age of 30.13 ± 11.23 years (median: 27, min. 21 –max. 56). On day 1, 7 participants received taVNS, while 8 participants received sham stimulation. The mean interval between each experimental session was of 21 ± 44 days (Median: 7 days, min. 3 days–max. 176 days).

#### 2.2 taVNS

Auricular stimulation was delivered to participants according to the procedure described in Experiment 1, for 1 hour approximately (depending on individual speed to rate the perceived somatosensory stimuli). Additionally, the experimenter continuously monitored the ongoing EEG recording for the presence of a 25Hz electrical artifact, suggestive of the active phases of the taVNS.

#### 2.3 Detection thresholds and intensity of perception

Detection thresholds and intensity ratings were retrieved similarly to Experiment 1. To improve SNR, suprathreshold stimuli were delivered slightly differently than in Experiment 1: laser stimuli were calibrated to reach 60°C with a 10ms ramp for a total duration of 50ms, and vibrotactile stimuli were calibrated with a 300Hz frequency, lasting 100ms.

#### 2.4 Event-related potentials

To follow the time course effects of taVNS, suprathreshold stimuli were delivered in 4 blocks, each consisting of 20 pairs of alternating laser and vibrotactile stimuli. Each stimulus was then categorized offline into ON or OFF phase of the duty cycle, based on the respective presence or absence of a stimulation artifact on the EEG. ERP recordings and analyses were conducted similarly to Experiment 1. In Experiment 2, the mean ± SD of rejected epochs was of 0.20 ± 0.48 for laser stimulations and 0.10 ± 0.35 for vibrotactile stimulations.

#### 2.5 Time course of Experiment

2Detection thresholds for laser and vibrotactile stimuli were tested at T0 and at the end of T1 (before and at +/- 1h of ongoing auricular stimulation). Note that comparatively to Experiment 1 where T1 referred to 1h30 of ongoing auricular stimulation, in Experiment 2, T1 represented the ongoing auricular stimulation, lasting approximately 1 hour depending on the participant. Suprathreshold stimulations were tested during T1, in 4 blocks of 20 pairs of alternating laser and vibrotactile stimuli, during which cerebral responses and intensity ratings were recorded (see Fig 2).

#### 2.6 Statistical analysis

Statistical analyses were conducted similarly to Experiment 1. Using a LMM, we assessed how the following fixed factors influenced the behavioral and cerebral responses to either laser or vibrotactile stimuli: ‘condition’ (2 levels: taVNS, sham stimulation), ‘time’ (2 levels: T0, T1, only for detection thresholds analyses), ‘phase of the duty cycle’ (2 levels: ON, OFF, for cerebral responses and intensity ratings).

A significant effect of ‘condition’ would indicate a differential effect between taVNS vs. sham stimulation, on the elicited cerebral or behavioral responses to the tested somatosensory modality. A significant effect of ‘time’ would demonstrate that regardless of taVNS or sham stimulation, +/-1h of auricular stimulation successfully altered detection thresholds to a specific somatosensory modality. A significant effect of factor ‘phase of the duty cycle’ would highlight a differential effect between the OFF vs. ON phases of the auricular stimulation on the elicited cerebral responses or intensity ratings to a specific somatosensory stimuli, with no distinction between taVNS and sham stimulation.

A significant 2-way interaction between ‘condition’ x ‘time’ would suggest a differential effect of 1h of taVNS vs. 1h of sham stimulation on the elicited detection thresholds of the tested somatosensory modality. A significant 2-way interaction between ‘condition’ x ‘phase of the duty cycle’ would demonstrate a differential effect of the OFF vs. ON phases of the duty cycles between taVNS vs. sham stimulation, observable on the recorded cerebral responses and/or intensity ratings of a specific somatosensory modality.

### 3. Experiment 3

In experiment 3, we tested epileptic patients implanted with a cVNS during a single session (lasting approximately 1 hour).

#### 3.1 Participants

Inclusion criteria were refractory epileptic patients implanted with a cVNS (regardless of implantation duration), aged between 18–65 years old. Exclusion criteria was moderate to severe cognitive impairment (assessed through neuropsychological evaluation done as a part of the standard presurgical evaluation). Experiment 3 was completed by 13 patients (7 males, 6 females), with mean age of 35.3 ± 12.6 years old (median: 33, min. 19—max. 61). The mean duration of cVNS treatment was of 5 years, 1 month and 27 days ± 3y 8m 19d (median: 4y 7m 28d; min. 4m 27d– max. 14y 4m 21d) (see Table 1 for other characteristics).

**Table 1:** Descriptive data: Epileptic patients.

Experiment 3	Age (years)	Gender	Epileps type (Etiology)	Number of antiepileptics	Anti-epileptic response to cVNS	cVNS parameters (device type)	Time since VNS implantation
Group level (Mean ± SD)	35.3 ± 12.6	6 males, 7 males		2	Responders (6)	Median: 1.25, 20Hz, 250μs,30”ON/5’OFF	5y 1m 27d ± 3y 8m 19d
					Non Responders (7)		
Patient 1	61	F	Focal (Sequellar)	2 (VPA, LTG)	NR	1.5mA, 20Hz, 250μs, 14”ON/1.1’ OFF Demipulse 103	8y 7m 29d
Patient 2	33	F	Focal (Genetic)	2 (LTG, BRV)	NR	2.0mA, 25Hz, 250μs, 30”ON/ 5’OFF Aspire 106	8y 4m 24d
Patient 3	31	F	Generalized (Genetic)	1 (VPA)	NR	1.375mA, 20Hz, 250μs, 30"ON/5’OFF Aspire 106	0y 8m 20d
Patient 4	52	M	Focal (Genetic)	2 (CBZ, LCM)	R	1.25mA, 30Hz, 500μs, 30"ON/ 5’OFF Demipulse 103	14y 4m 21d
Patient 5	25	F	Focal (Sequellar)	3 (VPA, OXC, PER)	NR	1.5mA, 20Hz, 250μs, 7"ON/ 0,3’ OFF Demipulse 103	4y 6m 17d
Patient 6	28	F	Generalized (Genetic)	2 (LEV, LTG)	R	1.0mA, 20Hz, 250μs, 30"ON/ 5’OFF Demipulse 103	6y 9m 14d
Patient 7	46	F	Focal (Unknown)	2 (LEV, LCM)	R	1.0mA, 20Hz, 250μs, 30"ON/ 5’ OFF Aspire 106	2y 1m 9d
Patient 8	37	M	Focal (Unknown)	2 (LEV, LCM)	R	0.75mA, 25Hz, 250μs, 30"ON/5’OFF Aspire 106	2y 7m 0d
Patient 9	20	F	Focal (Sequellar)	2 (LEV, LTG)	R	1.25mA, 20Hz, 250μs, 30"ON/5’OFF Aspire 106	3y 5m 22d
Patient 10	40	M	Focal (Genetic)	3 (CBZ, BRV, PER)	NR	2.0mA, 30Hz, 250μs, 14"ON/1,1’OFF Demipulse 103	4y 7m 28d
Patient 11	19	M	Focal (Unknown)	3 (LEV, LCM, PER)	NR	1.0mA, 30Hz, 250μs, 30"ON/5’OFF Aspire 106	0y 4m 24d
Patient 12	42	M	Focal (Unknown)	3 (LTG, CBZ, PER)	NR	1.75mA, 20Hz, 500μs, 30" ON/5’OFF Demipulse 103	4y 7m 28d
Patient 13	25	M	Generalized (Genetic)	3 (VPA, LTG, TPM)	R	1.0mA, 30Hz, 250μs, 30"ON/5’OFF Demipulse 103	7y 2m 22d

Descriptive data for epileptic patients. Gender and clinical response to VNS were balanced. The antiepileptic response to cVNS was defined as responsive if the patient observed a reduction of > 30% in their seizure frequency since cVNS implantation. *Abbreviations*: *cVNS = cervical vagus nerve stimulation*. ***Gender***: *F = female*. *M = male*. ***Antiepileptics***: *BRV = Brivaracetam*, *CBZ = Carbamazepine*, *LCM = Lacosamide*, *LEV = Levetiracetam*, *LTG = Lamotrigine*, *OXC = Oxcarbazepine*, *PER = Perampanel*, *VPA = Valproic acid*. ***Antiepileptic response***: *NR = Non responder*. *R = Responder*.

In Experiment 3, Patient 11 was excluded from intensity analysis, as this patient showed difficulties in scoring the intensities of the alternating vibrotactile and laser stimuli.

#### 3.2 cVNS

Patients had cVNS implants (Demipulse© Model 103 or AspireSR© Model 106) from Livanova (Inc., London, United Kingdom). To keep the experiment manageable duration-wise, the cVNS parameters were reprogrammed to a rapid cycling (30s ON/ 1.1min OFF). Other parameters were kept as programmed for the chronic treatment.

#### 3.3 Detection thresholds and intensity of perception

Procedures for detection thresholds and intensity of perception were conducted as in Experiment 2.

#### 3.4 Event-related potentials

Laser and vibrotactile-ERPs were recorded similarly to Experiment 2, with exception that stimulation artefacts of the cVNS were recorded with an additional pair of surface electrodes, placed on the lateral portion of the neck to follow the trajectory of the implanted VNS electrode. In Experiment 3, the mean ± SD of rejected epochs was of 0.23 ± 0.51 for both laser and vibrotactile stimulations.

#### 3.5 Time course of Experiment 3

Procedures were similar to Experiment 2, except for the fact that given the chronic nature of the cVNS implant, detection thresholds were only tested once at the start of the experiment (T0) (see Fig 2).

#### 3.6 Statistical analysis

Statistical analyses consisted of LMM, with one fixed factor to describe the effects on laser- and vibrotactile-evoked cerebral responses and intensity ratings: ‘phase of the duty cycle’ (2 levels: ON, OFF). A significant effect of factor ‘phase of the duty cycle’ would highlight a differential effect between the OFF vs. ON phases of cVNS, on the elicited cerebral responses or intensity ratings to a specific somatosensory stimuli.

## Results

### 1. Parameters of auricular and cervical conditions

In Experiment 1, mean current intensities were of 1.5 ± 0.9 mA for active taVNS compared to 1.8 ± 0.9 mA for Sham, with no significant difference between conditions (t = 1.72, p = .104). In Experiment 2, mean intensities were of 1.4 ± 0.9 mA for active taVNS and 2 ± 1.2 mA for sham, with no significant difference between conditions (t = 1.31, p = .211). Additionally, mean intensities did not differ either between conditions across Experiments 1 & 2 (for taVNS: t = 0.80, p = .937; for sham: t = 0.36, p = 0.972).

In Experiment 2, mean stimulation durations were of 62.56 ± 10.92 min for active taVNS and of 57.70 ± 9.92 min for sham. Despite variable length of auricular stimulation across individuals (see Methods, *2*.*5 Time course*), stimulation durations were equivalent between taVNS and sham conditions (t = 1.16, p = .271).

In Experiment 3, current intensities for cVNS ranged from 0.75 mA to 2.0 mA (median: 1.25 mA), with stimulation frequencies ranging between 20 and 30 Hz (See Table 1).

### 2. Effects of VNS on behavioral responses (detection thresholds and intensity of perception)

Changes in skin temperature showed a weakly significant positive correlation to changes in heat-sensitive C-fibers detection threshold (p < .000, R^2^ = 0.135), a moderately significant positive correlation with the detection threshold of cool-sensitive free nerve endings (p < .000, R^2^ = 0.545), and a weakly significant negative correlation with mechano-sensitive Aβ-fiber detection threshold (p < .000, R^2^ = 0.163). There was no correlation between skin temperature and heat-sensitive Aδ-fibers detection threshold (p = .301).

With exception of a weakly significant positive correlation with intensity ratings to laser stimulations (p < .000, R^2^ = 0.040), changes in skin temperature did not influence intensity ratings to cool (p = .997), pinprick (p = .764), nor vibrotactile (p = .664) stimuli.

#### Before, during and after 3 hours of taVNS (Experiment 1)

**The detection threshold of heat-sensitive Aδ-fibers** was not significantly altered by our experimental procedure (no effect of factors ‘condition’, ‘time’, nor their 2-way interaction) (see Table 2).

**Table 2:** Linear mixed models with within-subject factors ‘condition’ and ‘time’: Detection thresholds and perception intensity (Experiment 1–3).

**Experiment 1**	Condition x Time				Condition				Time			
	F_1,125_ Value	P	CI 95%		F_1,125_ Value	P	CI 95%		F_1,125_ Value	P	CI 95%	
			Inf	Sup			Inf	Sup			Inf	Sup
**Detection Thresholds**												
Heat-sensitive C-fibers	0.17	0.842	-1.382	1.922	**6.14**	0.015*	-2.032	.304	0.42	0.657	-1.608	.728
Heat-sensitive Aδ-fibers	0.39	0.676	-1.445	2.194	0.02	0.887	-1.631	.942	0.18	0.837	-1.566	1.007
Mechanosensitive Aβ-fibers	0.27	0.761	-.005	.004	0.80	0.372	-.003	.003	2.794	0.064	-.001	.006
Cool sensitive fibers	0.002	0.998	-.863	.913	0.380	0.539	-.756	.501	**8.644**	0.000*	**-1.456**	**-.199**
**Perception Intensity**												
Laser	0.02	0.982	-1.616	1.358	0.01	0.916	-1.038	1.065	0.15	0.859	-1.090	1.013
Vibrotactile	0.25	0.776	-1.044	2.220	2.09	0.151	-1.942	.380	0.11	0.895	-1.264	1.030
Cool	0.69	0.505	-.873	1.742	0.57	0.452	-1.530	.319	2.74	0.069	-.673	1.178
Pinprick	0.09	0.915			0.56	0.457			0.15	0.861		
**Experiment 2**	Condition x Time				Condition				Time			
	F_1,52_ Value	P	CI 95%		F_1,52_ Value	P	CI 95%		F_1,52_ Value	P	CI 95%	
			Inf	Sup			Inf	Sup			Inf	Sup
**Detection Thresholds**												
Heat-sensitive C fibers	0.30	0.585	-1.636	2.868	0.69	0.410	-1.406	1.722	2.306	0.135	-1.077	2.165
Heat-sensitive Aδ-fibers	0.18	0.673	-2.423	1.577	1.454	0.233	-.576	2.201	0.01	0.912	-1.283	1.595
Mechanosensitive Aβ-fibers	0.80	0.376	-.010	.004	0.30	0.585	-.004	.006	0.04	0.836	-.003	.007
	Condition x Duty Cycle Phase				Condition				Duty Cycle Phase			
	F_1,56_ Value	P	CI 95%		F_1,56_ Value	P	CI 95%		F_1,56_ Value	P	CI 95%	
			Inf	Sup			Inf	Sup			Inf	Sup
**Perception Intensity**												
Laser	0.05	0.942	-1.880	2.023	0.094	0.760	-1.266	1.493	0.000	0.988	-1.423	1.337
Vibrotactile	0.01	0.974	-2.085	2.019	0.360	0.551	-1.741	1.160	0.031	0.861	-1.344	1.558
**Experiment 3**	/				/				Duty Cycle Phase			
									F Value	P	CI 95%	
											Inf	Sup
**Perception Intensity**												
Laser	/				/				0.05	0.819	-1.243	1.556
Vibrotactile	/				/				0.00	0.972	-2.383	2.304

Values were results obtained from Linear Mixed Models with within-subject factors ‘Condition’ (taVNS vs. sham) and ‘Time’ (before vs. during vs. after auricular stimulation in Experiment 1; before vs. during auricular stimulation in Experiment 2), as well as ‘Duty Cycle Phase’ (OFF vs. ON in Experiment 2 and 3). Model dimensions can be found in the S1 Appendix. A 2-way interaction between ‘Condition * Time’ indicates a differentiel effect on somatosensory perception of taVNS vs. sham stimulation at one specific timepoint of the experiment. A 2-way interaction between ‘Condition*Duty Cycle Phase’ indicates a differentiel effect on somatosensory perception of taVNS vs. sham stimulation, at a specific phase of the duty cycle. A main effect of ‘Time’ indicate an effect of the experimental design for both auricular conditions. A main effect of ‘Duty Cycle Phase’ indicates a differential effect of the active (ON, immediate response) or inactive (OFF, delayed response) phase of the duty cycle of either conditions (taVNS/sham or cVNS). Significant values are in bold.

*p < .05.

**Compared to sham session, the detection threshold of heat-sensitive C-fibers** was slightly higher during taVNS session (F = 6.14, p = 0.015), although no significant effect of ‘time’ (F = 0.42, p = .657) nor ‘condition’ x ‘time’ (F = 0.17, p = .842) was observed. Using a Bonferroni comparison for post-hoc analysis, this difference was only marginally significant (p = .043, mean difference ± SD between taVNS-Sham: 0.86 ± 1.88°C). However, when using a 1-way Anova with factor ‘condition’ to compare changes in heat-sensitive C-fibers threshold over time, this result lost significance when comparing ΔT0-T1 (F = 0.004, p = .947) and ΔT0-T2 (F = 1.052, P = .311). Similarly, the use of Bayesian repeated measures Anova further suggests mild evidence in favor of the lack of differential effects between auricular conditions (BF_01_ = 3.193). (see S1 Appendix – Bayesian Analyses).

**The detection threshold of cool-sensitive free nerve endings** was significantly altered by factor ‘time’ (F = 8.644, p = .000), however with no effect from the interaction of ‘condition’ x ‘time’ (F = 0.38, p = .539), nor from ‘condition’ itself (F = 0.00, p = .998) (see Table 2). Compared to baseline, post-hoc analyses with a Bonferroni comparison showed that the detection threshold for cool significantly decreased during and after 3 hours of auricular treatment (mean difference ± SD between T0-T1: 0.81 ± 2.24°C (p = .001); for T0-T2: 0.80 ± 0.23°C (p = .002)).

**The detection threshold of mechano-sensitive Aβ-fibers** was not significantly altered by our experimental procedure (no effect of factors ‘condition’, ‘time’, nor their 2-way interaction) (see Table 2).

**Intensity ratings to laser, vibrotactile, cool and mechanical stimuli** were not significantly altered by our experimental procedure (no effect of factors ‘condition’, ‘time’, nor their 2-way interaction) (see Table 2).

#### During and after 1h of taVNS (OFF vs. ON phases) (Experiment 2)

**Detection thresholds for heat-sensitive C-fibers, heat-sensitive Aδ-fibers and mechano-sensitive Aβ-fibers** were not altered by our experimental procedure (no effect of factors ‘condition’, ‘time’, nor their 2-way interaction, see Table 2).

**Intensity ratings to laser and vibrotactile stimuli** were not significantly altered by our experimental procedure (no effect of factors ‘condition’, ‘time’, nor their 2-way interaction, see Table 2).

#### During cVNS (OFF vs. ON phases) (Experiment 3)

**Intensity ratings to laser stimuli, as well as vibrotactile stimuli**, did not significantly differ between the OFF vs. ON phases of the duty cycle (See Table 2).

*a*. *Conclusion for behavioral responses*. Since heat-sensitive C-fibers detection threshold only marginally differed between auricular conditions in Experiment 1, this observation is likely to be a chance effect from imbalanced randomization on the order of taVNS vs. sham sessions.

**Fig 3:**
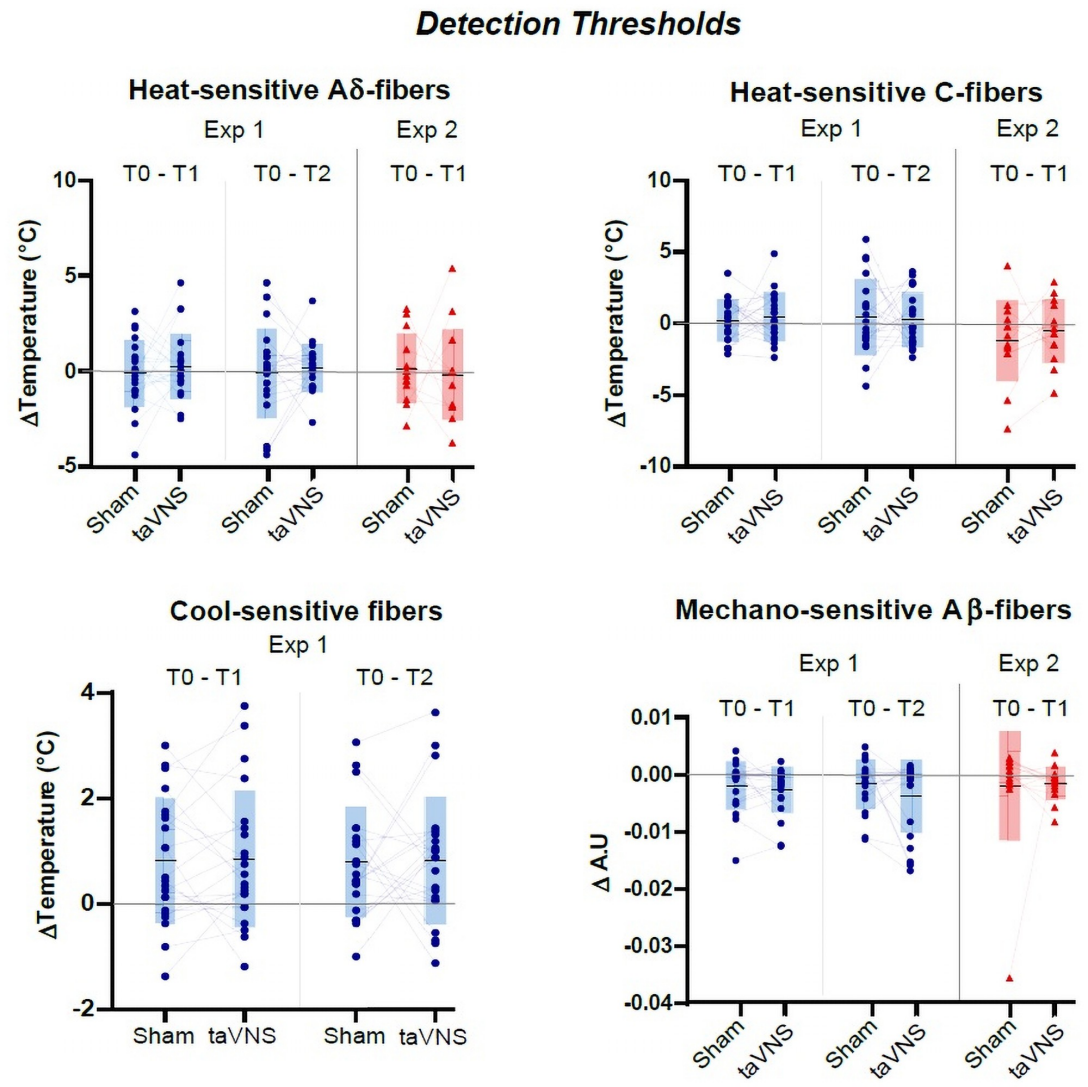
Detection thresholds. Each diagram represents the differences in detection thresholds between T0 and T1, or between T0 and T2, for one type of sensory fibers, under one experimental condition (taVNS vs. sham stimulation). Within each diagram, individual values are shape-coded according to the experiment in which they were retrieved (circles for Experiment 1, triangles for Experiment 2), with values from a same individual linked by a grey line. Grey boxes represent group statistics, with central horizontal lines and top/bottom extremities indicating the mean ± SD values. The Y axes represent the differences in detection thresholds between T0 vs. T1 or T0 vs. T2. Detection thresholds were measured in Celsius degrees (°C) for heat- and cool-sensitive fibers, and in an arbitrary unit (A.U) for mechano-sensitive A*β*-fibers. The X axes indicate which conditions are compared on the related Y axes. There was no significant alterations observed.

**Fig 4:**
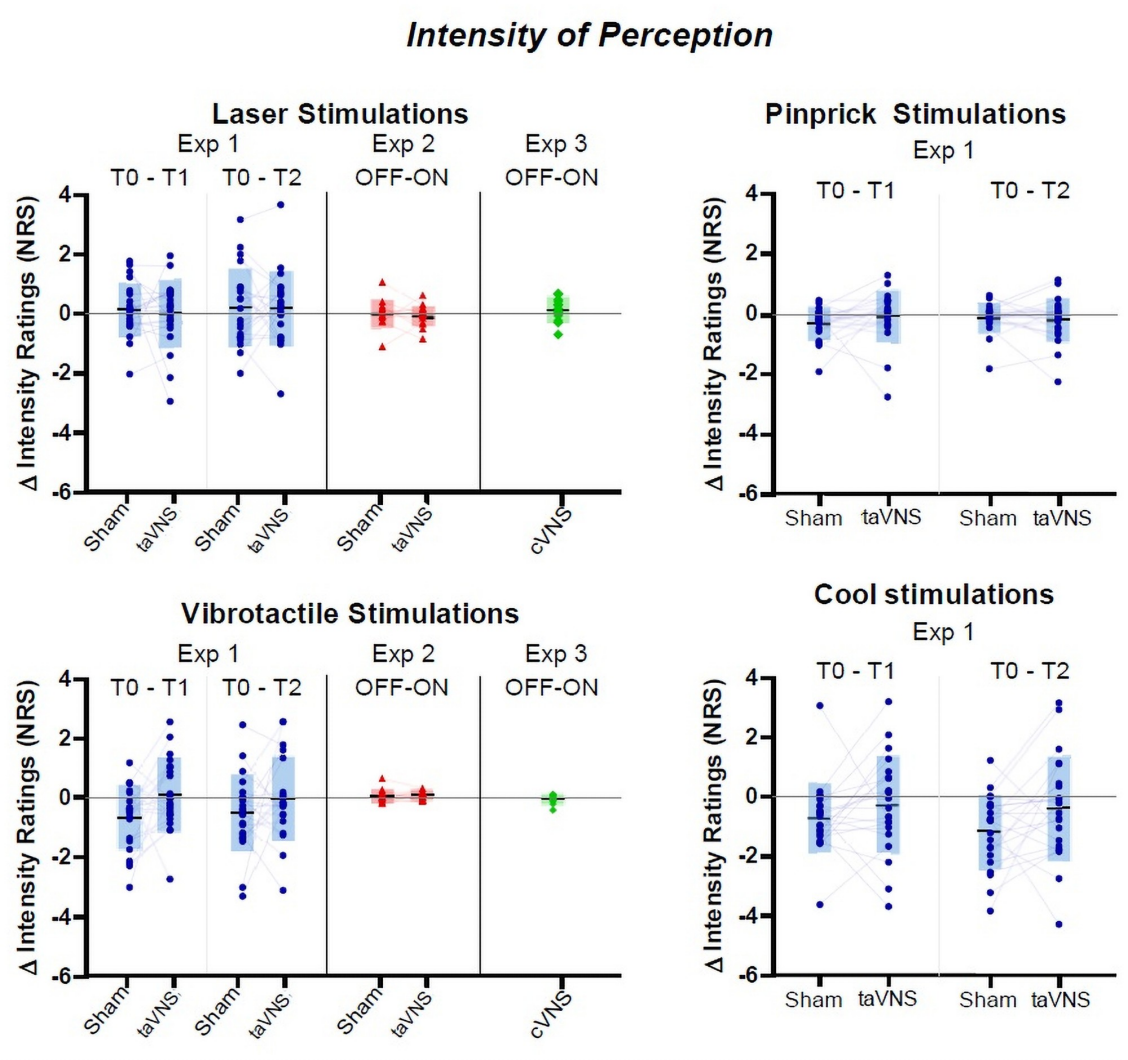
Behavioral responses: Intensity of perception. Each diagram represents the perceived intensity for one specific somatosensory modality, under different conditions (sham, taVNS or cVNS). Within each diagram, individual values are shape-coded according to the experiment in which they were retrieved (circles for Experiment 1, triangles for Experiment 2, diamonds for Experiment 3), with values from a same individual linked by a grey line. Grey boxes represent group statistics, with the central horizontal lines and top/bottom extremities indicating the means ± SD values. The Y axes represent the differences in intensity ratings between T0 vs. T1, T0 vs. T2 or OFF vs. ON phases. Intensity ratings were collected using a numerical rating scale (NRS) from 0 (no sensation) to 10 (maximal sensation). The X axes indicate which timepoints or phases of the duty cycle are compared on the related Y axes. There was no significant alterations observed.

We therefore conclude that there was neither prolonged, nor short-lasting effects of auricular stimulation on the behavioral responses elicited by various spinothalamic and lemniscal sensory stimuli. Likewise, the behavioral perceipt of spinothalamic and lemniscal stimuli were not affected by acute stimulation during chronic cVNS. (See Figs 3 and 4 for overview, Tables 3 and 4 for additional details on statistical analyses and measured values).

**Table 3:** Linear mixed models with within-subject factors ‘condition’: ΔDetection thresholds and ΔPerception intensity (Experiment 1–2).

**Experiment 1**	**ΔT0-T1**				**ΔT0-T2**			
	F_1,42_ Value	P	CI 95%		F_1,42_ Value	P	CI 95%	
**Detection Thresholds**								
Heat-sensitive C-fibers	0.328	.570	-1.221	.682	.059	.809	-1.218	1.551
Heat-sensitive Aδ-fibers	0.529	.471	-1.415	.665	1.351	.252	-2.039	.549
Mechanosensitive Aβ-fibers	0.309	.581	-.002	.003	1.167	.286	-.005	.005
Cool sensitive fibers	0.004	.947	-.778	.728	0.008	.930	-.725	.664
**Perception Intensity**								
Laser	0.180	.673	-.485	.744	0.001	.982	-.758	.775
Vibrotactile	**4.074**	.050*	-1.346	-.000	.937	.339	-1.163	.409
Cool	1.041	.313	-1.294	.425	2.880	.097	-1.690	.146
Pinprick	1.090	.302	-.658	.209	0.059	.809	-.332	.423
**Experiment 2**	**ΔT0-T1**							
	F_1,28_ Value	P	CI 95%					
**Detection Thresholds**								
Heat-sensitive C-fibers	0.422	.522	-2.625	1.366				
Heat-sensitive Aδ-fibers	0.155	.697	-1.364	2.009				
Mechanosensitive Aβ-fibers	1.367	.254	-.003	.010				
	**ΔOFF—ON**							
	F_1,28_ Value	P	CI 95%					
**Perception Intensity**								
Laser	0.239	.629	-.229	.372				
Vibrotactile	0.251	.620	-.168	.102				

Values were results obtained from Linear Mixed Models with within-subject factors ‘Condition’ (taVNS vs. sham). Model dimensions can be found in the S1 Appendix. A main effect of ‘Condition’ indicate a differential effect of taVNS vs. sham on the differences observed between recordings for detection thresholds/intensity ratings obtained a) at baseline vs. during auricular stimulation (ΔT0-T1), b) at baseline vs. after auricular stimulation (ΔT0-T2), c) during OFF vs. ON phase of the duty cycles (ΔOFF-ON). Significant values are in bold. *p < .05. To note, for Vibrotactile Intensity in Experiment 1: altough the difference observed between taVNS and sham conditions was weakly significant with a 1-way ANOVA (F = 4.074, p = .050, mean difference ± SE for taVNS and sham: 0.67 ± 0.33), this difference lost significance when using a paired bilateral t-student for post-hoc analysis (t = 1.965, p = .063, mean difference ± SD for taVNS and sham: 0.61 ± 1.42).

**Table 4:** Changes in detection thresholds and perception intensity (Experiment 1-2-3).

**Experiment 1 (mean ± SD)**	**ΔT0-T1**		**ΔT0-T2**	
	Sham	taVNS	Sham	taVNS
**Detection Thresholds**				
Heat-sensitive C-fibers (°C)	0.17 ± 1.46	0.44 ± 1.66	2.29 ± 9.19	0.24 ± 1.86
Heat-sensitive Aδ-fibers (°C)	-0.09 ± 1.72	0.28 ± 1.69	2.06 ± 10.24	0.68 ± 1.85
Mechanosensitive Aβ-fibers (A.U)	-0.0019 ± 0.0042	-0.0025 ± 0.0040	-0.0016 ± 0.0042	-0.0037 ± 0.0062
Cool sensitive Aδ-fibers (°C)	0.80 ± 1.18	0.83 ± 1.29	2.10 ± 6.28	0.81 ± 1.20
**Perception Intensity (NRS)**				
Laser	0.17 ± 0.89	0.04 ± 1.12	0.21 ± 1.23	0.20 ± 1.23
Vibrotactile	-0.68 ± 1.08	0.12 ± 1.19	-0.55 ± 1.28	-0.09 ± 1.36
Cool	-0.69 ± 1.17	-0.25 ± 1.62	-1.15 ± 1.23	-0.38 ± 1.74
Pinprick	-0.29 ± 0.53	-0.66 ± 0.86	-0.12 ± 0.46	-0.16 ± 0.61
**Experiment 2**	**ΔT0-T1**			
	Sham	taVNS		
**Detection Thresholds**				
Heat-sensitive C fibers	1.66 ± 11.41	2.26 ± 11.01		
Heat-sensitive Aδ-fibers	3.59 ± 13.50	3.15 ± 13.09		
Mechanosensitive Aβ-fibers	0.0017 ± 0.0095	-0.0014 ± 0.0028		
	**ΔOFF-ON**			
	Sham	taVNS		
**Perception Intensity**				
Laser	0.03 ± 0.45	-0.04 ± 0.35		
Vibrotactile	0.07 ± 0.21	0.11 ± 0.15		
**Experiment 3**	**ΔOFF-ON**			
**Perception Intensity**	cVNS			
Laser	0.16 ± 0.41			
Vibrotactile	-0.04 ± 0.15			

Values are group-level average ± SD representing the difference in Detection Thresholds or Perception Intensity between two timepoints (ΔT0-T1 or ΔT0-T2) or phases of the duty cyclce (ΔOFF-ON). Detection thresholds were obtained before (T0), during (T1) and after (T2) auricular stimulation in Experiment 1, before (T0) and during (T1) auricular stimulation in Experiment 2, as well as during (T1) cervical stimulation in Experiment 3. Detection Thresholds of heat- and cool-sensitive sensory fibers were obtained in Celsius°, while the detection threshold of mechanosensitive A*β*-fibers was obtained using a an Arbitrary Unit of a computerized scale (A.U). Perception intensity was retrieved during the OFF and ON phases of the duty cycle of auricular stimulation in Experiment 2, and cervical stimulation in Experiment 3. Perception intensity was assessed using a numerical rating scale (NRS), with no perception scored as 0 and maximal perception scored as 10, regardless of whether the sensation was painful or not. Symbols: Δ = difference between.

### 3. Effects of VNS on event-related brain potentials

#### Before and after 3 hours of taVNS (Experiment 1)

**Fig 5:**
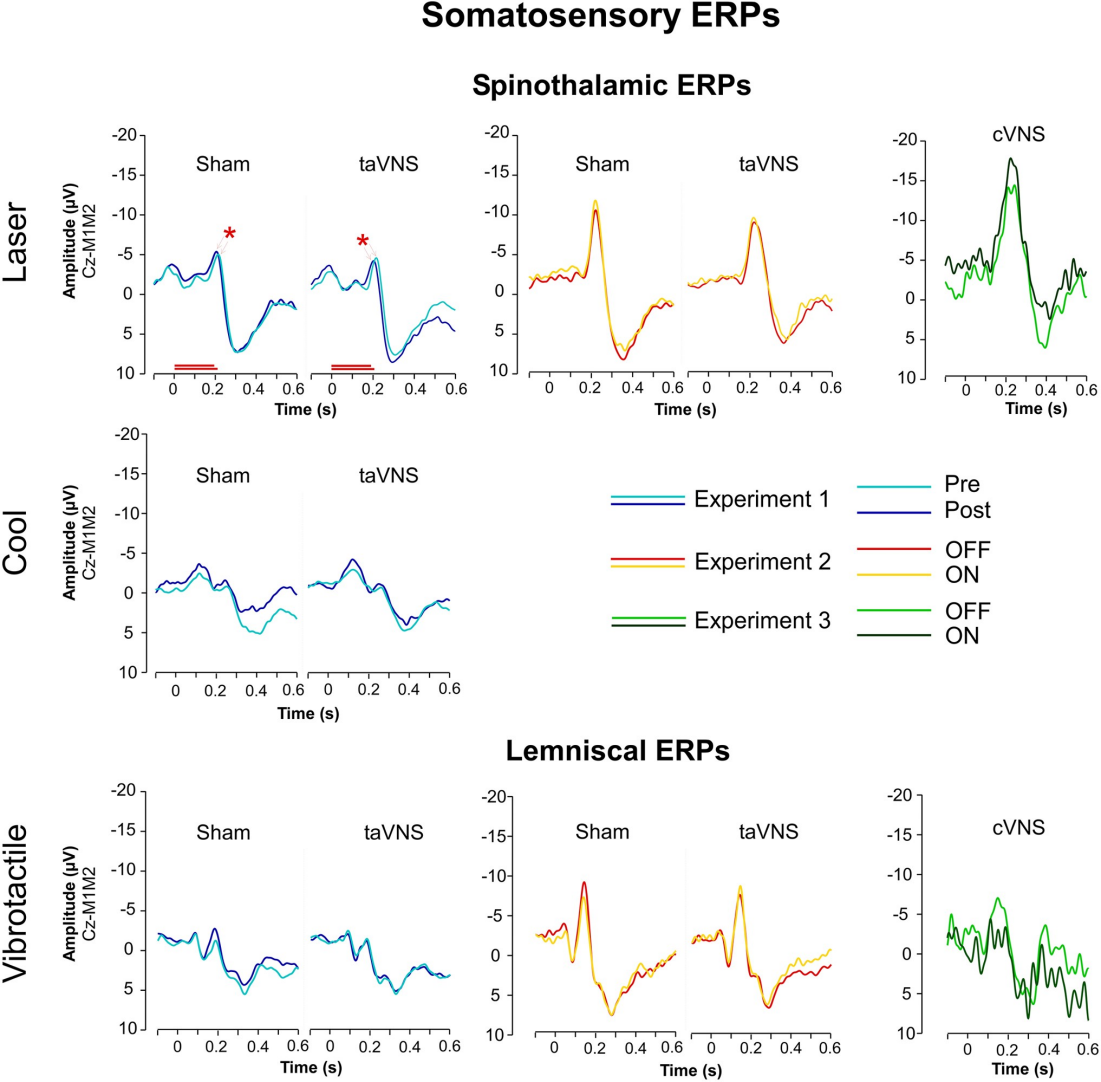
ERP waveforms. Each diagram represents the ERPs obtained at group level for one somatosensory modality and one experimental condition. The colors of the waveforms are specific to one experiment, one timepoint (T0 or T2) and one phase of the duty cycle (OFF or ON). The Y axes represent the amplitude of the difference in potentials (μV) observed at the Cz electrode, when referenced to the bilateral mastoid contacts (M1M2), with negative values at the upper end and positive values at the bottom of the axes. The X axes represent the evolution of time (in seconds) relative to the onset of the somatosensory stimulus (0s). The N2 peak was defined as the most negative deflection with a latency comprised between 0.1 to 0.5s. The P2 peak was defined as the first positive deflection after N2. The black circle and asterisk represent in Experiment 1, the statistically significant alteration observed over time in the latencies of the laser-evoked N2 peaks (p = .038). To note, the low signal to noise ratio in Experiment 1 (especially on Vibrotactile- and Cool-evoked ERPs obtained from healthy subjects) and Experiment 3 (epileptic patients).

**For Laser-evoked brain potentials**, a slight decrease in N2 latency was found over time (F = 4.46, p = .038), with no impact from factor ‘condition’ (F = 0.00, p = .980), nor between the interaction ‘condition’ x ‘time’ (F = 1.55, p = .216) (see Table 5). Using a Bonferroni comparison, post-hoc analysis showed a weakly significant decrease in N2 latency at T2 as compared with T0 (p = .038, mean difference ± SD: 0.012 ± 0.006 s) (Fig 5). At T0, the mean N2 latency is 0.218 ± 0.017s for taVNS, and 0.211 ± 0.032s for sham. At T2, the N2 latency is 0.199 ± 0.024s for taVNS, and 0.206 ± 0.029s for sham (see Table 6). However, when using a 1-way ANOVA with factor ‘condition’ for post-hoc analysis, this difference in N2 latency did not reach statistical significance (Δ T0-T2: F = 0.563, p = .461) (see Table 7). Beside this marginal change in latency, there was no significant change observed in the amplitude of the laser-evoked N2 peak (see Table 5). The amplitude and latency of the P2 peak, as well as global N2P2 amplitude of the laser-evoked ERPs, were unaffected by our experiment (see Tables 5 and 7).

**Table 5:** Linear mixed models with within-subject factors ‘condition’ and ‘time’: Cerebral responses (Experiment 1).

	Condition x Time				Condition				Time			
	F _1,82_ Value	P	CI 95%		F _1,82_ Value	P	CI 95%		F _1,82_ Value	P	CI 95%	
			Inf	Sup			Inf	Sup			Inf	Sup
**Laser-evoked ERPs**												
N2-P2 amplitude	0.05	0.826	-7.552	6.042	0.03	0.856	-4.740	4.872	0.14	0.709	-5.068	4.543
N2 amplitude	0.00	0.975	-3.757	3.876	0.08	0.778	-2.999	2.398	0.26	0.613	-2.241	3.157
N2 latency	1.55	0.216	-.008	.036	0.00	0.980	-0.228	.009	**4.46**	0.038*	**-.0346**	**-.003**
P2 amplitude	0.07	0.794	-5.984	4.593	0.02	0.663	-3.974	3.505	0.01	0.909	-3.544	3.935
P2 latency	0.21	0.648	-.045	.028	0.94	0.336	-.013	.039	0.29	0.594	-.026	.025
**Vibrotactile-evoked ERPs**												
N2-P2 amplitude	0.18	0.675	-4.755	3.096	0.15	0.700	-2.774	2.841	0.92	0.340	-3.308	2.244
N2 amplitude	0.26	0.589	-3.429	1.959	0.03	0.865	-1.675	2.179	0.34	0.561	-1.933	1.878
N2 latency	0.07	0.788	-.045	.034	0.15	0.697	-.030	.027	0.88	0.352	-.016	.040
P2 amplitude	0.56	0.458	-5.127	2.330	0.04	0.849	-2.147	3.188	1.80	0.183	-3.196	2.077
P2 latency	0.46	0.498	-0.467	.095	0.50	0.483	-.076	.027	1.41	0.239	-.084	.017
**Cool-evoked ERPs**												
N2-P2 amplitude	0.04	0.846	-4.692	3.855	0.33	0.568	-3.411	2.597	0.00	0.957	-2.889	3.190
N2 amplitude	0.19	0.667	-3.504	2.254	1.19	0.278	-.934	3.138	1.28	0.262	-2.565	1.554
N2 latency	1.47	0.229	-.0276	.114	0.61	0.437	-.058	.043	0.02	0.883	-.069	.031
P2 amplitude	0.08	0.773	-4.031	3.006	0.01	0.917	-2.325	2.651	1.67	0.200	-3.403	1.631
P2 latency	0.56	0.455	-.0894	.0404	0.29	0.588	-0.428	.0495	2.24	0.139	-.058	.034

Values were results obtained from Linear Mixed models with within-subject factors ‘Condition’ (taVNS vs. sham) and ‘Time’ (T0 vs. T2). Model dimensions can be found in the S1 Appendix. A 2-way interaction indicates a differential effect between taVNS vs. sham stimulation on one type of somatosensory modality after 3h of auricular stimulation. A main effect of ‘Time’ indicates an effect of the experimental design on one type of somatosensory modality, for both auricular conditions. Significant values are in bold, with p < .05 indicated by an asterisk (*).

**Table 6:** Cerebral responses (Experiment 1–3).

	Experiment 1				Experiment 2				Experiment 3	
	Sham		taVNS		sham		taVNS		cVNS	
	Pre	Post	Pre	Post	OFF	ON	OFF	ON	OFF	ON
**Laser-evoked ERPs**										
N2P2 amplitude (μV)	17.11 ± 9.88	16.10 ± 6.17	17.05 ± 7.93	16.79 ± 7.64	24.29 ± 11.96	24.26 ± 12.60	23.58 ± 11.00	21.36 ± 11.12	22.93 ± 15.62	26.85 ± 12.77
N2 amplitude (μV)	-6.82 ± 4.94	-6.30 ± 4.22	-6.52 ± 3.95	-6.06 ± 4.81	-12.03 ± 6.27	-12.68 ± 7.35	-13.85 ± 8.41	-12.61 ± 9.77	-15.08 ± 14.70	-17.66 ± 13.35
N2 latency (s)	0.21 ± 0.03	0.21 ± 0.03	0.22 ± 0.02	0.20 ± 0.02	0.23 ± 0.03	0.23 ± 0.04	0.24 ± 0.02	0.23 ± 0.02	0.26 ± 0.04	0.26 ± 0.07
P2 amplitude (μV)	10.30 ± 6.50	9.80 ± 5.81	10.53 ± 6.50	10.72 ± 6.12	12.26 ± 7.19	11.58 ± 6.91	9.73 ± 4.52	8.74 ± 6.31	7.86 ± 8.16	9.19 ± 4.91
P2 latency (s)	0.32 ± 0.04	0.32 ± 0.03	0.31 ± 0.05	0.31 ± 0.04	0.37 ± 0.05	0.36 ± 0.05	0.37 ± 0.06	0.36 ± 0.05	0.36 ± 0.09	0.37 ± 0.06
**Vibrotactile-evoked ERPs**										
N2P2 amplitude (μV)	11.22 ± 5.41	9.86 ± 4.34	11.19 ± 4.42	10.66 ±4.05	20.08 ± 6.57	17.89 ± 6.68	18.54 ± 5.71	17.72 ± 6.48	15.73 ± 5.54	19.68 ± 7.99
N2 amplitude (μV)	-3.21 ± 3.35	-3.97 ± 3.27	-3.46 ± 2.47	-3.49 ± 3.35	-9.12 ± 3.21	-7.45 ± 3.52	-8.41 ± 4.30	-8.47 ± 4.46	-7.28 ± 5.25	-8.09 ± 5.81
N2 latency (s)	0.17 ± 0.05	0.172 ± 0.03	0.166 ± 0.04	0.178 ± 0.06	0.14 ± 0.01	0.14 ± 0.01	0.14 ± 0.02	0.13 ± 0.03	0.18 ± 0.07	0.18 ± 0.06
P2 amplitude (μV)	8.20 ± 4.65	6.24 ± 5.13	7.67 ± 3.04	7.12 ± 4.24	10.96 ± 4.51	10.44 ± 4.35	10.13 ± 4.77	9.25 ± 5.09	8.45 ± 5.71	11.60 ± 8.36
P2 latency (s)	0.31 ± 0.09	0.300 ± 0.08	0.333 ± 0.08	0.300 ± 0.09	0.29 ± 0.06	0.28 ± 0.06	0.28 ± 0.05	0.29 ± 0.04	0.30 ± 0.09	0.33 ± 0.09
**Cool-evoked ERPs**										
N2P2 amplitude (μV)	10.753 ± 4.31	10.485 ± 4.39	11.160 ± 6.02	11.311 ± 5.13						
N2 amplitude (μV)	-3.943 ± 3.23	-5.073 ± 3.40	-5.045 ± 3.57	-5.550 ± 3.21						
N2 latency (s)	0.197 ± 0.08	0.221 ± 0.09	0.205 ± 0.10	0.186± 0.07						
P2 amplitude (μV)	6.810 ± 3.62	5.412 ± 4.27	6.647 ± 3.78	5.761 ± 4.66						
P2 latency (s)	0.371 ± 0.58	0.334 ± 0.07	0.367 ± 0.09	0.355 ± 0.09						

Values are group-level averages ± SD of the amplitude (μV) or latencies (s) of the N2 and P2 peaks of each types of somatosensory-evoked ERPs. Values were obtained before (T0) and after auricular stimulation (T2) in Experiment 1, while during the OFF and ON phases of the duty cycle for Experiment 2 and Experiment 3.

**Table 7:** Linear mixed models with within-subjects factors ‘condition’: ΔCerebral responses (Experiment 1–2).

	Experiment 1				Experiment 2			
	ΔT0-T2				ΔOFF-ON			
	F_1,42_Value	P	CI 95%		F_1,28_ Value	P	CI 95%	
			Inf	Sup			Inf	Sup
**Laser-evoked ERPs**								
N2-P2 amplitude	0.071	.791	-4.959	6.469	2.150	.154	-5.261	.871
N2 amplitude	0.001	.973	-3.586	3.468	1.944	.174	-.885	4.661
N2 latency	0.993	.325	-.039	.013	0.525	.475	-.017	.008
P2 amplitude	0.165	.687	-2.764	4.156	0.089	.768	-2.420	1.806
P2 latency	0.632	.429	-.015	.034	0.140	.711	-.025	.036
**Vibrotactile-evoked ERPs**								
N2-P2 amplitude	0.459	.502	-1.556	3.126	1.897	.179	-.670	3.419
N2 amplitude	0.694	.410	-1.182	2.840	**5.215**	.030*	**-3.290**	**-.178**
N2 latency	0.046	.831	-.025	.031	0.430	.517	-.025	.013
P2 amplitude	1.423	.240	-.992	3.849	0.195	.662	-2.029	1.310
P2 latency	1.028	.317	-.069	.023	1.234	.276	-.014	.048
**Cool-evoked ERPs**								
N2-P2 amplitude	0.008	.931	-2.716	2.490				
N2 amplitude	0.347	.559	-1.573	2.867				
N2 latency	3.669	.062	-.107	.003				
P2 amplitude	0.186	.668	-1.800	2.776				
P2 latency	0.052	.820	-.060	.075				

Values were results obtained from Linear Mixed models with within-subject factors ‘Condition’ (taVNS vs. sham) when assessing the differences in ERP waveforms between two timepoints (ΔT0-T2) or two phases of the duty cycle (ΔOFF-ON). Model dimensions can be found in the S1 Appendix. A main effect of ‘Condition’ indicates a differential effect of tVNS vs. sham stimulation on the recorded difference between T0 vs. T2, or OFF vs. ON phases. Significant values are in bold, with p < .05 indicated by an asterisk (*).

**For Cool-evoked ERPs, as well as Vibrotactile-evoked ERPs**, there were no significant alterations after 3 hours of taVNS/sham stimulation (See Table 5). Note the low signal to noise ratio in the averaged ERP waveforms (see Fig 5).

**Fig 6:**
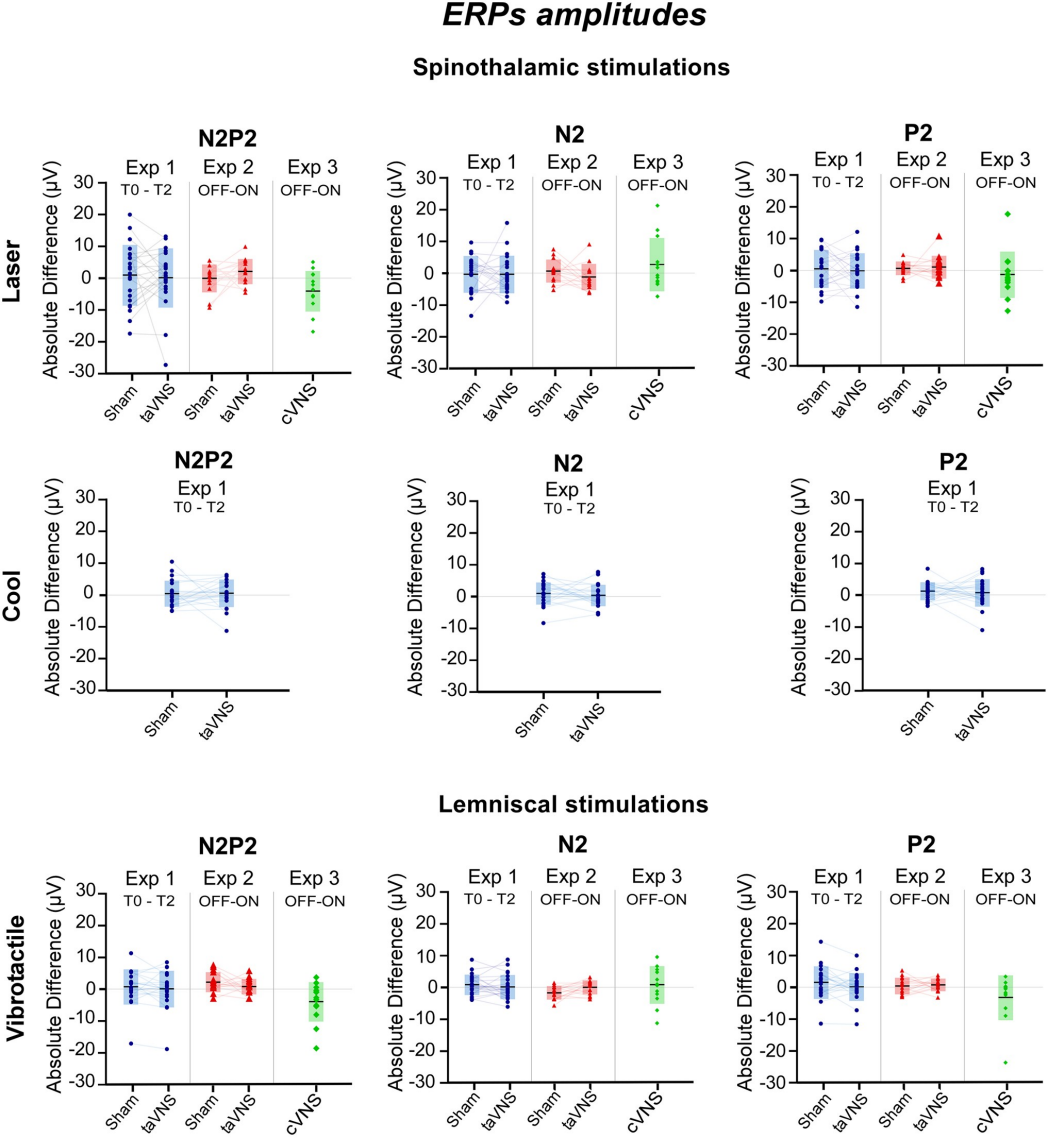
ERPs: Peak amplitudes. Each diagram represents the absolute difference in the amplitudes of specific peaks (N2,P2,N2P2 amplitude) of one type of somatosensory-ERP (Laser-, Vibrotactile-, Cool-ERPs), recorded at a specific timepoint (T0,T2) or phase of the duty cycle (OFF,ON) under one experimental condition (sham, taVNS or cVNS). Within each diagram, individual values are shape-coded according to the experiment in which they were retrieved (circles for Experiment 1, triangles for Experiment 2, diamonds for Experiment 3). Grey boxes represent group statistics, with the central horizontal lines and top/bottom extremities indicating the means ± SD values. The Y axes represent the absolute difference (in μV) measured between two recordings of a specific peak. The X axes indicate which timepoints or phases of the duty cycle are compared on the related Y axes. There were no statistically significant results obtained.

**During taVNS (OFF vs. ON phases) (Experiment 2). Laser- and Vibrotactile-evoked ERPs** were not affected by the duty cycle of either taVNS or sham stimulation (see Table 8). However, a post-hoc analysis was nevertheless conducted as we could visually observe a difference between the OFF vs. ON phases on the waveforms obtained under both auricular conditions (see Fig 5). Using a 1-way Anova with factor ‘condition’ to assess the differences between the OFF vs. ON phases, changes in the N2 amplitude of the vibrotactile-evoked ERPs appeared to be significantly larger under sham stimulation as compared with taVNS (ΔOFF-ON: F = 5.215, p = .030) (see Table 7, Figs 5 and 6). This differential effect of taVNS and sham conditions was further confirmed with mild evidence under Bayesian statistics (BF_01_ = 0.156) (See Supplementary Data, pg 36).

**Table 8:** Linear mixed models with within-subject factors condition and duty cycle phase: Cerebral responses (Experiment 2–3).

	Experiment 2												Experiment 3			
	Condition x Duty Cycle Phase				Condition				Duty Cycle Phase				Duty Cycle Phase			
	F_1,56_ Value	P	CI 95%		F_1,56_ Value	P	CI 95%		F_1,56_ Value	P	CI 95%		F_1,24_	P	CI 95%	
			Inf	Sup			Inf	Sup			Inf	Sup	value		Inf	Sup
**Laser-evoked ERPs**																
N2-P2 amplitude	0.13	0.718	-14.287	9.898	0.36	0.552	-5.649	11.453	0.14	0.711	-6.328	10.774	0.489	0.491	-15.463	7.636
N2 amplitude	0.21	0.652	-6.443	10.218	0.18	0.674	-5.955	5.826	0.02	0.887	-7.130	4.651	0.220	0.643	-8.777	13.947
N2 latency	0.08	0.772	-.035	.026	1.96	0.167	-.030	.013	0.94	0.337	-.012	.031	0.008	0.931	-.048	.044
P2 amplitude	0.01	0.925	-6.845	6.230	2.71	0.106	-1.785	7.460	0.26	0.613	-3.639	5.606	0.253	0.620	-6.782	4.125
P2 latency	0.04	0.836	-.048	.059	0.06	0.805	-.044	.031	0.50	0.483	-.031	.044	0.123	0.729	-.073	.052
**Vibrotactile-evoked ERPs**																
N2-P2 amplitude	0.18	0.678	-5.216	7.965	0.27	0.604	-3.843	5.477	0.84	0.364	-4.489	4.831	2.150	0.156	-9.516	1.611
N2 amplitude	0.74	0.394	-5.778	2.309	0.02	0.879	-1.838	3.880	0.64	0.427	-2.799	2.919	0.139	0.713	-3.671	5.291
N2 latency	0.36	0.552	-.026	.014	1.78	0.187	-.004	.024	0.15	0.699	-.009	.019	0.000	0.989	-.052	.052
P2 amplitude	0.02	0.882	-5.211	4.492	0.70	0.407	-2.239	4.623	0.33	0.567	-2.554	4.307	1.253	0.274	-8.938	2.652
P2 latency	0.35	0.555	-.040	.073	0.01	0.938	-.049	.030	0.00	0.979	-.048	.032	0.890	0.355	-.101	.038

Values were results obtained from Linear Mixed models with within-subject factors ‘Condition’ (taVNS vs. sham) and ‘Duty Cycle Phase’ (OFF vs. ON). Model dimensions can be found in the S1 Appendix. A 2-way interaction indicates a differentiel effect between taVNS vs. sham stimulation for one type of somatosensory-evoked ERPs, at a specific phase of the duty cycle. Given the design of the experiment, a main effect of ‘Condition’ suggests a differential effect of taVNS vs. sham stimulation on one type of somatosensory evoked-ERPs. A main effect of ‘Duty Cycle Phase’ indicates a differential effect of the active phase (ON, immediate response) vs. inactive (OFF, delayed response) phase of the duty cycle on one type of somatosensory evoked-ERPs, regardless of auricular conditions. Significant values are in bold, with with p < .05 indicated by an asterisk (*).

**During cVNS (OFF vs. ON Phases) (Experiment 3). Laser-evoked and Vibrotactile-evoked ERPs** were not affected by the duty cycle of chronic cVNS (see Table 8). However, the reader might note the low signal to noise ratio in the averaged ERP waveforms (see Fig 5).

*a*. *Conclusion for cerebral responses*. Although our 3 experiments tested different timescale analyses and devices, cerebral responses to laser, cool and vibrotactile stimuli were not significantly altered by taVNS, sham stimulation nor cVNS (see Fig 6 and Table 6 for details).

## Discussion

### 1. Summary of results

Our study aimed to examine how VNS affects the nociceptive system, by combining both psychophysical (detection thresholds, intensity ratings) and electrophysiological (spinothalamic- and lemniscal-evoked ERPs) responses to pain perception. Although neither ERPs nor behavioral testings are entirely specific to pain perception, their combination offers a complementary exploration of the neural representation underlying nociceptive processing [69].

In contrast to the multiple data from the literature showing the analgesic, but also pro-nociceptive properties of taVNS and cVNS, our experiments did not show any significant effect of VNS on pain perception, nor on the elicited spinothalamic-evoked ERPs, despite the different timescales analyzed. Although our study is the first to examine the effect of taVNS on spinothalamic and lemniscal ERPs combined with their behavioral correlates, our results are in contrast with other human studies in which taVNS was found to modulate behavioral responses elicited by the application of various spinothalamic stimuli [5, 54, 62–65]. In our study, we observed neither effect following 3h of taVNS (Experiment 1), nor short-lasting effect from the ON and OFF phases of its duty cycle (Experiment 2).

To address the potential lack of effectiveness of taVNS, we performed a third experiment in which we tested the acute effects of cVNS in implanted epileptic patients, which remains the “golden standard” in terms of vagus nerve stimulation (Experiment 3). In comparison with healthy subjects, the ERPs obtained from epileptic patients were of considerable poorer quality (see Fig 4), which could be explained by the attentional impairment commonly seen in epileptic patients [80]. In our hands however, cVNS did not alter pain perception when comparing the short-lasting effects of the ON and OFF phases of its duty cycle.

### 2. Comparison with other studies

Our experiments focused on ERPs elicited from thermonociceptive heat, which is the most studied noxious modality reported with taVNS [55, 62, 64]. To maximize the signal-to-noise ratio of our time-locked ERPs [81], we used a CO_2_ laser to deliver transient heat pulses. However, other groups reported using a contact thermode to deliver tonic heat pain [55, 62, 64] and observed decreased pain ratings [62, 64], as well as bidirectional alterations in pain thresholds [55], when pairing taVNS to noxious heat application. Similarly, the anti- and pro-nociceptive effects of taVNS were also observed under tonic electrical stimulation [54]. Thus, our choice of stimulus delivery might partially explain our contrasting results. While transient laser pulses mostly trigger fast-adapting fibers, tonic stimulation might better activate slowly adapting sensory fibers [82]. In addition, tonic stimulation might engage central sensitization processes [62, 83], whereas sensitization is less likely to occur with transient heat pulses. Since the vagus nerve is known to act on different anatomical levels (from both peripheral and central pathways) [84, 85], the induction of sensitization could be an important factor to consider when evaluating taVNS [5, 62, 64].

Nevertheless, our material differences cannot account for all the divergences observed between our study and the literature. For instance, while Busch et al reported an increase of mechanical pain threshold and a reduction of mechanical pain sensitivity under taVNS in healthy subjects [62], we did not find an effect of taVNS on mechanical pain sensitivity in our healthy participants. This difference cannot be explained by our use of a 128mN pinprick stimulus, as this stimulation intensity was not much different from their reported mechanical pain thresholds (150–200 mN).

Another difference to consider is that contrarily to our stimulation on the left cymba conchae, Busch et al delivered taVNS on the inner side of the tragus. Which auricular placement is most effective for taVNS is still a matter of debate, as the anatomy of the ABVN remains obscure [86]. While the cymba conchae might be predominantly innervated by the ABVN and the auriculo-temporal branch of the trigeminal nerve [59], the tragus might benefit from additional innervation from the greater auricular nerve of the cervical plexus [58, 87]. Moreover, regardless of the origins of the nervous fibers, the density of epidermal innervation was recently shown to be lower in the anterior-inferior wall of the ear canal (adjacent to the tragus), when compared to the superior and lower-posterior portion of the ear canal (prolonging the conchae) [88]. To date, an fMRI study further indicated that activation of the nucleus tractus solitarius, the primary relay of vagal afferents at brainstem level [89], was stronger when taVNS was conducted at the cymba conchae as compared to stimulation on the tragus [26, 27].

Anatomy aside, the use of continuous vs. intermittent acute taVNS might also induce differential observations [90, 91]. This programming difference might further explain the discrepancy observed between the absence of effect of intermittent taVNS in our hands, as compared to the observed analgesic effect on mechanical [62], thermal [62] and electrical pains [54] described in the literature with continuous taVNS. Although direct exploration of these programming effects is lacking, this question remains important as the rationale to use taVNS in pain conditions is mainly based on fMRI studies conducted with continuous taVNS [25–27, 55], while clinical practice traditionally favored intermittent VNS for better treatment tolerance.

In the same line of thought, how to parameter taVNS adequately for pain management remains unknown [54]. For instance, while our study and several others relied on the empirical 25-30Hz frequency proven useful with cVNS in epilepsy disorders [5, 62, 63, 65], other researchers opted for stimulation frequencies of either 2Hz [50] or alternating bursts of 100Hz/2Hz [54, 64], which were proven effective in chronic pain treatment with transcutaneous electrical nerve stimulation (TENS) or electro-acupuncture [92].

The length of stimulation to observe an effect of VNS on pain perception is also unclear. While we found no effect of taVNS/cVNS despite our multiple timescale analyses, others reported alteration in pain perception within 1h of taVNS in healthy subjects (under 25min at shortest [63]). Experiments in animals have showed that acute VNS was sufficient to induce an analgesic effect within seconds [1]. In humans however, there was no acute effect of cVNS found when assessing experimental pain in implanted epileptic patients [51, 53], suggesting that the underlying mechanisms might be different across species. Accordingly, in seizure therapy, the anti-epileptic effects of cVNS appear within 6 months of usage, usually to increase over time [93]. Similarly, in migraine prevention, taVNS effectively reduced migraine attacks when used 4h daily during 12 weeks [3]. These results indicate a potential, cumulative effect of neuromodulation, probably not obtained with immediate stimulation. Therefore, based on previous literature [3, 93], we cannot exclude that longer taVNS durations are needed to alter nociception.

Likewise, the optimal washout period to observe between two auricular sessions remains speculative. Although the 48h interval in our experiment was chosen in accordance with previous studies [62, 64], this timeframe remains however relatively short. Hence, a carry-over effect between taVNS and sham stimulation cannot be ruled out. Future studies relying on a cross-over design might thus benefit from longer washout periods between two experimental sessions.

Which fiber population within the vagus nerve is responsible for pain modulation is also unknown. In newborn rats, systemic capsaicin treatment was found to decrease the spinal inhibition produced by high intensity VNS, suggesting that afferent C-fibers activation might be necessary to induce anti-nociceptive effects [94]. In humans though, based on epileptic patients implanted with cVNS in whom the antinociceptive effects were first reported, the clinical effects of VNS are suggested to be mediated by Aβ-fibers [95, 96], accounting for approximately 20–30% of vagal fibers at cervical location [97]. The most common intensity titration for taVNS is therefore based on this premise, with maximal, sub-painful current intensity aimed at recruiting the Aβ-fibers of the sensory ABVN [72]. However, the ABVN appears to have approximately 6 times less Aβ-fibers in comparison to the cervical trunk of the vagus nerve [70], while the proportion of C-fibers is unknown [70, 71]. Thus, whether the usual intensity titration for taVNS is truly sufficient to alter central processes of pain perception [50], beyond the simple activation of somatosensory pathways (as observed on the fMRI [17–28]), remains unknown.

As a consequence, the current density delivered into vagal fibers is yet another area to explore, with potential correlation to the opposite pro- or anti-nociceptive characteristics of VNS reported at present [1, 53, 54]. As observed in early animal studies [35, 98] and a human report [53], low-intensity VNS increased pain perception, while higher intensities of stimulation led to an inhibitory, analgesic effect. In this context of fiber activation, while we used unilateral taVNS in agreement with other studies conducted with the Nemos’ device [62, 63, 65], other researchers favored the use of bilateral taVNS [54, 55, 64], possibly leading to increased fiber activation and stimulation efficacy. Indeed, cortical and hippocampal releases of noradrenalin, a key neurotransmitter underlying the therapeutical effects of VNS, are known to vary depending on current intensity [36]. Hence, while our choice was primarily motivated by experimental homogeneicity with our epileptic patients implanted with a left cVNS (Experiment 3), it is not excluded that the summative current density obtained with bilateral taVNS may modulate nociceptive ERPS’s differently as compared to unilateral taVNS.

Finally, considering Experiment 3, the long-lasting effects of cVNS could not be addressed in our study, as patients were chronically implanted with the device without baseline testing performed prior to their implantation. Given that the neuromodulatory effects of VNS increase after longer periods of stimulation [3, 93], our negative results could come from the possibility that chronic cVNS evened the specific effects of the OFF vs. ON periods. Despite this limitation, our findings are in line with the prospective report of Kirchner et al [51], in which the authors found no correlation between the acute ON-OFF cycles of cVNS and pain relief, while 14 weeks of cVNS led to decreased tolerance to repetitive noxious heat and tonic pressure pain as compared to baseline recordings.

### 3. Limitations

Despite our efforts, the signal to noise ratio (SNR) of our ERP recordings in Experiment 1 was rather low, especially for the non-nociceptive modalities (see Fig 5). As described in Methods (*2*. *Experiment 2*), only Laser- and Vibrotactile-evoked ERPs were reconducted in Experiment 2 and 3 with optimized stimulation parameters to improve the EEG recordings. As described earlier, the ERP recordings from our epileptic patients (Experiment 3) also came surprisingly with a low signal to noise ratio, even in spite of the improved somatosensory stimulation. As a correlate to the poor SNR, the dispersion of the individual data gathered into the group-level average can be visualized in Fig 6, with higher inter-individual variability in both Experiment 1 and 3 as compared to Experiment 2. For transparency, the individual averaged waveforms were uploaded in our OSF library (https://osf.io/2db3x/). With this in mind, whether or not this high inter-individual variability affected the apparent lack of cerebral effects of 3h of taVNS (Experiment 1) and the ON/OFF phases of chronic cVNS (Experiment 3) cannot be ruled out.

Secondly, the sample sizes in our experiments were determined a priori based on previous studies (see Methods, *1*.*7 Statistical Analysis*), in which large effect sizes of *η*_*p*_^*2*^ > 0.2 were reported for the analgesic effects of taVNS [62, 64]. However, our null results raised questions towards a potential lack of statistical power in our work and/or reports of overpowered effect sizes. To quantify the null, we provided a listing of complementary Bayesian analysis in our S1 Appendix. Overall, for Experiment 1, our analyses suggest mild evidence towards the absence of effect (BF_01_ > 3 in favor of the null model, especially for detection thresholds). Results for Experiment 2 and 3 were less interpretable, as the obtained Bayesian Factors were between 0.3 and 3 (see S1 Appendix Analysis). – Bayesian These post-hoc analyses could suggest that the real effect size of taVNS might be milder than what is currently reported in the literature [99]. Furthermore, if the likelihood of a small effect size is high, the null hypothesis model itself should too be considered with caution, as in this context, our Bayesian analyses might lead to 80% chances of wrongly favoring the null model [99, 100]. This might be true for our data, especially when the obtained signal to noise ratio was low (ERP recordings in Experiment 1 and 3). Hence, although it is difficult to draw strong conclusions directly from our work, increasing power and sample sizes of future studies might help disclose a potential smaller effect of taVNS.

Although not directly addressed in our study, our inability to tailor the effectiveness of taVNS at individual level was indeed limitative. We were unable to determine whether taVNS activated the vagus nerve in a physiological manner similar to cVNS, and to what extent a lack of vagal activation could explain our null results. Without scientific consensus on an objective biomarker reflecting the activation of the vagus nerve under taVNS, this is likely to remain a serious challenge. Although vagal evoked potentials appeared promising [101–103], they were later demonstrated to be from muscular origin [85, 104]. Potential alternatives include the recording of heart rate variability [71, 105], gastric mobility [63, 106, 107], pupil size and alpha amylase concentrations [108, 109]. Similarly, another limitation was the absence of auricular biometrics, such as ear sizes, skin properties (conductance, water and fat content), as well as vascular density and axonal distribution. Each of these measures could explain inter-individual variability in current fields distribution, therefore impacting the net current delivery at the targeted ABVN and its physiological translates [85, 109–111].

The use of earlobe stimulation as sham condition is also far from ideal [26, 28, 112]. Because the earlobe receives innervation from the greater auricular nerve of the cervical plexus, specifically from the C2 and C3 spinal roots [61], this sham condition could be far from inactive. Although the patterns of central activation do seem to differ between cymba conchae and earlobe stimulations, their respective effects still overlap in certain cerebral and brainstem regions such as the limbic system and the locus coeruleus [26, 28]. This finding calls for caution, even more so as that it was not excluded for the earlobe to shelter partial vagal innervation [58, 59]. Despite these anatomical considerations, we chose to use our sham stimulation based on the availability of multiple fMRI studies at this location [25–27]. While our choice was limited to the present context, the fast evolving literature might help identify and characterize better alternatives for sham conditions, such as the posterior scapha for instance [112].

Last, our study lacked the use of mood questionnaires. The pain-related effects of taVNS appear predominantly on tonic stimuli, which are known to produce a temporal increase of pain perception mostly by alterations in the affective components of the pain experience, rather than that of sensory-processing [113]. Traditionally, the vagus nerve is also highly investigated for its therapeutical effect on mood regulation [29] and was suggested to relay the emotional aspect of pain perception [84, 114]. Hence, while acute taVNS can be useful to primarily explore the sensory modulation related to pain perception, the chronic use of taVNS might offer complementary insight into the changes induced on the subjective component of the pain experience [115].

### 4. Future perspectives

To further understand how VNS influences the peripheral nociceptive system, future studies could include the use of ultra-late responses in laser-evoked ERPs, a cerebral correlate which better reflects the activity of heat-sensitive C-fibers nociceptors [116]. Investigating whether VNS modulates the activity of peripheral slow-adapting fibers might also benefit our current understanding [82].

Depending on the outcomes of such studies, the results may lead to the identification of a marker of taVNS-derived brain modulation, potentially useful in other areas of VNS application.

## Conclusion

To the best of our knowledge, our study is the first to report the complementary use of quantitative sensory testing to thermonociceptive heat and laser-evoked ERPs, with aim to explore how VNS modulates pain perception. In our hands, VNS did not alter pain perception at electrophysiological level (laser-evoked ERPs), nor at behavioral level (detection thresholds, perception intensity). Such negative results might be explained by the inability to monitor the effectiveness of the device, the inter-individual anatomical variability in ear size and innervation, as well as a lack of standardization in taVNS procedures.

## Supporting information

S1 Appendix (ZIP)
