# Supplementary material for: Transcutaneous auricular VNS applied to experimental pain: A paired behavioral and EEG study using thermonociceptive CO2 laser
Source: PLoS One. 2021 Jul 12;16(7):e0254480. doi: 10.1371/journal.pone.0254480 (PMC8274876; doi:10.1371/journal.pone.0254480)
Supplement: S1 Appendix — (ZIP) [file pone.0254480.s001.zip › Supplementary Analysis_LMM_Behavioral responses DIFF.pdf]

Transcutaneous VNS applied to experimental pain: a paired behavioral and EEG study using thermonociceptive CO2 laser

Supplementary Appendix

Linear Mixed Models: Detailed analysis.

Δ BEHAVIORAL RESPONSES

1. Experiment 1:

1.1. Heat-sensitive C-fibers Detection Thresholds (ΔT0-T1).

```
MIXED HeatsensitiveCfibersThresholdsΔT0T1 BY Condition
  /CRITERIA=CIN(95) MXITER(100) MXSTEP(10) SCORING(1)
SINGULAR(0.000000000001) HCONVERGE(0,
    ABSOLUTE) LCONVERGE(0, ABSOLUTE) PCONVERGE(0.000001, ABSOLUTE)
  /FIXED=Condition | SSTYPE(3)
  /METHOD=REML
  /PRINT=CPS CORB COVB DESCRIPTIVES G  SOLUTION TESTCOV
  /EMMEANS=TABLES(Condition) COMPARE ADJ(BONFERRONI).
```

Remarques

Sortie obtenue		04-MAY-2021 15:45:26
Commentaires		
Entrée	Jeu de données actif	Jeu_de_données1
	Filtre	<sans>
	Pondération	<sans>
	Fichier scindé	<sans>
	N de lignes dans le fichier de travail	44
Gestion des valeurs manquantes	Définition de la valeur manquante	Les valeurs manquantes définies par l'utilisateur sont traitées comme étant manquantes.
	Observations utilisées	Les statistiques sont basées sur toutes les observations comportant des données valides pour toutes les variables du modèle.

Syntaxe		MIXED HeatsensitiveCfibersThresholdsΔT0T1 BY Condition<br>/CRITERIA=CIN(95) MXITER(100) MXSTEP(10) SCORING(1) SINGULAR(0.000000000001) HCONVERGE(0, ABSOLUTE) LCONVERGE(0, ABSOLUTE) PCONVERGE(0.000001, ABSOLUTE)<br>/FIXED=Condition | SSTYPE(3)<br>/METHOD=REML<br>/PRINT=CPS CORB COVB DESCRIPTIVES G SOLUTION TESTCOV<br>/EMMEANS=TABLES(Condition) COMPARE ADJ(BONFERRONI).
Ressources	Temps de processeur	00:00:00,00
	Temps écoulé	00:00:00,01

Récapitulatif de traitement des observations

		Effectif	Pourcentage marginal
Condition	Sham	22	50,0%
	taVNS	22	50,0%
Valide		44	100,0%
Exclues		0	
Total		44	

Statistiques descriptives

Heat-sensitive C-fibers Thresholds ΔT0-T1

Condition	Effectif	Moyenne	Ecart type	Coefficient de variation
Sham	22	,170455	1,4600562	856,6%
taVNS	22	,440341	1,6615036	377,3%

Total	44	,305398	1,5517488	508,1%

Dimension du modèle[a]

		Nombre de niveaux	Nombre de paramètres
Effets fixes	Constante	1	1
	Condition	2	1
Résidu			1
Total		3	3

a. Variable dépendante : Heat-sensitive C-fibers Thresholds ΔT0-T1.

Critères d'information[a]

Log de vraisemblance restreint -2	162,943
Critère d'information d'Akaike (AIC)	164,943
Critère de Hurvich et Tsai (AICC)	165,043
Critère de Bozdogan (CAIC)	167,681
Critère bayésien de Schwartz (BIC)	166,681

Les critères d'informations sont présentés en plus petit, disposant d'un meilleur format.[a]

a. Variable dépendante : Heat-sensitive C-fibers Thresholds ΔT0-T1.

Effets fixes

Tests des effets fixes de type III[a]

Source	Ddl du numérateur	Ddl du dénominateur	F	Sig.
Constante	1	42	1,678	,202
Condition	1	42	,328	,570

a. Variable dépendante : Heat-sensitive C-fibers Thresholds ΔT0-T1.

Estimations des effets fixes[a]

Paramètre	Estimation	Erreur standard	ddl	t	Sig.	Intervalle de confiance à 95 % Borne inférieure
Constante	,440341	,333452	42	1,321	,194	-,232592
[Condition=Sham]	-,269886	,471572	42	-,572	,570	-1,221557
[Condition=taVNS]	0[b]	0	.	.	.	.

Estimations des effets fixes[a]

Paramètre	Intervalle de confiance à 95 % Borne supérieure
Constante	1,113273
[Condition=Sham]	,681784
[Condition=taVNS]	.

a. Variable dépendante : Heat-sensitive C-fibers Thresholds ΔT0-T1.

b. Ce paramètre est défini sur 0, car il est redondant.

Matrice de corrélation pour les estimations des effets fixes[a]

Paramètre	Constante	[Condition=Sham]	[Condition=taVNS]
Constante	1	-,707	.[b]
[Condition=Sham]	-,707	1	.[b]
[Condition=taVNS]	.[b]	.[b]	.[b]

a. Variable dépendante : Heat-sensitive C-fibers Thresholds ΔT0-T1.

b. La corrélation est manquante par défaut, car elle est associée à un paramètre redondant.

Matrice de covariance pour les estimations des effets fixes[a]

Paramètre	Constante	[Condition=Sham]	[Condition=taVNS]
Constante	,111190	-,111190	0[b]
[Condition=Sham]	-,111190	,222380	0[b]
[Condition=taVNS]	0[b]	0[b]	0[b]

a. Variable dépendante : Heat-sensitive C-fibers Thresholds ΔT0-T1.

b. La covariance est définie sur 0, car elle est associée à un paramètre redondant.

Paramètres de covariance

Estimations des paramètres de covariance[a]

Paramètre	Estimation	Erreur standard	Z de Wald	Sig.	Intervalle de confiance à 95 %	
					Borne inférieure	Borne supérieure
Résidu	2,446179	,533800	4,583	,000	1,594927	3,751765

a. Variable dépendante : Heat-sensitive C-fibers Thresholds ΔT0-T1.

Matrice de corrélation pour les estimations des paramètres de covariance[a]

Paramètre	Résidu
Résidu	1

a. Variable dépendante : Heat-sensitive C-fibers Thresholds ΔT0-T1.

Matrice de covariance pour les estimations des paramètres de covariance[a]

Paramètre	Résidu
Résidu	,284942

a. Variable dépendante : Heat-sensitive C-fibers Thresholds ΔT0-T1.

Moyenne marginale estimée

Condition

Estimations[a]

Condition	Moyenne	Erreur standard	ddl	Intervalle de confiance à 95 %

				Borne inférieure	Borne supérieure
Sham	,170	,333	42	-,502	,843
taVNS	,440	,333	42	-,233	1,113

a. Variable dépendante : Heat-sensitive C-fibers Thresholds ΔT0-T1.

Comparaisons appariées[a]

(I) Condition	(J) Condition	Différence moyenne (I-J)	Erreur standard	ddl	Sig.[b]
Sham	taVNS	-,270	,472	42	,570
taVNS	Sham	,270	,472	42	,570

Comparaisons appariées[a]

(I) Condition	(J) Condition	Intervalle de confiance à 95 % pour la différence[b] Borne inférieure	Borne supérieure
Sham	taVNS	-1,222	,682
taVNS	Sham	-,682	1,222

Basées sur les moyennes marginales estimées[a]

a. Variable dépendante : Heat-sensitive C-fibers Thresholds ΔT0-T1.

b. Ajustement pour les comparaisons multiples : Bonferroni.

Tests univariés[a]

Ddl du numérateur	Ddl du dénominateur	F	Sig.
1	42	,328	,570

Le test de F permet de tester l'effet de Condition. Il s'appuie sur les comparaisons appariées (indépendantes) linéaires parmi les moyennes marginales estimées.[a]

a. Variable dépendante : Heat-sensitive C-fibers Thresholds ΔT0-T1.

1.2. Heat-sensitive C-fibers Detection Thresholds (ΔT0-T2).

```
MIXED HeatsensitiveCfibersThresholdsΔT0T2 BY Condition
  /CRITERIA=CIN(95) MXITER(100) MXSTEP(10) SCORING(1)
SINGULAR(0.000000000001) HCONVERGE(0,
    ABSOLUTE) LCONVERGE(0, ABSOLUTE) PCONVERGE(0.000001, ABSOLUTE)
  /FIXED=Condition | SSTYPE(3)
  /METHOD=REML
  /PRINT=CPS CORB COVB DESCRIPTIVES G  SOLUTION TESTCOV
  /EMMEANS=TABLES(Condition) COMPARE ADJ(BONFERRONI).
```

Remarques

Sortie obtenue		04-MAY-2021 15:50:47
Commentaires		
Entrée	Jeu de données actif	Jeu_de_données1
	Filtre	<sans>
	Pondération	<sans>
	Fichier scindé	<sans>
	N de lignes dans le fichier de travail	44
Gestion des valeurs manquantes	Définition de la valeur manquante	Les valeurs manquantes définies par l'utilisateur sont traitées comme étant manquantes.
	Observations utilisées	Les statistiques sont basées sur toutes les observations comportant des données valides pour toutes les variables du modèle.
Syntaxe		MIXED HeatsensitiveCfibersThresholdsΔT0T2 BY Condition /CRITERIA=CIN(95) MXITER(100) MXSTEP(10) SCORING(1) SINGULAR(0.000000000001) HCONVERGE(0, ABSOLUTE) LCONVERGE(0, ABSOLUTE) PCONVERGE(0.000001, ABSOLUTE) /FIXED=Condition | SSTYPE(3) /METHOD=REML /PRINT=CPS CORB COVB DESCRIPTIVES G SOLUTION TESTCOV /EMMEANS=TABLES(Condition) COMPARE ADJ(BONFERRONI).
Ressources	Temps de processeur	00:00:00,02

Temps écoulé	00:00:00,01

Récapitulatif de traitement des observations

		Effectif	Pourcentage marginal
Condition	Sham	21	48,8%
	taVNS	22	51,2%
Valide		43	100,0%
Exclues		1	
Total		44	

Statistiques descriptives

Heat-sensitive C-fibers Thresholds ΔT0-T2

Condition	Effectif	Moyenne	Ecart type	Coefficient de variation
Sham	21	,41071	2,592701	631,3%
taVNS	22	,24432	1,859450	761,1%
Total	43	,32558	2,221905	682,4%

Dimension du modèle[a]

		Nombre de niveaux	Nombre de paramètres
Effets fixes	Constante	1	1
	Condition	2	1
Résidu			1
Total		3	3

a. Variable dépendante : Heat-sensitive C-fibers Thresholds ΔT0-T2.

Critères d'information[a]

Log de vraisemblance restreint -2	188,884
Critère d'information d'Akaike (AIC)	190,884
Critère de Hurvich et Tsai (AICC)	190,986
Critère de Bozdogan (CAIC)	193,597
Critère bayésien de Schwartz (BIC)	192,597

Les critères d'informations sont présentés en plus petit, disposant d'un meilleur format.[a]

a. Variable dépendante : Heat-sensitive C-fibers Thresholds ΔT0-T2.

Effets fixes

Tests des effets fixes de type III[a]

Source	Ddl du numérateur	Ddl du dénominateur	F	Sig.
Constante	1	41	,913	,345
Condition	1	41	,059	,809

a. Variable dépendante : Heat-sensitive C-fibers Thresholds ΔT0-T2.

Estimations des effets fixes[a]

Paramètre	Estimation	Erreur standard	ddl	t	Sig.	Intervalle de confiance à 95 % Borne inférieure
Constante	,244318	,479110	41	,510	,613	-,723264
[Condition=Sham]	,166396	,685582	41	,243	,809	-1,218165
[Condition=taVNS]	0[b]	0	.	.	.	.

Estimations des effets fixes[a]

Paramètre	Intervalle de confiance à 95 % Borne supérieure
Constante	1,211900
[Condition=Sham]	1,550958
[Condition=taVNS]	.

a. Variable dépendante : Heat-sensitive C-fibers Thresholds ΔT0-T2.

b. Ce paramètre est défini sur 0, car il est redondant.

Matrice de corrélation pour les estimations des effets fixes[a]

Paramètre	Constante	[Condition=Sham]	[Condition=taVNS]
Constante	1	-,699	.[b]

[Condition=Sham]	-,699	1	.[b]
[Condition=taVNS]	.[b]	.[b]	.[b]

a. Variable dépendante : Heat-sensitive C-fibers Thresholds ΔT0-T2.
b. La corrélation est manquante par défaut, car elle est associée à un paramètre redondant.

Matrice de covariance pour les estimations des effets fixes[a]

Paramètre	Constante	[Condition=Sham]	[Condition=taVNS]
Constante	,229546	-,229546	0[b]
[Condition=Sham]	-,229546	,470023	0[b]
[Condition=taVNS]	0[b]	0[b]	0[b]

a. Variable dépendante : Heat-sensitive C-fibers Thresholds ΔT0-T2.
b. La covariance est définie sur 0, car elle est associée à un paramètre redondant.

Paramètres de covariance

Estimations des paramètres de covariance[a]

Paramètre	Estimation	Erreur standard	Z de Wald	Sig.	Intervalle de confiance à 95 %	
					Borne inférieure	Borne supérieure
Résidu	5,050015	1,115362	4,528	,000	3,275622	7,785592

a. Variable dépendante : Heat-sensitive C-fibers Thresholds ΔT0-T2.

Matrice de corrélation pour les estimations des paramètres de covariance[a]

Paramètre	Résidu
Résidu	1

a. Variable dépendante : Heat-sensitive C-fibers Thresholds ΔT0-T2.

Matrice de covariance pour les estimations des paramètres de covariance[a]

Paramètre	Résidu
Résidu	1,244032

a. Variable dépendante : Heat-sensitive C-fibers Thresholds ΔT0-T2.

Moyenne marginale estimée

Condition

Estimations[a]

Condition	Moyenne	Erreur standard	ddl	Intervalle de confiance à 95 %	
				Borne inférieure	Borne supérieure
Sham	,411	,490	41	-,580	1,401
taVNS	,244	,479	41	-,723	1,212

a. Variable dépendante : Heat-sensitive C-fibers Thresholds ΔT0-T2.

Comparaisons appariées[a]

(I) Condition	(J) Condition	Différence moyenne (I-J)	Erreur standard	ddl	Sig.[b]
Sham	taVNS	,166	,686	41	,809
taVNS	Sham	-,166	,686	41	,809

Comparaisons appariées[a]

(I) Condition	(J) Condition	Intervalle de confiance à 95 % pour la différence[b]	
		Borne inférieure	Borne supérieure
Sham	taVNS	-1,218	1,551
taVNS	Sham	-1,551	1,218

Basées sur les moyennes marginales estimées[a]

a. Variable dépendante : Heat-sensitive C-fibers Thresholds ΔT0-T2.

b. Ajustement pour les comparaisons multiples : Bonferroni.

Tests univariés[a]

Ddl du numérateur	Ddl du dénominateur	F	Sig.
1	41	,059	,809

Le test de F permet de tester l'effet de Condition. Il s'appuie sur les comparaisons appariées (indépendantes) linéaires parmi les moyennes marginales estimées.[a]

a. Variable dépendante : Heat-sensitive C-fibers Thresholds ΔT0-T2.

1.3. Heat-sensitive Aδ-fibers Thresholds (ΔT0-T1).

```
MIXED HeatsensitiveAδfibersThresholdsΔT0T1 BY Condition
  /CRITERIA=CIN(95) MXITER(100) MXSTEP(10) SCORING(1)
SINGULAR(0.000000000001) HCONVERGE(0,
    ABSOLUTE) LCONVERGE(0, ABSOLUTE) PCONVERGE(0.000001, ABSOLUTE)
  /FIXED=Condition | SSTYPE(3)
  /METHOD=REML
  /PRINT=CPS CORB COVB DESCRIPTIVES G  SOLUTION TESTCOV
  /EMMEANS=TABLES(Condition) COMPARE ADJ(BONFERRONI).
```

Remarques

Sortie obtenue		04-MAY-2021 15:47:40
Commentaires		
Entrée	Jeu de données actif	Jeu_de_données1
	Filtre	<sans>
	Pondération	<sans>
	Fichier scindé	<sans>
	N de lignes dans le fichier de travail	44
Gestion des valeurs manquantes	Définition de la valeur manquante	Les valeurs manquantes définies par l'utilisateur sont traitées comme étant manquantes.

	Observations utilisées	Les statistiques sont basées sur toutes les observations comportant des données valides pour toutes les variables du modèle.
Syntaxe		MIXED HeatsensitiveAδfibersThresholdsΔT0T1 BY Condition<br>/CRITERIA=CIN(95) MXITER(100) MXSTEP(10) SCORING(1) SINGULAR(0.000000000001) HCONVERGE(0, ABSOLUTE) LCONVERGE(0, ABSOLUTE) PCONVERGE(0.000001, ABSOLUTE)<br>/FIXED=Condition | SSTYPE(3)<br>/METHOD=REML<br>/PRINT=CPS CORB COVB DESCRIPTIVES G SOLUTION TESTCOV<br>/EMMEANS=TABLES(Condition) COMPARE ADJ(BONFERRONI).
Ressources	Temps de processeur	00:00:00,02
	Temps écoulé	00:00:00,01

Récapitulatif de traitement des observations

		Effectif	Pourcentage marginal
Condition	Sham	22	50,0%
	taVNS	22	50,0%
Valide		44	100,0%
Exclues		0	
Total		44	

Statistiques descriptives

Heat-sensitive Aδ-fibers Thresholds ΔT0-T1

Condition	Effectif	Moyenne	Ecart type	Coefficient de variation
Sham	22	-,095340909090909	1,724322209051343	-1808,6%
taVNS	22	,279356090909091	1,694332986386985	606,5%
Total	44	,092007590909091	1,699996269469372	1847,7%

Dimension du modèle[a]

		Nombre de niveaux	Nombre de paramètres
Effets fixes	Constante	1	1
	Condition	2	1
Résidu			1
Total		3	3

a. Variable dépendante : Heat-sensitive Aδ-fibers Thresholds ΔT0-T1.

Critères d'information[a]

Log de vraisemblance restreint -2	170,409
Critère d'information d'Akaike (AIC)	172,409
Critère de Hurvich et Tsai (AICC)	172,509
Critère de Bozdogan (CAIC)	175,146
Critère bayésien de Schwartz (BIC)	174,146

Les critères d'informations sont présentés en plus petit, disposant d'un meilleur format.[a]

a. Variable dépendante : Heat-sensitive Aδ-fibers Thresholds ΔT0-T1.

Effets fixes

Tests des effets fixes de type III[a]

Source	Ddl du numérateur	Ddl du dénominateur	F	Sig.
Constante	1	42	,127	,723
Condition	1	42	,529	,471

a. Variable dépendante : Heat-sensitive Aδ-fibers Thresholds ΔT0-T1.

Estimations des effets fixes[a]

Paramètre	Estimation	Erreur standard	ddl	t	Sig.	Intervalle de confiance à 95 % Borne inférieure
Constante	,279356	,364444	42	,767	,448	-,456121
[Condition=Sham]	-,374697	,515401	42	-,727	,471	-1,414819
[Condition=taVNS]	0[b]	0	.	.	.	.

Estimations des effets fixes[a]

Paramètre	Intervalle de confiance à 95 % Borne supérieure
Constante	1,014834
[Condition=Sham]	,665425
[Condition=taVNS]	.

a. Variable dépendante : Heat-sensitive Aδ-fibers Thresholds ΔT0-T1.

b. Ce paramètre est défini sur 0, car il est redondant.

Matrice de corrélation pour les estimations des effets fixes[a]

Paramètre	Constante	[Condition=Sham]	[Condition=taVNS]
Constante	1	-,707	.[b]
[Condition=Sham]	-,707	1	.[b]
[Condition=taVNS]	.[b]	.[b]	.[b]

a. Variable dépendante : Heat-sensitive Aδ-fibers Thresholds ΔT0-T1.

b. La corrélation est manquante par défaut, car elle est associée à un paramètre redondant.

Matrice de covariance pour les estimations des effets fixes[a]

Paramètre	Constante	[Condition=Sham]	[Condition=taVNS]
Constante	,132819	-,132819	0[b]

[Condition=Sham]	-,132819	,265639	0[b]
[Condition=taVNS]	0[b]	0[b]	0[b]

a. Variable dépendante : Heat-sensitive Aδ-fibers Thresholds ΔT0-T1.

b. La covariance est définie sur 0, car elle est associée à un paramètre redondant.

Paramètres de covariance

Estimations des paramètres de covariance[a]

Paramètre	Estimation	Erreur standard	Z de Wald	Sig.	Intervalle de confiance à 95 %	
					Borne inférieure	Borne supérieure
Résidu	2,922026	,637638	4,583	,000	1,905183	4,481583

a. Variable dépendante : Heat-sensitive Aδ-fibers Thresholds ΔT0-T1.

Matrice de corrélation pour les estimations des paramètres de covariance[a]

Paramètre	Résidu
Résidu	1

a. Variable dépendante : Heat-sensitive Aδ-fibers Thresholds ΔT0-T1.

Matrice de covariance pour les estimations des paramètres de covariance[a]

Paramètre	Résidu
Résidu	,406583

a. Variable dépendante : Heat-sensitive Aδ-fibers Thresholds ΔT0-T1.

Moyenne marginale estimée

Condition

Estimations[a]

Condition	Moyenne	Erreur standard	ddl	Intervalle de confiance à 95 % Borne inférieure	Borne supérieure
Sham	-,095	,364	42	-,831	,640
taVNS	,279	,364	42	-,456	1,015

a. Variable dépendante : Heat-sensitive Aδ-fibers Thresholds ΔT0-T1.

Comparaisons appariées[a]

(I) Condition	(J) Condition	Différence moyenne (I-J)	Erreur standard	ddl	Sig.[b]
Sham	taVNS	-,375	,515	42	,471
taVNS	Sham	,375	,515	42	,471

Comparaisons appariées[a]

(I) Condition	(J) Condition	Intervalle de confiance à 95 % pour la différence[b] Borne inférieure	Borne supérieure
Sham	taVNS	-1,415	,665
taVNS	Sham	-,665	1,415

Basées sur les moyennes marginales estimées[a]

a. Variable dépendante : Heat-sensitive Aδ-fibers Thresholds ΔT0-T1.

b. Ajustement pour les comparaisons multiples : Bonferroni.

Tests univariés[a]

Ddl du numérateur	Ddl du dénominateur	F	Sig.
1	42	,529	,471

Le test de F permet de tester l'effet de Condition. Il s'appuie sur les comparaisons appariées (indépendantes) linéaires parmi les moyennes marginales estimées.[a]

a. Variable dépendante : Heat-sensitive Aδ-fibers Thresholds ΔT0-T1.

1.4. Heat-sensitive Aδ-fibers Thresholds (ΔT0-T2).

```
MIXED HeatsensitiveAδfibersThresholdsΔT0T2 BY Condition
  /CRITERIA=CIN(95) MXITER(100) MXSTEP(10) SCORING(1)
SINGULAR(0.000000000001) HCONVERGE(0,
    ABSOLUTE) LCONVERGE(0, ABSOLUTE) PCONVERGE(0.000001, ABSOLUTE)
  /FIXED=Condition | SSTYPE(3)
  /METHOD=REML
  /PRINT=CPS CORB COVB DESCRIPTIVES G  SOLUTION TESTCOV
  /EMMEANS=TABLES(Condition) COMPARE ADJ(BONFERRONI).
```

Remarques

Sortie obtenue		05-MAY-2021 08:56:57
Commentaires		
Entrée	Jeu de données actif	Jeu_de_données1
	Filtre	<sans>
	Pondération	<sans>
	Fichier scindé	<sans>
	N de lignes dans le fichier de travail	44
Gestion des valeurs manquantes	Définition de la valeur manquante	Les valeurs manquantes définies par l'utilisateur sont traitées comme étant manquantes.
	Observations utilisées	Les statistiques sont basées sur toutes les observations comportant des données valides pour toutes les variables du modèle.

Syntaxe		MIXED HeatsensitiveAδfibersThresholdsΔT0T2 BY Condition /CRITERIA=CIN(95) MXITER(100) MXSTEP(10) SCORING(1) SINGULAR(0.000000000001) HCONVERGE(0, ABSOLUTE) LCONVERGE(0, ABSOLUTE) PCONVERGE(0.000001, ABSOLUTE) /FIXED=Condition | SSTYPE(3) /METHOD=REML /PRINT=CPS CORB COVB DESCRIPTIVES G SOLUTION TESTCOV /EMMEANS=TABLES(Condition) COMPARE ADJ(BONFERRONI).
Ressources	Temps de processeur	00:00:00,00
	Temps écoulé	00:00:00,01

Récapitulatif de traitement des observations

		Effectif	Pourcentage marginal
Condition	Sham	21	48,8%
	taVNS	22	51,2%
Valide		43	100,0%
Exclues		1	
Total		44	

Statistiques descriptives

Heat-sensitive Aδ-fibers Thresholds ΔT0-T2

Condition	Effectif	Moyenne	Ecart type	Coefficient de variation
Sham	21	-,063690476190476	2,331580689554783	-3660,8%

taVNS	22	,681363636363636	1,854603141875052	272,2%
Total	43	,317500000000000	2,109615951846618	664,4%

Dimension du modèle[a]

		Nombre de niveaux	Nombre de paramètres
Effets fixes	Constante	1	1
	Condition	2	1
Résidu			1
Total		3	3

a. Variable dépendante : Heat-sensitive Aδ-fibers Thresholds ΔT0-T2.

Critères d'information[a]

Log de vraisemblance restreint -2	183,360
Critère d'information d'Akaike (AIC)	185,360
Critère de Hurvich et Tsai (AICC)	185,463
Critère de Bozdogan (CAIC)	188,074
Critère bayésien de Schwartz (BIC)	187,074

Les critères d'informations sont présentés en plus petit, disposant d'un meilleur format.[a]

a. Variable dépendante : Heat-sensitive Aδ-fibers Thresholds ΔT0-T2.

Effets fixes

Tests des effets fixes de type III[a]

Source	Ddl du numérateur	Ddl du dénominateur	F	Sig.
Constante	1	41	,929	,341
Condition	1	41	1,351	,252

a. Variable dépendante : Heat-sensitive Aδ-fibers Thresholds ΔT0-T2.

Estimations des effets fixes[a]

Paramètre	Estimation	Erreur standard	ddl	t	Sig.	Intervalle de confiance à 95 % Borne inférieure
Constante	,681364	,447902	41	1,521	,136	-,223193
[Condition=Sham]	-,745054	,640926	41	-1,162	,252	-2,039430
[Condition=taVNS]	0[b]	0	.	.	.	.

Estimations des effets fixes[a]

Paramètre	Intervalle de confiance à 95 % Borne supérieure
Constante	1,585920
[Condition=Sham]	,549322
[Condition=taVNS]	.

a. Variable dépendante : Heat-sensitive Aδ-fibers Thresholds ΔT0-T2.

b. Ce paramètre est défini sur 0, car il est redondant.

Matrice de corrélation pour les estimations des effets fixes[a]

Paramètre	Constante	[Condition=Sham]	[Condition=taVNS]
Constante	1	-,699	.[b]
[Condition=Sham]	-,699	1	.[b]
[Condition=taVNS]	.[b]	.[b]	.[b]

a. Variable dépendante : Heat-sensitive Aδ-fibers Thresholds ΔT0-T2.

b. La corrélation est manquante par défaut, car elle est associée à un paramètre redondant.

Matrice de covariance pour les estimations des effets fixes[a]

Paramètre	Constante	[Condition=Sham]	[Condition=taVNS]
Constante	,200616	-,200616	0[b]
[Condition=Sham]	-,200616	,410786	0[b]
[Condition=taVNS]	0[b]	0[b]	0[b]

a. Variable dépendante : Heat-sensitive Aδ-fibers Thresholds ΔT0-T2.

b. La covariance est définie sur 0, car elle est associée à un paramètre redondant.

Paramètres de covariance

Estimations des paramètres de covariance[a]

Paramètre	Estimation	Erreur standard	Z de Wald	Sig.	Intervalle de confiance à 95 % Borne inférieure	Borne supérieure
Résidu	4,413560	,974792	4,528	,000	2,862794	6,804372

a. Variable dépendante : Heat-sensitive Aδ-fibers Thresholds ΔT0-T2.

Matrice de corrélation pour les estimations des paramètres de covariance[a]

Paramètre	Résidu
Résidu	1

a. Variable dépendante : Heat-sensitive Aδ-fibers Thresholds ΔT0-T2.

Matrice de covariance pour les estimations des paramètres de covariance[a]

Paramètre	Résidu
Résidu	,950220

a. Variable dépendante : Heat-sensitive Aδ-fibers Thresholds ΔT0-T2.

Moyenne marginale estimée

Condition

Estimations[a]

Condition	Moyenne	Erreur standard	ddl	Intervalle de confiance à 95 % Borne inférieure	Intervalle de confiance à 95 % Borne supérieure
Sham	-,064	,458	41	-,990	,862
taVNS	,681	,448	41	-,223	1,586

a. Variable dépendante : Heat-sensitive Aδ-fibers Thresholds ΔT0-T2.

Comparaisons appariées[a]

(I) Condition	(J) Condition	Différence moyenne (I-J)	Erreur standard	ddl	Sig.[b]
Sham	taVNS	-,745	,641	41	,252
taVNS	Sham	,745	,641	41	,252

Comparaisons appariées[a]

(I) Condition	(J) Condition	Intervalle de confiance à 95 % pour la différence[b] Borne inférieure	Borne supérieure
Sham	taVNS	-2,039	,549
taVNS	Sham	-,549	2,039

Basées sur les moyennes marginales estimées[a]

a. Variable dépendante : Heat-sensitive Aδ-fibers Thresholds ΔT0-T2.

b. Ajustement pour les comparaisons multiples : Bonferroni.

Tests univariés[a]

Ddl du numérateur	Ddl du dénominateur	F	Sig.
1	41	1,351	,252

Le test de F permet de tester l'effet de Condition. Il s'appuie sur les comparaisons appariées (indépendantes) linéaires parmi les moyennes marginales estimées.[a]

a. Variable dépendante : Heat-sensitive Aδ-fibers Thresholds ΔT0-T2.

1.5. Mechanosensitive Aβ-fibers Thresholds (ΔT0-T1).

```
MIXED MechanosensitiveAβfibersThresholdsΔT0T1 BY Condition
  /CRITERIA=CIN(95) MXITER(100) MXSTEP(10) SCORING(1)
SINGULAR(0.000000000001) HCONVERGE(0,
    ABSOLUTE) LCONVERGE(0, ABSOLUTE) PCONVERGE(0.000001, ABSOLUTE)
  /FIXED=Condition | SSTYPE(3)
  /METHOD=REML
  /PRINT=CPS CORB COVB DESCRIPTIVES G  SOLUTION TESTCOV
  /EMMEANS=TABLES(Condition) COMPARE ADJ(BONFERRONI).
```

Remarques

Sortie obtenue		04-MAY-2021 15:49:02
Commentaires		
Entrée	Jeu de données actif	Jeu_de_données1
	Filtre	<sans>
	Pondération	<sans>
	Fichier scindé	<sans>
	N de lignes dans le fichier de travail	44
Gestion des valeurs manquantes	Définition de la valeur manquante	Les valeurs manquantes définies par l'utilisateur sont traitées comme étant manquantes.
	Observations utilisées	Les statistiques sont basées sur toutes les observations comportant des données valides pour toutes les variables du modèle.

Syntaxe		MIXED MechanosensitiveAβfibersThresholdsΔT0T1 BY Condition<br>/CRITERIA=CIN(95) MXITER(100) MXSTEP(10) SCORING(1) SINGULAR(0.000000000001) HCONVERGE(0, ABSOLUTE) LCONVERGE(0, ABSOLUTE) PCONVERGE(0.000001, ABSOLUTE)<br>/FIXED=Condition | SSTYPE(3)<br>/METHOD=REML<br>/PRINT=CPS CORB COVB DESCRIPTIVES G SOLUTION TESTCOV<br>/EMMEANS=TABLES(Condition) COMPARE ADJ(BONFERRONI).
Ressources	Temps de processeur	00:00:00,00
	Temps écoulé	00:00:00,01

Récapitulatif de traitement des observations

		Effectif	Pourcentage marginal
Condition	Sham	22	50,0%
	taVNS	22	50,0%
Valide		44	100,0%
Exclues		0	
Total		44	

Statistiques descriptives

Mechanosensitive Aβ-fibers Thresholds ΔT0-T1

Condition	Effectif	Moyenne	Ecart type	Coefficient de variation
Sham	22	-,001868153636364	,004182778657128	-223,9%

taVNS	22	-,002549772727273	,003966223007584	-155,6%
Total	44	-,002208963181818	,004042991229404	-183,0%

Dimension du modèle[a]

		Nombre de niveaux	Nombre de paramètres
Effets fixes	Constante	1	1
	Condition	2	1
Résidu			1
Total		3	3

a. Variable dépendante : Mechanosensitive Aβ-fibers Thresholds ΔT0-T1.

Critères d'information[a]

Log de vraisemblance restreint -2	-336,850
Critère d'information d'Akaike (AIC)	-334,850
Critère de Hurvich et Tsai (AICC)	-334,750
Critère de Bozdogan (CAIC)	-332,112
Critère bayésien de Schwartz (BIC)	-333,112

Les critères d'informations sont présentés en plus petit, disposant d'un meilleur format.[a]

a. Variable dépendante : Mechanosensitive Aβ-fibers Thresholds ΔT0-T1.

Effets fixes

Tests des effets fixes de type III[a]

Source	Ddl du numérateur	Ddl du dénominateur	F	Sig.
Constante	1	42	12,923	,001
Condition	1	42	,308	,582

a. Variable dépendante : Mechanosensitive Aβ-fibers Thresholds ΔT0-T1.

Estimations des effets fixes[a]

Paramètre	Estimation	Erreur standard	ddl	t	Sig.	Intervalle de confiance à 95 % Borne inférieure
Constante	-,002550	,000869	42	-2,934	,005	-,004303
[Condition=Sham]	,000682	,001229	42	,555	,582	-,001798
[Condition=taVNS]	0[b]	0	.	.	.	.

Estimations des effets fixes[a]

Paramètre	Intervalle de confiance à 95 % Borne supérieure
Constante	-,000796
[Condition=Sham]	,003162
[Condition=taVNS]	.

a. Variable dépendante : Mechanosensitive Aβ-fibers Thresholds ΔT0-T1.

b. Ce paramètre est défini sur 0, car il est redondant.

Matrice de corrélation pour les estimations des effets fixes[a]

Paramètre	Constante	[Condition=Sham]	[Condition=taVNS]
Constante	1	-,707	.[b]
[Condition=Sham]	-,707	1	.[b]
[Condition=taVNS]	.[b]	.[b]	.[b]

a. Variable dépendante : Mechanosensitive Aβ-fibers Thresholds ΔT0-T1.

b. La corrélation est manquante par défaut, car elle est associée à un paramètre redondant.

Paramètres de covariance

Matrice de covariance pour les estimations des effets fixes[a]

Paramètre	Constante	[Condition=Sham]	[Condition=taVNS]
Constante	7,551491E-7	-7,551491E-7	0[b]
[Condition=Sham]	-7,551491E-7	1,510298E-6	0[b]
[Condition=taVNS]	0[b]	0[b]	0[b]

a. Variable dépendante : Mechanosensitive Aβ-fibers Thresholds ΔT0-T1.

b. La covariance est définie sur 0, car elle est associée à un paramètre redondant.

Estimations des paramètres de covariance[a]

Paramètre	Estimation	Erreur standard	Z de Wald	Sig.	Intervalle de confiance à 95 %	
					Borne inférieure	Borne supérieure
Résidu	1,661328E-5	3,625315E-6	4,583	,000	1,083198E-5	2,548020E-5

a. Variable dépendante : Mechanosensitive Aβ-fibers Thresholds ΔT0-T1.

Matrice de corrélation pour les estimations des paramètres de covariance[a]

Paramètre	Résidu
Résidu	1

a. Variable dépendante : Mechanosensitive Aβ-fibers Thresholds ΔT0-T1.

Matrice de covariance pour les estimations des paramètres de covariance[a]

Paramètre	Résidu
Résidu	1,314291E-11

a. Variable dépendante : Mechanosensitive Aβ-fibers Thresholds ΔT0-T1.

Moyenne marginale estimée

Condition

Estimations[a]

Condition	Moyenne	Erreur standard	ddl	Intervalle de confiance à 95 %	
				Borne inférieure	Borne supérieure
Sham	-,002	,001	42	-,004	,000

taVNS	-,003	,001	42	-,004	-,001

a. Variable dépendante : Mechanosensitive Aβ-fibers Thresholds ΔT0-T1.

Comparaisons appariées[a]

(I) Condition	(J) Condition	Différence moyenne (I-J)	Erreur standard	ddl	Sig.[b]
Sham	taVNS	,001	,001	42	,582
taVNS	Sham	-,001	,001	42	,582

Comparaisons appariées[a]

(I) Condition	(J) Condition	Intervalle de confiance à 95 % pour la différence[b] Borne inférieure	Borne supérieure
Sham	taVNS	-,002	,003
taVNS	Sham	-,003	,002

Basées sur les moyennes marginales estimées[a]

a. Variable dépendante : Mechanosensitive Aβ-fibers Thresholds ΔT0-T1.

b. Ajustement pour les comparaisons multiples : Bonferroni.

Tests univariés[a]

Ddl du numérateur	Ddl du dénominateur	F	Sig.
1	42	,308	,582

Le test de F permet de tester l'effet de Condition. Il s'appuie sur les comparaisons appariées (indépendantes) linéaires parmi les moyennes marginales estimées.[a]

a. Variable dépendante : Mechanosensitive Aβ-fibers Thresholds ΔT0-T1.

1.6. Mechanosensitive Aβ-fibers Thresholds (ΔT0-T2).

```
MIXED MechanosensitiveAβfibersThresholdsΔT0T2 BY Condition
  /CRITERIA=CIN(95) MXITER(100) MXSTEP(10) SCORING(1)
SINGULAR(0.000000000001) HCONVERGE(0,
    ABSOLUTE) LCONVERGE(0, ABSOLUTE) PCONVERGE(0.000001, ABSOLUTE)
  /FIXED=Condition | SSTYPE(3)
  /METHOD=REML
  /PRINT=CPS CORB COVB DESCRIPTIVES  SOLUTION TESTCOV
  /EMMEANS=TABLES(OVERALL)
  /EMMEANS=TABLES(Condition) COMPARE ADJ(BONFERRONI).
```

Remarques

Sortie obtenue		11-MAY-2021 13:08:45
Commentaires		
Entrée	Jeu de données actif	Jeu_de_données1

	Filtre	<sans>
	Pondération	<sans>
	Fichier scindé	<sans>
	N de lignes dans le fichier de travail	44
Gestion des valeurs manquantes	Définition de la valeur manquante	Les valeurs manquantes définies par l'utilisateur sont traitées comme étant manquantes.
	Observations utilisées	Les statistiques sont basées sur toutes les observations comportant des données valides pour toutes les variables du modèle.
Syntaxe		MIXED MechanosensitiveAβfibersThresholdsΔT0T2 BY Condition<br>/CRITERIA=CIN(95) MXITER(100) MXSTEP(10) SCORING(1) SINGULAR(0.000000000001) HCONVERGE(0, ABSOLUTE) LCONVERGE(0, ABSOLUTE) PCONVERGE(0.000001, ABSOLUTE)<br>/FIXED=Condition | SSTYPE(3)<br>/METHOD=REML<br>/PRINT=CPS CORB COVB DESCRIPTIVES SOLUTION TESTCOV<br>/EMMEANS=TABLES(OVERALL)<br>/EMMEANS=TABLES(Condition) COMPARE ADJ(BONFERRONI).
Ressources	Temps de processeur	00:00:00,02
	Temps écoulé	00:00:00,01

Récapitulatif de traitement des observations

		Effectif	Pourcentage marginal
Condition	Sham	21	48,8%
	taVNS	22	51,2%
Valide		43	100,0%
Exclues		1	
Total		44	

Statistiques descriptives

Mechanosensitive Aβ-fibers Thresholds ΔT0-T2

Condition	Effectif	Moyenne	Ecart type	Coefficient de variation
Sham	21	-,001915952380952	,004078416549057	-212,9%
taVNS	22	-,003668863636364	,006275591023546	-171,0%
Total	43	-,002812790697674	,005329002330277	-189,5%

Dimension du modèle[a]

		Nombre de niveaux	Nombre de paramètres
Effets fixes	Constante	1	1
	Condition	2	1
Résidu			1
Total		3	3

a. Variable dépendante : Mechanosensitive Aβ-fibers Thresholds ΔT0-T2.

Critères d'information[a]

Log de vraisemblance restreint -2	-306,911
Critère d'information d'Akaike (AIC)	-304,911

Critère de Hurvich et Tsai (AICC)	-304,808
Critère de Bozdogan (CAIC)	-302,197
Critère bayésien de Schwartz (BIC)	-303,197

Les critères d'informations sont présentés en plus petit, disposant d'un meilleur format.[a]

a. Variable dépendante : Mechanosensitive Aβ-fibers Thresholds ΔT0-T2.

Effets fixes

Tests des effets fixes de type III[a]

Source	Ddl du numérateur	Ddl du dénominateur	F	Sig.
Constante	1	41	11,847	,001
Condition	1	41	1,167	,286

a. Variable dépendante : Mechanosensitive Aβ-fibers Thresholds ΔT0-T2.

Estimations des effets fixes[a]

Paramètre	Estimation	Erreur standard	ddl	t	Sig.	Intervalle de confiance à 95 %
						Borne inférieure
Constante	-,003669	,001134	41	-3,236	,002	-,005959
[Condition=Sham]	,001753	,001623	41	1,080	,286	-,001524
[Condition=taVNS]	0[b]	0	.	.	.	.

Estimations des effets fixes[a]

Paramètre	Intervalle de confiance à 95 %
	Borne supérieure
Constante	-,001379
[Condition=Sham]	,005030
[Condition=taVNS]	.

a. Variable dépendante : Mechanosensitive Aβ-fibers Thresholds ΔT0-T2.

b. Ce paramètre est défini sur 0, car il est redondant.

Matrice de corrélation pour les estimations des effets fixes[a]

Paramètre	Constante	[Condition=Sham]	[Condition=taVNS]
Constante	1	-,699	.[b]
[Condition=Sham]	-,699	1	.[b]
[Condition=taVNS]	.[b]	.[b]	.[b]

a. Variable dépendante : Mechanosensitive Aβ-fibers Thresholds ΔT0-T2.

b. La corrélation est manquante par défaut, car elle est associée à un paramètre redondant.

Matrice de covariance pour les estimations des effets fixes[a]

Paramètre	Constante	[Condition=Sham]	[Condition=taVNS]
Constante	1,285713E-6	-1,285713E-6	0[b]
[Condition=Sham]	-1,285713E-6	2,632651E-6	0[b]
[Condition=taVNS]	0[b]	0[b]	0[b]

a. Variable dépendante : Mechanosensitive Aβ-fibers Thresholds ΔT0-T2.

b. La covariance est définie sur 0, car elle est associée à un paramètre redondant.

Paramètres de covariance

Estimations des paramètres de covariance[a]

Paramètre	Estimation	Erreur standard	Z de Wald	Sig.	Intervalle de confiance à 95 % Borne inférieure	Borne supérieure
Résidu	2,828570E-5	6,247265E-6	4,528	,000	1,834712E-5	4,360797E-5

a. Variable dépendante : Mechanosensitive Aβ-fibers Thresholds ΔT0-T2.

Matrice de corrélation pour les estimations des paramètres de covariance[a]

Paramètre	Résidu
Résidu	1

a. Variable dépendante : Mechanosensitive Aβ-fibers Thresholds ΔT0-T2.

Matrice de covariance pour les estimations des paramètres de covariance[a]

Paramètre	Résidu
Résidu	3,902832E-11

a. Variable dépendante : Mechanosensitive Aβ-fibers Thresholds ΔT0-T2.

Moyenne marginale estimée

1. Grand Mean[a]

Moyenne	Erreur standard	ddl	Intervalle de confiance à 95 % Borne inférieure	Intervalle de confiance à 95 % Borne supérieure
-,003	,001	41	-,004	-,001

a. Variable dépendante : Mechanosensitive Aβ-fibers Thresholds ΔT0-T2.

2. Condition

Estimations[a]

Condition	Moyenne	Erreur standard	ddl	Intervalle de confiance à 95 % Borne inférieure	Intervalle de confiance à 95 % Borne supérieure
Sham	-,002	,001	41	-,004	,000
taVNS	-,004	,001	41	-,006	-,001

a. Variable dépendante : Mechanosensitive Aβ-fibers Thresholds ΔT0-T2.

Comparaisons appariées[a]

(I) Condition	(J) Condition	Différence moyenne (I-J)	Erreur standard	ddl	Sig.[b]
Sham	taVNS	,002	,002	41	,286
taVNS	Sham	-,002	,002	41	,286

Comparaisons appariées[a]

(I) Condition	(J) Condition	Intervalle de confiance à 95 % pour la différence[b]

		Borne inférieure	Borne supérieure
Sham	taVNS	-,002	,005
taVNS	Sham	-,005	,002

Basées sur les moyennes marginales estimées[a]

a. Variable dépendante : Mechanosensitive Aβ-fibers Thresholds ΔT0-T2.

b. Ajustement pour les comparaisons multiples : Bonferroni.

Tests univariés[a]

Ddl du numérateur	Ddl du dénominateur	F	Sig.
1	41	1,167	,286

Le test de F permet de tester l'effet de Condition. Il s'appuie sur les comparaisons appariées (indépendantes) linéaires parmi les moyennes marginales estimées.[a]

a. Variable dépendante : Mechanosensitive Aβ-fibers Thresholds ΔT0-T2.

1.7. Cool-sensitive Aδ-fibers Thresholds (ΔT0-T1).

```
MIXED CoolsensitiveAδfibersThresholdsΔT0T1 BY Condition
  /CRITERIA=CIN(95) MXITER(100) MXSTEP(10) SCORING(1)
SINGULAR(0.000000000001) HCONVERGE(0,
    ABSOLUTE) LCONVERGE(0, ABSOLUTE) PCONVERGE(0.000001, ABSOLUTE)
  /FIXED=Condition | SSTYPE(3)
  /METHOD=REML
  /PRINT=CPS CORB COVB DESCRIPTIVES G  SOLUTION TESTCOV
  /EMMEANS=TABLES(Condition) COMPARE ADJ(BONFERRONI).
```

Remarques

Sortie obtenue		04-MAY-2021 15:49:58
Commentaires		
Entrée	Jeu de données actif	Jeu_de_données1
	Filtre	<sans>
	Pondération	<sans>
	Fichier scindé	<sans>
	N de lignes dans le fichier de travail	44
Gestion des valeurs manquantes	Définition de la valeur manquante	Les valeurs manquantes définies par l'utilisateur sont traitées comme étant manquantes.

	Observations utilisées	Les statistiques sont basées sur toutes les observations comportant des données valides pour toutes les variables du modèle.
Syntaxe		MIXED CoolsensitiveAδfibersThresholdsΔT0T1 BY Condition /CRITERIA=CIN(95) MXITER(100) MXSTEP(10) SCORING(1) SINGULAR(0.000000000001) HCONVERGE(0, ABSOLUTE) LCONVERGE(0, ABSOLUTE) PCONVERGE(0.000001, ABSOLUTE) /FIXED=Condition | SSTYPE(3) /METHOD=REML /PRINT=CPS CORB COVB DESCRIPTIVES G SOLUTION TESTCOV /EMMEANS=TABLES(Condition) COMPARE ADJ(BONFERRONI).
Ressources	Temps de processeur	00:00:00,03
	Temps écoulé	00:00:00,01

Récapitulatif de traitement des observations

		Effectif	Pourcentage marginal
Condition	Sham	22	50,0%
	taVNS	22	50,0%
Valide		44	100,0%
Exclues		0	
Total		44	

Statistiques descriptives

Cool-sensitive Aδ-fibers Thresholds ΔT0-T1

Condition	Effectif	Moyenne	Ecart type	Coefficient de variation
Sham	22	,802897727272727	1,183849739749143	147,4%
taVNS	22	,827840909090909	1,290164094273095	155,8%
Total	44	,815369318181818	1,223731800216990	150,1%

Dimension du modèle[a]

		Nombre de niveaux	Nombre de paramètres
Effets fixes	Constante	1	1
	Condition	2	1
Résidu			1
Total		3	3

a. Variable dépendante : Cool-sensitive Aδ-fibers Thresholds ΔT0-T1.

Critères d'information[a]

Log de vraisemblance restreint -2	143,317
Critère d'information d'Akaike (AIC)	145,317
Critère de Hurvich et Tsai (AICC)	145,417
Critère de Bozdogan (CAIC)	148,054
Critère bayésien de Schwartz (BIC)	147,054

Les critères d'informations sont présentés en plus petit, disposant d'un meilleur format.[a]

a. Variable dépendante : Cool-sensitive Aδ-fibers Thresholds ΔT0-T1.

Effets fixes

Tests des effets fixes de type III[a]

Source	Ddl du numérateur	Ddl du dénominateur	F	Sig.
Constante	1	42	19,082	,000
Condition	1	42	,004	,947

a. Variable dépendante : Cool-sensitive Aδ-fibers Thresholds ΔT0-T1.

Estimations des effets fixes[a]

Paramètre	Estimation	Erreur standard	ddl	t	Sig.	Intervalle de confiance à 95 % Borne inférieure
Constante	,827841	,263974	42	3,136	,003	,295119
[Condition=Sham]	-,024943	,373316	42	-,067	,947	-,778325
[Condition=taVNS]	0[b]	0	.	.	.	.

Estimations des effets fixes[a]

Paramètre	Intervalle de confiance à 95 % Borne supérieure
Constante	1,360562
[Condition=Sham]	,728439
[Condition=taVNS]	.

a. Variable dépendante : Cool-sensitive Aδ-fibers Thresholds ΔT0-T1.

b. Ce paramètre est défini sur 0, car il est redondant.

Matrice de corrélation pour les estimations des effets fixes[a]

Paramètre	Constante	[Condition=Sham]	[Condition=taVNS]
Constante	1	-,707	.[b]
[Condition=Sham]	-,707	1	.[b]
[Condition=taVNS]	.[b]	.[b]	.[b]

a. Variable dépendante : Cool-sensitive Aδ-fibers Thresholds ΔT0-T1.

b. La corrélation est manquante par défaut, car elle est associée à un paramètre redondant.

Matrice de covariance pour les estimations des effets fixes[a]

Paramètre	Constante	[Condition=Sham]	[Condition=taVNS]

Constante	,069682	-,069682	0[b]
[Condition=Sham]	-,069682	,139365	0[b]
[Condition=taVNS]	0[b]	0[b]	0[b]

a. Variable dépendante : Cool-sensitive Aδ-fibers Thresholds ΔT0-T1.

b. La covariance est définie sur 0, car elle est associée à un paramètre redondant.

Paramètres de covariance

Estimations des paramètres de covariance[a]

Paramètre	Estimation	Erreur standard	Z de Wald	Sig.	Intervalle de confiance à 95 % Borne inférieure	Borne supérieure
Résidu	1,533012	,334531	4,583	,000	,999535	2,351218

a. Variable dépendante : Cool-sensitive Aδ-fibers Thresholds ΔT0-T1.

Matrice de corrélation pour les estimations des paramètres de covariance[a]

Paramètre	Résidu
Résidu	1

a. Variable dépendante : Cool-sensitive Aδ-fibers Thresholds ΔT0-T1.

Matrice de covariance pour les estimations des paramètres de covariance[a]

Paramètre	Résidu
Résidu	,111911

a. Variable dépendante : Cool-sensitive Aδ-fibers Thresholds ΔT0-T1.

Moyenne marginale estimée

Condition

Estimations[a]

Condition	Moyenne	Erreur standard	ddl	Intervalle de confiance à 95 % Borne inférieure	Borne supérieure
Sham	,803	,264	42	,270	1,336
taVNS	,828	,264	42	,295	1,361

a. Variable dépendante : Cool-sensitive Aδ-fibers Thresholds ΔT0-T1.

Comparaisons appariées[a]

(I) Condition	(J) Condition	Différence moyenne (I-J)	Erreur standard	ddl	Sig.[b]
Sham	taVNS	-,025	,373	42	,947
taVNS	Sham	,025	,373	42	,947

Comparaisons appariées[a]

(I) Condition	(J) Condition	Intervalle de confiance à 95 % pour la différence[b] Borne inférieure	Borne supérieure
Sham	taVNS	-,778	,728
taVNS	Sham	-,728	,778

Basées sur les moyennes marginales estimées[a]

a. Variable dépendante : Cool-sensitive Aδ-fibers Thresholds ΔT0-T1.

b. Ajustement pour les comparaisons multiples : Bonferroni.

Tests univariés[a]

Ddl du numérateur	Ddl du dénominateur	F	Sig.
1	42	,004	,947

Le test de F permet de tester l'effet de Condition. Il s'appuie sur les comparaisons appariées (indépendantes) linéaires parmi les moyennes marginales estimées.[a]

a. Variable dépendante : Cool-sensitive Aδ-fibers Thresholds ΔT0-T1.

1.8. Cool-sensitive Aδ-fibers Thresholds (ΔT0-T2).

```
MIXED CoolsensitiveAδfibersThresholdsΔT0T2 BY Condition
  /CRITERIA=CIN(95) MXITER(100) MXSTEP(10) SCORING(1)
SINGULAR(0.000000000001) HCONVERGE(0,
    ABSOLUTE) LCONVERGE(0, ABSOLUTE) PCONVERGE(0.000001, ABSOLUTE)
  /FIXED=Condition | SSTYPE(3)
  /METHOD=REML
  /PRINT=CPS CORB COVB DESCRIPTIVES G  SOLUTION TESTCOV
  /EMMEANS=TABLES(Condition) COMPARE ADJ(BONFERRONI).
```

Remarques

Sortie obtenue		05-MAY-2021 09:02:42
Commentaires		
Entrée	Jeu de données actif	Jeu_de_données1
	Filtre	<sans>
	Pondération	<sans>
	Fichier scindé	<sans>
	N de lignes dans le fichier de travail	44
Gestion des valeurs manquantes	Définition de la valeur manquante	Les valeurs manquantes définies par l'utilisateur sont traitées comme étant manquantes.
	Observations utilisées	Les statistiques sont basées sur toutes les observations comportant des données valides pour toutes les variables du modèle.

Syntaxe		MIXED CoolsensitiveAδfibersThresholdsΔT0T2 BY Condition /CRITERIA=CIN(95) MXITER(100) MXSTEP(10) SCORING(1) SINGULAR(0.000000000001) HCONVERGE(0, ABSOLUTE) LCONVERGE(0, ABSOLUTE) PCONVERGE(0.000001, ABSOLUTE) /FIXED=Condition | SSTYPE(3) /METHOD=REML /PRINT=CPS CORB COVB DESCRIPTIVES G SOLUTION TESTCOV /EMMEANS=TABLES(Condition) COMPARE ADJ(BONFERRONI).
Ressources	Temps de processeur	00:00:00,02
	Temps écoulé	00:00:00,01

Récapitulatif de traitement des observations

		Effectif	Pourcentage marginal
Condition	Sham	21	48,8%
	taVNS	22	51,2%
Valide		43	100,0%
Exclues		1	
Total		44	

Statistiques descriptives

Cool-sensitive Aδ-fibers Thresholds ΔT0-T2

Condition	Effectif	Moyenne	Ecart type	Coefficient de variation
Sham	21	,782738095238095	1,039572299302069	132,8%

taVNS	22	,813295454545455	1,203959585019077	148,0%
Total	43	,798372093023256	1,113383261425151	139,5%

Dimension du modèle[a]

		Nombre de niveaux	Nombre de paramètres
Effets fixes	Constante	1	1
	Condition	2	1
Résidu			1
Total		3	3

a. Variable dépendante : Cool-sensitive Aδ-fibers Thresholds ΔT0-T2.

Critères d'information[a]

Log de vraisemblance restreint -2	132,276
Critère d'information d'Akaike (AIC)	134,276
Critère de Hurvich et Tsai (AICC)	134,378
Critère de Bozdogan (CAIC)	136,989
Critère bayésien de Schwartz (BIC)	135,989

Les critères d'informations sont présentés en plus petit, disposant d'un meilleur format.[a]

a. Variable dépendante : Cool-sensitive Aδ-fibers Thresholds ΔT0-T2.

Effets fixes

Tests des effets fixes de type III[a]

Source	Ddl du numérateur	Ddl du dénominateur	F	Sig.
Constante	1	41	21,557	,000
Condition	1	41	,008	,930

a. Variable dépendante : Cool-sensitive Aδ-fibers Thresholds ΔT0-T2.

Estimations des effets fixes[a]

Paramètre	Estimation	Erreur standard	ddl	t	Sig.	Intervalle de confiance à 95 % Borne inférieure
Constante	,813295	,240228	41	3,386	,002	,328145
[Condition=Sham]	-,030557	,343755	41	-,089	,930	-,724784
[Condition=taVNS]	0[b]	0	.	.	.	.

Estimations des effets fixes[a]

Paramètre	Intervalle de confiance à 95 % Borne supérieure
Constante	1,298446
[Condition=Sham]	,663670
[Condition=taVNS]	.

a. Variable dépendante : Cool-sensitive Aδ-fibers Thresholds ΔT0-T2.

b. Ce paramètre est défini sur 0, car il est redondant.

Matrice de corrélation pour les estimations des effets fixes[a]

Paramètre	Constante	[Condition=Sham]	[Condition=taVNS]
Constante	1	-,699	.[b]
[Condition=Sham]	-,699	1	.[b]
[Condition=taVNS]	.[b]	.[b]	.[b]

a. Variable dépendante : Cool-sensitive Aδ-fibers Thresholds ΔT0-T2.

b. La corrélation est manquante par défaut, car elle est associée à un paramètre redondant.

Matrice de covariance pour les estimations des effets fixes[a]

Paramètre	Constante	[Condition=Sham]	[Condition=taVNS]
Constante	,057710	-,057710	0[b]
[Condition=Sham]	-,057710	,118167	0[b]
[Condition=taVNS]	0[b]	0[b]	0[b]

a. Variable dépendante : Cool-sensitive Aδ-fibers Thresholds ΔT0-T2.

b. La covariance est définie sur 0, car elle est associée à un paramètre redondant.

Paramètres de covariance

Estimations des paramètres de covariance[a]

Paramètre	Estimation	Erreur standard	Z de Wald	Sig.	Intervalle de confiance à 95 % Borne inférieure	Borne supérieure
Résidu	1,269612	,280410	4,528	,000	,823516	1,957357

a. Variable dépendante : Cool-sensitive Aδ-fibers Thresholds ΔT0-T2.

Matrice de corrélation pour les estimations des paramètres de covariance[a]

Paramètre	Résidu
Résidu	1

a. Variable dépendante : Cool-sensitive Aδ-fibers Thresholds ΔT0-T2.

Matrice de covariance pour les estimations des paramètres de covariance[a]

Paramètre	Résidu
Résidu	,078630

a. Variable dépendante : Cool-sensitive Aδ-fibers Thresholds ΔT0-T2.

Moyenne marginale estimée

Condition

Estimations[a]

Condition	Moyenne	Erreur standard	ddl	Intervalle de confiance à 95 % Borne inférieure	Borne supérieure
Sham	,783	,246	41	,286	1,279
taVNS	,813	,240	41	,328	1,298

a. Variable dépendante : Cool-sensitive Aδ-fibers Thresholds ΔT0-T2.

Comparaisons appariées[a]

(I) Condition	(J) Condition	Différence moyenne (I-J)	Erreur standard	ddl	Sig.[b]
Sham	taVNS	-,031	,344	41	,930
taVNS	Sham	,031	,344	41	,930

Comparaisons appariées[a]

(I) Condition	(J) Condition	Intervalle de confiance à 95 % pour la différence[b]	
		Borne inférieure	Borne supérieure
Sham	taVNS	-,725	,664
taVNS	Sham	-,664	,725

Basées sur les moyennes marginales estimées[a]

a. Variable dépendante : Cool-sensitive Aδ-fibers Thresholds ΔT0-T2.

b. Ajustement pour les comparaisons multiples : Bonferroni.

Tests univariés[a]

Ddl du numérateur	Ddl du dénominateur	F	Sig.
1	41	,008	,930

Le test de F permet de tester l'effet de Condition. Il s'appuie sur les comparaisons appariées (indépendantes) linéaires parmi les moyennes marginales estimées.[a]

a. Variable dépendante : Cool-sensitive Aδ-fibers Thresholds ΔT0-T2.

1.9. Laser Intensity (ΔT0-T1).

```
MIXED LaserIntensitiesΔT0T1 BY Condition
  /CRITERIA=CIN(95) MXITER(100) MXSTEP(10) SCORING(1)
SINGULAR(0.000000000001) HCONVERGE(0,
    ABSOLUTE) LCONVERGE(0, ABSOLUTE) PCONVERGE(0.000001, ABSOLUTE)
  /FIXED=Condition | SSTYPE(3)
  /METHOD=REML
  /PRINT=CPS CORB COVB DESCRIPTIVES G  SOLUTION TESTCOV
  /EMMEANS=TABLES(Condition) COMPARE ADJ(BONFERRONI).
```

Remarques

Sortie obtenue		05-MAY-2021 09:12:38
Commentaires		
Entrée	Jeu de données actif	Jeu_de_données1
	Filtre	<sans>
	Pondération	<sans>
	Fichier scindé	<sans>

	N de lignes dans le fichier de travail	44
Gestion des valeurs manquantes	Définition de la valeur manquante	Les valeurs manquantes définies par l'utilisateur sont traitées comme étant manquantes.
	Observations utilisées	Les statistiques sont basées sur toutes les observations comportant des données valides pour toutes les variables du modèle.
Syntaxe		MIXED LaserIntensitiesΔT0T1 BY Condition /CRITERIA=CIN(95) MXITER(100) MXSTEP(10) SCORING(1) SINGULAR(0.000000000001) HCONVERGE(0, ABSOLUTE) LCONVERGE(0, ABSOLUTE) PCONVERGE(0.000001, ABSOLUTE) /FIXED=Condition | SSTYPE(3) /METHOD=REML /PRINT=CPS CORB COVB DESCRIPTIVES G SOLUTION TESTCOV /EMMEANS=TABLES(Condition) COMPARE ADJ(BONFERRONI).
Ressources	Temps de processeur	00:00:00,02
	Temps écoulé	00:00:00,01

Récapitulatif de traitement des observations

		Effectif	Pourcentage marginal
Condition	Sham	22	50,0%

	taVNS	22	50,0%
Valide		44	100,0%
Exclues		0	
Total		44	

Statistiques descriptives

Laser Intensities ΔT0-T1

Condition	Effectif	Moyenne	Ecart type	Coefficient de variation
Sham	22	,167680484409091	,886737694321913	528,8%
taVNS	22	,038300387272727	1,120658528133782	2926,0%
Total	44	,102990435840909	1,000812415157106	971,8%

Dimension du modèle[a]

		Nombre de niveaux	Nombre de paramètres
Effets fixes	Constante	1	1
	Condition	2	1
Résidu			1
Total		3	3

a. Variable dépendante : Laser Intensities ΔT0-T1.

Critères d'information[a]

Log de vraisemblance restreint -2	126,249
Critère d'information d'Akaike (AIC)	128,249
Critère de Hurvich et Tsai (AICC)	128,349
Critère de Bozdogan (CAIC)	130,987
Critère bayésien de Schwartz (BIC)	129,987

Les critères d'informations sont présentés en plus petit, disposant d'un meilleur format.[a]

a. Variable dépendante : Laser Intensities ΔT0-T1.

Effets fixes

Tests des effets fixes de type III[a]

Source	Ddl du numérateur	Ddl du dénominateur	F	Sig.
Constante	1	42	,457	,503
Condition	1	42	,180	,673

a. Variable dépendante : Laser Intensities ΔT0-T1.

Estimations des effets fixes[a]

Paramètre	Estimation	Erreur standard	ddl	t	Sig.	Intervalle de confiance à 95 % Borne inférieure
Constante	,038300	,215437	42	,178	,860	-,396469
[Condition=Sham]	,129380	,304674	42	,425	,673	-,485477
[Condition=taVNS]	0[b]	0	.	.	.	.

Estimations des effets fixes[a]

Paramètre	Intervalle de confiance à 95 % Borne supérieure
Constante	,473070
[Condition=Sham]	,744237
[Condition=taVNS]	.

a. Variable dépendante : Laser Intensities ΔT0-T1.

b. Ce paramètre est défini sur 0, car il est redondant.

Matrice de corrélation pour les estimations des effets fixes[a]

Paramètre	Constante	[Condition=Sham]	[Condition=taVNS]
Constante	1	-,707	.[b]
[Condition=Sham]	-,707	1	.[b]
[Condition=taVNS]	.[b]	.[b]	.[b]

a. Variable dépendante : Laser Intensities ΔT0-T1.

b. La corrélation est manquante par défaut, car elle est associée à un paramètre redondant.

Matrice de covariance pour les estimations des effets fixes[a]

Paramètre	Constante	[Condition=Sham]	[Condition=taVNS]
Constante	,046413	-,046413	0[b]
[Condition=Sham]	-,046413	,092826	0[b]
[Condition=taVNS]	0[b]	0[b]	0[b]

a. Variable dépendante : Laser Intensities ΔT0-T1.

b. La covariance est définie sur 0, car elle est associée à un paramètre redondant.

Paramètres de covariance

Estimations des paramètres de covariance[a]

Paramètre	Estimation	Erreur standard	Z de Wald	Sig.	Intervalle de confiance à 95 %	
					Borne inférieure	Borne supérieure
Résidu	1,021090	,222820	4,583	,000	,665758	1,566070

a. Variable dépendante : Laser Intensities ΔT0-T1.

Matrice de corrélation pour les estimations des paramètres de covariance[a]

Paramètre	Résidu
Résidu	1

a. Variable dépendante : Laser Intensities ΔT0-T1.

Matrice de covariance pour les estimations des paramètres de covariance[a]

Paramètre	Résidu
Résidu	,049649

a. Variable dépendante : Laser Intensities ΔT0-T1.

Moyenne marginale estimée

Condition

Estimations[a]

Condition	Moyenne	Erreur standard	ddl	Intervalle de confiance à 95 % Borne inférieure	Borne supérieure
Sham	,168	,215	42	-,267	,602
taVNS	,038	,215	42	-,396	,473

a. Variable dépendante : Laser Intensities ΔT0-T1.

Comparaisons appariées[a]

(I) Condition	(J) Condition	Différence moyenne (I-J)	Erreur standard	ddl	Sig.[b]
Sham	taVNS	,129	,305	42	,673
taVNS	Sham	-,129	,305	42	,673

Comparaisons appariées[a]

(I) Condition	(J) Condition	Intervalle de confiance à 95 % pour la différence[b] Borne inférieure	Borne supérieure
Sham	taVNS	-,485	,744
taVNS	Sham	-,744	,485

Basées sur les moyennes marginales estimées[a]

a. Variable dépendante : Laser Intensities ΔT0-T1.

b. Ajustement pour les comparaisons multiples : Bonferroni.

Tests univariés[a]

Ddl du numérateur	Ddl du dénominateur	F	Sig.
1	42	,180	,673

Le test de F permet de tester l'effet de Condition. Il s'appuie sur les comparaisons appariées (indépendantes) linéaires parmi les moyennes marginales estimées.[a]

a. Variable dépendante : Laser Intensities ΔT0-T1.

1.10. Laser Intensity (ΔT0-T2).

```
MIXED LaserIntensitiesΔT0T2 BY Condition
  /CRITERIA=CIN(95) MXITER(100) MXSTEP(10) SCORING(1)
SINGULAR(0.000000000001) HCONVERGE(0,
    ABSOLUTE) LCONVERGE(0, ABSOLUTE) PCONVERGE(0.000001, ABSOLUTE)
  /FIXED=Condition | SSTYPE(3)
  /METHOD=REML
  /PRINT=CPS CORB COVB DESCRIPTIVES G  SOLUTION TESTCOV
  /EMMEANS=TABLES(Condition) COMPARE ADJ(BONFERRONI).
```

Remarques

Sortie obtenue		05-MAY-2021 09:13:47
Commentaires		
Entrée	Jeu de données actif	Jeu_de_données1
	Filtre	<sans>
	Pondération	<sans>
	Fichier scindé	<sans>
	N de lignes dans le fichier de travail	44
Gestion des valeurs manquantes	Définition de la valeur manquante	Les valeurs manquantes définies par l'utilisateur sont traitées comme étant manquantes.
	Observations utilisées	Les statistiques sont basées sur toutes les observations comportant des données valides pour toutes les variables du modèle.

Syntaxe		MIXED LaserIntensitiesΔT0T2 BY Condition<br>/CRITERIA=CIN(95) MXITER(100) MXSTEP(10) SCORING(1) SINGULAR(0.000000000001) HCONVERGE(0, ABSOLUTE) LCONVERGE(0, ABSOLUTE) PCONVERGE(0.000001, ABSOLUTE)<br>/FIXED=Condition | SSTYPE(3)<br>/METHOD=REML<br>/PRINT=CPS CORB COVB DESCRIPTIVES G SOLUTION TESTCOV<br>/EMMEANS=TABLES(Condition) COMPARE ADJ(BONFERRONI).
Ressources	Temps de processeur	00:00:00,02
	Temps écoulé	00:00:00,01

Récapitulatif de traitement des observations

		Effectif	Pourcentage marginal
Condition	Sham	22	50,0%
	taVNS	22	50,0%
Valide		44	100,0%
Exclues		0	
Total		44	

Statistiques descriptives

Laser Intensities ΔT0-T2

Condition	Effectif	Moyenne	Ecart type	Coefficient de variation
Sham	22	,211834549409091	1,292328156515841	610,1%

taVNS	22	,203302535000000	1,226024582123957	603,1%
Total	44	,207568542204546	1,244887366624374	599,7%

Dimension du modèle[a]

		Nombre de niveaux	Nombre de paramètres
Effets fixes	Constante	1	1
	Condition	2	1
Résidu			1
Total		3	3

a. Variable dépendante : Laser Intensities ΔT0-T2.

Critères d'information[a]

Log de vraisemblance restreint -2	144,760
Critère d'information d'Akaike (AIC)	146,760
Critère de Hurvich et Tsai (AICC)	146,860
Critère de Bozdogan (CAIC)	149,498
Critère bayésien de Schwartz (BIC)	148,498

Les critères d'informations sont présentés en plus petit, disposant d'un meilleur format.[a]

a. Variable dépendante : Laser Intensities ΔT0-T2.

Effets fixes

Tests des effets fixes de type III[a]

Source	Ddl du numérateur	Ddl du dénominateur	F	Sig.
Constante	1	42	1,195	,281
Condition	1	42	,001	,982

a. Variable dépendante : Laser Intensities ΔT0-T2.

Estimations des effets fixes[a]

Paramètre	Estimation	Erreur standard	ddl	t	Sig.	Intervalle de confiance à 95 % Borne inférieure
Constante	,203303	,268550	42	,757	,453	-,338654
[Condition=Sham]	,008532	,379788	42	,022	,982	-,757910
[Condition=taVNS]	0[b]	0	.	.	.	.

Estimations des effets fixes[a]

Paramètre	Intervalle de confiance à 95 % Borne supérieure
Constante	,745259
[Condition=Sham]	,774974
[Condition=taVNS]	.

a. Variable dépendante : Laser Intensities ΔT0-T2.

b. Ce paramètre est défini sur 0, car il est redondant.

Matrice de corrélation pour les estimations des effets fixes[a]

Paramètre	Constante	[Condition=Sham]	[Condition=taVNS]
Constante	1	-,707	.[b]
[Condition=Sham]	-,707	1	.[b]
[Condition=taVNS]	.[b]	.[b]	.[b]

a. Variable dépendante : Laser Intensities ΔT0-T2.

b. La corrélation est manquante par défaut, car elle est associée à un paramètre redondant.

Matrice de covariance pour les estimations des effets fixes[a]

Paramètre	Constante	[Condition=Sham]	[Condition=taVNS]
Constante	,072119	-,072119	0[b]
[Condition=Sham]	-,072119	,144239	0[b]
[Condition=taVNS]	0[b]	0[b]	0[b]

a. Variable dépendante : Laser Intensities ΔT0-T2.

b. La covariance est définie sur 0, car elle est associée à un paramètre redondant.

Paramètres de covariance

Estimations des paramètres de covariance[a]

Paramètre	Estimation	Erreur standard	Z de Wald	Sig.	Intervalle de confiance à 95 % Borne inférieure	Borne supérieure
Résidu	1,586624	,346230	4,583	,000	1,034491	2,433445

a. Variable dépendante : Laser Intensities ΔT0-T2.

Matrice de corrélation pour les estimations des paramètres de covariance[a]

Paramètre	Résidu
Résidu	1

a. Variable dépendante : Laser Intensities ΔT0-T2.

Matrice de covariance pour les estimations des paramètres de covariance[a]

Paramètre	Résidu
Résidu	,119875

a. Variable dépendante : Laser Intensities ΔT0-T2.

Moyenne marginale estimée

Condition

Estimations[a]

Condition	Moyenne	Erreur standard	ddl	Intervalle de confiance à 95 % Borne inférieure	Borne supérieure
Sham	,212	,269	42	-,330	,754
taVNS	,203	,269	42	-,339	,745

a. Variable dépendante : Laser Intensities ΔT0-T2.

Comparaisons appariées[a]

(I) Condition	(J) Condition	Différence moyenne (I-J)	Erreur standard	ddl	Sig.[b]
Sham	taVNS	,009	,380	42	,982
taVNS	Sham	-,009	,380	42	,982

Comparaisons appariées[a]

		Intervalle de confiance à 95 % pour la différence[b]	
(I) Condition	(J) Condition	Borne inférieure	Borne supérieure
Sham	taVNS	-,758	,775
taVNS	Sham	-,775	,758

Basées sur les moyennes marginales estimées[a]

a. Variable dépendante : Laser Intensities ΔT0-T2.

b. Ajustement pour les comparaisons multiples : Bonferroni.

Tests univariés[a]

Ddl du numérateur	Ddl du dénominateur	F	Sig.
1	42	,001	,982

Le test de F permet de tester l'effet de Condition. Il s'appuie sur les comparaisons appariées (indépendantes) linéaires parmi les moyennes marginales estimées.[a]

a. Variable dépendante : Laser Intensities ΔT0-T2.

1.11. Vibrotactile Intensity (ΔT0-T1).

```
MIXED VibrotactileIntensityΔT0T1 BY Condition
  /CRITERIA=CIN(95) MXITER(100) MXSTEP(10) SCORING(1)
SINGULAR(0.000000000001) HCONVERGE(0,
    ABSOLUTE) LCONVERGE(0, ABSOLUTE) PCONVERGE(0.000001, ABSOLUTE)
  /FIXED=Condition | SSTYPE(3)
  /METHOD=REML
  /PRINT=CPS CORB COVB DESCRIPTIVES G  SOLUTION TESTCOV
  /EMMEANS=TABLES(Condition) COMPARE ADJ(BONFERRONI).
```

Remarques

Sortie obtenue		05-MAY-2021 09:14:51
Commentaires		
Entrée	Jeu de données actif	Jeu_de_données1
	Filtre	<sans>
	Pondération	<sans>
	Fichier scindé	<sans>
	N de lignes dans le fichier de travail	44

Gestion des valeurs manquantes	Définition de la valeur manquante	Les valeurs manquantes définies par l'utilisateur sont traitées comme étant manquantes.
	Observations utilisées	Les statistiques sont basées sur toutes les observations comportant des données valides pour toutes les variables du modèle.
Syntaxe		MIXED VibrotactileIntensityΔT0T1 BY Condition /CRITERIA=CIN(95) MXITER(100) MXSTEP(10) SCORING(1) SINGULAR(0.000000000001) HCONVERGE(0, ABSOLUTE) LCONVERGE(0, ABSOLUTE) PCONVERGE(0.000001, ABSOLUTE) /FIXED=Condition | SSTYPE(3) /METHOD=REML /PRINT=CPS CORB COVB DESCRIPTIVES G SOLUTION TESTCOV /EMMEANS=TABLES(Condition) COMPARE ADJ(BONFERRONI).
Ressources	Temps de processeur	00:00:00,00
	Temps écoulé	00:00:00,01

Récapitulatif de traitement des observations

		Effectif	Pourcentage marginal
Condition	Sham	21	48,8%
	taVNS	22	51,2%
Valide		43	100,0%

Exclues	1	
Total	44	

Statistiques descriptives

Vibrotactile Intensity ΔT0-T1

Condition	Effectif	Moyenne	Ecart type	Coefficient de variation
Sham	21	-,556945980000000	,971623430440207	-174,5%
taVNS	22	,115909090909091	1,196636791340468	1032,4%
Total	43	-,212694548372093	1,131960282199060	-532,2%

Dimension du modèle[a]

		Nombre de niveaux	Nombre de paramètres
Effets fixes	Constante	1	1
	Condition	2	1
Résidu			1
Total		3	3

a. Variable dépendante : Vibrotactile Intensity ΔT0-T1.

Critères d'information[a]

Log de vraisemblance restreint -2	129,756
Critère d'information d'Akaike (AIC)	131,756
Critère de Hurvich et Tsai (AICC)	131,859
Critère de Bozdogan (CAIC)	134,470
Critère bayésien de Schwartz (BIC)	133,470

Les critères d'informations sont présentés en plus petit, disposant d'un meilleur format.[a]

a. Variable dépendante : Vibrotactile Intensity ΔT0-T1.

Effets fixes

Tests des effets fixes de type III[a]

Source	Ddl du numérateur	Ddl du dénominateur	F	Sig.
Constante	1	41	1,750	,193
Condition	1	41	4,074	,050

a. Variable dépendante : Vibrotactile Intensity ΔT0-T1.

Estimations des effets fixes[a]

Paramètre	Estimation	Erreur standard	ddl	t	Sig.	Intervalle de confiance à 95 % Borne inférieure
Constante	,115909	,232960	41	,498	,621	-,354563
[Condition=Sham]	-,672855	,333354	41	-2,018	,050	-1,346077
[Condition=taVNS]	0[b]	0	.	.	.	.

Estimations des effets fixes[a]

Paramètre	Intervalle de confiance à 95 % Borne supérieure
Constante	,586381
[Condition=Sham]	,000367
[Condition=taVNS]	.

a. Variable dépendante : Vibrotactile Intensity ΔT0-T1.

b. Ce paramètre est défini sur 0, car il est redondant.

Matrice de corrélation pour les estimations des effets fixes[a]

Paramètre	Constante	[Condition=Sham]	[Condition=taVNS]
Constante	1	-,699	.[b]
[Condition=Sham]	-,699	1	.[b]
[Condition=taVNS]	.[b]	.[b]	.[b]

a. Variable dépendante : Vibrotactile Intensity ΔT0-T1.

b. La corrélation est manquante par défaut, car elle est associée à un paramètre redondant.

Matrice de covariance pour les estimations des effets fixes[a]

Paramètre	Constante	[Condition=Sham]	[Condition=taVNS]
Constante	,054270	-,054270	0[b]
[Condition=Sham]	-,054270	,111125	0[b]
[Condition=taVNS]	0[b]	0[b]	0[b]

a. Variable dépendante : Vibrotactile Intensity ΔT0-T1.

b. La covariance est définie sur 0, car elle est associée à un paramètre redondant.

Paramètres de covarianc

Estimations des paramètres de covariance[a]

Paramètre	Estimation	Erreur standard	Z de Wald	Sig.	Intervalle de confiance à 95 %	
					Borne inférieure	Borne supérieure
Résidu	1,193946	,263698	4,528	,000	,774436	1,840702

a. Variable dépendante : Vibrotactile Intensity ΔT0-T1.

Matrice de corrélation pour les estimations des paramètres de covariance[a]

Paramètre	Résidu
Résidu	1

a. Variable dépendante : Vibrotactile Intensity ΔT0-T1.

Matrice de covariance pour les estimations des paramètres de covariance[a]

Paramètre	Résidu
Résidu	,069537

a. Variable dépendante :
Vibrotactile Intensity
ΔT0-T1.

Moyenne marginale estimée

Condition

Estimations[a]

Condition	Moyenne	Erreur standard	ddl	Intervalle de confiance à 95 % Borne inférieure	Borne supérieure
Sham	-,557	,238	41	-1,038	-,075
taVNS	,116	,233	41	-,355	,586

a. Variable dépendante : Vibrotactile Intensity ΔT0-T1.

Comparaisons appariées[a]

(I) Condition	(J) Condition	Différence moyenne (I-J)	Erreur standard	ddl	Sig.[b]
Sham	taVNS	-,673	,333	41	,050
taVNS	Sham	,673	,333	41	,050

Comparaisons appariées[a]

(I) Condition	(J) Condition	Intervalle de confiance à 95 % pour la différence[b] Borne inférieure	Borne supérieure
Sham	taVNS	-1,346	,000
taVNS	Sham	,000	1,346

Basées sur les moyennes marginales estimées[a]
a. Variable dépendante : Vibrotactile Intensity ΔT0-T1.
b. Ajustement pour les comparaisons multiples : Bonferroni.

Tests univariés[a]

Ddl du numérateur	Ddl du dénominateur	F	Sig.
1	41	4,074	,050

Le test de F permet de tester l'effet de Condition. Il s'appuie sur les comparaisons appariées (indépendantes) linéaires parmi les moyennes marginales estimées.[a]
a. Variable dépendante : Vibrotactile Intensity ΔT0-T1.

1.12. Vibrotactile Intensity (ΔT0-T2).

```
MIXED VibrotactileIntensityΔT0T2 BY Condition
  /CRITERIA=CIN(95) MXITER(100) MXSTEP(10) SCORING(1)
SINGULAR(0.000000000001) HCONVERGE(0,
    ABSOLUTE) LCONVERGE(0, ABSOLUTE) PCONVERGE(0.000001, ABSOLUTE)
  /FIXED=Condition | SSTYPE(3)
  /METHOD=REML
  /PRINT=CPS CORB COVB DESCRIPTIVES G  SOLUTION TESTCOV
  /EMMEANS=TABLES(Condition) COMPARE ADJ(BONFERRONI).
```

Remarques

Sortie obtenue		05-MAY-2021 09:24:17
Commentaires		
Entrée	Jeu de données actif	Jeu_de_données1
	Filtre	<sans>
	Pondération	<sans>
	Fichier scindé	<sans>
	N de lignes dans le fichier de travail	44
Gestion des valeurs manquantes	Définition de la valeur manquante	Les valeurs manquantes définies par l'utilisateur sont traitées comme étant manquantes.
	Observations utilisées	Les statistiques sont basées sur toutes les observations comportant des données valides pour toutes les variables du modèle.

Syntaxe		MIXED VibrotactileIntensityΔT0T2 BY Condition /CRITERIA=CIN(95) MXITER(100) MXSTEP(10) SCORING(1) SINGULAR(0.000000000001) HCONVERGE(0, ABSOLUTE) LCONVERGE(0, ABSOLUTE) PCONVERGE(0.000001, ABSOLUTE) /FIXED=Condition | SSTYPE(3) /METHOD=REML /PRINT=CPS CORB COVB DESCRIPTIVES G SOLUTION TESTCOV /EMMEANS=TABLES(Condition) COMPARE ADJ(BONFERRONI).
Ressources	Temps de processeur	00:00:00,03
	Temps écoulé	00:00:00,02

Récapitulatif de traitement des observations

		Effectif	Pourcentage marginal
Condition	Sham	21	48,8%
	taVNS	22	51,2%
Valide		43	100,0%
Exclues		1	
Total		44	

Statistiques descriptives

Vibrotactile Intensity ΔT0-T2

Condition	Effectif	Moyenne	Ecart type	Coefficient de variation
Sham	21	-,386328918095238	1,183325112888326	-306,3%

taVNS	22	-,009545454545455	1,358428634481163	-14231,2%
Total	43	-,193555983255814	1,275057128933047	-658,8%

Dimension du modèle[a]

		Nombre de niveaux	Nombre de paramètres
Effets fixes	Constante	1	1
	Condition	2	1
Résidu			1
Total		3	3

a. Variable dépendante : Vibrotactile Intensity ΔT0-T2.

Critères d'information[a]

Log de vraisemblance restreint -2	142,476
Critère d'information d'Akaike (AIC)	144,476
Critère de Hurvich et Tsai (AICC)	144,578
Critère de Bozdogan (CAIC)	147,189
Critère bayésien de Schwartz (BIC)	146,189

Les critères d'informations sont présentés en plus petit, disposant d'un meilleur format.[a]

a. Variable dépendante : Vibrotactile Intensity ΔT0-T2.

Effets fixes

Tests des effets fixes de type III[a]

Source	Ddl du numérateur	Ddl du dénominateur	F	Sig.
Constante	1	41	1,034	,315
Condition	1	41	,937	,339

a. Variable dépendante : Vibrotactile Intensity ΔT0-T2.

Estimations des effets fixes[a]

Paramètre	Estimation	Erreur standard	ddl	t	Sig.	Intervalle de confiance à 95 % Borne inférieure
Constante	-,009545	,272048	41	-,035	,972	-,558957
[Condition=Sham]	-,376783	,389287	41	-,968	,339	-1,162965
[Condition=taVNS]	0[b]	0	.	.	.	.

Estimations des effets fixes[a]

Paramètre	Intervalle de confiance à 95 % Borne supérieure
Constante	,539866
[Condition=Sham]	,409398
[Condition=taVNS]	.

a. Variable dépendante : Vibrotactile Intensity ΔT0-T2.

b. Ce paramètre est défini sur 0, car il est redondant.

Matrice de corrélation pour les estimations des effets fixes[a]

Paramètre	Constante	[Condition=Sham]	[Condition=taVNS]
Constante	1	-,699	.[b]
[Condition=Sham]	-,699	1	.[b]
[Condition=taVNS]	.[b]	.[b]	.[b]

a. Variable dépendante : Vibrotactile Intensity ΔT0-T2.

b. La corrélation est manquante par défaut, car elle est associée à un paramètre redondant.

Matrice de covariance pour les estimations des effets fixes[a]

Paramètre	Constante	[Condition=Sham]	[Condition=taVNS]
Constante	,074010	-,074010	0[b]
[Condition=Sham]	-,074010	,151544	0[b]
[Condition=taVNS]	0[b]	0[b]	0[b]

a. Variable dépendante : Vibrotactile Intensity ΔT0-T2.

b. La covariance est définie sur 0, car elle est associée à un paramètre redondant.

Paramètres de covariance

Estimations des paramètres de covariance[a]

Paramètre	Estimation	Erreur standard	Z de Wald	Sig.	Intervalle de confiance à 95 %	
					Borne inférieure	Borne supérieure
Résidu	1,628221	,359614	4,528	,000	1,056123	2,510223

a. Variable dépendante : Vibrotactile Intensity ΔT0-T2.

Matrice de corrélation pour les estimations des paramètres de covariance[a]

Paramètre	Résidu
Résidu	1

a. Variable dépendante : Vibrotactile Intensity ΔT0-T2.

Matrice de covariance pour les estimations des paramètres de covariance[a]

Paramètre	Résidu
Résidu	,129322

a. Variable dépendante : Vibrotactile Intensity ΔT0-T2.

Moyenne marginale estimée

Condition

Estimations[a]

Condition	Moyenne	Erreur standard	ddl	Intervalle de confiance à 95 % Borne inférieure	Borne supérieure
Sham	-,386	,278	41	-,949	,176
taVNS	-,010	,272	41	-,559	,540

a. Variable dépendante : Vibrotactile Intensity ΔT0-T2.

Comparaisons appariées[a]

(I) Condition	(J) Condition	Différence moyenne (I-J)	Erreur standard	ddl	Sig.[b]
Sham	taVNS	-,377	,389	41	,339
taVNS	Sham	,377	,389	41	,339

Comparaisons appariées[a]

(I) Condition	(J) Condition	Intervalle de confiance à 95 % pour la différence[b] Borne inférieure	Borne supérieure
Sham	taVNS	-1,163	,409
taVNS	Sham	-,409	1,163

Basées sur les moyennes marginales estimées[a]

a. Variable dépendante : Vibrotactile Intensity ΔT0-T2.

b. Ajustement pour les comparaisons multiples : Bonferroni.

Tests univariés[a]

Ddl du numérateur	Ddl du dénominateur	F	Sig.
1	41	,937	,339

Le test de F permet de tester l'effet de Condition. Il s'appuie sur les comparaisons appariées (indépendantes) linéaires parmi les moyennes marginales estimées.[a]

a. Variable dépendante : Vibrotactile Intensity ΔT0-T2.

1.13. Cool Intensity (ΔT0-T1).

```
MIXED CoolIntensityΔT0T1 BY Condition
  /CRITERIA=CIN(95) MXITER(100) MXSTEP(10) SCORING(1)
SINGULAR(0.000000000001) HCONVERGE(0,
    ABSOLUTE) LCONVERGE(0, ABSOLUTE) PCONVERGE(0.000001, ABSOLUTE)
  /FIXED=Condition | SSTYPE(3)
  /METHOD=REML
  /PRINT=CPS CORB COVB DESCRIPTIVES G  SOLUTION TESTCOV
  /EMMEANS=TABLES(Condition) COMPARE ADJ(BONFERRONI).
```

Remarques

Sortie obtenue		05-MAY-2021 09:29:11
Commentaires		
Entrée	Jeu de données actif	Jeu_de_données1
	Filtre	<sans>
	Pondération	<sans>
	Fichier scindé	<sans>
	N de lignes dans le fichier de travail	44
Gestion des valeurs manquantes	Définition de la valeur manquante	Les valeurs manquantes définies par l'utilisateur sont traitées comme étant manquantes.
	Observations utilisées	Les statistiques sont basées sur toutes les observations comportant des données valides pour toutes les variables du modèle.

Syntaxe		MIXED CoolIntensityΔT0T1 BY Condition<br>/CRITERIA=CIN(95) MXITER(100) MXSTEP(10) SCORING(1) SINGULAR(0.000000000001) HCONVERGE(0, ABSOLUTE) LCONVERGE(0, ABSOLUTE) PCONVERGE(0.000001, ABSOLUTE)<br>/FIXED=Condition | SSTYPE(3)<br>/METHOD=REML<br>/PRINT=CPS CORB COVB DESCRIPTIVES G SOLUTION TESTCOV<br>/EMMEANS=TABLES(Condition) COMPARE ADJ(BONFERRONI).
Ressources	Temps de processeur	00:00:00,00
	Temps écoulé	00:00:00,01

Récapitulatif de traitement des observations

		Effectif	Pourcentage marginal
Condition	Sham	22	50,0%
	taVNS	22	50,0%
Valide		44	100,0%
Exclues		0	
Total		44	

Statistiques descriptives

Cool Intensity ΔT0-T1

Condition	Effectif	Moyenne	Ecart type	Coefficient de variation
Sham	22	-,686673216772728	1,170194273926754	-170,4%

taVNS	22	-,252081486363636	1,619274074941044	-642,4%
Total	44	-,469377351568182	1,413366723031164	-301,1%

Dimension du modèle[a]

		Nombre de niveaux	Nombre de paramètres
Effets fixes	Constante	1	1
	Condition	2	1
Résidu			1
Total		3	3

a. Variable dépendante : Cool Intensity ΔT0-T1.

Critères d'information[a]

Log de vraisemblance restreint -2	154,395
Critère d'information d'Akaike (AIC)	156,395
Critère de Hurvich et Tsai (AICC)	156,495
Critère de Bozdogan (CAIC)	159,132
Critère bayésien de Schwartz (BIC)	158,132

Les critères d'informations sont présentés en plus petit, disposant d'un meilleur format.[a]

a. Variable dépendante : Cool Intensity ΔT0-T1.

Effets fixes

Tests des effets fixes de type III[a]

Source	Ddl du numérateur	Ddl du dénominateur	F	Sig.
Constante	1	42	4,857	,033
Condition	1	42	1,041	,313

a. Variable dépendante : Cool Intensity ΔT0-T1.

Estimations des effets fixes[a]

Paramètre	Estimation	Erreur standard	ddl	t	Sig.	Intervalle de confiance à 95 % Borne inférieure
Constante	-,252081	,301187	42	-,837	,407	-,859902
[Condition=Sham]	-,434592	,425943	42	-1,020	,313	-1,294179
[Condition=taVNS]	0[b]	0	.	.	.	.

Estimations des effets fixes[a]

Paramètre	Intervalle de confiance à 95 % Borne supérieure
Constante	,355739
[Condition=Sham]	,424996
[Condition=taVNS]	.

a. Variable dépendante : Cool Intensity ΔT0-T1.

b. Ce paramètre est défini sur 0, car il est redondant.

Matrice de corrélation pour les estimations des effets fixes[a]

Paramètre	Constante	[Condition=Sham]	[Condition=taVNS]
Constante	1	-,707	.[b]
[Condition=Sham]	-,707	1	.[b]
[Condition=taVNS]	.[b]	.[b]	.[b]

a. Variable dépendante : Cool Intensity ΔT0-T1.

b. La corrélation est manquante par défaut, car elle est associée à un paramètre redondant.

Matrice de covariance pour les estimations des effets fixes[a]

Paramètre	Constante	[Condition=Sham]	[Condition=taVNS]
Constante	,090714	-,090714	0[b]
[Condition=Sham]	-,090714	,181427	0[b]
[Condition=taVNS]	0[b]	0[b]	0[b]

a. Variable dépendante : Cool Intensity ΔT0-T1.

b. La covariance est définie sur 0, car elle est associée à un paramètre redondant.

Paramètres de covariance

Estimations des paramètres de covariance[a]

Paramètre	Estimation	Erreur standard	Z de Wald	Sig.	Intervalle de confiance à 95 % Borne inférieure	Borne supérieure
Résidu	1,995702	,435498	4,583	,000	1,301212	3,060857

a. Variable dépendante : Cool Intensity ΔT0-T1.

Matrice de corrélation pour les estimations des paramètres de covariance[a]

Paramètre	Résidu
Résidu	1

a. Variable dépendante : Cool Intensity ΔT0-T1.

Matrice de covariance pour les estimations des paramètres de covariance[a]

Paramètre	Résidu
Résidu	,189658

a. Variable dépendante : Cool Intensity ΔT0-T1.

Moyenne marginale estimée

Condition

Estimations[a]

Condition	Moyenne	Erreur standard	ddl	Intervalle de confiance à 95 % Borne inférieure	Borne supérieure
Sham	-,687	,301	42	-1,294	-,079
taVNS	-,252	,301	42	-,860	,356

a. Variable dépendante : Cool Intensity ΔT0-T1.

Comparaisons appariées[a]

(I) Condition	(J) Condition	Différence moyenne (I-J)	Erreur standard	ddl	Sig.[b]
Sham	taVNS	-,435	,426	42	,313
taVNS	Sham	,435	,426	42	,313

Comparaisons appariées[a]

(I) Condition	(J) Condition	Intervalle de confiance à 95 % pour la différence[b] Borne inférieure	Borne supérieure
Sham	taVNS	-1,294	,425
taVNS	Sham	-,425	1,294

Basées sur les moyennes marginales estimées[a]

a. Variable dépendante : Cool Intensity ΔT0-T1.

b. Ajustement pour les comparaisons multiples : Bonferroni.

Tests univariés[a]

Ddl du numérateur	Ddl du dénominateur	F	Sig.
1	42	1,041	,313

Le test de F permet de tester l'effet de Condition. Il s'appuie sur les comparaisons appariées (indépendantes) linéaires parmi les moyennes marginales estimées.[a]

a. Variable dépendante : Cool Intensity ΔT0-T1.

1.14. Cool Intensity (ΔT0-T2).

```
MIXED CoolIntensityΔT0T2 BY Condition
  /CRITERIA=CIN(95) MXITER(100) MXSTEP(10) SCORING(1)
SINGULAR(0.000000000001) HCONVERGE(0,
    ABSOLUTE) LCONVERGE(0, ABSOLUTE) PCONVERGE(0.000001, ABSOLUTE)
  /FIXED=Condition | SSTYPE(3)
  /METHOD=REML
  /PRINT=CPS CORB COVB DESCRIPTIVES G  SOLUTION TESTCOV
  /EMMEANS=TABLES(Condition) COMPARE ADJ(BONFERRONI).
```

Remarques

Sortie obtenue		05-MAY-2021 09:25:37
Commentaires		
Entrée	Jeu de données actif	Jeu_de_données1
	Filtre	<sans>
	Pondération	<sans>
	Fichier scindé	<sans>
	N de lignes dans le fichier de travail	44

Gestion des valeurs manquantes	Définition de la valeur manquante	Les valeurs manquantes définies par l'utilisateur sont traitées comme étant manquantes.
	Observations utilisées	Les statistiques sont basées sur toutes les observations comportant des données valides pour toutes les variables du modèle.
Syntaxe		MIXED CoolIntensityΔT0T2 BY Condition /CRITERIA=CIN(95) MXITER(100) MXSTEP(10) SCORING(1) SINGULAR(0.000000000001) HCONVERGE(0, ABSOLUTE) LCONVERGE(0, ABSOLUTE) PCONVERGE(0.000001, ABSOLUTE) /FIXED=Condition | SSTYPE(3) /METHOD=REML /PRINT=CPS CORB COVB DESCRIPTIVES G SOLUTION TESTCOV /EMMEANS=TABLES(Condition) COMPARE ADJ(BONFERRONI).
Ressources	Temps de processeur	00:00:00,02
	Temps écoulé	00:00:00,01

Récapitulatif de traitement des observations

		Effectif	Pourcentage marginal
Condition	Sham	22	50,0%
	taVNS	22	50,0%
Valide		44	100,0%
Exclues		0	

Total	44	

Statistiques descriptives

Cool Intensity ΔT0-T2

Condition	Effectif	Moyenne	Ecart type	Coefficient de variation
Sham	22	-1,152805944045455	1,229043407688672	-106,6%
taVNS	22	-,380670298000000	1,744487788243197	-458,3%
Total	44	-,766738121022727	1,541575909033226	-201,1%

Dimension du modèle[a]

		Nombre de niveaux	Nombre de paramètres
Effets fixes	Constante	1	1
	Condition	2	1
Résidu			1
Total		3	3

a. Variable dépendante : Cool Intensity ΔT0-T2.

Critères d'information[a]

Log de vraisemblance restreint -2	159,931
Critère d'information d'Akaike (AIC)	161,931
Critère de Hurvich et Tsai (AICC)	162,031
Critère de Bozdogan (CAIC)	164,669
Critère bayésien de Schwartz (BIC)	163,669

Les critères d'informations sont présentés en plus petit, disposant d'un meilleur format.[a]

a. Variable dépendante : Cool Intensity ΔT0-T2.

Effets fixes

Tests des effets fixes de type III[a]

Source	Ddl du numérateur	Ddl du dénominateur	F	Sig.
Constante	1	42	11,361	,002
Condition	1	42	2,880	,097

a. Variable dépendante : Cool Intensity ΔT0-T2.

Estimations des effets fixes[a]

Paramètre	Estimation	Erreur standard	ddl	t	Sig.	Intervalle de confiance à 95 % Borne inférieure
Constante	-,380670	,321707	42	-1,183	,243	-1,029900
[Condition=Sham]	-,772136	,454962	42	-1,697	,097	-1,690286
[Condition=taVNS]	0[b]	0	.	.	.	.

Estimations des effets fixes[a]

Paramètre	Intervalle de confiance à 95 % Borne supérieure
Constante	,268560
[Condition=Sham]	,146014
[Condition=taVNS]	.

a. Variable dépendante : Cool Intensity ΔT0-T2.

b. Ce paramètre est défini sur 0, car il est redondant.

Matrice de corrélation pour les estimations des effets fixes[a]

Paramètre	Constante	[Condition=Sham]	[Condition=taVNS]
Constante	1	-,707	.[b]
[Condition=Sham]	-,707	1	.[b]
[Condition=taVNS]	.[b]	.[b]	.[b]

a. Variable dépendante : Cool Intensity ΔT0-T2.

b. La corrélation est manquante par défaut, car elle est associée à un paramètre redondant.

Matrice de covariance pour les estimations des effets fixes[a]

Paramètre	Constante	[Condition=Sham]	[Condition=taVNS]

Constante	,103495	-,103495	0[b]
[Condition=Sham]	-,103495	,206990	0[b]
[Condition=taVNS]	0[b]	0[b]	0[b]

a. Variable dépendante : Cool Intensity ΔT0-T2.

b. La covariance est définie sur 0, car elle est associée à un paramètre redondant.

Paramètres de covariance

Estimations des paramètres de covariance[a]

Paramètre	Estimation	Erreur standard	Z de Wald	Sig.	Intervalle de confiance à 95 % Borne inférieure	Borne supérieure
Résidu	2,276893	,496859	4,583	,000	1,484551	3,492126

a. Variable dépendante : Cool Intensity ΔT0-T2.

Matrice de corrélation pour les estimations des paramètres de covariance[a]

Paramètre	Résidu
Résidu	1

a. Variable dépendante : Cool Intensity ΔT0-T2.

Matrice de covariance pour les estimations des paramètres de covariance[a]

Paramètre	Résidu
Résidu	,246869

a. Variable dépendante : Cool Intensity ΔT0-T2.

Moyenne marginale estimée

Condition

Estimations[a]

Condition	Moyenne	Erreur standard	ddl	Intervalle de confiance à 95 % Borne inférieure	Borne supérieure
Sham	-1,153	,322	42	-1,802	-,504
taVNS	-,381	,322	42	-1,030	,269

a. Variable dépendante : Cool Intensity ΔT0-T2.

Comparaisons appariées[a]

(I) Condition	(J) Condition	Différence moyenne (I-J)	Erreur standard	ddl	Sig.[b]
Sham	taVNS	-,772	,455	42	,097
taVNS	Sham	,772	,455	42	,097

Comparaisons appariées[a]

(I) Condition	(J) Condition	Intervalle de confiance à 95 % pour la différence[b] Borne inférieure	Borne supérieure
Sham	taVNS	-1,690	,146
taVNS	Sham	-,146	1,690

Basées sur les moyennes marginales estimées[a]

a. Variable dépendante : Cool Intensity ΔT0-T2.

b. Ajustement pour les comparaisons multiples : Bonferroni.

Tests univariés[a]

Ddl du numérateur	Ddl du dénominateur	F	Sig.
1	42	2,880	,097

Le test de F permet de tester l'effet de Condition. Il s'appuie sur les comparaisons appariées (indépendantes) linéaires parmi les moyennes marginales estimées.[a]

a. Variable dépendante : Cool Intensity ΔT0-T2.

1.15. Pinprick Intensity (ΔT0-T1).

```
MIXED PinprickIntensityΔT0T1 BY Condition
  /CRITERIA=CIN(95) MXITER(100) MXSTEP(10) SCORING(1)
SINGULAR(0.000000000001) HCONVERGE(0,
    ABSOLUTE) LCONVERGE(0, ABSOLUTE) PCONVERGE(0.000001, ABSOLUTE)
  /FIXED=Condition | SSTYPE(3)
  /METHOD=REML
  /PRINT=CPS CORB COVB DESCRIPTIVES G  SOLUTION TESTCOV
  /EMMEANS=TABLES(Condition) COMPARE ADJ(BONFERRONI).
```

Remarques

Sortie obtenue		05-MAY-2021 09:29:59
Commentaires		
Entrée	Jeu de données actif	Jeu_de_données1
	Filtre	<sans>
	Pondération	<sans>
	Fichier scindé	<sans>
	N de lignes dans le fichier de travail	44
Gestion des valeurs manquantes	Définition de la valeur manquante	Les valeurs manquantes définies par l'utilisateur sont traitées comme étant manquantes.
	Observations utilisées	Les statistiques sont basées sur toutes les observations comportant des données valides pour toutes les variables du modèle.

Syntaxe		MIXED PinprickIntensityΔT0T1 BY Condition<br>/CRITERIA=CIN(95) MXITER(100) MXSTEP(10) SCORING(1) SINGULAR(0.000000000001) HCONVERGE(0, ABSOLUTE) LCONVERGE(0, ABSOLUTE) PCONVERGE(0.000001, ABSOLUTE)<br>/FIXED=Condition | SSTYPE(3)<br>/METHOD=REML<br>/PRINT=CPS CORB COVB DESCRIPTIVES G SOLUTION TESTCOV<br>/EMMEANS=TABLES(Condition) COMPARE ADJ(BONFERRONI).
Ressources	Temps de processeur	00:00:00,02
	Temps écoulé	00:00:00,01

Récapitulatif de traitement des observations

		Effectif	Pourcentage marginal
Condition	Sham	22	50,0%
	taVNS	22	50,0%
Valide		44	100,0%
Exclues		0	
Total		44	

Statistiques descriptives

Pinprick Intensity ΔT0-T1

Condition	Effectif	Moyenne	Ecart type	Coefficient de variation
Sham	22	-,290789629272727	,531194772758536	-182,7%

taVNS	22	-,066332457272727	,856982896169882	-1292,0%
Total	44	-,178561043272727	,713695430973780	-399,7%

Dimension du modèle[a]

		Nombre de niveaux	Nombre de paramètres
Effets fixes	Constante	1	1
	Condition	2	1
Résidu			1
Total		3	3

a. Variable dépendante : Pinprick Intensity ΔT0-T1.

Critères d'information[a]

Log de vraisemblance restreint -2	96,952
Critère d'information d'Akaike (AIC)	98,952
Critère de Hurvich et Tsai (AICC)	99,052
Critère de Bozdogan (CAIC)	101,689
Critère bayésien de Schwartz (BIC)	100,689

Les critères d'informations sont présentés en plus petit, disposant d'un meilleur format.[a]

a. Variable dépendante : Pinprick Intensity ΔT0-T1.

Effets fixes

Tests des effets fixes de type III[a]

Source	Ddl du numérateur	Ddl du dénominateur	F	Sig.
Constante	1	42	2,760	,104
Condition	1	42	1,090	,302

a. Variable dépendante : Pinprick Intensity ΔT0-T1.

Estimations des effets fixes[a]

Paramètre	Estimation	Erreur standard	ddl	t	Sig.	Intervalle de confiance à 95 % Borne inférieure	Intervalle de confiance à 95 % Borne supérieure
Constante	-,066332	,152001	42	-,436	,665	-,373083	,240418
[Condition=Sham]	-,224457	,214962	42	-1,044	,302	-,658267	,209353
[Condition=taVNS]	0[b]	0	.	.	.	.	.

a. Variable dépendante : Pinprick Intensity ΔT0-T1.

b. Ce paramètre est défini sur 0, car il est redondant.

Matrice de corrélation pour les estimations des effets fixes[a]

Paramètre	Constante	[Condition=Sham]	[Condition=taVNS]
Constante	1	-,707	.[b]
[Condition=Sham]	-,707	1	.[b]
[Condition=taVNS]	.[b]	.[b]	.[b]

a. Variable dépendante : Pinprick Intensity ΔT0-T1.

b. La corrélation est manquante par défaut, car elle est associée à un paramètre redondant.

Matrice de covariance pour les estimations des effets fixes[a]

Paramètre	Constante	[Condition=Sham]	[Condition=taVNS]
Constante	,023104	-,023104	0[b]
[Condition=Sham]	-,023104	,046209	0[b]
[Condition=taVNS]	0[b]	0[b]	0[b]

a. Variable dépendante : Pinprick Intensity ΔT0-T1.

b. La covariance est définie sur 0, car elle est associée à un paramètre redondant.

Paramètres de covariance

Estimations des paramètres de covariance[a]

Paramètre	Estimation	Erreur standard	Z de Wald	Sig.	Intervalle de confiance à 95 % Borne inférieure	Borne supérieure
Résidu	,508294	,110919	4,583	,000	,331411	,779583

a. Variable dépendante : Pinprick Intensity ΔT0-T1.

Matrice de corrélation pour les estimations des paramètres de covariance[a]

Paramètre	Résidu
Résidu	1

a. Variable dépendante : Pinprick Intensity ΔT0-T1.

Matrice de covariance pour les estimations des paramètres de covariance[a]

Paramètre	Résidu
Résidu	,012303

a. Variable dépendante : Pinprick Intensity ΔT0-T1.

Moyenne marginale estimée

Condition

Estimations[a]

Condition	Moyenne	Erreur standard	ddl	Intervalle de confiance à 95 % Borne inférieure	Borne supérieure
Sham	-,291	,152	42	-,598	,016
taVNS	-,066	,152	42	-,373	,240

a. Variable dépendante : Pinprick Intensity ΔT0-T1.

Comparaisons appariées[a]

(I) Condition	(J) Condition	Différence moyenne (I-J)	Erreur standard	ddl	Sig.[b]
Sham	taVNS	-,224	,215	42	,302
taVNS	Sham	,224	,215	42	,302

Comparaisons appariées[a]

(I) Condition	(J) Condition	Intervalle de confiance à 95 % pour la différence[b] Borne inférieure	Borne supérieure
Sham	taVNS	-,658	,209
taVNS	Sham	-,209	,658

Basées sur les moyennes marginales estimées[a]

a. Variable dépendante : Pinprick Intensity ΔT0-T1.

b. Ajustement pour les comparaisons multiples : Bonferroni.

Tests univariés[a]

Ddl du numérateur	Ddl du dénominateur	F	Sig.
1	42	1,090	,302

Le test de F permet de tester l'effet de Condition. Il s'appuie sur les comparaisons appariées (indépendantes) linéaires parmi les moyennes marginales estimées.[a]

a. Variable dépendante : Pinprick Intensity ΔT0-T1.

1.16. Pinprick Intensity (ΔT0-T2).

```
MIXED PinprickIntensityΔT0T2 BY Condition
  /CRITERIA=CIN(95) MXITER(100) MXSTEP(10) SCORING(1)
SINGULAR(0.000000000001) HCONVERGE(0,
    ABSOLUTE) LCONVERGE(0, ABSOLUTE) PCONVERGE(0.000001, ABSOLUTE)
  /FIXED=Condition | SSTYPE(3)
  /METHOD=REML
  /PRINT=CPS CORB COVB DESCRIPTIVES G  SOLUTION TESTCOV
  /EMMEANS=TABLES(Condition) COMPARE ADJ(BONFERRONI).
```

Remarques

Sortie obtenue		05-MAY-2021 09:32:54
Commentaires		
Entrée	Jeu de données actif	Jeu_de_données1
	Filtre	<sans>
	Pondération	<sans>
	Fichier scindé	<sans>
	N de lignes dans le fichier de travail	44
Gestion des valeurs manquantes	Définition de la valeur manquante	Les valeurs manquantes définies par l'utilisateur sont traitées comme étant manquantes.
	Observations utilisées	Les statistiques sont basées sur toutes les observations comportant des données valides pour toutes les variables du modèle.

Syntaxe		MIXED PinprickIntensityΔT0T2 BY Condition<br>/CRITERIA=CIN(95) MXITER(100) MXSTEP(10) SCORING(1) SINGULAR(0.000000000001) HCONVERGE(0, ABSOLUTE) LCONVERGE(0, ABSOLUTE) PCONVERGE(0.000001, ABSOLUTE)<br>/FIXED=Condition | SSTYPE(3)<br>/METHOD=REML<br>/PRINT=CPS CORB COVB DESCRIPTIVES G SOLUTION TESTCOV<br>/EMMEANS=TABLES(Condition) COMPARE ADJ(BONFERRONI).
Ressources	Temps de processeur	00:00:00,02
	Temps écoulé	00:00:00,01

Récapitulatif de traitement des observations

		Effectif	Pourcentage marginal
Condition	Sham	22	50,0%
	taVNS	22	50,0%
Valide		44	100,0%
Exclues		0	
Total		44	

Statistiques descriptives

Pinprick Intensity ΔT0-T2

Condition	Effectif	Moyenne	Ecart type	Coefficient de variation
Sham	22	-,118687356545455	,485797714318563	-409,3%
taVNS	22	-,164110720454545	,731496254741052	-445,7%
Total	44	-,141399038500000	,614088587097774	-434,3%

Dimension du modèle[a]

		Nombre de niveaux	Nombre de paramètres
Effets fixes	Constante	1	1
	Condition	2	1
Résidu			1
Total		3	3

a. Variable dépendante : Pinprick Intensity ΔT0-T2.

Critères d'information[a]

Log de vraisemblance restreint -2	85,343
Critère d'information d'Akaike (AIC)	87,343
Critère de Hurvich et Tsai (AICC)	87,443
Critère de Bozdogan (CAIC)	90,080
Critère bayésien de Schwartz (BIC)	89,080

Les critères d'informations sont présentés en plus petit, disposant d'un meilleur format.[a]

a. Variable dépendante : Pinprick Intensity ΔT0-T2.

Effets fixes

Tests des effets fixes de type III[a]

Source	Ddl du numérateur	Ddl du dénominateur	F	Sig.
Constante	1	42	2,282	,138

Condition	1	42	,059	,809

a. Variable dépendante : Pinprick Intensity ΔT0-T2.

Estimations des effets fixes[a]

Paramètre	Estimation	Erreur standard	ddl	t	Sig.	Intervalle de confiance à 95 % Borne inférieure
Constante	-,164111	,132381	42	-1,240	,222	-,431266
[Condition=Sham]	,045423	,187215	42	,243	,809	-,332391
[Condition=taVNS]	0[b]	0	.	.	.	.

Estimations des effets fixes[a]

Paramètre	Intervalle de confiance à 95 % Borne supérieure
Constante	,103045
[Condition=Sham]	,423238
[Condition=taVNS]	.

a. Variable dépendante : Pinprick Intensity ΔT0-T2.

b. Ce paramètre est défini sur 0, car il est redondant.

Matrice de corrélation pour les estimations des effets fixes[a]

Paramètre	Constante	[Condition=Sham]	[Condition=taVNS]
Constante	1	-,707	.[b]
[Condition=Sham]	-,707	1	.[b]
[Condition=taVNS]	.[b]	.[b]	.[b]

a. Variable dépendante : Pinprick Intensity ΔT0-T2.

b. La corrélation est manquante par défaut, car elle est associée à un paramètre redondant.

Matrice de covariance pour les estimations des effets fixes[a]

Paramètre	Constante	[Condition=Sham]	[Condition=taVNS]
Constante	,017525	-,017525	0[b]
[Condition=Sham]	-,017525	,035049	0[b]
[Condition=taVNS]	0[b]	0[b]	0[b]

a. Variable dépendante : Pinprick Intensity ΔT0-T2.

b. La covariance est définie sur 0, car elle est associée à un paramètre redondant.

Paramètres de covariance

Estimations des paramètres de covariance[a]

Paramètre	Estimation	Erreur standard	Z de Wald	Sig.	Intervalle de confiance à 95 %	
					Borne inférieure	Borne supérieure
Résidu	,385543	,084132	4,583	,000	,251377	,591317

a. Variable dépendante : Pinprick Intensity ΔT0-T2.

Matrice de corrélation pour les estimations des paramètres de covariance[a]

Paramètre	Résidu
Résidu	1

a. Variable dépendante : Pinprick Intensity ΔT0-T2.

Matrice de covariance pour les estimations des paramètres de covariance[a]

Paramètre	Résidu
Résidu	,007078

a. Variable dépendante : Pinprick Intensity ΔT0-T2.

Moyenne marginale estimée

Condition

Estimations[a]

Condition	Moyenne	Erreur standard	ddl	Intervalle de confiance à 95 % Borne inférieure	Borne supérieure
Sham	-,119	,132	42	-,386	,148
taVNS	-,164	,132	42	-,431	,103

a. Variable dépendante : Pinprick Intensity ΔT0-T2.

Comparaisons appariées[a]

(I) Condition	(J) Condition	Différence moyenne (I-J)	Erreur standard	ddl	Sig.[b]
Sham	taVNS	,045	,187	42	,809
taVNS	Sham	-,045	,187	42	,809

Comparaisons appariées[a]

(I) Condition	(J) Condition	Intervalle de confiance à 95 % pour la différence[b] Borne inférieure	Borne supérieure
Sham	taVNS	-,332	,423
taVNS	Sham	-,423	,332

Basées sur les moyennes marginales estimées[a]

a. Variable dépendante : Pinprick Intensity ΔT0-T2.

b. Ajustement pour les comparaisons multiples : Bonferroni.

Tests univariés[a]

Ddl du numérateur	Ddl du dénominateur	F	Sig.
1	42	,059	,809

Le test de F permet de tester l'effet de Condition. Il s'appuie sur les comparaisons appariées (indépendantes) linéaires parmi les moyennes marginales estimées.[a]

a. Variable dépendante : Pinprick Intensity ΔT0-T2.

2. Experiment 2:

2.1. Heat-sensitive C-fibers Thresholds (ΔT0-T1).

```
DATASET NAME Jeu_de_données2 WINDOW=FRONT.
MIXED HeatsensitiveCfibersThresholdsΔT0T1 BY Condition
  /CRITERIA=CIN(95) MXITER(100) MXSTEP(10) SCORING(1)
SINGULAR(0.000000000001) HCONVERGE(0,
    ABSOLUTE) LCONVERGE(0, ABSOLUTE) PCONVERGE(0.000001, ABSOLUTE)
  /FIXED=Condition | SSTYPE(3)
  /METHOD=REML
  /PRINT=CPS CORB COVB DESCRIPTIVES G  SOLUTION TESTCOV
  /EMMEANS=TABLES(OVERALL)
  /EMMEANS=TABLES(Condition) COMPARE ADJ(BONFERRONI).
```

Remarques

Sortie obtenue		05-MAY-2021 09:41:24
Commentaires		
Entrée	Jeu de données actif	Jeu_de_données2
	Filtre	<sans>
	Pondération	<sans>
	Fichier scindé	<sans>
	N de lignes dans le fichier de travail	30
Gestion des valeurs manquantes	Définition de la valeur manquante	Les valeurs manquantes définies par l'utilisateur sont traitées comme étant manquantes.
	Observations utilisées	Les statistiques sont basées sur toutes les observations comportant des données valides pour toutes les variables du modèle.

Syntaxe		MIXED HeatsensitiveCfibersThresholdsΔT0T1 BY Condition /CRITERIA=CIN(95) MXITER(100) MXSTEP(10) SCORING(1) SINGULAR(0.000000000001) HCONVERGE(0, ABSOLUTE) LCONVERGE(0, ABSOLUTE) PCONVERGE(0.000001, ABSOLUTE) /FIXED=Condition | SSTYPE(3) /METHOD=REML /PRINT=CPS CORB COVB DESCRIPTIVES G SOLUTION TESTCOV /EMMEANS=TABLES(OVERALL) /EMMEANS=TABLES(Condition) COMPARE ADJ(BONFERRONI).
Ressources	Temps de processeur	00:00:00,02
	Temps écoulé	00:00:00,01

Récapitulatif de traitement des observations

		Effectif	Pourcentage marginal
Condition	Sham	14	51,9%
	taVNS	13	48,1%
Valide		27	100,0%
Exclues		3	
Total		30	

Statistiques descriptives

Heat-sensitive C-fibers Thresholds ΔT0-T1

Condition	Effectif	Moyenne	Ecart type	Coefficient de variation
Sham	14	-1,204428571428571	2,764360677633068	-229,5%
taVNS	13	-,575000000000000	2,215875485370662	-385,4%
Total	27	-,901370370370371	2,487922892512612	-276,0%

Dimension du modèle[a]

		Nombre de niveaux	Nombre de paramètres
Effets fixes	Constante	1	1
	Condition	2	1
Résidu			1
Total		3	3

a. Variable dépendante : Heat-sensitive C-fibers Thresholds ΔT0-T1.

Critères d'information[a]

Log de vraisemblance restreint -2	122,286
Critère d'information d'Akaike (AIC)	124,286
Critère de Hurvich et Tsai (AICC)	124,459
Critère de Bozdogan (CAIC)	126,504
Critère bayésien de Schwartz (BIC)	125,504

Les critères d'informations sont présentés en plus petit, disposant d'un meilleur format.[a]

a. Variable dépendante : Heat-sensitive C-fibers Thresholds ΔT0-T1.

Effets fixes

Tests des effets fixes de type III[a]

Source	Ddl du numérateur	Ddl du dénominateur	F	Sig.

Constante	1	25	3,372	,078
Condition	1	25	,422	,522

a. Variable dépendante : Heat-sensitive C-fibers Thresholds ΔT0-T1.

Estimations des effets fixes[a]

Paramètre	Estimation	Erreur standard	ddl	t	Sig.	Intervalle de confiance à 95 % Borne inférieure
Constante	-,575000	,697828	25	-,824	,418	-2,012203
[Condition=Sham]	-,629429	,969095	25	-,650	,522	-2,625316
[Condition=taVNS]	0[b]	0	.	.	.	.

Estimations des effets fixes[a]

Paramètre	Intervalle de confiance à 95 % Borne supérieure
Constante	,862203
[Condition=Sham]	1,366459
[Condition=taVNS]	.

a. Variable dépendante : Heat-sensitive C-fibers Thresholds ΔT0-T1.

b. Ce paramètre est défini sur 0, car il est redondant.

Matrice de corrélation pour les estimations des effets fixes[a]

Paramètre	Constante	[Condition=Sham]	[Condition=taVNS]
Constante	1	-,720	.[b]
[Condition=Sham]	-,720	1	.[b]
[Condition=taVNS]	.[b]	.[b]	.[b]

a. Variable dépendante : Heat-sensitive C-fibers Thresholds ΔT0-T1.

b. La corrélation est manquante par défaut, car elle est associée à un paramètre redondant.

Matrice de covariance pour les estimations des effets fixes[a]

Paramètre	Constante	[Condition=Sham]	[Condition=taVNS]
Constante	,486964	-,486964	0[b]
[Condition=Sham]	-,486964	,939144	0[b]
[Condition=taVNS]	0[b]	0[b]	0[b]

a. Variable dépendante : Heat-sensitive C-fibers Thresholds ΔT0-T1.
b. La covariance est définie sur 0, car elle est associée à un paramètre redondant.

Paramètres de covariance

Estimations des paramètres de covariance[a]

Paramètre	Estimation	Erreur standard	Z de Wald	Sig.	Intervalle de confiance à 95 % Borne inférieure	Intervalle de confiance à 95 % Borne supérieure
Résidu	6,330529	1,790544	3,536	,000	3,636502	11,020369

a. Variable dépendante : Heat-sensitive C-fibers Thresholds ΔT0-T1.

Matrice de corrélation pour les estimations des paramètres de covariance[a]

Paramètre	Résidu
Résidu	1

a. Variable dépendante : Heat-sensitive C-fibers Thresholds ΔT0-T1.

Matrice de covariance pour les estimations des paramètres de covariance[a]

Paramètre	Résidu
Résidu	3,206048

a. Variable dépendante : Heat-sensitive C-fibers Thresholds ΔT0-T1.

Moyenne marginale estimée

1. Grand Mean[a]

Moyenne	Erreur standard	ddl	Intervalle de confiance à 95 %

			Borne inférieure	Borne supérieure
-,890	,485	25	-1,888	,108

a. Variable dépendante : Heat-sensitive C-fibers Thresholds ΔT0-T1.

2. Condition

Estimations[a]

Condition	Moyenne	Erreur standard	ddl	Intervalle de confiance à 95 % Borne inférieure	Borne supérieure
Sham	-1,204	,672	25	-2,589	,180
taVNS	-,575	,698	25	-2,012	,862

a. Variable dépendante : Heat-sensitive C-fibers Thresholds ΔT0-T1.

Comparaisons appariées[a]

(I) Condition	(J) Condition	Différence moyenne (I-J)	Erreur standard	ddl	Sig.[b]
Sham	taVNS	-,629	,969	25	,522
taVNS	Sham	,629	,969	25	,522

Comparaisons appariées[a]

(I) Condition	(J) Condition	Intervalle de confiance à 95 % pour la différence[b] Borne inférieure	Borne supérieure
Sham	taVNS	-2,625	1,366
taVNS	Sham	-1,366	2,625

Basées sur les moyennes marginales estimées[a]

a. Variable dépendante : Heat-sensitive C-fibers Thresholds ΔT0-T1.

b. Ajustement pour les comparaisons multiples : Bonferroni.

Tests univariés[a]

Ddl du numérateur	Ddl du dénominateur	F	Sig.
1	25	,422	,522

Le test de F permet de tester l'effet de Condition. Il s'appuie sur les comparaisons appariées (indépendantes) linéaires parmi les moyennes marginales estimées.[a]

a. Variable dépendante : Heat-sensitive C-fibers Thresholds ΔT0-T1.

Heat-sensitive Aδ-fibers Thresholds (ΔT0-T1).

```
DATASET ACTIVATE Jeu_de_données2.
DATASET CLOSE Jeu_de_données1.
MIXED HeatsensitiveAδfibersThresholdsΔT0T1 BY Condition
  /CRITERIA=CIN(95) MXITER(100) MXSTEP(10) SCORING(1)
SINGULAR(0.000000000001) HCONVERGE(0,
    ABSOLUTE) LCONVERGE(0, ABSOLUTE) PCONVERGE(0.000001, ABSOLUTE)
  /FIXED=Condition | SSTYPE(3)
  /METHOD=REML
  /PRINT=CPS CORB COVB DESCRIPTIVES G  SOLUTION TESTCOV
  /EMMEANS=TABLES(OVERALL)
  /EMMEANS=TABLES(Condition) COMPARE ADJ(BONFERRONI).
```

Remarques

Sortie obtenue		05-MAY-2021 09:44:57
Commentaires		
Entrée	Jeu de données actif	Jeu_de_données2
	Filtre	<sans>
	Pondération	<sans>
	Fichier scindé	<sans>
	N de lignes dans le fichier de travail	30
Gestion des valeurs manquantes	Définition de la valeur manquante	Les valeurs manquantes définies par l'utilisateur sont traitées comme étant manquantes.
	Observations utilisées	Les statistiques sont basées sur toutes les observations comportant des données valides pour toutes les variables du modèle.

Syntaxe		MIXED HeatsensitiveAδfibersThresholdsΔT0T1 BY Condition<br>/CRITERIA=CIN(95) MXITER(100) MXSTEP(10) SCORING(1) SINGULAR(0.000000000001) HCONVERGE(0, ABSOLUTE) LCONVERGE(0, ABSOLUTE) PCONVERGE(0.000001, ABSOLUTE)<br>/FIXED=Condition | SSTYPE(3)<br>/METHOD=REML<br>/PRINT=CPS CORB COVB DESCRIPTIVES G SOLUTION TESTCOV<br>/EMMEANS=TABLES(OVERALL)<br>/EMMEANS=TABLES(Condition) COMPARE ADJ(BONFERRONI).
Ressources	Temps de processeur	00:00:00,02
	Temps écoulé	00:00:00,01

Récapitulatif de traitement des observations

		Effectif	Pourcentage marginal
Condition	Sham	14	51,9%
	taVNS	13	48,1%
Valide		27	100,0%
Exclues		3	
Total		30	

Statistiques descriptives

Heat-sensitive Aδ-fibers Thresholds ΔT0-T1

Condition	Effectif	Moyenne	Ecart type	Coefficient de variation
Sham	14	,133214285714286	1,775821161238737	1333,1%
taVNS	13	-,189038461538461	2,449878725412907	-1296,0%
Total	27	-,021944444444444	2,091364149642586	-9530,3%

Dimension du modèle[a]

		Nombre de niveaux	Nombre de paramètres
Effets fixes	Constante	1	1
	Condition	2	1
Résidu			1
Total		3	3

a. Variable dépendante : Heat-sensitive Aδ-fibers Thresholds ΔT0-T1.

Critères d'information[a]

Log de vraisemblance restreint -2	113,868
Critère d'information d'Akaike (AIC)	115,868
Critère de Hurvich et Tsai (AICC)	116,042
Critère de Bozdogan (CAIC)	118,087
Critère bayésien de Schwartz (BIC)	117,087

Les critères d'informations sont présentés en plus petit, disposant d'un meilleur format.[a]

a. Variable dépendante : Heat-sensitive Aδ-fibers Thresholds ΔT0-T1.

Effets fixes

Tests des effets fixes de type III[a]

Source	Ddl du numérateur	Ddl du dénominateur	F	Sig.
Constante	1	25	,005	,946
Condition	1	25	,155	,697

a. Variable dépendante : Heat-sensitive Aδ-fibers Thresholds ΔT0-T1.

Estimations des effets fixes[a]

Paramètre	Estimation	Erreur standard	ddl	t	Sig.	Intervalle de confiance à 95 %
						Borne inférieure
Constante	-,189038	,589704	25	-,321	,751	-1,403556
[Condition=Sham]	,322253	,818939	25	,394	,697	-1,364384
[Condition=taVNS]	0[b]	0	.	.	.	.

Estimations des effets fixes[a]

Paramètre	Intervalle de confiance à 95 %
	Borne supérieure
Constante	1,025479
[Condition=Sham]	2,008890
[Condition=taVNS]	.

a. Variable dépendante : Heat-sensitive Aδ-fibers Thresholds ΔT0-T1.

b. Ce paramètre est défini sur 0, car il est redondant.

Matrice de corrélation pour les estimations des effets fixes[a]

Paramètre	Constante	[Condition=Sham]	[Condition=taVNS]
Constante	1	-,720	.[b]
[Condition=Sham]	-,720	1	.[b]
[Condition=taVNS]	.[b]	.[b]	.[b]

a. Variable dépendante : Heat-sensitive Aδ-fibers Thresholds ΔT0-T1.

b. La corrélation est manquante par défaut, car elle est associée à un paramètre redondant.

Matrice de covariance pour les estimations des effets fixes[a]

Paramètre	Constante	[Condition=Sham]	[Condition=taVNS]
Constante	,347750	-,347750	0[b]
[Condition=Sham]	-,347750	,670662	0[b]
[Condition=taVNS]	0[b]	0[b]	0[b]

a. Variable dépendante : Heat-sensitive Aδ-fibers Thresholds ΔT0-T1.

b. La covariance est définie sur 0, car elle est associée à un paramètre redondant.

Paramètres de covariance

Estimations des paramètres de covariance[a]

Paramètre	Estimation	Erreur standard	Z de Wald	Sig.	Intervalle de confiance à 95 %	
					Borne inférieure	Borne supérieure
Résidu	4,520756	1,278663	3,536	,000	2,596898	7,869864

a. Variable dépendante : Heat-sensitive Aδ-fibers Thresholds ΔT0-T1.

Matrice de corrélation pour les estimations des paramètres de covariance[a]

Paramètre	Résidu
Résidu	1

a. Variable dépendante : Heat-sensitive Aδ-fibers Thresholds ΔT0-T1.

Matrice de covariance pour les estimations des paramètres de covariance[a]

Paramètre	Résidu
Résidu	1,634979

a. Variable dépendante :
Heat-sensitive Aδ-fibers
Thresholds ΔT0-T1.

Moyenne marginale estimée

1. Grand Mean[a]

			Intervalle de confiance à 95 %	
Moyenne	Erreur standard	ddl	Borne inférieure	Borne supérieure
-,028	,409	25	-,871	,815

a. Variable dépendante : Heat-sensitive Aδ-fibers Thresholds ΔT0-T1.

2. Condition

Estimations[a]

				Intervalle de confiance à 95 %	
Condition	Moyenne	Erreur standard	ddl	Borne inférieure	Borne supérieure
Sham	,133	,568	25	-1,037	1,304
taVNS	-,189	,590	25	-1,404	1,025

a. Variable dépendante : Heat-sensitive Aδ-fibers Thresholds ΔT0-T1.

Comparaisons appariées[a]

(I) Condition	(J) Condition	Différence moyenne (I-J)	Erreur standard	ddl	Sig.[b]
Sham	taVNS	,322	,819	25	,697
taVNS	Sham	-,322	,819	25	,697

Comparaisons appariées[a]

		Intervalle de confiance à 95 % pour la différence[b]	
(I) Condition	(J) Condition	Borne inférieure	Borne supérieure
Sham	taVNS	-1,364	2,009
taVNS	Sham	-2,009	1,364

Basées sur les moyennes marginales estimées[a]
a. Variable dépendante : Heat-sensitive Aδ-fibers Thresholds ΔT0-T1.
b. Ajustement pour les comparaisons multiples : Bonferroni.

Tests univariés[a]

Ddl du numérateur	Ddl du dénominateur	F	Sig.

1	25	,155	,697

Le test de F permet de tester l'effet de Condition. Il s'appuie sur les comparaisons appariées (indépendantes) linéaires parmi les moyennes marginales estimées.[a]

a. Variable dépendante : Heat-sensitive Aδ-fibers Thresholds ΔT0-T1.

2.3. Mechanosensitive Aβ-fibers Thresholds (ΔT0-T1).

```
MIXED MechanosensitiveAβfibersThresholdsΔT0T1 BY Condition
  /CRITERIA=CIN(95) MXITER(100) MXSTEP(10) SCORING(1)
SINGULAR(0.000000000001) HCONVERGE(0,
    ABSOLUTE) LCONVERGE(0, ABSOLUTE) PCONVERGE(0.000001, ABSOLUTE)
  /FIXED=Condition | SSTYPE(3)
  /METHOD=REML
  /PRINT=CPS CORB COVB DESCRIPTIVES G  SOLUTION TESTCOV
  /EMMEANS=TABLES(OVERALL)
  /EMMEANS=TABLES(Condition) COMPARE ADJ(BONFERRONI).
```

Remarques

Sortie obtenue		05-MAY-2021 09:46:36
Commentaires		
Entrée	Jeu de données actif	Jeu_de_données2
	Filtre	<sans>
	Pondération	<sans>
	Fichier scindé	<sans>
	N de lignes dans le fichier de travail	30
Gestion des valeurs manquantes	Définition de la valeur manquante	Les valeurs manquantes définies par l'utilisateur sont traitées comme étant manquantes.
	Observations utilisées	Les statistiques sont basées sur toutes les observations comportant des données valides pour toutes les variables du modèle.

Syntaxe		MIXED MechanosensitiveAβfibersThresholdsΔT0T1 BY Condition<br>/CRITERIA=CIN(95) MXITER(100) MXSTEP(10) SCORING(1) SINGULAR(0.000000000001) HCONVERGE(0, ABSOLUTE) LCONVERGE(0, ABSOLUTE) PCONVERGE(0.000001, ABSOLUTE)<br>/FIXED=Condition | SSTYPE(3)<br>/METHOD=REML<br>/PRINT=CPS CORB COVB DESCRIPTIVES G SOLUTION TESTCOV<br>/EMMEANS=TABLES(OVERALL)<br>/EMMEANS=TABLES(Condition) COMPARE ADJ(BONFERRONI).
Ressources	Temps de processeur	00:00:00,00
	Temps écoulé	00:00:00,01

Récapitulatif de traitement des observations

		Effectif	Pourcentage marginal
Condition	Sham	13	50,0%
	taVNS	13	50,0%
Valide		26	100,0%
Exclues		4	
Total		30	

Statistiques descriptives

Mechanosensitive Aβ-fibers Thresholds ΔT0-T1

Condition	Effectif	Moyenne	Ecart type	Coefficient de variation
Sham	13	,001663846153846	,010255580422760	616,4%
taVNS	13	-,001792307692308	,002900545649374	-161,8%
Total	26	-,000064230769231	,007591373089542	-11818,9%

Dimension du modèle[a]

		Nombre de niveaux	Nombre de paramètres
Effets fixes	Constante	1	1
	Condition	2	1
Résidu			1
Total		3	3

a. Variable dépendante : Mechanosensitive Aβ-fibers Thresholds ΔT0-T1.

Critères d'information[a]

Log de vraisemblance restreint -2	-161,387
Critère d'information d'Akaike (AIC)	-159,387
Critère de Hurvich et Tsai (AICC)	-159,205
Critère de Bozdogan (CAIC)	-157,208
Critère bayésien de Schwartz (BIC)	-158,208

Les critères d'informations sont présentés en plus petit, disposant d'un meilleur format.[a]

a. Variable dépendante : Mechanosensitive Aβ-fibers Thresholds ΔT0-T1.

Effets fixes

Tests des effets fixes de type III[a]

Source	Ddl du numérateur	Ddl du dénominateur	F	Sig.
Constante	1	24	,002	,966
Condition	1	24	1,367	,254

a. Variable dépendante : Mechanosensitive Aβ-fibers Thresholds ΔT0-T1.

Estimations des effets fixes[a]

Paramètre	Estimation	Erreur standard	ddl	t	Sig.	Intervalle de confiance à 95 % Borne inférieure
Constante	-,001792	,002090	24	-,857	,400	-,006106
[Condition=Sham]	,003456	,002956	24	1,169	,254	-,002645
[Condition=taVNS]	0[b]	0	.	.	.	.

Estimations des effets fixes[a]

Paramètre	Intervalle de confiance à 95 % Borne supérieure
Constante	,002522
[Condition=Sham]	,009557
[Condition=taVNS]	.

a. Variable dépendante : Mechanosensitive Aβ-fibers Thresholds ΔT0-T1.

b. Ce paramètre est défini sur 0, car il est redondant.

Matrice de corrélation pour les estimations des effets fixes[a]

Paramètre	Constante	[Condition=Sham]	[Condition=taVNS]
Constante	1	-,707	.[b]
[Condition=Sham]	-,707	1	.[b]
[Condition=taVNS]	.[b]	.[b]	.[b]

a. Variable dépendante : Mechanosensitive Aβ-fibers Thresholds ΔT0-T1.

b. La corrélation est manquante par défaut, car elle est associée à un paramètre redondant.

Matrice de covariance pour les estimations des effets fixes[a]

Paramètre	Constante	[Condition=Sham]	[Condition=taVNS]
Constante	4,368850E-6	-4,368850E-6	0[b]
[Condition=Sham]	-4,368850E-6	8,737700E-6	0[b]
[Condition=taVNS]	0[b]	0[b]	0[b]

a. Variable dépendante : Mechanosensitive Aβ-fibers Thresholds ΔT0-T1.

b. La covariance est définie sur 0, car elle est associée à un paramètre redondant.

Paramètres de covariance

Estimations des paramètres de covariance[a]

Paramètre	Estimation	Erreur standard	Z de Wald	Sig.	Intervalle de confiance à 95 %	
					Borne inférieure	Borne supérieure
Résidu	5,679505E-5	1,639532E-5	3,464	,001	3,225446E-5	,000100

a. Variable dépendante : Mechanosensitive Aβ-fibers Thresholds ΔT0-T1.

Matrice de corrélation pour les estimations des paramètres de covariance[a]

Paramètre	Résidu
Résidu	1

a. Variable dépendante : Mechanosensitive Aβ-fibers Thresholds ΔT0-T1.

Matrice de covariance pour les estimations des paramètres de covariance[a]

Paramètre	Résidu
Résidu	2,688065E-10

a. Variable dépendante : Mechanosensitive Aβ-fibers Thresholds ΔT0-T1.

Moyenne marginale estimée

1. Grand Mean[a]

Moyenne	Erreur standard	ddl	Intervalle de confiance à 95 % Borne inférieure	Borne supérieure
-6,423E-5	,001	24	-,003	,003

a. Variable dépendante : Mechanosensitive Aβ-fibers Thresholds ΔT0-T1.

2. Condition

Estimations[a]

Condition	Moyenne	Erreur standard	ddl	Intervalle de confiance à 95 % Borne inférieure	Borne supérieure
Sham	,002	,002	24	-,003	,006
taVNS	-,002	,002	24	-,006	,003

a. Variable dépendante : Mechanosensitive Aβ-fibers Thresholds ΔT0-T1.

Comparaisons appariées[a]

(I) Condition	(J) Condition	Différence moyenne (I-J)	Erreur standard	ddl	Sig.[b]
Sham	taVNS	,003	,003	24	,254
taVNS	Sham	-,003	,003	24	,254

Comparaisons appariées[a]

(I) Condition	(J) Condition	Intervalle de confiance à 95 % pour la différence[b] Borne inférieure	Borne supérieure
Sham	taVNS	-,003	,010
taVNS	Sham	-,010	,003

Basées sur les moyennes marginales estimées[a]

a. Variable dépendante : Mechanosensitive Aβ-fibers Thresholds ΔT0-T1.

b. Ajustement pour les comparaisons multiples : Bonferroni.

Tests univariés[a]

Ddl du numérateur	Ddl du dénominateur	F	Sig.
1	24	1,367	,254

Le test de F permet de tester l'effet de Condition. Il s'appuie sur les comparaisons appariées (indépendantes) linéaires parmi les moyennes marginales estimées.[a]

a. Variable dépendante : Mechanosensitive Aβ-fibers

Thresholds ΔT0-T1.

2.4. Laser Intensity (ΔOFF-ON).

```
MIXED LaserIntensityΔOFFON BY Condition
  /CRITERIA=CIN(95) MXITER(100) MXSTEP(10) SCORING(1)
SINGULAR(0.000000000001) HCONVERGE(0,
    ABSOLUTE) LCONVERGE(0, ABSOLUTE) PCONVERGE(0.000001, ABSOLUTE)
  /FIXED=Condition | SSTYPE(3)
  /METHOD=REML
  /PRINT=CPS CORB COVB DESCRIPTIVES G  SOLUTION TESTCOV
  /EMMEANS=TABLES(OVERALL)
  /EMMEANS=TABLES(Condition) COMPARE ADJ(BONFERRONI).
```

Remarques

Sortie obtenue		05-MAY-2021 09:48:57
Commentaires		
Entrée	Jeu de données actif	Jeu_de_données2
	Filtre	<sans>
	Pondération	<sans>
	Fichier scindé	<sans>
	N de lignes dans le fichier de travail	30
Gestion des valeurs manquantes	Définition de la valeur manquante	Les valeurs manquantes définies par l'utilisateur sont traitées comme étant manquantes.
	Observations utilisées	Les statistiques sont basées sur toutes les observations comportant des données valides pour toutes les variables du modèle.

Syntaxe		MIXED LaserIntensityΔOFFON BY Condition<br>/CRITERIA=CIN(95) MXITER(100) MXSTEP(10) SCORING(1) SINGULAR(0.000000000001) HCONVERGE(0, ABSOLUTE) LCONVERGE(0, ABSOLUTE) PCONVERGE(0.000001, ABSOLUTE)<br>/FIXED=Condition | SSTYPE(3)<br>/METHOD=REML<br>/PRINT=CPS CORB COVB DESCRIPTIVES G SOLUTION TESTCOV<br>/EMMEANS=TABLES(OVERALL)<br>/EMMEANS=TABLES(Condition) COMPARE ADJ(BONFERRONI).
Ressources	Temps de processeur	00:00:00,00
	Temps écoulé	00:00:00,01

Récapitulatif de traitement des observations

		Effectif	Pourcentage marginal
Condition	Sham	15	50,0%
	taVNS	15	50,0%
Valide		30	100,0%
Exclues		0	
Total		30	

Statistiques descriptives

Laser Intensity ΔOFF-ON

Condition	Effectif	Moyenne	Ecart type	Coefficient de variation
Sham	15	,028798948963482	,446796478582761	1551,4%
taVNS	15	-,042942766774562	,351632268210303	-818,8%
Total	30	-,007071908905540	,396728622083038	-5609,9%

Dimension du modèle[a]

		Nombre de niveaux	Nombre de paramètres
Effets fixes	Constante	1	1
	Condition	2	1
Résidu			1
Total		3	3

a. Variable dépendante : Laser Intensity ΔOFF-ON.

Critères d'information[a]

Log de vraisemblance restreint -2	33,849
Critère d'information d'Akaike (AIC)	35,849
Critère de Hurvich et Tsai (AICC)	36,003
Critère de Bozdogan (CAIC)	38,181
Critère bayésien de Schwartz (BIC)	37,181

Les critères d'informations sont présentés en plus petit, disposant d'un meilleur format.[a]

a. Variable dépendante : Laser Intensity ΔOFF-ON.

Effets fixes

Tests des effets fixes de type III[a]

Source	Ddl du numérateur	Ddl du dénominateur	F	Sig.
Constante	1	28	,009	,924
Condition	1	28	,239	,629

a. Variable dépendante : Laser Intensity ΔOFF-ON.

Estimations des effets fixes[a]

Paramètre	Estimation	Erreur standard	ddl	t	Sig.	Intervalle de confiance à 95 % Borne inférieure
Constante	-,042943	,103806	28	-,414	,682	-,255580
[Condition=Sham]	,071742	,146804	28	,489	,629	-,228973
[Condition=taVNS]	0[b]	0	.	.	.	.

Estimations des effets fixes[a]

Paramètre	Intervalle de confiance à 95 % Borne supérieure
Constante	,169695
[Condition=Sham]	,372457
[Condition=taVNS]	.

a. Variable dépendante : Laser Intensity ΔOFF-ON.

b. Ce paramètre est défini sur 0, car il est redondant.

Matrice de corrélation pour les estimations des effets fixes[a]

Paramètre	Constante	[Condition=Sham]	[Condition=taVNS]
Constante	1	-,707	.[b]
[Condition=Sham]	-,707	1	.[b]
[Condition=taVNS]	.[b]	.[b]	.[b]

a. Variable dépendante : Laser Intensity ΔOFF-ON.

b. La corrélation est manquante par défaut, car elle est associée à un paramètre redondant.

Matrice de covariance pour les estimations des effets fixes[a]

Paramètre	Constante	[Condition=Sham]	[Condition=taVNS]
Constante	,010776	-,010776	0[b]
[Condition=Sham]	-,010776	,021551	0[b]
[Condition=taVNS]	0[b]	0[b]	0[b]

a. Variable dépendante : Laser Intensity ΔOFF-ON.

b. La covariance est définie sur 0, car elle est associée à un paramètre redondant.

Paramètres de covariance

Estimations des paramètres de covariance[a]

Paramètre	Estimation	Erreur standard	Z de Wald	Sig.	Intervalle de confiance à 95 %	
					Borne inférieure	Borne supérieure
Résidu	,161636	,043199	3,742	,000	,095729	,272918

a. Variable dépendante : Laser Intensity ΔOFF-ON.

Matrice de corrélation pour les estimations des paramètres de covariance[a]

Paramètre	Résidu
Résidu	1

a. Variable dépendante : Laser Intensity ΔOFF-ON.

Matrice de covariance pour les estimations des paramètres de covariance[a]

Paramètre	Résidu
Résidu	,001866

a. Variable dépendante : Laser Intensity ΔOFF-ON.

Moyenne marginale estimée

1. Grand Mean[a]

Moyenne	Erreur standard	ddl	Intervalle de confiance à 95 %	
			Borne inférieure	Borne supérieure
-,007	,073	28	-,157	,143

a. Variable dépendante : Laser Intensity ΔOFF-ON.

2. Condition

Estimations[a]

Condition	Moyenne	Erreur standard	ddl	Intervalle de confiance à 95 % Borne inférieure	Borne supérieure
Sham	,029	,104	28	-,184	,241
taVNS	-,043	,104	28	-,256	,170

a. Variable dépendante : Laser Intensity ΔOFF-ON.

Comparaisons appariées[a]

(I) Condition	(J) Condition	Différence moyenne (I-J)	Erreur standard	ddl	Sig.[b]
Sham	taVNS	,072	,147	28	,629
taVNS	Sham	-,072	,147	28	,629

Comparaisons appariées[a]

(I) Condition	(J) Condition	Intervalle de confiance à 95 % pour la différence[b] Borne inférieure	Borne supérieure
Sham	taVNS	-,229	,372
taVNS	Sham	-,372	,229

Basées sur les moyennes marginales estimées[a]

a. Variable dépendante : Laser Intensity ΔOFF-ON.

b. Ajustement pour les comparaisons multiples : Bonferroni.

Tests univariés[a]

Ddl du numérateur	Ddl du dénominateur	F	Sig.
1	28	,239	,629

Le test de F permet de tester l'effet de Condition. Il s'appuie sur les comparaisons appariées (indépendantes) linéaires parmi les moyennes marginales estimées.[a]

a. Variable dépendante : Laser Intensity ΔOFF-ON.

2.5. Vibrotactile Intensity (ΔOFF-ON).

```
MIXED VibrotactileIntensityΔOFFON BY Condition
  /CRITERIA=CIN(95) MXITER(100) MXSTEP(10) SCORING(1)
SINGULAR(0.000000000001) HCONVERGE(0,
    ABSOLUTE) LCONVERGE(0, ABSOLUTE) PCONVERGE(0.000001, ABSOLUTE)
  /FIXED=Condition | SSTYPE(3)
  /METHOD=REML
  /PRINT=CPS CORB COVB DESCRIPTIVES G  SOLUTION TESTCOV
  /EMMEANS=TABLES(OVERALL)
  /EMMEANS=TABLES(Condition) COMPARE ADJ(BONFERRONI).
```

Remarques

Sortie obtenue		05-MAY-2021 09:50:50
Commentaires		
Entrée	Jeu de données actif	Jeu_de_données2
	Filtre	<sans>
	Pondération	<sans>
	Fichier scindé	<sans>
	N de lignes dans le fichier de travail	30
Gestion des valeurs manquantes	Définition de la valeur manquante	Les valeurs manquantes définies par l'utilisateur sont traitées comme étant manquantes.
	Observations utilisées	Les statistiques sont basées sur toutes les observations comportant des données valides pour toutes les variables du modèle.

Syntaxe		MIXED VibrotactileIntensityΔOFFON BY Condition<br>/CRITERIA=CIN(95) MXITER(100) MXSTEP(10) SCORING(1) SINGULAR(0.000000000001) HCONVERGE(0, ABSOLUTE) LCONVERGE(0, ABSOLUTE) PCONVERGE(0.000001, ABSOLUTE)<br>/FIXED=Condition | SSTYPE(3)<br>/METHOD=REML<br>/PRINT=CPS CORB COVB DESCRIPTIVES G SOLUTION TESTCOV<br>/EMMEANS=TABLES(OVERALL)<br>/EMMEANS=TABLES(Condition) COMPARE ADJ(BONFERRONI).
Ressources	Temps de processeur	00:00:00,02
	Temps écoulé	00:00:00,01

Récapitulatif de traitement des observations

		Effectif	Pourcentage marginal
Condition	Sham	15	50,0%
	taVNS	15	50,0%
Valide		30	100,0%
Exclues		0	
Total		30	

Statistiques descriptives

Vibrotactile Intensity ΔOFF-ON

Condition	Effectif	Moyenne	Ecart type	Coefficient de variation
Sham	15	,073785905260441	,207055896706353	280,6%
taVNS	15	,106920455309939	,150421198020340	140,7%
Total	30	,090353180285190	,178616886727144	197,7%

Dimension du modèle[a]

		Nombre de niveaux	Nombre de paramètres
Effets fixes	Constante	1	1
	Condition	2	1
Résidu			1
Total		3	3

a. Variable dépendante : Vibrotactile Intensity ΔOFF-ON.

Critères d'information[a]

Log de vraisemblance restreint -2	-10,852
Critère d'information d'Akaike (AIC)	-8,852
Critère de Hurvich et Tsai (AICC)	-8,698
Critère de Bozdogan (CAIC)	-6,520
Critère bayésien de Schwartz (BIC)	-7,520

Les critères d'informations sont présentés en plus petit, disposant d'un meilleur format.[a]

a. Variable dépendante : Vibrotactile Intensity ΔOFF-ON.

Effets fixes

Tests des effets fixes de type III[a]

Source	Ddl du numérateur	Ddl du dénominateur	F	Sig.
Constante	1	28	7,478	,011
Condition	1	28	,251	,620

a. Variable dépendante : Vibrotactile Intensity ΔOFF-ON.

Estimations des effets fixes[a]

Paramètre	Estimation	Erreur standard	ddl	t	Sig.	Intervalle de confiance à 95 % Borne inférieure
Constante	,106920	,046726	28	2,288	,030	,011207
[Condition=Sham]	-,033135	,066080	28	-,501	,620	-,168493
[Condition=taVNS]	0[b]	0	.	.	.	.

Estimations des effets fixes[a]

Paramètre	Intervalle de confiance à 95 % Borne supérieure
Constante	,202634
[Condition=Sham]	,102224
[Condition=taVNS]	.

a. Variable dépendante : Vibrotactile Intensity ΔOFF-ON.

b. Ce paramètre est défini sur 0, car il est redondant.

Matrice de corrélation pour les estimations des effets fixes[a]

Paramètre	Constante	[Condition=Sham]	[Condition=taVNS]
Constante	1	-,707	.[b]
[Condition=Sham]	-,707	1	.[b]
[Condition=taVNS]	.[b]	.[b]	.[b]

a. Variable dépendante : Vibrotactile Intensity ΔOFF-ON.

b. La corrélation est manquante par défaut, car elle est associée à un paramètre redondant.

Matrice de covariance pour les estimations des effets fixes[a]

Paramètre	Constante	[Condition=Sham]	[Condition=taVNS]
Constante	,002183	-,002183	0[b]
[Condition=Sham]	-,002183	,004367	0[b]
[Condition=taVNS]	0[b]	0[b]	0[b]

a. Variable dépendante : Vibrotactile Intensity ΔOFF-ON.

b. La covariance est définie sur 0, car elle est associée à un paramètre redondant.

Paramètres de covariance

Estimations des paramètres de covariance[a]

Paramètre	Estimation	Erreur standard	Z de Wald	Sig.	Intervalle de confiance à 95 %	
					Borne inférieure	Borne supérieure
Résidu	,032749	,008753	3,742	,000	,019396	,055296

a. Variable dépendante : Vibrotactile Intensity ΔOFF-ON.

Matrice de corrélation pour les estimations des paramètres de covariance[a]

Paramètre	Résidu
Résidu	1

a. Variable dépendante : Vibrotactile Intensity ΔOFF-ON.

Matrice de covariance pour les estimations des paramètres de covariance[a]

Paramètre	Résidu
Résidu	7,660852E-5

a. Variable dépendante : Vibrotactile Intensity ΔOFF-ON.

Moyenne marginale estimée

1. Grand Mean[a]

Moyenne	Erreur standard	ddl	Intervalle de confiance à 95 % Borne inférieure	Borne supérieure
,090	,033	28	,023	,158

a. Variable dépendante : Vibrotactile Intensity ΔOFF-ON.

2. Condition

Estimations[a]

Condition	Moyenne	Erreur standard	ddl	Intervalle de confiance à 95 % Borne inférieure	Borne supérieure
Sham	,074	,047	28	-,022	,169
taVNS	,107	,047	28	,011	,203

a. Variable dépendante : Vibrotactile Intensity ΔOFF-ON.

Comparaisons appariées[a]

(I) Condition	(J) Condition	Différence moyenne (I-J)	Erreur standard	ddl	Sig.[b]
Sham	taVNS	-,033	,066	28	,620
taVNS	Sham	,033	,066	28	,620

Comparaisons appariées[a]

(I) Condition	(J) Condition	Intervalle de confiance à 95 % pour la différence[b] Borne inférieure	Borne supérieure
Sham	taVNS	-,168	,102
taVNS	Sham	-,102	,168

Basées sur les moyennes marginales estimées[a]

a. Variable dépendante : Vibrotactile Intensity ΔOFF-ON.

b. Ajustement pour les comparaisons multiples : Bonferroni.

Tests univariés[a]

Ddl du numérateur	Ddl du dénominateur	F	Sig.
1	28	,251	,620

Le test de F permet de tester l'effet de Condition. Il s'appuie sur les comparaisons appariées (indépendantes) linéaires parmi les moyennes marginales estimées.[a]

a. Variable dépendante : Vibrotactile Intensity ΔOFF-ON.
