# Supplementary material for: Transcutaneous auricular VNS applied to experimental pain: A paired behavioral and EEG study using thermonociceptive CO2 laser
Source: PLoS One. 2021 Jul 12;16(7):e0254480. doi: 10.1371/journal.pone.0254480 (PMC8274876; doi:10.1371/journal.pone.0254480)
Supplement: S1 Appendix — (ZIP) [file pone.0254480.s001.zip › Supplementary Analysis_LMM_Behavioral Responses.pdf]

Transcutaneous VNS applied to experimental pain: a paired behavioral and EEG study using thermonociceptive CO2 laser

Supplementary Appendix

Linear Mixed Models: Detailed analysis.

BEHAVIORAL RESPONSES

1. Experiment 1:

1.1.Heat-sensitive C-fibers Detection Thresholds.

```
MIXED HeatsensitiveCfibersThresholds BY Condition Time
  /CRITERIA=CIN(95) MXITER(100) MXSTEP(10) SCORING(1)
SINGULAR(0.000000000001) HCONVERGE(0,
    ABSOLUTE) LCONVERGE(0, ABSOLUTE) PCONVERGE(0.000001, ABSOLUTE)
  /FIXED=Condition Time Condition*Time | SSTYPE(3)
  /METHOD=REML
  /PRINT=CPS CORB COVB DESCRIPTIVES  SOLUTION TESTCOV
  /EMMEANS=TABLES(Condition) COMPARE ADJ(BONFERRONI)
  /EMMEANS=TABLES(Time) COMPARE ADJ(BONFERRONI)
  /EMMEANS=TABLES(Condition*Time) .
```

Remarques

Sortie obtenue		04-MAY-2021 13:41:16
Commentaires		
Entrée	Jeu de données actif	Jeu_de_données1
	Filtre	<sans>
	Pondération	<sans>
	Fichier scindé	<sans>
	N de lignes dans le fichier de travail	1048509
Gestion des valeurs manquantes	Définition de la valeur manquante	Les valeurs manquantes définies par l'utilisateur sont traitées comme étant manquantes.
	Observations utilisées	Les statistiques sont basées sur toutes les observations comportant des données valides pour toutes les variables du modèle.

Syntaxe		MIXED HeatsensitiveCfibersThresholds BY Condition Time<br>/CRITERIA=CIN(95) MXITER(100) MXSTEP(10) SCORING(1) SINGULAR(0.000000000001) HCONVERGE(0, ABSOLUTE) LCONVERGE(0, ABSOLUTE) PCONVERGE(0.000001, ABSOLUTE)<br>/FIXED=Condition Time Condition*Time | SSTYPE(3)<br>/METHOD=REML<br>/PRINT=CPS CORB COVB DESCRIPTIVES SOLUTION TESTCOV<br>/EMMEANS=TABLES(Condition) COMPARE ADJ(BONFERRONI)<br>/EMMEANS=TABLES(Time) COMPARE ADJ(BONFERRONI)<br>/EMMEANS=TABLES(Condition*Time) .
Ressources	Temps de processeur	00:00:00,69
	Temps écoulé	00:00:00,68

Récapitulatif de traitement des observations

		Effectif	Pourcentage marginal
Condition	Sham	65	49,6%
	taVNS	66	50,4%
Time	T0	44	33,6%
	T1	44	33,6%

T2	43	32,8%
Valide	131	100,0%
Exclues	1048378	
Total	1048509	

Statistiques descriptives

Heat-sensitive C-fibers Thresholds

Condition	Time	Effectif	Moyenne	Ecart type	Coefficient de variation
Sham	T0	22	40,721591	1,4774756	3,6%
	T1	22	40,551136	1,9751654	4,9%
	T2	21	40,255952	2,8293871	7,0%
	Total	65	40,513462	2,1296047	5,3%
taVNS	T0	22	41,585227	1,6944352	4,1%
	T1	22	41,144886	1,4690997	3,6%
	T2	22	41,340909	2,0171076	4,9%
	Total	66	41,357008	1,7240440	4,2%
Total	T0	44	41,153409	1,6306618	4,0%
	T1	44	40,848011	1,7462791	4,3%
	T2	43	40,811047	2,4794351	6,1%
	Total	131	40,938454	1,9743698	4,8%

Dimension du modèle[a]

		Nombre de niveaux	Nombre de paramètres
Effets fixes	Constante	1	1
	Condition	2	1
	Time	3	2
	Condition * Time	6	2
Résidu			1
Total		12	7

a. Variable dépendante : Heat-sensitive C-fibers Thresholds.

Critères d'information[a]

Log de vraisemblance restreint -2	541,141

Critère d'information d'Akaike (AIC)	543,141
Critère de Hurvich et Tsai (AICC)	543,174
Critère de Bozdogan (CAIC)	546,970
Critère bayésien de Schwartz (BIC)	545,970

Les critères d'informations sont présentés en plus petit, disposant d'un meilleur format.[a]

a. Variable dépendante : Heat-sensitive C-fibers Thresholds.

Effets fixes

Tests des effets fixes de type III[a]

Source	Ddl du numérateur	Ddl du dénominateur	F	Sig.
Constante	1	125	57269,793	,000
Condition	1	125	6,137	,015
Time	2	125	,422	,657
Condition * Time	2	125	,172	,842

a. Variable dépendante : Heat-sensitive C-fibers Thresholds.

Estimations des effets fixes[a]

Paramètre	Estimation	Erreur standard	ddl	t	Sig.
Constante	41,340909	,417323	125	99,062	,000
[Condition=Sham]	-1,084957	,597169	125,000	-1,817	,072
[Condition=taVNS]	0[b]	0	.	.	.
[Time=T0]	,244318	,590185	125,000	,414	,680
[Time=T1]	-,196023	,590185	125	-,332	,740
[Time=T2]	0[b]	0	.	.	.
[Condition=Sham] * [Time=T0]	,221320	,839600	125	,264	,793
[Condition=Sham] * [Time=T1]	,491207	,839600	125	,585	,560

[Condition=Sham] * [Time=T2]	0[b]	0	.	.	.
[Condition=taVNS] * [Time=T0]	0[b]	0	.	.	.
[Condition=taVNS] * [Time=T1]	0[b]	0	.	.	.
[Condition=taVNS] * [Time=T2]	0[b]	0	.	.	.

Estimations des effets fixes[a]

Paramètre	Intervalle de confiance à 95 % Borne inférieure	Borne supérieure
Constante	40,514974	42,166844
[Condition=Sham]	-2,266829	,096915
[Condition=taVNS]	.	.
[Time=T0]	-,923730	1,412367
[Time=T1]	-1,364071	,972026
[Time=T2]	.	.
[Condition=Sham] * [Time=T0]	-1,440353	1,882994
[Condition=Sham] * [Time=T1]	-1,170467	2,152880
[Condition=Sham] * [Time=T2]	.	.
[Condition=taVNS] * [Time=T0]	.	.
[Condition=taVNS] * [Time=T1]	.	.
[Condition=taVNS] * [Time=T2]	.	.

a. Variable dépendante : Heat-sensitive C-fibers Thresholds.

b. Ce paramètre est défini sur 0, car il est redondant.

Matrice de corrélation pour les estimations des effets fixes[a]

Paramètre	Constante	[Condition=Sham]	[Condition=taVNS]	[Time=T0]	[Time=T1]
Constante	1	-,699	.[b]	-,707	-,707
[Condition=Sham]	-,699	1	.[b]	,494	,494
[Condition=taVNS]	.[b]	.[b]	.[b]	.[b]	.[b]
[Time=T0]	-,707	,494	.[b]	1	,500
[Time=T1]	-,707	,494	.[b]	,500	1
[Time=T2]	.[b]	.[b]	.[b]	.[b]	.[b]
[Condition=Sham] * [Time=T0]	,497	-,711	.[b]	-,703	-,351
[Condition=Sham] * [Time=T1]	,497	-,711	.[b]	-,351	-,703

[Condition=Sham] * [Time=T2]	.[b]	.[b]	.[b]	.[b]	.[b]
[Condition=taVNS] * [Time=T0]	.[b]	.[b]	.[b]	.[b]	.[b]
[Condition=taVNS] * [Time=T1]	.[b]	.[b]	.[b]	.[b]	.[b]
[Condition=taVNS] * [Time=T2]	.[b]	.[b]	.[b]	.[b]	.[b]

Matrice de corrélation pour les estimations des effets fixes[a]

Paramètre	[Time=T2]	[Condition=Sham] * [Time=T0]	[Condition=Sham] * [Time=T1]	[Condition=Sham] * [Time=T2]
Constante	.[b]	,497	,497	.[b]
[Condition=Sham]	.[b]	-,711	-,711	.[b]
[Condition=taVNS]	.[b]	.[b]	.[b]	.[b]
[Time=T0]	.[b]	-,703	-,351	.[b]
[Time=T1]	.[b]	-,351	-,703	.[b]
[Time=T2]	.[b]	.[b]	.[b]	.[b]
[Condition=Sham] * [Time=T0]	.[b]	1	,506	.[b]
[Condition=Sham] * [Time=T1]	.[b]	,506	1	.[b]
[Condition=Sham] * [Time=T2]	.[b]	.[b]	.[b]	.[b]
[Condition=taVNS] * [Time=T0]	.[b]	.[b]	.[b]	.[b]
[Condition=taVNS] * [Time=T1]	.[b]	.[b]	.[b]	.[b]
[Condition=taVNS] * [Time=T2]	.[b]	.[b]	.[b]	.[b]

Matrice de corrélation pour les estimations des effets fixes[a]

Paramètre	[Condition=taVNS] * [Time=T0]	[Condition=taVNS] * [Time=T1]	[Condition=taVNS] * [Time=T2]
Constante	.[b]	.[b]	.[b]
[Condition=Sham]	.[b]	.[b]	.[b]
[Condition=taVNS]	.[b]	.[b]	.[b]
[Time=T0]	.[b]	.[b]	.[b]
[Time=T1]	.[b]	.[b]	.[b]
[Time=T2]	.[b]	.[b]	.[b]
[Condition=Sham] * [Time=T0]	.[b]	.[b]	.[b]
[Condition=Sham] * [Time=T1]	.[b]	.[b]	.[b]
[Condition=Sham] * [Time=T2]	.[b]	.[b]	.[b]
[Condition=taVNS] * [Time=T0]	.[b]	.[b]	.[b]
[Condition=taVNS] * [Time=T1]	.[b]	.[b]	.[b]
[Condition=taVNS] * [Time=T2]	.[b]	.[b]	.[b]

a. Variable dépendante : Heat-sensitive C-fibers Thresholds.

b. La corrélation est manquante par défaut, car elle est associée à un paramètre redondant.

Matrice de covariance pour les estimations des effets fixes[a]

Paramètre	Constante	[Condition=Sham]	[Condition=taVNS]	[Time=T0]	[Time=T1]
Constante	,174159	-,174159	0[b]	-,174159	-,174159
[Condition=Sham]	-,174159	,356611	0[b]	,174159	,174159
[Condition=taVNS]	0[b]	0[b]	0[b]	0[b]	0[b]
[Time=T0]	-,174159	,174159	0[b]	,348318	,174159
[Time=T1]	-,174159	,174159	0[b]	,174159	,348318
[Time=T2]	0[b]	0[b]	0[b]	0[b]	0[b]
[Condition=Sham] * [Time=T0]	,174159	-,356611	0[b]	-,348318	-,174159
[Condition=Sham] * [Time=T1]	,174159	-,356611	0[b]	-,174159	-,348318
[Condition=Sham] * [Time=T2]	0[b]	0[b]	0[b]	0[b]	0[b]
[Condition=taVNS] * [Time=T0]	0[b]	0[b]	0[b]	0[b]	0[b]
[Condition=taVNS] * [Time=T1]	0[b]	0[b]	0[b]	0[b]	0[b]
[Condition=taVNS] * [Time=T2]	0[b]	0[b]	0[b]	0[b]	0[b]

Matrice de covariance pour les estimations des effets fixes[a]

Paramètre	[Time=T2]	[Condition=Sham] * [Time=T0]	[Condition=Sham] * [Time=T1]	[Condition=Sham] * [Time=T2]
Constante	0[b]	,174159	,174159	0[b]
[Condition=Sham]	0[b]	-,356611	-,356611	0[b]
[Condition=taVNS]	0[b]	0[b]	0[b]	0[b]
[Time=T0]	0[b]	-,348318	-,174159	0[b]
[Time=T1]	0[b]	-,174159	-,348318	0[b]
[Time=T2]	0[b]	0[b]	0[b]	0[b]
[Condition=Sham] * [Time=T0]	0[b]	,704929	,356611	0[b]
[Condition=Sham] * [Time=T1]	0[b]	,356611	,704929	0[b]
[Condition=Sham] * [Time=T2]	0[b]	0[b]	0[b]	0[b]
[Condition=taVNS] * [Time=T0]	0[b]	0[b]	0[b]	0[b]
[Condition=taVNS] * [Time=T1]	0[b]	0[b]	0[b]	0[b]
[Condition=taVNS] * [Time=T2]	0[b]	0[b]	0[b]	0[b]

Matrice de covariance pour les estimations des effets fixes[a]

Paramètre	[Condition=taVNS] * [Time=T0]	[Condition=taVNS] * [Time=T1]	[Condition=taVNS] * [Time=T2]
Constante	0[b]	0[b]	0[b]
[Condition=Sham]	0[b]	0[b]	0[b]
[Condition=taVNS]	0[b]	0[b]	0[b]
[Time=T0]	0[b]	0[b]	0[b]
[Time=T1]	0[b]	0[b]	0[b]
[Time=T2]	0[b]	0[b]	0[b]
[Condition=Sham] * [Time=T0]	0[b]	0[b]	0[b]
[Condition=Sham] * [Time=T1]	0[b]	0[b]	0[b]
[Condition=Sham] * [Time=T2]	0[b]	0[b]	0[b]
[Condition=taVNS] * [Time=T0]	0[b]	0[b]	0[b]
[Condition=taVNS] * [Time=T1]	0[b]	0[b]	0[b]
[Condition=taVNS] * [Time=T2]	0[b]	0[b]	0[b]

a. Variable dépendante : Heat-sensitive C-fibers Thresholds.

b. La covariance est définie sur 0, car elle est associée à un paramètre redondant.

Paramètres de covariance

Estimations des paramètres de covariance[a]

Paramètre	Estimation	Erreur standard	Z de Wald	Sig.	Intervalle de confiance à 95 %	
					Borne inférieure	Borne supérieure
Résidu	3,831495	,484650	7,906	,000	2,990191	4,909505

a. Variable dépendante : Heat-sensitive C-fibers Thresholds.

Matrice de corrélation pour les estimations des paramètres de covariance[a]

Paramètre	Résidu
Résidu	1

a. Variable dépendante : Heat-sensitive C-fibers Thresholds.

Matrice de covariance pour les estimations des paramètres de covariance[a]

Paramètre	Résidu
Résidu	,234886

a. Variable dépendante : Heat-sensitive C-fibers Thresholds.

Moyenne marginale estimée

1. Condition

Estimations[a]

Condition	Moyenne	Erreur standard	ddl	Intervalle de confiance à 95 % Borne inférieure	Intervalle de confiance à 95 % Borne supérieure
Sham	40,510	,243	125	40,029	40,990
taVNS	41,357	,241	125	40,880	41,834

a. Variable dépendante : Heat-sensitive C-fibers Thresholds.

Comparaisons appariées[a]

(I) Condition	(J) Condition	Différence moyenne (I-J)	Erreur standard	ddl	Sig.[c]
Sham	taVNS	-,847*	,342	125	,015
taVNS	Sham	,847*	,342	125	,015

Comparaisons appariées[a]

(I) Condition	(J) Condition	Intervalle de confiance à 95 % pour la différence[c] Borne inférieure	Intervalle de confiance à 95 % pour la différence[c] Borne supérieure
Sham	taVNS	-1,524	-,170
taVNS	Sham	,170	1,524

Basées sur les moyennes marginales estimées[a]

*. La différence moyenne est significative au niveau ,05.

a. Variable dépendante : Heat-sensitive C-fibers Thresholds.

c. Ajustement pour les comparaisons multiples : Bonferroni.

Tests univariés[a]

Ddl du numérateur	Ddl du dénominateur	F	Sig.
1	125	6,137	,015

Le test de F permet de tester l'effet de Condition. Il s'appuie sur les comparaisons appariées (indépendantes) linéaires parmi les moyennes marginales estimées.[a]

a. Variable dépendante : Heat-sensitive C-fibers Thresholds.

2. Time

Estimations[a]

Time	Moyenne	Erreur standard	ddl	Intervalle de confiance à 95 %	
				Borne inférieure	Borne supérieure
T0	41,153	,295	125	40,569	41,737
T1	40,848	,295	125	40,264	41,432
T2	40,798	,299	125	40,207	41,389

a. Variable dépendante : Heat-sensitive C-fibers Thresholds.

Comparaisons appariées[a]

(I) Time	(J) Time	Différence moyenne (I-J)	Erreur standard	ddl	Sig.[b]	Intervalle de confiance à 95 % pour la différence[b] Borne inférieure
T0	T1	,305	,417	125	1,000	-,707
	T2	,355	,420	125	1,000	-,664
T1	T0	-,305	,417	125	1,000	-1,318
	T2	,050	,420	125	1,000	-,969
T2	T0	-,355	,420	125	1,000	-1,374
	T1	-,050	,420	125	1,000	-1,068

Comparaisons appariées[a]

(I) Time	(J) Time	Intervalle de confiance à 95 % pour la différence Borne supérieure
T0	T1	1,318
	T2	1,374
T1	T0	,707
	T2	1,068
T2	T0	,664

T1	,969

Basées sur les moyennes marginales estimées[a]

a. Variable dépendante : Heat-sensitive C-fibers Thresholds.

b. Ajustement pour les comparaisons multiples : Bonferroni.

Tests univariés[a]

Ddl du numérateur	Ddl du dénominateur	F	Sig.
2	125	,422	,657

Le test de F permet de tester l'effet de Time. Il s'appuie sur les comparaisons appariées (indépendantes) linéaires parmi les moyennes marginales estimées.[a]

a. Variable dépendante : Heat-sensitive C-fibers Thresholds.

3. Condition * Time[a]

Condition	Time	Moyenne	Erreur standard	ddl	Intervalle de confiance à 95 % Borne inférieure	Intervalle de confiance à 95 % Borne supérieure
Sham	T0	40,722	,417	125	39,896	41,548
	T1	40,551	,417	125	39,725	41,377
	T2	40,256	,427	125	39,411	41,101
taVNS	T0	41,585	,417	125	40,759	42,411
	T1	41,145	,417	125	40,319	41,971
	T2	41,341	,417	125	40,515	42,167

a. Variable dépendante : Heat-sensitive C-fibers Thresholds.

1.2.Heat-sensitive Aδ-fibers Thresholds.

```
MIXED HeatsensitiveAδfibersThresholds BY Condition Time
  /CRITERIA=CIN(95) MXITER(100) MXSTEP(10) SCORING(1)
SINGULAR(0.000000000001) HCONVERGE(0,
    ABSOLUTE) LCONVERGE(0, ABSOLUTE) PCONVERGE(0.000001, ABSOLUTE)
  /FIXED=Condition Time Condition*Time | SSTYPE(3)
  /METHOD=REML
  /PRINT=CPS CORB COVB DESCRIPTIVES  SOLUTION TESTCOV
  /EMMEANS=TABLES(Condition) COMPARE ADJ(BONFERRONI)
  /EMMEANS=TABLES(Time) COMPARE ADJ(BONFERRONI)
  /EMMEANS=TABLES(Condition*Time) .
```

Remarques

Sortie obtenue		04-MAY-2021 13:42:18
Commentaires		
Entrée	Jeu de données actif	Jeu_de_données1
	Filtre	<sans>
	Pondération	<sans>
	Fichier scindé	<sans>
	N de lignes dans le fichier de travail	1048509
Gestion des valeurs manquantes	Définition de la valeur manquante	Les valeurs manquantes définies par l'utilisateur sont traitées comme étant manquantes.
	Observations utilisées	Les statistiques sont basées sur toutes les observations comportant des données valides pour toutes les variables du modèle.

Syntaxe		MIXED HeatsensitiveAδfibersThresholds BY Condition Time<br>/CRITERIA=CIN(95) MXITER(100) MXSTEP(10) SCORING(1) SINGULAR(0.000000000001) HCONVERGE(0, ABSOLUTE) LCONVERGE(0, ABSOLUTE) PCONVERGE(0.000001, ABSOLUTE)<br>/FIXED=Condition Time Condition*Time | SSTYPE(3)<br>/METHOD=REML<br>/PRINT=CPS CORB COVB DESCRIPTIVES SOLUTION TESTCOV<br><br>/EMMEANS=TABLES(Condition) COMPARE ADJ(BONFERRONI)<br><br>/EMMEANS=TABLES(Time) COMPARE ADJ(BONFERRONI)<br><br>/EMMEANS=TABLES(Condition*Time) .
Ressources	Temps de processeur	00:00:00,70
	Temps écoulé	00:00:00,69

Récapitulatif de traitement des observations

		Effectif	Pourcentage marginal
Condition	Sham	65	49,6%
	taVNS	66	50,4%
Time	T0	44	33,6%
	T1	44	33,6%
	T2	43	32,8%

Valide	131	100,0%
Exclues	1048378	
Total	1048509	

Statistiques descriptives

Heat-sensitive Aδ-fibers Thresholds

Condition	Time	Effectif	Moyenne	Ecart type	Coefficient de variation
Sham	T0	22	48,304204545454540	2,100731928338373	4,3%
	T1	22	48,399545454545446	2,136651758226112	4,4%
	T2	21	48,441904761904770	2,109302892065572	4,4%
	Total	65	48,380961538461520	2,083206085857530	4,3%
taVNS	T0	22	48,648636363636370	2,408112866075337	5,0%
	T1	22	48,369280272727270	2,048689541301421	4,2%
	T2	22	47,967272727272720	2,111806227172735	4,4%
	Total	66	48,328396454545440	2,179396813291120	4,5%
Total	T0	44	48,476420454545440	2,240009823855073	4,6%
	T1	44	48,384412863636360	2,068707476581019	4,3%
	T2	43	48,199069767441860	2,099080370014097	4,4%
	Total	131	48,354478366412210	2,124163930640162	4,4%

Dimension du modèle[a]

		Nombre de niveaux	Nombre de paramètres
Effets fixes	Constante	1	1
	Condition	2	1
	Time	3	2
	Condition * Time	6	2
Résidu			1

Total	12	7

a. Variable dépendante : Heat-sensitive Aδ-fibers Thresholds.

Critères d'information[a]

Log de vraisemblance restreint -2	565,311
Critère d'information d'Akaike (AIC)	567,311
Critère de Hurvich et Tsai (AICC)	567,343
Critère de Bozdogan (CAIC)	571,139
Critère bayésien de Schwartz (BIC)	570,139

Les critères d'informations sont présentés en plus petit, disposant d'un meilleur format.[a]
a. Variable dépendante : Heat-sensitive Aδ-fibers Thresholds.

Effets fixes

Tests des effets fixes de type III[a]

Source	Ddl du numérateur	Ddl du dénominateur	F	Sig.
Constante	1	125	65869,429	,000
Condition	1	125	,020	,887
Time	2	125	,178	,837
Condition * Time	2	125	,393	,676

a. Variable dépendante : Heat-sensitive Aδ-fibers Thresholds.

Estimations des effets fixes[a]

Paramètre	Estimation	Erreur standard	ddl	t	Sig.
Constante	47,967273	,459684	125	104,348	,000
[Condition=Sham]	,474632	,657786	125,000	,722	,472
[Condition=taVNS]	0[b]	0	.	.	.
[Time=T0]	,681364	,650092	125	1,048	,297

[Time=T1]	,402008	,650092	125	,618	,537
[Time=T2]	0[b]	0	.	.	.
[Condition=Sham] * [Time=T0]	-,819064	,924825	125	-,886	,378
[Condition=Sham] * [Time=T1]	-,444367	,924825	125	-,480	,632
[Condition=Sham] * [Time=T2]	0[b]	0	.	.	.
[Condition=taVNS] * [Time=T0]	0[b]	0	.	.	.
[Condition=taVNS] * [Time=T1]	0[b]	0	.	.	.
[Condition=taVNS] * [Time=T2]	0[b]	0	.	.	.

Estimations des effets fixes[a]

	Intervalle de confiance à 95 %	
Paramètre	Borne inférieure	Borne supérieure
Constante	47,057500	48,877045
[Condition=Sham]	-,827207	1,776471
[Condition=taVNS]	.	.
[Time=T0]	-,605249	1,967976
[Time=T1]	-,884605	1,688620
[Time=T2]	.	.
[Condition=Sham] * [Time=T0]	-2,649407	1,011280
[Condition=Sham] * [Time=T1]	-2,274710	1,385977
[Condition=Sham] * [Time=T2]	.	.
[Condition=taVNS] * [Time=T0]	.	.
[Condition=taVNS] * [Time=T1]	.	.
[Condition=taVNS] * [Time=T2]	.	.

a. Variable dépendante : Heat-sensitive Aδ-fibers Thresholds.

b. Ce paramètre est défini sur 0, car il est redondant.

Matrice de corrélation pour les estimations des effets fixes[a]

Paramètre	Constante	[Condition=Sham]	[Condition=taVNS]	[Time=T0]	[Time=T1]
Constante	1	-,699	.[b]	-,707	-,707
[Condition=Sham]	-,699	1	.[b]	,494	,494
[Condition=taVNS]	.[b]	.[b]	.[b]	.[b]	.[b]
[Time=T0]	-,707	,494	.[b]	1	,500

[Time=T1]	-,707	,494	.[b]	,500	1
[Time=T2]	.[b]	.[b]	.[b]	.[b]	.[b]
[Condition=Sham] * [Time=T0]	,497	-,711	.[b]	-,703	-,351
[Condition=Sham] * [Time=T1]	,497	-,711	.[b]	-,351	-,703
[Condition=Sham] * [Time=T2]	.[b]	.[b]	.[b]	.[b]	.[b]
[Condition=taVNS] * [Time=T0]	.[b]	.[b]	.[b]	.[b]	.[b]
[Condition=taVNS] * [Time=T1]	.[b]	.[b]	.[b]	.[b]	.[b]
[Condition=taVNS] * [Time=T2]	.[b]	.[b]	.[b]	.[b]	.[b]

Matrice de corrélation pour les estimations des effets fixes[a]

Paramètre	[Time=T2]	[Condition=Sham] * [Time=T0]	[Condition=Sham] * [Time=T1]	[Condition=Sham] * [Time=T2]
Constante	.[b]	,497	,497	.[b]
[Condition=Sham]	.[b]	-,711	-,711	.[b]
[Condition=taVNS]	.[b]	.[b]	.[b]	.[b]
[Time=T0]	.[b]	-,703	-,351	.[b]
[Time=T1]	.[b]	-,351	-,703	.[b]
[Time=T2]	.[b]	.[b]	.[b]	.[b]
[Condition=Sham] * [Time=T0]	.[b]	1	,506	.[b]
[Condition=Sham] * [Time=T1]	.[b]	,506	1	.[b]
[Condition=Sham] * [Time=T2]	.[b]	.[b]	.[b]	.[b]
[Condition=taVNS] * [Time=T0]	.[b]	.[b]	.[b]	.[b]
[Condition=taVNS] * [Time=T1]	.[b]	.[b]	.[b]	.[b]
[Condition=taVNS] * [Time=T2]	.[b]	.[b]	.[b]	.[b]

Matrice de corrélation pour les estimations des effets fixes[a]

Paramètre	[Condition=taVNS] * [Time=T0]	[Condition=taVNS] * [Time=T1]	[Condition=taVNS] * [Time=T2]
Constante	.[b]	.[b]	.[b]
[Condition=Sham]	.[b]	.[b]	.[b]
[Condition=taVNS]	.[b]	.[b]	.[b]
[Time=T0]	.[b]	.[b]	.[b]
[Time=T1]	.[b]	.[b]	.[b]
[Time=T2]	.[b]	.[b]	.[b]
[Condition=Sham] * [Time=T0]	.[b]	.[b]	.[b]
[Condition=Sham] * [Time=T1]	.[b]	.[b]	.[b]

[Condition=Sham] * [Time=T2]	.[b]	.[b]	.[b]
[Condition=taVNS] * [Time=T0]	.[b]	.[b]	.[b]
[Condition=taVNS] * [Time=T1]	.[b]	.[b]	.[b]
[Condition=taVNS] * [Time=T2]	.[b]	.[b]	.[b]

a. Variable dépendante : Heat-sensitive Aδ-fibers Thresholds.

b. La corrélation est manquante par défaut, car elle est associée à un paramètre redondant.

Matrice de covariance pour les estimations des effets fixes[a]

Paramètre	Constante	[Condition=Sham]	[Condition=taVNS]	[Time=T0]	[Time=T1]
Constante	,211310	-,211310	0[b]	-,211310	-,211310
[Condition=Sham]	-,211310	,432682	0[b]	,211310	,211310
[Condition=taVNS]	0[b]	0[b]	0[b]	0[b]	0[b]
[Time=T0]	-,211310	,211310	0[b]	,422619	,211310
[Time=T1]	-,211310	,211310	0[b]	,211310	,422619
[Time=T2]	0[b]	0[b]	0[b]	0[b]	0[b]
[Condition=Sham] * [Time=T0]	,211310	-,432682	0[b]	-,422619	-,211310
[Condition=Sham] * [Time=T1]	,211310	-,432682	0[b]	-,211310	-,422619
[Condition=Sham] * [Time=T2]	0[b]	0[b]	0[b]	0[b]	0[b]
[Condition=taVNS] * [Time=T0]	0[b]	0[b]	0[b]	0[b]	0[b]
[Condition=taVNS] * [Time=T1]	0[b]	0[b]	0[b]	0[b]	0[b]
[Condition=taVNS] * [Time=T2]	0[b]	0[b]	0[b]	0[b]	0[b]

Matrice de covariance pour les estimations des effets fixes[a]

Paramètre	[Time=T2]	[Condition=Sham] * [Time=T0]	[Condition=Sham] * [Time=T1]	[Condition=Sham] * [Time=T2]
Constante	0[b]	,211310	,211310	0[b]
[Condition=Sham]	0[b]	-,432682	-,432682	0[b]
[Condition=taVNS]	0[b]	0[b]	0[b]	0[b]
[Time=T0]	0[b]	-,422619	-,211310	0[b]
[Time=T1]	0[b]	-,211310	-,422619	0[b]
[Time=T2]	0[b]	0[b]	0[b]	0[b]
[Condition=Sham] * [Time=T0]	0[b]	,855301	,432682	0[b]
[Condition=Sham] * [Time=T1]	0[b]	,432682	,855301	0[b]

[Condition=Sham] * [Time=T2]	0[b]	0[b]	0[b]	0[b]
[Condition=taVNS] * [Time=T0]	0[b]	0[b]	0[b]	0[b]
[Condition=taVNS] * [Time=T1]	0[b]	0[b]	0[b]	0[b]
[Condition=taVNS] * [Time=T2]	0[b]	0[b]	0[b]	0[b]

Matrice de covariance pour les estimations des effets fixes[a]

Paramètre	[Condition=taVNS] * [Time=T0]	[Condition=taVNS] * [Time=T1]	[Condition=taVNS] * [Time=T2]
Constante	0[b]	0[b]	0[b]
[Condition=Sham]	0[b]	0[b]	0[b]
[Condition=taVNS]	0[b]	0[b]	0[b]
[Time=T0]	0[b]	0[b]	0[b]
[Time=T1]	0[b]	0[b]	0[b]
[Time=T2]	0[b]	0[b]	0[b]
[Condition=Sham] * [Time=T0]	0[b]	0[b]	0[b]
[Condition=Sham] * [Time=T1]	0[b]	0[b]	0[b]
[Condition=Sham] * [Time=T2]	0[b]	0[b]	0[b]
[Condition=taVNS] * [Time=T0]	0[b]	0[b]	0[b]
[Condition=taVNS] * [Time=T1]	0[b]	0[b]	0[b]
[Condition=taVNS] * [Time=T2]	0[b]	0[b]	0[b]

a. Variable dépendante : Heat-sensitive Aδ-fibers Thresholds.

b. La covariance est définie sur 0, car elle est associée à un paramètre redondant.

Paramètres de covariance

Estimations des paramètres de covariance[a]

Paramètre	Estimation	Erreur standard	Z de Wald	Sig.	Intervalle de confiance à 95 %	
					Borne inférieure	Borne supérieure
Résidu	4,648814	,588034	7,906	,000	3,628046	5,956780

a. Variable dépendante : Heat-sensitive Aδ-fibers Thresholds.

Matrice de corrélation pour les estimations des paramètres de covariance[a]

Paramètre	Résidu
Résidu	1

a. Variable dépendante : Heat-sensitive Aδ-fibers Thresholds.

Matrice de covariance pour les estimations des paramètres de covariance[a]

Paramètre	Résidu
Résidu	,345784

a. Variable dépendante : Heat-sensitive Aδ-fibers Thresholds.

Moyenne marginale estimée

1. Condition

Estimations[a]

Condition	Moyenne	Erreur standard	ddl	Intervalle de confiance à 95 %	
				Borne inférieure	Borne supérieure
Sham	48,382	,267	125	47,852	48,911
taVNS	48,328	,265	125	47,803	48,854

a. Variable dépendante : Heat-sensitive Aδ-fibers Thresholds.

Comparaisons appariées[a]

(I) Condition	(J) Condition	Différence moyenne (I-J)	Erreur standard	ddl	Sig.[b]
Sham	taVNS	,053	,377	125	,887
taVNS	Sham	-,053	,377	125	,887

Comparaisons appariées[a]

(I) Condition	(J) Condition	Intervalle de confiance à 95 % pour la différence[b]	
		Borne inférieure	Borne supérieure
Sham	taVNS	-,692	,799
taVNS	Sham	-,799	,692

Basées sur les moyennes marginales estimées[a]

a. Variable dépendante : Heat-sensitive Aδ-fibers Thresholds.

b. Ajustement pour les comparaisons multiples : Bonferroni.

Tests univariés[a]

Ddl du numérateur	Ddl du dénominateur	F	Sig.
1	125	,020	,887

Le test de F permet de tester l'effet de Condition. Il s'appuie sur les comparaisons appariées (indépendantes) linéaires parmi les moyennes marginales estimées.[a]

a. Variable dépendante : Heat-sensitive Aδ-fibers Thresholds.

2. Time

Estimations[a]

Time	Moyenne	Erreur standard	ddl	Intervalle de confiance à 95 %	
				Borne inférieure	Borne supérieure
T0	48,476	,325	125	47,833	49,120
T1	48,384	,325	125	47,741	49,028
T2	48,205	,329	125	47,554	48,856

a. Variable dépendante : Heat-sensitive Aδ-fibers Thresholds.

Comparaisons appariées[a]

(I) Time	(J) Time	Différence moyenne (I-J)	Erreur standard	ddl	Sig.[b]	Intervalle de confiance à 95 % pour la différence[b]
						Borne inférieure
T0	T1	,092	,460	125	1,000	-1,023
	T2	,272	,462	125	1,000	-,850
T1	T0	-,092	,460	125	1,000	-1,207
	T2	,180	,462	125	1,000	-,942
T2	T0	-,272	,462	125	1,000	-1,394
	T1	-,180	,462	125	1,000	-1,302

Comparaisons appariées[a]

(I) Time	(J) Time	Intervalle de confiance à 95 % pour la différence
		Borne supérieure
T0	T1	1,207
	T2	1,394

T1	T0	1,023
	T2	1,302
T2	T0	,850
	T1	,942

Basées sur les moyennes marginales estimées[a]

a. Variable dépendante : Heat-sensitive Aδ-fibers Thresholds.

b. Ajustement pour les comparaisons multiples : Bonferroni.

Tests univariés[a]

Ddl du numérateur	Ddl du dénominateur	F	Sig.
2	125,000	,178	,837

Le test de F permet de tester l'effet de Time. Il s'appuie sur les comparaisons appariées (indépendantes) linéaires parmi les moyennes marginales estimées.[a]

a. Variable dépendante : Heat-sensitive Aδ-fibers Thresholds.

3. Condition * Time[a]

Condition	Time	Moyenne	Erreur standard	ddl	Intervalle de confiance à 95 % Borne inférieure	Intervalle de confiance à 95 % Borne supérieure
Sham	T0	48,304	,460	125	47,394	49,214
	T1	48,400	,460	125	47,490	49,309
	T2	48,442	,471	125	47,511	49,373
taVNS	T0	48,649	,460	125	47,739	49,558
	T1	48,369	,460	125	47,460	49,279
	T2	47,967	,460	125	47,058	48,877

a. Variable dépendante : Heat-sensitive Aδ-fibers Thresholds.

1.3. Mechanosensitive Aβ-fibers Detection Thresholds.

```
MIXED MechanosensitiveAβfibersThresholds BY Condition Time
  /CRITERIA=CIN(95) MXITER(100) MXSTEP(10) SCORING(1)
SINGULAR(0.000000000001) HCONVERGE(0,
    ABSOLUTE) LCONVERGE(0, ABSOLUTE) PCONVERGE(0.000001, ABSOLUTE)
  /FIXED=Condition Time Condition*Time | SSTYPE(3)
  /METHOD=REML
  /PRINT=CPS CORB COVB DESCRIPTIVES  SOLUTION TESTCOV
  /EMMEANS=TABLES(Condition) COMPARE ADJ(BONFERRONI)
  /EMMEANS=TABLES(Time) COMPARE ADJ(BONFERRONI)
  /EMMEANS=TABLES(Condition*Time) .
```

Remarques

Sortie obtenue		04-MAY-2021 13:43:33
Commentaires		
Entrée	Jeu de données actif	Jeu_de_données1
	Filtre	<sans>
	Pondération	<sans>
	Fichier scindé	<sans>
	N de lignes dans le fichier de travail	1048509
Gestion des valeurs manquantes	Définition de la valeur manquante	Les valeurs manquantes définies par l'utilisateur sont traitées comme étant manquantes.
	Observations utilisées	Les statistiques sont basées sur toutes les observations comportant des données valides pour toutes les variables du modèle.

Syntaxe		MIXED MechanosensitiveAβfibersThresholds BY Condition Time /CRITERIA=CIN(95) MXITER(100) MXSTEP(10) SCORING(1) SINGULAR(0.000000000001) HCONVERGE(0, ABSOLUTE) LCONVERGE(0, ABSOLUTE) PCONVERGE(0.000001, ABSOLUTE) /FIXED=Condition Time Condition*Time | SSTYPE(3) /METHOD=REML /PRINT=CPS CORB COVB DESCRIPTIVES SOLUTION TESTCOV /EMMEANS=TABLES(Condition) COMPARE ADJ(BONFERRONI) /EMMEANS=TABLES(Time) COMPARE ADJ(BONFERRONI) /EMMEANS=TABLES(Condition*Time) .
Ressources	Temps de processeur	00:00:00,67
	Temps écoulé	00:00:00,66

Récapitulatif de traitement des observations

		Effectif	Pourcentage marginal
Condition	Sham	65	49,6%
	taVNS	66	50,4%
Time	T0	44	33,6%
	T1	44	33,6%

	T2	43	32,8%
Valide		131	100,0%
Exclues		1048378	
Total		1048509	

Statistiques descriptives

Mechanosensitive Aβ-fibers Thresholds

Condition	Time	Effectif	Moyenne	Ecart type	Coefficient de variation
Sham	T0	22	,0035545455	,00288293043	81,1%
	T1	22	,0054226991	,00577672646	106,5%
	T2	21	,0054088095	,00533791905	98,7%
	Total	65	,0047859135	,00483414509	101,0%
taVNS	T0	22	,0036297727	,00343257868	94,6%
	T1	22	,0061795455	,00654135669	105,9%
	T2	22	,0072986364	,00868962123	119,1%
	Total	66	,0057026515	,00666481226	116,9%
Total	T0	44	,0035921591	,00313284854	87,2%
	T1	44	,0058011223	,00611072107	105,3%
	T2	43	,0063756977	,00722749336	113,4%
	Total	131	,0052477815	,00582462749	111,0%

Dimension du modèle[a]

		Nombre de niveaux	Nombre de paramètres
Effets fixes	Constante	1	1
	Condition	2	1
	Time	3	2
	Condition * Time	6	2
Résidu			1
Total		12	7

a. Variable dépendante : Mechanosensitive Aβ-fibers Thresholds.

Critères d'information[a]

Log de vraisemblance restreint -2	-915,078
Critère d'information d'Akaike (AIC)	-913,078

Critère de Hurvich et Tsai (AICC)	-913,046
Critère de Bozdogan (CAIC)	-909,250
Critère bayésien de Schwartz (BIC)	-910,250

Les critères d'informations sont présentés en plus petit, disposant d'un meilleur format.[a]

a. Variable dépendante : Mechanosensitive Aβ-fibers Thresholds.

Effets fixes

Tests des effets fixes de type III[a]

Source	Ddl du numérateur	Ddl du dénominateur	F	Sig.
Constante	1	125	107,982	,000
Condition	1	125	,807	,371
Time	2	125	2,793	,065
Condition * Time	2	125,000	,273	,762

a. Variable dépendante : Mechanosensitive Aβ-fibers Thresholds.

Estimations des effets fixes[a]

Paramètre	Estimation	Erreur standard	ddl	t	Sig.
Constante	,007299	,001232	125	5,922	,000
[Condition=Sham]	-,001890	,001764	125	-1,072	,286
[Condition=taVNS]	0[b]	0	.	.	.
[Time=T0]	-,003669	,001743	125	-2,105	,037
[Time=T1]	-,001119	,001743	125	-,642	,522
[Time=T2]	0[b]	0	.	.	.
[Condition=Sham] * [Time=T0]	,001815	,002479	125	,732	,466
[Condition=Sham] * [Time=T1]	,001133	,002479	125	,457	,649
[Condition=Sham] * [Time=T2]	0[b]	0	.	.	.

[Condition=taVNS] * [Time=T0]	0[b]	0	.	.	.
[Condition=taVNS] * [Time=T1]	0[b]	0	.	.	.
[Condition=taVNS] * [Time=T2]	0[b]	0	.	.	.

Estimations des effets fixes[a]

Paramètre	Intervalle de confiance à 95 % Borne inférieure	Borne supérieure
Constante	,004860	,009738
[Condition=Sham]	-,005380	,001600
[Condition=taVNS]	.	.
[Time=T0]	-,007118	-,000219
[Time=T1]	-,004569	,002330
[Time=T2]	.	.
[Condition=Sham] * [Time=T0]	-,003093	,006722
[Condition=Sham] * [Time=T1]	-,003774	,006040
[Condition=Sham] * [Time=T2]	.	.
[Condition=taVNS] * [Time=T0]	.	.
[Condition=taVNS] * [Time=T1]	.	.
[Condition=taVNS] * [Time=T2]	.	.

a. Variable dépendante : Mechanosensitive Aβ-fibers Thresholds.

b. Ce paramètre est défini sur 0, car il est redondant.

Matrice de corrélation pour les estimations des effets fixes[a]

Paramètre	Constante	[Condition=Sham]	[Condition=taVNS]	[Time=T0]	[Time=T1]
Constante	1	-,699	.[b]	-,707	-,707
[Condition=Sham]	-,699	1	.[b]	,494	,494
[Condition=taVNS]	.[b]	.[b]	.[b]	.[b]	.[b]
[Time=T0]	-,707	,494	.[b]	1	,500
[Time=T1]	-,707	,494	.[b]	,500	1
[Time=T2]	.[b]	.[b]	.[b]	.[b]	.[b]
[Condition=Sham] * [Time=T0]	,497	-,711	.[b]	-,703	-,351
[Condition=Sham] * [Time=T1]	,497	-,711	.[b]	-,351	-,703
[Condition=Sham] * [Time=T2]	.[b]	.[b]	.[b]	.[b]	.[b]

[Condition=taVNS] * [Time=T0]	.[b]	.[b]	.[b]	.[b]	.[b]
[Condition=taVNS] * [Time=T1]	.[b]	.[b]	.[b]	.[b]	.[b]
[Condition=taVNS] * [Time=T2]	.[b]	.[b]	.[b]	.[b]	.[b]

Matrice de corrélation pour les estimations des effets fixes[a]

Paramètre	[Time=T2]	[Condition=Sham] * [Time=T0]	[Condition=Sham] * [Time=T1]	[Condition=Sham] * [Time=T2]
Constante	.[b]	,497	,497	.[b]
[Condition=Sham]	.[b]	-,711	-,711	.[b]
[Condition=taVNS]	.[b]	.[b]	.[b]	.[b]
[Time=T0]	.[b]	-,703	-,351	.[b]
[Time=T1]	.[b]	-,351	-,703	.[b]
[Time=T2]	.[b]	.[b]	.[b]	.[b]
[Condition=Sham] * [Time=T0]	.[b]	1	,506	.[b]
[Condition=Sham] * [Time=T1]	.[b]	,506	1	.[b]
[Condition=Sham] * [Time=T2]	.[b]	.[b]	.[b]	.[b]
[Condition=taVNS] * [Time=T0]	.[b]	.[b]	.[b]	.[b]
[Condition=taVNS] * [Time=T1]	.[b]	.[b]	.[b]	.[b]
[Condition=taVNS] * [Time=T2]	.[b]	.[b]	.[b]	.[b]

Matrice de corrélation pour les estimations des effets fixes[a]

Paramètre	[Condition=taVNS] * [Time=T0]	[Condition=taVNS] * [Time=T1]	[Condition=taVNS] * [Time=T2]
Constante	.[b]	.[b]	.[b]
[Condition=Sham]	.[b]	.[b]	.[b]
[Condition=taVNS]	.[b]	.[b]	.[b]
[Time=T0]	.[b]	.[b]	.[b]
[Time=T1]	.[b]	.[b]	.[b]
[Time=T2]	.[b]	.[b]	.[b]
[Condition=Sham] * [Time=T0]	.[b]	.[b]	.[b]
[Condition=Sham] * [Time=T1]	.[b]	.[b]	.[b]
[Condition=Sham] * [Time=T2]	.[b]	.[b]	.[b]
[Condition=taVNS] * [Time=T0]	.[b]	.[b]	.[b]
[Condition=taVNS] * [Time=T1]	.[b]	.[b]	.[b]
[Condition=taVNS] * [Time=T2]	.[b]	.[b]	.[b]

a. Variable dépendante : Mechanosensitive Aβ-fibers Thresholds.

b. La corrélation est manquante par défaut, car elle est associée à un paramètre redondant.

Matrice de covariance pour les estimations des effets fixes[a]

Paramètre	Constante	[Condition=Sham]	[Condition=taVNS]	[Time=T0]
Constante	1,518872E-6	-1,518872E-6	0[b]	-1,518872E-6
[Condition=Sham]	-1,518872E-6	3,110071E-6	0[b]	1,518872E-6
[Condition=taVNS]	0[b]	0[b]	0[b]	0[b]
[Time=T0]	-1,518872E-6	1,518872E-6	0[b]	3,037743E-6
[Time=T1]	-1,518872E-6	1,518872E-6	0[b]	1,518872E-6
[Time=T2]	0[b]	0[b]	0[b]	0[b]
[Condition=Sham] * [Time=T0]	1,518872E-6	-3,110071E-6	0[b]	-3,037743E-6
[Condition=Sham] * [Time=T1]	1,518872E-6	-3,110071E-6	0[b]	-1,518872E-6
[Condition=Sham] * [Time=T2]	0[b]	0[b]	0[b]	0[b]
[Condition=taVNS] * [Time=T0]	0[b]	0[b]	0[b]	0[b]
[Condition=taVNS] * [Time=T1]	0[b]	0[b]	0[b]	0[b]
[Condition=taVNS] * [Time=T2]	0[b]	0[b]	0[b]	0[b]

Matrice de covariance pour les estimations des effets fixes[a]

Paramètre	[Time=T1]	[Time=T2]	[Condition=Sham] * [Time=T0]	[Condition=Sham] * [Time=T1]
Constante	-1,518872E-6	0[b]	1,518872E-6	1,518872E-6
[Condition=Sham]	1,518872E-6	0[b]	-3,110071E-6	-3,110071E-6
[Condition=taVNS]	0[b]	0[b]	0[b]	0[b]
[Time=T0]	1,518872E-6	0[b]	-3,037743E-6	-1,518872E-6
[Time=T1]	3,037743E-6	0[b]	-1,518872E-6	-3,037743E-6
[Time=T2]	0[b]	0[b]	0[b]	0[b]
[Condition=Sham] * [Time=T0]	-1,518872E-6	0[b]	6,147814E-6	3,110071E-6
[Condition=Sham] * [Time=T1]	-3,037743E-6	0[b]	3,110071E-6	6,147814E-6
[Condition=Sham] * [Time=T2]	0[b]	0[b]	0[b]	0[b]
[Condition=taVNS] * [Time=T0]	0[b]	0[b]	0[b]	0[b]
[Condition=taVNS] * [Time=T1]	0[b]	0[b]	0[b]	0[b]
[Condition=taVNS] * [Time=T2]	0[b]	0[b]	0[b]	0[b]

Matrice de covariance pour les estimations des effets fixes[a]

Paramètre	[Condition=Sham] * [Time=T2]	[Condition=taVNS] * [Time=T0]	[Condition=taVNS] * [Time=T1]	[Condition=taVNS] * [Time=T2]

Constante	0[b]	0[b]	0[b]	0[b]
[Condition=Sham]	0[b]	0[b]	0[b]	0[b]
[Condition=taVNS]	0[b]	0[b]	0[b]	0[b]
[Time=T0]	0[b]	0[b]	0[b]	0[b]
[Time=T1]	0[b]	0[b]	0[b]	0[b]
[Time=T2]	0[b]	0[b]	0[b]	0[b]
[Condition=Sham] * [Time=T0]	0[b]	0[b]	0[b]	0[b]
[Condition=Sham] * [Time=T1]	0[b]	0[b]	0[b]	0[b]
[Condition=Sham] * [Time=T2]	0[b]	0[b]	0[b]	0[b]
[Condition=taVNS] * [Time=T0]	0[b]	0[b]	0[b]	0[b]
[Condition=taVNS] * [Time=T1]	0[b]	0[b]	0[b]	0[b]
[Condition=taVNS] * [Time=T2]	0[b]	0[b]	0[b]	0[b]

a. Variable dépendante : Mechanosensitive Aβ-fibers Thresholds.

b. La covariance est définie sur 0, car elle est associée à un paramètre redondant.

Paramètres de covariance

Estimations des paramètres de covariance[a]

Paramètre	Estimation	Erreur standard	Z de Wald	Sig.	Intervalle de confiance à 95 % Borne inférieure	Intervalle de confiance à 95 % Borne supérieure
Résidu	3,341518E-5	4,226723E-6	7,906	,000	2,607800E-5	4,281670E-5

a. Variable dépendante : Mechanosensitive Aβ-fibers Thresholds.

Matrice de corrélation pour les estimations des paramètres de covariance[a]

Paramètre	Résidu
Résidu	1

a. Variable dépendante : Mechanosensitive Aβ-fibers Thresholds.

Matrice de covariance pour les estimations des paramètres de covariance[a]

Paramètre	Résidu
Résidu	1,786519E-11

a. Variable dépendante : Mechanosensitive Aβ-fibers Thresholds.

Moyenne marginale estimée

1. Condition

Estimations[a]

Condition	Moyenne	Erreur standard	ddl	Intervalle de confiance à 95 %	
				Borne inférieure	Borne supérieure
Sham	,005	,001	125	,003	,006
taVNS	,006	,001	125	,004	,007

a. Variable dépendante : Mechanosensitive Aβ-fibers Thresholds.

Comparaisons appariées[a]

(I) Condition	(J) Condition	Différence moyenne (I-J)	Erreur standard	ddl	Sig.[b]
Sham	taVNS	-,001	,001	125	,371
taVNS	Sham	,001	,001	125	,371

Comparaisons appariées[a]

(I) Condition	(J) Condition	Intervalle de confiance à 95 % pour la différence[b]	
		Borne inférieure	Borne supérieure
Sham	taVNS	-,003	,001
taVNS	Sham	-,001	,003

Basées sur les moyennes marginales estimées[a]

a. Variable dépendante : Mechanosensitive Aβ-fibers Thresholds.

b. Ajustement pour les comparaisons multiples : Bonferroni.

Tests univariés[a]

Ddl du numérateur	Ddl du dénominateur	F	Sig.

1	125	,807	,371

Le test de F permet de tester l'effet de Condition. Il s'appuie sur les comparaisons appariées (indépendantes) linéaires parmi les moyennes marginales estimées.[a]

a. Variable dépendante : Mechanosensitive Aβ-fibers Thresholds.

2. Time

Estimations[a]

Time	Moyenne	Erreur standard	ddl	Intervalle de confiance à 95 % Borne inférieure	Intervalle de confiance à 95 % Borne supérieure
T0	,004	,001	125	,002	,005
T1	,006	,001	125	,004	,008
T2	,006	,001	125	,005	,008

a. Variable dépendante : Mechanosensitive Aβ-fibers Thresholds.

Comparaisons appariées[a]

(I) Time	(J) Time	Différence moyenne (I-J)	Erreur standard	ddl	Sig.[b]	Intervalle de confiance à 95 % pour la différence[b] Borne inférieure
T0	T1	-,002	,001	125	,226	-,005
	T2	-,003	,001	125	,083	-,006
T1	T0	,002	,001	125	,226	-,001
	T2	-,001	,001	125	1,000	-,004
T2	T0	,003	,001	125	,083	,000
	T1	,001	,001	125	1,000	-,002

Comparaisons appariées[a]

(I) Time	(J) Time	Intervalle de confiance à 95 % pour la différence Borne supérieure
T0	T1	,001
	T2	,000
T1	T0	,005
	T2	,002
T2	T0	,006
	T1	,004

Basées sur les moyennes marginales estimées[a]

a. Variable dépendante : Mechanosensitive Aβ-fibers Thresholds.

b. Ajustement pour les comparaisons multiples : Bonferroni.

Tests univariés[a]

Ddl du numérateur	Ddl du dénominateur	F	Sig.
2	125	2,793	,065

Le test de F permet de tester l'effet de Time. Il s'appuie sur les comparaisons appariées (indépendantes) linéaires parmi les moyennes marginales estimées.[a]

a. Variable dépendante : Mechanosensitive Aβ-fibers Thresholds.

3. Condition * Time[a]

					Intervalle de confiance à 95 %	
Condition	Time	Moyenne	Erreur standard	ddl	Borne inférieure	Borne supérieure
Sham	T0	,004	,001	125	,001	,006
	T1	,005	,001	125	,003	,008
	T2	,005	,001	125	,003	,008
taVNS	T0	,004	,001	125	,001	,006
	T1	,006	,001	125	,004	,009
	T2	,007	,001	125	,005	,010

a. Variable dépendante : Mechanosensitive Aβ-fibers Thresholds.

1.4.Cool-sensitive Aδ-fibers Detection Thresholds.

```
MIXED CoolsensitiveAδfibersThresholds BY Condition Time
  /CRITERIA=CIN(95) MXITER(100) MXSTEP(10) SCORING(1)
SINGULAR(0.000000000001) HCONVERGE(0,
    ABSOLUTE) LCONVERGE(0, ABSOLUTE) PCONVERGE(0.000001, ABSOLUTE)
  /FIXED=Condition Time Condition*Time | SSTYPE(3)
  /METHOD=REML
  /PRINT=CPS CORB COVB DESCRIPTIVES  SOLUTION TESTCOV
  /EMMEANS=TABLES(Condition) COMPARE ADJ(BONFERRONI)
  /EMMEANS=TABLES(Time) COMPARE ADJ(BONFERRONI)
  /EMMEANS=TABLES(Condition*Time) .
```

Remarques

Sortie obtenue		04-MAY-2021 13:44:27
Commentaires		
Entrée	Jeu de données actif	Jeu_de_données1
	Filtre	<sans>
	Pondération	<sans>
	Fichier scindé	<sans>
	N de lignes dans le fichier de travail	1048509
Gestion des valeurs manquantes	Définition de la valeur manquante	Les valeurs manquantes définies par l'utilisateur sont traitées comme étant manquantes.
	Observations utilisées	Les statistiques sont basées sur toutes les observations comportant des données valides pour toutes les variables du modèle.

Syntaxe		MIXED CoolsensitiveAδfibersThresholds BY Condition Time<br>/CRITERIA=CIN(95) MXITER(100) MXSTEP(10) SCORING(1) SINGULAR(0.000000000001) HCONVERGE(0, ABSOLUTE) LCONVERGE(0, ABSOLUTE) PCONVERGE(0.000001, ABSOLUTE)<br>/FIXED=Condition Time Condition*Time | SSTYPE(3)<br>/METHOD=REML<br>/PRINT=CPS CORB COVB DESCRIPTIVES SOLUTION TESTCOV<br>/EMMEANS=TABLES(Condition) COMPARE ADJ(BONFERRONI)<br>/EMMEANS=TABLES(Time) COMPARE ADJ(BONFERRONI)<br>/EMMEANS=TABLES(Condition*Time) .
Ressources	Temps de processeur	00:00:00,69
	Temps écoulé	00:00:00,67

Récapitulatif de traitement des observations

		Effectif	Pourcentage marginal
Condition	Sham	65	49,6%
	taVNS	66	50,4%
Time	T0	44	33,6%
	T1	44	33,6%

	T2	43	32,8%
Valide		131	100,0%
Exclues		1048378	
Total		1048509	

Statistiques descriptives

Cool-sensitive Aδ-fibers Thresholds

Condition	Time	Effectif	Moyenne	Ecart type	Coefficient de variation
Sham	T0	22	29,610795454545450	,735710049949979	2,5%
	T1	22	28,807897727272724	1,088298925636797	3,8%
	T2	21	28,815476190476193	1,014634361188681	3,5%
	Total	65	29,082096153846150	1,016465832412256	3,5%
taVNS	T0	22	29,738522727272724	1,041831508602047	3,5%
	T1	22	28,910681818181820	1,229667294776989	4,3%
	T2	22	28,925227272727273	1,138451828113541	3,9%
	Total	66	29,191477272727287	1,187387401728359	4,1%
Total	T0	44	29,674659090909092	,893643995325067	3,0%
	T1	44	28,859289772727262	1,148732472527646	4,0%
	T2	43	28,871627906976748	1,068338703052901	3,7%
	Total	131	29,137204198473288	1,103001355917261	3,8%

Dimension du modèle[a]

		Nombre de niveaux	Nombre de paramètres
Effets fixes	Constante	1	1
	Condition	2	1

	Time	3	2
	Condition * Time	6	2
Résidu			1
Total		12	7

a. Variable dépendante : Cool-sensitive Aδ-fibers Thresholds.

Critères d'information[a]

Log de vraisemblance restreint -2	386,138
Critère d'information d'Akaike (AIC)	388,138
Critère de Hurvich et Tsai (AICC)	388,171
Critère de Bozdogan (CAIC)	391,967
Critère bayésien de Schwartz (BIC)	390,967

Les critères d'informations sont présentés en plus petit, disposant d'un meilleur format.[a]

a. Variable dépendante : Cool-sensitive Aδ-fibers Thresholds.

Effets fixes

Tests des effets fixes de type III[a]

Source	Ddl du numérateur	Ddl du dénominateur	F	Sig.
Constante	1	125	100260,756	,000
Condition	1	125	,380	,539
Time	2	125	8,644	,000
Condition * Time	2	125	,002	,998

a. Variable dépendante : Cool-sensitive Aδ-fibers Thresholds.

Estimations des effets fixes[a]

Paramètre	Estimation	Erreur standard	ddl	t	Sig.
Constante	28,925227	,224494	125	128,846	,000

[Condition=Sham]	-,109751	,321240	125,000	-,342	,733
[Condition=taVNS]	0[b]	0	.	.	.
[Time=T0]	,813295	,317483	125,000	2,562	,012
[Time=T1]	-,014545	,317483	125	-,046	,964
[Time=T2]	0[b]	0	.	.	.
[Condition=Sham] * [Time=T0]	-,017976	,451653	125	-,040	,968
[Condition=Sham] * [Time=T1]	,006967	,451653	125	,015	,988
[Condition=Sham] * [Time=T2]	0[b]	0	.	.	.
[Condition=taVNS] * [Time=T0]	0[b]	0	.	.	.
[Condition=taVNS] * [Time=T1]	0[b]	0	.	.	.
[Condition=taVNS] * [Time=T2]	0[b]	0	.	.	.

Estimations des effets fixes[a]

	Intervalle de confiance à 95 %	
Paramètre	Borne inférieure	Borne supérieure
Constante	28,480925	29,369529
[Condition=Sham]	-,745525	,526023
[Condition=taVNS]	.	.
[Time=T0]	,184958	1,441633
[Time=T1]	-,642883	,613792
[Time=T2]	.	.
[Condition=Sham] * [Time=T0]	-,911854	,875901
[Condition=Sham] * [Time=T1]	-,886910	,900844
[Condition=Sham] * [Time=T2]	.	.
[Condition=taVNS] * [Time=T0]	.	.
[Condition=taVNS] * [Time=T1]	.	.
[Condition=taVNS] * [Time=T2]	.	.

a. Variable dépendante : Cool-sensitive Aδ-fibers Thresholds.

b. Ce paramètre est défini sur 0, car il est redondant.

Matrice de corrélation pour les estimations des effets fixes[a]

Paramètre	Constante	[Condition=Sham]	[Condition=taVNS]	[Time=T0]	[Time=T1]
Constante	1	-,699	.[b]	-,707	-,707

[Condition=Sham]	-,699	1	.[b]	,494	,494
[Condition=taVNS]	.[b]	.[b]	.[b]	.[b]	.[b]
[Time=T0]	-,707	,494	.[b]	1	,500
[Time=T1]	-,707	,494	.[b]	,500	1
[Time=T2]	.[b]	.[b]	.[b]	.[b]	.[b]
[Condition=Sham] * [Time=T0]	,497	-,711	.[b]	-,703	-,351
[Condition=Sham] * [Time=T1]	,497	-,711	.[b]	-,351	-,703
[Condition=Sham] * [Time=T2]	.[b]	.[b]	.[b]	.[b]	.[b]
[Condition=taVNS] * [Time=T0]	.[b]	.[b]	.[b]	.[b]	.[b]
[Condition=taVNS] * [Time=T1]	.[b]	.[b]	.[b]	.[b]	.[b]
[Condition=taVNS] * [Time=T2]	.[b]	.[b]	.[b]	.[b]	.[b]

Matrice de corrélation pour les estimations des effets fixes[a]

Paramètre	[Time=T2]	[Condition=Sham] * [Time=T0]	[Condition=Sham] * [Time=T1]	[Condition=Sham] * [Time=T2]
Constante	.[b]	,497	,497	.[b]
[Condition=Sham]	.[b]	-,711	-,711	.[b]
[Condition=taVNS]	.[b]	.[b]	.[b]	.[b]
[Time=T0]	.[b]	-,703	-,351	.[b]
[Time=T1]	.[b]	-,351	-,703	.[b]
[Time=T2]	.[b]	.[b]	.[b]	.[b]
[Condition=Sham] * [Time=T0]	.[b]	1	,506	.[b]
[Condition=Sham] * [Time=T1]	.[b]	,506	1	.[b]
[Condition=Sham] * [Time=T2]	.[b]	.[b]	.[b]	.[b]
[Condition=taVNS] * [Time=T0]	.[b]	.[b]	.[b]	.[b]
[Condition=taVNS] * [Time=T1]	.[b]	.[b]	.[b]	.[b]
[Condition=taVNS] * [Time=T2]	.[b]	.[b]	.[b]	.[b]

Matrice de corrélation pour les estimations des effets fixes[a]

Paramètre	[Condition=taVNS] * [Time=T0]	[Condition=taVNS] * [Time=T1]	[Condition=taVNS] * [Time=T2]
Constante	.[b]	.[b]	.[b]
[Condition=Sham]	.[b]	.[b]	.[b]
[Condition=taVNS]	.[b]	.[b]	.[b]
[Time=T0]	.[b]	.[b]	.[b]
[Time=T1]	.[b]	.[b]	.[b]

[Time=T2]	.[b]	.[b]	.[b]
[Condition=Sham] * [Time=T0]	.[b]	.[b]	.[b]
[Condition=Sham] * [Time=T1]	.[b]	.[b]	.[b]
[Condition=Sham] * [Time=T2]	.[b]	.[b]	.[b]
[Condition=taVNS] * [Time=T0]	.[b]	.[b]	.[b]
[Condition=taVNS] * [Time=T1]	.[b]	.[b]	.[b]
[Condition=taVNS] * [Time=T2]	.[b]	.[b]	.[b]

a. Variable dépendante : Cool-sensitive Aδ-fibers Thresholds.

b. La corrélation est manquante par défaut, car elle est associée à un paramètre redondant.

Matrice de covariance pour les estimations des effets fixes[a]

Paramètre	Constante	[Condition=Sham]	[Condition=taVNS]	[Time=T0]	[Time=T1]
Constante	,050398	-,050398	0[b]	-,050398	-,050398
[Condition=Sham]	-,050398	,103195	0[b]	,050398	,050398
[Condition=taVNS]	0[b]	0[b]	0[b]	0[b]	0[b]
[Time=T0]	-,050398	,050398	0[b]	,100795	,050398
[Time=T1]	-,050398	,050398	0[b]	,050398	,100795
[Time=T2]	0[b]	0[b]	0[b]	0[b]	0[b]
[Condition=Sham] * [Time=T0]	,050398	-,103195	0[b]	-,100795	-,050398
[Condition=Sham] * [Time=T1]	,050398	-,103195	0[b]	-,050398	-,100795
[Condition=Sham] * [Time=T2]	0[b]	0[b]	0[b]	0[b]	0[b]
[Condition=taVNS] * [Time=T0]	0[b]	0[b]	0[b]	0[b]	0[b]
[Condition=taVNS] * [Time=T1]	0[b]	0[b]	0[b]	0[b]	0[b]
[Condition=taVNS] * [Time=T2]	0[b]	0[b]	0[b]	0[b]	0[b]

Matrice de covariance pour les estimations des effets fixes[a]

Paramètre	[Time=T2]	[Condition=Sham] * [Time=T0]	[Condition=Sham] * [Time=T1]	[Condition=Sham] * [Time=T2]
Constante	0[b]	,050398	,050398	0[b]
[Condition=Sham]	0[b]	-,103195	-,103195	0[b]
[Condition=taVNS]	0[b]	0[b]	0[b]	0[b]
[Time=T0]	0[b]	-,100795	-,050398	0[b]
[Time=T1]	0[b]	-,050398	-,100795	0[b]
[Time=T2]	0[b]	0[b]	0[b]	0[b]

[Condition=Sham] * [Time=T0]	0[b]	,203990	,103195	0[b]
[Condition=Sham] * [Time=T1]	0[b]	,103195	,203990	0[b]
[Condition=Sham] * [Time=T2]	0[b]	0[b]	0[b]	0[b]
[Condition=taVNS] * [Time=T0]	0[b]	0[b]	0[b]	0[b]
[Condition=taVNS] * [Time=T1]	0[b]	0[b]	0[b]	0[b]
[Condition=taVNS] * [Time=T2]	0[b]	0[b]	0[b]	0[b]

Matrice de covariance pour les estimations des effets fixes[a]

Paramètre	[Condition=taVNS] * [Time=T0]	[Condition=taVNS] * [Time=T1]	[Condition=taVNS] * [Time=T2]
Constante	0[b]	0[b]	0[b]
[Condition=Sham]	0[b]	0[b]	0[b]
[Condition=taVNS]	0[b]	0[b]	0[b]
[Time=T0]	0[b]	0[b]	0[b]
[Time=T1]	0[b]	0[b]	0[b]
[Time=T2]	0[b]	0[b]	0[b]
[Condition=Sham] * [Time=T0]	0[b]	0[b]	0[b]
[Condition=Sham] * [Time=T1]	0[b]	0[b]	0[b]
[Condition=Sham] * [Time=T2]	0[b]	0[b]	0[b]
[Condition=taVNS] * [Time=T0]	0[b]	0[b]	0[b]
[Condition=taVNS] * [Time=T1]	0[b]	0[b]	0[b]
[Condition=taVNS] * [Time=T2]	0[b]	0[b]	0[b]

a. Variable dépendante : Cool-sensitive Aδ-fibers Thresholds.

b. La covariance est définie sur 0, car elle est associée à un paramètre redondant.

Paramètres de covariance

Estimations des paramètres de covariance[a]

Paramètre	Estimation	Erreur standard	Z de Wald	Sig.	Intervalle de confiance à 95 % Borne inférieure	Borne supérieure
Résidu	1,108748	,140247	7,906	,000	,865294	1,420700

a. Variable dépendante : Cool-sensitive Aδ-fibers Thresholds.

Matrice de corrélation pour les estimations des paramètres de covariance[a]

Paramètre	Résidu
Résidu	1

a. Variable dépendante : Cool-sensitive Aδ-fibers Thresholds.

Matrice de covariance pour les estimations des paramètres de covariance[a]

Paramètre	Résidu
Résidu	,019669

a. Variable dépendante : Cool-sensitive Aδ-fibers Thresholds.

Moyenne marginale estimée

1. Condition

Estimations[a]

Condition	Moyenne	Erreur standard	ddl	Intervalle de confiance à 95 % Borne inférieure	Borne supérieure
Sham	29,078	,131	125	28,820	29,337
taVNS	29,191	,130	125	28,935	29,448

a. Variable dépendante : Cool-sensitive Aδ-fibers Thresholds.

Comparaisons appariées[a]

(I) Condition	(J) Condition	Différence moyenne (I-J)	Erreur standard	ddl	Sig.[b]
Sham	taVNS	-,113	,184	125	,539
taVNS	Sham	,113	,184	125	,539

Comparaisons appariées[a]

(I) Condition	(J) Condition	Intervalle de confiance à 95 % pour la différence[b] Borne inférieure	Borne supérieure

Sham	taVNS	-,478	,251
taVNS	Sham	-,251	,478

Basées sur les moyennes marginales estimées[a]

a. Variable dépendante : Cool-sensitive Aδ-fibers Thresholds.

b. Ajustement pour les comparaisons multiples : Bonferroni.

Tests univariés[a]

Ddl du numérateur	Ddl du dénominateur	F	Sig.
1	125	,380	,539

Le test de F permet de tester l'effet de Condition. Il s'appuie sur les comparaisons appariées (indépendantes) linéaires parmi les moyennes marginales estimées.[a]

a. Variable dépendante : Cool-sensitive Aδ-fibers Thresholds.

2. Time

Estimations[a]

Time	Moyenne	Erreur standard	ddl	Intervalle de confiance à 95 % Borne inférieure	Borne supérieure
T0	29,675	,159	125	29,360	29,989
T1	28,859	,159	125	28,545	29,173
T2	28,870	,161	125	28,552	29,188

a. Variable dépendante : Cool-sensitive Aδ-fibers Thresholds.

Comparaisons appariées[a]

(I) Time	(J) Time	Différence moyenne (I-J)	Erreur standard	ddl	Sig.[c]	Intervalle de confiance à 95 % pour la différence[c] Borne inférieure
T0	T1	,815*	,224	125	,001	,271
	T2	,804*	,226	125	,002	,256
T1	T0	-,815*	,224	125	,001	-1,360
	T2	-,011	,226	125	1,000	-,559
T2	T0	-,804*	,226	125	,002	-1,352
	T1	,011	,226	125	1,000	-,537

Comparaisons appariées[a]

(I) Time	(J) Time	Intervalle de confiance à 95 % pour la différence<br>Borne supérieure
T0	T1	1,360
	T2	1,352
T1	T0	-,271
	T2	,537
T2	T0	-,256
	T1	,559

Basées sur les moyennes marginales estimées[a]

*. La différence moyenne est significative au niveau ,05.

a. Variable dépendante : Cool-sensitive Aδ-fibers Thresholds.

c. Ajustement pour les comparaisons multiples : Bonferroni.

Tests univariés[a]

Ddl du numérateur	Ddl du dénominateur	F	Sig.
2	125,000	8,644	,000

Le test de F permet de tester l'effet de Time. Il s'appuie sur les comparaisons appariées (indépendantes) linéaires parmi les moyennes marginales estimées.[a]

a. Variable dépendante : Cool-sensitive Aδ-fibers Thresholds.

3. Condition * Time[a]

Condition	Time	Moyenne	Erreur standard	ddl	Intervalle de confiance à 95 %<br>Borne inférieure	 Borne supérieure
Sham	T0	29,611	,224	125	29,166	30,055
	T1	28,808	,224	125	28,364	29,252
	T2	28,815	,230	125	28,361	29,270
taVNS	T0	29,739	,224	125	29,294	30,183
	T1	28,911	,224	125	28,466	29,355
	T2	28,925	,224	125	28,481	29,370

a. Variable dépendante : Cool-sensitive Aδ-fibers Thresholds.

1.5. Laser Intensity.

```
MIXED LaserIntensities BY Condition Time
  /CRITERIA=CIN(95) MXITER(100) MXSTEP(10) SCORING(1)
SINGULAR(0.000000000001) HCONVERGE(0,
    ABSOLUTE) LCONVERGE(0, ABSOLUTE) PCONVERGE(0.000001, ABSOLUTE)
  /FIXED=Condition Time Condition*Time | SSTYPE(3)
  /METHOD=REML
  /PRINT=CPS CORB COVB DESCRIPTIVES  SOLUTION TESTCOV
  /EMMEANS=TABLES(Condition) COMPARE ADJ(BONFERRONI)
  /EMMEANS=TABLES(Time) COMPARE ADJ(BONFERRONI)
  /EMMEANS=TABLES(Condition*Time) .
```

Remarques

Sortie obtenue		04-MAY-2021 13:45:28
Commentaires		
Entrée	Jeu de données actif	Jeu_de_données1
	Filtre	<sans>
	Pondération	<sans>
	Fichier scindé	<sans>
	N de lignes dans le fichier de travail	1048509
Gestion des valeurs manquantes	Définition de la valeur manquante	Les valeurs manquantes définies par l'utilisateur sont traitées comme étant manquantes.
	Observations utilisées	Les statistiques sont basées sur toutes les observations comportant des données valides pour toutes les variables du modèle.

Syntaxe		MIXED LaserIntensities BY Condition Time<br>/CRITERIA=CIN(95) MXITER(100) MXSTEP(10) SCORING(1) SINGULAR(0.000000000001) HCONVERGE(0, ABSOLUTE) LCONVERGE(0, ABSOLUTE) PCONVERGE(0.000001, ABSOLUTE)<br>/FIXED=Condition Time Condition*Time | SSTYPE(3)<br>/METHOD=REML<br>/PRINT=CPS CORB COVB DESCRIPTIVES SOLUTION TESTCOV<br>/EMMEANS=TABLES(Condition) COMPARE ADJ(BONFERRONI)<br>/EMMEANS=TABLES(Time) COMPARE ADJ(BONFERRONI)<br>/EMMEANS=TABLES(Condition*Time) .
Ressources	Temps de processeur	00:00:00,67
	Temps écoulé	00:00:00,67

Récapitulatif de traitement des observations

		Effectif	Pourcentage marginal
Condition	Sham	66	50,0%
	taVNS	66	50,0%
Time	T0	44	33,3%
	T1	44	33,3%
	T2	44	33,3%

Valide	132	100,0%
Exclues	1048377	
Total	1048509	

Statistiques descriptives

Laser Intensities

Condition	Time	Effectif	Moyenne	Ecart type	Coefficient de variation
Sham	T0	22	4,809480161681819	1,675675379001842	34,8%
	T1	22	4,641799677272728	1,775954340858245	38,3%
	T2	22	4,597645612272727	1,710231261915005	37,2%
	Total	66	4,682975150409091	1,696930303489445	36,2%
taVNS	T0	22	4,795870128181819	1,787966847953329	37,3%
	T1	22	4,757569740909091	1,808244992811797	38,0%
	T2	22	4,592567593181818	1,811399760719567	39,4%
	Total	66	4,715335820757574	1,776843128818465	37,7%
Total	T0	44	4,802675144931821	1,712478276839075	35,7%
	T1	44	4,699684709090909	1,772178196298003	37,7%
	T2	44	4,595106602727274	1,740940302990185	37,9%
	Total	132	4,699155485583334	1,730778661161254	36,8%

Dimension du modèle[a]

		Nombre de niveaux	Nombre de paramètres
Effets fixes	Constante	1	1
	Condition	2	1
	Time	3	2

Condition * Time	6	2
Résidu		1
Total	12	7

a. Variable dépendante : Laser Intensities.

Critères d'information[a]

Log de vraisemblance restreint -2	518,909
Critère d'information d'Akaike (AIC)	520,909
Critère de Hurvich et Tsai (AICC)	520,941
Critère de Bozdogan (CAIC)	524,745
Critère bayésien de Schwartz (BIC)	523,745

Les critères d'informations sont présentés en plus petit, disposant d'un meilleur format.[a]

a. Variable dépendante : Laser Intensities.

Effets fixes

Tests des effets fixes de type III[a]

Source	Ddl du numérateur	Ddl du dénominateur	F	Sig.
Constante	1	126	938,526	,000
Condition	1	126	,011	,916
Time	2	126	,153	,859
Condition * Time	2	126	,019	,982

a. Variable dépendante : Laser Intensities.

Estimations des effets fixes[a]

Paramètre	Estimation	Erreur standard	ddl	t	Sig.
Constante	4,592568	,375727	126	12,223	,000
[Condition=Sham]	,005078	,531358	126	,010	,992
[Condition=taVNS]	0[b]	0	.	.	.

[Time=T0]	,203303	,531358	126	,383	,703
[Time=T1]	,165002	,531358	126	,311	,757
[Time=T2]	0[b]	0	.	.	.
[Condition=Sham] * [Time=T0]	,008532	,751454	126	,011	,991
[Condition=Sham] * [Time=T1]	-,120848	,751454	126,000	-,161	,872
[Condition=Sham] * [Time=T2]	0[b]	0	.	.	.
[Condition=taVNS] * [Time=T0]	0[b]	0	.	.	.
[Condition=taVNS] * [Time=T1]	0[b]	0	.	.	.
[Condition=taVNS] * [Time=T2]	0[b]	0	.	.	.

Estimations des effets fixes[a]

	Intervalle de confiance à 95 %	
Paramètre	Borne inférieure	Borne supérieure
Constante	3,849015	5,336120
[Condition=Sham]	-1,046464	1,056620
[Condition=taVNS]	.	.
[Time=T0]	-,848240	1,254845
[Time=T1]	-,886540	1,216544
[Time=T2]	.	.
[Condition=Sham] * [Time=T0]	-1,478573	1,495637
[Condition=Sham] * [Time=T1]	-1,607953	1,366257
[Condition=Sham] * [Time=T2]	.	.
[Condition=taVNS] * [Time=T0]	.	.
[Condition=taVNS] * [Time=T1]	.	.
[Condition=taVNS] * [Time=T2]	.	.

a. Variable dépendante : Laser Intensities.

b. Ce paramètre est défini sur 0, car il est redondant.

Matrice de corrélation pour les estimations des effets fixes[a]

Paramètre	Constante	[Condition=Sham]	[Condition=taVNS]	[Time=T0]	[Time=T1]
Constante	1	-,707	.[b]	-,707	-,707
[Condition=Sham]	-,707	1	.[b]	,500	,500
[Condition=taVNS]	.[b]	.[b]	.[b]	.[b]	.[b]

[Time=T0]	-,707	,500	.[b]	1	,500
[Time=T1]	-,707	,500	.[b]	,500	1
[Time=T2]	.[b]	.[b]	.[b]	.[b]	.[b]
[Condition=Sham] * [Time=T0]	,500	-,707	.[b]	-,707	-,354
[Condition=Sham] * [Time=T1]	,500	-,707	.[b]	-,354	-,707
[Condition=Sham] * [Time=T2]	.[b]	.[b]	.[b]	.[b]	.[b]
[Condition=taVNS] * [Time=T0]	.[b]	.[b]	.[b]	.[b]	.[b]
[Condition=taVNS] * [Time=T1]	.[b]	.[b]	.[b]	.[b]	.[b]
[Condition=taVNS] * [Time=T2]	.[b]	.[b]	.[b]	.[b]	.[b]

Matrice de corrélation pour les estimations des effets fixes[a]

Paramètre	[Time=T2]	[Condition=Sham] * [Time=T0]	[Condition=Sham] * [Time=T1]	[Condition=Sham] * [Time=T2]
Constante	.[b]	,500	,500	.[b]
[Condition=Sham]	.[b]	-,707	-,707	.[b]
[Condition=taVNS]	.[b]	.[b]	.[b]	.[b]
[Time=T0]	.[b]	-,707	-,354	.[b]
[Time=T1]	.[b]	-,354	-,707	.[b]
[Time=T2]	.[b]	.[b]	.[b]	.[b]
[Condition=Sham] * [Time=T0]	.[b]	1	,500	.[b]
[Condition=Sham] * [Time=T1]	.[b]	,500	1	.[b]
[Condition=Sham] * [Time=T2]	.[b]	.[b]	.[b]	.[b]
[Condition=taVNS] * [Time=T0]	.[b]	.[b]	.[b]	.[b]
[Condition=taVNS] * [Time=T1]	.[b]	.[b]	.[b]	.[b]
[Condition=taVNS] * [Time=T2]	.[b]	.[b]	.[b]	.[b]

Matrice de corrélation pour les estimations des effets fixes[a]

Paramètre	[Condition=taVNS] * [Time=T0]	[Condition=taVNS] * [Time=T1]	[Condition=taVNS] * [Time=T2]
Constante	.[b]	.[b]	.[b]
[Condition=Sham]	.[b]	.[b]	.[b]
[Condition=taVNS]	.[b]	.[b]	.[b]
[Time=T0]	.[b]	.[b]	.[b]
[Time=T1]	.[b]	.[b]	.[b]
[Time=T2]	.[b]	.[b]	.[b]
[Condition=Sham] * [Time=T0]	.[b]	.[b]	.[b]

[Condition=Sham] * [Time=T1]	.[b]	.[b]	.[b]
[Condition=Sham] * [Time=T2]	.[b]	.[b]	.[b]
[Condition=taVNS] * [Time=T0]	.[b]	.[b]	.[b]
[Condition=taVNS] * [Time=T1]	.[b]	.[b]	.[b]
[Condition=taVNS] * [Time=T2]	.[b]	.[b]	.[b]

a. Variable dépendante : Laser Intensities.

b. La corrélation est manquante par défaut, car elle est associée à un paramètre redondant.

Matrice de covariance pour les estimations des effets fixes[a]

Paramètre	Constante	[Condition=Sham]	[Condition=taVNS]	[Time=T0]	[Time=T1]
Constante	,141171	-,141171	0[b]	-,141171	-,141171
[Condition=Sham]	-,141171	,282341	0[b]	,141171	,141171
[Condition=taVNS]	0[b]	0[b]	0[b]	0[b]	0[b]
[Time=T0]	-,141171	,141171	0[b]	,282341	,141171
[Time=T1]	-,141171	,141171	0[b]	,141171	,282341
[Time=T2]	0[b]	0[b]	0[b]	0[b]	0[b]
[Condition=Sham] * [Time=T0]	,141171	-,282341	0[b]	-,282341	-,141171
[Condition=Sham] * [Time=T1]	,141171	-,282341	0[b]	-,141171	-,282341
[Condition=Sham] * [Time=T2]	0[b]	0[b]	0[b]	0[b]	0[b]
[Condition=taVNS] * [Time=T0]	0[b]	0[b]	0[b]	0[b]	0[b]
[Condition=taVNS] * [Time=T1]	0[b]	0[b]	0[b]	0[b]	0[b]
[Condition=taVNS] * [Time=T2]	0[b]	0[b]	0[b]	0[b]	0[b]

Matrice de covariance pour les estimations des effets fixes[a]

Paramètre	[Time=T2]	[Condition=Sham] * [Time=T0]	[Condition=Sham] * [Time=T1]	[Condition=Sham] * [Time=T2]
Constante	0[b]	,141171	,141171	0[b]
[Condition=Sham]	0[b]	-,282341	-,282341	0[b]
[Condition=taVNS]	0[b]	0[b]	0[b]	0[b]
[Time=T0]	0[b]	-,282341	-,141171	0[b]
[Time=T1]	0[b]	-,141171	-,282341	0[b]
[Time=T2]	0[b]	0[b]	0[b]	0[b]
[Condition=Sham] * [Time=T0]	0[b]	,564683	,282341	0[b]

[Condition=Sham] * [Time=T1]	0[b]	,282341	,564683	0[b]
[Condition=Sham] * [Time=T2]	0[b]	0[b]	0[b]	0[b]
[Condition=taVNS] * [Time=T0]	0[b]	0[b]	0[b]	0[b]
[Condition=taVNS] * [Time=T1]	0[b]	0[b]	0[b]	0[b]
[Condition=taVNS] * [Time=T2]	0[b]	0[b]	0[b]	0[b]

Matrice de covariance pour les estimations des effets fixes[a]

Paramètre	[Condition=taVNS] * [Time=T0]	[Condition=taVNS] * [Time=T1]	[Condition=taVNS] * [Time=T2]
Constante	0[b]	0[b]	0[b]
[Condition=Sham]	0[b]	0[b]	0[b]
[Condition=taVNS]	0[b]	0[b]	0[b]
[Time=T0]	0[b]	0[b]	0[b]
[Time=T1]	0[b]	0[b]	0[b]
[Time=T2]	0[b]	0[b]	0[b]
[Condition=Sham] * [Time=T0]	0[b]	0[b]	0[b]
[Condition=Sham] * [Time=T1]	0[b]	0[b]	0[b]
[Condition=Sham] * [Time=T2]	0[b]	0[b]	0[b]
[Condition=taVNS] * [Time=T0]	0[b]	0[b]	0[b]
[Condition=taVNS] * [Time=T1]	0[b]	0[b]	0[b]
[Condition=taVNS] * [Time=T2]	0[b]	0[b]	0[b]

a. Variable dépendante : Laser Intensities.

b. La covariance est définie sur 0, car elle est associée à un paramètre redondant.

Paramètres de covariance

Estimations des paramètres de covariance[a]

Paramètre	Estimation	Erreur standard	Z de Wald	Sig.	Intervalle de confiance à 95 % Borne inférieure	Borne supérieure
Résidu	3,105756	,391289	7,937	,000	2,426197	3,975655

a. Variable dépendante : Laser Intensities.

Matrice de corrélation pour les estimations des paramètres de covariance[a]

Paramètre	Résidu
Résidu	1

a. Variable dépendante : Laser Intensities.

Matrice de covariance pour les estimations des paramètres de covariance[a]

Paramètre	Résidu
Résidu	,153107

a. Variable dépendante : Laser Intensities.

Moyenne marginale estimée

1. Condition

Estimations[a]

Condition	Moyenne	Erreur standard	ddl	Intervalle de confiance à 95 %	
				Borne inférieure	Borne supérieure
Sham	4,683	,217	126	4,254	5,112
taVNS	4,715	,217	126	4,286	5,145

a. Variable dépendante : Laser Intensities.

Comparaisons appariées[a]

(I) Condition	(J) Condition	Différence moyenne (I-J)	Erreur standard	ddl	Sig.[b]
Sham	taVNS	-,032	,307	126	,916
taVNS	Sham	,032	,307	126	,916

Comparaisons appariées[a]

(I) Condition	(J) Condition	Intervalle de confiance à 95 % pour la différence[b]	
		Borne inférieure	Borne supérieure
Sham	taVNS	-,639	,575
taVNS	Sham	-,575	,639

Basées sur les moyennes marginales estimées[a]

a. Variable dépendante : Laser Intensities.

b. Ajustement pour les comparaisons multiples : Bonferroni.

Tests univariés[a]

Ddl du numérateur	Ddl du dénominateur	F	Sig.
1	126	,011	,916

Le test de F permet de tester l'effet de Condition. Il s'appuie sur les comparaisons appariées (indépendantes) linéaires parmi les moyennes marginales estimées.[a]

a. Variable dépendante : Laser Intensities.

2. Time

Estimations[a]

Time	Moyenne	Erreur standard	ddl	Intervalle de confiance à 95 % Borne inférieure	Intervalle de confiance à 95 % Borne supérieure
T0	4,803	,266	126	4,277	5,328
T1	4,700	,266	126	4,174	5,225
T2	4,595	,266	126	4,069	5,121

a. Variable dépendante : Laser Intensities.

Comparaisons appariées[a]

(I) Time	(J) Time	Différence moyenne (I-J)	Erreur standard	ddl	Sig.[b]	Intervalle de confiance à 95 % pour la différence[b] Borne inférieure
T0	T1	,103	,376	126	1,000	-,809
	T2	,208	,376	126	1,000	-,704
T1	T0	-,103	,376	126	1,000	-1,015
	T2	,105	,376	126	1,000	-,807
T2	T0	-,208	,376	126	1,000	-1,119
	T1	-,105	,376	126	1,000	-1,016

Comparaisons appariées[a]

(I) Time	(J) Time	Intervalle de confiance à 95 % pour la différence Borne supérieure
T0	T1	1,015

	T2	1,119
T1	T0	,809
	T2	1,016
T2	T0	,704
	T1	,807

Basées sur les moyennes marginales estimées[a]

a. Variable dépendante : Laser Intensities.

b. Ajustement pour les comparaisons multiples : Bonferroni.

Tests univariés[a]

Ddl du numérateur	Ddl du dénominateur	F	Sig.
2	126	,153	,859

Le test de F permet de tester l'effet de Time. Il s'appuie sur les comparaisons appariées (indépendantes) linéaires parmi les moyennes marginales estimées.[a]

a. Variable dépendante : Laser Intensities.

3. Condition * Time[a]

Condition	Time	Moyenne	Erreur standard	ddl	Intervalle de confiance à 95 % Borne inférieure	Intervalle de confiance à 95 % Borne supérieure
Sham	T0	4,809	,376	126	4,066	5,553
	T1	4,642	,376	126	3,898	5,385
	T2	4,598	,376	126	3,854	5,341
taVNS	T0	4,796	,376	126	4,052	5,539
	T1	4,758	,376	126	4,014	5,501
	T2	4,593	,376	126	3,849	5,336

a. Variable dépendante : Laser Intensities.

1.6. Vibrotactile Intensity.

```
MIXED VibrotactileIntensity BY Condition Time
  /CRITERIA=CIN(95) MXITER(100) MXSTEP(10) SCORING(1)
SINGULAR(0.000000000001) HCONVERGE(0,
    ABSOLUTE) LCONVERGE(0, ABSOLUTE) PCONVERGE(0.000001, ABSOLUTE)
  /FIXED=Condition Time Condition*Time | SSTYPE(3)
  /METHOD=REML
  /PRINT=CPS CORB COVB DESCRIPTIVES  SOLUTION TESTCOV
  /EMMEANS=TABLES(Condition) COMPARE ADJ(BONFERRONI)
  /EMMEANS=TABLES(Time) COMPARE ADJ(BONFERRONI)
  /EMMEANS=TABLES(Condition*Time) .
```

Remarques

Sortie obtenue		04-MAY-2021 13:46:02
Commentaires		
Entrée	Jeu de données actif	Jeu_de_données1
	Filtre	<sans>
	Pondération	<sans>
	Fichier scindé	<sans>
	N de lignes dans le fichier de travail	1048509
Gestion des valeurs manquantes	Définition de la valeur manquante	Les valeurs manquantes définies par l'utilisateur sont traitées comme étant manquantes.
	Observations utilisées	Les statistiques sont basées sur toutes les observations comportant des données valides pour toutes les variables du modèle.

Syntaxe		MIXED VibrotactileIntensity BY Condition Time /CRITERIA=CIN(95) MXITER(100) MXSTEP(10) SCORING(1) SINGULAR(0.000000000001) HCONVERGE(0, ABSOLUTE) LCONVERGE(0, ABSOLUTE) PCONVERGE(0.000001, ABSOLUTE) /FIXED=Condition Time Condition*Time | SSTYPE(3) /METHOD=REML /PRINT=CPS CORB COVB DESCRIPTIVES SOLUTION TESTCOV /EMMEANS=TABLES(Condition) COMPARE ADJ(BONFERRONI) /EMMEANS=TABLES(Time) COMPARE ADJ(BONFERRONI) /EMMEANS=TABLES(Condition*Time) .
Ressources	Temps de processeur	00:00:00,67
	Temps écoulé	00:00:00,69

Récapitulatif de traitement des observations

		Effectif	Pourcentage marginal
Condition	Sham	65	49,6%
	taVNS	66	50,4%
Time	T0	43	32,8%
	T1	44	33,6%
	T2	44	33,6%

Valide	131	100,0%
Exclues	1048378	
Total	1048509	

Statistiques descriptives

Vibrotactile Intensity

Condition	Time	Effectif	Moyenne	Ecart type	Coefficient de variation
Sham	T0	21	4,325542115238096	1,968979549371218	45,5%
	T1	22	4,796920454545456	1,804432870583620	37,6%
	T2	22	4,634058713636365	1,968903806460639	42,5%
	Total	65	4,589506555692308	1,894790201769444	41,3%
taVNS	T0	22	5,107727272727273	2,095062067084187	41,0%
	T1	22	4,991818181818182	1,863970765056956	37,3%
	T2	22	5,117272727272727	1,820562921365079	35,6%
	Total	66	5,072272727272727	1,901235060492194	37,5%
Total	T0	43	4,725729870232558	2,048728031904973	43,4%
	T1	44	4,894369318181818	1,815665047681963	37,1%
	T2	44	4,875665720454546	1,889875818224731	38,8%
	Total	131	4,832732260458017	1,906188802296078	39,4%

Dimension du modèle[a]

		Nombre de niveaux	Nombre de paramètres
Effets fixes	Constante	1	1
	Condition	2	1
	Time	3	2
	Condition * Time	6	2
Résidu			1

Total	12	7

a. Variable dépendante : Vibrotactile Intensity.

Critères d'information[a]

Log de vraisemblance restreint -2	536,658
Critère d'information d'Akaike (AIC)	538,658
Critère de Hurvich et Tsai (AICC)	538,690
Critère de Bozdogan (CAIC)	542,486
Critère bayésien de Schwartz (BIC)	541,486

Les critères d'informations sont présentés en plus petit, disposant d'un meilleur format.[a]
a. Variable dépendante : Vibrotactile Intensity.

Effets fixes

Tests des effets fixes de type III[a]

Source	Ddl du numérateur	Ddl du dénominateur	F	Sig.
Constante	1	125	826,124	,000
Condition	1	125	2,099	,150
Time	2	125,000	,112	,894
Condition * Time	2	125,000	,254	,776

a. Variable dépendante : Vibrotactile Intensity.

Estimations des effets fixes[a]

Paramètre	Estimation	Erreur standard	ddl	t	Sig.
Constante	5,117273	,409905	125	12,484	,000
[Condition=Sham]	-,483214	,579694	125	-,834	,406
[Condition=taVNS]	0[b]	0	.	.	.
[Time=T0]	-,009545	,579694	125	-,016	,987
[Time=T1]	-,125455	,579694	125	-,216	,829
[Time=T2]	0[b]	0	.	.	.

[Condition=Sham] * [Time=T0]	-,298971	,824676	125	-,363	,718
[Condition=Sham] * [Time=T1]	,288316	,819811	125	,352	,726
[Condition=Sham] * [Time=T2]	0[b]	0	.	.	.
[Condition=taVNS] * [Time=T0]	0[b]	0	.	.	.
[Condition=taVNS] * [Time=T1]	0[b]	0	.	.	.
[Condition=taVNS] * [Time=T2]	0[b]	0	.	.	.

Estimations des effets fixes[a]

	Intervalle de confiance à 95 %	
Paramètre	Borne inférieure	Borne supérieure
Constante	4,306019	5,928527
[Condition=Sham]	-1,630500	,664072
[Condition=taVNS]	.	.
[Time=T0]	-1,156832	1,137741
[Time=T1]	-1,272741	1,021832
[Time=T2]	.	.
[Condition=Sham] * [Time=T0]	-1,931108	1,333166
[Condition=Sham] * [Time=T1]	-1,334191	1,910824
[Condition=Sham] * [Time=T2]	.	.
[Condition=taVNS] * [Time=T0]	.	.
[Condition=taVNS] * [Time=T1]	.	.
[Condition=taVNS] * [Time=T2]	.	.

a. Variable dépendante : Vibrotactile Intensity.

b. Ce paramètre est défini sur 0, car il est redondant.

Matrice de corrélation pour les estimations des effets fixes[a]

Paramètre	Constante	[Condition=Sham]	[Condition=taVNS]	[Time=T0]	[Time=T1]
Constante	1	-,707	.[b]	-,707	-,707
[Condition=Sham]	-,707	1	.[b]	,500	,500
[Condition=taVNS]	.[b]	.[b]	.[b]	.[b]	.[b]
[Time=T0]	-,707	,500	.[b]	1	,500
[Time=T1]	-,707	,500	.[b]	,500	1
[Time=T2]	.[b]	.[b]	.[b]	.[b]	.[b]

[Condition=Sham] * [Time=T0]	,497	-,703	.[b]	-,703	-,351
[Condition=Sham] * [Time=T1]	,500	-,707	.[b]	-,354	-,707
[Condition=Sham] * [Time=T2]	.[b]	.[b]	.[b]	.[b]	.[b]
[Condition=taVNS] * [Time=T0]	.[b]	.[b]	.[b]	.[b]	.[b]
[Condition=taVNS] * [Time=T1]	.[b]	.[b]	.[b]	.[b]	.[b]
[Condition=taVNS] * [Time=T2]	.[b]	.[b]	.[b]	.[b]	.[b]

Matrice de corrélation pour les estimations des effets fixes[a]

Paramètre	[Time=T2]	[Condition=Sham] * [Time=T0]	[Condition=Sham] * [Time=T1]	[Condition=Sham] * [Time=T2]
Constante	.[b]	,497	,500	.[b]
[Condition=Sham]	.[b]	-,703	-,707	.[b]
[Condition=taVNS]	.[b]	.[b]	.[b]	.[b]
[Time=T0]	.[b]	-,703	-,354	.[b]
[Time=T1]	.[b]	-,351	-,707	.[b]
[Time=T2]	.[b]	.[b]	.[b]	.[b]
[Condition=Sham] * [Time=T0]	.[b]	1	,497	.[b]
[Condition=Sham] * [Time=T1]	.[b]	,497	1	.[b]
[Condition=Sham] * [Time=T2]	.[b]	.[b]	.[b]	.[b]
[Condition=taVNS] * [Time=T0]	.[b]	.[b]	.[b]	.[b]
[Condition=taVNS] * [Time=T1]	.[b]	.[b]	.[b]	.[b]
[Condition=taVNS] * [Time=T2]	.[b]	.[b]	.[b]	.[b]

Matrice de corrélation pour les estimations des effets fixes[a]

Paramètre	[Condition=taVNS] * [Time=T0]	[Condition=taVNS] * [Time=T1]	[Condition=taVNS] * [Time=T2]
Constante	.[b]	.[b]	.[b]
[Condition=Sham]	.[b]	.[b]	.[b]
[Condition=taVNS]	.[b]	.[b]	.[b]
[Time=T0]	.[b]	.[b]	.[b]
[Time=T1]	.[b]	.[b]	.[b]
[Time=T2]	.[b]	.[b]	.[b]
[Condition=Sham] * [Time=T0]	.[b]	.[b]	.[b]
[Condition=Sham] * [Time=T1]	.[b]	.[b]	.[b]
[Condition=Sham] * [Time=T2]	.[b]	.[b]	.[b]
[Condition=taVNS] * [Time=T0]	.[b]	.[b]	.[b]

[Condition=taVNS] * [Time=T1]	.[b]	.[b]	.[b]
[Condition=taVNS] * [Time=T2]	.[b]	.[b]	.[b]

a. Variable dépendante : Vibrotactile Intensity.

b. La corrélation est manquante par défaut, car elle est associée à un paramètre redondant.

Matrice de covariance pour les estimations des effets fixes[a]

Paramètre	Constante	[Condition=Sham]	[Condition=taVNS]	[Time=T0]	[Time=T1]
Constante	,168023	-,168023	0[b]	-,168023	-,168023
[Condition=Sham]	-,168023	,336045	0[b]	,168023	,168023
[Condition=taVNS]	0[b]	0[b]	0[b]	0[b]	0[b]
[Time=T0]	-,168023	,168023	0[b]	,336045	,168023
[Time=T1]	-,168023	,168023	0[b]	,168023	,336045
[Time=T2]	0[b]	0[b]	0[b]	0[b]	0[b]
[Condition=Sham] * [Time=T0]	,168023	-,336045	0[b]	-,336045	-,168023
[Condition=Sham] * [Time=T1]	,168023	-,336045	0[b]	-,168023	-,336045
[Condition=Sham] * [Time=T2]	0[b]	0[b]	0[b]	0[b]	0[b]
[Condition=taVNS] * [Time=T0]	0[b]	0[b]	0[b]	0[b]	0[b]
[Condition=taVNS] * [Time=T1]	0[b]	0[b]	0[b]	0[b]	0[b]
[Condition=taVNS] * [Time=T2]	0[b]	0[b]	0[b]	0[b]	0[b]

Matrice de covariance pour les estimations des effets fixes[a]

Paramètre	[Time=T2]	[Condition=Sham] * [Time=T0]	[Condition=Sham] * [Time=T1]	[Condition=Sham] * [Time=T2]
Constante	0[b]	,168023	,168023	0[b]
[Condition=Sham]	0[b]	-,336045	-,336045	0[b]
[Condition=taVNS]	0[b]	0[b]	0[b]	0[b]
[Time=T0]	0[b]	-,336045	-,168023	0[b]
[Time=T1]	0[b]	-,168023	-,336045	0[b]
[Time=T2]	0[b]	0[b]	0[b]	0[b]
[Condition=Sham] * [Time=T0]	0[b]	,680091	,336045	0[b]
[Condition=Sham] * [Time=T1]	0[b]	,336045	,672090	0[b]
[Condition=Sham] * [Time=T2]	0[b]	0[b]	0[b]	0[b]
[Condition=taVNS] * [Time=T0]	0[b]	0[b]	0[b]	0[b]

[Condition=taVNS] * [Time=T1]	0[b]	0[b]	0[b]	0[b]
[Condition=taVNS] * [Time=T2]	0[b]	0[b]	0[b]	0[b]

Matrice de covariance pour les estimations des effets fixes[a]

Paramètre	[Condition=taVNS] * [Time=T0]	[Condition=taVNS] * [Time=T1]	[Condition=taVNS] * [Time=T2]
Constante	0[b]	0[b]	0[b]
[Condition=Sham]	0[b]	0[b]	0[b]
[Condition=taVNS]	0[b]	0[b]	0[b]
[Time=T0]	0[b]	0[b]	0[b]
[Time=T1]	0[b]	0[b]	0[b]
[Time=T2]	0[b]	0[b]	0[b]
[Condition=Sham] * [Time=T0]	0[b]	0[b]	0[b]
[Condition=Sham] * [Time=T1]	0[b]	0[b]	0[b]
[Condition=Sham] * [Time=T2]	0[b]	0[b]	0[b]
[Condition=taVNS] * [Time=T0]	0[b]	0[b]	0[b]
[Condition=taVNS] * [Time=T1]	0[b]	0[b]	0[b]
[Condition=taVNS] * [Time=T2]	0[b]	0[b]	0[b]

a. Variable dépendante : Vibrotactile Intensity.

b. La covariance est définie sur 0, car elle est associée à un paramètre redondant.

Paramètres de covariance

Estimations des paramètres de covariance[a]

Paramètre	Estimation	Erreur standard	Z de Wald	Sig.	Intervalle de confiance à 95 % Borne inférieure	Intervalle de confiance à 95 % Borne supérieure
Résidu	3,696495	,467574	7,906	,000	2,884833	4,736522

a. Variable dépendante : Vibrotactile Intensity.

Matrice de corrélation pour les estimations des paramètres de covariance[a]

Paramètre	Résidu
Résidu	1

a. Variable dépendante : Vibrotactile Intensity.

Matrice de covariance pour les estimations des paramètres de covariance[a]

Paramètre	Résidu
Résidu	,218625

a. Variable dépendante : Vibrotactile Intensity.

Moyenne marginale estimée

1. Condition

Estimations[a]

Condition	Moyenne	Erreur standard	ddl	Intervalle de confiance à 95 %	
				Borne inférieure	Borne supérieure
Sham	4,586	,239	125	4,113	5,058
taVNS	5,072	,237	125	4,604	5,541

a. Variable dépendante : Vibrotactile Intensity.

Comparaisons appariées[a]

(I) Condition	(J) Condition	Différence moyenne (I-J)	Erreur standard	ddl	Sig.[b]
Sham	taVNS	-,487	,336	125	,150
taVNS	Sham	,487	,336	125	,150

Comparaisons appariées[a]

(I) Condition	(J) Condition	Intervalle de confiance à 95 % pour la différence[b]	
		Borne inférieure	Borne supérieure
Sham	taVNS	-1,152	,178
taVNS	Sham	-,178	1,152

Basées sur les moyennes marginales estimées[a]

a. Variable dépendante : Vibrotactile Intensity.

b. Ajustement pour les comparaisons multiples : Bonferroni.

Tests univariés[a]

Ddl du numérateur	Ddl du dénominateur	F	Sig.
1	125	2,099	,150

Le test de F permet de tester l'effet de Condition. Il s'appuie sur les comparaisons appariées (indépendantes) linéaires parmi les moyennes marginales estimées.[a]

a. Variable dépendante : Vibrotactile Intensity.

2. Time

Estimations[a]

Time	Moyenne	Erreur standard	ddl	Intervalle de confiance à 95 %	
				Borne inférieure	Borne supérieure
T0	4,717	,293	125	4,136	5,297
T1	4,894	,290	125	4,321	5,468
T2	4,876	,290	125	4,302	5,449

a. Variable dépendante : Vibrotactile Intensity.

Comparaisons appariées[a]

(I) Time	(J) Time	Différence moyenne (I-J)	Erreur standard	ddl	Sig.[b]	Intervalle de confiance à 95 % pour la différence[b] Borne inférieure
T0	T1	-,178	,412	125	1,000	-1,178
	T2	-,159	,412	125	1,000	-1,160
T1	T0	,178	,412	125	1,000	-,823
	T2	,019	,410	125	1,000	-,976
T2	T0	,159	,412	125	1,000	-,842
	T1	-,019	,410	125	1,000	-1,013

Comparaisons appariées[a]

(I) Time	(J) Time	Intervalle de confiance à 95 % pour la différence
		Borne supérieure

T0	T1	,823
	T2	,842
T1	T0	1,178
	T2	1,013
T2	T0	1,160
	T1	,976

Basées sur les moyennes marginales estimées[a]

a. Variable dépendante : Vibrotactile Intensity.

b. Ajustement pour les comparaisons multiples : Bonferroni.

Tests univariés[a]

Ddl du numérateur	Ddl du dénominateur	F	Sig.
2	125,000	,112	,894

Le test de F permet de tester l'effet de Time. Il s'appuie sur les comparaisons appariées (indépendantes) linéaires parmi les moyennes marginales estimées.[a]

a. Variable dépendante : Vibrotactile Intensity.

3. Condition * Time[a]

					Intervalle de confiance à 95 %	
Condition	Time	Moyenne	Erreur standard	ddl	Borne inférieure	Borne supérieure
Sham	T0	4,326	,420	125	3,495	5,156
	T1	4,797	,410	125	3,986	5,608
	T2	4,634	,410	125	3,823	5,445
taVNS	T0	5,108	,410	125	4,296	5,919
	T1	4,992	,410	125	4,181	5,803
	T2	5,117	,410	125	4,306	5,929

a. Variable dépendante : Vibrotactile Intensity.

1.7.Cool Intensity.

```
MIXED CoolIntensity BY Condition Time
  /CRITERIA=CIN(95) MXITER(100) MXSTEP(10) SCORING(1)
SINGULAR(0.000000000001) HCONVERGE(0,
    ABSOLUTE) LCONVERGE(0, ABSOLUTE) PCONVERGE(0.000001, ABSOLUTE)
  /FIXED=Condition Time Condition*Time | SSTYPE(3)
  /METHOD=REML
  /PRINT=CPS CORB COVB DESCRIPTIVES  SOLUTION TESTCOV
  /EMMEANS=TABLES(Condition) COMPARE ADJ(BONFERRONI)
  /EMMEANS=TABLES(Time) COMPARE ADJ(BONFERRONI)
  /EMMEANS=TABLES(Condition*Time) .
```

Remarques

Sortie obtenue		04-MAY-2021 13:46:49
Commentaires		
Entrée	Jeu de données actif	Jeu_de_données1
	Filtre	<sans>
	Pondération	<sans>
	Fichier scindé	<sans>
	N de lignes dans le fichier de travail	1048509
Gestion des valeurs manquantes	Définition de la valeur manquante	Les valeurs manquantes définies par l'utilisateur sont traitées comme étant manquantes.
	Observations utilisées	Les statistiques sont basées sur toutes les observations comportant des données valides pour toutes les variables du modèle.

Syntaxe		MIXED CoolIntensity BY Condition Time<br>/CRITERIA=CIN(95) MXITER(100) MXSTEP(10) SCORING(1) SINGULAR(0.000000000001) HCONVERGE(0, ABSOLUTE) LCONVERGE(0, ABSOLUTE) PCONVERGE(0.000001, ABSOLUTE)<br>/FIXED=Condition Time Condition*Time | SSTYPE(3)<br>/METHOD=REML<br>/PRINT=CPS CORB COVB DESCRIPTIVES SOLUTION TESTCOV<br>/EMMEANS=TABLES(Condition) COMPARE ADJ(BONFERRONI)<br>/EMMEANS=TABLES(Time) COMPARE ADJ(BONFERRONI)<br>/EMMEANS=TABLES(Condition*Time) .
Ressources	Temps de processeur	00:00:00,69
	Temps écoulé	00:00:00,69

Récapitulatif de traitement des observations

		Effectif	Pourcentage marginal
Condition	Sham	66	50,0%
	taVNS	66	50,0%
Time	T0	44	33,3%
	T1	44	33,3%
	T2	44	33,3%

Valide	132	100,0%
Exclues	1048377	
Total	1048509	

Statistiques descriptives

Cool Intensity

Condition	Time	Effectif	Moyenne	Ecart type	Coefficient de variation
Sham	T0	22	3,732648601409091	1,452017627496125	38,9%
	T1	22	4,419321818181818	1,567797146498221	35,5%
	T2	22	4,885454545454545	1,677840455190419	34,3%
	Total	66	4,345808321681817	1,616302722591314	37,2%
taVNS	T0	22	4,338442349999999	1,698990573972106	39,2%
	T1	22	4,590523836363637	1,383433123392595	30,1%
	T2	22	4,719112648000000	1,493513459830401	31,6%
	Total	66	4,549359611454545	1,515569140056889	33,3%
Total	T0	44	4,035545475704546	1,591622355767313	39,4%
	T1	44	4,504922827272727	1,463762184666364	32,5%
	T2	44	4,802283596727271	1,572028151550315	32,7%
	Total	132	4,447583966568180	1,564094434287668	35,2%

Dimension du modèle[a]

		Nombre de niveaux	Nombre de paramètres
Effets fixes	Constante	1	1
	Condition	2	1
	Time	3	2

Condition * Time	6	2
Résidu		1
Total	12	7

a. Variable dépendante : Cool Intensity.

Critères d'information[a]

Log de vraisemblance restreint -2	486,537
Critère d'information d'Akaike (AIC)	488,537
Critère de Hurvich et Tsai (AICC)	488,570
Critère de Bozdogan (CAIC)	492,374
Critère bayésien de Schwartz (BIC)	491,374

Les critères d'informations sont présentés en plus petit, disposant d'un meilleur format.[a]

a. Variable dépendante : Cool Intensity.

Effets fixes

Tests des effets fixes de type III[a]

Source	Ddl du numérateur	Ddl du dénominateur	F	Sig.
Constante	1	126	1087,009	,000
Condition	1	126	,569	,452
Time	2	126	2,737	,069
Condition * Time	2	126	,686	,505

a. Variable dépendante : Cool Intensity.

Estimations des effets fixes[a]

Paramètre	Estimation	Erreur standard	ddl	t	Sig.
Constante	4,719113	,330433	126	14,282	,000
[Condition=Sham]	,166342	,467303	126	,356	,722
[Condition=taVNS]	0[b]	0	.	.	.
[Time=T0]	-,380670	,467303	126,000	-,815	,417
[Time=T1]	-,128589	,467303	126	-,275	,784

[Time=T2]	0[b]	0	.	.	.
[Condition=Sham] * [Time=T0]	-,772136	,660866	126	-1,168	,245
[Condition=Sham] * [Time=T1]	-,337544	,660866	126	-,511	,610
[Condition=Sham] * [Time=T2]	0[b]	0	.	.	.
[Condition=taVNS] * [Time=T0]	0[b]	0	.	.	.
[Condition=taVNS] * [Time=T1]	0[b]	0	.	.	.
[Condition=taVNS] * [Time=T2]	0[b]	0	.	.	.

Estimations des effets fixes[a]

	Intervalle de confiance à 95 %	
Paramètre	Borne inférieure	Borne supérieure
Constante	4,065196	5,373029
[Condition=Sham]	-,758436	1,091120
[Condition=taVNS]	.	.
[Time=T0]	-1,305448	,544108
[Time=T1]	-1,053367	,796189
[Time=T2]	.	.
[Condition=Sham] * [Time=T0]	-2,079969	,535698
[Condition=Sham] * [Time=T1]	-1,645378	,970290
[Condition=Sham] * [Time=T2]	.	.
[Condition=taVNS] * [Time=T0]	.	.
[Condition=taVNS] * [Time=T1]	.	.
[Condition=taVNS] * [Time=T2]	.	.

a. Variable dépendante : Cool Intensity.

b. Ce paramètre est défini sur 0, car il est redondant.

Matrice de corrélation pour les estimations des effets fixes[a]

Paramètre	Constante	[Condition=Sham]	[Condition=taVNS]	[Time=T0]	[Time=T1]
Constante	1	-,707	.[b]	-,707	-,707
[Condition=Sham]	-,707	1	.[b]	,500	,500
[Condition=taVNS]	.[b]	.[b]	.[b]	.[b]	.[b]
[Time=T0]	-,707	,500	.[b]	1	,500
[Time=T1]	-,707	,500	.[b]	,500	1

[Time=T2]	.[b]	.[b]	.[b]	.[b]	.[b]
[Condition=Sham] * [Time=T0]	,500	-,707	.[b]	-,707	-,354
[Condition=Sham] * [Time=T1]	,500	-,707	.[b]	-,354	-,707
[Condition=Sham] * [Time=T2]	.[b]	.[b]	.[b]	.[b]	.[b]
[Condition=taVNS] * [Time=T0]	.[b]	.[b]	.[b]	.[b]	.[b]
[Condition=taVNS] * [Time=T1]	.[b]	.[b]	.[b]	.[b]	.[b]
[Condition=taVNS] * [Time=T2]	.[b]	.[b]	.[b]	.[b]	.[b]

Matrice de corrélation pour les estimations des effets fixes[a]

Paramètre	[Time=T2]	[Condition=Sham] * [Time=T0]	[Condition=Sham] * [Time=T1]	[Condition=Sham] * [Time=T2]
Constante	.[b]	,500	,500	.[b]
[Condition=Sham]	.[b]	-,707	-,707	.[b]
[Condition=taVNS]	.[b]	.[b]	.[b]	.[b]
[Time=T0]	.[b]	-,707	-,354	.[b]
[Time=T1]	.[b]	-,354	-,707	.[b]
[Time=T2]	.[b]	.[b]	.[b]	.[b]
[Condition=Sham] * [Time=T0]	.[b]	1	,500	.[b]
[Condition=Sham] * [Time=T1]	.[b]	,500	1	.[b]
[Condition=Sham] * [Time=T2]	.[b]	.[b]	.[b]	.[b]
[Condition=taVNS] * [Time=T0]	.[b]	.[b]	.[b]	.[b]
[Condition=taVNS] * [Time=T1]	.[b]	.[b]	.[b]	.[b]
[Condition=taVNS] * [Time=T2]	.[b]	.[b]	.[b]	.[b]

Matrice de corrélation pour les estimations des effets fixes[a]

Paramètre	[Condition=taVNS] * [Time=T0]	[Condition=taVNS] * [Time=T1]	[Condition=taVNS] * [Time=T2]
Constante	.[b]	.[b]	.[b]
[Condition=Sham]	.[b]	.[b]	.[b]
[Condition=taVNS]	.[b]	.[b]	.[b]
[Time=T0]	.[b]	.[b]	.[b]
[Time=T1]	.[b]	.[b]	.[b]
[Time=T2]	.[b]	.[b]	.[b]
[Condition=Sham] * [Time=T0]	.[b]	.[b]	.[b]
[Condition=Sham] * [Time=T1]	.[b]	.[b]	.[b]
[Condition=Sham] * [Time=T2]	.[b]	.[b]	.[b]

[Condition=taVNS] * [Time=T0]	.[b]	.[b]	.[b]
[Condition=taVNS] * [Time=T1]	.[b]	.[b]	.[b]
[Condition=taVNS] * [Time=T2]	.[b]	.[b]	.[b]

a. Variable dépendante : Cool Intensity.

b. La corrélation est manquante par défaut, car elle est associée à un paramètre redondant.

Matrice de covariance pour les estimations des effets fixes[a]

Paramètre	Constante	[Condition=Sham]	[Condition=taVNS]	[Time=T0]	[Time=T1]
Constante	,109186	-,109186	0[b]	-,109186	-,109186
[Condition=Sham]	-,109186	,218372	0[b]	,109186	,109186
[Condition=taVNS]	0[b]	0[b]	0[b]	0[b]	0[b]
[Time=T0]	-,109186	,109186	0[b]	,218372	,109186
[Time=T1]	-,109186	,109186	0[b]	,109186	,218372
[Time=T2]	0[b]	0[b]	0[b]	0[b]	0[b]
[Condition=Sham] * [Time=T0]	,109186	-,218372	0[b]	-,218372	-,109186
[Condition=Sham] * [Time=T1]	,109186	-,218372	0[b]	-,109186	-,218372
[Condition=Sham] * [Time=T2]	0[b]	0[b]	0[b]	0[b]	0[b]
[Condition=taVNS] * [Time=T0]	0[b]	0[b]	0[b]	0[b]	0[b]
[Condition=taVNS] * [Time=T1]	0[b]	0[b]	0[b]	0[b]	0[b]
[Condition=taVNS] * [Time=T2]	0[b]	0[b]	0[b]	0[b]	0[b]

Matrice de covariance pour les estimations des effets fixes[a]

Paramètre	[Time=T2]	[Condition=Sham] * [Time=T0]	[Condition=Sham] * [Time=T1]	[Condition=Sham] * [Time=T2]
Constante	0[b]	,109186	,109186	0[b]
[Condition=Sham]	0[b]	-,218372	-,218372	0[b]
[Condition=taVNS]	0[b]	0[b]	0[b]	0[b]
[Time=T0]	0[b]	-,218372	-,109186	0[b]
[Time=T1]	0[b]	-,109186	-,218372	0[b]
[Time=T2]	0[b]	0[b]	0[b]	0[b]
[Condition=Sham] * [Time=T0]	0[b]	,436743	,218372	0[b]
[Condition=Sham] * [Time=T1]	0[b]	,218372	,436743	0[b]
[Condition=Sham] * [Time=T2]	0[b]	0[b]	0[b]	0[b]

[Condition=taVNS] * [Time=T0]	0[b]	0[b]	0[b]	0[b]
[Condition=taVNS] * [Time=T1]	0[b]	0[b]	0[b]	0[b]
[Condition=taVNS] * [Time=T2]	0[b]	0[b]	0[b]	0[b]

Matrice de covariance pour les estimations des effets fixes[a]

Paramètre	[Condition=taVNS] * [Time=T0]	[Condition=taVNS] * [Time=T1]	[Condition=taVNS] * [Time=T2]
Constante	0[b]	0[b]	0[b]
[Condition=Sham]	0[b]	0[b]	0[b]
[Condition=taVNS]	0[b]	0[b]	0[b]
[Time=T0]	0[b]	0[b]	0[b]
[Time=T1]	0[b]	0[b]	0[b]
[Time=T2]	0[b]	0[b]	0[b]
[Condition=Sham] * [Time=T0]	0[b]	0[b]	0[b]
[Condition=Sham] * [Time=T1]	0[b]	0[b]	0[b]
[Condition=Sham] * [Time=T2]	0[b]	0[b]	0[b]
[Condition=taVNS] * [Time=T0]	0[b]	0[b]	0[b]
[Condition=taVNS] * [Time=T1]	0[b]	0[b]	0[b]
[Condition=taVNS] * [Time=T2]	0[b]	0[b]	0[b]

a. Variable dépendante : Cool Intensity.

b. La covariance est définie sur 0, car elle est associée à un paramètre redondant.

Paramètres de covariance

Estimations des paramètres de covariance[a]

Paramètre	Estimation	Erreur standard	Z de Wald	Sig.	Intervalle de confiance à 95 %	
					Borne inférieure	Borne supérieure
Résidu	2,402088	,302635	7,937	,000	1,876496	3,074895

a. Variable dépendante : Cool Intensity.

Matrice de corrélation pour les estimations des paramètres de covariance[a]

Paramètre	Résidu
Résidu	1

a. Variable dépendante : Cool Intensity.

Matrice de covariance pour les estimations des paramètres de covariance[a]

Paramètre	Résidu
Résidu	,091588

a. Variable dépendante : Cool Intensity.

Moyenne marginale estimée

1. Condition

Estimations[a]

Condition	Moyenne	Erreur standard	ddl	Intervalle de confiance à 95 %	
				Borne inférieure	Borne supérieure
Sham	4,346	,191	126	3,968	4,723
taVNS	4,549	,191	126	4,172	4,927

a. Variable dépendante : Cool Intensity.

Comparaisons appariées[a]

(I) Condition	(J) Condition	Différence moyenne (I-J)	Erreur standard	ddl	Sig.[b]
Sham	taVNS	-,204	,270	126	,452
taVNS	Sham	,204	,270	126	,452

Comparaisons appariées[a]

(I) Condition	(J) Condition	Intervalle de confiance à 95 % pour la différence[b]	
		Borne inférieure	Borne supérieure
Sham	taVNS	-,737	,330
taVNS	Sham	-,330	,737

Basées sur les moyennes marginales estimées[a]

a. Variable dépendante : Cool Intensity.

b. Ajustement pour les comparaisons multiples : Bonferroni.

Tests univariés[a]

Ddl du numérateur	Ddl du dénominateur	F	Sig.
1	126	,569	,452

Le test de F permet de tester l'effet de Condition. Il s'appuie sur les comparaisons appariées (indépendantes) linéaires parmi les moyennes marginales estimées.[a]

a. Variable dépendante : Cool Intensity.

2. Time

Estimations[a]

Time	Moyenne	Erreur standard	ddl	Intervalle de confiance à 95 % Borne inférieure	Intervalle de confiance à 95 % Borne supérieure
T0	4,036	,234	126	3,573	4,498
T1	4,505	,234	126	4,043	4,967
T2	4,802	,234	126	4,340	5,265

a. Variable dépendante : Cool Intensity.

Comparaisons appariées[a]

(I) Time	(J) Time	Différence moyenne (I-J)	Erreur standard	ddl	Sig.[b]	Intervalle de confiance à 95 % pour la différence[b] Borne inférieure
T0	T1	-,469	,330	126	,474	-1,271
	T2	-,767	,330	126	,066	-1,568
T1	T0	,469	,330	126	,474	-,332
	T2	-,297	,330	126	1,000	-1,099
T2	T0	,767	,330	126	,066	-,035
	T1	,297	,330	126	1,000	-,504

Comparaisons appariées[a]

(I) Time	(J) Time	Intervalle de confiance à 95 % pour la différence Borne supérieure
T0	T1	,332
	T2	,035
T1	T0	1,271
	T2	,504
T2	T0	1,568
	T1	1,099

Basées sur les moyennes marginales estimées[a]

a. Variable dépendante : Cool Intensity.

b. Ajustement pour les comparaisons multiples : Bonferroni.

Tests univariés[a]

Ddl du numérateur	Ddl du dénominateur	F	Sig.
2	126	2,737	,069

Le test de F permet de tester l'effet de Time. Il s'appuie sur les comparaisons appariées (indépendantes) linéaires parmi les moyennes marginales estimées.[a]

a. Variable dépendante : Cool Intensity.

3. Condition * Time[a]

Condition	Time	Moyenne	Erreur standard	ddl	Intervalle de confiance à 95 % Borne inférieure	Borne supérieure
Sham	T0	3,733	,330	126	3,079	4,387
	T1	4,419	,330	126	3,765	5,073
	T2	4,885	,330	126	4,232	5,539
taVNS	T0	4,338	,330	126	3,685	4,992
	T1	4,591	,330	126	3,937	5,244
	T2	4,719	,330	126	4,065	5,373

a. Variable dépendante : Cool Intensity.

1.8.Pinprick Intensity.

```
MIXED PinprickIntensity BY Condition Time
  /CRITERIA=CIN(95) MXITER(100) MXSTEP(10) SCORING(1)
SINGULAR(0.000000000001) HCONVERGE(0,
    ABSOLUTE) LCONVERGE(0, ABSOLUTE) PCONVERGE(0.000001, ABSOLUTE)
  /FIXED=Condition Time Condition*Time | SSTYPE(3)
  /METHOD=REML
  /PRINT=CPS CORB COVB DESCRIPTIVES  SOLUTION TESTCOV
  /EMMEANS=TABLES(Condition) COMPARE ADJ(BONFERRONI)
  /EMMEANS=TABLES(Time) COMPARE ADJ(BONFERRONI)
  /EMMEANS=TABLES(Condition*Time) .
```

Remarques

Sortie obtenue		04-MAY-2021 13:47:18
Commentaires		
Entrée	Jeu de données actif	Jeu_de_données1
	Filtre	<sans>
	Pondération	<sans>
	Fichier scindé	<sans>
	N de lignes dans le fichier de travail	1048509
Gestion des valeurs manquantes	Définition de la valeur manquante	Les valeurs manquantes définies par l'utilisateur sont traitées comme étant manquantes.
	Observations utilisées	Les statistiques sont basées sur toutes les observations comportant des données valides pour toutes les variables du modèle.

Syntaxe		MIXED PinprickIntensity BY Condition Time<br>/CRITERIA=CIN(95) MXITER(100) MXSTEP(10) SCORING(1) SINGULAR(0.000000000001) HCONVERGE(0, ABSOLUTE) LCONVERGE(0, ABSOLUTE) PCONVERGE(0.000001, ABSOLUTE)<br>/FIXED=Condition Time Condition*Time | SSTYPE(3)<br>/METHOD=REML<br>/PRINT=CPS CORB COVB DESCRIPTIVES SOLUTION TESTCOV<br>/EMMEANS=TABLES(Condition) COMPARE ADJ(BONFERRONI)<br>/EMMEANS=TABLES(Time) COMPARE ADJ(BONFERRONI)<br>/EMMEANS=TABLES(Condition*Time) .
Ressources	Temps de processeur	00:00:00,70
	Temps écoulé	00:00:00,71

Récapitulatif de traitement des observations

		Effectif	Pourcentage marginal
Condition	Sham	66	50,0%
	taVNS	66	50,0%
Time	T0	44	33,3%
	T1	44	33,3%
	T2	44	33,3%

Valide	132	100,0%
Exclues	1048377	
Total	1048509	

Statistiques descriptives

Pinprick Intensity

Condition	Time	Effectif	Moyenne	Ecart type	Coefficient de variation
Sham	T0	22	3,154778552545455	1,620685501848566	51,4%
	T1	22	3,445568181818183	1,593867700008338	46,3%
	T2	22	3,273465909090909	1,548992468808459	47,3%
	Total	66	3,291270881151515	1,568121305423565	47,6%
taVNS	T0	22	3,005201633636364	1,641949674364796	54,6%
	T1	22	3,071534090909091	1,673895688377320	54,5%
	T2	22	3,169312354090910	1,587536525205402	50,1%
	Total	66	3,082016026212122	1,610933955745409	52,3%
Total	T0	44	3,079990093090909	1,614045405915863	52,4%
	T1	44	3,258551136363637	1,626296105275285	49,9%
	T2	44	3,221389131590909	1,550933516532217	48,1%
	Total	132	3,186643453681816	1,587071603799149	49,8%

Dimension du modèle[a]

		Nombre de niveaux	Nombre de paramètres
Effets fixes	Constante	1	1
	Condition	2	1
	Time	3	2

Condition * Time	6	2
Résidu		1
Total	12	7

a. Variable dépendante : Pinprick Intensity.

Critères d'information[a]

Log de vraisemblance restreint -2	496,389
Critère d'information d'Akaike (AIC)	498,389
Critère de Hurvich et Tsai (AICC)	498,421
Critère de Bozdogan (CAIC)	502,225
Critère bayésien de Schwartz (BIC)	501,225

Les critères d'informations sont présentés en plus petit, disposant d'un meilleur format.[a]

a. Variable dépendante : Pinprick Intensity.

Effets fixes

Tests des effets fixes de type III[a]

Source	Ddl du numérateur	Ddl du dénominateur	F	Sig.
Constante	1	126	516,055	,000
Condition	1	126	,556	,457
Time	2	126	,150	,861
Condition * Time	2	126	,088	,915

a. Variable dépendante : Pinprick Intensity.

Estimations des effets fixes[a]

Paramètre	Estimation	Erreur standard	ddl	t	Sig.
Constante	3,169312	,343606	126,000	9,224	,000
[Condition=Sham]	,104154	,485933	126,000	,214	,831
[Condition=taVNS]	0[b]	0	.	.	.
[Time=T0]	-,164111	,485933	126	-,338	,736

[Time=T1]	-,097778	,485933	126	-,201	,841
[Time=T2]	0[b]	0	.	.	.
[Condition=Sham] * [Time=T0]	,045423	,687212	126	,066	,947
[Condition=Sham] * [Time=T1]	,269881	,687212	126	,393	,695
[Condition=Sham] * [Time=T2]	0[b]	0	.	.	.
[Condition=taVNS] * [Time=T0]	0[b]	0	.	.	.
[Condition=taVNS] * [Time=T1]	0[b]	0	.	.	.
[Condition=taVNS] * [Time=T2]	0[b]	0	.	.	.

Estimations des effets fixes[a]

Paramètre	Intervalle de confiance à 95 % Borne inférieure	Borne supérieure
Constante	2,489326	3,849299
[Condition=Sham]	-,857493	1,065800
[Condition=taVNS]	.	.
[Time=T0]	-1,125757	,797536
[Time=T1]	-1,059425	,863868
[Time=T2]	.	.
[Condition=Sham] * [Time=T0]	-1,314550	1,405397
[Condition=Sham] * [Time=T1]	-1,090093	1,629854
[Condition=Sham] * [Time=T2]	.	.
[Condition=taVNS] * [Time=T0]	.	.
[Condition=taVNS] * [Time=T1]	.	.
[Condition=taVNS] * [Time=T2]	.	.

a. Variable dépendante : Pinprick Intensity.

b. Ce paramètre est défini sur 0, car il est redondant.

Matrice de corrélation pour les estimations des effets fixes[a]

Paramètre	Constante	[Condition=Sham]	[Condition=taVNS]	[Time=T0]	[Time=T1]
Constante	1	-,707	.[b]	-,707	-,707
[Condition=Sham]	-,707	1	.[b]	,500	,500
[Condition=taVNS]	.[b]	.[b]	.[b]	.[b]	.[b]
[Time=T0]	-,707	,500	.[b]	1	,500

[Time=T1]	-,707	,500	.[b]	,500	1
[Time=T2]	.[b]	.[b]	.[b]	.[b]	.[b]
[Condition=Sham] * [Time=T0]	,500	-,707	.[b]	-,707	-,354
[Condition=Sham] * [Time=T1]	,500	-,707	.[b]	-,354	-,707
[Condition=Sham] * [Time=T2]	.[b]	.[b]	.[b]	.[b]	.[b]
[Condition=taVNS] * [Time=T0]	.[b]	.[b]	.[b]	.[b]	.[b]
[Condition=taVNS] * [Time=T1]	.[b]	.[b]	.[b]	.[b]	.[b]
[Condition=taVNS] * [Time=T2]	.[b]	.[b]	.[b]	.[b]	.[b]

Matrice de corrélation pour les estimations des effets fixes[a]

Paramètre	[Time=T2]	[Condition=Sham] * [Time=T0]	[Condition=Sham] * [Time=T1]	[Condition=Sham] * [Time=T2]
Constante	.[b]	,500	,500	.[b]
[Condition=Sham]	.[b]	-,707	-,707	.[b]
[Condition=taVNS]	.[b]	.[b]	.[b]	.[b]
[Time=T0]	.[b]	-,707	-,354	.[b]
[Time=T1]	.[b]	-,354	-,707	.[b]
[Time=T2]	.[b]	.[b]	.[b]	.[b]
[Condition=Sham] * [Time=T0]	.[b]	1	,500	.[b]
[Condition=Sham] * [Time=T1]	.[b]	,500	1	.[b]
[Condition=Sham] * [Time=T2]	.[b]	.[b]	.[b]	.[b]
[Condition=taVNS] * [Time=T0]	.[b]	.[b]	.[b]	.[b]
[Condition=taVNS] * [Time=T1]	.[b]	.[b]	.[b]	.[b]
[Condition=taVNS] * [Time=T2]	.[b]	.[b]	.[b]	.[b]

Matrice de corrélation pour les estimations des effets fixes[a]

Paramètre	[Condition=taVNS] * [Time=T0]	[Condition=taVNS] * [Time=T1]	[Condition=taVNS] * [Time=T2]
Constante	.[b]	.[b]	.[b]
[Condition=Sham]	.[b]	.[b]	.[b]
[Condition=taVNS]	.[b]	.[b]	.[b]
[Time=T0]	.[b]	.[b]	.[b]
[Time=T1]	.[b]	.[b]	.[b]
[Time=T2]	.[b]	.[b]	.[b]
[Condition=Sham] * [Time=T0]	.[b]	.[b]	.[b]
[Condition=Sham] * [Time=T1]	.[b]	.[b]	.[b]

[Condition=Sham] * [Time=T2]	.[b]	.[b]	.[b]
[Condition=taVNS] * [Time=T0]	.[b]	.[b]	.[b]
[Condition=taVNS] * [Time=T1]	.[b]	.[b]	.[b]
[Condition=taVNS] * [Time=T2]	.[b]	.[b]	.[b]

a. Variable dépendante : Pinprick Intensity.

b. La corrélation est manquante par défaut, car elle est associée à un paramètre redondant.

Matrice de covariance pour les estimations des effets fixes[a]

Paramètre	Constante	[Condition=Sham]	[Condition=taVNS]	[Time=T0]	[Time=T1]
Constante	,118065	-,118065	0[b]	-,118065	-,118065
[Condition=Sham]	-,118065	,236130	0[b]	,118065	,118065
[Condition=taVNS]	0[b]	0[b]	0[b]	0[b]	0[b]
[Time=T0]	-,118065	,118065	0[b]	,236130	,118065
[Time=T1]	-,118065	,118065	0[b]	,118065	,236130
[Time=T2]	0[b]	0[b]	0[b]	0[b]	0[b]
[Condition=Sham] * [Time=T0]	,118065	-,236130	0[b]	-,236130	-,118065
[Condition=Sham] * [Time=T1]	,118065	-,236130	0[b]	-,118065	-,236130
[Condition=Sham] * [Time=T2]	0[b]	0[b]	0[b]	0[b]	0[b]
[Condition=taVNS] * [Time=T0]	0[b]	0[b]	0[b]	0[b]	0[b]
[Condition=taVNS] * [Time=T1]	0[b]	0[b]	0[b]	0[b]	0[b]
[Condition=taVNS] * [Time=T2]	0[b]	0[b]	0[b]	0[b]	0[b]

Matrice de covariance pour les estimations des effets fixes[a]

Paramètre	[Time=T2]	[Condition=Sham] * [Time=T0]	[Condition=Sham] * [Time=T1]	[Condition=Sham] * [Time=T2]
Constante	0[b]	,118065	,118065	0[b]
[Condition=Sham]	0[b]	-,236130	-,236130	0[b]
[Condition=taVNS]	0[b]	0[b]	0[b]	0[b]
[Time=T0]	0[b]	-,236130	-,118065	0[b]
[Time=T1]	0[b]	-,118065	-,236130	0[b]
[Time=T2]	0[b]	0[b]	0[b]	0[b]
[Condition=Sham] * [Time=T0]	0[b]	,472261	,236130	0[b]
[Condition=Sham] * [Time=T1]	0[b]	,236130	,472261	0[b]
[Condition=Sham] * [Time=T2]	0[b]	0[b]	0[b]	0[b]

[Condition=taVNS] * [Time=T0]	0[b]	0[b]	0[b]	0[b]
[Condition=taVNS] * [Time=T1]	0[b]	0[b]	0[b]	0[b]
[Condition=taVNS] * [Time=T2]	0[b]	0[b]	0[b]	0[b]

Matrice de covariance pour les estimations des effets fixes[a]

Paramètre	[Condition=taVNS] * [Time=T0]	[Condition=taVNS] * [Time=T1]	[Condition=taVNS] * [Time=T2]
Constante	0[b]	0[b]	0[b]
[Condition=Sham]	0[b]	0[b]	0[b]
[Condition=taVNS]	0[b]	0[b]	0[b]
[Time=T0]	0[b]	0[b]	0[b]
[Time=T1]	0[b]	0[b]	0[b]
[Time=T2]	0[b]	0[b]	0[b]
[Condition=Sham] * [Time=T0]	0[b]	0[b]	0[b]
[Condition=Sham] * [Time=T1]	0[b]	0[b]	0[b]
[Condition=Sham] * [Time=T2]	0[b]	0[b]	0[b]
[Condition=taVNS] * [Time=T0]	0[b]	0[b]	0[b]
[Condition=taVNS] * [Time=T1]	0[b]	0[b]	0[b]
[Condition=taVNS] * [Time=T2]	0[b]	0[b]	0[b]

a. Variable dépendante : Pinprick Intensity.

b. La covariance est définie sur 0, car elle est associée à un paramètre redondant.

Paramètres de covariance

Estimations des paramètres de covariance[a]

Paramètre	Estimation	Erreur standard	Z de Wald	Sig.	Intervalle de confiance à 95 %	
					Borne inférieure	Borne supérieure
Résidu	2,597435	,327246	7,937	,000	2,029100	3,324957

a. Variable dépendante : Pinprick Intensity.

Matrice de corrélation pour les estimations des paramètres de covariance[a]

Paramètre	Résidu
Résidu	1

a. Variable dépendante : Pinprick Intensity.

Matrice de covariance pour les estimations des paramètres de covariance[a]

Paramètre	Résidu
Résidu	,107090

a. Variable dépendante : Pinprick Intensity.

Moyenne marginale estimée

1. Condition

Estimations[a]

Condition	Moyenne	Erreur standard	ddl	Intervalle de confiance à 95 % Borne inférieure	Borne supérieure
Sham	3,291	,198	126	2,899	3,684
taVNS	3,082	,198	126	2,689	3,475

a. Variable dépendante : Pinprick Intensity.

Comparaisons appariées[a]

(I) Condition	(J) Condition	Différence moyenne (I-J)	Erreur standard	ddl	Sig.[b]
Sham	taVNS	,209	,281	126	,457
taVNS	Sham	-,209	,281	126	,457

Comparaisons appariées[a]

(I) Condition	(J) Condition	Intervalle de confiance à 95 % pour la différence[b] Borne inférieure	Borne supérieure
Sham	taVNS	-,346	,764
taVNS	Sham	-,764	,346

Basées sur les moyennes marginales estimées[a]

a. Variable dépendante : Pinprick Intensity.

b. Ajustement pour les comparaisons multiples : Bonferroni.

Tests univariés[a]

Ddl du numérateur	Ddl du dénominateur	F	Sig.
1	126	,556	,457

Le test de F permet de tester l'effet de Condition. Il s'appuie sur les comparaisons appariées (indépendantes) linéaires parmi les moyennes marginales estimées.[a]

a. Variable dépendante : Pinprick Intensity.

2. Time

Estimations[a]

Time	Moyenne	Erreur standard	ddl	Intervalle de confiance à 95 % Borne inférieure	Intervalle de confiance à 95 % Borne supérieure
T0	3,080	,243	126	2,599	3,561
T1	3,259	,243	126	2,778	3,739
T2	3,221	,243	126	2,741	3,702

a. Variable dépendante : Pinprick Intensity.

Comparaisons appariées[a]

(I) Time	(J) Time	Différence moyenne (I-J)	Erreur standard	ddl	Sig.[b]	Intervalle de confiance à 95 % pour la différence[b] Borne inférieure
T0	T1	-,179	,344	126	1,000	-1,012
	T2	-,141	,344	126	1,000	-,975
T1	T0	,179	,344	126	1,000	-,655
	T2	,037	,344	126	1,000	-,797
T2	T0	,141	,344	126	1,000	-,692
	T1	-,037	,344	126	1,000	-,871

Comparaisons appariées[a]

(I) Time	(J) Time	Intervalle de confiance à 95 % pour la différence Borne supérieure
T0	T1	,655
	T2	,692
T1	T0	1,012
	T2	,871
T2	T0	,975
	T1	,797

Basées sur les moyennes marginales estimées[a]

a. Variable dépendante : Pinprick Intensity.

b. Ajustement pour les comparaisons multiples : Bonferroni.

Tests univariés[a]

Ddl du numérateur	Ddl du dénominateur	F	Sig.
2	126	,150	,861

Le test de F permet de tester l'effet de Time. Il s'appuie sur les comparaisons appariées (indépendantes) linéaires parmi les moyennes marginales estimées.[a]

a. Variable dépendante : Pinprick Intensity.

3. Condition * Time[a]

Condition	Time	Moyenne	Erreur standard	ddl	Intervalle de confiance à 95 % Borne inférieure	Intervalle de confiance à 95 % Borne supérieure
Sham	T0	3,155	,344	126	2,475	3,835
	T1	3,446	,344	126	2,766	4,126
	T2	3,273	,344	126	2,593	3,953
taVNS	T0	3,005	,344	126	2,325	3,685
	T1	3,072	,344	126	2,392	3,752
	T2	3,169	,344	126,000	2,489	3,849

a. Variable dépendante : Pinprick Intensity.

2. Experiment 2:

2.1. Heat-sensitive C-fibers Detection Thresholds.

```
GET DATA
  /TYPE=XLSX
  /FILE='C:\Users\Mandumoulin\Documents\PhD 1ère année\TVNS and
pain\Articles\Review\Data\EEGs\Experiment 2\Experiment 2.xlsx'
  /SHEET=name 'Behavioral'
  /CELLRANGE=FULL
  /READNAMES=ON
  /DATATYPEMIN PERCENTAGE=95.0
  /HIDDEN IGNORE=YES.
EXECUTE.
DATASET NAME Jeu_de_données2 WINDOW=FRONT.
MIXED HeatsensitiveCfibersThresholds BY Condition Time
  /CRITERIA=CIN(95) MXITER(100) MXSTEP(10) SCORING(1)
SINGULAR(0.000000000001) HCONVERGE(0,
    ABSOLUTE) LCONVERGE(0, ABSOLUTE) PCONVERGE(0.000001, ABSOLUTE)
  /FIXED=Condition Time Condition*Time | SSTYPE(3)
  /METHOD=REML
  /PRINT=CPS CORB COVB DESCRIPTIVES G  SOLUTION TESTCOV
  /EMMEANS=TABLES(Condition) COMPARE ADJ(BONFERRONI)
  /EMMEANS=TABLES(Time) COMPARE ADJ(BONFERRONI)
  /EMMEANS=TABLES(Condition*Time) .
```

Remarques

Sortie obtenue		04-MAY-2021 13:51:34
Commentaires		
Entrée	Jeu de données actif	Jeu_de_données2
	Filtre	<sans>
	Pondération	<sans>
	Fichier scindé	<sans>
	N de lignes dans le fichier de travail	60
Gestion des valeurs manquantes	Définition de la valeur manquante	Les valeurs manquantes définies par l'utilisateur sont traitées comme étant manquantes.
	Observations utilisées	Les statistiques sont basées sur toutes les observations comportant des données valides pour toutes les variables du modèle.

Syntaxe		MIXED HeatsensitiveCfibersThresholds BY Condition Time /CRITERIA=CIN(95) MXITER(100) MXSTEP(10) SCORING(1) SINGULAR(0.000000000001) HCONVERGE(0, ABSOLUTE) LCONVERGE(0, ABSOLUTE) PCONVERGE(0.000001, ABSOLUTE) /FIXED=Condition Time Condition*Time | SSTYPE(3) /METHOD=REML /PRINT=CPS CORB COVB DESCRIPTIVES G SOLUTION TESTCOV /EMMEANS=TABLES(Condition) COMPARE ADJ(BONFERRONI) /EMMEANS=TABLES(Time) COMPARE ADJ(BONFERRONI) /EMMEANS=TABLES(Condition*Time) .
Ressources	Temps de processeur	00:00:00,02
	Temps écoulé	00:00:00,02

Récapitulatif de traitement des observations

		Effectif	Pourcentage marginal
Condition	Sham	29	51,8%
	taVNS	27	48,2%
Time	T0	29	51,8%
	T1	27	48,2%
Valide		56	100,0%

Exclues	4	
Total	60	

Statistiques descriptives

Heat-sensitive C-fibers Thresholds

Condition	Time	Effectif	Moyenne	Ecart type	Coefficient de variation
Sham	T0	15	41,133333333333340	2,108373329189614	5,1%
	T1	14	42,293714285714280	2,767469558683845	6,5%
	Total	29	41,693517241379320	2,475229247978712	5,9%
taVNS	T0	14	40,975000000000010	1,640561583023418	4,0%
	T1	13	41,519230769230774	1,631274867184465	3,9%
	Total	27	41,237037037037034	1,628094248866104	3,9%
Total	T0	29	41,056896551724140	1,865127539584428	4,5%
	T1	27	41,920814814814820	2,283228955752678	5,4%
	Total	56	41,473428571428580	2,103593270894031	5,1%

Dimension du modèle[a]

		Nombre de niveaux	Nombre de paramètres
Effets fixes	Constante	1	1
	Condition	2	1
	Time	2	1
	Condition * Time	4	1
Résidu			1
Total		9	5

a. Variable dépendante : Heat-sensitive C-fibers Thresholds.

Critères d'information[a]

Log de vraisemblance restreint -2	235,146
Critère d'information d'Akaike (AIC)	237,146
Critère de Hurvich et Tsai (AICC)	237,226
Critère de Bozdogan (CAIC)	240,097
Critère bayésien de Schwartz (BIC)	239,097

Les critères d'informations sont présentés en plus petit, disposant d'un meilleur format.[a]

a. Variable dépendante : Heat-sensitive C-fibers Thresholds.

Effets fixes

Tests des effets fixes de type III[a]

Source	Ddl du numérateur	Ddl du dénominateur	F	Sig.
Constante	1	52	21850,363	,000
Condition	1	52	,691	,410
Time	1	52	2,306	,135
Condition * Time	1	52	,301	,585

a. Variable dépendante : Heat-sensitive C-fibers Thresholds.

Estimations des effets fixes[a]

Paramètre	Estimation	Erreur standard	ddl	t	Sig.
Constante	41,519231	,581673	52	71,379	,000
[Condition=Sham]	,774484	,807787	52	,959	,342
[Condition=taVNS]	0[b]	0	.	.	.
[Time=T0]	-,544231	,807787	52	-,674	,503
[Time=T1]	0[b]	0	.	.	.
[Condition=Sham] * [Time=T0]	-,616150	1,122465	52	-,549	,585
[Condition=Sham] * [Time=T1]	0[b]	0	.	.	.

[Condition=taVNS] * [Time=T0]	0[b]	0	.	.	.
[Condition=taVNS] * [Time=T1]	0[b]	0	.	.	.

Estimations des effets fixes[a]

Paramètre	Intervalle de confiance à 95 % Borne inférieure	Borne supérieure
Constante	40,352018	42,686443
[Condition=Sham]	-,846460	2,395427
[Condition=taVNS]	.	.
[Time=T0]	-2,165174	1,076713
[Time=T1]	.	.
[Condition=Sham] * [Time=T0]	-2,868541	1,636240
[Condition=Sham] * [Time=T1]	.	.
[Condition=taVNS] * [Time=T0]	.	.
[Condition=taVNS] * [Time=T1]	.	.

a. Variable dépendante : Heat-sensitive C-fibers Thresholds.

b. Ce paramètre est défini sur 0, car il est redondant.

Matrice de corrélation pour les estimations des effets fixes[a]

Paramètre	Constante	[Condition=Sham]	[Condition=taVNS]	[Time=T0]	[Time=T1]
Constante	1	-,720	.[b]	-,720	.[b]
[Condition=Sham]	-,720	1	.[b]	,519	.[b]
[Condition=taVNS]	.[b]	.[b]	.[b]	.[b]	.[b]
[Time=T0]	-,720	,519	.[b]	1	.[b]
[Time=T1]	.[b]	.[b]	.[b]	.[b]	.[b]
[Condition=Sham] * [Time=T0]	,518	-,720	.[b]	-,720	.[b]
[Condition=Sham] * [Time=T1]	.[b]	.[b]	.[b]	.[b]	.[b]
[Condition=taVNS] * [Time=T0]	.[b]	.[b]	.[b]	.[b]	.[b]
[Condition=taVNS] * [Time=T1]	.[b]	.[b]	.[b]	.[b]	.[b]

Matrice de corrélation pour les estimations des effets fixes[a]

Paramètre	[Condition=Sham] * [Time=T0]	[Condition=Sham] * [Time=T1]	[Condition=taVNS] * [Time=T0]	[Condition=taVNS] * [Time=T1]
Constante	,518	.[b]	.[b]	.[b]

[Condition=Sham]	-,720	.[b]	.[b]	.[b]
[Condition=taVNS]	.[b]	.[b]	.[b]	.[b]
[Time=T0]	-,720	.[b]	.[b]	.[b]
[Time=T1]	.[b]	.[b]	.[b]	.[b]
[Condition=Sham] * [Time=T0]	1	.[b]	.[b]	.[b]
[Condition=Sham] * [Time=T1]	.[b]	.[b]	.[b]	.[b]
[Condition=taVNS] * [Time=T0]	.[b]	.[b]	.[b]	.[b]
[Condition=taVNS] * [Time=T1]	.[b]	.[b]	.[b]	.[b]

a. Variable dépendante : Heat-sensitive C-fibers Thresholds.

b. La corrélation est manquante par défaut, car elle est associée à un paramètre redondant.

Matrice de covariance pour les estimations des effets fixes[a]

Paramètre	Constante	[Condition=Sham]	[Condition=taVNS]	[Time=T0]	[Time=T1]
Constante	,338344	-,338344	0[b]	-,338344	0[b]
[Condition=Sham]	-,338344	,652520	0[b]	,338344	0[b]
[Condition=taVNS]	0[b]	0[b]	0[b]	0[b]	0[b]
[Time=T0]	-,338344	,338344	0[b]	,652520	0[b]
[Time=T1]	0[b]	0[b]	0[b]	0[b]	0[b]
[Condition=Sham] * [Time=T0]	,338344	-,652520	0[b]	-,652520	0[b]
[Condition=Sham] * [Time=T1]	0[b]	0[b]	0[b]	0[b]	0[b]
[Condition=taVNS] * [Time=T0]	0[b]	0[b]	0[b]	0[b]	0[b]
[Condition=taVNS] * [Time=T1]	0[b]	0[b]	0[b]	0[b]	0[b]

Matrice de covariance pour les estimations des effets fixes[a]

Paramètre	[Condition=Sham] * [Time=T0]	[Condition=Sham] * [Time=T1]	[Condition=taVNS] * [Time=T0]	[Condition=taVNS] * [Time=T1]
Constante	,338344	0[b]	0[b]	0[b]
[Condition=Sham]	-,652520	0[b]	0[b]	0[b]
[Condition=taVNS]	0[b]	0[b]	0[b]	0[b]
[Time=T0]	-,652520	0[b]	0[b]	0[b]
[Time=T1]	0[b]	0[b]	0[b]	0[b]
[Condition=Sham] * [Time=T0]	1,259927	0[b]	0[b]	0[b]
[Condition=Sham] * [Time=T1]	0[b]	0[b]	0[b]	0[b]
[Condition=taVNS] * [Time=T0]	0[b]	0[b]	0[b]	0[b]
[Condition=taVNS] * [Time=T1]	0[b]	0[b]	0[b]	0[b]

a. Variable dépendante : Heat-sensitive C-fibers Thresholds.

b. La covariance est définie sur 0, car elle est associée à un paramètre redondant.

Paramètres de covariance

Estimations des paramètres de covariance[a]

Paramètre	Estimation	Erreur standard	Z de Wald	Sig.	Intervalle de confiance à 95 %	
					Borne inférieure	Borne supérieure
Résidu	4,398468	,862610	5,099	,000	2,994795	6,460048

a. Variable dépendante : Heat-sensitive C-fibers Thresholds.

Matrice de corrélation pour les estimations des paramètres de covariance[a]

Paramètre	Résidu
Résidu	1

a. Variable dépendante : Heat-sensitive C-fibers Thresholds.

Matrice de covariance pour les estimations des paramètres de covariance[a]

Paramètre	Résidu
Résidu	,744097

a. Variable dépendante : Heat-sensitive C-fibers Thresholds.

Moyenne marginale estimée

1. Condition

Estimations[a]

Condition	Moyenne	Erreur standard	ddl	Intervalle de confiance à 95 % Borne inférieure	Borne supérieure
Sham	41,714	,390	52	40,932	42,495
taVNS	41,247	,404	52	40,437	42,058

a. Variable dépendante : Heat-sensitive C-fibers Thresholds.

Comparaisons appariées[a]

(I) Condition	(J) Condition	Différence moyenne (I-J)	Erreur standard	ddl	Sig.[b]
Sham	taVNS	,466	,561	52	,410
taVNS	Sham	-,466	,561	52	,410

Comparaisons appariées[a]

(I) Condition	(J) Condition	Intervalle de confiance à 95 % pour la différence[b] Borne inférieure	Borne supérieure
Sham	taVNS	-,660	1,593
taVNS	Sham	-1,593	,660

Basées sur les moyennes marginales estimées[a]

a. Variable dépendante : Heat-sensitive C-fibers Thresholds.

b. Ajustement pour les comparaisons multiples : Bonferroni.

Tests univariés[a]

Ddl du numérateur	Ddl du dénominateur	F	Sig.
1	52	,691	,410

Le test de F permet de tester l'effet de Condition. Il s'appuie sur les comparaisons appariées (indépendantes) linéaires parmi les moyennes marginales estimées.[a]

a. Variable dépendante : Heat-sensitive C-fibers Thresholds.

2. Time

Estimations[a]

Time	Moyenne	Erreur standard	ddl	Intervalle de confiance à 95 % Borne inférieure	Intervalle de confiance à 95 % Borne supérieure
T0	41,054	,390	52	40,272	41,836
T1	41,906	,404	52	41,096	42,717

a. Variable dépendante : Heat-sensitive C-fibers Thresholds.

Comparaisons appariées[a]

(I) Time	(J) Time	Différence moyenne (I-J)	Erreur standard	ddl	Sig.[b]	Intervalle de confiance à 95 % pour la différence[b] Borne inférieure
T0	T1	-,852	,561	52	,135	-1,979
T1	T0	,852	,561	52	,135	-,274

Comparaisons appariées[a]

(I) Time	(J) Time	Intervalle de confiance à 95 % pour la différence Borne supérieure
T0	T1	,274
T1	T0	1,979

Basées sur les moyennes marginales estimées[a]

a. Variable dépendante : Heat-sensitive C-fibers Thresholds.

b. Ajustement pour les comparaisons multiples : Bonferroni.

Tests univariés[a]

Ddl du numérateur	Ddl du dénominateur	F	Sig.
1	52	2,306	,135

Le test de F permet de tester l'effet de Time. Il s'appuie sur les comparaisons appariées (indépendantes) linéaires parmi les moyennes marginales estimées.[a]

a. Variable dépendante : Heat-sensitive C-fibers Thresholds.

3. Condition * Time[a]

Condition	Time	Moyenne	Erreur standard	ddl	Intervalle de confiance à 95 %

					Borne inférieure	Borne supérieure
Sham	T0	41,133	,542	52	40,047	42,220
	T1	42,294	,561	52	41,169	43,418
taVNS	T0	40,975	,561	52	39,850	42,100
	T1	41,519	,582	52	40,352	42,686

a. Variable dépendante : Heat-sensitive C-fibers Thresholds.

2.2 Heat-sensitive Aδ-fibers Detection Thresholds.

```
MIXED HeatsensitiveAδfibersThresholds BY Condition Time
  /CRITERIA=CIN(95) MXITER(100) MXSTEP(10) SCORING(1)
SINGULAR(0.000000000001) HCONVERGE(0,
    ABSOLUTE) LCONVERGE(0, ABSOLUTE) PCONVERGE(0.000001, ABSOLUTE)
  /FIXED=Condition Time Condition*Time | SSTYPE(3)
  /METHOD=REML
  /PRINT=CPS CORB COVB DESCRIPTIVES G  SOLUTION TESTCOV
  /EMMEANS=TABLES(Condition) COMPARE ADJ(BONFERRONI)
  /EMMEANS=TABLES(Time) COMPARE ADJ(BONFERRONI)
  /EMMEANS=TABLES(Condition*Time) .
```

Remarques

Sortie obtenue		04-MAY-2021 13:53:21
Commentaires		
Entrée	Jeu de données actif	Jeu_de_données2
	Filtre	<sans>
	Pondération	<sans>
	Fichier scindé	<sans>
	N de lignes dans le fichier de travail	60
Gestion des valeurs manquantes	Définition de la valeur manquante	Les valeurs manquantes définies par l'utilisateur sont traitées comme étant manquantes.
	Observations utilisées	Les statistiques sont basées sur toutes les observations comportant des données valides pour toutes les variables du modèle.

Syntaxe		MIXED HeatsensitiveAδfibersThresholds BY Condition Time<br>/CRITERIA=CIN(95) MXITER(100) MXSTEP(10) SCORING(1) SINGULAR(0.000000000001) HCONVERGE(0, ABSOLUTE) LCONVERGE(0, ABSOLUTE) PCONVERGE(0.000001, ABSOLUTE)<br>/FIXED=Condition Time Condition*Time | SSTYPE(3)<br>/METHOD=REML<br>/PRINT=CPS CORB COVB DESCRIPTIVES G SOLUTION TESTCOV<br><br>/EMMEANS=TABLES(Condition) COMPARE ADJ(BONFERRONI)<br><br>/EMMEANS=TABLES(Time) COMPARE ADJ(BONFERRONI)<br><br>/EMMEANS=TABLES(Condition*Time) .
Ressources	Temps de processeur	00:00:00,02
	Temps écoulé	00:00:00,02

Récapitulatif de traitement des observations

		Effectif	Pourcentage marginal
Condition	Sham	29	51,8%
	taVNS	27	48,2%
Time	T0	29	51,8%
	T1	27	48,2%
Valide		56	100,0%

Exclues	4	
Total	60	

Statistiques descriptives

Heat-sensitive Aδ-fibers Thresholds

Condition	Time	Effectif	Moyenne	Ecart type	Coefficient de variation
Sham	T0	15	50,135333	1,6291556	3,2%
	T1	14	49,868214	1,8131259	3,6%
	Total	29	50,006379	1,6946480	3,4%
taVNS	T0	14	49,322857	1,9446353	3,9%
	T1	13	49,479038	2,0671381	4,2%
	Total	27	49,398056	1,9670559	4,0%
Total	T0	29	49,743103	1,8037572	3,6%
	T1	27	49,680833	1,9118476	3,8%
	Total	56	49,713080	1,8398988	3,7%

Dimension du modèle[a]

		Nombre de niveaux	Nombre de paramètres
Effets fixes	Constante	1	1
	Condition	2	1
	Time	2	1
	Condition * Time	4	1
Résidu			1
Total		9	5

a. Variable dépendante : Heat-sensitive Aδ-fibers Thresholds.

Critères d'information[a]

Log de vraisemblance restreint -2	222,786
Critère d'information d'Akaike (AIC)	224,786
Critère de Hurvich et Tsai (AICC)	224,866
Critère de Bozdogan (CAIC)	227,737
Critère bayésien de Schwartz (BIC)	226,737

Les critères d'informations sont présentés en plus petit, disposant d'un meilleur format.[a]

a. Variable dépendante : Heat-sensitive Aδ-fibers Thresholds.

Effets fixes

Tests des effets fixes de type III[a]

Source	Ddl du numérateur	Ddl du dénominateur	F	Sig.
Constante	1	52	39787,118	,000
Condition	1	52	1,454	,233
Time	1	52	,012	,912
Condition * Time	1	52	,180	,673

a. Variable dépendante : Heat-sensitive Aδ-fibers Thresholds.

Estimations des effets fixes[a]

Paramètre	Estimation	Erreur standard	ddl	t	Sig.
Constante	49,479038	,516492	52	95,798	,000
[Condition=Sham]	,389176	,717268	52	,543	,590
[Condition=taVNS]	0[b]	0	.	.	.
[Time=T0]	-,156181	,717268	52	-,218	,828
[Time=T1]	0[b]	0	.	.	.
[Condition=Sham] * [Time=T0]	,423300	,996683	52	,425	,673
[Condition=Sham] * [Time=T1]	0[b]	0	.	.	.
[Condition=taVNS] * [Time=T0]	0[b]	0	.	.	.
[Condition=taVNS] * [Time=T1]	0[b]	0	.	.	.

Estimations des effets fixes[a]

Paramètre	Intervalle de confiance à 95 % Borne inférieure	Borne supérieure
Constante	48,442622	50,515455
[Condition=Sham]	-1,050127	1,828478
[Condition=taVNS]	.	.
[Time=T0]	-1,595484	1,283121

[Time=T1]	.	.
[Condition=Sham] * [Time=T0]	-1,576690	2,423291
[Condition=Sham] * [Time=T1]	.	.
[Condition=taVNS] * [Time=T0]	.	.
[Condition=taVNS] * [Time=T1]	.	.

a. Variable dépendante : Heat-sensitive Aδ-fibers Thresholds.

b. Ce paramètre est défini sur 0, car il est redondant.

Matrice de corrélation pour les estimations des effets fixes[a]

Paramètre	Constante	[Condition=Sham]	[Condition=taVNS]	[Time=T0]	[Time=T1]
Constante	1	-,720	.[b]	-,720	.[b]
[Condition=Sham]	-,720	1	.[b]	,519	.[b]
[Condition=taVNS]	.[b]	.[b]	.[b]	.[b]	.[b]
[Time=T0]	-,720	,519	.[b]	1	.[b]
[Time=T1]	.[b]	.[b]	.[b]	.[b]	.[b]
[Condition=Sham] * [Time=T0]	,518	-,720	.[b]	-,720	.[b]
[Condition=Sham] * [Time=T1]	.[b]	.[b]	.[b]	.[b]	.[b]
[Condition=taVNS] * [Time=T0]	.[b]	.[b]	.[b]	.[b]	.[b]
[Condition=taVNS] * [Time=T1]	.[b]	.[b]	.[b]	.[b]	.[b]

Matrice de corrélation pour les estimations des effets fixes[a]

Paramètre	[Condition=Sham] * [Time=T0]	[Condition=Sham] * [Time=T1]	[Condition=taVNS] * [Time=T0]	[Condition=taVNS] * [Time=T1]
Constante	,518	.[b]	.[b]	.[b]
[Condition=Sham]	-,720	.[b]	.[b]	.[b]
[Condition=taVNS]	.[b]	.[b]	.[b]	.[b]
[Time=T0]	-,720	.[b]	.[b]	.[b]
[Time=T1]	.[b]	.[b]	.[b]	.[b]
[Condition=Sham] * [Time=T0]	1	.[b]	.[b]	.[b]
[Condition=Sham] * [Time=T1]	.[b]	.[b]	.[b]	.[b]
[Condition=taVNS] * [Time=T0]	.[b]	.[b]	.[b]	.[b]
[Condition=taVNS] * [Time=T1]	.[b]	.[b]	.[b]	.[b]

a. Variable dépendante : Heat-sensitive Aδ-fibers Thresholds.

b. La corrélation est manquante par défaut, car elle est associée à un paramètre redondant.

Matrice de covariance pour les estimations des effets fixes[a]

Paramètre	Constante	[Condition=Sham]	[Condition=taVNS]	[Time=T0]	[Time=T1]
Constante	,266764	-,266764	0[b]	-,266764	0[b]
[Condition=Sham]	-,266764	,514473	0[b]	,266764	0[b]
[Condition=taVNS]	0[b]	0[b]	0[b]	0[b]	0[b]
[Time=T0]	-,266764	,266764	0[b]	,514473	0[b]
[Time=T1]	0[b]	0[b]	0[b]	0[b]	0[b]
[Condition=Sham] * [Time=T0]	,266764	-,514473	0[b]	-,514473	0[b]
[Condition=Sham] * [Time=T1]	0[b]	0[b]	0[b]	0[b]	0[b]
[Condition=taVNS] * [Time=T0]	0[b]	0[b]	0[b]	0[b]	0[b]
[Condition=taVNS] * [Time=T1]	0[b]	0[b]	0[b]	0[b]	0[b]

Matrice de covariance pour les estimations des effets fixes[a]

Paramètre	[Condition=Sham] * [Time=T0]	[Condition=Sham] * [Time=T1]	[Condition=taVNS] * [Time=T0]	[Condition=taVNS] * [Time=T1]
Constante	,266764	0[b]	0[b]	0[b]
[Condition=Sham]	-,514473	0[b]	0[b]	0[b]
[Condition=taVNS]	0[b]	0[b]	0[b]	0[b]
[Time=T0]	-,514473	0[b]	0[b]	0[b]
[Time=T1]	0[b]	0[b]	0[b]	0[b]
[Condition=Sham] * [Time=T0]	,993377	0[b]	0[b]	0[b]
[Condition=Sham] * [Time=T1]	0[b]	0[b]	0[b]	0[b]
[Condition=taVNS] * [Time=T0]	0[b]	0[b]	0[b]	0[b]
[Condition=taVNS] * [Time=T1]	0[b]	0[b]	0[b]	0[b]

a. Variable dépendante : Heat-sensitive Aδ-fibers Thresholds.

b. La covariance est définie sur 0, car elle est associée à un paramètre redondant.

Paramètres de covariance

Estimations des paramètres de covariance[a]

Paramètre	Estimation	Erreur standard	Z de Wald	Sig.	Intervalle de confiance à 95 % Borne inférieure	Intervalle de confiance à 95 % Borne supérieure
Résidu	3,467927	,680116	5,099	,000	2,361215	5,093359

a. Variable dépendante : Heat-sensitive Aδ-fibers Thresholds.

Matrice de corrélation pour les estimations des paramètres de covariance[a]

Paramètre	Résidu
Résidu	1

a. Variable dépendante : Heat-sensitive Aδ-fibers Thresholds.

Matrice de covariance pour les estimations des paramètres de covariance[a]

Paramètre	Résidu
Résidu	,462558

a. Variable dépendante : Heat-sensitive Aδ-fibers Thresholds.

Moyenne marginale estimée

1. Condition

Estimations[a]

Condition	Moyenne	Erreur standard	ddl	Intervalle de confiance à 95 % Borne inférieure	Intervalle de confiance à 95 % Borne supérieure
Sham	50,002	,346	52	49,307	50,696
taVNS	49,401	,359	52	48,681	50,121

a. Variable dépendante : Heat-sensitive Aδ-fibers Thresholds.

Comparaisons appariées[a]

(I) Condition	(J) Condition	Différence moyenne (I-J)	Erreur standard	ddl	Sig.[b]
Sham	taVNS	,601	,498	52	,233
taVNS	Sham	-,601	,498	52	,233

Comparaisons appariées[a]

(I) Condition	(J) Condition	Intervalle de confiance à 95 % pour la différence[b] Borne inférieure	Borne supérieure
Sham	taVNS	-,399	1,601
taVNS	Sham	-1,601	,399

Basées sur les moyennes marginales estimées[a]

a. Variable dépendante : Heat-sensitive Aδ-fibers Thresholds.

b. Ajustement pour les comparaisons multiples : Bonferroni.

Tests univariés[a]

Ddl du numérateur	Ddl du dénominateur	F	Sig.
1	52	1,454	,233

Le test de F permet de tester l'effet de Condition. Il s'appuie sur les comparaisons appariées (indépendantes) linéaires parmi les moyennes marginales estimées.[a]

a. Variable dépendante : Heat-sensitive Aδ-fibers Thresholds.

2. Time

Estimations[a]

Time	Moyenne	Erreur standard	ddl	Intervalle de confiance à 95 % Borne inférieure	Borne supérieure
T0	49,729	,346	52	49,035	50,423
T1	49,674	,359	52	48,954	50,393

a. Variable dépendante : Heat-sensitive Aδ-fibers Thresholds.

Comparaisons appariées[a]

(I) Time	(J) Time	Différence moyenne (I-J)	Erreur standard	ddl	Sig.[b]	Intervalle de confiance à 95 % pour la différence[b] Borne inférieure
T0	T1	,055	,498	52	,912	-,945
T1	T0	-,055	,498	52	,912	-1,055

Comparaisons appariées[a]

(I) Time	(J) Time	Intervalle de confiance à 95 % pour la différence Borne supérieure
T0	T1	1,055
T1	T0	,945

Basées sur les moyennes marginales estimées[a]

a. Variable dépendante : Heat-sensitive Aδ-fibers Thresholds.

b. Ajustement pour les comparaisons multiples : Bonferroni.

Tests univariés[a]

Ddl du numérateur	Ddl du dénominateur	F	Sig.
1	52	,012	,912

Le test de F permet de tester l'effet de Time. Il s'appuie sur les comparaisons appariées (indépendantes) linéaires parmi les moyennes marginales estimées.[a]

a. Variable dépendante : Heat-sensitive Aδ-fibers Thresholds.

3. Condition * Time[a]

Condition	Time	Moyenne	Erreur standard	ddl	Intervalle de confiance à 95 % Borne inférieure	Borne supérieure
Sham	T0	50,135	,481	52	49,170	51,100
	T1	49,868	,498	52	48,869	50,867
taVNS	T0	49,323	,498	52	48,324	50,322
	T1	49,479	,516	52	48,443	50,515

a. Variable dépendante : Heat-sensitive Aδ-fibers Thresholds.

2.3 Mechanosensitive Aβ-fibers Detection Thresholds.

```
MIXED MechanosensitiveAβfibersThresholds BY Condition Time
  /CRITERIA=CIN(95) MXITER(100) MXSTEP(10) SCORING(1)
SINGULAR(0.000000000001) HCONVERGE(0,
    ABSOLUTE) LCONVERGE(0, ABSOLUTE) PCONVERGE(0.000001, ABSOLUTE)
  /FIXED=Condition Time Condition*Time | SSTYPE(3)
  /METHOD=REML
  /PRINT=CPS CORB COVB DESCRIPTIVES G  SOLUTION TESTCOV
  /EMMEANS=TABLES(Condition) COMPARE ADJ(BONFERRONI)
  /EMMEANS=TABLES(Time) COMPARE ADJ(BONFERRONI)
  /EMMEANS=TABLES(Condition*Time) .
```

Remarques

Sortie obtenue		04-MAY-2021 13:54:11
Commentaires		
Entrée	Jeu de données actif	Jeu_de_données2
	Filtre	<sans>
	Pondération	<sans>
	Fichier scindé	<sans>
	N de lignes dans le fichier de travail	60
Gestion des valeurs manquantes	Définition de la valeur manquante	Les valeurs manquantes définies par l'utilisateur sont traitées comme étant manquantes.
	Observations utilisées	Les statistiques sont basées sur toutes les observations comportant des données valides pour toutes les variables du modèle.

Syntaxe		MIXED MechanosensitiveAβfibersThresholds BY Condition Time<br>/CRITERIA=CIN(95) MXITER(100) MXSTEP(10) SCORING(1) SINGULAR(0.000000000001) HCONVERGE(0, ABSOLUTE) LCONVERGE(0, ABSOLUTE) PCONVERGE(0.000001, ABSOLUTE)<br>/FIXED=Condition Time Condition*Time | SSTYPE(3)<br>/METHOD=REML<br>/PRINT=CPS CORB COVB DESCRIPTIVES G SOLUTION TESTCOV<br>/EMMEANS=TABLES(Condition) COMPARE ADJ(BONFERRONI)<br>/EMMEANS=TABLES(Time) COMPARE ADJ(BONFERRONI)<br>/EMMEANS=TABLES(Condition*Time) .
Ressources	Temps de processeur	00:00:00,03
	Temps écoulé	00:00:00,03

Récapitulatif de traitement des observations

		Effectif	Pourcentage marginal
Condition	Sham	28	50,9%
	taVNS	27	49,1%
Time	T0	29	52,7%
	T1	26	47,3%

Valide	55	100,0%
Exclues	5	
Total	60	

Statistiques descriptives

Mechanosensitive Aβ-fibers Thresholds

Condition	Time	Effectif	Moyenne	Ecart type	Coefficient de variation
Sham	T0	15	,00480167	,009474470	197,3%
	T1	13	,00356500	,001789538	50,2%
	Total	28	,00422750	,006954344	164,5%
taVNS	T0	14	,00418214	,006703231	160,3%
	T1	13	,00617115	,005798032	94,0%
	Total	27	,00513981	,006245634	121,5%
Total	T0	29	,00450259	,008114428	180,2%
	T1	26	,00486808	,004409007	90,6%
	Total	55	,00467536	,006570762	140,5%

Dimension du modèle[a]

		Nombre de niveaux	Nombre de paramètres
Effets fixes	Constante	1	1
	Condition	2	1
	Time	2	1
	Condition * Time	4	1
Résidu			1
Total		9	5

a. Variable dépendante : Mechanosensitive Aβ-fibers Thresholds.

Critères d'information[a]

Log de vraisemblance restreint -2	-355,517
Critère d'information d'Akaike (AIC)	-353,517
Critère de Hurvich et Tsai (AICC)	-353,435
Critère de Bozdogan (CAIC)	-350,585
Critère bayésien de Schwartz (BIC)	-351,585

Les critères d'informations sont présentés en plus petit, disposant d'un meilleur format.[a]

a. Variable dépendante : Mechanosensitive Aβ-fibers Thresholds.

Effets fixes

Tests des effets fixes de type III[a]

Source	Ddl du numérateur	Ddl du dénominateur	F	Sig.
Constante	1	51	26,819	,000
Condition	1	51	,302	,585
Time	1	51	,043	,836
Condition * Time	1	51	,796	,376

a. Variable dépendante : Mechanosensitive Aβ-fibers Thresholds.

Estimations des effets fixes[a]

Paramètre	Estimation	Erreur standard	ddl	t	Sig.
Constante	,006171	,001856	51	3,326	,002
[Condition=Sham]	-,002606	,002624	51	-,993	,325
[Condition=taVNS]	0[b]	0	.	.	.
[Time=T0]	-,001989	,002577	51	-,772	,444
[Time=T1]	0[b]	0	.	.	.
[Condition=Sham] * [Time=T0]	,003226	,003615	51	,892	,376
[Condition=Sham] * [Time=T1]	0[b]	0	.	.	.
[Condition=taVNS] * [Time=T0]	0[b]	0	.	.	.
[Condition=taVNS] * [Time=T1]	0[b]	0	.	.	.

Estimations des effets fixes[a]

Paramètre	Intervalle de confiance à 95 %	
	Borne inférieure	Borne supérieure
Constante	,002446	,009896
[Condition=Sham]	-,007874	,002662
[Condition=taVNS]	.	.
[Time=T0]	-,007162	,003184
[Time=T1]	.	.
[Condition=Sham] * [Time=T0]	-,004031	,010483

[Condition=Sham] * [Time=T1]	.	.
[Condition=taVNS] * [Time=T0]	.	.
[Condition=taVNS] * [Time=T1]	.	.

a. Variable dépendante : Mechanosensitive Aβ-fibers Thresholds.

b. Ce paramètre est défini sur 0, car il est redondant.

Matrice de corrélation pour les estimations des effets fixes[a]

Paramètre	Constante	[Condition=Sham]	[Condition=taVNS]	[Time=T0]	[Time=T1]
Constante	1	-,707	.[b]	-,720	.[b]
[Condition=Sham]	-,707	1	.[b]	,509	.[b]
[Condition=taVNS]	.[b]	.[b]	.[b]	.[b]	.[b]
[Time=T0]	-,720	,509	.[b]	1	.[b]
[Time=T1]	.[b]	.[b]	.[b]	.[b]	.[b]
[Condition=Sham] * [Time=T0]	,513	-,726	.[b]	-,713	.[b]
[Condition=Sham] * [Time=T1]	.[b]	.[b]	.[b]	.[b]	.[b]
[Condition=taVNS] * [Time=T0]	.[b]	.[b]	.[b]	.[b]	.[b]
[Condition=taVNS] * [Time=T1]	.[b]	.[b]	.[b]	.[b]	.[b]

Matrice de corrélation pour les estimations des effets fixes[a]

Paramètre	[Condition=Sham] * [Time=T0]	[Condition=Sham] * [Time=T1]	[Condition=taVNS] * [Time=T0]	[Condition=taVNS] * [Time=T1]
Constante	,513	.[b]	.[b]	.[b]
[Condition=Sham]	-,726	.[b]	.[b]	.[b]
[Condition=taVNS]	.[b]	.[b]	.[b]	.[b]
[Time=T0]	-,713	.[b]	.[b]	.[b]
[Time=T1]	.[b]	.[b]	.[b]	.[b]
[Condition=Sham] * [Time=T0]	1	.[b]	.[b]	.[b]
[Condition=Sham] * [Time=T1]	.[b]	.[b]	.[b]	.[b]
[Condition=taVNS] * [Time=T0]	.[b]	.[b]	.[b]	.[b]
[Condition=taVNS] * [Time=T1]	.[b]	.[b]	.[b]	.[b]

a. Variable dépendante : Mechanosensitive Aβ-fibers Thresholds.

b. La corrélation est manquante par défaut, car elle est associée à un paramètre redondant.

Matrice de covariance pour les estimations des effets fixes[a]

Paramètre	Constante	[Condition=Sham]	[Condition=taVNS]	[Time=T0]
Constante	3,442966E-6	-3,442966E-6	0[b]	-3,442966E-6
[Condition=Sham]	-3,442966E-6	6,885932E-6	0[b]	3,442966E-6
[Condition=taVNS]	0[b]	0[b]	0[b]	0[b]
[Time=T0]	-3,442966E-6	3,442966E-6	0[b]	6,640006E-6
[Time=T1]	0[b]	0[b]	0[b]	0[b]
[Condition=Sham] * [Time=T0]	3,442966E-6	-6,885932E-6	0[b]	-6,640006E-6
[Condition=Sham] * [Time=T1]	0[b]	0[b]	0[b]	0[b]
[Condition=taVNS] * [Time=T0]	0[b]	0[b]	0[b]	0[b]
[Condition=taVNS] * [Time=T1]	0[b]	0[b]	0[b]	0[b]

Matrice de covariance pour les estimations des effets fixes[a]

Paramètre	[Time=T1]	[Condition=Sham] * [Time=T0]	[Condition=Sham] * [Time=T1]	[Condition=taVNS] * [Time=T0]
Constante	0[b]	3,442966E-6	0[b]	0[b]
[Condition=Sham]	0[b]	-6,885932E-6	0[b]	0[b]
[Condition=taVNS]	0[b]	0[b]	0[b]	0[b]
[Time=T0]	0[b]	-6,640006E-6	0[b]	0[b]
[Time=T1]	0[b]	0[b]	0[b]	0[b]
[Condition=Sham] * [Time=T0]	0[b]	1,306688E-5	0[b]	0[b]
[Condition=Sham] * [Time=T1]	0[b]	0[b]	0[b]	0[b]
[Condition=taVNS] * [Time=T0]	0[b]	0[b]	0[b]	0[b]
[Condition=taVNS] * [Time=T1]	0[b]	0[b]	0[b]	0[b]

Matrice de covariance pour les estimations des effets fixes[a]

Paramètre	[Condition=taVNS] * [Time=T1]
Constante	0[b]
[Condition=Sham]	0[b]
[Condition=taVNS]	0[b]
[Time=T0]	0[b]
[Time=T1]	0[b]
[Condition=Sham] * [Time=T0]	0[b]
[Condition=Sham] * [Time=T1]	0[b]
[Condition=taVNS] * [Time=T0]	0[b]
[Condition=taVNS] * [Time=T1]	0[b]

a. Variable dépendante : Mechanosensitive Aβ-fibers Thresholds.

b. La covariance est définie sur 0, car elle est associée à un paramètre redondant.

Paramètres de covariance

Estimations des paramètres de covariance[a]

Paramètre	Estimation	Erreur standard	Z de Wald	Sig.	Intervalle de confiance à 95 %	
					Borne inférieure	Borne supérieure
Résidu	4,475856E-5	8,863516E-6	5,050	,000	3,036079E-5	6,598407E-5

a. Variable dépendante : Mechanosensitive Aβ-fibers Thresholds.

Matrice de corrélation pour les estimations des paramètres de covariance[a]

Paramètre	Résidu
Résidu	1

a. Variable dépendante : Mechanosensitive Aβ-fibers Thresholds.

Matrice de covariance pour les estimations des paramètres de covariance[a]

Paramètre	Résidu
Résidu	7,856191E-11

a. Variable dépendante : Mechanosensitive Aβ-fibers Thresholds.

Moyenne marginale estimée

1. Condition

Estimations[a]

Condition	Moyenne	Erreur standard	ddl	Intervalle de confiance à 95 %

				Borne inférieure	Borne supérieure
Sham	,004	,001	51	,002	,007
taVNS	,005	,001	51	,003	,008

a. Variable dépendante : Mechanosensitive Aβ-fibers Thresholds.

Comparaisons appariées[a]

(I) Condition	(J) Condition	Différence moyenne (I-J)	Erreur standard	ddl	Sig.[b]
Sham	taVNS	-,001	,002	51	,585
taVNS	Sham	,001	,002	51	,585

Comparaisons appariées[a]

		Intervalle de confiance à 95 % pour la différence[b]	
(I) Condition	(J) Condition	Borne inférieure	Borne supérieure
Sham	taVNS	-,005	,003
taVNS	Sham	-,003	,005

Basées sur les moyennes marginales estimées[a]

a. Variable dépendante : Mechanosensitive Aβ-fibers Thresholds.

b. Ajustement pour les comparaisons multiples : Bonferroni.

Tests univariés[a]

Ddl du numérateur	Ddl du dénominateur	F	Sig.
1	51	,302	,585

Le test de F permet de tester l'effet de Condition. Il s'appuie sur les comparaisons appariées (indépendantes) linéaires parmi les moyennes marginales estimées.[a]

a. Variable dépendante : Mechanosensitive Aβ-fibers Thresholds.

2. Time

Estimations[a]

				Intervalle de confiance à 95 %	
Time	Moyenne	Erreur standard	ddl	Borne inférieure	Borne supérieure
T0	,004	,001	51	,002	,007
T1	,005	,001	51	,002	,008

a. Variable dépendante : Mechanosensitive Aβ-fibers Thresholds.

Comparaisons appariées[a]

(I) Time	(J) Time	Différence moyenne (I-J)	Erreur standard	ddl	Sig.[b]	Intervalle de confiance à 95 % pour la différence[b] Borne inférieure
T0	T1	,000	,002	51	,836	-,004
T1	T0	,000	,002	51	,836	-,003

Comparaisons appariées[a]

(I) Time	(J) Time	Intervalle de confiance à 95 % pour la différence Borne supérieure
T0	T1	,003
T1	T0	,004

Basées sur les moyennes marginales estimées[a]

a. Variable dépendante : Mechanosensitive Aβ-fibers Thresholds.

b. Ajustement pour les comparaisons multiples : Bonferroni.

Tests univariés[a]

Ddl du numérateur	Ddl du dénominateur	F	Sig.
1	51	,043	,836

Le test de F permet de tester l'effet de Time. Il s'appuie sur les comparaisons appariées (indépendantes) linéaires parmi les moyennes marginales estimées.[a]

a. Variable dépendante : Mechanosensitive Aβ-fibers Thresholds.

3. Condition * Time[a]

Condition	Time	Moyenne	Erreur standard	ddl	Intervalle de confiance à 95 % Borne inférieure	Intervalle de confiance à 95 % Borne supérieure
Sham	T0	,005	,002	51	,001	,008
	T1	,004	,002	51	,000	,007
taVNS	T0	,004	,002	51	,001	,008
	T1	,006	,002	51	,002	,010

a. Variable dépendante : Mechanosensitive Aβ-fibers Thresholds.

2.4. Laser Intensity.

```
MIXED LaserIntensity BY Condition Phase
  /CRITERIA=CIN(95) MXITER(100) MXSTEP(10) SCORING(1)
SINGULAR(0.000000000001) HCONVERGE(0,
    ABSOLUTE) LCONVERGE(0, ABSOLUTE) PCONVERGE(0.000001, ABSOLUTE)
  /FIXED=Condition Phase Condition*Phase | SSTYPE(3)
  /METHOD=REML
  /PRINT=CPS CORB COVB DESCRIPTIVES G  SOLUTION TESTCOV
  /EMMEANS=TABLES(OVERALL)
  /EMMEANS=TABLES(Condition) COMPARE ADJ(BONFERRONI)
  /EMMEANS=TABLES(Phase) COMPARE ADJ(BONFERRONI)
  /EMMEANS=TABLES(Condition*Phase) .
```

Remarques

Sortie obtenue		04-MAY-2021 13:55:53
Commentaires		
Entrée	Jeu de données actif	Jeu_de_données2
	Filtre	<sans>
	Pondération	<sans>
	Fichier scindé	<sans>
	N de lignes dans le fichier de travail	60
Gestion des valeurs manquantes	Définition de la valeur manquante	Les valeurs manquantes définies par l'utilisateur sont traitées comme étant manquantes.
	Observations utilisées	Les statistiques sont basées sur toutes les observations comportant des données valides pour toutes les variables du modèle.

Syntaxe		MIXED LaserIntensity BY Condition Phase /CRITERIA=CIN(95) MXITER(100) MXSTEP(10) SCORING(1) SINGULAR(0.000000000001) HCONVERGE(0, ABSOLUTE) LCONVERGE(0, ABSOLUTE) PCONVERGE(0.000001, ABSOLUTE) /FIXED=Condition Phase Condition*Phase | SSTYPE(3) /METHOD=REML /PRINT=CPS CORB COVB DESCRIPTIVES G SOLUTION TESTCOV /EMMEANS=TABLES(OVERALL) /EMMEANS=TABLES(Condition) COMPARE ADJ(BONFERRONI) /EMMEANS=TABLES(Phase) COMPARE ADJ(BONFERRONI) /EMMEANS=TABLES(Condition*Phase) .
Ressources	Temps de processeur	00:00:00,02
	Temps écoulé	00:00:00,02

Récapitulatif de traitement des observations

		Effectif	Pourcentage marginal
Condition	Sham	30	50,0%

	taVNS	30	50,0%
Phase	OFF	30	50,0%
	ON	30	50,0%
Valide		60	100,0%
Exclues		0	
Total		60	

Statistiques descriptives

Laser Intensity

Condition	Phase	Effectif	Moyenne	Ecart type	Coefficient de variation
Sham	OFF	15	5,009446443337920	1,642149410434609	32,8%
	ON	15	4,980647494374438	1,568691229540226	31,5%
	Total	30	4,995046968856181	1,577978588897406	31,6%
taVNS	OFF	15	4,824184666553781	2,161368955183174	44,8%
	ON	15	4,867127433328342	2,099169542273677	43,1%
	Total	30	4,845656049941062	2,093555217361327	43,2%
Total	OFF	30	4,916815554945850	1,888365473623391	38,4%
	ON	30	4,923887463851391	1,821697959405777	37,0%
	Total	60	4,920351509398620	1,839544301895055	37,4%

Dimension du modèle[a]

		Nombre de niveaux	Nombre de paramètres
Effets fixes	Constante	1	1
	Condition	2	1
	Phase	2	1
	Condition * Phase	4	1
Résidu			1
Total		9	5

a. Variable dépendante : Laser Intensity.

Critères d'information[a]

Log de vraisemblance restreint -2	240,842
Critère d'information d'Akaike (AIC)	242,842
Critère de Hurvich et Tsai (AICC)	242,916
Critère de Bozdogan (CAIC)	245,867
Critère bayésien de Schwartz (BIC)	244,867

Les critères d'informations sont présentés en plus petit, disposant d'un meilleur format.[a]

a. Variable dépendante : Laser Intensity.

Effets fixes

Tests des effets fixes de type III[a]

Source	Ddl du numérateur	Ddl du dénominateur	F	Sig.
Constante	1	56	408,161	,000
Condition	1	56	,094	,760
Phase	1	56	,000	,988
Condition * Phase	1	56	,005	,942

a. Variable dépendante : Laser Intensity.

Estimations des effets fixes[a]

Paramètre	Estimation	Erreur standard	ddl	t	Sig.
Constante	4,867127	,487091	56	9,992	,000
[Condition=Sham]	,113520	,688851	56	,165	,870
[Condition=taVNS]	0[b]	0	.	.	.
[Phase=OFF]	-,042943	,688851	56	-,062	,951
[Phase=ON]	0[b]	0	.	.	.
[Condition=Sham] * [Phase=OFF]	,071742	,974183	56	,074	,942
[Condition=Sham] * [Phase=ON]	0[b]	0	.	.	.

[Condition=taVNS] * [Phase=OFF]	0[b]	0	.	.	.
[Condition=taVNS] * [Phase=ON]	0[b]	0	.	.	.

Estimations des effets fixes[a]

Paramètre	Intervalle de confiance à 95 % Borne inférieure	Borne supérieure
Constante	3,891366	5,842889
[Condition=Sham]	-1,266415	1,493455
[Condition=taVNS]	.	.
[Phase=OFF]	-1,422877	1,336992
[Phase=ON]	.	.
[Condition=Sham] * [Phase=OFF]	-1,879780	2,023264
[Condition=Sham] * [Phase=ON]	.	.
[Condition=taVNS] * [Phase=OFF]	.	.
[Condition=taVNS] * [Phase=ON]	.	.

a. Variable dépendante : Laser Intensity.

b. Ce paramètre est défini sur 0, car il est redondant.

Matrice de corrélation pour les estimations des effets fixes[a]

Paramètre	Constante	[Condition=Sham]	[Condition=taVNS]	[Phase=OFF]
Constante	1	-,707	.[b]	-,707
[Condition=Sham]	-,707	1	.[b]	,500
[Condition=taVNS]	.[b]	.[b]	.[b]	.[b]
[Phase=OFF]	-,707	,500	.[b]	1
[Phase=ON]	.[b]	.[b]	.[b]	.[b]
[Condition=Sham] * [Phase=OFF]	,500	-,707	.[b]	-,707
[Condition=Sham] * [Phase=ON]	.[b]	.[b]	.[b]	.[b]
[Condition=taVNS] * [Phase=OFF]	.[b]	.[b]	.[b]	.[b]
[Condition=taVNS] * [Phase=ON]	.[b]	.[b]	.[b]	.[b]

Matrice de corrélation pour les estimations des effets fixes[a]

Paramètre	[Phase=ON]	[Condition=Sham] * [Phase=OFF]	[Condition=Sham] * [Phase=ON]	[Condition=taVNS] * [Phase=OFF]
Constante	.[b]	,500	.[b]	.[b]

[Condition=Sham]	.[b]	-,707	.[b]	.[b]
[Condition=taVNS]	.[b]	.[b]	.[b]	.[b]
[Phase=OFF]	.[b]	-,707	.[b]	.[b]
[Phase=ON]	.[b]	.[b]	.[b]	.[b]
[Condition=Sham] * [Phase=OFF]	.[b]	1	.[b]	.[b]
[Condition=Sham] * [Phase=ON]	.[b]	.[b]	.[b]	.[b]
[Condition=taVNS] * [Phase=OFF]	.[b]	.[b]	.[b]	.[b]
[Condition=taVNS] * [Phase=ON]	.[b]	.[b]	.[b]	.[b]

Matrice de corrélation pour les estimations des effets fixes[a]

Paramètre	[Condition=taVNS] * [Phase=ON]
Constante	.[b]
[Condition=Sham]	.[b]
[Condition=taVNS]	.[b]
[Phase=OFF]	.[b]
[Phase=ON]	.[b]
[Condition=Sham] * [Phase=OFF]	.[b]
[Condition=Sham] * [Phase=ON]	.[b]
[Condition=taVNS] * [Phase=OFF]	.[b]
[Condition=taVNS] * [Phase=ON]	.[b]

a. Variable dépendante : Laser Intensity.

b. La corrélation est manquante par défaut, car elle est associée à un paramètre redondant.

Matrice de covariance pour les estimations des effets fixes[a]

Paramètre	Constante	[Condition=Sham]	[Condition=taVNS]	[Phase=OFF]
Constante	,237258	-,237258	0[b]	-,237258
[Condition=Sham]	-,237258	,474516	0[b]	,237258
[Condition=taVNS]	0[b]	0[b]	0[b]	0[b]
[Phase=OFF]	-,237258	,237258	0[b]	,474516
[Phase=ON]	0[b]	0[b]	0[b]	0[b]
[Condition=Sham] * [Phase=OFF]	,237258	-,474516	0[b]	-,474516
[Condition=Sham] * [Phase=ON]	0[b]	0[b]	0[b]	0[b]

[Condition=taVNS] * [Phase=OFF]	0[b]	0[b]	0[b]	0[b]
[Condition=taVNS] * [Phase=ON]	0[b]	0[b]	0[b]	0[b]

Matrice de covariance pour les estimations des effets fixes[a]

Paramètre	[Phase=ON]	[Condition=Sham] * [Phase=OFF]	[Condition=Sham] * [Phase=ON]	[Condition=taVNS] * [Phase=OFF]
Constante	0[b]	,237258	0[b]	0[b]
[Condition=Sham]	0[b]	-,474516	0[b]	0[b]
[Condition=taVNS]	0[b]	0[b]	0[b]	0[b]
[Phase=OFF]	0[b]	-,474516	0[b]	0[b]
[Phase=ON]	0[b]	0[b]	0[b]	0[b]
[Condition=Sham] * [Phase=OFF]	0[b]	,949032	0[b]	0[b]
[Condition=Sham] * [Phase=ON]	0[b]	0[b]	0[b]	0[b]
[Condition=taVNS] * [Phase=OFF]	0[b]	0[b]	0[b]	0[b]
[Condition=taVNS] * [Phase=ON]	0[b]	0[b]	0[b]	0[b]

Matrice de covariance pour les estimations des effets fixes[a]

Paramètre	[Condition=taVNS] * [Phase=ON]
Constante	0[b]
[Condition=Sham]	0[b]
[Condition=taVNS]	0[b]
[Phase=OFF]	0[b]
[Phase=ON]	0[b]
[Condition=Sham] * [Phase=OFF]	0[b]
[Condition=Sham] * [Phase=ON]	0[b]
[Condition=taVNS] * [Phase=OFF]	0[b]
[Condition=taVNS] * [Phase=ON]	0[b]

a. Variable dépendante : Laser Intensity.

b. La covariance est définie sur 0, car elle est associée à un paramètre redondant.

Paramètres de covariance

Estimations des paramètres de covariance[a]

Paramètre	Estimation	Erreur standard	Z de Wald	Sig.	Intervalle de confiance à 95 % Borne inférieure	Borne supérieure
Résidu	3,558869	,672563	5,292	,000	2,457254	5,154351

a. Variable dépendante : Laser Intensity.

Matrice de corrélation pour les estimations des paramètres de covariance[a]

Paramètre	Résidu
Résidu	1

a. Variable dépendante : Laser Intensity.

Matrice de covariance pour les estimations des paramètres de covariance[a]

Paramètre	Résidu
Résidu	,452341

a. Variable dépendante : Laser Intensity.

Moyenne marginale estimée

1. Grand Mean[a]

Moyenne	Erreur standard	ddl	Intervalle de confiance à 95 % Borne inférieure	Borne supérieure
4,920	,244	56	4,432	5,408

a. Variable dépendante : Laser Intensity.

2. Condition

Estimations[a]

Condition	Moyenne	Erreur standard	ddl	Intervalle de confiance à 95 % Borne inférieure	Borne supérieure
Sham	4,995	,344	56	4,305	5,685
taVNS	4,846	,344	56	4,156	5,536

a. Variable dépendante : Laser Intensity.

Comparaisons appariées[a]

(I) Condition	(J) Condition	Différence moyenne (I-J)	Erreur standard	ddl	Sig.[b]
Sham	taVNS	,149	,487	56	,760
taVNS	Sham	-,149	,487	56	,760

Comparaisons appariées[a]

(I) Condition	(J) Condition	Intervalle de confiance à 95 % pour la différence[b] Borne inférieure	Borne supérieure
Sham	taVNS	-,826	1,125
taVNS	Sham	-1,125	,826

Basées sur les moyennes marginales estimées[a]

a. Variable dépendante : Laser Intensity.

b. Ajustement pour les comparaisons multiples : Bonferroni.

Tests univariés[a]

Ddl du numérateur	Ddl du dénominateur	F	Sig.
1	56	,094	,760

Le test de F permet de tester l'effet de Condition. Il s'appuie sur les comparaisons appariées (indépendantes) linéaires parmi les moyennes marginales estimées.[a]

a. Variable dépendante : Laser Intensity.

3. Phase

Estimations[a]

Phase	Moyenne	Erreur standard	ddl	Intervalle de confiance à 95 % Borne inférieure	Borne supérieure
OFF	4,917	,344	56	4,227	5,607
ON	4,924	,344	56	4,234	5,614

a. Variable dépendante : Laser Intensity.

Comparaisons appariées[a]

(I) Phase	(J) Phase	Différence moyenne (I-J)	Erreur standard	ddl	Sig.[b]	Intervalle de confiance à 95 % pour la différence[b] Borne inférieure
OFF	ON	-,007	,487	56	,988	-,983
ON	OFF	,007	,487	56	,988	-,969

Comparaisons appariées[a]

(I) Phase	(J) Phase	Intervalle de confiance à 95 % pour la différence Borne supérieure
OFF	ON	,969
ON	OFF	,983

Basées sur les moyennes marginales estimées[a]

a. Variable dépendante : Laser Intensity.

b. Ajustement pour les comparaisons multiples : Bonferroni.

Tests univariés[a]

Ddl du numérateur	Ddl du dénominateur	F	Sig.
1	56	,000	,988

Le test de F permet de tester l'effet de Phase. Il s'appuie sur les comparaisons appariées (indépendantes) linéaires parmi les moyennes marginales estimées.[a]

a. Variable dépendante : Laser Intensity.

4. Condition * Phase[a]

Condition	Phase	Moyenne	Erreur standard	ddl	Intervalle de confiance à 95 % Borne inférieure	Intervalle de confiance à 95 % Borne supérieure
Sham	OFF	5,009	,487	56	4,034	5,985
	ON	4,981	,487	56	4,005	5,956
taVNS	OFF	4,824	,487	56	3,848	5,800
	ON	4,867	,487	56	3,891	5,843

a. Variable dépendante : Laser Intensity.

2.4. Vibrotactile Intensity.

```
MIXED VibrotactileIntensity BY Condition Phase
  /CRITERIA=CIN(95) MXITER(100) MXSTEP(10) SCORING(1)
SINGULAR(0.000000000001) HCONVERGE(0,
    ABSOLUTE) LCONVERGE(0, ABSOLUTE) PCONVERGE(0.000001, ABSOLUTE)
  /FIXED=Condition Phase Condition*Phase | SSTYPE(3)
  /METHOD=REML
  /PRINT=CPS CORB COVB DESCRIPTIVES G  SOLUTION TESTCOV
  /EMMEANS=TABLES(OVERALL)
  /EMMEANS=TABLES(Condition) COMPARE ADJ(BONFERRONI)
  /EMMEANS=TABLES(Phase) COMPARE ADJ(BONFERRONI)
  /EMMEANS=TABLES(Condition*Phase) .
```

Remarques

Sortie obtenue		04-MAY-2021 13:56:49
Commentaires		
Entrée	Jeu de données actif	Jeu_de_données2
	Filtre	<sans>
	Pondération	<sans>
	Fichier scindé	<sans>
	N de lignes dans le fichier de travail	60
Gestion des valeurs manquantes	Définition de la valeur manquante	Les valeurs manquantes définies par l'utilisateur sont traitées comme étant manquantes.
	Observations utilisées	Les statistiques sont basées sur toutes les observations comportant des données valides pour toutes les variables du modèle.

Syntaxe		MIXED VibrotactileIntensity BY Condition Phase /CRITERIA=CIN(95) MXITER(100) MXSTEP(10) SCORING(1) SINGULAR(0.000000000001) HCONVERGE(0, ABSOLUTE) LCONVERGE(0, ABSOLUTE) PCONVERGE(0.000001, ABSOLUTE) /FIXED=Condition Phase Condition*Phase | SSTYPE(3) /METHOD=REML /PRINT=CPS CORB COVB DESCRIPTIVES G SOLUTION TESTCOV /EMMEANS=TABLES(OVERALL) /EMMEANS=TABLES(Condition) COMPARE ADJ(BONFERRONI) /EMMEANS=TABLES(Phase) COMPARE ADJ(BONFERRONI) /EMMEANS=TABLES(Condition*Phase) .
Ressources	Temps de processeur	00:00:00,02
	Temps écoulé	00:00:00,02

Récapitulatif de traitement des observations

		Effectif	Pourcentage marginal
Condition	Sham	30	50,0%

	taVNS	30	50,0%
Phase	OFF	30	50,0%
	ON	30	50,0%
Valide		60	100,0%
Exclues		0	
Total		60	

Statistiques descriptives

Vibrotactile Intensity

Condition	Phase	Effectif	Moyenne	Ecart type	Coefficient de variation
Sham	OFF	15	4,532198285505938	2,009976571306574	44,3%
	ON	15	4,458412380245497	2,036997503668679	45,7%
	Total	30	4,495305332875717	1,988691631569532	44,2%
taVNS	OFF	15	4,855873775757067	1,939327817010551	39,9%
	ON	15	4,748953320447128	1,946352608144864	41,0%
	Total	30	4,802413548102098	1,909826421767106	39,8%
Total	OFF	30	4,694036030631501	1,947586645045882	41,5%
	ON	30	4,603682850346313	1,963109492941954	42,6%
	Total	60	4,648859440488907	1,939256984542746	41,7%

Dimension du modèle[a]

		Nombre de niveaux	Nombre de paramètres
Effets fixes	Constante	1	1
	Condition	2	1
	Phase	2	1
	Condition * Phase	4	1
Résidu			1
Total		9	5

a. Variable dépendante : Vibrotactile Intensity.

Critères d'information[a]

Log de vraisemblance restreint -2	246,464
Critère d'information d'Akaike (AIC)	248,464
Critère de Hurvich et Tsai (AICC)	248,538
Critère de Bozdogan (CAIC)	251,489
Critère bayésien de Schwartz (BIC)	250,489

Les critères d'informations sont présentés en plus petit, disposant d'un meilleur format.[a]

a. Variable dépendante : Vibrotactile Intensity.

Effets fixes

Tests des effets fixes de type III[a]

Source	Ddl du numérateur	Ddl du dénominateur	F	Sig.
Constante	1	56	329,562	,000
Condition	1	56	,360	,551
Phase	1	56	,031	,861
Condition * Phase	1	56	,001	,974

a. Variable dépendante : Vibrotactile Intensity.

Estimations des effets fixes[a]

Paramètre	Estimation	Erreur standard	ddl	t	Sig.
Constante	4,748953	,512163	56	9,272	,000
[Condition=Sham]	-,290541	,724308	56	-,401	,690
[Condition=taVNS]	0[b]	0	.	.	.
[Phase=OFF]	,106920	,724308	56	,148	,883
[Phase=ON]	0[b]	0	.	.	.
[Condition=Sham] * [Phase=OFF]	-,033135	1,024326	56	-,032	,974
[Condition=Sham] * [Phase=ON]	0[b]	0	.	.	.

[Condition=taVNS] * [Phase=OFF]	0[b]	0	.	.	.
[Condition=taVNS] * [Phase=ON]	0[b]	0	.	.	.

Estimations des effets fixes[a]

Paramètre	Intervalle de confiance à 95 % Borne inférieure	Borne supérieure
Constante	3,722968	5,774939
[Condition=Sham]	-1,741503	1,160422
[Condition=taVNS]	.	.
[Phase=OFF]	-1,344042	1,557883
[Phase=ON]	.	.
[Condition=Sham] * [Phase=OFF]	-2,085105	2,018836
[Condition=Sham] * [Phase=ON]	.	.
[Condition=taVNS] * [Phase=OFF]	.	.
[Condition=taVNS] * [Phase=ON]	.	.

a. Variable dépendante : Vibrotactile Intensity.

b. Ce paramètre est défini sur 0, car il est redondant.

Matrice de corrélation pour les estimations des effets fixes[a]

Paramètre	Constante	[Condition=Sham]	[Condition=taVNS]	[Phase=OFF]
Constante	1	-,707	.[b]	-,707
[Condition=Sham]	-,707	1	.[b]	,500
[Condition=taVNS]	.[b]	.[b]	.[b]	.[b]
[Phase=OFF]	-,707	,500	.[b]	1
[Phase=ON]	.[b]	.[b]	.[b]	.[b]
[Condition=Sham] * [Phase=OFF]	,500	-,707	.[b]	-,707
[Condition=Sham] * [Phase=ON]	.[b]	.[b]	.[b]	.[b]
[Condition=taVNS] * [Phase=OFF]	.[b]	.[b]	.[b]	.[b]
[Condition=taVNS] * [Phase=ON]	.[b]	.[b]	.[b]	.[b]

Matrice de corrélation pour les estimations des effets fixes[a]

Paramètre	[Phase=ON]	[Condition=Sham] * [Phase=OFF]	[Condition=Sham] * [Phase=ON]	[Condition=taVNS] * [Phase=OFF]

Constante	.[b]	,500	.[b]	.[b]
[Condition=Sham]	.[b]	-,707	.[b]	.[b]
[Condition=taVNS]	.[b]	.[b]	.[b]	.[b]
[Phase=OFF]	.[b]	-,707	.[b]	.[b]
[Phase=ON]	.[b]	.[b]	.[b]	.[b]
[Condition=Sham] * [Phase=OFF]	.[b]	1	.[b]	.[b]
[Condition=Sham] * [Phase=ON]	.[b]	.[b]	.[b]	.[b]
[Condition=taVNS] * [Phase=OFF]	.[b]	.[b]	.[b]	.[b]
[Condition=taVNS] * [Phase=ON]	.[b]	.[b]	.[b]	.[b]

Matrice de corrélation pour les estimations des effets fixes[a]

Paramètre	[Condition=taVNS] * [Phase=ON]
Constante	.[b]
[Condition=Sham]	.[b]
[Condition=taVNS]	.[b]
[Phase=OFF]	.[b]
[Phase=ON]	.[b]
[Condition=Sham] * [Phase=OFF]	.[b]
[Condition=Sham] * [Phase=ON]	.[b]
[Condition=taVNS] * [Phase=OFF]	.[b]
[Condition=taVNS] * [Phase=ON]	.[b]

a. Variable dépendante : Vibrotactile Intensity.

b. La corrélation est manquante par défaut, car elle est associée à un paramètre redondant.

Matrice de covariance pour les estimations des effets fixes[a]

Paramètre	Constante	[Condition=Sham]	[Condition=taVNS]	[Phase=OFF]
Constante	,262311	-,262311	0[b]	-,262311
[Condition=Sham]	-,262311	,524622	0[b]	,262311
[Condition=taVNS]	0[b]	0[b]	0[b]	0[b]
[Phase=OFF]	-,262311	,262311	0[b]	,524622
[Phase=ON]	0[b]	0[b]	0[b]	0[b]
[Condition=Sham] * [Phase=OFF]	,262311	-,524622	0[b]	-,524622
[Condition=Sham] * [Phase=ON]	0[b]	0[b]	0[b]	0[b]

[Condition=taVNS] * [Phase=OFF]	0[b]	0[b]	0[b]	0[b]
[Condition=taVNS] * [Phase=ON]	0[b]	0[b]	0[b]	0[b]

Matrice de covariance pour les estimations des effets fixes[a]

Paramètre	[Phase=ON]	[Condition=Sham] * [Phase=OFF]	[Condition=Sham] * [Phase=ON]	[Condition=taVNS] * [Phase=OFF]
Constante	0[b]	,262311	0[b]	0[b]
[Condition=Sham]	0[b]	-,524622	0[b]	0[b]
[Condition=taVNS]	0[b]	0[b]	0[b]	0[b]
[Phase=OFF]	0[b]	-,524622	0[b]	0[b]
[Phase=ON]	0[b]	0[b]	0[b]	0[b]
[Condition=Sham] * [Phase=OFF]	0[b]	1,049243	0[b]	0[b]
[Condition=Sham] * [Phase=ON]	0[b]	0[b]	0[b]	0[b]
[Condition=taVNS] * [Phase=OFF]	0[b]	0[b]	0[b]	0[b]
[Condition=taVNS] * [Phase=ON]	0[b]	0[b]	0[b]	0[b]

Matrice de covariance pour les estimations des effets fixes[a]

Paramètre	[Condition=taVNS] * [Phase=ON]
Constante	0[b]
[Condition=Sham]	0[b]
[Condition=taVNS]	0[b]
[Phase=OFF]	0[b]
[Phase=ON]	0[b]
[Condition=Sham] * [Phase=OFF]	0[b]
[Condition=Sham] * [Phase=ON]	0[b]
[Condition=taVNS] * [Phase=OFF]	0[b]
[Condition=taVNS] * [Phase=ON]	0[b]

a. Variable dépendante : Vibrotactile Intensity.

b. La covariance est définie sur 0, car elle est associée à un paramètre redondant.

Paramètres de covariance

Estimations des paramètres de covariance[a]

Paramètre	Estimation	Erreur standard	Z de Wald	Sig.	Intervalle de confiance à 95 %	
					Borne inférieure	Borne supérieure

Résidu	3,934661	,743581	5,292	,000	2,716723	5,698615

a. Variable dépendante : Vibrotactile Intensity.

Matrice de corrélation pour les estimations des paramètres de covariance[a]

Paramètre	Résidu
Résidu	1

a. Variable dépendante : Vibrotactile Intensity.

Matrice de covariance pour les estimations des paramètres de covariance[a]

Paramètre	Résidu
Résidu	,552913

a. Variable dépendante : Vibrotactile Intensity.

Moyenne marginale estimée

1. Grand Mean[a]

Moyenne	Erreur standard	ddl	Intervalle de confiance à 95 % Borne inférieure	Borne supérieure
4,649	,256	56	4,136	5,162

a. Variable dépendante : Vibrotactile Intensity.

2. Condition

Estimations[a]

Condition	Moyenne	Erreur standard	ddl	Intervalle de confiance à 95 % Borne inférieure	Borne supérieure
Sham	4,495	,362	56	3,770	5,221

taVNS	4,802	,362	56	4,077	5,528

a. Variable dépendante : Vibrotactile Intensity.

Comparaisons appariées[a]

(I) Condition	(J) Condition	Différence moyenne (I-J)	Erreur standard	ddl	Sig.[b]
Sham	taVNS	-,307	,512	56	,551
taVNS	Sham	,307	,512	56	,551

Comparaisons appariées[a]

		Intervalle de confiance à 95 % pour la différence[b]	
(I) Condition	(J) Condition	Borne inférieure	Borne supérieure
Sham	taVNS	-1,333	,719
taVNS	Sham	-,719	1,333

Basées sur les moyennes marginales estimées[a]

a. Variable dépendante : Vibrotactile Intensity.

b. Ajustement pour les comparaisons multiples : Bonferroni.

Tests univariés[a]

Ddl du numérateur	Ddl du dénominateur	F	Sig.
1	56	,360	,551

Le test de F permet de tester l'effet de Condition. Il s'appuie sur les comparaisons appariées (indépendantes) linéaires parmi les moyennes marginales estimées.[a]

a. Variable dépendante : Vibrotactile Intensity.

3. Phase

Estimations[a]

				Intervalle de confiance à 95 %	
Phase	Moyenne	Erreur standard	ddl	Borne inférieure	Borne supérieure
OFF	4,694	,362	56	3,969	5,420
ON	4,604	,362	56	3,878	5,329

a. Variable dépendante : Vibrotactile Intensity.

Comparaisons appariées[a]

(I) Phase	(J) Phase	Différence moyenne (I-J)	Erreur standard	ddl	Sig.[b]	Intervalle de confiance à 95 % pour la différence[b] Borne inférieure
OFF	ON	,090	,512	56	,861	-,936
ON	OFF	-,090	,512	56	,861	-1,116

Comparaisons appariées[a]

(I) Phase	(J) Phase	Intervalle de confiance à 95 % pour la différence Borne supérieure
OFF	ON	1,116
ON	OFF	,936

Basées sur les moyennes marginales estimées[a]

a. Variable dépendante : Vibrotactile Intensity.

b. Ajustement pour les comparaisons multiples : Bonferroni.

Tests univariés[a]

Ddl du numérateur	Ddl du dénominateur	F	Sig.
1	56	,031	,861

Le test de F permet de tester l'effet de Phase. Il s'appuie sur les comparaisons appariées (indépendantes) linéaires parmi les moyennes marginales estimées.[a]

a. Variable dépendante : Vibrotactile Intensity.

4. Condition * Phase[a]

Condition	Phase	Moyenne	Erreur standard	ddl	Intervalle de confiance à 95 % Borne inférieure	Borne supérieure
Sham	OFF	4,532	,512	56	3,506	5,558
	ON	4,458	,512	56	3,432	5,484
taVNS	OFF	4,856	,512	56	3,830	5,882
	ON	4,749	,512	56	3,723	5,775

a. Variable dépendante : Vibrotactile Intensity.

```
DATASET ACTIVATE Jeu_de_données1.

GET DATA
  /TYPE=XLSX
```

3. Experiment 3 :

3.1. Laser Intensity.

```
 /FILE='C:\Users\Mandumoulin\Documents\PhD 1ère année\TVNS and
pain\Articles\Review\Data\EEGs\Experiment 3\Experiment 3.xlsx'
  /SHEET=name 'Behavioral'
  /CELLRANGE=FULL
  /READNAMES=ON
  /DATATYPEMIN PERCENTAGE=95.0
  /HIDDEN IGNORE=YES.
EXECUTE.
DATASET NAME Jeu_de_données3 WINDOW=FRONT.
MIXED LaserIntensity BY Phase
  /CRITERIA=CIN(95) MXITER(100) MXSTEP(10) SCORING(1)
SINGULAR(0.000000000001) HCONVERGE(0,
    ABSOLUTE) LCONVERGE(0, ABSOLUTE) PCONVERGE(0.000001, ABSOLUTE)
  /FIXED=Phase | SSTYPE(3)
  /METHOD=REML
  /PRINT=CPS CORB COVB DESCRIPTIVES G  SOLUTION TESTCOV
  /EMMEANS=TABLES(OVERALL)
  /EMMEANS=TABLES(Phase) COMPARE ADJ(BONFERRONI).
```

Remarques

Sortie obtenue		04-MAY-2021 13:59:58
Commentaires		
Entrée	Jeu de données actif	Jeu_de_données3
	Filtre	<sans>
	Pondération	<sans>
	Fichier scindé	<sans>
	N de lignes dans le fichier de travail	41
Gestion des valeurs manquantes	Définition de la valeur manquante	Les valeurs manquantes définies par l'utilisateur sont traitées comme étant manquantes.
	Observations utilisées	Les statistiques sont basées sur toutes les observations comportant des données valides pour toutes les variables du modèle.

Syntaxe		MIXED LaserIntensity BY Phase<br>/CRITERIA=CIN(95) MXITER(100) MXSTEP(10) SCORING(1) SINGULAR(0.000000000001) HCONVERGE(0, ABSOLUTE) LCONVERGE(0, ABSOLUTE) PCONVERGE(0.000001, ABSOLUTE)<br>/FIXED=Phase | SSTYPE(3)<br>/METHOD=REML<br>/PRINT=CPS CORB COVB DESCRIPTIVES G SOLUTION TESTCOV<br>/EMMEANS=TABLES(OVERALL)<br>/EMMEANS=TABLES(Phase) COMPARE ADJ(BONFERRONI).
Ressources	Temps de processeur	00:00:00,00
	Temps écoulé	00:00:00,01

Récapitulatif de traitement des observations

		Effectif	Pourcentage marginal
Phase	OFF	12	50,0%
	ON	12	50,0%
Valide		24	100,0%
Exclues		17	
Total		41	

Statistiques descriptives

Laser Intensity

Phase	Effectif	Moyenne	Ecart type	Coefficient de variation
OFF	12	6,666517968030622	1,695862876545869	25,4%
ON	12	6,510206309391093	1,608603809598662	24,7%
Total	24	6,588362138710856	1,618449717732846	24,6%

Dimension du modèle[a]

		Nombre de niveaux	Nombre de paramètres
Effets fixes	Constante	1	1
	Phase	2	1
Résidu			1
Total		3	3

a. Variable dépendante : Laser Intensity.

Critères d'information[a]

Log de vraisemblance restreint -2	89,512
Critère d'information d'Akaike (AIC)	91,512
Critère de Hurvich et Tsai (AICC)	91,712
Critère de Bozdogan (CAIC)	93,603
Critère bayésien de Schwartz (BIC)	92,603

Les critères d'informations sont présentés en plus petit, disposant d'un meilleur format.[a]

a. Variable dépendante : Laser Intensity.

Effets fixes

Tests des effets fixes de type III[a]

Source	Ddl du numérateur	Ddl du dénominateur	F	Sig.
Constante	1	22	381,347	,000
Phase	1	22	,054	,819

a. Variable dépendante : Laser Intensity.

Estimations des effets fixes[a]

Paramètre	Estimation	Erreur standard	ddl	t	Sig.	Intervalle de confiance à 95 % Borne inférieure
Constante	6,510206	,477125	22	13,645	,000	5,520710
[Phase=OFF]	,156312	,674757	22	,232	,819	-1,243048
[Phase=ON]	0[b]	0	.	.	.	.

Estimations des effets fixes[a]

Paramètre	Intervalle de confiance à 95 % Borne supérieure
Constante	7,499703
[Phase=OFF]	1,555671
[Phase=ON]	.

a. Variable dépendante : Laser Intensity.

b. Ce paramètre est défini sur 0, car il est redondant.

Matrice de corrélation pour les estimations des effets fixes[a]

Paramètre	Constante	[Phase=OFF]	[Phase=ON]
Constante	1	-,707	.[b]
[Phase=OFF]	-,707	1	.[b]
[Phase=ON]	.[b]	.[b]	.[b]

a. Variable dépendante : Laser Intensity.

b. La corrélation est manquante par défaut, car elle est associée à un paramètre redondant.

Matrice de covariance pour les estimations des effets fixes[a]

Paramètre	Constante	[Phase=OFF]	[Phase=ON]
Constante	,227648	-,227648	0[b]

[Phase=OFF]	-,227648	,455296	0[b]
[Phase=ON]	0[b]	0[b]	0[b]

a. Variable dépendante : Laser Intensity.

b. La covariance est définie sur 0, car elle est associée à un paramètre redondant.

Paramètres de covariance

Estimations des paramètres de covariance[a]

Paramètre	Estimation	Erreur standard	Z de Wald	Sig.	Intervalle de confiance à 95 % Borne inférieure	Borne supérieure
Résidu	2,731779	,823662	3,317	,001	1,512859	4,932788

a. Variable dépendante : Laser Intensity.

Matrice de corrélation pour les estimations des paramètres de covariance[a]

Paramètre	Résidu
Résidu	1

a. Variable dépendante : Laser Intensity.

Matrice de covariance pour les estimations des paramètres de covariance[a]

Paramètre	Résidu
Résidu	,678419

a. Variable dépendante : Laser Intensity.

Moyenne marginale estimée

1. Grand Mean[a]

Moyenne	Erreur standard	ddl	Intervalle de confiance à 95 % Borne inférieure	Borne supérieure
6,588	,337	22	5,889	7,288

a. Variable dépendante : Laser Intensity.

2. Phase

Estimations[a]

Phase	Moyenne	Erreur standard	ddl	Intervalle de confiance à 95 % Borne inférieure	Borne supérieure
OFF	6,667	,477	22	5,677	7,656
ON	6,510	,477	22	5,521	7,500

a. Variable dépendante : Laser Intensity.

Comparaisons appariées[a]

(I) Phase	(J) Phase	Différence moyenne (I-J)	Erreur standard	ddl	Sig.[b]	Intervalle de confiance à 95 % pour la différence[b] Borne inférieure
OFF	ON	,156	,675	22	,819	-1,243
ON	OFF	-,156	,675	22	,819	-1,556

Comparaisons appariées[a]

(I) Phase	(J) Phase	Intervalle de confiance à 95 % pour la différence Borne supérieure
OFF	ON	1,556
ON	OFF	1,243

Basées sur les moyennes marginales estimées[a]

a. Variable dépendante : Laser Intensity.

b. Ajustement pour les comparaisons multiples : Bonferroni.

Tests univariés[a]

Ddl du numérateur	Ddl du dénominateur	F	Sig.

1	22	,054	,819

Le test de F permet de tester l'effet de Phase. Il s'appuie sur les comparaisons appariées (indépendantes) linéaires parmi les moyennes marginales estimées.[a]

a. Variable dépendante : Laser Intensity.

3.2.Vibrotactile Intensity.

```
MIXED VibrotactileIntensity BY Phase
  /CRITERIA=CIN(95) MXITER(100) MXSTEP(10) SCORING(1)
SINGULAR(0.000000000001) HCONVERGE(0,
    ABSOLUTE) LCONVERGE(0, ABSOLUTE) PCONVERGE(0.000001, ABSOLUTE)
  /FIXED=Phase | SSTYPE(3)
  /METHOD=REML
  /PRINT=CPS CORB COVB DESCRIPTIVES G  SOLUTION TESTCOV
  /EMMEANS=TABLES(OVERALL)
  /EMMEANS=TABLES(Phase) COMPARE ADJ(BONFERRONI).
```

Remarques

Sortie obtenue		04-MAY-2021 14:01:00
Commentaires		
Entrée	Jeu de données actif	Jeu_de_données3
	Filtre	<sans>
	Pondération	<sans>
	Fichier scindé	<sans>
	N de lignes dans le fichier de travail	41
Gestion des valeurs manquantes	Définition de la valeur manquante	Les valeurs manquantes définies par l'utilisateur sont traitées comme étant manquantes.
	Observations utilisées	Les statistiques sont basées sur toutes les observations comportant des données valides pour toutes les variables du modèle.

Syntaxe		MIXED VibrotactileIntensity BY Phase<br>/CRITERIA=CIN(95) MXITER(100) MXSTEP(10) SCORING(1) SINGULAR(0.000000000001) HCONVERGE(0, ABSOLUTE) LCONVERGE(0, ABSOLUTE) PCONVERGE(0.000001, ABSOLUTE)<br>/FIXED=Phase | SSTYPE(3)<br>/METHOD=REML<br>/PRINT=CPS CORB COVB DESCRIPTIVES G SOLUTION TESTCOV<br>/EMMEANS=TABLES(OVERALL)<br>/EMMEANS=TABLES(Phase) COMPARE ADJ(BONFERRONI).
Ressources	Temps de processeur	00:00:00,02
	Temps écoulé	00:00:00,01

Récapitulatif de traitement des observations

		Effectif	Pourcentage marginal
Phase	OFF	11	50,0%
	ON	11	50,0%
Valide		22	100,0%
Exclues		19	
Total		41	

Statistiques descriptives

Vibrotactile Intensity

Phase	Effectif	Moyenne	Ecart type	Coefficient de variation
OFF	11	6,452689604019543	2,598610267059415	40,3%
ON	11	6,492239643846227	2,671569560666501	41,2%
Total	22	6,472464623932885	2,571910495244645	39,7%

Dimension du modèle[a]

		Nombre de niveaux	Nombre de paramètres
Effets fixes	Constante	1	1
	Phase	2	1
Résidu			1
Total		3	3

a. Variable dépendante : Vibrotactile Intensity.

Critères d'information[a]

Log de vraisemblance restreint -2	100,314
Critère d'information d'Akaike (AIC)	102,314
Critère de Hurvich et Tsai (AICC)	102,536
Critère de Bozdogan (CAIC)	104,310
Critère bayésien de Schwartz (BIC)	103,310

Les critères d'informations sont présentés en plus petit, disposant d'un meilleur format.[a]

a. Variable dépendante : Vibrotactile Intensity.

Effets fixes

Tests des effets fixes de type III[a]

Source	Ddl du numérateur	Ddl du dénominateur	F	Sig.
Constante	1	20	132,705	,000
Phase	1	20	,001	,972

a. Variable dépendante : Vibrotactile Intensity.

Estimations des effets fixes[a]

Paramètre	Estimation	Erreur standard	ddl	t	Sig.	Intervalle de confiance à 95 % Borne inférieure
Constante	6,492240	,794586	20	8,171	,000	4,834763
[Phase=OFF]	-,039550	1,123714	20	-,035	,972	-2,383576
[Phase=ON]	0[b]	0	.	.	.	.

Estimations des effets fixes[a]

Paramètre	Intervalle de confiance à 95 % Borne supérieure
Constante	8,149716
[Phase=OFF]	2,304476
[Phase=ON]	.

a. Variable dépendante : Vibrotactile Intensity.

b. Ce paramètre est défini sur 0, car il est redondant.

Matrice de corrélation pour les estimations des effets fixes[a]

Paramètre	Constante	[Phase=OFF]	[Phase=ON]
Constante	1	-,707	.[b]
[Phase=OFF]	-,707	1	.[b]
[Phase=ON]	.[b]	.[b]	.[b]

a. Variable dépendante : Vibrotactile Intensity.

b. La corrélation est manquante par défaut, car elle est associée à un paramètre redondant.

Matrice de covariance pour les estimations des effets fixes[a]

Paramètre	Constante	[Phase=OFF]	[Phase=ON]
Constante	,631366	-,631366	0[b]

[Phase=OFF]	-,631366	1,262733	0[b]
[Phase=ON]	0[b]	0[b]	0[b]

a. Variable dépendante : Vibrotactile Intensity.

b. La covariance est définie sur 0, car elle est associée à un paramètre redondant.

Paramètres de covariance

Estimations des paramètres de covariance[a]

Paramètre	Estimation	Erreur standard	Z de Wald	Sig.	Intervalle de confiance à 95 % Borne inférieure	Intervalle de confiance à 95 % Borne supérieure
Résidu	6,945030	2,196211	3,162	,002	3,736806	12,907664

a. Variable dépendante : Vibrotactile Intensity.

Matrice de corrélation pour les estimations des paramètres de covariance[a]

Paramètre	Résidu
Résidu	1

a. Variable dépendante : Vibrotactile Intensity.

Matrice de covariance pour les estimations des paramètres de covariance[a]

Paramètre	Résidu
Résidu	4,823344

a. Variable dépendante : Vibrotactile Intensity.

Moyenne marginale estimée

1. Grand Mean[a]

Moyenne	Erreur standard	ddl	Intervalle de confiance à 95 % Borne inférieure	Intervalle de confiance à 95 % Borne supérieure
6,472	,562	20	5,300	7,644

a. Variable dépendante : Vibrotactile Intensity.

2. Phase

Estimations[a]

Phase	Moyenne	Erreur standard	ddl	Intervalle de confiance à 95 % Borne inférieure	Intervalle de confiance à 95 % Borne supérieure
OFF	6,453	,795	20	4,795	8,110
ON	6,492	,795	20	4,835	8,150

a. Variable dépendante : Vibrotactile Intensity.

Comparaisons appariées[a]

(I) Phase	(J) Phase	Différence moyenne (I-J)	Erreur standard	ddl	Sig.[b]	Intervalle de confiance à 95 % pour la différence[b] Borne inférieure
OFF	ON	-,040	1,124	20	,972	-2,384
ON	OFF	,040	1,124	20	,972	-2,304

Comparaisons appariées[a]

(I) Phase	(J) Phase	Intervalle de confiance à 95 % pour la différence Borne supérieure
OFF	ON	2,304
ON	OFF	2,384

Basées sur les moyennes marginales estimées[a]

a. Variable dépendante : Vibrotactile Intensity.

b. Ajustement pour les comparaisons multiples : Bonferroni.

Tests univariés[a]

Ddl du numérateur	Ddl du dénominateur	F	Sig.
1	20	,001	,972

Le test de F permet de tester l'effet de Phase. Il s'appuie sur les comparaisons appariées (indépendantes) linéaires parmi les moyennes marginales estimées.[a]

a. Variable dépendante : Vibrotactile Intensity.
