# Supplementary material for: Transcutaneous auricular VNS applied to experimental pain: A paired behavioral and EEG study using thermonociceptive CO2 laser
Source: PLoS One. 2021 Jul 12;16(7):e0254480. doi: 10.1371/journal.pone.0254480 (PMC8274876; doi:10.1371/journal.pone.0254480)
Supplement: S1 Appendix — (ZIP) [file pone.0254480.s001.zip › Supplementary Analysis_LMM_Cerebral responses DIFF.pdf]

Transcutaneous VNS applied to experimental pain: a paired behavioral and EEG study using thermonociceptive CO2 laser

Supplementary Appendix

Linear Mixed Models: Detailed analysis.

ΔCEREBRAL RESPONSES

1. Experiment 1.

1.1. Laser-evoked potentials (LEPs).

1.1.1. LEPs N2P2 Amplitude (ΔT0-T2).

```
MIXED LaserN2P2AmplitudeΔT0T2 BY Condition
  /CRITERIA=CIN(95) MXITER(100) MXSTEP(10) SCORING(1)
SINGULAR(0.000000000001) HCONVERGE(0,
    ABSOLUTE) LCONVERGE(0, ABSOLUTE) PCONVERGE(0.000001, ABSOLUTE)
  /FIXED=Condition | SSTYPE(3)
  /METHOD=REML
  /PRINT=CPS CORB COVB DESCRIPTIVES G  SOLUTION TESTCOV
  /EMMEANS=TABLES(OVERALL)
  /EMMEANS=TABLES(Condition) COMPARE ADJ(BONFERRONI).
```

Remarques

Sortie obtenue		05-MAY-2021 12:42:16
Commentaires		
Entrée	Jeu de données actif	Jeu_de_données4
	Filtre	<sans>
	Pondération	<sans>
	Fichier scindé	<sans>
	N de lignes dans le fichier de travail	44
Gestion des valeurs manquantes	Définition de la valeur manquante	Les valeurs manquantes définies par l'utilisateur sont traitées comme étant manquantes.
	Observations utilisées	Les statistiques sont basées sur toutes les observations comportant des données valides pour toutes les variables du modèle.

Syntaxe		MIXED LaserN2P2AmplitudeΔT0T2 BY Condition<br>/CRITERIA=CIN(95) MXITER(100) MXSTEP(10) SCORING(1) SINGULAR(0.000000000001) HCONVERGE(0, ABSOLUTE) LCONVERGE(0, ABSOLUTE) PCONVERGE(0.000001, ABSOLUTE)<br>/FIXED=Condition | SSTYPE(3)<br>/METHOD=REML<br>/PRINT=CPS CORB COVB DESCRIPTIVES G SOLUTION TESTCOV<br>/EMMEANS=TABLES(OVERALL)<br>/EMMEANS=TABLES(Condition) COMPARE ADJ(BONFERRONI).
Ressources	Temps de processeur	00:00:00,02
	Temps écoulé	00:00:00,02

Récapitulatif de traitement des observations

		Effectif	Pourcentage marginal
Condition	Sham	22	50,0%
	taVNS	22	50,0%
Valide		44	100,0%
Exclues		0	
Total		44	

Statistiques descriptives

Laser N2P2 Amplitude ΔT0-T2

Condition	Effectif	Moyenne	Ecart type	Coefficient de variation
Sham	22	1,017713636363637	9,604607758253845	943,7%
taVNS	22	,262665909090909	9,171984676945884	3491,9%
Total	44	,640189772727273	9,288803866921572	1450,9%

Dimension du modèle[a]

		Nombre de niveaux	Nombre de paramètres
Effets fixes	Constante	1	1
	Condition	2	1
Résidu			1
Total		3	3

a. Variable dépendante : Laser N2P2 Amplitude ΔT0-T2.

Critères d'information[a]

Log de vraisemblance restreint -2	313,510
Critère d'information d'Akaike (AIC)	315,510
Critère de Hurvich et Tsai (AICC)	315,610
Critère de Bozdogan (CAIC)	318,248
Critère bayésien de Schwartz (BIC)	317,248

Les critères d'informations sont présentés en plus petit, disposant d'un meilleur format.[a]

a. Variable dépendante : Laser N2P2 Amplitude ΔT0-T2.

Effets fixes

Tests des effets fixes de type III[a]

Source	Ddl du numérateur	Ddl du dénominateur	F	Sig.
Constante	1	42	,204	,653
Condition	1	42	,071	,791

a. Variable dépendante : Laser N2P2 Amplitude ΔT0-T2.

Estimations des effets fixes[a]

Paramètre	Estimation	Erreur standard	ddl	t	Sig.	Intervalle de confiance à 95 % Borne inférieure
Constante	,262666	2,002123	42	,131	,896	-3,777781
[Condition=Sham]	,755048	2,831429	42	,267	,791	-4,959007
[Condition=taVNS]	0[b]	0	.	.	.	.

Estimations des effets fixes[a]

Paramètre	Intervalle de confiance à 95 % Borne supérieure
Constante	4,303113
[Condition=Sham]	6,469103
[Condition=taVNS]	.

a. Variable dépendante : Laser N2P2 Amplitude ΔT0-T2.

b. Ce paramètre est défini sur 0, car il est redondant.

Matrice de corrélation pour les estimations des effets fixes[a]

Paramètre	Constante	[Condition=Sham]	[Condition=taVNS]
Constante	1	-,707	.[b]
[Condition=Sham]	-,707	1	.[b]
[Condition=taVNS]	.[b]	.[b]	.[b]

a. Variable dépendante : Laser N2P2 Amplitude ΔT0-T2.

b. La corrélation est manquante par défaut, car elle est associée à un paramètre redondant.

Matrice de covariance pour les estimations des effets fixes[a]

Paramètre	Constante	[Condition=Sham]	[Condition=taVNS]
Constante	4,008495	-4,008495	0[b]
[Condition=Sham]	-4,008495	8,016991	0[b]
[Condition=taVNS]	0[b]	0[b]	0[b]

a. Variable dépendante : Laser N2P2 Amplitude ΔT0-T2.

b. La covariance est définie sur 0, car elle est associée à un paramètre redondant.

Paramètres de covariance

Estimations des paramètres de covariance[a]

Paramètre	Estimation	Erreur standard	Z de Wald	Sig.	Intervalle de confiance à 95 %	
					Borne inférieure	Borne supérieure
Résidu	88,186897	19,243959	4,583	,000	57,498518	135,254419

a. Variable dépendante : Laser N2P2 Amplitude ΔT0-T2.

Matrice de corrélation pour les estimations des paramètres de covariance[a]

Paramètre	Résidu
Résidu	1

a. Variable dépendante : Laser N2P2 Amplitude ΔT0-T2.

Matrice de covariance pour les estimations des paramètres de covariance[a]

Paramètre	Résidu
Résidu	370,329939

a. Variable dépendante : Laser N2P2 Amplitude ΔT0-T2.

Moyenne marginale estimée

1. Grand Mean[a]

Moyenne	Erreur standard	ddl	Intervalle de confiance à 95 % Borne inférieure	Borne supérieure
,640	1,416	42	-2,217	3,497

a. Variable dépendante : Laser N2P2 Amplitude ΔT0-T2.

2. Condition

Estimations[a]

Condition	Moyenne	Erreur standard	ddl	Intervalle de confiance à 95 % Borne inférieure	Borne supérieure
Sham	1,018	2,002	42	-3,023	5,058
taVNS	,263	2,002	42	-3,778	4,303

a. Variable dépendante : Laser N2P2 Amplitude ΔT0-T2.

Comparaisons appariées[a]

(I) Condition	(J) Condition	Différence moyenne (I-J)	Erreur standard	ddl	Sig.[b]
Sham	taVNS	,755	2,831	42	,791
taVNS	Sham	-,755	2,831	42	,791

Comparaisons appariées[a]

(I) Condition	(J) Condition	Intervalle de confiance à 95 % pour la différence[b] Borne inférieure	Borne supérieure
Sham	taVNS	-4,959	6,469
taVNS	Sham	-6,469	4,959

Basées sur les moyennes marginales estimées[a]

a. Variable dépendante : Laser N2P2 Amplitude ΔT0-T2.

b. Ajustement pour les comparaisons multiples : Bonferroni.

Tests univariés[a]

Ddl du numérateur	Ddl du dénominateur	F	Sig.
1	42	,071	,791

Le test de F permet de tester l'effet de Condition. Il s'appuie sur les comparaisons appariées (indépendantes) linéaires parmi les moyennes marginales estimées.[a]

a. Variable dépendante : Laser N2P2 Amplitude ΔT0-T2.

1.1.2. LEPs N2 Amplitude (ΔT0-T2).

```
MIXED LaserN2AmplitudeΔT0T2 BY Condition
  /CRITERIA=CIN(95) MXITER(100) MXSTEP(10) SCORING(1)
SINGULAR(0.000000000001) HCONVERGE(0,
    ABSOLUTE) LCONVERGE(0, ABSOLUTE) PCONVERGE(0.000001, ABSOLUTE)
  /FIXED=Condition | SSTYPE(3)
  /METHOD=REML
  /PRINT=CPS CORB COVB DESCRIPTIVES G  SOLUTION TESTCOV
  /EMMEANS=TABLES(OVERALL)
  /EMMEANS=TABLES(Condition) COMPARE ADJ(BONFERRONI).
```

Remarques

Sortie obtenue		05-MAY-2021 12:43:35
Commentaires		
Entrée	Jeu de données actif	Jeu_de_données4
	Filtre	<sans>
	Pondération	<sans>
	Fichier scindé	<sans>
	N de lignes dans le fichier de travail	44
Gestion des valeurs manquantes	Définition de la valeur manquante	Les valeurs manquantes définies par l'utilisateur sont traitées comme étant manquantes.
	Observations utilisées	Les statistiques sont basées sur toutes les observations comportant des données valides pour toutes les variables du modèle.

Syntaxe		MIXED LaserN2AmplitudeΔT0T2 BY Condition<br>/CRITERIA=CIN(95) MXITER(100) MXSTEP(10) SCORING(1) SINGULAR(0.000000000001) HCONVERGE(0, ABSOLUTE) LCONVERGE(0, ABSOLUTE) PCONVERGE(0.000001, ABSOLUTE)<br>/FIXED=Condition | SSTYPE(3)<br>/METHOD=REML<br>/PRINT=CPS CORB COVB DESCRIPTIVES G SOLUTION TESTCOV<br>/EMMEANS=TABLES(OVERALL)<br>/EMMEANS=TABLES(Condition) COMPARE ADJ(BONFERRONI).
Ressources	Temps de processeur	00:00:00,00
	Temps écoulé	00:00:00,01

Récapitulatif de traitement des observations

		Effectif	Pourcentage marginal
Condition	Sham	22	50,0%
	taVNS	22	50,0%
Valide		44	100,0%
Exclues		0	
Total		44	

Statistiques descriptives

Laser N2 Amplitude ΔT0-T2

Condition	Effectif	Moyenne	Ecart type	Coefficient de variation
Sham	22	-,517331818181818	5,730130199102216	-1107,6%
taVNS	22	-,458023636363636	5,862874318800704	-1280,0%
Total	44	-,487677727272727	5,729158643891652	-1174,8%

Dimension du modèle[a]

		Nombre de niveaux	Nombre de paramètres
Effets fixes	Constante	1	1
	Condition	2	1
Résidu			1
Total		3	3

a. Variable dépendante : Laser N2 Amplitude ΔT0-T2.

Critères d'information[a]

Log de vraisemblance restreint -2	272,988
Critère d'information d'Akaike (AIC)	274,988
Critère de Hurvich et Tsai (AICC)	275,088
Critère de Bozdogan (CAIC)	277,725
Critère bayésien de Schwartz (BIC)	276,725

Les critères d'informations sont présentés en plus petit, disposant d'un meilleur format.[a]

a. Variable dépendante : Laser N2 Amplitude ΔT0-T2.

Effets fixes

Tests des effets fixes de type III[a]

Source	Ddl du numérateur	Ddl du dénominateur	F	Sig.
Constante	1	42	,311	,580
Condition	1	42	,001	,973

a. Variable dépendante : Laser N2 Amplitude ΔT0-T2.

Estimations des effets fixes[a]

Paramètre	Estimation	Erreur standard	ddl	t	Sig.	Intervalle de confiance à 95 % Borne inférieure
Constante	-,458024	1,235899	42	-,371	,713	-2,952170
[Condition=Sham]	-,059308	1,747826	42	-,034	,973	-3,586563
[Condition=taVNS]	0[b]	0	.	.	.	.

Estimations des effets fixes[a]

Paramètre	Intervalle de confiance à 95 % Borne supérieure
Constante	2,036122
[Condition=Sham]	3,467947
[Condition=taVNS]	.

a. Variable dépendante : Laser N2 Amplitude ΔT0-T2.

b. Ce paramètre est défini sur 0, car il est redondant.

Matrice de corrélation pour les estimations des effets fixes[a]

Paramètre	Constante	[Condition=Sham]	[Condition=taVNS]
Constante	1	-,707	.[b]
[Condition=Sham]	-,707	1	.[b]
[Condition=taVNS]	.[b]	.[b]	.[b]

a. Variable dépendante : Laser N2 Amplitude ΔT0-T2.

b. La corrélation est manquante par défaut, car elle est associée à un paramètre redondant.

Matrice de covariance pour les estimations des effets fixes[a]

Paramètre	Constante	[Condition=Sham]	[Condition=taVNS]
Constante	1,527447	-1,527447	0[b]

[Condition=Sham]	-1,527447	3,054895	0[b]
[Condition=taVNS]	0[b]	0[b]	0[b]

a. Variable dépendante : Laser N2 Amplitude ΔT0-T2.

b. La covariance est définie sur 0, car elle est associée à un paramètre redondant.

Paramètres de covariance

Estimations des paramètres de covariance[a]

Paramètre	Estimation	Erreur standard	Z de Wald	Sig.	Intervalle de confiance à 95 % Borne inférieure	Borne supérieure
Résidu	33,603844	7,332960	4,583	,000	21,909958	51,539044

a. Variable dépendante : Laser N2 Amplitude ΔT0-T2.

Matrice de corrélation pour les estimations des paramètres de covariance[a]

Paramètre	Résidu
Résidu	1

a. Variable dépendante : Laser N2 Amplitude ΔT0-T2.

Matrice de covariance pour les estimations des paramètres de covariance[a]

Paramètre	Résidu
Résidu	53,772301

a. Variable dépendante : Laser N2 Amplitude ΔT0-T2.

Moyenne marginale estimée

1. Grand Mean[a]

Moyenne	Erreur standard	ddl	Intervalle de confiance à 95 % Borne inférieure	Borne supérieure
-,488	,874	42	-2,251	1,276

a. Variable dépendante : Laser N2 Amplitude ΔT0-T2.

2. Condition

Estimations[a]

Condition	Moyenne	Erreur standard	ddl	Intervalle de confiance à 95 % Borne inférieure	Borne supérieure
Sham	-,517	1,236	42	-3,011	1,977
taVNS	-,458	1,236	42	-2,952	2,036

a. Variable dépendante : Laser N2 Amplitude ΔT0-T2.

Comparaisons appariées[a]

(I) Condition	(J) Condition	Différence moyenne (I-J)	Erreur standard	ddl	Sig.[b]
Sham	taVNS	-,059	1,748	42	,973
taVNS	Sham	,059	1,748	42	,973

Comparaisons appariées[a]

(I) Condition	(J) Condition	Intervalle de confiance à 95 % pour la différence[b] Borne inférieure	Borne supérieure
Sham	taVNS	-3,587	3,468
taVNS	Sham	-3,468	3,587

Basées sur les moyennes marginales estimées[a]

a. Variable dépendante : Laser N2 Amplitude ΔT0-T2.

b. Ajustement pour les comparaisons multiples : Bonferroni.

Tests univariés[a]

Ddl du numérateur	Ddl du dénominateur	F	Sig.
1	42	,001	,973

Le test de F permet de tester l'effet de Condition. Il s'appuie sur les comparaisons appariées (indépendantes) linéaires parmi les moyennes marginales estimées.[a]

a. Variable dépendante : Laser N2 Amplitude ΔT0-T2.

1.1.3. LEPs N2 Latency (ΔT0-T2).

```
MIXED LaserN2LatencyΔT0T2 BY Condition
  /CRITERIA=CIN(95) MXITER(100) MXSTEP(10) SCORING(1)
SINGULAR(0.000000000001) HCONVERGE(0,
    ABSOLUTE) LCONVERGE(0, ABSOLUTE) PCONVERGE(0.000001, ABSOLUTE)
  /FIXED=Condition | SSTYPE(3)
  /METHOD=REML
  /PRINT=CPS CORB COVB DESCRIPTIVES G  SOLUTION TESTCOV
  /EMMEANS=TABLES(OVERALL)
  /EMMEANS=TABLES(Condition) COMPARE ADJ(BONFERRONI).
```

Remarques

Sortie obtenue		05-MAY-2021 12:44:33
Commentaires		
Entrée	Jeu de données actif	Jeu_de_données4
	Filtre	<sans>
	Pondération	<sans>
	Fichier scindé	<sans>
	N de lignes dans le fichier de travail	44
Gestion des valeurs manquantes	Définition de la valeur manquante	Les valeurs manquantes définies par l'utilisateur sont traitées comme étant manquantes.
	Observations utilisées	Les statistiques sont basées sur toutes les observations comportant des données valides pour toutes les variables du modèle.

Syntaxe		MIXED LaserN2LatencyΔT0T2 BY Condition<br>/CRITERIA=CIN(95) MXITER(100) MXSTEP(10) SCORING(1) SINGULAR(0.000000000001) HCONVERGE(0, ABSOLUTE) LCONVERGE(0, ABSOLUTE) PCONVERGE(0.000001, ABSOLUTE)<br>/FIXED=Condition | SSTYPE(3)<br>/METHOD=REML<br>/PRINT=CPS CORB COVB DESCRIPTIVES G SOLUTION TESTCOV<br>/EMMEANS=TABLES(OVERALL)<br>/EMMEANS=TABLES(Condition) COMPARE ADJ(BONFERRONI).
Ressources	Temps de processeur	00:00:00,02
	Temps écoulé	00:00:00,01

Récapitulatif de traitement des observations

		Effectif	Pourcentage marginal
Condition	Sham	21	50,0%
	taVNS	21	50,0%
Valide		42	100,0%
Exclues		2	
Total		44	

Statistiques descriptives

Laser N2 Latency ΔT0-T2

Condition	Effectif	Moyenne	Ecart type	Coefficient de variation
Sham	21	,005238571428571	,052018614580332	993,0%
taVNS	21	,018191428571429	,029018761738867	159,5%
Total	42	,011715000000000	,042115447965290	359,5%

Dimension du modèle[a]

		Nombre de niveaux	Nombre de paramètres
Effets fixes	Constante	1	1
	Condition	2	1
Résidu			1
Total		3	3

a. Variable dépendante : Laser N2 Latency ΔT0-T2.

Critères d'information[a]

Log de vraisemblance restreint -2	-133,776
Critère d'information d'Akaike (AIC)	-131,776
Critère de Hurvich et Tsai (AICC)	-131,671
Critère de Bozdogan (CAIC)	-129,087
Critère bayésien de Schwartz (BIC)	-130,087

Les critères d'informations sont présentés en plus petit, disposant d'un meilleur format.[a]

a. Variable dépendante : Laser N2 Latency ΔT0-T2.

Effets fixes

Tests des effets fixes de type III[a]

Source	Ddl du numérateur	Ddl du dénominateur	F	Sig.
Constante	1	40	3,249	,079
Condition	1	40	,993	,325

a. Variable dépendante : Laser N2 Latency ΔT0-T2.

Estimations des effets fixes[a]

Paramètre	Estimation	Erreur standard	ddl	t	Sig.	Intervalle de confiance à 95 % Borne inférieure
Constante	,018191	,009191	40	1,979	,055	-,000385
[Condition=Sham]	-,012953	,012998	40	-,997	,325	-,039223
[Condition=taVNS]	0[b]	0	.	.	.	.

Estimations des effets fixes[a]

Paramètre	Intervalle de confiance à 95 % Borne supérieure
Constante	,036767
[Condition=Sham]	,013318
[Condition=taVNS]	.

a. Variable dépendante : Laser N2 Latency ΔT0-T2.

b. Ce paramètre est défini sur 0, car il est redondant.

Matrice de corrélation pour les estimations des effets fixes[a]

Paramètre	Constante	[Condition=Sham]	[Condition=taVNS]
Constante	1	-,707	.[b]
[Condition=Sham]	-,707	1	.[b]
[Condition=taVNS]	.[b]	.[b]	.[b]

a. Variable dépendante : Laser N2 Latency ΔT0-T2.

b. La corrélation est manquante par défaut, car elle est associée à un paramètre redondant.

Matrice de covariance pour les estimations des effets fixes[a]

Paramètre	Constante	[Condition=Sham]	[Condition=taVNS]
Constante	8,447678E-5	-8,447678E-5	0[b]

[Condition=Sham]	-8,447678E-5	,000169	0[b]
[Condition=taVNS]	0[b]	0[b]	0[b]

a. Variable dépendante : Laser N2 Latency ΔT0-T2.

b. La covariance est définie sur 0, car elle est associée à un paramètre redondant.

Paramètres de covariance

Estimations des paramètres de covariance[a]

Paramètre	Estimation	Erreur standard	Z de Wald	Sig.	Intervalle de confiance à 95 % Borne inférieure	Borne supérieure
Résidu	,001774	,000397	4,472	,000	,001145	,002750

a. Variable dépendante : Laser N2 Latency ΔT0-T2.

Matrice de corrélation pour les estimations des paramètres de covariance[a]

Paramètre	Résidu
Résidu	1

a. Variable dépendante : Laser N2 Latency ΔT0-T2.

Matrice de covariance pour les estimations des paramètres de covariance[a]

Paramètre	Résidu
Résidu	1,573560E-7

a. Variable dépendante : Laser N2 Latency ΔT0-T2.

Moyenne marginale estimée

1. Grand Mean[a]

Moyenne	Erreur standard	ddl	Intervalle de confiance à 95 % Borne inférieure	Borne supérieure
,012	,006	40	-,001	,025

a. Variable dépendante : Laser N2 Latency ΔT0-T2.

2. Condition

Estimations[a]

Condition	Moyenne	Erreur standard	ddl	Intervalle de confiance à 95 % Borne inférieure	Borne supérieure
Sham	,005	,009	40	-,013	,024
taVNS	,018	,009	40	,000	,037

a. Variable dépendante : Laser N2 Latency ΔT0-T2.

Comparaisons appariées[a]

(I) Condition	(J) Condition	Différence moyenne (I-J)	Erreur standard	ddl	Sig.[b]
Sham	taVNS	-,013	,013	40	,325
taVNS	Sham	,013	,013	40	,325

Comparaisons appariées[a]

(I) Condition	(J) Condition	Intervalle de confiance à 95 % pour la différence[b] Borne inférieure	Borne supérieure
Sham	taVNS	-,039	,013
taVNS	Sham	-,013	,039

Basées sur les moyennes marginales estimées[a]

a. Variable dépendante : Laser N2 Latency ΔT0-T2.

b. Ajustement pour les comparaisons multiples : Bonferroni.

Tests univariés[a]

Ddl du numérateur	Ddl du dénominateur	F	Sig.
1	40	,993	,325

Le test de F permet de tester l'effet de Condition. Il s'appuie sur les comparaisons appariées (indépendantes) linéaires parmi les moyennes marginales estimées.[a]

a. Variable dépendante : Laser N2 Latency ΔT0-T2.

1.1.4. LEPs P2 Amplitude (ΔT0-T2).

```
MIXED LaserP2AmplitudeΔT0T2 BY Condition
  /CRITERIA=CIN(95) MXITER(100) MXSTEP(10) SCORING(1)
SINGULAR(0.000000000001) HCONVERGE(0,
    ABSOLUTE) LCONVERGE(0, ABSOLUTE) PCONVERGE(0.000001, ABSOLUTE)
  /FIXED=Condition | SSTYPE(3)
  /METHOD=REML
  /PRINT=CPS CORB COVB DESCRIPTIVES G  SOLUTION TESTCOV
  /EMMEANS=TABLES(OVERALL)
  /EMMEANS=TABLES(Condition) COMPARE ADJ(BONFERRONI).
```

Remarques

Sortie obtenue		05-MAY-2021 12:45:04
Commentaires		
Entrée	Jeu de données actif	Jeu_de_données4
	Filtre	<sans>
	Pondération	<sans>
	Fichier scindé	<sans>
	N de lignes dans le fichier de travail	44
Gestion des valeurs manquantes	Définition de la valeur manquante	Les valeurs manquantes définies par l'utilisateur sont traitées comme étant manquantes.
	Observations utilisées	Les statistiques sont basées sur toutes les observations comportant des données valides pour toutes les variables du modèle.

Syntaxe		MIXED LaserP2AmplitudeΔT0T2 BY Condition<br>/CRITERIA=CIN(95) MXITER(100) MXSTEP(10) SCORING(1) SINGULAR(0.000000000001) HCONVERGE(0,<br>ABSOLUTE) LCONVERGE(0, ABSOLUTE) PCONVERGE(0.000001, ABSOLUTE)<br>/FIXED=Condition | SSTYPE(3)<br>/METHOD=REML<br>/PRINT=CPS CORB COVB DESCRIPTIVES G SOLUTION TESTCOV<br><br>/EMMEANS=TABLES(OVERALL)<br><br>/EMMEANS=TABLES(Condition) COMPARE ADJ(BONFERRONI).
Ressources	Temps de processeur	00:00:00,02
	Temps écoulé	00:00:00,01

Récapitulatif de traitement des observations

		Effectif	Pourcentage marginal
Condition	Sham	22	50,0%
	taVNS	22	50,0%
Valide		44	100,0%
Exclues		0	
Total		44	

Statistiques descriptives

Laser P2 Amplitude ΔT0-T2

Condition	Effectif	Moyenne	Ecart type	Coefficient de variation
Sham	22	,500381818181819	5,861089478004707	1171,3%
taVNS	22	-,195357727272728	5,506191521056249	-2818,5%
Total	44	,152512045454545	5,630905999877273	3692,1%

Dimension du modèle[a]

		Nombre de niveaux	Nombre de paramètres
Effets fixes	Constante	1	1
	Condition	2	1
Résidu			1
Total		3	3

a. Variable dépendante : Laser P2 Amplitude ΔT0-T2.

Critères d'information[a]

Log de vraisemblance restreint -2	271,372
Critère d'information d'Akaike (AIC)	273,372
Critère de Hurvich et Tsai (AICC)	273,472
Critère de Bozdogan (CAIC)	276,109
Critère bayésien de Schwartz (BIC)	275,109

Les critères d'informations sont présentés en plus petit, disposant d'un meilleur format.[a]

a. Variable dépendante : Laser P2 Amplitude ΔT0-T2.

Effets fixes

Tests des effets fixes de type III[a]

Source	Ddl du numérateur	Ddl du dénominateur	F	Sig.
Constante	1	42	,032	,860
Condition	1	42	,165	,687

a. Variable dépendante : Laser P2 Amplitude ΔT0-T2.

Estimations des effets fixes[a]

Paramètre	Estimation	Erreur standard	ddl	t	Sig.	Intervalle de confiance à 95 % Borne inférieure
Constante	-,195358	1,212347	42	-,161	,873	-2,641972
[Condition=Sham]	,695740	1,714517	42	,406	,687	-2,764296
[Condition=taVNS]	0[b]	0	.	.	.	.

Estimations des effets fixes[a]

Paramètre	Intervalle de confiance à 95 % Borne supérieure
Constante	2,251257
[Condition=Sham]	4,155775
[Condition=taVNS]	.

a. Variable dépendante : Laser P2 Amplitude ΔT0-T2.

b. Ce paramètre est défini sur 0, car il est redondant.

Matrice de corrélation pour les estimations des effets fixes[a]

Paramètre	Constante	[Condition=Sham]	[Condition=taVNS]
Constante	1	-,707	.[b]
[Condition=Sham]	-,707	1	.[b]
[Condition=taVNS]	.[b]	.[b]	.[b]

a. Variable dépendante : Laser P2 Amplitude ΔT0-T2.

b. La corrélation est manquante par défaut, car elle est associée à un paramètre redondant.

Matrice de covariance pour les estimations des effets fixes[a]

Paramètre	Constante	[Condition=Sham]	[Condition=taVNS]
Constante	1,469784	-1,469784	0[b]

[Condition=Sham]	-1,469784	2,939569	0[b]
[Condition=taVNS]	0[b]	0[b]	0[b]

a. Variable dépendante : Laser P2 Amplitude ΔT0-T2.

b. La covariance est définie sur 0, car elle est associée à un paramètre redondant.

Paramètres de covariance

Estimations des paramètres de covariance[a]

Paramètre	Estimation	Erreur standard	Z de Wald	Sig.	Intervalle de confiance à 95 % Borne inférieure	Borne supérieure
Résidu	32,335257	7,056132	4,583	,000	21,082831	49,593382

a. Variable dépendante : Laser P2 Amplitude ΔT0-T2.

Matrice de corrélation pour les estimations des paramètres de covariance[a]

Paramètre	Résidu
Résidu	1

a. Variable dépendante : Laser P2 Amplitude ΔT0-T2.

Matrice de covariance pour les estimations des paramètres de covariance[a]

Paramètre	Résidu
Résidu	49,788994

a. Variable dépendante : Laser P2 Amplitude ΔT0-T2.

Moyenne marginale estimée

1. Grand Mean[a]

Moyenne	Erreur standard	ddl	Intervalle de confiance à 95 % Borne inférieure	Borne supérieure
,153	,857	42	-1,578	1,883

a. Variable dépendante : Laser P2 Amplitude ΔT0-T2.

2. Condition

Estimations[a]

Condition	Moyenne	Erreur standard	ddl	Intervalle de confiance à 95 % Borne inférieure	Intervalle de confiance à 95 % Borne supérieure
Sham	,500	1,212	42	-1,946	2,947
taVNS	-,195	1,212	42	-2,642	2,251

a. Variable dépendante : Laser P2 Amplitude ΔT0-T2.

Comparaisons appariées[a]

(I) Condition	(J) Condition	Différence moyenne (I-J)	Erreur standard	ddl	Sig.[b]
Sham	taVNS	,696	1,715	42	,687
taVNS	Sham	-,696	1,715	42	,687

Comparaisons appariées[a]

(I) Condition	(J) Condition	Intervalle de confiance à 95 % pour la différence[b] Borne inférieure	Intervalle de confiance à 95 % pour la différence[b] Borne supérieure
Sham	taVNS	-2,764	4,156
taVNS	Sham	-4,156	2,764

Basées sur les moyennes marginales estimées[a]

a. Variable dépendante : Laser P2 Amplitude ΔT0-T2.

b. Ajustement pour les comparaisons multiples : Bonferroni.

Tests univariés[a]

Ddl du numérateur	Ddl du dénominateur	F	Sig.
1	42	,165	,687

Le test de F permet de tester l'effet de Condition. Il s'appuie sur les comparaisons appariées (indépendantes) linéaires parmi les moyennes marginales estimées.[a]

a. Variable dépendante : Laser P2 Amplitude ΔT0-T2.

1.1.5. LEPs P2 Latency (ΔT0-T2).

```
MIXED LaserP2LatencyΔT0T2 BY Condition
  /CRITERIA=CIN(95) MXITER(100) MXSTEP(10) SCORING(1)
SINGULAR(0.000000000001) HCONVERGE(0,
    ABSOLUTE) LCONVERGE(0, ABSOLUTE) PCONVERGE(0.000001, ABSOLUTE)
  /FIXED=Condition | SSTYPE(3)
  /METHOD=REML
  /PRINT=CPS CORB COVB DESCRIPTIVES G  SOLUTION TESTCOV
  /EMMEANS=TABLES(OVERALL)
  /EMMEANS=TABLES(Condition) COMPARE ADJ(BONFERRONI).
```

Remarques

Sortie obtenue		05-MAY-2021 12:45:39
Commentaires		
Entrée	Jeu de données actif	Jeu_de_données4
	Filtre	<sans>
	Pondération	<sans>
	Fichier scindé	<sans>
	N de lignes dans le fichier de travail	44
Gestion des valeurs manquantes	Définition de la valeur manquante	Les valeurs manquantes définies par l'utilisateur sont traitées comme étant manquantes.
	Observations utilisées	Les statistiques sont basées sur toutes les observations comportant des données valides pour toutes les variables du modèle.

Syntaxe		MIXED LaserP2LatencyΔT0T2 BY Condition<br>/CRITERIA=CIN(95) MXITER(100) MXSTEP(10) SCORING(1) SINGULAR(0.000000000001) HCONVERGE(0, ABSOLUTE) LCONVERGE(0, ABSOLUTE) PCONVERGE(0.000001, ABSOLUTE)<br>/FIXED=Condition | SSTYPE(3)<br>/METHOD=REML<br>/PRINT=CPS CORB COVB DESCRIPTIVES G SOLUTION TESTCOV<br>/EMMEANS=TABLES(OVERALL)<br>/EMMEANS=TABLES(Condition) COMPARE ADJ(BONFERRONI).
Ressources	Temps de processeur	00:00:00,02
	Temps écoulé	00:00:00,01

Récapitulatif de traitement des observations

		Effectif	Pourcentage marginal
Condition	Sham	21	50,0%
	taVNS	21	50,0%
Valide		42	100,0%
Exclues		2	
Total		44	

Statistiques descriptives

Laser P2 Latency ΔT0-T2

Condition	Effectif	Moyenne	Ecart type	Coefficient de variation
Sham	21	,009333333333333	,041165815227362	441,1%
taVNS	21	-,000238095238095	,036399855716616	-15287,9%
Total	42	,004547619047619	,038683646687225	850,6%

Dimension du modèle[a]

		Nombre de niveaux	Nombre de paramètres
Effets fixes	Constante	1	1
	Condition	2	1
Résidu			1
Total		3	3

a. Variable dépendante : Laser P2 Latency ΔT0-T2.

Critères d'information[a]

Log de vraisemblance restreint -2	-140,227
Critère d'information d'Akaike (AIC)	-138,227
Critère de Hurvich et Tsai (AICC)	-138,122
Critère de Bozdogan (CAIC)	-135,538
Critère bayésien de Schwartz (BIC)	-136,538

Les critères d'informations sont présentés en plus petit, disposant d'un meilleur format.[a]

a. Variable dépendante : Laser P2 Latency ΔT0-T2.

Effets fixes

Tests des effets fixes de type III[a]

Source	Ddl du numérateur	Ddl du dénominateur	F	Sig.
Constante	1	40	,575	,453
Condition	1	40	,637	,429

a. Variable dépendante : Laser P2 Latency ΔT0-T2.

Estimations des effets fixes[a]

Paramètre	Estimation	Erreur standard	ddl	t	Sig.	Intervalle de confiance à 95 % Borne inférieure
Constante	-,000238	,008479	40	-,028	,978	-,017375
[Condition=Sham]	,009571	,011991	40	,798	,429	-,014664
[Condition=taVNS]	0[b]	0	.	.	.	.

Estimations des effets fixes[a]

Paramètre	Intervalle de confiance à 95 % Borne supérieure
Constante	,016899
[Condition=Sham]	,033807
[Condition=taVNS]	.

a. Variable dépendante : Laser P2 Latency ΔT0-T2.

b. Ce paramètre est défini sur 0, car il est redondant.

Matrice de corrélation pour les estimations des effets fixes[a]

Paramètre	Constante	[Condition=Sham]	[Condition=taVNS]
Constante	1	-,707	.[b]
[Condition=Sham]	-,707	1	.[b]
[Condition=taVNS]	.[b]	.[b]	.[b]

a. Variable dépendante : Laser P2 Latency ΔT0-T2.

b. La corrélation est manquante par défaut, car elle est associée à un paramètre redondant.

Matrice de covariance pour les estimations des effets fixes[a]

Paramètre	Constante	[Condition=Sham]	[Condition=taVNS]
Constante	7,189462E-5	-7,189462E-5	0[b]

[Condition=Sham]	-7,189462E-5	,000144	0[b]
[Condition=taVNS]	0[b]	0[b]	0[b]

a. Variable dépendante : Laser P2 Latency ΔT0-T2.

b. La covariance est définie sur 0, car elle est associée à un paramètre redondant.

Paramètres de covariance

Estimations des paramètres de covariance[a]

					Intervalle de confiance à 95 %	
Paramètre	Estimation	Erreur standard	Z de Wald	Sig.	Borne inférieure	Borne supérieure
Résidu	,001510	,000338	4,472	,000	,000974	,002340

a. Variable dépendante : Laser P2 Latency ΔT0-T2.

Matrice de corrélation pour les estimations des paramètres de covariance[a]

Paramètre	Résidu
Résidu	1

a. Variable dépendante : Laser P2 Latency ΔT0-T2.

Matrice de covariance pour les estimations des paramètres de covariance[a]

Paramètre	Résidu
Résidu	1,139728E-7

a. Variable dépendante : Laser P2 Latency ΔT0-T2.

Moyenne marginale estimée

1. Grand Mean[a]

			Intervalle de confiance à 95 %	
Moyenne	Erreur standard	ddl	Borne inférieure	Borne supérieure
,005	,006	40	-,008	,017

a. Variable dépendante : Laser P2 Latency ΔT0-T2.

2. Condition

Estimations[a]

Condition	Moyenne	Erreur standard	ddl	Intervalle de confiance à 95 %	
				Borne inférieure	Borne supérieure
Sham	,009	,008	40	-,008	,026
taVNS	,000	,008	40	-,017	,017

a. Variable dépendante : Laser P2 Latency ΔT0-T2.

Comparaisons appariées[a]

(I) Condition	(J) Condition	Différence moyenne (I-J)	Erreur standard	ddl	Sig.[b]
Sham	taVNS	,010	,012	40	,429
taVNS	Sham	-,010	,012	40	,429

Comparaisons appariées[a]

(I) Condition	(J) Condition	Intervalle de confiance à 95 % pour la différence[b]	
		Borne inférieure	Borne supérieure
Sham	taVNS	-,015	,034
taVNS	Sham	-,034	,015

Basées sur les moyennes marginales estimées[a]

a. Variable dépendante : Laser P2 Latency ΔT0-T2.

b. Ajustement pour les comparaisons multiples : Bonferroni.

Tests univariés[a]

Ddl du numérateur	Ddl du dénominateur	F	Sig.
1	40	,637	,429

Le test de F permet de tester l'effet de Condition. Il s'appuie sur les comparaisons appariées (indépendantes) linéaires parmi les moyennes marginales estimées.[a]

a. Variable dépendante : Laser P2 Latency ΔT0-T2.

1.2. Vibrotactile-evoked potentials.

1.2.1. Vibrotactile N2P2 Amplitude (ΔT0-T2).

```
MIXED VibrotactileN2P2AmplitudeΔT0T2 BY Condition
  /CRITERIA=CIN(95) MXITER(100) MXSTEP(10) SCORING(1)
SINGULAR(0.000000000001) HCONVERGE(0,
    ABSOLUTE) LCONVERGE(0, ABSOLUTE) PCONVERGE(0.000001, ABSOLUTE)
  /FIXED=Condition | SSTYPE(3)
  /METHOD=REML
  /PRINT=CPS CORB COVB DESCRIPTIVES G  SOLUTION TESTCOV
  /EMMEANS=TABLES(OVERALL)
  /EMMEANS=TABLES(Condition) COMPARE ADJ(BONFERRONI).
```

Remarques

Sortie obtenue		05-MAY-2021 12:46:21
Commentaires		
Entrée	Jeu de données actif	Jeu_de_données4
	Filtre	<sans>
	Pondération	<sans>
	Fichier scindé	<sans>
	N de lignes dans le fichier de travail	44
Gestion des valeurs manquantes	Définition de la valeur manquante	Les valeurs manquantes définies par l'utilisateur sont traitées comme étant manquantes.
	Observations utilisées	Les statistiques sont basées sur toutes les observations comportant des données valides pour toutes les variables du modèle.

Syntaxe		MIXED VibrotactileN2P2AmplitudeΔT0T2 BY Condition<br>/CRITERIA=CIN(95) MXITER(100) MXSTEP(10) SCORING(1) SINGULAR(0.000000000001) HCONVERGE(0,<br>ABSOLUTE) LCONVERGE(0, ABSOLUTE) PCONVERGE(0.000001, ABSOLUTE)<br>/FIXED=Condition | SSTYPE(3)<br>/METHOD=REML<br>/PRINT=CPS CORB COVB DESCRIPTIVES G SOLUTION TESTCOV<br><br>/EMMEANS=TABLES(OVERALL)<br><br>/EMMEANS=TABLES(Condition) COMPARE ADJ(BONFERRONI).
Ressources	Temps de processeur	00:00:00,02
	Temps écoulé	00:00:00,01

Récapitulatif de traitement des observations

		Effectif	Pourcentage marginal
Condition	Sham	21	50,0%
	taVNS	21	50,0%
Valide		42	100,0%
Exclues		2	
Total		44	

Statistiques descriptives

Vibrotactile N2P2 Amplitude ΔT0-T2

Condition	Effectif	Moyenne	Ecart type	Coefficient de variation
Sham	21	1,705383309523810	3,660099781966027	214,6%
taVNS	21	,920534238095238	3,844806637836445	417,7%
Total	42	1,312958773809524	3,728745439341015	284,0%

Dimension du modèle[a]

		Nombre de niveaux	Nombre de paramètres
Effets fixes	Constante	1	1
	Condition	2	1
Résidu			1
Total		3	3

a. Variable dépendante : Vibrotactile N2P2 Amplitude ΔT0-T2.

Critères d'information[a]

Log de vraisemblance restreint -2	225,421
Critère d'information d'Akaike (AIC)	227,421
Critère de Hurvich et Tsai (AICC)	227,526
Critère de Bozdogan (CAIC)	230,110
Critère bayésien de Schwartz (BIC)	229,110

Les critères d'informations sont présentés en plus petit, disposant d'un meilleur format.[a]

a. Variable dépendante : Vibrotactile N2P2 Amplitude ΔT0-T2.

Effets fixes

Tests des effets fixes de type III[a]

Source	Ddl du numérateur	Ddl du dénominateur	F	Sig.
Constante	1	40	5,139	,029
Condition	1	40	,459	,502

a. Variable dépendante : Vibrotactile N2P2 Amplitude ΔT0-T2.

Estimations des effets fixes[a]

Paramètre	Estimation	Erreur standard	ddl	t	Sig.	Intervalle de confiance à 95 % Borne inférieure
Constante	,920534	,819100	40	1,124	,268	-,734929
[Condition=Sham]	,784849	1,158383	40	,678	,502	-1,556330
[Condition=taVNS]	0[b]	0	.	.	.	.

Estimations des effets fixes[a]

Paramètre	Intervalle de confiance à 95 % Borne supérieure
Constante	2,575998
[Condition=Sham]	3,126028
[Condition=taVNS]	.

a. Variable dépendante : Vibrotactile N2P2 Amplitude ΔT0-T2.

b. Ce paramètre est défini sur 0, car il est redondant.

Matrice de corrélation pour les estimations des effets fixes[a]

Paramètre	Constante	[Condition=Sham]	[Condition=taVNS]
Constante	1	-,707	.[b]
[Condition=Sham]	-,707	1	.[b]
[Condition=taVNS]	.[b]	.[b]	.[b]

a. Variable dépendante : Vibrotactile N2P2 Amplitude ΔT0-T2.

b. La corrélation est manquante par défaut, car elle est associée à un paramètre redondant.

Matrice de covariance pour les estimations des effets fixes[a]

Paramètre	Constante	[Condition=Sham]	[Condition=taVNS]
Constante	,670925	-,670925	0[b]

[Condition=Sham]	-,670925	1,341851	0[b]
[Condition=taVNS]	0[b]	0[b]	0[b]

a. Variable dépendante : Vibrotactile N2P2 Amplitude ΔT0-T2.

b. La covariance est définie sur 0, car elle est associée à un paramètre redondant.

Paramètres de covariance

Estimations des paramètres de covariance[a]

					Intervalle de confiance à 95 %	
Paramètre	Estimation	Erreur standard	Z de Wald	Sig.	Borne inférieure	Borne supérieure
Résidu	14,089434	3,150493	4,472	,000	9,089900	21,838762

a. Variable dépendante : Vibrotactile N2P2 Amplitude ΔT0-T2.

Matrice de corrélation pour les estimations des paramètres de covariance[a]

Paramètre	Résidu
Résidu	1

a. Variable dépendante : Vibrotactile N2P2 Amplitude ΔT0-T2.

Matrice de covariance pour les estimations des paramètres de covariance[a]

Paramètre	Résidu
Résidu	9,925608

a. Variable dépendante : Vibrotactile N2P2 Amplitude ΔT0-T2.

Moyenne marginale estimée

1. Grand Mean[a]

Moyenne	Erreur standard	ddl	Intervalle de confiance à 95 % Borne inférieure	Borne supérieure
1,313	,579	40	,142	2,484

a. Variable dépendante : Vibrotactile N2P2 Amplitude ΔT0-T2.

2. Condition

Estimations[a]

Condition	Moyenne	Erreur standard	ddl	Intervalle de confiance à 95 % Borne inférieure	Borne supérieure
Sham	1,705	,819	40	,050	3,361
taVNS	,921	,819	40	-,735	2,576

a. Variable dépendante : Vibrotactile N2P2 Amplitude ΔT0-T2.

Comparaisons appariées[a]

(I) Condition	(J) Condition	Différence moyenne (I-J)	Erreur standard	ddl	Sig.[b]
Sham	taVNS	,785	1,158	40	,502
taVNS	Sham	-,785	1,158	40	,502

Comparaisons appariées[a]

(I) Condition	(J) Condition	Intervalle de confiance à 95 % pour la différence[b] Borne inférieure	Borne supérieure
Sham	taVNS	-1,556	3,126
taVNS	Sham	-3,126	1,556

Basées sur les moyennes marginales estimées[a]

a. Variable dépendante : Vibrotactile N2P2 Amplitude ΔT0-T2.

b. Ajustement pour les comparaisons multiples : Bonferroni.

Tests univariés[a]

Ddl du numérateur	Ddl du dénominateur	F	Sig.
1	40	,459	,502

Le test de F permet de tester l'effet de Condition. Il s'appuie sur les comparaisons appariées (indépendantes) linéaires parmi les moyennes marginales estimées.[a]

a. Variable dépendante : Vibrotactile N2P2 Amplitude ΔT0-T2.

1.2.2. Vibrotactile N2 Amplitude (ΔT0-T2).

```
MIXED VibrotactileN2AmplitudeΔT0T2 BY Condition
  /CRITERIA=CIN(95) MXITER(100) MXSTEP(10) SCORING(1)
SINGULAR(0.000000000001) HCONVERGE(0,
    ABSOLUTE) LCONVERGE(0, ABSOLUTE) PCONVERGE(0.000001, ABSOLUTE)
  /FIXED=Condition | SSTYPE(3)
  /METHOD=REML
  /PRINT=CPS CORB COVB DESCRIPTIVES G  SOLUTION TESTCOV
  /EMMEANS=TABLES(OVERALL)
  /EMMEANS=TABLES(Condition) COMPARE ADJ(BONFERRONI).
```

Remarques

Sortie obtenue		05-MAY-2021 12:47:02
Commentaires		
Entrée	Jeu de données actif	Jeu_de_données4
	Filtre	<sans>
	Pondération	<sans>
	Fichier scindé	<sans>
	N de lignes dans le fichier de travail	44
Gestion des valeurs manquantes	Définition de la valeur manquante	Les valeurs manquantes définies par l'utilisateur sont traitées comme étant manquantes.
	Observations utilisées	Les statistiques sont basées sur toutes les observations comportant des données valides pour toutes les variables du modèle.

Syntaxe		MIXED VibrotactileN2AmplitudeΔT0T2 BY Condition<br>/CRITERIA=CIN(95) MXITER(100) MXSTEP(10) SCORING(1) SINGULAR(0.000000000001) HCONVERGE(0, ABSOLUTE) LCONVERGE(0, ABSOLUTE) PCONVERGE(0.000001, ABSOLUTE)<br>/FIXED=Condition | SSTYPE(3)<br>/METHOD=REML<br>/PRINT=CPS CORB COVB DESCRIPTIVES G SOLUTION TESTCOV<br>/EMMEANS=TABLES(OVERALL)<br>/EMMEANS=TABLES(Condition) COMPARE ADJ(BONFERRONI).
Ressources	Temps de processeur	00:00:00,02
	Temps écoulé	00:00:00,02

Récapitulatif de traitement des observations

		Effectif	Pourcentage marginal
Condition	Sham	21	50,0%
	taVNS	21	50,0%
Valide		42	100,0%
Exclues		2	
Total		44	

Statistiques descriptives

Vibrotactile N2 Amplitude ΔT0-T2

Condition	Effectif	Moyenne	Ecart type	Coefficient de variation
Sham	21	,681112738095238	2,951637735567738	433,4%
taVNS	21	-,148070952380953	3,476274936875541	-2347,7%
Total	42	,266520892857143	3,212598233849018	1205,4%

Dimension du modèle[a]

		Nombre de niveaux	Nombre de paramètres
Effets fixes	Constante	1	1
	Condition	2	1
Résidu			1
Total		3	3

a. Variable dépendante : Vibrotactile N2 Amplitude ΔT0-T2.

Critères d'information[a]

Log de vraisemblance restreint -2	213,270
Critère d'information d'Akaike (AIC)	215,270
Critère de Hurvich et Tsai (AICC)	215,375
Critère de Bozdogan (CAIC)	217,959
Critère bayésien de Schwartz (BIC)	216,959

Les critères d'informations sont présentés en plus petit, disposant d'un meilleur format.[a]

a. Variable dépendante : Vibrotactile N2 Amplitude ΔT0-T2.

Effets fixes

Tests des effets fixes de type III[a]

Source	Ddl du numérateur	Ddl du dénominateur	F	Sig.
Constante	1	40	,287	,595
Condition	1	40	,694	,410

a. Variable dépendante : Vibrotactile N2 Amplitude ΔT0-T2.

Estimations des effets fixes[a]

Paramètre	Estimation	Erreur standard	ddl	t	Sig.	Intervalle de confiance à 95 % Borne inférieure
Constante	-,148071	,703675	40	-,210	,834	-1,570251
[Condition=Sham]	,829184	,995147	40	,833	,410	-1,182083
[Condition=taVNS]	0[b]	0	.	.	.	.

Estimations des effets fixes[a]

Paramètre	Intervalle de confiance à 95 % Borne supérieure
Constante	1,274109
[Condition=Sham]	2,840450
[Condition=taVNS]	.

a. Variable dépendante : Vibrotactile N2 Amplitude ΔT0-T2.

b. Ce paramètre est défini sur 0, car il est redondant.

Matrice de corrélation pour les estimations des effets fixes[a]

Paramètre	Constante	[Condition=Sham]	[Condition=taVNS]
Constante	1	-,707	.[b]
[Condition=Sham]	-,707	1	.[b]
[Condition=taVNS]	.[b]	.[b]	.[b]

a. Variable dépendante : Vibrotactile N2 Amplitude ΔT0-T2.

b. La corrélation est manquante par défaut, car elle est associée à un paramètre redondant.

Matrice de covariance pour les estimations des effets fixes[a]

Paramètre	Constante	[Condition=Sham]	[Condition=taVNS]
Constante	,495158	-,495158	0[b]
[Condition=Sham]	-,495158	,990317	0[b]
[Condition=taVNS]	0[b]	0[b]	0[b]

a. Variable dépendante : Vibrotactile N2 Amplitude ΔT0-T2.

b. La covariance est définie sur 0, car elle est associée à un paramètre redondant.

Paramètres de covariance

Estimations des paramètres de covariance[a]

Paramètre	Estimation	Erreur standard	Z de Wald	Sig.	Intervalle de confiance à 95 %	
					Borne inférieure	Borne supérieure
Résidu	10,398326	2,325136	4,472	,000	6,708555	16,117508

a. Variable dépendante : Vibrotactile N2 Amplitude ΔT0-T2.

Matrice de corrélation pour les estimations des paramètres de covariance[a]

Paramètre	Résidu
Résidu	1

a. Variable dépendante : Vibrotactile N2 Amplitude ΔT0-T2.

Matrice de covariance pour les estimations des paramètres de covariance[a]

Paramètre	Résidu
Résidu	5,406260

a. Variable dépendante : Vibrotactile N2 Amplitude ΔT0-T2.

Moyenne marginale estimée

1. Grand Mean[a]

Moyenne	Erreur standard	ddl	Intervalle de confiance à 95 %	
			Borne inférieure	Borne supérieure
,267	,498	40	-,739	1,272

a. Variable dépendante : Vibrotactile N2 Amplitude ΔT0-T2.

2. Condition

Estimations[a]

Condition	Moyenne	Erreur standard	ddl	Intervalle de confiance à 95 % Borne inférieure	Borne supérieure
Sham	,681	,704	40	-,741	2,103
taVNS	-,148	,704	40	-1,570	1,274

a. Variable dépendante : Vibrotactile N2 Amplitude ΔT0-T2.

Comparaisons appariées[a]

(I) Condition	(J) Condition	Différence moyenne (I-J)	Erreur standard	ddl	Sig.[b]
Sham	taVNS	,829	,995	40	,410
taVNS	Sham	-,829	,995	40	,410

Comparaisons appariées[a]

(I) Condition	(J) Condition	Intervalle de confiance à 95 % pour la différence[b] Borne inférieure	Borne supérieure
Sham	taVNS	-1,182	2,840
taVNS	Sham	-2,840	1,182

Basées sur les moyennes marginales estimées[a]

a. Variable dépendante : Vibrotactile N2 Amplitude ΔT0-T2.

b. Ajustement pour les comparaisons multiples : Bonferroni.

Tests univariés[a]

Ddl du numérateur	Ddl du dénominateur	F	Sig.
1	40	,694	,410

Le test de F permet de tester l'effet de Condition. Il s'appuie sur les comparaisons appariées (indépendantes) linéaires parmi les moyennes marginales estimées.[a]

a. Variable dépendante : Vibrotactile N2 Amplitude ΔT0-T2.

1.2.3. Vibrotactile N2 Latency (ΔT0-T2).

```
MIXED VibrotactileN2LatencyΔT0T2 BY Condition
  /CRITERIA=CIN(95) MXITER(100) MXSTEP(10) SCORING(1)
SINGULAR(0.000000000001) HCONVERGE(0,
    ABSOLUTE) LCONVERGE(0, ABSOLUTE) PCONVERGE(0.000001, ABSOLUTE)
  /FIXED=Condition | SSTYPE(3)
  /METHOD=REML
  /PRINT=CPS CORB COVB DESCRIPTIVES G  SOLUTION TESTCOV
  /EMMEANS=TABLES(OVERALL)
  /EMMEANS=TABLES(Condition) COMPARE ADJ(BONFERRONI).
```

Remarques

Sortie obtenue		05-MAY-2021 12:47:28
Commentaires		
Entrée	Jeu de données actif	Jeu_de_données4
	Filtre	<sans>
	Pondération	<sans>
	Fichier scindé	<sans>
	N de lignes dans le fichier de travail	44
Gestion des valeurs manquantes	Définition de la valeur manquante	Les valeurs manquantes définies par l'utilisateur sont traitées comme étant manquantes.
	Observations utilisées	Les statistiques sont basées sur toutes les observations comportant des données valides pour toutes les variables du modèle.

Syntaxe		MIXED VibrotactileN2LatencyΔT0T2 BY Condition<br>/CRITERIA=CIN(95) MXITER(100) MXSTEP(10) SCORING(1) SINGULAR(0.000000000001) HCONVERGE(0, ABSOLUTE) LCONVERGE(0, ABSOLUTE) PCONVERGE(0.000001, ABSOLUTE)<br>/FIXED=Condition | SSTYPE(3)<br>/METHOD=REML<br>/PRINT=CPS CORB COVB DESCRIPTIVES G SOLUTION TESTCOV<br>/EMMEANS=TABLES(OVERALL)<br>/EMMEANS=TABLES(Condition) COMPARE ADJ(BONFERRONI).
Ressources	Temps de processeur	00:00:00,00
	Temps écoulé	00:00:00,01

Récapitulatif de traitement des observations

		Effectif	Pourcentage marginal
Condition	Sham	20	50,0%
	taVNS	20	50,0%
Valide		40	100,0%
Exclues		4	
Total		44	

Statistiques descriptives

Vibrotactile N2 Latency ΔT0-T2

Condition	Effectif	Moyenne	Ecart type	Coefficient de variation
Sham	20	-,006500050000000	,028669630916566	-441,1%
taVNS	20	-,009500500000000	,055671258338572	-586,0%
Total	40	-,008000275000000	,043733905493240	-546,7%

Dimension du modèle[a]

		Nombre de niveaux	Nombre de paramètres
Effets fixes	Constante	1	1
	Condition	2	1
Résidu			1
Total		3	3

a. Variable dépendante : Vibrotactile N2 Latency ΔT0-T2.

Critères d'information[a]

Log de vraisemblance restreint -2	-123,080
Critère d'information d'Akaike (AIC)	-121,080
Critère de Hurvich et Tsai (AICC)	-120,969
Critère de Bozdogan (CAIC)	-118,442
Critère bayésien de Schwartz (BIC)	-119,442

Les critères d'informations sont présentés en plus petit, disposant d'un meilleur format.[a]

a. Variable dépendante : Vibrotactile N2 Latency ΔT0-T2.

Effets fixes

Tests des effets fixes de type III[a]

Source	Ddl du numérateur	Ddl du dénominateur	F	Sig.
Constante	1	38	1,306	,260
Condition	1	38	,046	,831

a. Variable dépendante : Vibrotactile N2 Latency ΔT0-T2.

Estimations des effets fixes[a]

Paramètre	Estimation	Erreur standard	ddl	t	Sig.	Intervalle de confiance à 95 % Borne inférieure
Constante	-,009501	,009901	38	-,960	,343	-,029544
[Condition=Sham]	,003000	,014002	38	,214	,831	-,025346
[Condition=taVNS]	0[b]	0	.	.	.	.

Estimations des effets fixes[a]

Paramètre	Intervalle de confiance à 95 % Borne supérieure
Constante	,010543
[Condition=Sham]	,031346
[Condition=taVNS]	.

a. Variable dépendante : Vibrotactile N2 Latency ΔT0-T2.

b. Ce paramètre est défini sur 0, car il est redondant.

Matrice de corrélation pour les estimations des effets fixes[a]

Paramètre	Constante	[Condition=Sham]	[Condition=taVNS]
Constante	1	-,707	.[b]
[Condition=Sham]	-,707	1	.[b]
[Condition=taVNS]	.[b]	.[b]	.[b]

a. Variable dépendante : Vibrotactile N2 Latency ΔT0-T2.

b. La corrélation est manquante par défaut, car elle est associée à un paramètre redondant.

Matrice de covariance pour les estimations des effets fixes[a]

Paramètre	Constante	[Condition=Sham]	[Condition=taVNS]
Constante	9,803092E-5	-9,803092E-5	0[b]
[Condition=Sham]	-9,803092E-5	,000196	0[b]

[Condition=taVNS]	0[b]	0[b]	0[b]

a. Variable dépendante : Vibrotactile N2 Latency ΔT0-T2.

b. La covariance est définie sur 0, car elle est associée à un paramètre redondant.

Paramètres de covariance

Estimations des paramètres de covariance[a]

Paramètre	Estimation	Erreur standard	Z de Wald	Sig.	Intervalle de confiance à 95 % Borne inférieure	Intervalle de confiance à 95 % Borne supérieure
Résidu	,001961	,000450	4,359	,000	,001251	,003074

a. Variable dépendante : Vibrotactile N2 Latency ΔT0-T2.

Matrice de corrélation pour les estimations des paramètres de covariance[a]

Paramètre	Résidu
Résidu	1

a. Variable dépendante : Vibrotactile N2 Latency ΔT0-T2.

Matrice de covariance pour les estimations des paramètres de covariance[a]

Paramètre	Résidu
Résidu	2,023171E-7

a. Variable dépendante : Vibrotactile N2 Latency ΔT0-T2.

Moyenne marginale estimée

1. Grand Mean[a]

Moyenne	Erreur standard	ddl	Intervalle de confiance à 95 % Borne inférieure	Borne supérieure
-,008	,007	38	-,022	,006

a. Variable dépendante : Vibrotactile N2 Latency ΔT0-T2.

2. Condition

Estimations[a]

Condition	Moyenne	Erreur standard	ddl	Intervalle de confiance à 95 % Borne inférieure	Borne supérieure
Sham	-,007	,010	38	-,027	,014
taVNS	-,010	,010	38	-,030	,011

a. Variable dépendante : Vibrotactile N2 Latency ΔT0-T2.

Comparaisons appariées[a]

(I) Condition	(J) Condition	Différence moyenne (I-J)	Erreur standard	ddl	Sig.[b]
Sham	taVNS	,003	,014	38	,831
taVNS	Sham	-,003	,014	38	,831

Comparaisons appariées[a]

(I) Condition	(J) Condition	Intervalle de confiance à 95 % pour la différence[b] Borne inférieure	Borne supérieure
Sham	taVNS	-,025	,031
taVNS	Sham	-,031	,025

Basées sur les moyennes marginales estimées[a]

a. Variable dépendante : Vibrotactile N2 Latency ΔT0-T2.

b. Ajustement pour les comparaisons multiples : Bonferroni.

Tests univariés[a]

Ddl du numérateur	Ddl du dénominateur	F	Sig.
1	38	,046	,831

Le test de F permet de tester l'effet de Condition. Il s'appuie sur les comparaisons appariées (indépendantes) linéaires parmi les moyennes marginales estimées.[a]

a. Variable dépendante : Vibrotactile N2 Latency ΔT0-T2.

1.2.4. Vibrotactile P2 Amplitude (ΔT0-T2).

```
MIXED VibrotactileP2AmplitudeΔT0T2 BY Condition
  /CRITERIA=CIN(95) MXITER(100) MXSTEP(10) SCORING(1)
SINGULAR(0.000000000001) HCONVERGE(0,
    ABSOLUTE) LCONVERGE(0, ABSOLUTE) PCONVERGE(0.000001, ABSOLUTE)
  /FIXED=Condition | SSTYPE(3)
  /METHOD=REML
  /PRINT=CPS CORB COVB DESCRIPTIVES G  SOLUTION TESTCOV
  /EMMEANS=TABLES(OVERALL)
  /EMMEANS=TABLES(Condition) COMPARE ADJ(BONFERRONI).
```

Remarques

Sortie obtenue		05-MAY-2021 12:47:53
Commentaires		
Entrée	Jeu de données actif	Jeu_de_données4
	Filtre	<sans>
	Pondération	<sans>
	Fichier scindé	<sans>
	N de lignes dans le fichier de travail	44
Gestion des valeurs manquantes	Définition de la valeur manquante	Les valeurs manquantes définies par l'utilisateur sont traitées comme étant manquantes.
	Observations utilisées	Les statistiques sont basées sur toutes les observations comportant des données valides pour toutes les variables du modèle.

Syntaxe		MIXED VibrotactileP2AmplitudeΔT0T2 BY Condition<br>/CRITERIA=CIN(95) MXITER(100) MXSTEP(10) SCORING(1) SINGULAR(0.000000000001) HCONVERGE(0, ABSOLUTE) LCONVERGE(0, ABSOLUTE) PCONVERGE(0.000001, ABSOLUTE)<br>/FIXED=Condition | SSTYPE(3)<br>/METHOD=REML<br>/PRINT=CPS CORB COVB DESCRIPTIVES G SOLUTION TESTCOV<br>/EMMEANS=TABLES(OVERALL)<br>/EMMEANS=TABLES(Condition) COMPARE ADJ(BONFERRONI).
Ressources	Temps de processeur	00:00:00,02
	Temps écoulé	00:00:00,01

Récapitulatif de traitement des observations

		Effectif	Pourcentage marginal
Condition	Sham	21	50,0%
	taVNS	21	50,0%
Valide		42	100,0%
Exclues		2	
Total		44	

Statistiques descriptives

Vibrotactile P2 Amplitude ΔT0-T2

Condition	Effectif	Moyenne	Ecart type	Coefficient de variation
Sham	21	2,203742714285714	4,264250178185693	193,5%
taVNS	21	,775068523809524	3,455540605434374	445,8%
Total	42	1,489405619047619	3,900980063084348	261,9%

Dimension du modèle[a]

		Nombre de niveaux	Nombre de paramètres
Effets fixes	Constante	1	1
	Condition	2	1
Résidu			1
Total		3	3

a. Variable dépendante : Vibrotactile P2 Amplitude ΔT0-T2.

Critères d'information[a]

Log de vraisemblance restreint -2	228,092
Critère d'information d'Akaike (AIC)	230,092
Critère de Hurvich et Tsai (AICC)	230,197
Critère de Bozdogan (CAIC)	232,781
Critère bayésien de Schwartz (BIC)	231,781

Les critères d'informations sont présentés en plus petit, disposant d'un meilleur format.[a]

a. Variable dépendante : Vibrotactile P2 Amplitude ΔT0-T2.

Effets fixes

Tests des effets fixes de type III[a]

Source	Ddl du numérateur	Ddl du dénominateur	F	Sig.
Constante	1	40	6,186	,017
Condition	1	40	1,423	,240

a. Variable dépendante : Vibrotactile P2 Amplitude ΔT0-T2.

Estimations des effets fixes[a]

Paramètre	Estimation	Erreur standard	ddl	t	Sig.	Intervalle de confiance à 95 % Borne inférieure
Constante	,775069	,846907	40	,915	,366	-,936595
[Condition=Sham]	1,428674	1,197708	40	1,193	,240	-,991984
[Condition=taVNS]	0[b]	0	.	.	.	.

Estimations des effets fixes[a]

Paramètre	Intervalle de confiance à 95 % Borne supérieure
Constante	2,486732
[Condition=Sham]	3,849332
[Condition=taVNS]	.

a. Variable dépendante : Vibrotactile P2 Amplitude ΔT0-T2.

b. Ce paramètre est défini sur 0, car il est redondant.

Matrice de corrélation pour les estimations des effets fixes[a]

Paramètre	Constante	[Condition=Sham]	[Condition=taVNS]
Constante	1	-,707	.[b]
[Condition=Sham]	-,707	1	.[b]
[Condition=taVNS]	.[b]	.[b]	.[b]

a. Variable dépendante : Vibrotactile P2 Amplitude ΔT0-T2.

b. La corrélation est manquante par défaut, car elle est associée à un paramètre redondant.

Matrice de covariance pour les estimations des effets fixes[a]

Paramètre	Constante	[Condition=Sham]	[Condition=taVNS]
Constante	,717252	-,717252	0[b]

[Condition=Sham]	-,717252	1,434504	0[b]
[Condition=taVNS]	0[b]	0[b]	0[b]

a. Variable dépendante : Vibrotactile P2 Amplitude ΔT0-T2.

b. La covariance est définie sur 0, car elle est associée à un paramètre redondant.

Paramètres de covariance

Estimations des paramètres de covariance[a]

Paramètre	Estimation	Erreur standard	Z de Wald	Sig.	Intervalle de confiance à 95 % Borne inférieure	Intervalle de confiance à 95 % Borne supérieure
Résidu	15,062295	3,368032	4,472	,000	9,717548	23,346706

a. Variable dépendante : Vibrotactile P2 Amplitude ΔT0-T2.

Matrice de corrélation pour les estimations des paramètres de covariance[a]

Paramètre	Résidu
Résidu	1

a. Variable dépendante : Vibrotactile P2 Amplitude ΔT0-T2.

Matrice de covariance pour les estimations des paramètres de covariance[a]

Paramètre	Résidu
Résidu	11,343637

a. Variable dépendante : Vibrotactile P2 Amplitude ΔT0-T2.

Moyenne marginale estimée

1. Grand Mean[a]

Moyenne	Erreur standard	ddl	Intervalle de confiance à 95 %

			Borne inférieure	Borne supérieure
1,489	,599	40	,279	2,700

a. Variable dépendante : Vibrotactile P2 Amplitude ΔT0-T2.

2. Condition

Estimations[a]

Condition	Moyenne	Erreur standard	ddl	Intervalle de confiance à 95 % Borne inférieure	Borne supérieure
Sham	2,204	,847	40	,492	3,915
taVNS	,775	,847	40	-,937	2,487

a. Variable dépendante : Vibrotactile P2 Amplitude ΔT0-T2.

Comparaisons appariées[a]

(I) Condition	(J) Condition	Différence moyenne (I-J)	Erreur standard	ddl	Sig.[b]
Sham	taVNS	1,429	1,198	40	,240
taVNS	Sham	-1,429	1,198	40	,240

Comparaisons appariées[a]

(I) Condition	(J) Condition	Intervalle de confiance à 95 % pour la différence[b] Borne inférieure	Borne supérieure
Sham	taVNS	-,992	3,849
taVNS	Sham	-3,849	,992

Basées sur les moyennes marginales estimées[a]

a. Variable dépendante : Vibrotactile P2 Amplitude ΔT0-T2.

b. Ajustement pour les comparaisons multiples : Bonferroni.

Tests univariés[a]

Ddl du numérateur	Ddl du dénominateur	F	Sig.
1	40	1,423	,240

Le test de F permet de tester l'effet de Condition. Il s'appuie sur les comparaisons appariées (indépendantes) linéaires parmi les moyennes marginales estimées.[a]

a. Variable dépendante : Vibrotactile P2 Amplitude ΔT0-T2.

1.2.5. Vibrotactile-ERPs_P2 Latency (ΔT0-T2).

```
MIXED VibrotactileP2LatencyΔT0T2 BY Condition
  /CRITERIA=CIN(95) MXITER(100) MXSTEP(10) SCORING(1)
SINGULAR(0.000000000001) HCONVERGE(0,
    ABSOLUTE) LCONVERGE(0, ABSOLUTE) PCONVERGE(0.000001, ABSOLUTE)
  /FIXED=Condition | SSTYPE(3)
  /METHOD=REML
  /PRINT=CPS CORB COVB DESCRIPTIVES G  SOLUTION TESTCOV
  /EMMEANS=TABLES(OVERALL)
  /EMMEANS=TABLES(Condition) COMPARE ADJ(BONFERRONI).
```

Remarques

Sortie obtenue		05-MAY-2021 12:48:19
Commentaires		
Entrée	Jeu de données actif	Jeu_de_données4
	Filtre	<sans>
	Pondération	<sans>
	Fichier scindé	<sans>
	N de lignes dans le fichier de travail	44
Gestion des valeurs manquantes	Définition de la valeur manquante	Les valeurs manquantes définies par l'utilisateur sont traitées comme étant manquantes.
	Observations utilisées	Les statistiques sont basées sur toutes les observations comportant des données valides pour toutes les variables du modèle.

Syntaxe		MIXED VibrotactileP2LatencyΔT0T2 BY Condition<br>/CRITERIA=CIN(95) MXITER(100) MXSTEP(10) SCORING(1) SINGULAR(0.000000000001) HCONVERGE(0, ABSOLUTE) LCONVERGE(0, ABSOLUTE) PCONVERGE(0.000001, ABSOLUTE)<br>/FIXED=Condition | SSTYPE(3)<br>/METHOD=REML<br>/PRINT=CPS CORB COVB DESCRIPTIVES G SOLUTION TESTCOV<br>/EMMEANS=TABLES(OVERALL)<br>/EMMEANS=TABLES(Condition) COMPARE ADJ(BONFERRONI).
Ressources	Temps de processeur	00:00:00,02
	Temps écoulé	00:00:00,01

Récapitulatif de traitement des observations

		Effectif	Pourcentage marginal
Condition	Sham	20	50,0%
	taVNS	20	50,0%
Valide		40	100,0%
Exclues		4	
Total		44	

Statistiques descriptives

Vibrotactile P2 Latency ΔT0-T2

Condition	Effectif	Moyenne	Ecart type	Coefficient de variation
Sham	20	,009949500000000	,061078659247943	613,9%
taVNS	20	,032851000000000	,080487121290826	245,0%
Total	40	,021400250000000	,071470200693124	334,0%

Dimension du modèle[a]

		Nombre de niveaux	Nombre de paramètres
Effets fixes	Constante	1	1
	Condition	2	1
Résidu			1
Total		3	3

a. Variable dépendante : Vibrotactile P2 Latency ΔT0-T2.

Critères d'information[a]

Log de vraisemblance restreint -2	-86,720
Critère d'information d'Akaike (AIC)	-84,720
Critère de Hurvich et Tsai (AICC)	-84,609
Critère de Bozdogan (CAIC)	-82,082
Critère bayésien de Schwartz (BIC)	-83,082

Les critères d'informations sont présentés en plus petit, disposant d'un meilleur format.[a]

a. Variable dépendante : Vibrotactile P2 Latency ΔT0-T2.

Effets fixes

Tests des effets fixes de type III[a]

Source	Ddl du numérateur	Ddl du dénominateur	F	Sig.
Constante	1	38	3,589	,066
Condition	1	38	1,028	,317

a. Variable dépendante : Vibrotactile P2 Latency ΔT0-T2.

Estimations des effets fixes[a]

Paramètre	Estimation	Erreur standard	ddl	t	Sig.	Intervalle de confiance à 95 % Borne inférieure
Constante	,032851	,015976	38	2,056	,047	,000510
[Condition=Sham]	-,022902	,022593	38	-1,014	,317	-,068638
[Condition=taVNS]	0[b]	0	.	.	.	.

Estimations des effets fixes[a]

Paramètre	Intervalle de confiance à 95 % Borne supérieure
Constante	,065192
[Condition=Sham]	,022835
[Condition=taVNS]	.

a. Variable dépendante : Vibrotactile P2 Latency ΔT0-T2.

b. Ce paramètre est défini sur 0, car il est redondant.

Matrice de corrélation pour les estimations des effets fixes[a]

Paramètre	Constante	[Condition=Sham]	[Condition=taVNS]
Constante	1	-,707	.[b]
[Condition=Sham]	-,707	1	.[b]
[Condition=taVNS]	.[b]	.[b]	.[b]

a. Variable dépendante : Vibrotactile P2 Latency ΔT0-T2.

b. La corrélation est manquante par défaut, car elle est associée à un paramètre redondant.

Matrice de covariance pour les estimations des effets fixes[a]

Paramètre	Constante	[Condition=Sham]	[Condition=taVNS]
Constante	,000255	-,000255	0[b]

[Condition=Sham]	-,000255	,000510	0[b]
[Condition=taVNS]	0[b]	0[b]	0[b]

a. Variable dépendante : Vibrotactile P2 Latency ΔT0-T2.

b. La covariance est définie sur 0, car elle est associée à un paramètre redondant.

Paramètres de covariance

Estimations des paramètres de covariance[a]

Paramètre	Estimation	Erreur standard	Z de Wald	Sig.	Intervalle de confiance à 95 % Borne inférieure	Borne supérieure
Résidu	,005104	,001171	4,359	,000	,003256	,008002

a. Variable dépendante : Vibrotactile P2 Latency ΔT0-T2.

Matrice de corrélation pour les estimations des paramètres de covariance[a]

Paramètre	Résidu
Résidu	1

a. Variable dépendante : Vibrotactile P2 Latency ΔT0-T2.

Matrice de covariance pour les estimations des paramètres de covariance[a]

Paramètre	Résidu
Résidu	1,371305E-6

a. Variable dépendante : Vibrotactile P2 Latency ΔT0-T2.

Moyenne marginale estimée

1. Grand Mean[a]

Moyenne	Erreur standard	ddl	Intervalle de confiance à 95 % Borne inférieure	Intervalle de confiance à 95 % Borne supérieure
,021	,011	38	-,001	,044

a. Variable dépendante : Vibrotactile P2 Latency ΔT0-T2.

2. Condition

Estimations[a]

Condition	Moyenne	Erreur standard	ddl	Intervalle de confiance à 95 % Borne inférieure	Intervalle de confiance à 95 % Borne supérieure
Sham	,010	,016	38	-,022	,042
taVNS	,033	,016	38	,001	,065

a. Variable dépendante : Vibrotactile P2 Latency ΔT0-T2.

Comparaisons appariées[a]

(I) Condition	(J) Condition	Différence moyenne (I-J)	Erreur standard	ddl	Sig.[b]
Sham	taVNS	-,023	,023	38	,317
taVNS	Sham	,023	,023	38	,317

Comparaisons appariées[a]

(I) Condition	(J) Condition	Intervalle de confiance à 95 % pour la différence[b] Borne inférieure	Intervalle de confiance à 95 % pour la différence[b] Borne supérieure
Sham	taVNS	-,069	,023
taVNS	Sham	-,023	,069

Basées sur les moyennes marginales estimées[a]

a. Variable dépendante : Vibrotactile P2 Latency ΔT0-T2.

b. Ajustement pour les comparaisons multiples : Bonferroni.

Tests univariés[a]

Ddl du numérateur	Ddl du dénominateur	F	Sig.
1	38	1,028	,317

Le test de F permet de tester l'effet de Condition. Il s'appuie sur les comparaisons appariées (indépendantes) linéaires parmi les moyennes marginales estimées.[a]

a. Variable dépendante : Vibrotactile P2 Latency ΔT0-T2.

1.3. Cool-evoked potentials

1.3.1. Cool N2P2 Amplitude (ΔT0-T2).

```
MIXED CoolN2P2AmplitudeΔT0T2 BY Condition
  /CRITERIA=CIN(95) MXITER(100) MXSTEP(10) SCORING(1)
SINGULAR(0.000000000001) HCONVERGE(0,
    ABSOLUTE) LCONVERGE(0, ABSOLUTE) PCONVERGE(0.000001, ABSOLUTE)
  /FIXED=Condition | SSTYPE(3)
  /METHOD=REML
  /PRINT=CPS CORB COVB DESCRIPTIVES G  SOLUTION TESTCOV
  /EMMEANS=TABLES(OVERALL)
  /EMMEANS=TABLES(Condition) COMPARE ADJ(BONFERRONI)
```

Remarques

Sortie obtenue		05-MAY-2021 12:48:56
Commentaires		
Entrée	Jeu de données actif	Jeu_de_données4
	Filtre	<sans>
	Pondération	<sans>
	Fichier scindé	<sans>
	N de lignes dans le fichier de travail	44
Gestion des valeurs manquantes	Définition de la valeur manquante	Les valeurs manquantes définies par l'utilisateur sont traitées comme étant manquantes.
	Observations utilisées	Les statistiques sont basées sur toutes les observations comportant des données valides pour toutes les variables du modèle.

Syntaxe		MIXED CoolN2P2AmplitudeΔT0T2 BY Condition /CRITERIA=CIN(95) MXITER(100) MXSTEP(10) SCORING(1) SINGULAR(0.000000000001) HCONVERGE(0, ABSOLUTE) LCONVERGE(0, ABSOLUTE) PCONVERGE(0.000001, ABSOLUTE) /FIXED=Condition | SSTYPE(3) /METHOD=REML /PRINT=CPS CORB COVB DESCRIPTIVES G SOLUTION TESTCOV /EMMEANS=TABLES(OVERALL) /EMMEANS=TABLES(Condition) COMPARE ADJ(BONFERRONI).
Ressources	Temps de processeur	00:00:00,02
	Temps écoulé	00:00:00,01

Récapitulatif de traitement des observations

		Effectif	Pourcentage marginal
Condition	Sham	22	51,2%
	taVNS	21	48,8%
Valide		43	100,0%
Exclues		1	
Total		44	

Statistiques descriptives

Cool N2P2 Amplitude ΔT0-T2

Condition	Effectif	Moyenne	Ecart type	Coefficient de variation
Sham	22	,267732727272727	4,073520280582388	1521,5%
taVNS	21	,380755714285714	4,377775662226848	1149,8%
Total	43	,322930000000000	4,174470483422048	1292,7%

Dimension du modèle[a]

		Nombre de niveaux	Nombre de paramètres
Effets fixes	Constante	1	1
	Condition	2	1
Résidu			1
Total		3	3

a. Variable dépendante : Cool N2P2 Amplitude ΔT0-T2.

Critères d'information[a]

Log de vraisemblance restreint -2	240,646
Critère d'information d'Akaike (AIC)	242,646
Critère de Hurvich et Tsai (AICC)	242,748
Critère de Bozdogan (CAIC)	245,359
Critère bayésien de Schwartz (BIC)	244,359

Les critères d'informations sont présentés en plus petit, disposant d'un meilleur format.[a]

a. Variable dépendante : Cool N2P2 Amplitude ΔT0-T2.

Effets fixes

Tests des effets fixes de type III[a]

Source	Ddl du numérateur	Ddl du dénominateur	F	Sig.
Constante	1	41	,253	,618
Condition	1	41	,008	,931

a. Variable dépendante : Cool N2P2 Amplitude ΔT0-T2.

Estimations des effets fixes[a]

Paramètre	Estimation	Erreur standard	ddl	t	Sig.	Intervalle de confiance à 95 % Borne inférieure
Constante	,380756	,921900	41	,413	,682	-1,481059
[Condition=Sham]	-,113023	1,288863	41	-,088	,931	-2,715934
[Condition=taVNS]	0[b]	0	.	.	.	.

Estimations des effets fixes[a]

Paramètre	Intervalle de confiance à 95 % Borne supérieure
Constante	2,242570
[Condition=Sham]	2,489888
[Condition=taVNS]	.

a. Variable dépendante : Cool N2P2 Amplitude ΔT0-T2.

b. Ce paramètre est défini sur 0, car il est redondant.

Matrice de corrélation pour les estimations des effets fixes[a]

Paramètre	Constante	[Condition=Sham]	[Condition=taVNS]
Constante	1	-,715	.[b]
[Condition=Sham]	-,715	1	.[b]
[Condition=taVNS]	.[b]	.[b]	.[b]

a. Variable dépendante : Cool N2P2 Amplitude ΔT0-T2.

b. La corrélation est manquante par défaut, car elle est associée à un paramètre redondant.

Matrice de covariance pour les estimations des effets fixes[a]

Paramètre	Constante	[Condition=Sham]	[Condition=taVNS]
Constante	,849899	-,849899	0[b]

[Condition=Sham]	-,849899	1,661167	0[b]
[Condition=taVNS]	0[b]	0[b]	0[b]

a. Variable dépendante : Cool N2P2 Amplitude ΔT0-T2.

b. La covariance est définie sur 0, car elle est associée à un paramètre redondant.

Paramètres de covariance

Estimations des paramètres de covariance[a]

Paramètre	Estimation	Erreur standard	Z de Wald	Sig.	Intervalle de confiance à 95 % Borne inférieure	Borne supérieure
Résidu	17,847886	3,941939	4,528	,000	11,576781	27,516028

a. Variable dépendante : Cool N2P2 Amplitude ΔT0-T2.

Matrice de corrélation pour les estimations des paramètres de covariance[a]

Paramètre	Résidu
Résidu	1

a. Variable dépendante : Cool N2P2 Amplitude ΔT0-T2.

Matrice de covariance pour les estimations des paramètres de covariance[a]

Paramètre	Résidu
Résidu	15,538879

a. Variable dépendante : Cool N2P2 Amplitude ΔT0-T2.

Moyenne marginale estimée

1. Grand Mean[a]

Moyenne	Erreur standard	ddl	Intervalle de confiance à 95 % Borne inférieure	Borne supérieure
,324	,644	41	-,977	1,626

a. Variable dépendante : Cool N2P2 Amplitude ΔT0-T2.

2. Condition

Estimations[a]

Condition	Moyenne	Erreur standard	ddl	Intervalle de confiance à 95 % Borne inférieure	Borne supérieure
Sham	,268	,901	41	-1,551	2,087
taVNS	,381	,922	41	-1,481	2,243

a. Variable dépendante : Cool N2P2 Amplitude ΔT0-T2.

Comparaisons appariées[a]

(I) Condition	(J) Condition	Différence moyenne (I-J)	Erreur standard	ddl	Sig.[b]
Sham	taVNS	-,113	1,289	41	,931
taVNS	Sham	,113	1,289	41	,931

Comparaisons appariées[a]

(I) Condition	(J) Condition	Intervalle de confiance à 95 % pour la différence[b] Borne inférieure	Borne supérieure
Sham	taVNS	-2,716	2,490
taVNS	Sham	-2,490	2,716

Basées sur les moyennes marginales estimées[a]

a. Variable dépendante : Cool N2P2 Amplitude ΔT0-T2.

b. Ajustement pour les comparaisons multiples : Bonferroni.

Tests univariés[a]

Ddl du numérateur	Ddl du dénominateur	F	Sig.
1	41	,008	,931

Le test de F permet de tester l'effet de Condition. Il s'appuie sur les comparaisons appariées (indépendantes) linéaires parmi les moyennes marginales estimées.[a]

a. Variable dépendante : Cool N2P2 Amplitude ΔT0-T2.

1.3.2. Cool N2 Amplitude (ΔT0-T2).

```
MIXED CoolN2AmplitudeΔT0T2 BY Condition
  /CRITERIA=CIN(95) MXITER(100) MXSTEP(10) SCORING(1)
SINGULAR(0.000000000001) HCONVERGE(0,
    ABSOLUTE) LCONVERGE(0, ABSOLUTE) PCONVERGE(0.000001, ABSOLUTE)
  /FIXED=Condition | SSTYPE(3)
  /METHOD=REML
  /PRINT=CPS CORB COVB DESCRIPTIVES G  SOLUTION TESTCOV
  /EMMEANS=TABLES(OVERALL)
  /EMMEANS=TABLES(Condition) COMPARE ADJ(BONFERRONI).
```

Remarques

Sortie obtenue		05-MAY-2021 12:49:46
Commentaires		
Entrée	Jeu de données actif	Jeu_de_données4
	Filtre	<sans>
	Pondération	<sans>
	Fichier scindé	<sans>
	N de lignes dans le fichier de travail	44
Gestion des valeurs manquantes	Définition de la valeur manquante	Les valeurs manquantes définies par l'utilisateur sont traitées comme étant manquantes.
	Observations utilisées	Les statistiques sont basées sur toutes les observations comportant des données valides pour toutes les variables du modèle.

Syntaxe		MIXED CoolN2AmplitudeΔT0T2 BY Condition<br>/CRITERIA=CIN(95) MXITER(100) MXSTEP(10) SCORING(1) SINGULAR(0.000000000001) HCONVERGE(0, ABSOLUTE) LCONVERGE(0, ABSOLUTE) PCONVERGE(0.000001, ABSOLUTE)<br>/FIXED=Condition | SSTYPE(3)<br>/METHOD=REML<br>/PRINT=CPS CORB COVB DESCRIPTIVES G SOLUTION TESTCOV<br>/EMMEANS=TABLES(OVERALL)<br>/EMMEANS=TABLES(Condition) COMPARE ADJ(BONFERRONI).
Ressources	Temps de processeur	00:00:00,00
	Temps écoulé	00:00:00,01

Récapitulatif de traitement des observations

		Effectif	Pourcentage marginal
Condition	Sham	21	50,0%
	taVNS	21	50,0%
Valide		42	100,0%
Exclues		2	
Total		44	

Statistiques descriptives

Cool N2 Amplitude ΔT0-T2

Condition	Effectif	Moyenne	Ecart type	Coefficient de variation
Sham	21	1,152464285714286	3,686786034288634	319,9%
taVNS	21	,505338571428571	3,428270420118993	678,4%
Total	42	,828901428571428	3,531411966171930	426,0%

Dimension du modèle[a]

		Nombre de niveaux	Nombre de paramètres
Effets fixes	Constante	1	1
	Condition	2	1
Résidu			1
Total		3	3

a. Variable dépendante : Cool N2 Amplitude ΔT0-T2.

Critères d'information[a]

Log de vraisemblance restreint -2	221,182
Critère d'information d'Akaike (AIC)	223,182
Critère de Hurvich et Tsai (AICC)	223,287
Critère de Bozdogan (CAIC)	225,871
Critère bayésien de Schwartz (BIC)	224,871

Les critères d'informations sont présentés en plus petit, disposant d'un meilleur format.[a]

a. Variable dépendante : Cool N2 Amplitude ΔT0-T2.

Effets fixes

Tests des effets fixes de type III[a]

Source	Ddl du numérateur	Ddl du dénominateur	F	Sig.
Constante	1	40	2,277	,139
Condition	1	40	,347	,559

a. Variable dépendante : Cool N2 Amplitude ΔT0-T2.

Estimations des effets fixes[a]

Paramètre	Estimation	Erreur standard	ddl	t	Sig.	Intervalle de confiance à 95 %
						Borne inférieure
Constante	,505339	,776829	40	,651	,519	-1,064691
[Condition=Sham]	,647126	1,098601	40	,589	,559	-1,573231
[Condition=taVNS]	0[b]	0	.	.	.	.

Estimations des effets fixes[a]

Paramètre	Intervalle de confiance à 95 %
	Borne supérieure
Constante	2,075368
[Condition=Sham]	2,867482
[Condition=taVNS]	.

a. Variable dépendante : Cool N2 Amplitude ΔT0-T2.

b. Ce paramètre est défini sur 0, car il est redondant.

Matrice de corrélation pour les estimations des effets fixes[a]

Paramètre	Constante	[Condition=Sham]	[Condition=taVNS]
Constante	1	-,707	.[b]
[Condition=Sham]	-,707	1	.[b]
[Condition=taVNS]	.[b]	.[b]	.[b]

a. Variable dépendante : Cool N2 Amplitude ΔT0-T2.

b. La corrélation est manquante par défaut, car elle est associée à un paramètre redondant.

Matrice de covariance pour les estimations des effets fixes[a]

Paramètre	Constante	[Condition=Sham]	[Condition=taVNS]
Constante	,603463	-,603463	0[b]
[Condition=Sham]	-,603463	1,206925	0[b]
[Condition=taVNS]	0[b]	0[b]	0[b]

a. Variable dépendante : Cool N2 Amplitude ΔT0-T2.

b. La covariance est définie sur 0, car elle est associée à un paramètre redondant.

Paramètres de covariance

Estimations des paramètres de covariance[a]

Paramètre	Estimation	Erreur standard	Z de Wald	Sig.	Intervalle de confiance à 95 % Borne inférieure	Borne supérieure
Résidu	12,672715	2,833705	4,472	,000	8,175893	19,642832

a. Variable dépendante : Cool N2 Amplitude ΔT0-T2.

Matrice de corrélation pour les estimations des paramètres de covariance[a]

Paramètre	Résidu
Résidu	1

a. Variable dépendante : Cool N2 Amplitude ΔT0-T2.

Matrice de covariance pour les estimations des paramètres de covariance[a]

Paramètre	Résidu
Résidu	8,029885

a. Variable dépendante : Cool N2 Amplitude ΔT0-T2.

Moyenne marginale estimée

1. Grand Mean[a]

Moyenne	Erreur standard	ddl	Intervalle de confiance à 95 % Borne inférieure	Borne supérieure
,829	,549	40	-,281	1,939

a. Variable dépendante : Cool N2 Amplitude ΔT0-T2.

2. Condition

Estimations[a]

Condition	Moyenne	Erreur standard	ddl	Intervalle de confiance à 95 % Borne inférieure	Borne supérieure
Sham	1,152	,777	40	-,418	2,722
taVNS	,505	,777	40	-1,065	2,075

a. Variable dépendante : Cool N2 Amplitude ΔT0-T2.

Comparaisons appariées[a]

(I) Condition	(J) Condition	Différence moyenne (I-J)	Erreur standard	ddl	Sig.[b]
Sham	taVNS	,647	1,099	40	,559
taVNS	Sham	-,647	1,099	40	,559

Comparaisons appariées[a]

(I) Condition	(J) Condition	Intervalle de confiance à 95 % pour la différence[b] Borne inférieure	Borne supérieure
Sham	taVNS	-1,573	2,867
taVNS	Sham	-2,867	1,573

Basées sur les moyennes marginales estimées[a]

a. Variable dépendante : Cool N2 Amplitude ΔT0-T2.

b. Ajustement pour les comparaisons multiples : Bonferroni.

Tests univariés[a]

Ddl du numérateur	Ddl du dénominateur	F	Sig.
1	40	,347	,559

Le test de F permet de tester l'effet de Condition. Il s'appuie sur les comparaisons appariées (indépendantes) linéaires parmi les moyennes marginales estimées.[a]

a. Variable dépendante : Cool N2 Amplitude ΔT0-T2.

1.3.3. Cool N2 Latency (ΔT0-T2).

```
MIXED CoolN2LatencyΔT0T2 BY Condition
  /CRITERIA=CIN(95) MXITER(100) MXSTEP(10) SCORING(1)
SINGULAR(0.000000000001) HCONVERGE(0,
    ABSOLUTE) LCONVERGE(0, ABSOLUTE) PCONVERGE(0.000001, ABSOLUTE)
  /FIXED=Condition | SSTYPE(3)
  /METHOD=REML
  /PRINT=CPS CORB COVB DESCRIPTIVES G  SOLUTION TESTCOV
  /EMMEANS=TABLES(OVERALL)
  /EMMEANS=TABLES(Condition) COMPARE ADJ(BONFERRONI).
```

Remarques

Sortie obtenue		05-MAY-2021 12:50:12
Commentaires		
Entrée	Jeu de données actif	Jeu_de_données4
	Filtre	<sans>
	Pondération	<sans>
	Fichier scindé	<sans>
	N de lignes dans le fichier de travail	44
Gestion des valeurs manquantes	Définition de la valeur manquante	Les valeurs manquantes définies par l'utilisateur sont traitées comme étant manquantes.
	Observations utilisées	Les statistiques sont basées sur toutes les observations comportant des données valides pour toutes les variables du modèle.

Syntaxe		MIXED CoolN2LatencyΔT0T2 BY Condition /CRITERIA=CIN(95) MXITER(100) MXSTEP(10) SCORING(1) SINGULAR(0.000000000001) HCONVERGE(0, ABSOLUTE) LCONVERGE(0, ABSOLUTE) PCONVERGE(0.000001, ABSOLUTE) /FIXED=Condition | SSTYPE(3) /METHOD=REML /PRINT=CPS CORB COVB DESCRIPTIVES G SOLUTION TESTCOV /EMMEANS=TABLES(OVERALL) /EMMEANS=TABLES(Condition) COMPARE ADJ(BONFERRONI).
Ressources	Temps de processeur	00:00:00,02
	Temps écoulé	00:00:00,01

Récapitulatif de traitement des observations

		Effectif	Pourcentage marginal
Condition	Sham	22	51,2%
	taVNS	21	48,8%
Valide		43	100,0%
Exclues		1	
Total		44	

Statistiques descriptives

Cool N2 Latency ΔT0-T2

Condition	Effectif	Moyenne	Ecart type	Coefficient de variation
Sham	22	-,033140045454545	,098726729945642	-297,9%
taVNS	21	,018950000000000	,077812079075167	410,6%
Total	43	-,007700720930233	,091928244128447	-1193,8%

Dimension du modèle[a]

		Nombre de niveaux	Nombre de paramètres
Effets fixes	Constante	1	1
	Condition	2	1
Résidu			1
Total		3	3

a. Variable dépendante : Cool N2 Latency ΔT0-T2.

Critères d'information[a]

Log de vraisemblance restreint -2	-75,751
Critère d'information d'Akaike (AIC)	-73,751
Critère de Hurvich et Tsai (AICC)	-73,648
Critère de Bozdogan (CAIC)	-71,037
Critère bayésien de Schwartz (BIC)	-72,037

Les critères d'informations sont présentés en plus petit, disposant d'un meilleur format.[a]

a. Variable dépendante : Cool N2 Latency ΔT0-T2.

Effets fixes

Tests des effets fixes de type III[a]

Source	Ddl du numérateur	Ddl du dénominateur	F	Sig.
Constante	1	41	,272	,605
Condition	1	41	3,669	,062

a. Variable dépendante : Cool N2 Latency ΔT0-T2.

Estimations des effets fixes[a]

Paramètre	Estimation	Erreur standard	ddl	t	Sig.	Intervalle de confiance à 95 % Borne inférieure
Constante	,018950	,019452	41	,974	,336	-,020334
[Condition=Sham]	-,052090	,027195	41	-1,915	,062	-,107011
[Condition=taVNS]	0[b]	0	.	.	.	.

Estimations des effets fixes[a]

Paramètre	Intervalle de confiance à 95 % Borne supérieure
Constante	,058234
[Condition=Sham]	,002831
[Condition=taVNS]	.

a. Variable dépendante : Cool N2 Latency ΔT0-T2.

b. Ce paramètre est défini sur 0, car il est redondant.

Matrice de corrélation pour les estimations des effets fixes[a]

Paramètre	Constante	[Condition=Sham]	[Condition=taVNS]
Constante	1	-,715	.[b]
[Condition=Sham]	-,715	1	.[b]
[Condition=taVNS]	.[b]	.[b]	.[b]

a. Variable dépendante : Cool N2 Latency ΔT0-T2.

b. La corrélation est manquante par défaut, car elle est associée à un paramètre redondant.

Matrice de covariance pour les estimations des effets fixes[a]

Paramètre	Constante	[Condition=Sham]	[Condition=taVNS]
Constante	,000378	-,000378	0[b]
[Condition=Sham]	-,000378	,000740	0[b]
[Condition=taVNS]	0[b]	0[b]	0[b]

a. Variable dépendante : Cool N2 Latency ΔT0-T2.

b. La covariance est définie sur 0, car elle est associée à un paramètre redondant.

Paramètres de covariance

Estimations des paramètres de covariance[a]

Paramètre	Estimation	Erreur standard	Z de Wald	Sig.	Intervalle de confiance à 95 %	
					Borne inférieure	Borne supérieure
Résidu	,007946	,001755	4,528	,000	,005154	,012250

a. Variable dépendante : Cool N2 Latency ΔT0-T2.

Matrice de corrélation pour les estimations des paramètres de covariance[a]

Paramètre	Résidu
Résidu	1

a. Variable dépendante : Cool N2 Latency ΔT0-T2.

Matrice de covariance pour les estimations des paramètres de covariance[a]

Paramètre	Résidu
Résidu	3,079847E-6

a. Variable dépendante : Cool N2 Latency ΔT0-T2.

Moyenne marginale estimée

1. Grand Mean[a]

Moyenne	Erreur standard	ddl	Intervalle de confiance à 95 % Borne inférieure	Borne supérieure
-,007	,014	41	-,035	,020

a. Variable dépendante : Cool N2 Latency ΔT0-T2.

2. Condition

Estimations[a]

Condition	Moyenne	Erreur standard	ddl	Intervalle de confiance à 95 % Borne inférieure	Borne supérieure
Sham	-,033	,019	41	-,072	,005
taVNS	,019	,019	41	-,020	,058

a. Variable dépendante : Cool N2 Latency ΔT0-T2.

Comparaisons appariées[a]

(I) Condition	(J) Condition	Différence moyenne (I-J)	Erreur standard	ddl	Sig.[b]
Sham	taVNS	-,052	,027	41	,062
taVNS	Sham	,052	,027	41	,062

Comparaisons appariées[a]

(I) Condition	(J) Condition	Intervalle de confiance à 95 % pour la différence[b] Borne inférieure	Borne supérieure
Sham	taVNS	-,107	,003
taVNS	Sham	-,003	,107

Basées sur les moyennes marginales estimées[a]

a. Variable dépendante : Cool N2 Latency ΔT0-T2.

b. Ajustement pour les comparaisons multiples : Bonferroni.

Tests univariés[a]

Ddl du numérateur	Ddl du dénominateur	F	Sig.
1	41	3,669	,062

Le test de F permet de tester l'effet de Condition. Il s'appuie sur les comparaisons appariées (indépendantes) linéaires parmi les moyennes marginales estimées.[a]

a. Variable dépendante : Cool N2 Latency ΔT0-T2.

1.3.4. Cool P2 Amplitude (ΔT0-T2).

```
MIXED CoolP2AmplitudeΔT0T2 BY Condition
  /CRITERIA=CIN(95) MXITER(100) MXSTEP(10) SCORING(1)
SINGULAR(0.000000000001) HCONVERGE(0,
    ABSOLUTE) LCONVERGE(0, ABSOLUTE) PCONVERGE(0.000001, ABSOLUTE)
  /FIXED=Condition | SSTYPE(3)
  /METHOD=REML
  /PRINT=CPS CORB COVB DESCRIPTIVES G  SOLUTION TESTCOV
  /EMMEANS=TABLES(OVERALL)
  /EMMEANS=TABLES(Condition) COMPARE ADJ(BONFERRONI).
```

Remarques

Sortie obtenue		05-MAY-2021 12:50:55
Commentaires		
Entrée	Jeu de données actif	Jeu_de_données4
	Filtre	<sans>
	Pondération	<sans>
	Fichier scindé	<sans>
	N de lignes dans le fichier de travail	44
Gestion des valeurs manquantes	Définition de la valeur manquante	Les valeurs manquantes définies par l'utilisateur sont traitées comme étant manquantes.
	Observations utilisées	Les statistiques sont basées sur toutes les observations comportant des données valides pour toutes les variables du modèle.

Syntaxe		MIXED CoolP2AmplitudeΔT0T2 BY Condition<br>/CRITERIA=CIN(95) MXITER(100) MXSTEP(10) SCORING(1) SINGULAR(0.000000000001) HCONVERGE(0, ABSOLUTE) LCONVERGE(0, ABSOLUTE) PCONVERGE(0.000001, ABSOLUTE)<br>/FIXED=Condition | SSTYPE(3)<br>/METHOD=REML<br>/PRINT=CPS CORB COVB DESCRIPTIVES G SOLUTION TESTCOV<br>/EMMEANS=TABLES(OVERALL)<br>/EMMEANS=TABLES(Condition) COMPARE ADJ(BONFERRONI).
Ressources	Temps de processeur	00:00:00,00
	Temps écoulé	00:00:00,01

Récapitulatif de traitement des observations

		Effectif	Pourcentage marginal
Condition	Sham	21	50,0%
	taVNS	21	50,0%
Valide		42	100,0%
Exclues		2	
Total		44	

Statistiques descriptives

Cool P2 Amplitude ΔT0-T2

Condition	Effectif	Moyenne	Ecart type	Coefficient de variation
Sham	21	1,374417619047619	2,783398319055153	202,5%
taVNS	21	,886094285714286	4,377082754996268	494,0%
Total	42	1,130255952380952	3,631256651225383	321,3%

Dimension du modèle[a]

		Nombre de niveaux	Nombre de paramètres
Effets fixes	Constante	1	1
	Condition	2	1
Résidu			1
Total		3	3

a. Variable dépendante : Cool P2 Amplitude ΔT0-T2.

Critères d'information[a]

Log de vraisemblance restreint -2	223,572
Critère d'information d'Akaike (AIC)	225,572
Critère de Hurvich et Tsai (AICC)	225,678
Critère de Bozdogan (CAIC)	228,261
Critère bayésien de Schwartz (BIC)	227,261

Les critères d'informations sont présentés en plus petit, disposant d'un meilleur format.[a]

a. Variable dépendante : Cool P2 Amplitude ΔT0-T2.

Effets fixes

Tests des effets fixes de type III[a]

Source	Ddl du numérateur	Ddl du dénominateur	F	Sig.
Constante	1	40	3,988	,053
Condition	1	40	,186	,668

a. Variable dépendante : Cool P2 Amplitude ΔT0-T2.

Estimations des effets fixes[a]

Paramètre	Estimation	Erreur standard	ddl	t	Sig.	Intervalle de confiance à 95 % Borne inférieure
Constante	,886094	,800389	40	1,107	,275	-,731553
[Condition=Sham]	,488323	1,131921	40	,431	,668	-1,799375
[Condition=taVNS]	0[b]	0	.	.	.	.

Estimations des effets fixes[a]

Paramètre	Intervalle de confiance à 95 % Borne supérieure
Constante	2,503741
[Condition=Sham]	2,776021
[Condition=taVNS]	.

a. Variable dépendante : Cool P2 Amplitude ΔT0-T2.

b. Ce paramètre est défini sur 0, car il est redondant.

Matrice de corrélation pour les estimations des effets fixes[a]

Paramètre	Constante	[Condition=Sham]	[Condition=taVNS]
Constante	1	-,707	.[b]
[Condition=Sham]	-,707	1	.[b]
[Condition=taVNS]	.[b]	.[b]	.[b]

a. Variable dépendante : Cool P2 Amplitude ΔT0-T2.

b. La corrélation est manquante par défaut, car elle est associée à un paramètre redondant.

Matrice de covariance pour les estimations des effets fixes[a]

Paramètre	Constante	[Condition=Sham]	[Condition=taVNS]
Constante	,640623	-,640623	0[b]
[Condition=Sham]	-,640623	1,281246	0[b]
[Condition=taVNS]	0[b]	0[b]	0[b]

a. Variable dépendante : Cool P2 Amplitude ΔT0-T2.

b. La covariance est définie sur 0, car elle est associée à un paramètre redondant.

Paramètres de covariance

Estimations des paramètres de covariance[a]

Paramètre	Estimation	Erreur standard	Z de Wald	Sig.	Intervalle de confiance à 95 %	
					Borne inférieure	Borne supérieure
Résidu	13,453080	3,008200	4,472	,000	8,679351	20,852406

a. Variable dépendante : Cool P2 Amplitude ΔT0-T2.

Matrice de corrélation pour les estimations des paramètres de covariance[a]

Paramètre	Résidu
Résidu	1

a. Variable dépendante : Cool P2 Amplitude ΔT0-T2.

Matrice de covariance pour les estimations des paramètres de covariance[a]

Paramètre	Résidu
Résidu	9,049268

a. Variable dépendante : Cool P2 Amplitude ΔT0-T2.

Moyenne marginale estimée

1. Grand Mean[a]

Moyenne	Erreur standard	ddl	Intervalle de confiance à 95 % Borne inférieure	Intervalle de confiance à 95 % Borne supérieure
1,130	,566	40	-,014	2,274

a. Variable dépendante : Cool P2 Amplitude ΔT0-T2.

2. Condition

Estimations[a]

Condition	Moyenne	Erreur standard	ddl	Intervalle de confiance à 95 % Borne inférieure	Intervalle de confiance à 95 % Borne supérieure
Sham	1,374	,800	40	-,243	2,992
taVNS	,886	,800	40	-,732	2,504

a. Variable dépendante : Cool P2 Amplitude ΔT0-T2.

Comparaisons appariées[a]

(I) Condition	(J) Condition	Différence moyenne (I-J)	Erreur standard	ddl	Sig.[b]
Sham	taVNS	,488	1,132	40	,668
taVNS	Sham	-,488	1,132	40	,668

Comparaisons appariées[a]

(I) Condition	(J) Condition	Intervalle de confiance à 95 % pour la différence[b] Borne inférieure	Intervalle de confiance à 95 % pour la différence[b] Borne supérieure
Sham	taVNS	-1,799	2,776
taVNS	Sham	-2,776	1,799

Basées sur les moyennes marginales estimées[a]

a. Variable dépendante : Cool P2 Amplitude ΔT0-T2.

b. Ajustement pour les comparaisons multiples : Bonferroni.

Tests univariés[a]

Ddl du numérateur	Ddl du dénominateur	F	Sig.
1	40	,186	,668

Le test de F permet de tester l'effet de Condition. Il s'appuie sur les comparaisons appariées (indépendantes) linéaires parmi les moyennes marginales estimées.[a]

a. Variable dépendante : Cool P2 Amplitude ΔT0-T2.

1.3.5. Cool P2 Latency (ΔT0-T2).

```
MIXED CoolP2latencyΔT0T2 BY Condition
  /CRITERIA=CIN(95) MXITER(100) MXSTEP(10) SCORING(1)
SINGULAR(0.000000000001) HCONVERGE(0,
    ABSOLUTE) LCONVERGE(0, ABSOLUTE) PCONVERGE(0.000001, ABSOLUTE)
  /FIXED=Condition | SSTYPE(3)
  /METHOD=REML
  /PRINT=CPS CORB COVB DESCRIPTIVES G  SOLUTION TESTCOV
  /EMMEANS=TABLES(OVERALL)
  /EMMEANS=TABLES(Condition) COMPARE ADJ(BONFERRONI).
```

Remarques

Sortie obtenue		05-MAY-2021 12:51:21
Commentaires		
Entrée	Jeu de données actif	Jeu_de_données4
	Filtre	<sans>
	Pondération	<sans>
	Fichier scindé	<sans>
	N de lignes dans le fichier de travail	44
Gestion des valeurs manquantes	Définition de la valeur manquante	Les valeurs manquantes définies par l'utilisateur sont traitées comme étant manquantes.
	Observations utilisées	Les statistiques sont basées sur toutes les observations comportant des données valides pour toutes les variables du modèle.

Syntaxe		MIXED CoolP2latencyΔT0T2 BY Condition<br>/CRITERIA=CIN(95) MXITER(100) MXSTEP(10) SCORING(1) SINGULAR(0.000000000001) HCONVERGE(0, ABSOLUTE) LCONVERGE(0, ABSOLUTE) PCONVERGE(0.000001, ABSOLUTE)<br>/FIXED=Condition | SSTYPE(3)<br>/METHOD=REML<br>/PRINT=CPS CORB COVB DESCRIPTIVES G SOLUTION TESTCOV<br>/EMMEANS=TABLES(OVERALL)<br>/EMMEANS=TABLES(Condition) COMPARE ADJ(BONFERRONI).
Ressources	Temps de processeur	00:00:00,00
	Temps écoulé	00:00:00,01

Récapitulatif de traitement des observations

		Effectif	Pourcentage marginal
Condition	Sham	22	51,2%
	taVNS	21	48,8%
Valide		43	100,0%
Exclues		1	
Total		44	

Statistiques descriptives

Cool P2 latency ΔT0-T2

Condition	Effectif	Moyenne	Ecart type	Coefficient de variation
Sham	22	,019770000000000	,099912481607417	505,4%
taVNS	21	,012138095238095	,118802021641850	978,8%
Total	43	,016042790697674	,108291581265484	675,0%

Dimension du modèle[a]

		Nombre de niveaux	Nombre de paramètres
Effets fixes	Constante	1	1
	Condition	2	1
Résidu			1
Total		3	3

a. Variable dépendante : Cool P2 latency ΔT0-T2.

Critères d'information[a]

Log de vraisemblance restreint -2	-58,856
Critère d'information d'Akaike (AIC)	-56,856
Critère de Hurvich et Tsai (AICC)	-56,753
Critère de Bozdogan (CAIC)	-54,142
Critère bayésien de Schwartz (BIC)	-55,142

Les critères d'informations sont présentés en plus petit, disposant d'un meilleur format.[a]

a. Variable dépendante : Cool P2 latency ΔT0-T2.

Effets fixes

Tests des effets fixes de type III[a]

Source	Ddl du numérateur	Ddl du dénominateur	F	Sig.
Constante	1	41	,912	,345
Condition	1	41	,052	,820

a. Variable dépendante : Cool P2 latency ΔT0-T2.

Estimations des effets fixes[a]

Paramètre	Estimation	Erreur standard	ddl	t	Sig.	Intervalle de confiance à 95 % Borne inférieure
Constante	,012138	,023902	41	,508	,614	-,036134
[Condition=Sham]	,007632	,033417	41	,228	,820	-,059855
[Condition=taVNS]	0[b]	0	.	.	.	.

Estimations des effets fixes[a]

Paramètre	Intervalle de confiance à 95 % Borne supérieure
Constante	,060410
[Condition=Sham]	,075118
[Condition=taVNS]	.

a. Variable dépendante : Cool P2 latency ΔT0-T2.

b. Ce paramètre est défini sur 0, car il est redondant.

Matrice de corrélation pour les estimations des effets fixes[a]

Paramètre	Constante	[Condition=Sham]	[Condition=taVNS]
Constante	1	-,715	.[b]
[Condition=Sham]	-,715	1	.[b]
[Condition=taVNS]	.[b]	.[b]	.[b]

a. Variable dépendante : Cool P2 latency ΔT0-T2.

b. La corrélation est manquante par défaut, car elle est associée à un paramètre redondant.

Matrice de covariance pour les estimations des effets fixes[a]

Paramètre	Constante	[Condition=Sham]	[Condition=taVNS]
Constante	,000571	-,000571	0[b]

[Condition=Sham]	-,000571	,001117	0[b]
[Condition=taVNS]	0[b]	0[b]	0[b]

a. Variable dépendante : Cool P2 latency ΔT0-T2.

b. La covariance est définie sur 0, car elle est associée à un paramètre redondant.

Paramètres de covariance

Estimations des paramètres de covariance[a]

Paramètre	Estimation	Erreur standard	Z de Wald	Sig.	Intervalle de confiance à 95 %	
					Borne inférieure	Borne supérieure
Résidu	,011998	,002650	4,528	,000	,007782	,018497

a. Variable dépendante : Cool P2 latency ΔT0-T2.

Matrice de corrélation pour les estimations des paramètres de covariance[a]

Paramètre	Résidu
Résidu	1

a. Variable dépendante : Cool P2 latency ΔT0-T2.

Matrice de covariance pour les estimations des paramètres de covariance[a]

Paramètre	Résidu
Résidu	7,021849E-6

a. Variable dépendante : Cool P2 latency ΔT0-T2.

Moyenne marginale estimée

1. Grand Mean[a]

Moyenne	Erreur standard	ddl	Intervalle de confiance à 95 %	
			Borne inférieure	Borne supérieure
,016	,017	41	-,018	,050

a. Variable dépendante : Cool P2 latency ΔT0-T2.

2. Condition

Estimations[a]

Condition	Moyenne	Erreur standard	ddl	Intervalle de confiance à 95 % Borne inférieure	Intervalle de confiance à 95 % Borne supérieure
Sham	,020	,023	41	-,027	,067
taVNS	,012	,024	41	-,036	,060

a. Variable dépendante : Cool P2 latency ΔT0-T2.

Comparaisons appariées[a]

(I) Condition	(J) Condition	Différence moyenne (I-J)	Erreur standard	ddl	Sig.[b]
Sham	taVNS	,008	,033	41	,820
taVNS	Sham	-,008	,033	41	,820

Comparaisons appariées[a]

(I) Condition	(J) Condition	Intervalle de confiance à 95 % pour la différence[b] Borne inférieure	Intervalle de confiance à 95 % pour la différence[b] Borne supérieure
Sham	taVNS	-,060	,075
taVNS	Sham	-,075	,060

Basées sur les moyennes marginales estimées[a]

a. Variable dépendante : Cool P2 latency ΔT0-T2.

b. Ajustement pour les comparaisons multiples : Bonferroni.

Tests univariés[a]

Ddl du numérateur	Ddl du dénominateur	F	Sig.
1	41	,052	,820

Le test de F permet de tester l'effet de Condition. Il s'appuie sur les comparaisons appariées (indépendantes) linéaires parmi les moyennes marginales estimées.[a]

a. Variable dépendante : Cool P2 latency ΔT0-T2.

2. Experiment 2

2.1. Laser-evoked potentials (LEPs)

2.1.1. LEPs N2P2 Amplitude (ΔOFF-ON)..

```
MIXED LaserN2P2AmplitudeΔOFFON BY Condition
  /CRITERIA=CIN(95) MXITER(100) MXSTEP(10) SCORING(1)
SINGULAR(0.000000000001) HCONVERGE(0,
    ABSOLUTE) LCONVERGE(0, ABSOLUTE) PCONVERGE(0.000001, ABSOLUTE)
  /FIXED=Condition | SSTYPE(3)
  /METHOD=REML
  /PRINT=CPS CORB COVB DESCRIPTIVES G  SOLUTION TESTCOV
  /EMMEANS=TABLES(OVERALL)
  /EMMEANS=TABLES(Condition) COMPARE ADJ(BONFERRONI).
```

Remarques

Sortie obtenue		05-MAY-2021 13:09:06
Commentaires		
Entrée	Jeu de données actif	Jeu_de_données5
	Filtre	<sans>
	Pondération	<sans>
	Fichier scindé	<sans>
	N de lignes dans le fichier de travail	30
Gestion des valeurs manquantes	Définition de la valeur manquante	Les valeurs manquantes définies par l'utilisateur sont traitées comme étant manquantes.
	Observations utilisées	Les statistiques sont basées sur toutes les observations comportant des données valides pour toutes les variables du modèle.

Syntaxe		MIXED LaserN2P2AmplitudeΔOFFON BY Condition<br>/CRITERIA=CIN(95) MXITER(100) MXSTEP(10) SCORING(1) SINGULAR(0.000000000001) HCONVERGE(0, ABSOLUTE) LCONVERGE(0, ABSOLUTE) PCONVERGE(0.000001, ABSOLUTE)<br>/FIXED=Condition | SSTYPE(3)<br>/METHOD=REML<br>/PRINT=CPS CORB COVB DESCRIPTIVES G SOLUTION TESTCOV<br>/EMMEANS=TABLES(OVERALL)<br>/EMMEANS=TABLES(Condition) COMPARE ADJ(BONFERRONI).
Ressources	Temps de processeur	00:00:00,02
	Temps écoulé	00:00:00,01

Récapitulatif de traitement des observations

		Effectif	Pourcentage marginal
Condition	Sham	15	50,0%
	taVNS	15	50,0%
Valide		30	100,0%
Exclues		0	
Total		30	

Statistiques descriptives

Laser N2P2 Amplitude ΔOFF-ON

Condition	Effectif	Moyenne	Ecart type	Coefficient de variation
Sham	15	,028460000000000	4,324213143914162	15194,0%
taVNS	15	2,223239999999999	3,860363607559711	173,6%
Total	30	1,125850000000000	4,179362743516833	371,2%

Dimension du modèle[a]

		Nombre de niveaux	Nombre de paramètres
Effets fixes	Constante	1	1
	Condition	2	1
Résidu			1
Total		3	3

a. Variable dépendante : Laser N2P2 Amplitude ΔOFF-ON.

Critères d'information[a]

Log de vraisemblance restreint -2	163,876
Critère d'information d'Akaike (AIC)	165,876
Critère de Hurvich et Tsai (AICC)	166,030
Critère de Bozdogan (CAIC)	168,208
Critère bayésien de Schwartz (BIC)	167,208

Les critères d'informations sont présentés en plus petit, disposant d'un meilleur format.[a]

a. Variable dépendante : Laser N2P2 Amplitude ΔOFF-ON.

Effets fixes

Tests des effets fixes de type III[a]

Source	Ddl du numérateur	Ddl du dénominateur	F	Sig.
Constante	1	28	2,263	,144
Condition	1	28	2,150	,154

a. Variable dépendante : Laser N2P2 Amplitude ΔOFF-ON.

Estimations des effets fixes[a]

Paramètre	Estimation	Erreur standard	ddl	t	Sig.	Intervalle de confiance à 95 % Borne inférieure
Constante	2,223240	1,058320	28	2,101	,045	,055370
[Condition=Sham]	-2,194780	1,496690	28	-1,466	,154	-5,260611
[Condition=taVNS]	0[b]	0	.	.	.	.

Estimations des effets fixes[a]

Paramètre	Intervalle de confiance à 95 % Borne supérieure
Constante	4,391110
[Condition=Sham]	,871051
[Condition=taVNS]	.

a. Variable dépendante : Laser N2P2 Amplitude ΔOFF-ON.

b. Ce paramètre est défini sur 0, car il est redondant.

Matrice de corrélation pour les estimations des effets fixes[a]

Paramètre	Constante	[Condition=Sham]	[Condition=taVNS]
Constante	1	-,707	.[b]
[Condition=Sham]	-,707	1	.[b]
[Condition=taVNS]	.[b]	.[b]	.[b]

a. Variable dépendante : Laser N2P2 Amplitude ΔOFF-ON.

b. La corrélation est manquante par défaut, car elle est associée à un paramètre redondant.

Matrice de covariance pour les estimations des effets fixes[a]

Paramètre	Constante	[Condition=Sham]	[Condition=taVNS]
Constante	1,120041	-1,120041	0[b]

[Condition=Sham]	-1,120041	2,240082	0[b]
[Condition=taVNS]	0[b]	0[b]	0[b]

a. Variable dépendante : Laser N2P2 Amplitude ΔOFF-ON.

b. La covariance est définie sur 0, car elle est associée à un paramètre redondant.

Paramètres de covariance

Estimations des paramètres de covariance[a]

					Intervalle de confiance à 95 %	
Paramètre	Estimation	Erreur standard	Z de Wald	Sig.	Borne inférieure	Borne supérieure
Résidu	16,800613	4,490153	3,742	,000	9,950203	28,367321

a. Variable dépendante : Laser N2P2 Amplitude ΔOFF-ON.

Matrice de corrélation pour les estimations des paramètres de covariance[a]

Paramètre	Résidu
Résidu	1

a. Variable dépendante : Laser N2P2 Amplitude ΔOFF-ON.

Matrice de covariance pour les estimations des paramètres de covariance[a]

Paramètre	Résidu
Résidu	20,161472

a. Variable dépendante : Laser N2P2 Amplitude ΔOFF-ON.

Moyenne marginale estimée

1. Grand Mean[a]

			Intervalle de confiance à 95 %	
Moyenne	Erreur standard	ddl	Borne inférieure	Borne supérieure
1,126	,748	28	-,407	2,659

a. Variable dépendante : Laser N2P2 Amplitude ΔOFF-ON.

2. Condition

Estimations[a]

Condition	Moyenne	Erreur standard	ddl	Intervalle de confiance à 95 %	
				Borne inférieure	Borne supérieure
Sham	,028	1,058	28	-2,139	2,196
taVNS	2,223	1,058	28	,055	4,391

a. Variable dépendante : Laser N2P2 Amplitude ΔOFF-ON.

Comparaisons appariées[a]

(I) Condition	(J) Condition	Différence moyenne (I-J)	Erreur standard	ddl	Sig.[b]
Sham	taVNS	-2,195	1,497	28	,154
taVNS	Sham	2,195	1,497	28	,154

Comparaisons appariées[a]

(I) Condition	(J) Condition	Intervalle de confiance à 95 % pour la différence[b]	
		Borne inférieure	Borne supérieure
Sham	taVNS	-5,261	,871
taVNS	Sham	-,871	5,261

Basées sur les moyennes marginales estimées[a]

a. Variable dépendante : Laser N2P2 Amplitude ΔOFF-ON.

b. Ajustement pour les comparaisons multiples : Bonferroni.

Tests univariés[a]

Ddl du numérateur	Ddl du dénominateur	F	Sig.
1	28	2,150	,154

Le test de F permet de tester l'effet de Condition. Il s'appuie sur les comparaisons appariées (indépendantes) linéaires parmi les moyennes marginales estimées.[a]

a. Variable dépendante : Laser N2P2 Amplitude ΔOFF-ON.

2.1.2. LEPs N2 Amplitude (ΔOFF-ON).

```
MIXED LaserN2AmplitudeΔOFFON BY Condition
  /CRITERIA=CIN(95) MXITER(100) MXSTEP(10) SCORING(1)
SINGULAR(0.000000000001) HCONVERGE(0,
    ABSOLUTE) LCONVERGE(0, ABSOLUTE) PCONVERGE(0.000001, ABSOLUTE)
  /FIXED=Condition | SSTYPE(3)
  /METHOD=REML
  /PRINT=CPS CORB COVB DESCRIPTIVES G  SOLUTION TESTCOV
  /EMMEANS=TABLES(OVERALL)
  /EMMEANS=TABLES(Condition) COMPARE ADJ(BONFERRONI).
```

Remarques

Sortie obtenue		05-MAY-2021 13:10:12
Commentaires		
Entrée	Jeu de données actif	Jeu_de_données5
	Filtre	<sans>
	Pondération	<sans>
	Fichier scindé	<sans>
	N de lignes dans le fichier de travail	30
Gestion des valeurs manquantes	Définition de la valeur manquante	Les valeurs manquantes définies par l'utilisateur sont traitées comme étant manquantes.
	Observations utilisées	Les statistiques sont basées sur toutes les observations comportant des données valides pour toutes les variables du modèle.

Syntaxe		MIXED LaserN2AmplitudeΔOFFON BY Condition<br>/CRITERIA=CIN(95) MXITER(100) MXSTEP(10) SCORING(1) SINGULAR(0.000000000001) HCONVERGE(0,<br>ABSOLUTE) LCONVERGE(0, ABSOLUTE) PCONVERGE(0.000001, ABSOLUTE)<br>/FIXED=Condition | SSTYPE(3)<br>/METHOD=REML<br>/PRINT=CPS CORB COVB DESCRIPTIVES G SOLUTION TESTCOV<br>/EMMEANS=TABLES(OVERALL)<br>/EMMEANS=TABLES(Condition) COMPARE ADJ(BONFERRONI).
Ressources	Temps de processeur	00:00:00,02
	Temps écoulé	00:00:00,01

Récapitulatif de traitement des observations

		Effectif	Pourcentage marginal
Condition	Sham	15	50,0%
	taVNS	15	50,0%
Valide		30	100,0%
Exclues		0	
Total		30	

Statistiques descriptives

Laser N2 Amplitude ΔOFF-ON

Condition	Effectif	Moyenne	Ecart type	Coefficient de variation
Sham	15	,647980000000000	3,483491943274326	537,6%
taVNS	15	-1,239573333333333	3,918513718867446	-316,1%
Total	30	-,295796666666666	3,767254347617596	-1273,6%

Dimension du modèle[a]

		Nombre de niveaux	Nombre de paramètres
Effets fixes	Constante	1	1
	Condition	2	1
Résidu			1
Total		3	3

a. Variable dépendante : Laser N2 Amplitude ΔOFF-ON.

Critères d'information[a]

Log de vraisemblance restreint -2	158,255
Critère d'information d'Akaike (AIC)	160,255
Critère de Hurvich et Tsai (AICC)	160,409
Critère de Bozdogan (CAIC)	162,587
Critère bayésien de Schwartz (BIC)	161,587

Les critères d'informations sont présentés en plus petit, disposant d'un meilleur format.[a]

a. Variable dépendante : Laser N2 Amplitude ΔOFF-ON.

Effets fixes

Tests des effets fixes de type III[a]

Source	Ddl du numérateur	Ddl du dénominateur	F	Sig.
Constante	1	28	,191	,665
Condition	1	28	1,944	,174

a. Variable dépendante : Laser N2 Amplitude ΔOFF-ON.

Estimations des effets fixes[a]

Paramètre	Estimation	Erreur standard	ddl	t	Sig.	Intervalle de confiance à 95 % Borne inférieure
Constante	-1,239573	,957244	28	-1,295	,206	-3,200398
[Condition=Sham]	1,887553	1,353747	28	1,394	,174	-,885472
[Condition=taVNS]	0[b]	0	.	.	.	.

Estimations des effets fixes[a]

Paramètre	Intervalle de confiance à 95 % Borne supérieure
Constante	,721252
[Condition=Sham]	4,660578
[Condition=taVNS]	.

a. Variable dépendante : Laser N2 Amplitude ΔOFF-ON.

b. Ce paramètre est défini sur 0, car il est redondant.

Matrice de corrélation pour les estimations des effets fixes[a]

Paramètre	Constante	[Condition=Sham]	[Condition=taVNS]
Constante	1	-,707	.[b]
[Condition=Sham]	-,707	1	.[b]
[Condition=taVNS]	.[b]	.[b]	.[b]

a. Variable dépendante : Laser N2 Amplitude ΔOFF-ON.

b. La corrélation est manquante par défaut, car elle est associée à un paramètre redondant.

Matrice de covariance pour les estimations des effets fixes[a]

Paramètre	Constante	[Condition=Sham]	[Condition=taVNS]
Constante	,916316	-,916316	0[b]

[Condition=Sham]	-,916316	1,832631	0[b]
[Condition=taVNS]	0[b]	0[b]	0[b]

a. Variable dépendante : Laser N2 Amplitude ΔOFF-ON.

b. La covariance est définie sur 0, car elle est associée à un paramètre redondant.

Paramètres de covariance

Estimations des paramètres de covariance[a]

Paramètre	Estimation	Erreur standard	Z de Wald	Sig.	Intervalle de confiance à 95 % Borne inférieure	Borne supérieure
Résidu	13,744733	3,673434	3,742	,000	8,140351	23,207560

a. Variable dépendante : Laser N2 Amplitude ΔOFF-ON.

Matrice de corrélation pour les estimations des paramètres de covariance[a]

Paramètre	Résidu
Résidu	1

a. Variable dépendante : Laser N2 Amplitude ΔOFF-ON.

Matrice de covariance pour les estimations des paramètres de covariance[a]

Paramètre	Résidu
Résidu	13,494120

a. Variable dépendante : Laser N2 Amplitude ΔOFF-ON.

Moyenne marginale estimée

1. Grand Mean[a]

Moyenne	Erreur standard	ddl	Intervalle de confiance à 95 % Borne inférieure	Borne supérieure
-,296	,677	28	-1,682	1,091

a. Variable dépendante : Laser N2 Amplitude ΔOFF-ON.

2. Condition

Estimations[a]

Condition	Moyenne	Erreur standard	ddl	Intervalle de confiance à 95 % Borne inférieure	Intervalle de confiance à 95 % Borne supérieure
Sham	,648	,957	28	-1,313	2,609
taVNS	-1,240	,957	28	-3,200	,721

a. Variable dépendante : Laser N2 Amplitude ΔOFF-ON.

Comparaisons appariées[a]

(I) Condition	(J) Condition	Différence moyenne (I-J)	Erreur standard	ddl	Sig.[b]
Sham	taVNS	1,888	1,354	28	,174
taVNS	Sham	-1,888	1,354	28	,174

Comparaisons appariées[a]

(I) Condition	(J) Condition	Intervalle de confiance à 95 % pour la différence[b] Borne inférieure	Intervalle de confiance à 95 % pour la différence[b] Borne supérieure
Sham	taVNS	-,885	4,661
taVNS	Sham	-4,661	,885

Basées sur les moyennes marginales estimées[a]

a. Variable dépendante : Laser N2 Amplitude ΔOFF-ON.

b. Ajustement pour les comparaisons multiples : Bonferroni.

Tests univariés[a]

Ddl du numérateur	Ddl du dénominateur	F	Sig.
1	28	1,944	,174

Le test de F permet de tester l'effet de Condition. Il s'appuie sur les comparaisons appariées (indépendantes) linéaires parmi les moyennes marginales estimées.[a]

a. Variable dépendante : Laser N2 Amplitude ΔOFF-ON.

2.1.3. LEPs N2 Latency (ΔOFF-ON).

```
MIXED LaserN2LatencyΔOFFON BY Condition
  /CRITERIA=CIN(95) MXITER(100) MXSTEP(10) SCORING(1)
SINGULAR(0.000000000001) HCONVERGE(0,
    ABSOLUTE) LCONVERGE(0, ABSOLUTE) PCONVERGE(0.000001, ABSOLUTE)
  /FIXED=Condition | SSTYPE(3)
  /METHOD=REML
  /PRINT=CPS CORB COVB DESCRIPTIVES G  SOLUTION TESTCOV
  /EMMEANS=TABLES(OVERALL)
  /EMMEANS=TABLES(Condition) COMPARE ADJ(BONFERRONI).
```

Remarques

Sortie obtenue		05-MAY-2021 13:11:01
Commentaires		
Entrée	Jeu de données actif	Jeu_de_données5
	Filtre	<sans>
	Pondération	<sans>
	Fichier scindé	<sans>
	N de lignes dans le fichier de travail	30
Gestion des valeurs manquantes	Définition de la valeur manquante	Les valeurs manquantes définies par l'utilisateur sont traitées comme étant manquantes.
	Observations utilisées	Les statistiques sont basées sur toutes les observations comportant des données valides pour toutes les variables du modèle.

Syntaxe		MIXED LaserN2LatencyΔOFFON BY Condition<br>/CRITERIA=CIN(95) MXITER(100) MXSTEP(10) SCORING(1) SINGULAR(0.000000000001) HCONVERGE(0,<br>ABSOLUTE) LCONVERGE(0, ABSOLUTE) PCONVERGE(0.000001, ABSOLUTE)<br>/FIXED=Condition | SSTYPE(3)<br>/METHOD=REML<br>/PRINT=CPS CORB COVB DESCRIPTIVES G SOLUTION TESTCOV<br><br>/EMMEANS=TABLES(OVERALL)<br><br>/EMMEANS=TABLES(Condition) COMPARE ADJ(BONFERRONI).
Ressources	Temps de processeur	00:00:00,02
	Temps écoulé	00:00:00,01

Récapitulatif de traitement des observations

		Effectif	Pourcentage marginal
Condition	Sham	15	50,0%
	taVNS	15	50,0%
Valide		30	100,0%
Exclues		0	
Total		30	

Statistiques descriptives

Laser N2 Latency ΔOFF-ON

Condition	Effectif	Moyenne	Ecart type	Coefficient de variation
Sham	15	,005133333333333	,015127395520647	294,7%
taVNS	15	,009533333333333	,018019301820416	189,0%
Total	30	,007333333333333	,016499390445028	225,0%

Dimension du modèle[a]

		Nombre de niveaux	Nombre de paramètres
Effets fixes	Constante	1	1
	Condition	2	1
Résidu			1
Total		3	3

a. Variable dépendante : Laser N2 Latency ΔOFF-ON.

Critères d'information[a]

Log de vraisemblance restreint -2	-144,509
Critère d'information d'Akaike (AIC)	-142,509
Critère de Hurvich et Tsai (AICC)	-142,355
Critère de Bozdogan (CAIC)	-140,177
Critère bayésien de Schwartz (BIC)	-141,177

Les critères d'informations sont présentés en plus petit, disposant d'un meilleur format.[a]

a. Variable dépendante : Laser N2 Latency ΔOFF-ON.

Effets fixes

Tests des effets fixes de type III[a]

Source	Ddl du numérateur	Ddl du dénominateur	F	Sig.
Constante	1	28	5,829	,023
Condition	1	28	,525	,475

a. Variable dépendante : Laser N2 Latency ΔOFF-ON.

Estimations des effets fixes[a]

Paramètre	Estimation	Erreur standard	ddl	t	Sig.	Intervalle de confiance à 95 % Borne inférieure
Constante	,009533	,004295	28	2,219	,035	,000734
[Condition=Sham]	-,004400	,006075	28	-,724	,475	-,016843
[Condition=taVNS]	0[b]	0	.	.	.	.

Estimations des effets fixes[a]

Paramètre	Intervalle de confiance à 95 % Borne supérieure
Constante	,018332
[Condition=Sham]	,008043
[Condition=taVNS]	.

a. Variable dépendante : Laser N2 Latency ΔOFF-ON.

b. Ce paramètre est défini sur 0, car il est redondant.

Matrice de corrélation pour les estimations des effets fixes[a]

Paramètre	Constante	[Condition=Sham]	[Condition=taVNS]
Constante	1	-,707	.[b]
[Condition=Sham]	-,707	1	.[b]
[Condition=taVNS]	.[b]	.[b]	.[b]

a. Variable dépendante : Laser N2 Latency ΔOFF-ON.

b. La corrélation est manquante par défaut, car elle est associée à un paramètre redondant.

Matrice de covariance pour les estimations des effets fixes[a]

Paramètre	Constante	[Condition=Sham]	[Condition=taVNS]
Constante	1,845111E-5	-1,845111E-5	0[b]

[Condition=Sham]	-1,845111E-5	3,690222E-5	0[b]
[Condition=taVNS]	0[b]	0[b]	0[b]

a. Variable dépendante : Laser N2 Latency ΔOFF-ON.

b. La covariance est définie sur 0, car elle est associée à un paramètre redondant.

Paramètres de covariance

Estimations des paramètres de covariance[a]

Paramètre	Estimation	Erreur standard	Z de Wald	Sig.	Intervalle de confiance à 95 % Borne inférieure	Borne supérieure
Résidu	,000277	7,396900E-5	3,742	,000	,000164	,000467

a. Variable dépendante : Laser N2 Latency ΔOFF-ON.

Matrice de corrélation pour les estimations des paramètres de covariance[a]

Paramètre	Résidu
Résidu	1

a. Variable dépendante : Laser N2 Latency ΔOFF-ON.

Matrice de covariance pour les estimations des paramètres de covariance[a]

Paramètre	Résidu
Résidu	5,471413E-9

a. Variable dépendante : Laser N2 Latency ΔOFF-ON.

Moyenne marginale estimée

1. Grand Mean[a]

Moyenne	Erreur standard	ddl	Intervalle de confiance à 95 % Borne inférieure	Borne supérieure
,007	,003	28	,001	,014

a. Variable dépendante : Laser N2 Latency ΔOFF-ON.

2. Condition

Estimations[a]

Condition	Moyenne	Erreur standard	ddl	Intervalle de confiance à 95 % Borne inférieure	Borne supérieure
Sham	,005	,004	28	-,004	,014
taVNS	,010	,004	28	,001	,018

a. Variable dépendante : Laser N2 Latency ΔOFF-ON.

Comparaisons appariées[a]

(I) Condition	(J) Condition	Différence moyenne (I-J)	Erreur standard	ddl	Sig.[b]
Sham	taVNS	-,004	,006	28	,475
taVNS	Sham	,004	,006	28	,475

Comparaisons appariées[a]

(I) Condition	(J) Condition	Intervalle de confiance à 95 % pour la différence[b] Borne inférieure	Borne supérieure
Sham	taVNS	-,017	,008
taVNS	Sham	-,008	,017

Basées sur les moyennes marginales estimées[a]

a. Variable dépendante : Laser N2 Latency ΔOFF-ON.

b. Ajustement pour les comparaisons multiples : Bonferroni.

Tests univariés[a]

Ddl du numérateur	Ddl du dénominateur	F	Sig.
1	28	,525	,475

Le test de F permet de tester l'effet de Condition. Il s'appuie sur les comparaisons appariées (indépendantes) linéaires parmi les moyennes marginales estimées.[a]

a. Variable dépendante : Laser N2 Latency ΔOFF-ON.

2.1.4. LEPs P2 Amplitude (ΔOFF-ON).

```
MIXED LaserP2AmplitudeΔOFFON BY Condition
  /CRITERIA=CIN(95) MXITER(100) MXSTEP(10) SCORING(1)
SINGULAR(0.000000000001) HCONVERGE(0,
    ABSOLUTE) LCONVERGE(0, ABSOLUTE) PCONVERGE(0.000001, ABSOLUTE)
  /FIXED=Condition | SSTYPE(3)
  /METHOD=REML
  /PRINT=CPS CORB COVB DESCRIPTIVES G  SOLUTION TESTCOV
  /EMMEANS=TABLES(OVERALL)
  /EMMEANS=TABLES(Condition) COMPARE ADJ(BONFERRONI).
```

Remarques

Sortie obtenue		05-MAY-2021 13:11:47
Commentaires		
Entrée	Jeu de données actif	Jeu_de_données5
	Filtre	<sans>
	Pondération	<sans>
	Fichier scindé	<sans>
	N de lignes dans le fichier de travail	30
Gestion des valeurs manquantes	Définition de la valeur manquante	Les valeurs manquantes définies par l'utilisateur sont traitées comme étant manquantes.
	Observations utilisées	Les statistiques sont basées sur toutes les observations comportant des données valides pour toutes les variables du modèle.

Syntaxe		MIXED LaserP2AmplitudeΔOFFON BY Condition<br>/CRITERIA=CIN(95) MXITER(100) MXSTEP(10) SCORING(1) SINGULAR(0.000000000001) HCONVERGE(0,<br>ABSOLUTE) LCONVERGE(0, ABSOLUTE) PCONVERGE(0.000001, ABSOLUTE)<br>/FIXED=Condition | SSTYPE(3)<br>/METHOD=REML<br>/PRINT=CPS CORB COVB DESCRIPTIVES G SOLUTION TESTCOV<br>/EMMEANS=TABLES(OVERALL)<br>/EMMEANS=TABLES(Condition) COMPARE ADJ(BONFERRONI).
Ressources	Temps de processeur	00:00:00,00
	Temps écoulé	00:00:00,01

Récapitulatif de traitement des observations

		Effectif	Pourcentage marginal
Condition	Sham	15	50,0%
	taVNS	15	50,0%
Valide		30	100,0%
Exclues		0	
Total		30	

Statistiques descriptives

Laser P2 Amplitude ΔOFF-ON

Condition	Effectif	Moyenne	Ecart type	Coefficient de variation
Sham	15	,676440000000000	2,028702606452846	299,9%
taVNS	15	,983666666666667	3,442340253304982	349,9%
Total	30	,830053333333333	2,780614427726031	335,0%

Dimension du modèle[a]

		Nombre de niveaux	Nombre de paramètres
Effets fixes	Constante	1	1
	Condition	2	1
Résidu			1
Total		3	3

a. Variable dépendante : Laser P2 Amplitude ΔOFF-ON.

Critères d'information[a]

Log de vraisemblance restreint -2	143,040
Critère d'information d'Akaike (AIC)	145,040
Critère de Hurvich et Tsai (AICC)	145,194
Critère de Bozdogan (CAIC)	147,373
Critère bayésien de Schwartz (BIC)	146,373

Les critères d'informations sont présentés en plus petit, disposant d'un meilleur format.[a]

a. Variable dépendante : Laser P2 Amplitude ΔOFF-ON.

Effets fixes

Tests des effets fixes de type III[a]

Source	Ddl du numérateur	Ddl du dénominateur	F	Sig.
Constante	1	28	2,589	,119
Condition	1	28	,089	,768

a. Variable dépendante : Laser P2 Amplitude ΔOFF-ON.

Estimations des effets fixes[a]

Paramètre	Estimation	Erreur standard	ddl	t	Sig.	Intervalle de confiance à 95 % Borne inférieure
Constante	,983667	,729505	28	1,348	,188	-,510657
[Condition=Sham]	-,307227	1,031676	28	-,298	,768	-2,420520
[Condition=taVNS]	0[b]	0	.	.	.	.

Estimations des effets fixes[a]

Paramètre	Intervalle de confiance à 95 % Borne supérieure
Constante	2,477991
[Condition=Sham]	1,806066
[Condition=taVNS]	.

a. Variable dépendante : Laser P2 Amplitude ΔOFF-ON.

b. Ce paramètre est défini sur 0, car il est redondant.

Matrice de corrélation pour les estimations des effets fixes[a]

Paramètre	Constante	[Condition=Sham]	[Condition=taVNS]
Constante	1	-,707	.[b]
[Condition=Sham]	-,707	1	.[b]
[Condition=taVNS]	.[b]	.[b]	.[b]

a. Variable dépendante : Laser P2 Amplitude ΔOFF-ON.

b. La corrélation est manquante par défaut, car elle est associée à un paramètre redondant.

Matrice de covariance pour les estimations des effets fixes[a]

Paramètre	Constante	[Condition=Sham]	[Condition=taVNS]
Constante	,532178	-,532178	0[b]
[Condition=Sham]	-,532178	1,064356	0[b]
[Condition=taVNS]	0[b]	0[b]	0[b]

a. Variable dépendante : Laser P2 Amplitude ΔOFF-ON.

b. La covariance est définie sur 0, car elle est associée à un paramètre redondant.

Paramètres de covariance

Estimations des paramètres de covariance[a]

Paramètre	Estimation	Erreur standard	Z de Wald	Sig.	Intervalle de confiance à 95 % Borne inférieure	Borne supérieure
Résidu	7,982670	2,133458	3,742	,000	4,727756	13,478494

a. Variable dépendante : Laser P2 Amplitude ΔOFF-ON.

Matrice de corrélation pour les estimations des paramètres de covariance[a]

Paramètre	Résidu
Résidu	1

a. Variable dépendante : Laser P2 Amplitude ΔOFF-ON.

Matrice de covariance pour les estimations des paramètres de covariance[a]

Paramètre	Résidu
Résidu	4,551645

a. Variable dépendante : Laser P2 Amplitude ΔOFF-ON.

Moyenne marginale estimée

1. Grand Mean[a]

Moyenne	Erreur standard	ddl	Intervalle de confiance à 95 % Borne inférieure	Borne supérieure
,830	,516	28	-,227	1,887

a. Variable dépendante : Laser P2 Amplitude ΔOFF-ON.

2. Condition

Estimations[a]

Condition	Moyenne	Erreur standard	ddl	Intervalle de confiance à 95 %	
				Borne inférieure	Borne supérieure
Sham	,676	,730	28	-,818	2,171
taVNS	,984	,730	28	-,511	2,478

a. Variable dépendante : Laser P2 Amplitude ΔOFF-ON.

Comparaisons appariées[a]

(I) Condition	(J) Condition	Différence moyenne (I-J)	Erreur standard	ddl	Sig.[b]
Sham	taVNS	-,307	1,032	28	,768
taVNS	Sham	,307	1,032	28	,768

Comparaisons appariées[a]

(I) Condition	(J) Condition	Intervalle de confiance à 95 % pour la différence[b]	
		Borne inférieure	Borne supérieure
Sham	taVNS	-2,421	1,806
taVNS	Sham	-1,806	2,421

Basées sur les moyennes marginales estimées[a]

a. Variable dépendante : Laser P2 Amplitude ΔOFF-ON.

b. Ajustement pour les comparaisons multiples : Bonferroni.

Tests univariés[a]

Ddl du numérateur	Ddl du dénominateur	F	Sig.
1	28	,089	,768

Le test de F permet de tester l'effet de Condition. Il s'appuie sur les comparaisons appariées (indépendantes) linéaires parmi les moyennes marginales estimées.[a]

a. Variable dépendante : Laser P2 Amplitude ΔOFF-ON.

2.1.5. LEPs P2 Latency (ΔOFF-ON).

Remarques

Sortie obtenue		05-MAY-2021 13:12:23
Commentaires		
Entrée	Jeu de données actif	Jeu_de_données5
	Filtre	<sans>
	Pondération	<sans>
	Fichier scindé	<sans>
	N de lignes dans le fichier de travail	30
Gestion des valeurs manquantes	Définition de la valeur manquante	Les valeurs manquantes définies par l'utilisateur sont traitées comme étant manquantes.
	Observations utilisées	Les statistiques sont basées sur toutes les observations comportant des données valides pour toutes les variables du modèle.

Syntaxe		MIXED LaserP2LatencyΔOFFON BY Condition<br>/CRITERIA=CIN(95) MXITER(100) MXSTEP(10) SCORING(1) SINGULAR(0.000000000001) HCONVERGE(0,<br>ABSOLUTE) LCONVERGE(0, ABSOLUTE) PCONVERGE(0.000001, ABSOLUTE)<br>/FIXED=Condition | SSTYPE(3)<br>/METHOD=REML<br>/PRINT=CPS CORB COVB DESCRIPTIVES G SOLUTION TESTCOV<br><br>/EMMEANS=TABLES(OVERALL)<br><br>/EMMEANS=TABLES(Condition) COMPARE ADJ(BONFERRONI).
Ressources	Temps de processeur	00:00:00,02
	Temps écoulé	00:00:00,01

Récapitulatif de traitement des observations

		Effectif	Pourcentage marginal
Condition	Sham	15	50,0%
	taVNS	15	50,0%
Valide		30	100,0%
Exclues		0	
Total		30	

Statistiques descriptives

Laser P2 Latency ΔOFF-ON

Condition	Effectif	Moyenne	Ecart type	Coefficient de variation
Sham	15	,012132666666667	,043469394893200	358,3%
taVNS	15	,006600000000000	,037140274635495	562,7%
Total	30	,009366333333333	,039825227707497	425,2%

Dimension du modèle[a]

		Nombre de niveaux	Nombre de paramètres
Effets fixes	Constante	1	1
	Condition	2	1
Résidu			1
Total		3	3

a. Variable dépendante : Laser P2 Latency ΔOFF-ON.

Critères d'information[a]

Log de vraisemblance restreint -2	-94,783
Critère d'information d'Akaike (AIC)	-92,783
Critère de Hurvich et Tsai (AICC)	-92,629
Critère de Bozdogan (CAIC)	-90,451
Critère bayésien de Schwartz (BIC)	-91,451

Les critères d'informations sont présentés en plus petit, disposant d'un meilleur format.[a]

a. Variable dépendante : Laser P2 Latency ΔOFF-ON.

Effets fixes

Tests des effets fixes de type III[a]

Source	Ddl du numérateur	Ddl du dénominateur	F	Sig.
Constante	1	28	1,610	,215
Condition	1	28	,140	,711

a. Variable dépendante : Laser P2 Latency ΔOFF-ON.

Estimations des effets fixes[a]

Paramètre	Estimation	Erreur standard	ddl	t	Sig.	Intervalle de confiance à 95 % Borne inférieure
Constante	,006600	,010439	28	,632	,532	-,014783
[Condition=Sham]	,005533	,014763	28	,375	,711	-,024707
[Condition=taVNS]	0[b]	0	.	.	.	.

Estimations des effets fixes[a]

Paramètre	Intervalle de confiance à 95 % Borne supérieure
Constante	,027983
[Condition=Sham]	,035772
[Condition=taVNS]	.

a. Variable dépendante : Laser P2 Latency ΔOFF-ON.

b. Ce paramètre est défini sur 0, car il est redondant.

Matrice de corrélation pour les estimations des effets fixes[a]

Paramètre	Constante	[Condition=Sham]	[Condition=taVNS]
Constante	1	-,707	.[b]
[Condition=Sham]	-,707	1	.[b]
[Condition=taVNS]	.[b]	.[b]	.[b]

a. Variable dépendante : Laser P2 Latency ΔOFF-ON.

b. La corrélation est manquante par défaut, car elle est associée à un paramètre redondant.

Matrice de covariance pour les estimations des effets fixes[a]

Paramètre	Constante	[Condition=Sham]	[Condition=taVNS]
Constante	,000109	-,000109	0[b]

[Condition=Sham]	-,000109	,000218	0[b]
[Condition=taVNS]	0[b]	0[b]	0[b]

a. Variable dépendante : Laser P2 Latency ΔOFF-ON.

b. La covariance est définie sur 0, car elle est associée à un paramètre redondant.

Paramètres de covariance

Estimations des paramètres de covariance[a]

Paramètre	Estimation	Erreur standard	Z de Wald	Sig.	Intervalle de confiance à 95 % Borne inférieure	Intervalle de confiance à 95 % Borne supérieure
Résidu	,001634	,000437	3,742	,000	,000968	,002760

a. Variable dépendante : Laser P2 Latency ΔOFF-ON.

Matrice de corrélation pour les estimations des paramètres de covariance[a]

Paramètre	Résidu
Résidu	1

a. Variable dépendante : Laser P2 Latency ΔOFF-ON.

Matrice de covariance pour les estimations des paramètres de covariance[a]

Paramètre	Résidu
Résidu	1,908265E-7

a. Variable dépendante : Laser P2 Latency ΔOFF-ON.

Moyenne marginale estimée

1. Grand Mean[a]

Moyenne	Erreur standard	ddl	Intervalle de confiance à 95 % Borne inférieure	Intervalle de confiance à 95 % Borne supérieure

,009	,007	28	-,006	,024

a. Variable dépendante : Laser P2 Latency ΔOFF-ON.

2. Condition

Estimations[a]

Condition	Moyenne	Erreur standard	ddl	Intervalle de confiance à 95 %	
				Borne inférieure	Borne supérieure
Sham	,012	,010	28	-,009	,034
taVNS	,007	,010	28	-,015	,028

a. Variable dépendante : Laser P2 Latency ΔOFF-ON.

Comparaisons appariées[a]

(I) Condition	(J) Condition	Différence moyenne (I-J)	Erreur standard	ddl	Sig.[b]
Sham	taVNS	,006	,015	28	,711
taVNS	Sham	-,006	,015	28	,711

Comparaisons appariées[a]

(I) Condition	(J) Condition	Intervalle de confiance à 95 % pour la différence[b]	
		Borne inférieure	Borne supérieure
Sham	taVNS	-,025	,036
taVNS	Sham	-,036	,025

Basées sur les moyennes marginales estimées[a]

a. Variable dépendante : Laser P2 Latency ΔOFF-ON.

b. Ajustement pour les comparaisons multiples : Bonferroni.

Tests univariés[a]

Ddl du numérateur	Ddl du dénominateur	F	Sig.
1	28	,140	,711

Le test de F permet de tester l'effet de Condition. Il s'appuie sur les comparaisons appariées (indépendantes) linéaires parmi les moyennes marginales estimées.[a]

a. Variable dépendante : Laser P2 Latency ΔOFF-ON.

2.2. Vibrotactile-evoked potentials

2.2.1. Vibrotactile N2P2 Amplitude (ΔOFF-ON).

```
MIXED VibrotactileN2P2AmplitudeΔOFFON BY Condition
  /CRITERIA=CIN(95) MXITER(100) MXSTEP(10) SCORING(1)
SINGULAR(0.000000000001) HCONVERGE(0,
    ABSOLUTE) LCONVERGE(0, ABSOLUTE) PCONVERGE(0.000001, ABSOLUTE)
  /FIXED=Condition | SSTYPE(3)
  /METHOD=REML
  /PRINT=CPS CORB COVB DESCRIPTIVES G  SOLUTION TESTCOV
  /EMMEANS=TABLES(OVERALL)
  /EMMEANS=TABLES(Condition) COMPARE ADJ(BONFERRONI).
```

Remarques

Sortie obtenue		05-MAY-2021 13:13:18
Commentaires		
Entrée	Jeu de données actif	Jeu_de_données5
	Filtre	<sans>
	Pondération	<sans>
	Fichier scindé	<sans>
	N de lignes dans le fichier de travail	30
Gestion des valeurs manquantes	Définition de la valeur manquante	Les valeurs manquantes définies par l'utilisateur sont traitées comme étant manquantes.
	Observations utilisées	Les statistiques sont basées sur toutes les observations comportant des données valides pour toutes les variables du modèle.

Syntaxe		MIXED VibrotactileN2P2AmplitudeΔOFFON BY Condition<br>/CRITERIA=CIN(95) MXITER(100) MXSTEP(10) SCORING(1) SINGULAR(0.000000000001) HCONVERGE(0,<br>ABSOLUTE) LCONVERGE(0, ABSOLUTE) PCONVERGE(0.000001, ABSOLUTE)<br>/FIXED=Condition | SSTYPE(3)<br>/METHOD=REML<br>/PRINT=CPS CORB COVB DESCRIPTIVES G SOLUTION TESTCOV<br><br>/EMMEANS=TABLES(OVERALL)<br><br>/EMMEANS=TABLES(Condition) COMPARE ADJ(BONFERRONI).
Ressources	Temps de processeur	00:00:00,00
	Temps écoulé	00:00:00,01

Récapitulatif de traitement des observations

		Effectif	Pourcentage marginal
Condition	Sham	15	50,0%
	taVNS	15	50,0%
Valide		30	100,0%
Exclues		0	
Total		30	

Statistiques descriptives

Vibrotactile N2P2 Amplitude ΔOFF-ON

Condition	Effectif	Moyenne	Ecart type	Coefficient de variation
Sham	15	2,191446666666666	3,032783501496996	138,4%
taVNS	15	,816873333333334	2,396225997112563	293,3%
Total	30	1,504160000000000	2,775051015449054	184,5%

Dimension du modèle[a]

		Nombre de niveaux	Nombre de paramètres
Effets fixes	Constante	1	1
	Condition	2	1
Résidu			1
Total		3	3

a. Variable dépendante : Vibrotactile N2P2 Amplitude ΔOFF-ON.

Critères d'information[a]

Log de vraisemblance restreint -2	141,181
Critère d'information d'Akaike (AIC)	143,181
Critère de Hurvich et Tsai (AICC)	143,335
Critère de Bozdogan (CAIC)	145,513
Critère bayésien de Schwartz (BIC)	144,513

Les critères d'informations sont présentés en plus petit, disposant d'un meilleur format.[a]

a. Variable dépendante : Vibrotactile N2P2 Amplitude ΔOFF-ON.

Effets fixes

Tests des effets fixes de type III[a]

Source	Ddl du numérateur	Ddl du dénominateur	F	Sig.
Constante	1	28	9,087	,005
Condition	1	28	1,897	,179

a. Variable dépendante : Vibrotactile N2P2 Amplitude ΔOFF-ON.

Estimations des effets fixes[a]

Paramètre	Estimation	Erreur standard	ddl	t	Sig.	Intervalle de confiance à 95 % Borne inférieure
Constante	,816873	,705683	28	1,158	,257	-,628654
[Condition=Sham]	1,374573	,997987	28	1,377	,179	-,669711
[Condition=taVNS]	0[b]	0	.	.	.	.

Estimations des effets fixes[a]

Paramètre	Intervalle de confiance à 95 % Borne supérieure
Constante	2,262400
[Condition=Sham]	3,418857
[Condition=taVNS]	.

a. Variable dépendante : Vibrotactile N2P2 Amplitude ΔOFF-ON.

b. Ce paramètre est défini sur 0, car il est redondant.

Matrice de corrélation pour les estimations des effets fixes[a]

Paramètre	Constante	[Condition=Sham]	[Condition=taVNS]
Constante	1	-,707	.[b]
[Condition=Sham]	-,707	1	.[b]
[Condition=taVNS]	.[b]	.[b]	.[b]

a. Variable dépendante : Vibrotactile N2P2 Amplitude ΔOFF-ON.

b. La corrélation est manquante par défaut, car elle est associée à un paramètre redondant.

Matrice de covariance pour les estimations des effets fixes[a]

Paramètre	Constante	[Condition=Sham]	[Condition=taVNS]
Constante	,497989	-,497989	0[b]

[Condition=Sham]	-,497989	,995978	0[b]
[Condition=taVNS]	0[b]	0[b]	0[b]

a. Variable dépendante : Vibrotactile N2P2 Amplitude ΔOFF-ON.

b. La covariance est définie sur 0, car elle est associée à un paramètre redondant.

Paramètres de covariance

Estimations des paramètres de covariance[a]

Paramètre	Estimation	Erreur standard	Z de Wald	Sig.	Intervalle de confiance à 95 % Borne inférieure	Intervalle de confiance à 95 % Borne supérieure
Résidu	7,469837	1,996398	3,742	,000	4,424029	12,612591

a. Variable dépendante : Vibrotactile N2P2 Amplitude ΔOFF-ON.

Matrice de corrélation pour les estimations des paramètres de covariance[a]

Paramètre	Résidu
Résidu	1

a. Variable dépendante : Vibrotactile N2P2 Amplitude ΔOFF-ON.

Matrice de covariance pour les estimations des paramètres de covariance[a]

Paramètre	Résidu
Résidu	3,985605

a. Variable dépendante : Vibrotactile N2P2 Amplitude ΔOFF-ON.

Moyenne marginale estimée

1. Grand Mean[a]

Moyenne	Erreur standard	ddl	Intervalle de confiance à 95 % Borne inférieure	Borne supérieure
1,504	,499	28	,482	2,526

a. Variable dépendante : Vibrotactile N2P2 Amplitude ΔOFF-ON.

2. Condition

Estimations[a]

Condition	Moyenne	Erreur standard	ddl	Intervalle de confiance à 95 % Borne inférieure	Borne supérieure
Sham	2,191	,706	28	,746	3,637
taVNS	,817	,706	28	-,629	2,262

a. Variable dépendante : Vibrotactile N2P2 Amplitude ΔOFF-ON.

Comparaisons appariées[a]

(I) Condition	(J) Condition	Différence moyenne (I-J)	Erreur standard	ddl	Sig.[b]
Sham	taVNS	1,375	,998	28	,179
taVNS	Sham	-1,375	,998	28	,179

Comparaisons appariées[a]

(I) Condition	(J) Condition	Intervalle de confiance à 95 % pour la différence[b] Borne inférieure	Borne supérieure
Sham	taVNS	-,670	3,419
taVNS	Sham	-3,419	,670

Basées sur les moyennes marginales estimées[a]

a. Variable dépendante : Vibrotactile N2P2 Amplitude ΔOFF-ON.

b. Ajustement pour les comparaisons multiples : Bonferroni.

Tests univariés[a]

Ddl du numérateur	Ddl du dénominateur	F	Sig.
1	28	1,897	,179

Le test de F permet de tester l'effet de Condition. Il s'appuie sur les comparaisons appariées (indépendantes) linéaires parmi les moyennes marginales estimées.[a]

a. Variable dépendante : Vibrotactile N2P2 Amplitude ΔOFF-ON.

2.2.2 Vibrotactile N2 Amplitude (ΔOFF-ON).

```
MIXED VibrotactileN2AmplitudeΔOFFON BY Condition
  /CRITERIA=CIN(95) MXITER(100) MXSTEP(10) SCORING(1)
SINGULAR(0.000000000001) HCONVERGE(0,
    ABSOLUTE) LCONVERGE(0, ABSOLUTE) PCONVERGE(0.000001, ABSOLUTE)
  /FIXED=Condition | SSTYPE(3)
  /METHOD=REML
  /PRINT=CPS CORB COVB DESCRIPTIVES G  SOLUTION TESTCOV
  /EMMEANS=TABLES(OVERALL)
  /EMMEANS=TABLES(Condition) COMPARE ADJ(BONFERRONI).
```

Remarques

Sortie obtenue		05-MAY-2021 13:14:54
Commentaires		
Entrée	Jeu de données actif	Jeu_de_données5
	Filtre	<sans>
	Pondération	<sans>
	Fichier scindé	<sans>
	N de lignes dans le fichier de travail	30
Gestion des valeurs manquantes	Définition de la valeur manquante	Les valeurs manquantes définies par l'utilisateur sont traitées comme étant manquantes.
	Observations utilisées	Les statistiques sont basées sur toutes les observations comportant des données valides pour toutes les variables du modèle.

Syntaxe		MIXED VibrotactileN2AmplitudeΔOFFON BY Condition<br>/CRITERIA=CIN(95) MXITER(100) MXSTEP(10) SCORING(1) SINGULAR(0.000000000001) HCONVERGE(0, ABSOLUTE) LCONVERGE(0, ABSOLUTE) PCONVERGE(0.000001, ABSOLUTE)<br>/FIXED=Condition | SSTYPE(3)<br>/METHOD=REML<br>/PRINT=CPS CORB COVB DESCRIPTIVES G SOLUTION TESTCOV<br>/EMMEANS=TABLES(OVERALL)<br>/EMMEANS=TABLES(Condition) COMPARE ADJ(BONFERRONI).
Ressources	Temps de processeur	00:00:00,00
	Temps écoulé	00:00:00,01

Récapitulatif de traitement des observations

		Effectif	Pourcentage marginal
Condition	Sham	15	50,0%
	taVNS	15	50,0%
Valide		30	100,0%
Exclues		0	
Total		30	

Statistiques descriptives

Vibrotactile N2 Amplitude ΔOFF-ON

Condition	Effectif	Moyenne	Ecart type	Coefficient de variation
Sham	15	-1,674413333333333	2,040876900069697	-121,9%
taVNS	15	,059880000000000	2,117971157297204	3537,0%
Total	30	-,807266666666667	2,225804969200927	-275,7%

Dimension du modèle[a]

		Nombre de niveaux	Nombre de paramètres
Effets fixes	Constante	1	1
	Condition	2	1
Résidu			1
Total		3	3

a. Variable dépendante : Vibrotactile N2 Amplitude ΔOFF-ON.

Critères d'information[a]

Log de vraisemblance restreint -2	125,883
Critère d'information d'Akaike (AIC)	127,883
Critère de Hurvich et Tsai (AICC)	128,037
Critère de Bozdogan (CAIC)	130,216
Critère bayésien de Schwartz (BIC)	129,216

Les critères d'informations sont présentés en plus petit, disposant d'un meilleur format.[a]

a. Variable dépendante : Vibrotactile N2 Amplitude ΔOFF-ON.

Effets fixes

Tests des effets fixes de type III[a]

Source	Ddl du numérateur	Ddl du dénominateur	F	Sig.
Constante	1	28	4,520	,042
Condition	1	28	5,215	,030

a. Variable dépendante : Vibrotactile N2 Amplitude ΔOFF-ON.

Estimations des effets fixes[a]

Paramètre	Estimation	Erreur standard	ddl	t	Sig.	Intervalle de confiance à 95 % Borne inférieure
Constante	,059880	,536997	28	,112	,912	-1,040109
[Condition=Sham]	-1,734293	,759429	28	-2,284	,030	-3,289913
[Condition=taVNS]	0[b]	0	.	.	.	.

Estimations des effets fixes[a]

Paramètre	Intervalle de confiance à 95 % Borne supérieure
Constante	1,159869
[Condition=Sham]	-,178674
[Condition=taVNS]	.

a. Variable dépendante : Vibrotactile N2 Amplitude ΔOFF-ON.

b. Ce paramètre est défini sur 0, car il est redondant.

Matrice de corrélation pour les estimations des effets fixes[a]

Paramètre	Constante	[Condition=Sham]	[Condition=taVNS]
Constante	1	-,707	.[b]
[Condition=Sham]	-,707	1	.[b]
[Condition=taVNS]	.[b]	.[b]	.[b]

a. Variable dépendante : Vibrotactile N2 Amplitude ΔOFF-ON.

b. La corrélation est manquante par défaut, car elle est associée à un paramètre redondant.

Matrice de covariance pour les estimations des effets fixes[a]

Paramètre	Constante	[Condition=Sham]	[Condition=taVNS]
Constante	,288366	-,288366	0[b]

[Condition=Sham]	-,288366	,576732	0[b]
[Condition=taVNS]	0[b]	0[b]	0[b]

a. Variable dépendante : Vibrotactile N2 Amplitude ΔOFF-ON.

b. La covariance est définie sur 0, car elle est associée à un paramètre redondant.

Paramètres de covariance

Estimations des paramètres de covariance[a]

Paramètre	Estimation	Erreur standard	Z de Wald	Sig.	Intervalle de confiance à 95 % Borne inférieure	Borne supérieure
Résidu	4,325490	1,156036	3,742	,000	2,561782	7,303458

a. Variable dépendante : Vibrotactile N2 Amplitude ΔOFF-ON.

Matrice de corrélation pour les estimations des paramètres de covariance[a]

Paramètre	Résidu
Résidu	1

a. Variable dépendante : Vibrotactile N2 Amplitude ΔOFF-ON.

Matrice de covariance pour les estimations des paramètres de covariance[a]

Paramètre	Résidu
Résidu	1,336419

a. Variable dépendante : Vibrotactile N2 Amplitude ΔOFF-ON.

Moyenne marginale estimée

1. Grand Mean[a]

Moyenne	Erreur standard	ddl	Intervalle de confiance à 95 % Borne inférieure	Borne supérieure
-,807	,380	28	-1,585	-,029

a. Variable dépendante : Vibrotactile N2 Amplitude ΔOFF-ON.

2. Condition

Estimations[a]

Condition	Moyenne	Erreur standard	ddl	Intervalle de confiance à 95 % Borne inférieure	Borne supérieure
Sham	-1,674	,537	28	-2,774	-,574
taVNS	,060	,537	28	-1,040	1,160

a. Variable dépendante : Vibrotactile N2 Amplitude ΔOFF-ON.

Comparaisons appariées[a]

(I) Condition	(J) Condition	Différence moyenne (I-J)	Erreur standard	ddl	Sig.[c]
Sham	taVNS	-1,734*	,759	28	,030
taVNS	Sham	1,734*	,759	28	,030

Comparaisons appariées[a]

(I) Condition	(J) Condition	Intervalle de confiance à 95 % pour la différence[c] Borne inférieure	Borne supérieure
Sham	taVNS	-3,290	-,179
taVNS	Sham	,179	3,290

Basées sur les moyennes marginales estimées[a]

*. La différence moyenne est significative au niveau ,05.

a. Variable dépendante : Vibrotactile N2 Amplitude ΔOFF-ON.

c. Ajustement pour les comparaisons multiples : Bonferroni.

Tests univariés[a]

Ddl du numérateur	Ddl du dénominateur	F	Sig.
1	28	5,215	,030

Le test de F permet de tester l'effet de Condition. Il s'appuie sur les comparaisons appariées (indépendantes) linéaires parmi les moyennes marginales estimées.[a]

a. Variable dépendante : Vibrotactile N2 Amplitude ΔOFF-ON.

2.2.3. Vibrotactile N2 Latency (ΔOFF-ON).

```
MIXED VibrotactileN2LatencyΔOFFON BY Condition
  /CRITERIA=CIN(95) MXITER(100) MXSTEP(10) SCORING(1)
SINGULAR(0.000000000001) HCONVERGE(0,
    ABSOLUTE) LCONVERGE(0, ABSOLUTE) PCONVERGE(0.000001, ABSOLUTE)
  /FIXED=Condition | SSTYPE(3)
  /METHOD=REML
  /PRINT=CPS CORB COVB DESCRIPTIVES G  SOLUTION TESTCOV
  /EMMEANS=TABLES(OVERALL)
  /EMMEANS=TABLES(Condition) COMPARE ADJ(BONFERRONI).
```

Remarques

Sortie obtenue		05-MAY-2021 13:15:38
Commentaires		
Entrée	Jeu de données actif	Jeu_de_données5
	Filtre	<sans>
	Pondération	<sans>
	Fichier scindé	<sans>
	N de lignes dans le fichier de travail	30
Gestion des valeurs manquantes	Définition de la valeur manquante	Les valeurs manquantes définies par l'utilisateur sont traitées comme étant manquantes.
	Observations utilisées	Les statistiques sont basées sur toutes les observations comportant des données valides pour toutes les variables du modèle.

Syntaxe		MIXED VibrotactileN2LatencyΔOFFON BY Condition<br>/CRITERIA=CIN(95) MXITER(100) MXSTEP(10) SCORING(1) SINGULAR(0.000000000001) HCONVERGE(0,<br>ABSOLUTE) LCONVERGE(0, ABSOLUTE) PCONVERGE(0.000001, ABSOLUTE)<br>/FIXED=Condition | SSTYPE(3)<br>/METHOD=REML<br>/PRINT=CPS CORB COVB DESCRIPTIVES G SOLUTION TESTCOV<br><br>/EMMEANS=TABLES(OVERALL)<br><br>/EMMEANS=TABLES(Condition) COMPARE ADJ(BONFERRONI).
Ressources	Temps de processeur	00:00:00,02
	Temps écoulé	00:00:00,01

Récapitulatif de traitement des observations

		Effectif	Pourcentage marginal
Condition	Sham	15	50,0%
	taVNS	15	50,0%
Valide		30	100,0%
Exclues		0	
Total		30	

Statistiques descriptives

Vibrotactile N2 Latency ΔOFF-ON

Condition	Effectif	Moyenne	Ecart type	Coefficient de variation
Sham	15	-,001066000000000	,009838607479865	-922,9%
taVNS	15	,005000000000000	,034450793065721	689,0%
Total	30	,001967000000000	,025084098156897	1275,2%

Dimension du modèle[a]

		Nombre de niveaux	Nombre de paramètres
Effets fixes	Constante	1	1
	Condition	2	1
Résidu			1
Total		3	3

a. Variable dépendante : Vibrotactile N2 Latency ΔOFF-ON.

Critères d'information[a]

Log de vraisemblance restreint -2	-120,957
Critère d'information d'Akaike (AIC)	-118,957
Critère de Hurvich et Tsai (AICC)	-118,803
Critère de Bozdogan (CAIC)	-116,624
Critère bayésien de Schwartz (BIC)	-117,624

Les critères d'informations sont présentés en plus petit, disposant d'un meilleur format.[a]

a. Variable dépendante : Vibrotactile N2 Latency ΔOFF-ON.

Effets fixes

Tests des effets fixes de type III[a]

Source	Ddl du numérateur	Ddl du dénominateur	F	Sig.
Constante	1	28	,181	,674
Condition	1	28	,430	,517

a. Variable dépendante : Vibrotactile N2 Latency ΔOFF-ON.

Estimations des effets fixes[a]

Paramètre	Estimation	Erreur standard	ddl	t	Sig.	Intervalle de confiance à 95 % Borne inférieure	Intervalle de confiance à 95 % Borne supérieure
Constante	,005000	,006541	28	,764	,451	-,008399	,018399
[Condition=Sham]	-,006066	,009251	28	-,656	,517	-,025015	,012883
[Condition=taVNS]	0[b]	0	.	.	.	.	.

a. Variable dépendante : Vibrotactile N2 Latency ΔOFF-ON.

b. Ce paramètre est défini sur 0, car il est redondant.

Matrice de corrélation pour les estimations des effets fixes[a]

Paramètre	Constante	[Condition=Sham]	[Condition=taVNS]
Constante	1	-,707	.[b]
[Condition=Sham]	-,707	1	.[b]
[Condition=taVNS]	.[b]	.[b]	.[b]

a. Variable dépendante : Vibrotactile N2 Latency ΔOFF-ON.

b. La corrélation est manquante par défaut, car elle est associée à un paramètre redondant.

Matrice de covariance pour les estimations des effets fixes[a]

Paramètre	Constante	[Condition=Sham]	[Condition=taVNS]
Constante	4,278851E-5	-4,278851E-5	0[b]
[Condition=Sham]	-4,278851E-5	8,557702E-5	0[b]
[Condition=taVNS]	0[b]	0[b]	0[b]

a. Variable dépendante : Vibrotactile N2 Latency ΔOFF-ON.

b. La covariance est définie sur 0, car elle est associée à un paramètre redondant.

Paramètres de covariance

Estimations des paramètres de covariance[a]

Paramètre	Estimation	Erreur standard	Z de Wald	Sig.	Intervalle de confiance à 95 % Borne inférieure	Borne supérieure
Résidu	,000642	,000172	3,742	,000	,000380	,001084

a. Variable dépendante : Vibrotactile N2 Latency ΔOFF-ON.

Matrice de corrélation pour les estimations des paramètres de covariance[a]

Paramètre	Résidu
Résidu	1

a. Variable dépendante : Vibrotactile N2 Latency ΔOFF-ON.

Matrice de covariance pour les estimations des paramètres de covariance[a]

Paramètre	Résidu
Résidu	2,942448E-8

a. Variable dépendante : Vibrotactile N2 Latency ΔOFF-ON.

Moyenne marginale estimée

1. Grand Mean[a]

Moyenne	Erreur standard	ddl	Intervalle de confiance à 95 % Borne inférieure	Borne supérieure
,002	,005	28	-,008	,011

a. Variable dépendante : Vibrotactile N2 Latency ΔOFF-ON.

2. Condition

Estimations[a]

Condition	Moyenne	Erreur standard	ddl	Intervalle de confiance à 95 %	
				Borne inférieure	Borne supérieure
Sham	-,001	,007	28	-,014	,012
taVNS	,005	,007	28	-,008	,018

a. Variable dépendante : Vibrotactile N2 Latency ΔOFF-ON.

Comparaisons appariées[a]

(I) Condition	(J) Condition	Différence moyenne (I-J)	Erreur standard	ddl	Sig.[b]
Sham	taVNS	-,006	,009	28	,517
taVNS	Sham	,006	,009	28	,517

Comparaisons appariées[a]

(I) Condition	(J) Condition	Intervalle de confiance à 95 % pour la différence[b]	
		Borne inférieure	Borne supérieure
Sham	taVNS	-,025	,013
taVNS	Sham	-,013	,025

Basées sur les moyennes marginales estimées[a]

a. Variable dépendante : Vibrotactile N2 Latency ΔOFF-ON.

b. Ajustement pour les comparaisons multiples : Bonferroni.

Tests univariés[a]

Ddl du numérateur	Ddl du dénominateur	F	Sig.
1	28	,430	,517

Le test de F permet de tester l'effet de Condition. Il s'appuie sur les comparaisons appariées (indépendantes) linéaires parmi les moyennes marginales estimées.[a]

a. Variable dépendante : Vibrotactile N2 Latency ΔOFF-ON.

2.2.4. P2 Amplitude (ΔOFF-ON).

```
MIXED VibrotactileP2AmplitudeΔOFFON BY Condition
  /CRITERIA=CIN(95) MXITER(100) MXSTEP(10) SCORING(1)
SINGULAR(0.000000000001) HCONVERGE(0,
    ABSOLUTE) LCONVERGE(0, ABSOLUTE) PCONVERGE(0.000001, ABSOLUTE)
  /FIXED=Condition | SSTYPE(3)
  /METHOD=REML
  /PRINT=CPS CORB COVB DESCRIPTIVES G  SOLUTION TESTCOV
  /EMMEANS=TABLES(OVERALL)
  /EMMEANS=TABLES(Condition) COMPARE ADJ(BONFERRONI).
```

Remarques

Sortie obtenue		05-MAY-2021 13:16:26
Commentaires		
Entrée	Jeu de données actif	Jeu_de_données5
	Filtre	<sans>
	Pondération	<sans>
	Fichier scindé	<sans>
	N de lignes dans le fichier de travail	30
Gestion des valeurs manquantes	Définition de la valeur manquante	Les valeurs manquantes définies par l'utilisateur sont traitées comme étant manquantes.
	Observations utilisées	Les statistiques sont basées sur toutes les observations comportant des données valides pour toutes les variables du modèle.

Syntaxe		MIXED VibrotactileP2AmplitudeΔOFFON BY Condition<br>/CRITERIA=CIN(95) MXITER(100) MXSTEP(10) SCORING(1) SINGULAR(0.000000000001) HCONVERGE(0, ABSOLUTE) LCONVERGE(0, ABSOLUTE) PCONVERGE(0.000001, ABSOLUTE)<br>/FIXED=Condition | SSTYPE(3)<br>/METHOD=REML<br>/PRINT=CPS CORB COVB DESCRIPTIVES G SOLUTION TESTCOV<br>/EMMEANS=TABLES(OVERALL)<br>/EMMEANS=TABLES(Condition) COMPARE ADJ(BONFERRONI).
Ressources	Temps de processeur	00:00:00,00
	Temps écoulé	00:00:00,01

Récapitulatif de traitement des observations

		Effectif	Pourcentage marginal
Condition	Sham	15	50,0%
	taVNS	15	50,0%
Valide		30	100,0%
Exclues		0	
Total		30	

Statistiques descriptives

Vibrotactile P2 Amplitude ΔOFF-ON

Condition	Effectif	Moyenne	Ecart type	Coefficient de variation
Sham	15	,517033333333333	2,558456732280689	494,8%
taVNS	15	,876753333333333	1,848331307989787	210,8%
Total	30	,696893333333333	2,200618261391061	315,8%

Dimension du modèle[a]

		Nombre de niveaux	Nombre de paramètres
Effets fixes	Constante	1	1
	Condition	2	1
Résidu			1
Total		3	3

a. Variable dépendante : Vibrotactile P2 Amplitude ΔOFF-ON.

Critères d'information[a]

Log de vraisemblance restreint -2	129,834
Critère d'information d'Akaike (AIC)	131,834
Critère de Hurvich et Tsai (AICC)	131,988
Critère de Bozdogan (CAIC)	134,167
Critère bayésien de Schwartz (BIC)	133,167

Les critères d'informations sont présentés en plus petit, disposant d'un meilleur format.[a]

a. Variable dépendante : Vibrotactile P2 Amplitude ΔOFF-ON.

Effets fixes

Tests des effets fixes de type III[a]

Source	Ddl du numérateur	Ddl du dénominateur	F	Sig.
Constante	1	28	2,925	,098

Condition	1	28	,195	,662

a. Variable dépendante : Vibrotactile P2 Amplitude ΔOFF-ON.

Estimations des effets fixes[a]

Paramètre	Estimation	Erreur standard	ddl	t	Sig.	Intervalle de confiance à 95 % Borne inférieure
Constante	,876753	,576253	28	1,521	,139	-,303648
[Condition=Sham]	-,359720	,814945	28	-,441	,662	-2,029059
[Condition=taVNS]	0[b]	0	.	.	.	.

Estimations des effets fixes[a]

Paramètre	Intervalle de confiance à 95 % Borne supérieure
Constante	2,057154
[Condition=Sham]	1,309619
[Condition=taVNS]	.

a. Variable dépendante : Vibrotactile P2 Amplitude ΔOFF-ON.

b. Ce paramètre est défini sur 0, car il est redondant.

Matrice de corrélation pour les estimations des effets fixes[a]

Paramètre	Constante	[Condition=Sham]	[Condition=taVNS]
Constante	1	-,707	.[b]
[Condition=Sham]	-,707	1	.[b]
[Condition=taVNS]	.[b]	.[b]	.[b]

a. Variable dépendante : Vibrotactile P2 Amplitude ΔOFF-ON.

b. La corrélation est manquante par défaut, car elle est associée à un paramètre redondant.

Matrice de covariance pour les estimations des effets fixes[a]

Paramètre	Constante	[Condition=Sham]	[Condition=taVNS]
Constante	,332068	-,332068	0[b]
[Condition=Sham]	-,332068	,664135	0[b]
[Condition=taVNS]	0[b]	0[b]	0[b]

a. Variable dépendante : Vibrotactile P2 Amplitude ΔOFF-ON.

b. La covariance est définie sur 0, car elle est associée à un paramètre redondant.

Paramètres de covariance

Estimations des paramètres de covariance[a]

Paramètre	Estimation	Erreur standard	Z de Wald	Sig.	Intervalle de confiance à 95 % Borne inférieure	Intervalle de confiance à 95 % Borne supérieure
Résidu	4,981015	1,331232	3,742	,000	2,950018	8,410291

a. Variable dépendante : Vibrotactile P2 Amplitude ΔOFF-ON.

Matrice de corrélation pour les estimations des paramètres de covariance[a]

Paramètre	Résidu
Résidu	1

a. Variable dépendante : Vibrotactile P2 Amplitude ΔOFF-ON.

Matrice de covariance pour les estimations des paramètres de covariance[a]

Paramètre	Résidu
Résidu	1,772179

a. Variable dépendante : Vibrotactile P2 Amplitude ΔOFF-ON.

Moyenne marginale estimée

1. Grand Mean[a]

Moyenne	Erreur standard	ddl	Intervalle de confiance à 95 % Borne inférieure	Intervalle de confiance à 95 % Borne supérieure
,697	,407	28	-,138	1,532

a. Variable dépendante : Vibrotactile P2 Amplitude ΔOFF-ON.

2. Condition

Estimations[a]

Condition	Moyenne	Erreur standard	ddl	Intervalle de confiance à 95 % Borne inférieure	Borne supérieure
Sham	,517	,576	28	-,663	1,697
taVNS	,877	,576	28	-,304	2,057

a. Variable dépendante : Vibrotactile P2 Amplitude ΔOFF-ON.

Comparaisons appariées[a]

(I) Condition	(J) Condition	Différence moyenne (I-J)	Erreur standard	ddl	Sig.[b]
Sham	taVNS	-,360	,815	28	,662
taVNS	Sham	,360	,815	28	,662

Comparaisons appariées[a]

(I) Condition	(J) Condition	Intervalle de confiance à 95 % pour la différence[b] Borne inférieure	Borne supérieure
Sham	taVNS	-2,029	1,310
taVNS	Sham	-1,310	2,029

Basées sur les moyennes marginales estimées[a]

a. Variable dépendante : Vibrotactile P2 Amplitude ΔOFF-ON.

b. Ajustement pour les comparaisons multiples : Bonferroni.

Tests univariés[a]

Ddl du numérateur	Ddl du dénominateur	F	Sig.
1	28	,195	,662

Le test de F permet de tester l'effet de Condition. Il s'appuie sur les comparaisons appariées (indépendantes) linéaires parmi les moyennes marginales estimées.[a]

a. Variable dépendante : Vibrotactile P2 Amplitude ΔOFF-ON.

2.2.5. Vibrotactile P2 Latency (ΔOFF-ON).

```
MIXED VibrotactileP2LatencyΔOFFON BY Condition
  /CRITERIA=CIN(95) MXITER(100) MXSTEP(10) SCORING(1)
SINGULAR(0.000000000001) HCONVERGE(0,
    ABSOLUTE) LCONVERGE(0, ABSOLUTE) PCONVERGE(0.000001, ABSOLUTE)
  /FIXED=Condition | SSTYPE(3)
  /METHOD=REML
  /PRINT=CPS CORB COVB DESCRIPTIVES G  SOLUTION TESTCOV
  /EMMEANS=TABLES(OVERALL)
  /EMMEANS=TABLES(Condition) COMPARE ADJ(BONFERRONI).
```

Remarques

Sortie obtenue		05-MAY-2021 13:17:02
Commentaires		
Entrée	Jeu de données actif	Jeu_de_données5
	Filtre	<sans>
	Pondération	<sans>
	Fichier scindé	<sans>
	N de lignes dans le fichier de travail	30
Gestion des valeurs manquantes	Définition de la valeur manquante	Les valeurs manquantes définies par l'utilisateur sont traitées comme étant manquantes.
	Observations utilisées	Les statistiques sont basées sur toutes les observations comportant des données valides pour toutes les variables du modèle.

Syntaxe		MIXED VibrotactileP2LatencyΔOFFON BY Condition<br>/CRITERIA=CIN(95) MXITER(100) MXSTEP(10) SCORING(1) SINGULAR(0.000000000001) HCONVERGE(0, ABSOLUTE) LCONVERGE(0, ABSOLUTE) PCONVERGE(0.000001, ABSOLUTE)<br>/FIXED=Condition | SSTYPE(3)<br>/METHOD=REML<br>/PRINT=CPS CORB COVB DESCRIPTIVES G SOLUTION TESTCOV<br>/EMMEANS=TABLES(OVERALL)<br>/EMMEANS=TABLES(Condition) COMPARE ADJ(BONFERRONI).
Ressources	Temps de processeur	00:00:00,02
	Temps écoulé	00:00:00,01

Récapitulatif de traitement des observations

		Effectif	Pourcentage marginal
Condition	Sham	15	50,0%
	taVNS	15	50,0%
Valide		30	100,0%
Exclues		0	
Total		30	

Statistiques descriptives

Vibrotactile P2 Latency ΔOFF-ON

Condition	Effectif	Moyenne	Ecart type	Coefficient de variation
Sham	15	,008733333333333	,028932104428587	331,3%
taVNS	15	-,008000000000000	,050672618698014	-633,4%
Total	30	,000366666666667	,041425865094776	11298,0%

Dimension du modèle[a]

		Nombre de niveaux	Nombre de paramètres
Effets fixes	Constante	1	1
	Condition	2	1
Résidu			1
Total		3	3

a. Variable dépendante : Vibrotactile P2 Latency ΔOFF-ON.

Critères d'information[a]

Log de vraisemblance restreint -2	-93,644
Critère d'information d'Akaike (AIC)	-91,644
Critère de Hurvich et Tsai (AICC)	-91,490
Critère de Bozdogan (CAIC)	-89,311
Critère bayésien de Schwartz (BIC)	-90,311

Les critères d'informations sont présentés en plus petit, disposant d'un meilleur format.[a]

a. Variable dépendante : Vibrotactile P2 Latency ΔOFF-ON.

Effets fixes

Tests des effets fixes de type III[a]

Source	Ddl du numérateur	Ddl du dénominateur	F	Sig.
Constante	1	28	,002	,962
Condition	1	28	1,234	,276

a. Variable dépendante : Vibrotactile P2 Latency ΔOFF-ON.

Estimations des effets fixes[a]

Paramètre	Estimation	Erreur standard	ddl	t	Sig.	Intervalle de confiance à 95 % Borne inférieure
Constante	-,008000	,010653	28	-,751	,459	-,029822
[Condition=Sham]	,016733	,015066	28	1,111	,276	-,014128
[Condition=taVNS]	0[b]	0	.	.	.	.

Estimations des effets fixes[a]

Paramètre	Intervalle de confiance à 95 % Borne supérieure
Constante	,013822
[Condition=Sham]	,047595
[Condition=taVNS]	.

a. Variable dépendante : Vibrotactile P2 Latency ΔOFF-ON.

b. Ce paramètre est défini sur 0, car il est redondant.

Matrice de corrélation pour les estimations des effets fixes[a]

Paramètre	Constante	[Condition=Sham]	[Condition=taVNS]
Constante	1	-,707	.[b]
[Condition=Sham]	-,707	1	.[b]
[Condition=taVNS]	.[b]	.[b]	.[b]

a. Variable dépendante : Vibrotactile P2 Latency ΔOFF-ON.

b. La corrélation est manquante par défaut, car elle est associée à un paramètre redondant.

Matrice de covariance pour les estimations des effets fixes[a]

Paramètre	Constante	[Condition=Sham]	[Condition=taVNS]
Constante	,000113	-,000113	0[b]

[Condition=Sham]	-,000113	,000227	0[b]
[Condition=taVNS]	0[b]	0[b]	0[b]

a. Variable dépendante : Vibrotactile P2 Latency ΔOFF-ON.

b. La covariance est définie sur 0, car elle est associée à un paramètre redondant.

Paramètres de covariance

Estimations des paramètres de covariance[a]

Paramètre	Estimation	Erreur standard	Z de Wald	Sig.	Intervalle de confiance à 95 % Borne inférieure	Borne supérieure
Résidu	,001702	,000455	3,742	,000	,001008	,002874

a. Variable dépendante : Vibrotactile P2 Latency ΔOFF-ON.

Matrice de corrélation pour les estimations des paramètres de covariance[a]

Paramètre	Résidu
Résidu	1

a. Variable dépendante : Vibrotactile P2 Latency ΔOFF-ON.

Matrice de covariance pour les estimations des paramètres de covariance[a]

Paramètre	Résidu
Résidu	2,070095E-7

a. Variable dépendante : Vibrotactile P2 Latency ΔOFF-ON.

Moyenne marginale estimée

1. Grand Mean[a]

Moyenne	Erreur standard	ddl	Intervalle de confiance à 95 % Borne inférieure	Borne supérieure
,000	,008	28	-,015	,016

a. Variable dépendante : Vibrotactile P2 Latency ΔOFF-ON.

2. Condition

Estimations[a]

Condition	Moyenne	Erreur standard	ddl	Intervalle de confiance à 95 % Borne inférieure	Borne supérieure
Sham	,009	,011	28	-,013	,031
taVNS	-,008	,011	28	-,030	,014

a. Variable dépendante : Vibrotactile P2 Latency ΔOFF-ON.

Comparaisons appariées[a]

(I) Condition	(J) Condition	Différence moyenne (I-J)	Erreur standard	ddl	Sig.[b]
Sham	taVNS	,017	,015	28	,276
taVNS	Sham	-,017	,015	28	,276

Comparaisons appariées[a]

(I) Condition	(J) Condition	Intervalle de confiance à 95 % pour la différence[b] Borne inférieure	Borne supérieure
Sham	taVNS	-,014	,048
taVNS	Sham	-,048	,014

Basées sur les moyennes marginales estimées[a]

a. Variable dépendante : Vibrotactile P2 Latency ΔOFF-ON.

b. Ajustement pour les comparaisons multiples : Bonferroni.

Tests univariés[a]

Ddl du numérateur	Ddl du dénominateur	F	Sig.
1	28	1,234	,276

Le test de F permet de tester l'effet de Condition. Il s'appuie sur les comparaisons appariées (indépendantes) linéaires parmi les moyennes marginales estimées.[a]

a. Variable dépendante : Vibrotactile P2 Latency ΔOFF-ON.
