# Supplementary material for: Transcutaneous auricular VNS applied to experimental pain: A paired behavioral and EEG study using thermonociceptive CO2 laser
Source: PLoS One. 2021 Jul 12;16(7):e0254480. doi: 10.1371/journal.pone.0254480 (PMC8274876; doi:10.1371/journal.pone.0254480)
Supplement: S1 Appendix — (ZIP) [file pone.0254480.s001.zip › Supplementary Analysis_LMM_Cerebral responses.pdf]

Transcutaneous VNS applied to experimental pain: a paired behavioral and EEG study using thermonociceptive CO2 laser

Supplementary Appendix

Linear Mixed Models: Detailed analysis.

CEREBRAL RESPONSES

1. Experiment 1

1.1 Laser-evoked potentials (LEPs).

1.1.1 LEPs N2P2 Amplitude.

```
MIXED LaserN2P2Amplitude BY Condition Time
  /CRITERIA=CIN(95) MXITER(100) MXSTEP(10) SCORING(1)
SINGULAR(0.000000000001) HCONVERGE(0,
    ABSOLUTE) LCONVERGE(0, ABSOLUTE) PCONVERGE(0.000001, ABSOLUTE)
  /FIXED=Condition Time Condition*Time | SSTYPE(3)
  /METHOD=REML
  /PRINT=CPS CORB COVB DESCRIPTIVES G  SOLUTION TESTCOV
  /EMMEANS=TABLES(OVERALL)
  /EMMEANS=TABLES(Condition) COMPARE ADJ(BONFERRONI)
  /EMMEANS=TABLES(Time) COMPARE ADJ(BONFERRONI)
  /EMMEANS=TABLES(Condition*Time) .
```

Remarques

Sortie obtenue		05-MAY-2021 12:11:24
Commentaires		
Entrée	Jeu de données actif	Jeu_de_données1
	Filtre	<sans>
	Pondération	<sans>
	Fichier scindé	<sans>
	N de lignes dans le fichier de travail	1048530
Gestion des valeurs manquantes	Définition de la valeur manquante	Les valeurs manquantes définies par l'utilisateur sont traitées comme étant manquantes.
	Observations utilisées	Les statistiques sont basées sur toutes les observations comportant des données valides pour toutes les variables du modèle.

Syntaxe		MIXED LaserN2P2Amplitude BY Condition Time<br>/CRITERIA=CIN(95) MXITER(100) MXSTEP(10) SCORING(1) SINGULAR(0.000000000001) HCONVERGE(0, ABSOLUTE) LCONVERGE(0, ABSOLUTE) PCONVERGE(0.000001, ABSOLUTE)<br>/FIXED=Condition Time Condition*Time | SSTYPE(3)<br>/METHOD=REML<br>/PRINT=CPS CORB COVB DESCRIPTIVES G SOLUTION TESTCOV<br>/EMMEANS=TABLES(OVERALL)<br>/EMMEANS=TABLES(Condition) COMPARE ADJ(BONFERRONI)<br>/EMMEANS=TABLES(Time) COMPARE ADJ(BONFERRONI)<br>/EMMEANS=TABLES(Condition*Time) .
Ressources	Temps de processeur	00:00:00,55
	Temps écoulé	00:00:00,56

Récapitulatif de traitement des observations

		Effectif	Pourcentage marginal
Condition	Sham	44	50,0%
	taVNS	44	50,0%
Time	T0	44	50,0%

	T2	44	50,0%
Valide		88	100,0%
Exclues		1048442	
Total		1048530	

Statistiques descriptives

Laser N2P2 Amplitude

Condition	Time	Effectif	Moyenne	Ecart type	Coefficient de variation
Sham	T0	22	17,116626818181818	9,880144822927102	57,7%
	T2	22	16,098913181818183	6,178795752649992	38,4%
	Total	44	16,607770000000000	8,159861352670440	49,1%
taVNS	T0	22	17,050644090909092	7,928772911154279	46,5%
	T2	22	16,787978181818180	7,638998516896630	45,5%
	Total	44	16,919311136363635	7,695322393403041	45,5%
Total	T0	44	17,083635454545455	8,853043918120452	51,8%
	T2	44	16,443445681818180	6,874947843341912	41,8%
	Total	88	16,763540568181817	7,886837723279133	47,0%

Dimension du modèle[a]

		Nombre de niveaux	Nombre de paramètres
Effets fixes	Constante	1	1
	Condition	2	1
	Time	2	1
	Condition * Time	4	1
Résidu			1
Total		9	5

a. Variable dépendante : Laser N2P2 Amplitude.

Critères d'information[a]

Log de vraisemblance restreint -2	600,424
Critère d'information d'Akaike (AIC)	602,424
Critère de Hurvich et Tsai (AICC)	602,473
Critère de Bozdogan (CAIC)	605,855
Critère bayésien de Schwartz (BIC)	604,855

Les critères d'informations sont présentés en plus petit, disposant d'un meilleur format.[a]

a. Variable dépendante : Laser N2P2 Amplitude.

Effets fixes

Tests des effets fixes de type III[a]

Source	Ddl du numérateur	Ddl du dénominateur	F	Sig.
Constante	1	84	384,872	,000
Condition	1	84	,033	,856
Time	1	84	,140	,709
Condition * Time	1	84	,049	,826

a. Variable dépendante : Laser N2P2 Amplitude.

Estimations des effets fixes[a]

Paramètre	Estimation	Erreur standard	ddl	t	Sig.
Constante	16,787978	1,708982	84	9,823	,000
[Condition=Sham]	-,689065	2,416866	84	-,285	,776
[Condition=taVNS]	0[b]	0	.	.	.
[Time=T0]	,262666	2,416866	84	,109	,914
[Time=T2]	0[b]	0	.	.	.
[Condition=Sham] * [Time=T0]	,755048	3,417964	84	,221	,826
[Condition=Sham] * [Time=T2]	0[b]	0	.	.	.
[Condition=taVNS] * [Time=T0]	0[b]	0	.	.	.

[Condition=taVNS] * [Time=T2]	0[b]	0	.	.	.

Estimations des effets fixes[a]

Paramètre	Intervalle de confiance à 95 % Borne inférieure	Borne supérieure
Constante	13,389480	20,186476
[Condition=Sham]	-5,495267	4,117137
[Condition=taVNS]	.	.
[Time=T0]	-4,543536	5,068868
[Time=T2]	.	.
[Condition=Sham] * [Time=T0]	-6,041949	7,552044
[Condition=Sham] * [Time=T2]	.	.
[Condition=taVNS] * [Time=T0]	.	.
[Condition=taVNS] * [Time=T2]	.	.

a. Variable dépendante : Laser N2P2 Amplitude.

b. Ce paramètre est défini sur 0, car il est redondant.

Matrice de corrélation pour les estimations des effets fixes[a]

Paramètre	Constante	[Condition=Sham]	[Condition=taVNS]	[Time=T0]	[Time=T2]
Constante	1	-,707	.[b]	-,707	.[b]
[Condition=Sham]	-,707	1	.[b]	,500	.[b]
[Condition=taVNS]	.[b]	.[b]	.[b]	.[b]	.[b]
[Time=T0]	-,707	,500	.[b]	1	.[b]
[Time=T2]	.[b]	.[b]	.[b]	.[b]	.[b]
[Condition=Sham] * [Time=T0]	,500	-,707	.[b]	-,707	.[b]
[Condition=Sham] * [Time=T2]	.[b]	.[b]	.[b]	.[b]	.[b]
[Condition=taVNS] * [Time=T0]	.[b]	.[b]	.[b]	.[b]	.[b]
[Condition=taVNS] * [Time=T2]	.[b]	.[b]	.[b]	.[b]	.[b]

Matrice de corrélation pour les estimations des effets fixes[a]

Paramètre	[Condition=Sham] * [Time=T0]	[Condition=Sham] * [Time=T2]	[Condition=taVNS] * [Time=T0]	[Condition=taVNS] * [Time=T2]
Constante	,500	.[b]	.[b]	.[b]
[Condition=Sham]	-,707	.[b]	.[b]	.[b]
[Condition=taVNS]	.[b]	.[b]	.[b]	.[b]

[Time=T0]	-,707	.[b]	.[b]	.[b]
[Time=T2]	.[b]	.[b]	.[b]	.[b]
[Condition=Sham] * [Time=T0]	1	.[b]	.[b]	.[b]
[Condition=Sham] * [Time=T2]	.[b]	.[b]	.[b]	.[b]
[Condition=taVNS] * [Time=T0]	.[b]	.[b]	.[b]	.[b]
[Condition=taVNS] * [Time=T2]	.[b]	.[b]	.[b]	.[b]

a. Variable dépendante : Laser N2P2 Amplitude.

b. La corrélation est manquante par défaut, car elle est associée à un paramètre redondant.

Matrice de covariance pour les estimations des effets fixes[a]

Paramètre	Constante	[Condition=Sham]	[Condition=taVNS]	[Time=T0]	[Time=T2]
Constante	2,920620	-2,920620	0[b]	-2,920620	0[b]
[Condition=Sham]	-2,920620	5,841239	0[b]	2,920620	0[b]
[Condition=taVNS]	0[b]	0[b]	0[b]	0[b]	0[b]
[Time=T0]	-2,920620	2,920620	0[b]	5,841239	0[b]
[Time=T2]	0[b]	0[b]	0[b]	0[b]	0[b]
[Condition=Sham] * [Time=T0]	2,920620	-5,841239	0[b]	-5,841239	0[b]
[Condition=Sham] * [Time=T2]	0[b]	0[b]	0[b]	0[b]	0[b]
[Condition=taVNS] * [Time=T0]	0[b]	0[b]	0[b]	0[b]	0[b]
[Condition=taVNS] * [Time=T2]	0[b]	0[b]	0[b]	0[b]	0[b]

Matrice de covariance pour les estimations des effets fixes[a]

Paramètre	[Condition=Sham] * [Time=T0]	[Condition=Sham] * [Time=T2]	[Condition=taVNS] * [Time=T0]	[Condition=taVNS] * [Time=T2]
Constante	2,920620	0[b]	0[b]	0[b]
[Condition=Sham]	-5,841239	0[b]	0[b]	0[b]
[Condition=taVNS]	0[b]	0[b]	0[b]	0[b]
[Time=T0]	-5,841239	0[b]	0[b]	0[b]
[Time=T2]	0[b]	0[b]	0[b]	0[b]
[Condition=Sham] * [Time=T0]	11,682478	0[b]	0[b]	0[b]
[Condition=Sham] * [Time=T2]	0[b]	0[b]	0[b]	0[b]
[Condition=taVNS] * [Time=T0]	0[b]	0[b]	0[b]	0[b]
[Condition=taVNS] * [Time=T2]	0[b]	0[b]	0[b]	0[b]

a. Variable dépendante : Laser N2P2 Amplitude.

b. La covariance est définie sur 0, car elle est associée à un paramètre redondant.

Paramètres de covariance

Estimations des paramètres de covariance[a]

Paramètre	Estimation	Erreur standard	Z de Wald	Sig.	Intervalle de confiance à 95 % Borne inférieure	Borne supérieure
Résidu	64,253629	9,914550	6,481	,000	47,484778	86,944259

a. Variable dépendante : Laser N2P2 Amplitude.

Matrice de corrélation pour les estimations des paramètres de covariance[a]

Paramètre	Résidu
Résidu	1

a. Variable dépendante : Laser N2P2 Amplitude.

Matrice de covariance pour les estimations des paramètres de covariance[a]

Paramètre	Résidu
Résidu	98,298306

a. Variable dépendante : Laser N2P2 Amplitude.

Moyenne marginale estimée

1. Grand Mean[a]

Moyenne	Erreur standard	ddl	Intervalle de confiance à 95 % Borne inférieure	Borne supérieure
16,764	,854	84	15,064	18,463

a. Variable dépendante : Laser N2P2 Amplitude.

2. Condition

Estimations[a]

Condition	Moyenne	Erreur standard	ddl	Intervalle de confiance à 95 % Borne inférieure	Borne supérieure
Sham	16,608	1,208	84	14,205	19,011
taVNS	16,919	1,208	84	14,516	19,322

a. Variable dépendante : Laser N2P2 Amplitude.

Comparaisons appariées[a]

(I) Condition	(J) Condition	Différence moyenne (I-J)	Erreur standard	ddl	Sig.[b]
Sham	taVNS	-,312	1,709	84	,856
taVNS	Sham	,312	1,709	84	,856

Comparaisons appariées[a]

(I) Condition	(J) Condition	Intervalle de confiance à 95 % pour la différence[b] Borne inférieure	Borne supérieure
Sham	taVNS	-3,710	3,087
taVNS	Sham	-3,087	3,710

Basées sur les moyennes marginales estimées[a]

a. Variable dépendante : Laser N2P2 Amplitude.

b. Ajustement pour les comparaisons multiples : Bonferroni.

Tests univariés[a]

Ddl du numérateur	Ddl du dénominateur	F	Sig.
1	84	,033	,856

Le test de F permet de tester l'effet de Condition. Il s'appuie sur les comparaisons appariées (indépendantes) linéaires parmi les moyennes marginales estimées.[a]

a. Variable dépendante : Laser N2P2 Amplitude.

3. Time

Estimations[a]

Time	Moyenne	Erreur standard	ddl	Intervalle de confiance à 95 % Borne inférieure	Borne supérieure
T0	17,084	1,208	84	14,681	19,487
T2	16,443	1,208	84	14,040	18,847

a. Variable dépendante : Laser N2P2 Amplitude.

Comparaisons appariées[a]

(I) Time	(J) Time	Différence moyenne (I-J)	Erreur standard	ddl	Sig.[b]	Intervalle de confiance à 95 % pour la différence[b] Borne inférieure
T0	T2	,640	1,709	84	,709	-2,758
T2	T0	-,640	1,709	84	,709	-4,039

Comparaisons appariées[a]

(I) Time	(J) Time	Intervalle de confiance à 95 % pour la différence Borne supérieure
T0	T2	4,039
T2	T0	2,758

Basées sur les moyennes marginales estimées[a]

a. Variable dépendante : Laser N2P2 Amplitude.

b. Ajustement pour les comparaisons multiples : Bonferroni.

Tests univariés[a]

Ddl du numérateur	Ddl du dénominateur	F	Sig.
1	84	,140	,709

Le test de F permet de tester l'effet de Time. Il s'appuie sur les comparaisons appariées (indépendantes) linéaires parmi les moyennes marginales estimées.[a]

a. Variable dépendante : Laser N2P2 Amplitude.

4. Condition * Time[a]

Condition	Time	Moyenne	Erreur standard	ddl	Intervalle de confiance à 95 % Borne inférieure	Intervalle de confiance à 95 % Borne supérieure
Sham	T0	17,117	1,709	84	13,718	20,515
	T2	16,099	1,709	84	12,700	19,497
taVNS	T0	17,051	1,709	84	13,652	20,449
	T2	16,788	1,709	84	13,389	20,186

a. Variable dépendante : Laser N2P2 Amplitude.

1.1.2. LEPs N2 Amplitude.

```
MIXED LaserN2Amplitude BY Condition Time
  /CRITERIA=CIN(95) MXITER(100) MXSTEP(10) SCORING(1)
SINGULAR(0.000000000001) HCONVERGE(0,
    ABSOLUTE) LCONVERGE(0, ABSOLUTE) PCONVERGE(0.000001, ABSOLUTE)
  /FIXED=Condition Time Condition*Time | SSTYPE(3)
  /METHOD=REML
  /PRINT=CPS CORB COVB DESCRIPTIVES G  SOLUTION TESTCOV
  /EMMEANS=TABLES(OVERALL)
  /EMMEANS=TABLES(Condition) COMPARE ADJ(BONFERRONI)
  /EMMEANS=TABLES(Time) COMPARE ADJ(BONFERRONI)
  /EMMEANS=TABLES(Condition*Time) .
```

Remarques

Sortie obtenue		05-MAY-2021 12:12:16
Commentaires		
Entrée	Jeu de données actif	Jeu_de_données1
	Filtre	<sans>
	Pondération	<sans>
	Fichier scindé	<sans>
	N de lignes dans le fichier de travail	1048530
Gestion des valeurs manquantes	Définition de la valeur manquante	Les valeurs manquantes définies par l'utilisateur sont traitées comme étant manquantes.
	Observations utilisées	Les statistiques sont basées sur toutes les observations comportant des données valides pour toutes les variables du modèle.

Syntaxe		MIXED LaserN2Amplitude BY Condition Time /CRITERIA=CIN(95) MXITER(100) MXSTEP(10) SCORING(1) SINGULAR(0.000000000001) HCONVERGE(0, ABSOLUTE) LCONVERGE(0, ABSOLUTE) PCONVERGE(0.000001, ABSOLUTE) /FIXED=Condition Time Condition*Time | SSTYPE(3) /METHOD=REML /PRINT=CPS CORB COVB DESCRIPTIVES G SOLUTION TESTCOV /EMMEANS=TABLES(OVERALL) /EMMEANS=TABLES(Condition) COMPARE ADJ(BONFERRONI) /EMMEANS=TABLES(Time) COMPARE ADJ(BONFERRONI) /EMMEANS=TABLES(Condition*Time) .
Ressources	Temps de processeur	00:00:00,58
	Temps écoulé	00:00:00,58

Récapitulatif de traitement des observations

		Effectif	Pourcentage marginal
Condition	Sham	44	50,0%
	taVNS	44	50,0%
Time	T0	44	50,0%

	T2	44	50,0%
Valide		88	100,0%
Exclues		1048442	
Total		1048530	

Statistiques descriptives

Laser N2 Amplitude

Condition	Time	Effectif	Moyenne	Ecart type	Coefficient de variation
Sham	T0	22	-6,8216177	4,94306670	-72,5%
	T2	22	-6,3042859	4,21934881	-66,9%
	Total	44	-6,5629518	4,54925760	-69,3%
taVNS	T0	22	-6,5211555	3,95284756	-60,6%
	T2	22	-6,0631318	4,81369042	-79,4%
	Total	44	-6,2921436	4,35899671	-69,3%
Total	T0	44	-6,6713866	4,42569193	-66,3%
	T2	44	-6,1837089	4,47500715	-72,4%
	Total	88	-6,4275477	4,43155753	-68,9%

Dimension du modèle[a]

		Nombre de niveaux	Nombre de paramètres
Effets fixes	Constante	1	1
	Condition	2	1
	Time	2	1
	Condition * Time	4	1
Résidu			1
Total		9	5

a. Variable dépendante : Laser N2 Amplitude.

Critères d'information[a]

Log de vraisemblance restreint -2	503,466
Critère d'information d'Akaike (AIC)	505,466
Critère de Hurvich et Tsai (AICC)	505,514
Critère de Bozdogan (CAIC)	508,896
Critère bayésien de Schwartz (BIC)	507,896

Les critères d'informations sont présentés en plus petit, disposant d'un meilleur format.[a]

a. Variable dépendante : Laser N2 Amplitude.

Effets fixes

Tests des effets fixes de type III[a]

Source	Ddl du numérateur	Ddl du dénominateur	F	Sig.
Constante	1	84	179,461	,000
Condition	1	84	,080	,778
Time	1	84	,258	,613
Condition * Time	1	84	,001	,975

a. Variable dépendante : Laser N2 Amplitude.

Estimations des effets fixes[a]

Paramètre	Estimation	Erreur standard	ddl	t	Sig.
Constante	-6,063132	,959601	84	-6,318	,000
[Condition=Sham]	-,241154	1,357081	84	-,178	,859
[Condition=taVNS]	0[b]	0	.	.	.
[Time=T0]	-,458024	1,357081	84	-,338	,737
[Time=T2]	0[b]	0	.	.	.
[Condition=Sham] * [Time=T0]	-,059308	1,919202	84	-,031	,975
[Condition=Sham] * [Time=T2]	0[b]	0	.	.	.
[Condition=taVNS] * [Time=T0]	0[b]	0	.	.	.
[Condition=taVNS] * [Time=T2]	0[b]	0	.	.	.

Estimations des effets fixes[a]

Paramètre	Intervalle de confiance à 95 % Borne inférieure	Borne supérieure
Constante	-7,971404	-4,154860
[Condition=Sham]	-2,939859	2,457550
[Condition=taVNS]	.	.
[Time=T0]	-3,156728	2,240681
[Time=T2]	.	.

[Condition=Sham] * [Time=T0]	-3,875853	3,757236
[Condition=Sham] * [Time=T2]	.	.
[Condition=taVNS] * [Time=T0]	.	.
[Condition=taVNS] * [Time=T2]	.	.

a. Variable dépendante : Laser N2 Amplitude.

b. Ce paramètre est défini sur 0, car il est redondant.

Matrice de corrélation pour les estimations des effets fixes[a]

Paramètre	Constante	[Condition=Sham]	[Condition=taVNS]	[Time=T0]	[Time=T2]
Constante	1	-,707	.[b]	-,707	.[b]
[Condition=Sham]	-,707	1	.[b]	,500	.[b]
[Condition=taVNS]	.[b]	.[b]	.[b]	.[b]	.[b]
[Time=T0]	-,707	,500	.[b]	1	.[b]
[Time=T2]	.[b]	.[b]	.[b]	.[b]	.[b]
[Condition=Sham] * [Time=T0]	,500	-,707	.[b]	-,707	.[b]
[Condition=Sham] * [Time=T2]	.[b]	.[b]	.[b]	.[b]	.[b]
[Condition=taVNS] * [Time=T0]	.[b]	.[b]	.[b]	.[b]	.[b]
[Condition=taVNS] * [Time=T2]	.[b]	.[b]	.[b]	.[b]	.[b]

Matrice de corrélation pour les estimations des effets fixes[a]

Paramètre	[Condition=Sham] * [Time=T0]	[Condition=Sham] * [Time=T2]	[Condition=taVNS] * [Time=T0]	[Condition=taVNS] * [Time=T2]
Constante	,500	.[b]	.[b]	.[b]
[Condition=Sham]	-,707	.[b]	.[b]	.[b]
[Condition=taVNS]	.[b]	.[b]	.[b]	.[b]
[Time=T0]	-,707	.[b]	.[b]	.[b]
[Time=T2]	.[b]	.[b]	.[b]	.[b]
[Condition=Sham] * [Time=T0]	1	.[b]	.[b]	.[b]
[Condition=Sham] * [Time=T2]	.[b]	.[b]	.[b]	.[b]
[Condition=taVNS] * [Time=T0]	.[b]	.[b]	.[b]	.[b]
[Condition=taVNS] * [Time=T2]	.[b]	.[b]	.[b]	.[b]

a. Variable dépendante : Laser N2 Amplitude.

b. La corrélation est manquante par défaut, car elle est associée à un paramètre redondant.

Matrice de covariance pour les estimations des effets fixes[a]

Paramètre	Constante	[Condition=Sham]	[Condition=taVNS]	[Time=T0]	[Time=T2]
Constante	,920834	-,920834	0[b]	-,920834	0[b]
[Condition=Sham]	-,920834	1,841669	0[b]	,920834	0[b]
[Condition=taVNS]	0[b]	0[b]	0[b]	0[b]	0[b]
[Time=T0]	-,920834	,920834	0[b]	1,841669	0[b]
[Time=T2]	0[b]	0[b]	0[b]	0[b]	0[b]
[Condition=Sham] * [Time=T0]	,920834	-1,841669	0[b]	-1,841669	0[b]
[Condition=Sham] * [Time=T2]	0[b]	0[b]	0[b]	0[b]	0[b]
[Condition=taVNS] * [Time=T0]	0[b]	0[b]	0[b]	0[b]	0[b]
[Condition=taVNS] * [Time=T2]	0[b]	0[b]	0[b]	0[b]	0[b]

Matrice de covariance pour les estimations des effets fixes[a]

Paramètre	[Condition=Sham] * [Time=T0]	[Condition=Sham] * [Time=T2]	[Condition=taVNS] * [Time=T0]	[Condition=taVNS] * [Time=T2]
Constante	,920834	0[b]	0[b]	0[b]
[Condition=Sham]	-1,841669	0[b]	0[b]	0[b]
[Condition=taVNS]	0[b]	0[b]	0[b]	0[b]
[Time=T0]	-1,841669	0[b]	0[b]	0[b]
[Time=T2]	0[b]	0[b]	0[b]	0[b]
[Condition=Sham] * [Time=T0]	3,683338	0[b]	0[b]	0[b]
[Condition=Sham] * [Time=T2]	0[b]	0[b]	0[b]	0[b]
[Condition=taVNS] * [Time=T0]	0[b]	0[b]	0[b]	0[b]
[Condition=taVNS] * [Time=T2]	0[b]	0[b]	0[b]	0[b]

a. Variable dépendante : Laser N2 Amplitude.

b. La covariance est définie sur 0, car elle est associée à un paramètre redondant.

Paramètres de covariance

Estimations des paramètres de covariance[a]

Paramètre	Estimation	Erreur standard	Z de Wald	Sig.	Intervalle de confiance à 95 % Borne inférieure	Intervalle de confiance à 95 % Borne supérieure
Résidu	20,258358	3,125933	6,481	,000	14,971351	27,412427

a. Variable dépendante : Laser N2 Amplitude.

Matrice de corrélation pour les estimations des paramètres de covariance[a]

Paramètre	Résidu
Résidu	1

a. Variable dépendante : Laser N2 Amplitude.

Matrice de covariance pour les estimations des paramètres de covariance[a]

Paramètre	Résidu
Résidu	9,771454

a. Variable dépendante : Laser N2 Amplitude.

Moyenne marginale estimée

1. Grand Mean[a]

Moyenne	Erreur standard	ddl	Intervalle de confiance à 95 % Borne inférieure	Intervalle de confiance à 95 % Borne supérieure
-6,428	,480	84	-7,382	-5,473

a. Variable dépendante : Laser N2 Amplitude.

2. Condition

Estimations[a]

Condition	Moyenne	Erreur standard	ddl	Intervalle de confiance à 95 % Borne inférieure	Intervalle de confiance à 95 % Borne supérieure
Sham	-6,563	,679	84	-7,912	-5,214
taVNS	-6,292	,679	84	-7,641	-4,943

a. Variable dépendante : Laser N2 Amplitude.

Comparaisons appariées[a]

(I) Condition	(J) Condition	Différence moyenne (I-J)	Erreur standard	ddl	Sig.[b]
Sham	taVNS	-,271	,960	84	,778
taVNS	Sham	,271	,960	84	,778

Comparaisons appariées[a]

(I) Condition	(J) Condition	Intervalle de confiance à 95 % pour la différence[b]	
		Borne inférieure	Borne supérieure
Sham	taVNS	-2,179	1,637
taVNS	Sham	-1,637	2,179

Basées sur les moyennes marginales estimées[a]

a. Variable dépendante : Laser N2 Amplitude.

b. Ajustement pour les comparaisons multiples : Bonferroni.

Tests univariés[a]

Ddl du numérateur	Ddl du dénominateur	F	Sig.
1	84	,080	,778

Le test de F permet de tester l'effet de Condition. Il s'appuie sur les comparaisons appariées (indépendantes) linéaires parmi les moyennes marginales estimées.[a]

a. Variable dépendante : Laser N2 Amplitude.

3. Time

Estimations[a]

Time	Moyenne	Erreur standard	ddl	Intervalle de confiance à 95 %	
				Borne inférieure	Borne supérieure
T0	-6,671	,679	84	-8,021	-5,322
T2	-6,184	,679	84	-7,533	-4,834

a. Variable dépendante : Laser N2 Amplitude.

Comparaisons appariées[a]

(I) Time	(J) Time	Différence moyenne (I-J)	Erreur standard	ddl	Sig.[b]	Intervalle de confiance à 95 % pour la différence[b] Borne inférieure
T0	T2	-,488	,960	84	,613	-2,396
T2	T0	,488	,960	84	,613	-1,421

Comparaisons appariées[a]

(I) Time	(J) Time	Intervalle de confiance à 95 % pour la différence Borne supérieure
T0	T2	1,421
T2	T0	2,396

Basées sur les moyennes marginales estimées[a]

a. Variable dépendante : Laser N2 Amplitude.

b. Ajustement pour les comparaisons multiples : Bonferroni.

Tests univariés[a]

Ddl du numérateur	Ddl du dénominateur	F	Sig.
1	84	,258	,613

Le test de F permet de tester l'effet de Time. Il s'appuie sur les comparaisons appariées (indépendantes) linéaires parmi les moyennes marginales estimées.[a]

a. Variable dépendante : Laser N2 Amplitude.

4. Condition * Time[a]

Condition	Time	Moyenne	Erreur standard	ddl	Intervalle de confiance à 95 % Borne inférieure	Borne supérieure
Sham	T0	-6,822	,960	84	-8,730	-4,913
	T2	-6,304	,960	84	-8,213	-4,396
taVNS	T0	-6,521	,960	84	-8,429	-4,613
	T2	-6,063	,960	84	-7,971	-4,155

a. Variable dépendante : Laser N2 Amplitude.

1.1.3. LEPs N2 Latency.

```
MIXED LaserN2Latency BY Condition Time
  /CRITERIA=CIN(95) MXITER(100) MXSTEP(10) SCORING(1)
SINGULAR(0.000000000001) HCONVERGE(0,
    ABSOLUTE) LCONVERGE(0, ABSOLUTE) PCONVERGE(0.000001, ABSOLUTE)
  /FIXED=Condition Time Condition*Time | SSTYPE(3)
  /METHOD=REML
  /PRINT=CPS CORB COVB DESCRIPTIVES G  SOLUTION TESTCOV
  /EMMEANS=TABLES(OVERALL)
  /EMMEANS=TABLES(Condition) COMPARE ADJ(BONFERRONI)
  /EMMEANS=TABLES(Time) COMPARE ADJ(BONFERRONI)
  /EMMEANS=TABLES(Condition*Time) .
```

Remarques

Sortie obtenue		05-MAY-2021 12:12:47
Commentaires		
Entrée	Jeu de données actif	Jeu_de_données1
	Filtre	<sans>
	Pondération	<sans>
	Fichier scindé	<sans>
	N de lignes dans le fichier de travail	1048530
Gestion des valeurs manquantes	Définition de la valeur manquante	Les valeurs manquantes définies par l'utilisateur sont traitées comme étant manquantes.
	Observations utilisées	Les statistiques sont basées sur toutes les observations comportant des données valides pour toutes les variables du modèle.

Syntaxe		MIXED LaserN2Latency BY Condition Time<br>/CRITERIA=CIN(95) MXITER(100) MXSTEP(10) SCORING(1) SINGULAR(0.000000000001) HCONVERGE(0, ABSOLUTE) LCONVERGE(0, ABSOLUTE) PCONVERGE(0.000001, ABSOLUTE)<br>/FIXED=Condition Time Condition*Time | SSTYPE(3)<br>/METHOD=REML<br>/PRINT=CPS CORB COVB DESCRIPTIVES G SOLUTION TESTCOV<br>/EMMEANS=TABLES(OVERALL)<br>/EMMEANS=TABLES(Condition) COMPARE ADJ(BONFERRONI)<br>/EMMEANS=TABLES(Time) COMPARE ADJ(BONFERRONI)<br>/EMMEANS=TABLES(Condition*Time) .
Ressources	Temps de processeur	00:00:00,56
	Temps écoulé	00:00:00,56

Récapitulatif de traitement des observations

		Effectif	Pourcentage marginal
Condition	Sham	43	50,0%
	taVNS	43	50,0%
Time	T0	42	48,8%

	T2	44	51,2%
Valide		86	100,0%
Exclues		1048444	
Total		1048530	

Statistiques descriptives

Laser N2 Latency

Condition	Time	Effectif	Moyenne	Ecart type	Coefficient de variation
Sham	T0	21	,2110433	,03209447	15,2%
	T2	22	,2061805	,02863284	13,9%
	Total	43	,2085553	,03010767	14,4%
taVNS	T0	21	,2179005	,01706657	7,8%
	T2	22	,1990436	,02382104	12,0%
	Total	43	,2082528	,02265794	10,9%
Total	T0	42	,2144719	,02562400	11,9%
	T2	44	,2026120	,02627811	13,0%
	Total	86	,2084041	,02648770	12,7%

Dimension du modèle[a]

		Nombre de niveaux	Nombre de paramètres
Effets fixes	Constante	1	1
	Condition	2	1
	Time	2	1
	Condition * Time	4	1
Résidu			1
Total		9	5

a. Variable dépendante : Laser N2 Latency .

Critères d'information[a]

Log de vraisemblance restreint -2	-353,379
Critère d'information d'Akaike (AIC)	-351,379
Critère de Hurvich et Tsai (AICC)	-351,329
Critère de Bozdogan (CAIC)	-347,972
Critère bayésien de Schwartz (BIC)	-348,972

Les critères d'informations sont présentés en plus petit, disposant d'un meilleur format.[a]

a. Variable dépendante : Laser N2 Latency
.

Effets fixes

Tests des effets fixes de type III[a]

Source	Ddl du numérateur	Ddl du dénominateur	F	Sig.
Constante	1	82	5517,051	,000
Condition	1	82	,001	,980
Time	1	82	4,461	,038
Condition * Time	1	82	1,553	,216

a. Variable dépendante : Laser N2 Latency .

Estimations des effets fixes[a]

Paramètre	Estimation	Erreur standard	ddl	t	Sig.
Constante	,199044	,005550	82	35,866	,000
[Condition=Sham]	,007137	,007848	82	,909	,366
[Condition=taVNS]	0[b]	0	.	.	.
[Time=T0]	,018857	,007941	82	2,375	,020
[Time=T2]	0[b]	0	.	.	.
[Condition=Sham] * [Time=T0]	-,013994	,011231	82	-1,246	,216
[Condition=Sham] * [Time=T2]	0[b]	0	.	.	.
[Condition=taVNS] * [Time=T0]	0[b]	0	.	.	.
[Condition=taVNS] * [Time=T2]	0[b]	0	.	.	.

Estimations des effets fixes[a]

Paramètre	Intervalle de confiance à 95 %	
	Borne inférieure	Borne supérieure
Constante	,188004	,210084
[Condition=Sham]	-,008476	,022750
[Condition=taVNS]	.	.
[Time=T0]	,003059	,034654
[Time=T2]	.	.

[Condition=Sham] * [Time=T0]	-,036335	,008347
[Condition=Sham] * [Time=T2]	.	.
[Condition=taVNS] * [Time=T0]	.	.
[Condition=taVNS] * [Time=T2]	.	.

a. Variable dépendante : Laser N2 Latency .

b. Ce paramètre est défini sur 0, car il est redondant.

Matrice de corrélation pour les estimations des effets fixes[a]

Paramètre	Constante	[Condition=Sham]	[Condition=taVNS]	[Time=T0]	[Time=T2]
Constante	1	-,707	.[b]	-,699	.[b]
[Condition=Sham]	-,707	1	.[b]	,494	.[b]
[Condition=taVNS]	.[b]	.[b]	.[b]	.[b]	.[b]
[Time=T0]	-,699	,494	.[b]	1	.[b]
[Time=T2]	.[b]	.[b]	.[b]	.[b]	.[b]
[Condition=Sham] * [Time=T0]	,494	-,699	.[b]	-,707	.[b]
[Condition=Sham] * [Time=T2]	.[b]	.[b]	.[b]	.[b]	.[b]
[Condition=taVNS] * [Time=T0]	.[b]	.[b]	.[b]	.[b]	.[b]
[Condition=taVNS] * [Time=T2]	.[b]	.[b]	.[b]	.[b]	.[b]

Matrice de corrélation pour les estimations des effets fixes[a]

Paramètre	[Condition=Sham] * [Time=T0]	[Condition=Sham] * [Time=T2]	[Condition=taVNS] * [Time=T0]	[Condition=taVNS] * [Time=T2]
Constante	,494	.[b]	.[b]	.[b]
[Condition=Sham]	-,699	.[b]	.[b]	.[b]
[Condition=taVNS]	.[b]	.[b]	.[b]	.[b]
[Time=T0]	-,707	.[b]	.[b]	.[b]
[Time=T2]	.[b]	.[b]	.[b]	.[b]
[Condition=Sham] * [Time=T0]	1	.[b]	.[b]	.[b]
[Condition=Sham] * [Time=T2]	.[b]	.[b]	.[b]	.[b]
[Condition=taVNS] * [Time=T0]	.[b]	.[b]	.[b]	.[b]
[Condition=taVNS] * [Time=T2]	.[b]	.[b]	.[b]	.[b]

a. Variable dépendante : Laser N2 Latency .

b. La corrélation est manquante par défaut, car elle est associée à un paramètre redondant.

Matrice de covariance pour les estimations des effets fixes[a]

Paramètre	Constante	[Condition=Sham]	[Condition=taVNS]	[Time=T0]
Constante	3,079788E-5	-3,079788E-5	0[b]	-3,079788E-5
[Condition=Sham]	-3,079788E-5	6,159576E-5	0[b]	3,079788E-5
[Condition=taVNS]	0[b]	0[b]	0[b]	0[b]
[Time=T0]	-3,079788E-5	3,079788E-5	0[b]	6,306233E-5
[Time=T2]	0[b]	0[b]	0[b]	0[b]
[Condition=Sham] * [Time=T0]	3,079788E-5	-6,159576E-5	0[b]	-6,306233E-5
[Condition=Sham] * [Time=T2]	0[b]	0[b]	0[b]	0[b]
[Condition=taVNS] * [Time=T0]	0[b]	0[b]	0[b]	0[b]
[Condition=taVNS] * [Time=T2]	0[b]	0[b]	0[b]	0[b]

Matrice de covariance pour les estimations des effets fixes[a]

Paramètre	[Time=T2]	[Condition=Sham] * [Time=T0]	[Condition=Sham] * [Time=T2]	[Condition=taVNS] * [Time=T0]
Constante	0[b]	3,079788E-5	0[b]	0[b]
[Condition=Sham]	0[b]	-6,159576E-5	0[b]	0[b]
[Condition=taVNS]	0[b]	0[b]	0[b]	0[b]
[Time=T0]	0[b]	-6,306233E-5	0[b]	0[b]
[Time=T2]	0[b]	0[b]	0[b]	0[b]
[Condition=Sham] * [Time=T0]	0[b]	,000126	0[b]	0[b]
[Condition=Sham] * [Time=T2]	0[b]	0[b]	0[b]	0[b]
[Condition=taVNS] * [Time=T0]	0[b]	0[b]	0[b]	0[b]
[Condition=taVNS] * [Time=T2]	0[b]	0[b]	0[b]	0[b]

Matrice de covariance pour les estimations des effets fixes[a]

Paramètre	[Condition=taVNS] * [Time=T2]
Constante	0[b]
[Condition=Sham]	0[b]
[Condition=taVNS]	0[b]
[Time=T0]	0[b]
[Time=T2]	0[b]
[Condition=Sham] * [Time=T0]	0[b]
[Condition=Sham] * [Time=T2]	0[b]
[Condition=taVNS] * [Time=T0]	0[b]
[Condition=taVNS] * [Time=T2]	0[b]

a. Variable dépendante : Laser N2 Latency .

b. La covariance est définie sur 0, car elle est associée à un paramètre redondant.

Paramètres de covariance

Estimations des paramètres de covariance[a]

Paramètre	Estimation	Erreur standard	Z de Wald	Sig.	Intervalle de confiance à 95 %	
					Borne inférieure	Borne supérieure
Résidu	,000678	,000106	6,403	,000	,000499	,000920

a. Variable dépendante : Laser N2 Latency .

Matrice de corrélation pour les estimations des paramètres de covariance[a]

Paramètre	Résidu
Résidu	1

a. Variable dépendante : Laser N2 Latency .

Matrice de covariance pour les estimations des paramètres de covariance[a]

Paramètre	Résidu
Résidu	1,119704E-8

a. Variable dépendante : Laser N2 Latency .

Moyenne marginale estimée

1. Grand Mean[a]

Moyenne	Erreur standard	ddl	Intervalle de confiance à 95 %	
			Borne inférieure	Borne supérieure
,209	,003	82	,203	,214

a. Variable dépendante : Laser N2 Latency .

2. Condition

Estimations[a]

Condition	Moyenne	Erreur standard	ddl	Intervalle de confiance à 95 %	
				Borne inférieure	Borne supérieure
Sham	,209	,004	82	,201	,217
taVNS	,208	,004	82	,201	,216

a. Variable dépendante : Laser N2 Latency .

Comparaisons appariées[a]

(I) Condition	(J) Condition	Différence moyenne (I-J)	Erreur standard	ddl	Sig.[b]
Sham	taVNS	,000	,006	82	,980
taVNS	Sham	,000	,006	82	,980

Comparaisons appariées[a]

(I) Condition	(J) Condition	Intervalle de confiance à 95 % pour la différence[b]	
		Borne inférieure	Borne supérieure
Sham	taVNS	-,011	,011
taVNS	Sham	-,011	,011

Basées sur les moyennes marginales estimées[a]

a. Variable dépendante : Laser N2 Latency .

b. Ajustement pour les comparaisons multiples : Bonferroni.

Tests univariés[a]

Ddl du numérateur	Ddl du dénominateur	F	Sig.
1	82	,001	,980

Le test de F permet de tester l'effet de Condition. Il s'appuie sur les comparaisons appariées (indépendantes) linéaires parmi les moyennes marginales estimées.[a]

a. Variable dépendante : Laser N2 Latency .

3. Time

Estimations[a]

Time	Moyenne	Erreur standard	ddl	Intervalle de confiance à 95 % Borne inférieure	Intervalle de confiance à 95 % Borne supérieure
T0	,214	,004	82	,206	,222
T2	,203	,004	82	,195	,210

a. Variable dépendante : Laser N2 Latency .

Comparaisons appariées[a]

(I) Time	(J) Time	Différence moyenne (I-J)	Erreur standard	ddl	Sig.[c]	Intervalle de confiance à 95 % pour la différence[c] Borne inférieure
T0	T2	,012*	,006	82	,038	,001
T2	T0	-,012*	,006	82	,038	-,023

Comparaisons appariées[a]

(I) Time	(J) Time	Intervalle de confiance à 95 % pour la différence Borne supérieure
T0	T2	,023
T2	T0	-,001

Basées sur les moyennes marginales estimées[a]

*. La différence moyenne est significative au niveau ,05.

a. Variable dépendante : Laser N2 Latency .

c. Ajustement pour les comparaisons multiples : Bonferroni.

Tests univariés[a]

Ddl du numérateur	Ddl du dénominateur	F	Sig.
1	82	4,461	,038

Le test de F permet de tester l'effet de Time. Il s'appuie sur les comparaisons appariées (indépendantes) linéaires parmi les moyennes marginales estimées.[a]

a. Variable dépendante : Laser N2 Latency .

4. Condition * Time[a]

Condition	Time	Moyenne	Erreur standard	ddl	Intervalle de confiance à 95 % Borne inférieure	Borne supérieure
Sham	T0	,211	,006	82	,200	,222
	T2	,206	,006	82	,195	,217
taVNS	T0	,218	,006	82	,207	,229
	T2	,199	,006	82	,188	,210

a. Variable dépendante : Laser N2 Latency .

1.1.4. LEPs P2 Amplitude.

```
MIXED LaserP2Amplitude BY Condition Time
  /CRITERIA=CIN(95) MXITER(100) MXSTEP(10) SCORING(1)
SINGULAR(0.000000000001) HCONVERGE(0,
    ABSOLUTE) LCONVERGE(0, ABSOLUTE) PCONVERGE(0.000001, ABSOLUTE)
  /FIXED=Condition Time Condition*Time | SSTYPE(3)
  /METHOD=REML
  /PRINT=CPS CORB COVB DESCRIPTIVES G  SOLUTION TESTCOV
  /EMMEANS=TABLES(OVERALL)
  /EMMEANS=TABLES(Condition) COMPARE ADJ(BONFERRONI)
  /EMMEANS=TABLES(Time) COMPARE ADJ(BONFERRONI)
  /EMMEANS=TABLES(Condition*Time) .
```

Remarques

Sortie obtenue		05-MAY-2021 12:09:54
Commentaires		
Entrée	Jeu de données actif	Jeu_de_données1
	Filtre	<sans>
	Pondération	<sans>
	Fichier scindé	<sans>
	N de lignes dans le fichier de travail	1048530
Gestion des valeurs manquantes	Définition de la valeur manquante	Les valeurs manquantes définies par l'utilisateur sont traitées comme étant manquantes.
	Observations utilisées	Les statistiques sont basées sur toutes les observations comportant des données valides pour toutes les variables du modèle.

Syntaxe		MIXED LaserP2Amplitude BY Condition Time<br>/CRITERIA=CIN(95) MXITER(100) MXSTEP(10) SCORING(1) SINGULAR(0.000000000001) HCONVERGE(0, ABSOLUTE) LCONVERGE(0, ABSOLUTE) PCONVERGE(0.000001, ABSOLUTE)<br>/FIXED=Condition Time Condition*Time | SSTYPE(3)<br>/METHOD=REML<br>/PRINT=CPS CORB COVB DESCRIPTIVES G SOLUTION TESTCOV<br>/EMMEANS=TABLES(OVERALL)<br>/EMMEANS=TABLES(Condition) COMPARE ADJ(BONFERRONI)<br>/EMMEANS=TABLES(Time) COMPARE ADJ(BONFERRONI)<br>/EMMEANS=TABLES(Condition*Time) .
Ressources	Temps de processeur	00:00:00,58
	Temps écoulé	00:00:00,58

Récapitulatif de traitement des observations

		Effectif	Pourcentage marginal
Condition	Sham	44	50,0%
	taVNS	44	50,0%
Time	T0	44	50,0%

	T2	44	50,0%
Valide		88	100,0%
Exclues		1048442	
Total		1048530	

Statistiques descriptives

Laser P2 Amplitude

Condition	Time	Effectif	Moyenne	Ecart type	Coefficient de variation
Sham	T0	22	10,2950091	6,49648736	63,1%
	T2	22	9,7946273	5,80687470	59,3%
	Total	44	10,0448182	6,09452896	60,7%
taVNS	T0	22	10,5294886	6,49763040	61,7%
	T2	22	10,7248464	6,12175060	57,1%
	Total	44	10,6271675	6,23943846	58,7%
Total	T0	44	10,4122489	6,42216241	61,7%
	T2	44	10,2597368	5,91534561	57,7%
	Total	88	10,3359928	6,13885077	59,4%

Dimension du modèle[a]

		Nombre de niveaux	Nombre de paramètres
Effets fixes	Constante	1	1
	Condition	2	1
	Time	2	1
	Condition * Time	4	1
Résidu			1
Total		9	5

a. Variable dépendante : Laser P2 Amplitude.

Critères d'information[a]

Log de vraisemblance restreint -2	558,280
Critère d'information d'Akaike (AIC)	560,280
Critère de Hurvich et Tsai (AICC)	560,328
Critère de Bozdogan (CAIC)	563,711
Critère bayésien de Schwartz (BIC)	562,711

Les critères d'informations sont présentés en plus petit, disposant d'un meilleur format.[a]

a. Variable dépendante : Laser P2 Amplitude.

Effets fixes

Tests des effets fixes de type III[a]

Source	Ddl du numérateur	Ddl du dénominateur	F	Sig.
Constante	1	84	241,648	,000
Condition	1	84	,192	,663
Time	1	84	,013	,909
Condition * Time	1	84	,068	,794

a. Variable dépendante : Laser P2 Amplitude.

Estimations des effets fixes[a]

Paramètre	Estimation	Erreur standard	ddl	t	Sig.
Constante	10,724846	1,329812	84	8,065	,000
[Condition=Sham]	-,930219	1,880638	84	-,495	,622
[Condition=taVNS]	0[b]	0	.	.	.
[Time=T0]	-,195358	1,880638	84	-,104	,918
[Time=T2]	0[b]	0	.	.	.
[Condition=Sham] * [Time=T0]	,695740	2,659624	84	,262	,794
[Condition=Sham] * [Time=T2]	0[b]	0	.	.	.
[Condition=taVNS] * [Time=T0]	0[b]	0	.	.	.
[Condition=taVNS] * [Time=T2]	0[b]	0	.	.	.

Estimations des effets fixes[a]

Paramètre	Intervalle de confiance à 95 %	
	Borne inférieure	Borne supérieure
Constante	8,080370	13,369323
[Condition=Sham]	-4,670074	2,809636
[Condition=taVNS]	.	.
[Time=T0]	-3,935213	3,544497
[Time=T2]	.	.

[Condition=Sham] * [Time=T0]	-4,593214	5,984693
[Condition=Sham] * [Time=T2]	.	.
[Condition=taVNS] * [Time=T0]	.	.
[Condition=taVNS] * [Time=T2]	.	.

a. Variable dépendante : Laser P2 Amplitude.

b. Ce paramètre est défini sur 0, car il est redondant.

Matrice de corrélation pour les estimations des effets fixes[a]

Paramètre	Constante	[Condition=Sham]	[Condition=taVNS]	[Time=T0]	[Time=T2]
Constante	1	-,707	.[b]	-,707	.[b]
[Condition=Sham]	-,707	1	.[b]	,500	.[b]
[Condition=taVNS]	.[b]	.[b]	.[b]	.[b]	.[b]
[Time=T0]	-,707	,500	.[b]	1	.[b]
[Time=T2]	.[b]	.[b]	.[b]	.[b]	.[b]
[Condition=Sham] * [Time=T0]	,500	-,707	.[b]	-,707	.[b]
[Condition=Sham] * [Time=T2]	.[b]	.[b]	.[b]	.[b]	.[b]
[Condition=taVNS] * [Time=T0]	.[b]	.[b]	.[b]	.[b]	.[b]
[Condition=taVNS] * [Time=T2]	.[b]	.[b]	.[b]	.[b]	.[b]

Matrice de corrélation pour les estimations des effets fixes[a]

Paramètre	[Condition=Sham] * [Time=T0]	[Condition=Sham] * [Time=T2]	[Condition=taVNS] * [Time=T0]	[Condition=taVNS] * [Time=T2]
Constante	,500	.[b]	.[b]	.[b]
[Condition=Sham]	-,707	.[b]	.[b]	.[b]
[Condition=taVNS]	.[b]	.[b]	.[b]	.[b]
[Time=T0]	-,707	.[b]	.[b]	.[b]
[Time=T2]	.[b]	.[b]	.[b]	.[b]
[Condition=Sham] * [Time=T0]	1	.[b]	.[b]	.[b]
[Condition=Sham] * [Time=T2]	.[b]	.[b]	.[b]	.[b]
[Condition=taVNS] * [Time=T0]	.[b]	.[b]	.[b]	.[b]
[Condition=taVNS] * [Time=T2]	.[b]	.[b]	.[b]	.[b]

a. Variable dépendante : Laser P2 Amplitude.

b. La corrélation est manquante par défaut, car elle est associée à un paramètre redondant.

Matrice de covariance pour les estimations des effets fixes[a]

Paramètre	Constante	[Condition=Sham]	[Condition=taVNS]	[Time=T0]	[Time=T2]
Constante	1,768400	-1,768400	0[b]	-1,768400	0[b]
[Condition=Sham]	-1,768400	3,536799	0[b]	1,768400	0[b]
[Condition=taVNS]	0[b]	0[b]	0[b]	0[b]	0[b]
[Time=T0]	-1,768400	1,768400	0[b]	3,536799	0[b]
[Time=T2]	0[b]	0[b]	0[b]	0[b]	0[b]
[Condition=Sham] * [Time=T0]	1,768400	-3,536799	0[b]	-3,536799	0[b]
[Condition=Sham] * [Time=T2]	0[b]	0[b]	0[b]	0[b]	0[b]
[Condition=taVNS] * [Time=T0]	0[b]	0[b]	0[b]	0[b]	0[b]
[Condition=taVNS] * [Time=T2]	0[b]	0[b]	0[b]	0[b]	0[b]

Matrice de covariance pour les estimations des effets fixes[a]

Paramètre	[Condition=Sham] * [Time=T0]	[Condition=Sham] * [Time=T2]	[Condition=taVNS] * [Time=T0]	[Condition=taVNS] * [Time=T2]
Constante	1,768400	0[b]	0[b]	0[b]
[Condition=Sham]	-3,536799	0[b]	0[b]	0[b]
[Condition=taVNS]	0[b]	0[b]	0[b]	0[b]
[Time=T0]	-3,536799	0[b]	0[b]	0[b]
[Time=T2]	0[b]	0[b]	0[b]	0[b]
[Condition=Sham] * [Time=T0]	7,073599	0[b]	0[b]	0[b]
[Condition=Sham] * [Time=T2]	0[b]	0[b]	0[b]	0[b]
[Condition=taVNS] * [Time=T0]	0[b]	0[b]	0[b]	0[b]
[Condition=taVNS] * [Time=T2]	0[b]	0[b]	0[b]	0[b]

a. Variable dépendante : Laser P2 Amplitude.

b. La covariance est définie sur 0, car elle est associée à un paramètre redondant.

Paramètres de covariance

Estimations des paramètres de covariance[a]

Paramètre	Estimation	Erreur standard	Z de Wald	Sig.	Intervalle de confiance à 95 %	
					Borne inférieure	Borne supérieure
Résidu	38,904793	6,003140	6,481	,000	28,751457	52,643695

a. Variable dépendante : Laser P2 Amplitude.

Matrice de corrélation pour les estimations des paramètres de covariance[a]

Paramètre	Résidu
Résidu	1

a. Variable dépendante : Laser P2 Amplitude.

Matrice de covariance pour les estimations des paramètres de covariance[a]

Paramètre	Résidu
Résidu	36,037689

a. Variable dépendante : Laser P2 Amplitude.

Moyenne marginale estimée

1. Grand Mean[a]

Moyenne	Erreur standard	ddl	Intervalle de confiance à 95 %	
			Borne inférieure	Borne supérieure
10,336	,665	84	9,014	11,658

a. Variable dépendante : Laser P2 Amplitude.

2. Condition

Estimations[a]

Condition	Moyenne	Erreur standard	ddl	Intervalle de confiance à 95 %	
				Borne inférieure	Borne supérieure
Sham	10,045	,940	84	8,175	11,915
taVNS	10,627	,940	84	8,757	12,497

a. Variable dépendante : Laser P2 Amplitude.

Comparaisons appariées[a]

(I) Condition	(J) Condition	Différence	Erreur standard	ddl	Sig.[b]

		moyenne (I-J)			
Sham	taVNS	-,582	1,330	84	,663
taVNS	Sham	,582	1,330	84	,663

Comparaisons appariées[a]

		Intervalle de confiance à 95 % pour la différence[b]	
(I) Condition	(J) Condition	Borne inférieure	Borne supérieure
Sham	taVNS	-3,227	2,062
taVNS	Sham	-2,062	3,227

Basées sur les moyennes marginales estimées[a]

a. Variable dépendante : Laser P2 Amplitude.

b. Ajustement pour les comparaisons multiples : Bonferroni.

Tests univariés[a]

Ddl du numérateur	Ddl du dénominateur	F	Sig.
1	84	,192	,663

Le test de F permet de tester l'effet de Condition. Il s'appuie sur les comparaisons appariées (indépendantes) linéaires parmi les moyennes marginales estimées.[a]

a. Variable dépendante : Laser P2 Amplitude.

3. Time

Estimations[a]

				Intervalle de confiance à 95 %	
Time	Moyenne	Erreur standard	ddl	Borne inférieure	Borne supérieure
T0	10,412	,940	84	8,542	12,282
T2	10,260	,940	84	8,390	12,130

a. Variable dépendante : Laser P2 Amplitude.

Comparaisons appariées[a]

						Intervalle de confiance à 95 % pour la différence[b]
(I) Time	(J) Time	Différence moyenne (I-J)	Erreur standard	ddl	Sig.[b]	Borne inférieure
T0	T2	,153	1,330	84	,909	-2,492

T2	T0	-,153	1,330	84	,909	-2,797

Comparaisons appariées[a]

		Intervalle de confiance à 95 % pour la différence
(I) Time	(J) Time	Borne supérieure
T0	T2	2,797
T2	T0	2,492

Basées sur les moyennes marginales estimées[a]

a. Variable dépendante : Laser P2 Amplitude.

b. Ajustement pour les comparaisons multiples : Bonferroni.

Tests univariés[a]

Ddl du numérateur	Ddl du dénominateur	F	Sig.
1	84	,013	,909

Le test de F permet de tester l'effet de Time. Il s'appuie sur les comparaisons appariées (indépendantes) linéaires parmi les moyennes marginales estimées.[a]

a. Variable dépendante : Laser P2 Amplitude.

4. Condition * Time[a]

					Intervalle de confiance à 95 %	
Condition	Time	Moyenne	Erreur standard	ddl	Borne inférieure	Borne supérieure
Sham	T0	10,295	1,330	84	7,651	12,939
	T2	9,795	1,330	84	7,150	12,439
taVNS	T0	10,529	1,330	84	7,885	13,174
	T2	10,725	1,330	84	8,080	13,369

a. Variable dépendante : Laser P2 Amplitude.

1.1.5 LEPs P2 Latency.

```
MIXED LaserP2Latency BY Condition Time
  /CRITERIA=CIN(95) MXITER(100) MXSTEP(10) SCORING(1)
SINGULAR(0.000000000001) HCONVERGE(0,
    ABSOLUTE) LCONVERGE(0, ABSOLUTE) PCONVERGE(0.000001, ABSOLUTE)
  /FIXED=Condition Time Condition*Time | SSTYPE(3)
  /METHOD=REML
  /PRINT=CPS CORB COVB DESCRIPTIVES G  SOLUTION TESTCOV
  /EMMEANS=TABLES(OVERALL)
  /EMMEANS=TABLES(Condition) COMPARE ADJ(BONFERRONI)
  /EMMEANS=TABLES(Time) COMPARE ADJ(BONFERRONI)
  /EMMEANS=TABLES(Condition*Time) .
```

Remarques

Sortie obtenue		05-MAY-2021 12:04:12
Commentaires		
Entrée	Jeu de données actif	Jeu_de_données1
	Filtre	<sans>
	Pondération	<sans>
	Fichier scindé	<sans>
	N de lignes dans le fichier de travail	1048530
Gestion des valeurs manquantes	Définition de la valeur manquante	Les valeurs manquantes définies par l'utilisateur sont traitées comme étant manquantes.
	Observations utilisées	Les statistiques sont basées sur toutes les observations comportant des données valides pour toutes les variables du modèle.

Syntaxe		MIXED LaserP2Latency BY Condition Time<br>/CRITERIA=CIN(95) MXITER(100) MXSTEP(10) SCORING(1) SINGULAR(0.000000000001) HCONVERGE(0, ABSOLUTE) LCONVERGE(0, ABSOLUTE) PCONVERGE(0.000001, ABSOLUTE)<br>/FIXED=Condition Time Condition*Time | SSTYPE(3)<br>/METHOD=REML<br>/PRINT=CPS CORB COVB DESCRIPTIVES G SOLUTION TESTCOV<br>/EMMEANS=TABLES(OVERALL)<br>/EMMEANS=TABLES(Condition) COMPARE ADJ(BONFERRONI)<br>/EMMEANS=TABLES(Time) COMPARE ADJ(BONFERRONI)<br>/EMMEANS=TABLES(Condition*Time) .
Ressources	Temps de processeur	00:00:00,56
	Temps écoulé	00:00:00,56

Récapitulatif de traitement des observations

		Effectif	Pourcentage marginal
Condition	Sham	43	50,0%
	taVNS	43	50,0%
Time	T0	42	48,8%

	T2	44	51,2%
Valide		86	100,0%
Exclues		1048444	
Total		1048530	

Statistiques descriptives

Laser P2 Latency

Condition	Time	Effectif	Moyenne	Ecart type	Coefficient de variation
Sham	T0	21	,3247171	,04279053	13,2%
	T2	22	,3155973	,03262634	10,3%
	Total	43	,3200512	,03775493	11,8%
taVNS	T0	21	,3116219	,05016204	16,1%
	T2	22	,3109155	,04311428	13,9%
	Total	43	,3112605	,04612757	14,8%
Total	T0	42	,3181695	,04652452	14,6%
	T2	44	,3132564	,03785862	12,1%
	Total	86	,3156558	,04213358	13,3%

Dimension du modèle[a]

		Nombre de niveaux	Nombre de paramètres
Effets fixes	Constante	1	1
	Condition	2	1
	Time	2	1
	Condition * Time	4	1
Résidu			1
Total		9	5

a. Variable dépendante : Laser P2 Latency.

Critères d'information[a]

Log de vraisemblance restreint -2	-272,853
Critère d'information d'Akaike (AIC)	-270,853
Critère de Hurvich et Tsai (AICC)	-270,803
Critère de Bozdogan (CAIC)	-267,446
Critère bayésien de Schwartz (BIC)	-268,446

Les critères d'informations sont présentés en plus petit, disposant d'un meilleur format.[a]

a. Variable dépendante : Laser P2 Latency

.

Effets fixes

Tests des effets fixes de type III[a]

Source	Ddl du numérateur	Ddl du dénominateur	F	Sig.
Constante	1	82	4736,078	,000
Condition	1	82	,938	,336
Time	1	82	,287	,594
Condition * Time	1	82	,210	,648

a. Variable dépendante : Laser P2 Latency .

Estimations des effets fixes[a]

Paramètre	Estimation	Erreur standard	ddl	t	Sig.
Constante	,310915	,009068	82	34,288	,000
[Condition=Sham]	,004682	,012824	82	,365	,716
[Condition=taVNS]	0[b]	0	.	.	.
[Time=T0]	,000706	,012976	82	,054	,957
[Time=T2]	0[b]	0	.	.	.
[Condition=Sham] * [Time=T0]	,008413	,018350	82	,458	,648
[Condition=Sham] * [Time=T2]	0[b]	0	.	.	.
[Condition=taVNS] * [Time=T0]	0[b]	0	.	.	.
[Condition=taVNS] * [Time=T2]	0[b]	0	.	.	.

Estimations des effets fixes[a]

Paramètre	Intervalle de confiance à 95 % Borne inférieure	Borne supérieure
Constante	,292877	,328954
[Condition=Sham]	-,020829	,030193
[Condition=taVNS]	.	.
[Time=T0]	-,025106	,026519

[Time=T2]	.	.
[Condition=Sham] * [Time=T0]	-,028091	,044918
[Condition=Sham] * [Time=T2]	.	.
[Condition=taVNS] * [Time=T0]	.	.
[Condition=taVNS] * [Time=T2]	.	.

a. Variable dépendante : Laser P2 Latency .

b. Ce paramètre est défini sur 0, car il est redondant.

Matrice de corrélation pour les estimations des effets fixes[a]

Paramètre	Constante	[Condition=Sham]	[Condition=taVNS]	[Time=T0]	[Time=T2]
Constante	1	-,707	.[b]	-,699	.[b]
[Condition=Sham]	-,707	1	.[b]	,494	.[b]
[Condition=taVNS]	.[b]	.[b]	.[b]	.[b]	.[b]
[Time=T0]	-,699	,494	.[b]	1	.[b]
[Time=T2]	.[b]	.[b]	.[b]	.[b]	.[b]
[Condition=Sham] * [Time=T0]	,494	-,699	.[b]	-,707	.[b]
[Condition=Sham] * [Time=T2]	.[b]	.[b]	.[b]	.[b]	.[b]
[Condition=taVNS] * [Time=T0]	.[b]	.[b]	.[b]	.[b]	.[b]
[Condition=taVNS] * [Time=T2]	.[b]	.[b]	.[b]	.[b]	.[b]

Matrice de corrélation pour les estimations des effets fixes[a]

Paramètre	[Condition=Sham] * [Time=T0]	[Condition=Sham] * [Time=T2]	[Condition=taVNS] * [Time=T0]	[Condition=taVNS] * [Time=T2]
Constante	,494	.[b]	.[b]	.[b]
[Condition=Sham]	-,699	.[b]	.[b]	.[b]
[Condition=taVNS]	.[b]	.[b]	.[b]	.[b]
[Time=T0]	-,707	.[b]	.[b]	.[b]
[Time=T2]	.[b]	.[b]	.[b]	.[b]
[Condition=Sham] * [Time=T0]	1	.[b]	.[b]	.[b]
[Condition=Sham] * [Time=T2]	.[b]	.[b]	.[b]	.[b]
[Condition=taVNS] * [Time=T0]	.[b]	.[b]	.[b]	.[b]
[Condition=taVNS] * [Time=T2]	.[b]	.[b]	.[b]	.[b]

a. Variable dépendante : Laser P2 Latency .

b. La corrélation est manquante par défaut, car elle est associée à un paramètre redondant.

Matrice de covariance pour les estimations des effets fixes[a]

Paramètre	Constante	[Condition=Sham]	[Condition=taVNS]	[Time=T0]
Constante	8,222555E-5	-8,222555E-5	0[b]	-8,222555E-5
[Condition=Sham]	-8,222555E-5	,000164	0[b]	8,222555E-5
[Condition=taVNS]	0[b]	0[b]	0[b]	0[b]
[Time=T0]	-8,222555E-5	8,222555E-5	0[b]	,000168
[Time=T2]	0[b]	0[b]	0[b]	0[b]
[Condition=Sham] * [Time=T0]	8,222555E-5	-,000164	0[b]	-,000168
[Condition=Sham] * [Time=T2]	0[b]	0[b]	0[b]	0[b]
[Condition=taVNS] * [Time=T0]	0[b]	0[b]	0[b]	0[b]
[Condition=taVNS] * [Time=T2]	0[b]	0[b]	0[b]	0[b]

Matrice de covariance pour les estimations des effets fixes[a]

Paramètre	[Time=T2]	[Condition=Sham] * [Time=T0]	[Condition=Sham] * [Time=T2]	[Condition=taVNS] * [Time=T0]
Constante	0[b]	8,222555E-5	0[b]	0[b]
[Condition=Sham]	0[b]	-,000164	0[b]	0[b]
[Condition=taVNS]	0[b]	0[b]	0[b]	0[b]
[Time=T0]	0[b]	-,000168	0[b]	0[b]
[Time=T2]	0[b]	0[b]	0[b]	0[b]
[Condition=Sham] * [Time=T0]	0[b]	,000337	0[b]	0[b]
[Condition=Sham] * [Time=T2]	0[b]	0[b]	0[b]	0[b]
[Condition=taVNS] * [Time=T0]	0[b]	0[b]	0[b]	0[b]
[Condition=taVNS] * [Time=T2]	0[b]	0[b]	0[b]	0[b]

Matrice de covariance pour les estimations des effets fixes[a]

Paramètre	[Condition=taVNS] * [Time=T2]
Constante	0[b]
[Condition=Sham]	0[b]
[Condition=taVNS]	0[b]
[Time=T0]	0[b]
[Time=T2]	0[b]
[Condition=Sham] * [Time=T0]	0[b]
[Condition=Sham] * [Time=T2]	0[b]
[Condition=taVNS] * [Time=T0]	0[b]
[Condition=taVNS] * [Time=T2]	0[b]

a. Variable dépendante : Laser P2 Latency .

b. La covariance est définie sur 0, car elle est associée à un paramètre redondant.

Paramètres de covariance

Estimations des paramètres de covariance[a]

Paramètre	Estimation	Erreur standard	Z de Wald	Sig.	Intervalle de confiance à 95 %	
					Borne inférieure	Borne supérieure
Résidu	,001809	,000283	6,403	,000	,001332	,002457

a. Variable dépendante : Laser P2 Latency .

Matrice de corrélation pour les estimations des paramètres de covariance[a]

Paramètre	Résidu
Résidu	1

a. Variable dépendante : Laser P2 Latency .

Matrice de covariance pour les estimations des paramètres de covariance[a]

Paramètre	Résidu
Résidu	7,981327E-8

a. Variable dépendante : Laser P2 Latency .

Moyenne marginale estimée

1. Grand Mean[a]

Moyenne	Erreur standard	ddl	Intervalle de confiance à 95 %	
			Borne inférieure	Borne supérieure
,316	,005	82	,307	,325

a. Variable dépendante : Laser P2 Latency .

2. Condition

Estimations[a]

Condition	Moyenne	Erreur standard	ddl	Intervalle de confiance à 95 % Borne inférieure	Borne supérieure
Sham	,320	,006	82	,307	,333
taVNS	,311	,006	82	,298	,324

a. Variable dépendante : Laser P2 Latency .

Comparaisons appariées[a]

(I) Condition	(J) Condition	Différence moyenne (I-J)	Erreur standard	ddl	Sig.[b]
Sham	taVNS	,009	,009	82	,336
taVNS	Sham	-,009	,009	82	,336

Comparaisons appariées[a]

(I) Condition	(J) Condition	Intervalle de confiance à 95 % pour la différence[b] Borne inférieure	Borne supérieure
Sham	taVNS	-,009	,027
taVNS	Sham	-,027	,009

Basées sur les moyennes marginales estimées[a]

a. Variable dépendante : Laser P2 Latency .

b. Ajustement pour les comparaisons multiples : Bonferroni.

Tests univariés[a]

Ddl du numérateur	Ddl du dénominateur	F	Sig.
1	82	,938	,336

Le test de F permet de tester l'effet de Condition. Il s'appuie sur les comparaisons appariées (indépendantes) linéaires parmi les moyennes marginales estimées.[a]

a. Variable dépendante : Laser P2 Latency .

3. Time

Estimations[a]

Time	Moyenne	Erreur standard	ddl	Intervalle de confiance à 95 % Borne inférieure	Borne supérieure
T0	,318	,007	82	,305	,331
T2	,313	,006	82	,301	,326

a. Variable dépendante : Laser P2 Latency .

Comparaisons appariées[a]

(I) Time	(J) Time	Différence moyenne (I-J)	Erreur standard	ddl	Sig.[b]	Intervalle de confiance à 95 % pour la différence[b] Borne inférieure
T0	T2	,005	,009	82	,594	-,013
T2	T0	-,005	,009	82	,594	-,023

Comparaisons appariées[a]

(I) Time	(J) Time	Intervalle de confiance à 95 % pour la différence Borne supérieure
T0	T2	,023
T2	T0	,013

Basées sur les moyennes marginales estimées[a]

a. Variable dépendante : Laser P2 Latency .

b. Ajustement pour les comparaisons multiples : Bonferroni.

Tests univariés[a]

Ddl du numérateur	Ddl du dénominateur	F	Sig.
1	82	,287	,594

Le test de F permet de tester l'effet de Time. Il s'appuie sur les comparaisons appariées (indépendantes) linéaires parmi les moyennes marginales estimées.[a]

a. Variable dépendante : Laser P2 Latency .

4. Condition * Time[a]

Condition	Time	Moyenne	Erreur standard	ddl	Intervalle de confiance à 95 % Borne inférieure	Intervalle de confiance à 95 % Borne supérieure
Sham	T0	,325	,009	82	,306	,343
	T2	,316	,009	82	,298	,334
taVNS	T0	,312	,009	82	,293	,330
	T2	,311	,009	82	,293	,329

a. Variable dépendante : Laser P2 Latency .

1.2. Vibrotactile evoked potentials.

1.2.1 Vibrotactile N2P2 Amplitude.

```
MIXED VibrotactileN2P2Amplitude BY Condition Time
  /CRITERIA=CIN(95) MXITER(100) MXSTEP(10) SCORING(1)
SINGULAR(0.000000000001) HCONVERGE(0,
    ABSOLUTE) LCONVERGE(0, ABSOLUTE) PCONVERGE(0.000001, ABSOLUTE)
  /FIXED=Condition Time Condition*Time | SSTYPE(3)
  /METHOD=REML
  /PRINT=CPS CORB COVB DESCRIPTIVES G  SOLUTION TESTCOV
  /EMMEANS=TABLES(OVERALL)
  /EMMEANS=TABLES(Condition) COMPARE ADJ(BONFERRONI)
  /EMMEANS=TABLES(Time) COMPARE ADJ(BONFERRONI)
  /EMMEANS=TABLES(Condition*Time) .
```

Remarques

Sortie obtenue		05-MAY-2021 12:14:22
Commentaires		
Entrée	Jeu de données actif	Jeu_de_données1
	Filtre	<sans>
	Pondération	<sans>
	Fichier scindé	<sans>
	N de lignes dans le fichier de travail	1048530
Gestion des valeurs manquantes	Définition de la valeur manquante	Les valeurs manquantes définies par l'utilisateur sont traitées comme étant manquantes.
	Observations utilisées	Les statistiques sont basées sur toutes les observations comportant des données valides pour toutes les variables du modèle.

Syntaxe		MIXED VibrotactileN2P2Amplitude BY Condition Time /CRITERIA=CIN(95) MXITER(100) MXSTEP(10) SCORING(1) SINGULAR(0.000000000001) HCONVERGE(0, ABSOLUTE) LCONVERGE(0, ABSOLUTE) PCONVERGE(0.000001, ABSOLUTE) /FIXED=Condition Time Condition*Time | SSTYPE(3) /METHOD=REML /PRINT=CPS CORB COVB DESCRIPTIVES G SOLUTION TESTCOV /EMMEANS=TABLES(OVERALL) /EMMEANS=TABLES(Condition) COMPARE ADJ(BONFERRONI) /EMMEANS=TABLES(Time) COMPARE ADJ(BONFERRONI) /EMMEANS=TABLES(Condition*Time) .
Ressources	Temps de processeur	00:00:00,56
	Temps écoulé	00:00:00,56

Récapitulatif de traitement des observations

		Effectif	Pourcentage marginal
Condition	Sham	43	50,0%
	taVNS	43	50,0%

Time	T0	42	48,8%
	T2	44	51,2%
Valide		86	100,0%
Exclues		1048444	
Total		1048530	

Statistiques descriptives

Vibrotactile N2P2 Amplitude

Condition	Time	Effectif	Moyenne	Ecart type	Coefficient de variation
Sham	T0	21	11,223580119047620	5,411122931097371	48,2%
	T2	22	9,861865136363638	4,338065262456726	44,0%
	Total	43	10,526888732558138	4,881263105566324	46,4%
taVNS	T0	21	11,190152380952382	4,420526241001070	39,5%
	T2	22	10,658235499999998	4,045728873453462	38,0%
	Total	43	10,918008860465116	4,190656619192797	38,4%
Total	T0	42	11,206866250000003	4,880118464426052	43,5%
	T2	44	10,260050318181818	4,164907778180733	40,6%
	Total	86	10,722448796511630	4,526521742902212	42,2%

Dimension du modèle[a]

		Nombre de niveaux	Nombre de paramètres
Effets fixes	Constante	1	1
	Condition	2	1
	Time	2	1
	Condition * Time	4	1
Résidu			1
Total		9	5

a. Variable dépendante : Vibrotactile N2P2 Amplitude.

Critères d'information[a]

Log de vraisemblance restreint -2	494,310
Critère d'information d'Akaike (AIC)	496,310
Critère de Hurvich et Tsai (AICC)	496,360
Critère de Bozdogan (CAIC)	499,717
Critère bayésien de Schwartz (BIC)	498,717

Les critères d'informations sont présentés en plus petit, disposant d'un meilleur format.[a]

a. Variable dépendante : Vibrotactile N2P2 Amplitude.

Effets fixes

Tests des effets fixes de type III[a]

Source	Ddl du numérateur	Ddl du dénominateur	F	Sig.
Constante	1	82	473,374	,000
Condition	1	82	,149	,700
Time	1	82	,921	,340
Condition * Time	1	82	,177	,675

a. Variable dépendante : Vibrotactile N2P2 Amplitude.

Estimations des effets fixes[a]

Paramètre	Estimation	Erreur standard	ddl	t	Sig.
Constante	10,658236	,975119	82	10,930	,000
[Condition=Sham]	-,796370	1,379027	82	-,577	,565
[Condition=taVNS]	0[b]	0	.	.	.
[Time=T0]	,531917	1,395347	82	,381	,704
[Time=T2]	0[b]	0	.	.	.
[Condition=Sham] * [Time=T0]	,829798	1,973319	82	,421	,675
[Condition=Sham] * [Time=T2]	0[b]	0	.	.	.

[Condition=taVNS] * [Time=T0]	0[b]	0	.	.	.
[Condition=taVNS] * [Time=T2]	0[b]	0	.	.	.

Estimations des effets fixes[a]

	Intervalle de confiance à 95 %	
Paramètre	Borne inférieure	Borne supérieure
Constante	8,718412	12,598059
[Condition=Sham]	-3,539694	1,946954
[Condition=taVNS]	.	.
[Time=T0]	-2,243874	3,307707
[Time=T2]	.	.
[Condition=Sham] * [Time=T0]	-3,095763	4,755359
[Condition=Sham] * [Time=T2]	.	.
[Condition=taVNS] * [Time=T0]	.	.
[Condition=taVNS] * [Time=T2]	.	.

a. Variable dépendante : Vibrotactile N2P2 Amplitude.

b. Ce paramètre est défini sur 0, car il est redondant.

Matrice de corrélation pour les estimations des effets fixes[a]

Paramètre	Constante	[Condition=Sham]	[Condition=taVNS]	[Time=T0]	[Time=T2]
Constante	1	-,707	.[b]	-,699	.[b]
[Condition=Sham]	-,707	1	.[b]	,494	.[b]
[Condition=taVNS]	.[b]	.[b]	.[b]	.[b]	.[b]
[Time=T0]	-,699	,494	.[b]	1	.[b]
[Time=T2]	.[b]	.[b]	.[b]	.[b]	.[b]
[Condition=Sham] * [Time=T0]	,494	-,699	.[b]	-,707	.[b]
[Condition=Sham] * [Time=T2]	.[b]	.[b]	.[b]	.[b]	.[b]
[Condition=taVNS] * [Time=T0]	.[b]	.[b]	.[b]	.[b]	.[b]
[Condition=taVNS] * [Time=T2]	.[b]	.[b]	.[b]	.[b]	.[b]

Matrice de corrélation pour les estimations des effets fixes[a]

Paramètre	[Condition=Sham] * [Time=T0]	[Condition=Sham] * [Time=T2]	[Condition=taVNS] * [Time=T0]	[Condition=taVNS] * [Time=T2]
Constante	,494	.[b]	.[b]	.[b]

[Condition=Sham]	-,699	.[b]	.[b]	.[b]
[Condition=taVNS]	.[b]	.[b]	.[b]	.[b]
[Time=T0]	-,707	.[b]	.[b]	.[b]
[Time=T2]	.[b]	.[b]	.[b]	.[b]
[Condition=Sham] * [Time=T0]	1	.[b]	.[b]	.[b]
[Condition=Sham] * [Time=T2]	.[b]	.[b]	.[b]	.[b]
[Condition=taVNS] * [Time=T0]	.[b]	.[b]	.[b]	.[b]
[Condition=taVNS] * [Time=T2]	.[b]	.[b]	.[b]	.[b]

a. Variable dépendante : Vibrotactile N2P2 Amplitude.

b. La corrélation est manquante par défaut, car elle est associée à un paramètre redondant.

Matrice de covariance pour les estimations des effets fixes[a]

Paramètre	Constante	[Condition=Sham]	[Condition=taVNS]	[Time=T0]	[Time=T2]
Constante	,950858	-,950858	0[b]	-,950858	0[b]
[Condition=Sham]	-,950858	1,901716	0[b]	,950858	0[b]
[Condition=taVNS]	0[b]	0[b]	0[b]	0[b]	0[b]
[Time=T0]	-,950858	,950858	0[b]	1,946995	0[b]
[Time=T2]	0[b]	0[b]	0[b]	0[b]	0[b]
[Condition=Sham] * [Time=T0]	,950858	-1,901716	0[b]	-1,946995	0[b]
[Condition=Sham] * [Time=T2]	0[b]	0[b]	0[b]	0[b]	0[b]
[Condition=taVNS] * [Time=T0]	0[b]	0[b]	0[b]	0[b]	0[b]
[Condition=taVNS] * [Time=T2]	0[b]	0[b]	0[b]	0[b]	0[b]

Matrice de covariance pour les estimations des effets fixes[a]

Paramètre	[Condition=Sham] * [Time=T0]	[Condition=Sham] * [Time=T2]	[Condition=taVNS] * [Time=T0]	[Condition=taVNS] * [Time=T2]
Constante	,950858	0[b]	0[b]	0[b]
[Condition=Sham]	-1,901716	0[b]	0[b]	0[b]
[Condition=taVNS]	0[b]	0[b]	0[b]	0[b]
[Time=T0]	-1,946995	0[b]	0[b]	0[b]
[Time=T2]	0[b]	0[b]	0[b]	0[b]
[Condition=Sham] * [Time=T0]	3,893989	0[b]	0[b]	0[b]
[Condition=Sham] * [Time=T2]	0[b]	0[b]	0[b]	0[b]
[Condition=taVNS] * [Time=T0]	0[b]	0[b]	0[b]	0[b]
[Condition=taVNS] * [Time=T2]	0[b]	0[b]	0[b]	0[b]

a. Variable dépendante : Vibrotactile N2P2 Amplitude.

b. La covariance est définie sur 0, car elle est associée à un paramètre redondant.

Paramètres de covariance

Estimations des paramètres de covariance[a]

Paramètre	Estimation	Erreur standard	Z de Wald	Sig.	Intervalle de confiance à 95 % Borne inférieure	Intervalle de confiance à 95 % Borne supérieure
Résidu	20,918871	3,266979	6,403	,000	15,402914	28,410155

a. Variable dépendante : Vibrotactile N2P2 Amplitude.

Matrice de corrélation pour les estimations des paramètres de covariance[a]

Paramètre	Résidu
Résidu	1

a. Variable dépendante : Vibrotactile N2P2 Amplitude.

Matrice de covariance pour les estimations des paramètres de covariance[a]

Paramètre	Résidu
Résidu	10,673151

a. Variable dépendante : Vibrotactile N2P2 Amplitude.

Moyenne marginale estimée

1. Grand Mean[a]

Moyenne	Erreur standard	ddl	Intervalle de confiance à 95 % Borne inférieure	Intervalle de confiance à 95 % Borne supérieure
10,733	,493	82	9,752	11,715

a. Variable dépendante : Vibrotactile N2P2 Amplitude.

2. Condition

Estimations[a]

Condition	Moyenne	Erreur standard	ddl	Intervalle de confiance à 95 % Borne inférieure	Borne supérieure
Sham	10,543	,698	82	9,155	11,931
taVNS	10,924	,698	82	9,536	12,312

a. Variable dépendante : Vibrotactile N2P2 Amplitude.

Comparaisons appariées[a]

(I) Condition	(J) Condition	Différence moyenne (I-J)	Erreur standard	ddl	Sig.[b]
Sham	taVNS	-,381	,987	82	,700
taVNS	Sham	,381	,987	82	,700

Comparaisons appariées[a]

(I) Condition	(J) Condition	Intervalle de confiance à 95 % pour la différence[b] Borne inférieure	Borne supérieure
Sham	taVNS	-2,344	1,581
taVNS	Sham	-1,581	2,344

Basées sur les moyennes marginales estimées[a]

a. Variable dépendante : Vibrotactile N2P2 Amplitude.

b. Ajustement pour les comparaisons multiples : Bonferroni.

Tests univariés[a]

Ddl du numérateur	Ddl du dénominateur	F	Sig.
1	82	,149	,700

Le test de F permet de tester l'effet de Condition. Il s'appuie sur les comparaisons appariées (indépendantes) linéaires parmi les moyennes marginales estimées.[a]

a. Variable dépendante : Vibrotactile N2P2 Amplitude.

3. Time

Estimations[a]

Time	Moyenne	Erreur standard	ddl	Intervalle de confiance à 95 % Borne inférieure	Borne supérieure
T0	11,207	,706	82	9,803	12,611
T2	10,260	,690	82	8,888	11,632

a. Variable dépendante : Vibrotactile N2P2 Amplitude.

Comparaisons appariées[a]

(I) Time	(J) Time	Différence moyenne (I-J)	Erreur standard	ddl	Sig.[b]	Intervalle de confiance à 95 % pour la différence[b] Borne inférieure
T0	T2	,947	,987	82	,340	-1,016
T2	T0	-,947	,987	82	,340	-2,910

Comparaisons appariées[a]

(I) Time	(J) Time	Intervalle de confiance à 95 % pour la différence Borne supérieure
T0	T2	2,910
T2	T0	1,016

Basées sur les moyennes marginales estimées[a]

a. Variable dépendante : Vibrotactile N2P2 Amplitude.

b. Ajustement pour les comparaisons multiples : Bonferroni.

Tests univariés[a]

Ddl du numérateur	Ddl du dénominateur	F	Sig.
1	82	,921	,340

Le test de F permet de tester l'effet de Time. Il s'appuie sur les comparaisons appariées (indépendantes) linéaires parmi les moyennes marginales estimées.[a]

a. Variable dépendante : Vibrotactile N2P2 Amplitude.

4. Condition * Time[a]

Condition	Time	Moyenne	Erreur standard	ddl	Intervalle de confiance à 95 % Borne inférieure	Intervalle de confiance à 95 % Borne supérieure
Sham	T0	11,224	,998	82	9,238	13,209
	T2	9,862	,975	82	7,922	11,802
taVNS	T0	11,190	,998	82	9,205	13,176
	T2	10,658	,975	82	8,718	12,598

a. Variable dépendante : Vibrotactile N2P2 Amplitude.

1.2.2. Vibrotactile N2 Amplitude.

```
MIXED VibrotactileN2Amplitude BY Condition Time
  /CRITERIA=CIN(95) MXITER(100) MXSTEP(10) SCORING(1)
SINGULAR(0.000000000001) HCONVERGE(0,
    ABSOLUTE) LCONVERGE(0, ABSOLUTE) PCONVERGE(0.000001, ABSOLUTE)
  /FIXED=Condition Time Condition*Time | SSTYPE(3)
  /METHOD=REML
  /PRINT=CPS CORB COVB DESCRIPTIVES G  SOLUTION TESTCOV
  /EMMEANS=TABLES(OVERALL)
  /EMMEANS=TABLES(Condition) COMPARE ADJ(BONFERRONI)
  /EMMEANS=TABLES(Time) COMPARE ADJ(BONFERRONI)
  /EMMEANS=TABLES(Condition*Time) .
```

Remarques

Sortie obtenue		05-MAY-2021 12:15:06
Commentaires		
Entrée	Jeu de données actif	Jeu_de_données1
	Filtre	<sans>
	Pondération	<sans>
	Fichier scindé	<sans>
	N de lignes dans le fichier de travail	1048530
Gestion des valeurs manquantes	Définition de la valeur manquante	Les valeurs manquantes définies par l'utilisateur sont traitées comme étant manquantes.
	Observations utilisées	Les statistiques sont basées sur toutes les observations comportant des données valides pour toutes les variables du modèle.

Syntaxe		MIXED VibrotactileN2Amplitude BY Condition Time /CRITERIA=CIN(95) MXITER(100) MXSTEP(10) SCORING(1) SINGULAR(0.000000000001) HCONVERGE(0, ABSOLUTE) LCONVERGE(0, ABSOLUTE) PCONVERGE(0.000001, ABSOLUTE) /FIXED=Condition Time Condition*Time | SSTYPE(3) /METHOD=REML /PRINT=CPS CORB COVB DESCRIPTIVES G SOLUTION TESTCOV /EMMEANS=TABLES(OVERALL) /EMMEANS=TABLES(Condition) COMPARE ADJ(BONFERRONI) /EMMEANS=TABLES(Time) COMPARE ADJ(BONFERRONI) /EMMEANS=TABLES(Condition*Time) .
Ressources	Temps de processeur	00:00:00,56
	Temps écoulé	00:00:00,57

Récapitulatif de traitement des observations

		Effectif	Pourcentage marginal
Condition	Sham	43	50,0%
	taVNS	43	50,0%

Time	T0	42	48,8%
	T2	44	51,2%
Valide		86	100,0%
Exclues		1048444	
Total		1048530	

Statistiques descriptives

Vibrotactile N2 Amplitude

Condition	Time	Effectif	Moyenne	Ecart type	Coefficient de variation
Sham	T0	21	-3,206082976190476	3,351057846095544	-104,5%
	T2	22	-3,968582272727273	3,273854799021414	-82,5%
	Total	43	-3,596198895348837	3,294725688764521	-91,6%
taVNS	T0	21	-3,458267142857143	2,472741136296201	-71,5%
	T2	22	-3,485719090909091	3,353580231415672	-96,2%
	Total	43	-3,472312325581395	2,921486816519839	-84,1%
Total	T0	42	-3,332175059523809	2,911493268973220	-87,4%
	T2	44	-3,727150681818182	3,284289110230492	-88,1%
	Total	86	-3,534255610465117	3,095960723199332	-87,6%

Dimension du modèle[a]

		Nombre de niveaux	Nombre de paramètres
Effets fixes	Constante	1	1
	Condition	2	1
	Time	2	1
	Condition * Time	4	1
Résidu			1
Total		9	5

a. Variable dépendante : Vibrotactile N2 Amplitude.

Critères d'information[a]

Log de vraisemblance restreint -2	432,594
Critère d'information d'Akaike (AIC)	434,594
Critère de Hurvich et Tsai (AICC)	434,644
Critère de Bozdogan (CAIC)	438,001
Critère bayésien de Schwartz (BIC)	437,001

Les critères d'informations sont présentés en plus petit, disposant d'un meilleur format.[a]

a. Variable dépendante : Vibrotactile N2 Amplitude.

Effets fixes

Tests des effets fixes de type III[a]

Source	Ddl du numérateur	Ddl du dénominateur	F	Sig.
Constante	1	82	108,657	,000
Condition	1	82	,029	,865
Time	1	82	,340	,561
Condition * Time	1	82	,295	,589

a. Variable dépendante : Vibrotactile N2 Amplitude.

Estimations des effets fixes[a]

Paramètre	Estimation	Erreur standard	ddl	t	Sig.
Constante	-3,485719	,669306	82	-5,208	,000
[Condition=Sham]	-,482863	,946541	82	-,510	,611
[Condition=taVNS]	0[b]	0	.	.	.
[Time=T0]	,027452	,957743	82	,029	,977
[Time=T2]	0[b]	0	.	.	.
[Condition=Sham] * [Time=T0]	,735047	1,354453	82	,543	,589
[Condition=Sham] * [Time=T2]	0[b]	0	.	.	.

[Condition=taVNS] * [Time=T0]	0[b]	0	.	.	.
[Condition=taVNS] * [Time=T2]	0[b]	0	.	.	.

Estimations des effets fixes[a]

Paramètre	Intervalle de confiance à 95 % Borne inférieure	Borne supérieure
Constante	-4,817181	-2,154257
[Condition=Sham]	-2,365835	1,400109
[Condition=taVNS]	.	.
[Time=T0]	-1,877804	1,932708
[Time=T2]	.	.
[Condition=Sham] * [Time=T0]	-1,959392	3,429486
[Condition=Sham] * [Time=T2]	.	.
[Condition=taVNS] * [Time=T0]	.	.
[Condition=taVNS] * [Time=T2]	.	.

a. Variable dépendante : Vibrotactile N2 Amplitude.

b. Ce paramètre est défini sur 0, car il est redondant.

Matrice de corrélation pour les estimations des effets fixes[a]

Paramètre	Constante	[Condition=Sham]	[Condition=taVNS]	[Time=T0]	[Time=T2]
Constante	1	-,707	.[b]	-,699	.[b]
[Condition=Sham]	-,707	1	.[b]	,494	.[b]
[Condition=taVNS]	.[b]	.[b]	.[b]	.[b]	.[b]
[Time=T0]	-,699	,494	.[b]	1	.[b]
[Time=T2]	.[b]	.[b]	.[b]	.[b]	.[b]
[Condition=Sham] * [Time=T0]	,494	-,699	.[b]	-,707	.[b]
[Condition=Sham] * [Time=T2]	.[b]	.[b]	.[b]	.[b]	.[b]
[Condition=taVNS] * [Time=T0]	.[b]	.[b]	.[b]	.[b]	.[b]
[Condition=taVNS] * [Time=T2]	.[b]	.[b]	.[b]	.[b]	.[b]

Matrice de corrélation pour les estimations des effets fixes[a]

Paramètre	[Condition=Sham] * [Time=T0]	[Condition=Sham] * [Time=T2]	[Condition=taVNS] * [Time=T0]	[Condition=taVNS] * [Time=T2]
Constante	,494	.[b]	.[b]	.[b]

[Condition=Sham]	-,699	.[b]	.[b]	.[b]
[Condition=taVNS]	.[b]	.[b]	.[b]	.[b]
[Time=T0]	-,707	.[b]	.[b]	.[b]
[Time=T2]	.[b]	.[b]	.[b]	.[b]
[Condition=Sham] * [Time=T0]	1	.[b]	.[b]	.[b]
[Condition=Sham] * [Time=T2]	.[b]	.[b]	.[b]	.[b]
[Condition=taVNS] * [Time=T0]	.[b]	.[b]	.[b]	.[b]
[Condition=taVNS] * [Time=T2]	.[b]	.[b]	.[b]	.[b]

a. Variable dépendante : Vibrotactile N2 Amplitude.

b. La corrélation est manquante par défaut, car elle est associée à un paramètre redondant.

Matrice de covariance pour les estimations des effets fixes[a]

Paramètre	Constante	[Condition=Sham]	[Condition=taVNS]	[Time=T0]	[Time=T2]
Constante	,447970	-,447970	0[b]	-,447970	0[b]
[Condition=Sham]	-,447970	,895940	0[b]	,447970	0[b]
[Condition=taVNS]	0[b]	0[b]	0[b]	0[b]	0[b]
[Time=T0]	-,447970	,447970	0[b]	,917272	0[b]
[Time=T2]	0[b]	0[b]	0[b]	0[b]	0[b]
[Condition=Sham] * [Time=T0]	,447970	-,895940	0[b]	-,917272	0[b]
[Condition=Sham] * [Time=T2]	0[b]	0[b]	0[b]	0[b]	0[b]
[Condition=taVNS] * [Time=T0]	0[b]	0[b]	0[b]	0[b]	0[b]
[Condition=taVNS] * [Time=T2]	0[b]	0[b]	0[b]	0[b]	0[b]

Matrice de covariance pour les estimations des effets fixes[a]

Paramètre	[Condition=Sham] * [Time=T0]	[Condition=Sham] * [Time=T2]	[Condition=taVNS] * [Time=T0]	[Condition=taVNS] * [Time=T2]
Constante	,447970	0[b]	0[b]	0[b]
[Condition=Sham]	-,895940	0[b]	0[b]	0[b]
[Condition=taVNS]	0[b]	0[b]	0[b]	0[b]
[Time=T0]	-,917272	0[b]	0[b]	0[b]
[Time=T2]	0[b]	0[b]	0[b]	0[b]
[Condition=Sham] * [Time=T0]	1,834544	0[b]	0[b]	0[b]
[Condition=Sham] * [Time=T2]	0[b]	0[b]	0[b]	0[b]
[Condition=taVNS] * [Time=T0]	0[b]	0[b]	0[b]	0[b]
[Condition=taVNS] * [Time=T2]	0[b]	0[b]	0[b]	0[b]

a. Variable dépendante : Vibrotactile N2 Amplitude.

b. La covariance est définie sur 0, car elle est associée à un paramètre redondant.

Paramètres de covariance

Estimations des paramètres de covariance[a]

Paramètre	Estimation	Erreur standard	Z de Wald	Sig.	Intervalle de confiance à 95 % Borne inférieure	Intervalle de confiance à 95 % Borne supérieure
Résidu	9,855340	1,539146	6,403	,000	7,256652	13,384648

a. Variable dépendante : Vibrotactile N2 Amplitude.

Matrice de corrélation pour les estimations des paramètres de covariance[a]

Paramètre	Résidu
Résidu	1

a. Variable dépendante : Vibrotactile N2 Amplitude.

Matrice de covariance pour les estimations des paramètres de covariance[a]

Paramètre	Résidu
Résidu	2,368969

a. Variable dépendante : Vibrotactile N2 Amplitude.

Moyenne marginale estimée

1. Grand Mean[a]

Moyenne	Erreur standard	ddl	Intervalle de confiance à 95 % Borne inférieure	Intervalle de confiance à 95 % Borne supérieure
-3,530	,339	82	-4,203	-2,856

a. Variable dépendante : Vibrotactile N2 Amplitude.

2. Condition

Estimations[a]

Condition	Moyenne	Erreur standard	ddl	Intervalle de confiance à 95 % Borne inférieure	Borne supérieure
Sham	-3,587	,479	82	-4,540	-2,635
taVNS	-3,472	,479	82	-4,425	-2,519

a. Variable dépendante : Vibrotactile N2 Amplitude.

Comparaisons appariées[a]

(I) Condition	(J) Condition	Différence moyenne (I-J)	Erreur standard	ddl	Sig.[b]
Sham	taVNS	-,115	,677	82	,865
taVNS	Sham	,115	,677	82	,865

Comparaisons appariées[a]

(I) Condition	(J) Condition	Intervalle de confiance à 95 % pour la différence[b] Borne inférieure	Borne supérieure
Sham	taVNS	-1,463	1,232
taVNS	Sham	-1,232	1,463

Basées sur les moyennes marginales estimées[a]

a. Variable dépendante : Vibrotactile N2 Amplitude.

b. Ajustement pour les comparaisons multiples : Bonferroni.

Tests univariés[a]

Ddl du numérateur	Ddl du dénominateur	F	Sig.
1	82	,029	,865

Le test de F permet de tester l'effet de Condition. Il s'appuie sur les comparaisons appariées (indépendantes) linéaires parmi les moyennes marginales estimées.[a]

a. Variable dépendante : Vibrotactile N2 Amplitude.

3. Time

Estimations[a]

Time	Moyenne	Erreur standard	ddl	Intervalle de confiance à 95 % Borne inférieure	Borne supérieure
T0	-3,332	,484	82	-4,296	-2,369
T2	-3,727	,473	82	-4,669	-2,786

a. Variable dépendante : Vibrotactile N2 Amplitude.

Comparaisons appariées[a]

(I) Time	(J) Time	Différence moyenne (I-J)	Erreur standard	ddl	Sig.[b]	Intervalle de confiance à 95 % pour la différence[b] Borne inférieure
T0	T2	,395	,677	82	,561	-,952
T2	T0	-,395	,677	82	,561	-1,742

Comparaisons appariées[a]

(I) Time	(J) Time	Intervalle de confiance à 95 % pour la différence Borne supérieure
T0	T2	1,742
T2	T0	,952

Basées sur les moyennes marginales estimées[a]

a. Variable dépendante : Vibrotactile N2 Amplitude.

b. Ajustement pour les comparaisons multiples : Bonferroni.

Tests univariés[a]

Ddl du numérateur	Ddl du dénominateur	F	Sig.
1	82	,340	,561

Le test de F permet de tester l'effet de Time. Il s'appuie sur les comparaisons appariées (indépendantes) linéaires parmi les moyennes marginales estimées.[a]

a. Variable dépendante : Vibrotactile N2 Amplitude.

4. Condition * Time[a]

Condition	Time	Moyenne	Erreur standard	ddl	Intervalle de confiance à 95 % Borne inférieure	Borne supérieure
Sham	T0	-3,206	,685	82	-4,569	-1,843
	T2	-3,969	,669	82	-5,300	-2,637
taVNS	T0	-3,458	,685	82	-4,821	-2,095
	T2	-3,486	,669	82	-4,817	-2,154

a. Variable dépendante : Vibrotactile N2 Amplitude.

1.2.3. Vibrotactile N2 Latency.

```
MIXED VibrotactileN2Latency BY Condition Time
  /CRITERIA=CIN(95) MXITER(100) MXSTEP(10) SCORING(1)
SINGULAR(0.000000000001) HCONVERGE(0,
    ABSOLUTE) LCONVERGE(0, ABSOLUTE) PCONVERGE(0.000001, ABSOLUTE)
  /FIXED=Condition Time Condition*Time | SSTYPE(3)
  /METHOD=REML
  /PRINT=CPS CORB COVB DESCRIPTIVES G  SOLUTION TESTCOV
  /EMMEANS=TABLES(OVERALL)
  /EMMEANS=TABLES(Condition) COMPARE ADJ(BONFERRONI)
  /EMMEANS=TABLES(Time) COMPARE ADJ(BONFERRONI)
  /EMMEANS=TABLES(Condition*Time) .
```

Remarques

Sortie obtenue		05-MAY-2021 12:16:39
Commentaires		
Entrée	Jeu de données actif	Jeu_de_données1
	Filtre	<sans>
	Pondération	<sans>
	Fichier scindé	<sans>
	N de lignes dans le fichier de travail	1048530
Gestion des valeurs manquantes	Définition de la valeur manquante	Les valeurs manquantes définies par l'utilisateur sont traitées comme étant manquantes.
	Observations utilisées	Les statistiques sont basées sur toutes les observations comportant des données valides pour toutes les variables du modèle.

Syntaxe		MIXED VibrotactileN2Latency BY Condition Time<br>/CRITERIA=CIN(95) MXITER(100) MXSTEP(10) SCORING(1) SINGULAR(0.000000000001) HCONVERGE(0, ABSOLUTE) LCONVERGE(0, ABSOLUTE) PCONVERGE(0.000001, ABSOLUTE)<br>/FIXED=Condition Time Condition*Time | SSTYPE(3)<br>/METHOD=REML<br>/PRINT=CPS CORB COVB DESCRIPTIVES G SOLUTION TESTCOV<br>/EMMEANS=TABLES(OVERALL)<br>/EMMEANS=TABLES(Condition) COMPARE ADJ(BONFERRONI)<br>/EMMEANS=TABLES(Time) COMPARE ADJ(BONFERRONI)<br>/EMMEANS=TABLES(Condition*Time) .
Ressources	Temps de processeur	00:00:00,56
	Temps écoulé	00:00:00,56

Récapitulatif de traitement des observations

		Effectif	Pourcentage marginal
Condition	Sham	42	50,0%
	taVNS	42	50,0%

Time	T0	40	47,6%
	T2	44	52,4%
Valide		84	100,0%
Exclues		1048446	
Total		1048530	

Statistiques descriptives

Vibrotactile N2 Latency

Condition	Time	Effectif	Moyenne	Ecart type	Coefficient de variation
Sham	T0	20	,16499490	,044914029	27,2%
	T2	22	,17158950	,031340821	18,3%
	Total	42	,16844921	,038066346	22,6%
taVNS	T0	20	,16619500	,042201485	25,4%
	T2	22	,17813545	,058347337	32,8%
	Total	42	,17244952	,051043890	29,6%
Total	T0	40	,16559495	,043020828	26,0%
	T2	44	,17486248	,046403483	26,5%
	Total	84	,17044937	,044798288	26,3%

Dimension du modèle[a]

		Nombre de niveaux	Nombre de paramètres
Effets fixes	Constante	1	1
	Condition	2	1
	Time	2	1
	Condition * Time	4	1
Résidu			1
Total		9	5

a. Variable dépendante : Vibrotactile N2 Latency .

Critères d'information[a]

Log de vraisemblance restreint -2	-255,850
Critère d'information d'Akaike (AIC)	-253,850
Critère de Hurvich et Tsai (AICC)	-253,799
Critère de Bozdogan (CAIC)	-250,468

Critère bayésien de Schwartz (BIC)	-251,468

Les critères d'informations sont présentés en plus petit, disposant d'un meilleur format.[a]

a. Variable dépendante : Vibrotactile N2 Latency .

Effets fixes

Tests des effets fixes de type III[a]

Source	Ddl du numérateur	Ddl du dénominateur	F	Sig.
Constante	1	80	1182,627	,000
Condition	1	80	,153	,697
Time	1	80	,876	,352
Condition * Time	1	80	,073	,788

a. Variable dépendante : Vibrotactile N2 Latency .

Estimations des effets fixes[a]

Paramètre	Estimation	Erreur standard	ddl	t	Sig.
Constante	,178135	,009661	80	18,438	,000
[Condition=Sham]	-,006546	,013663	80	-,479	,633
[Condition=taVNS]	0[b]	0	.	.	.
[Time=T0]	-,011940	,014001	80	-,853	,396
[Time=T2]	0[b]	0	.	.	.
[Condition=Sham] * [Time=T0]	,005346	,019800	80	,270	,788
[Condition=Sham] * [Time=T2]	0[b]	0	.	.	.
[Condition=taVNS] * [Time=T0]	0[b]	0	.	.	.
[Condition=taVNS] * [Time=T2]	0[b]	0	.	.	.

Estimations des effets fixes[a]

Paramètre	Intervalle de confiance à 95 %	
	Borne inférieure	Borne supérieure
Constante	,158908	,197362
[Condition=Sham]	-,033737	,020645
[Condition=taVNS]	.	.

[Time=T0]	-,039803	,015922
[Time=T2]	.	.
[Condition=Sham] * [Time=T0]	-,034058	,044749
[Condition=Sham] * [Time=T2]	.	.
[Condition=taVNS] * [Time=T0]	.	.
[Condition=taVNS] * [Time=T2]	.	.

a. Variable dépendante : Vibrotactile N2 Latency .

b. Ce paramètre est défini sur 0, car il est redondant.

Matrice de corrélation pour les estimations des effets fixes[a]

Paramètre	Constante	[Condition=Sham]	[Condition=taVNS]	[Time=T0]	[Time=T2]
Constante	1	-,707	.[b]	-,690	.[b]
[Condition=Sham]	-,707	1	.[b]	,488	.[b]
[Condition=taVNS]	.[b]	.[b]	.[b]	.[b]	.[b]
[Time=T0]	-,690	,488	.[b]	1	.[b]
[Time=T2]	.[b]	.[b]	.[b]	.[b]	.[b]
[Condition=Sham] * [Time=T0]	,488	-,690	.[b]	-,707	.[b]
[Condition=Sham] * [Time=T2]	.[b]	.[b]	.[b]	.[b]	.[b]
[Condition=taVNS] * [Time=T0]	.[b]	.[b]	.[b]	.[b]	.[b]
[Condition=taVNS] * [Time=T2]	.[b]	.[b]	.[b]	.[b]	.[b]

Matrice de corrélation pour les estimations des effets fixes[a]

Paramètre	[Condition=Sham] * [Time=T0]	[Condition=Sham] * [Time=T2]	[Condition=taVNS] * [Time=T0]	[Condition=taVNS] * [Time=T2]
Constante	,488	.[b]	.[b]	.[b]
[Condition=Sham]	-,690	.[b]	.[b]	.[b]
[Condition=taVNS]	.[b]	.[b]	.[b]	.[b]
[Time=T0]	-,707	.[b]	.[b]	.[b]
[Time=T2]	.[b]	.[b]	.[b]	.[b]
[Condition=Sham] * [Time=T0]	1	.[b]	.[b]	.[b]
[Condition=Sham] * [Time=T2]	.[b]	.[b]	.[b]	.[b]
[Condition=taVNS] * [Time=T0]	.[b]	.[b]	.[b]	.[b]
[Condition=taVNS] * [Time=T2]	.[b]	.[b]	.[b]	.[b]

a. Variable dépendante : Vibrotactile N2 Latency .

b. La corrélation est manquante par défaut, car elle est associée à un paramètre redondant.

Matrice de covariance pour les estimations des effets fixes[a]

Paramètre	Constante	[Condition=Sham]	[Condition=taVNS]	[Time=T0]
Constante	9,334449E-5	-9,334449E-5	0[b]	-9,334449E-5
[Condition=Sham]	-9,334449E-5	,000187	0[b]	9,334449E-5
[Condition=taVNS]	0[b]	0[b]	0[b]	0[b]
[Time=T0]	-9,334449E-5	9,334449E-5	0[b]	,000196
[Time=T2]	0[b]	0[b]	0[b]	0[b]
[Condition=Sham] * [Time=T0]	9,334449E-5	-,000187	0[b]	-,000196
[Condition=Sham] * [Time=T2]	0[b]	0[b]	0[b]	0[b]
[Condition=taVNS] * [Time=T0]	0[b]	0[b]	0[b]	0[b]
[Condition=taVNS] * [Time=T2]	0[b]	0[b]	0[b]	0[b]

Matrice de covariance pour les estimations des effets fixes[a]

Paramètre	[Time=T2]	[Condition=Sham] * [Time=T0]	[Condition=Sham] * [Time=T2]	[Condition=taVNS] * [Time=T0]
Constante	0[b]	9,334449E-5	0[b]	0[b]
[Condition=Sham]	0[b]	-,000187	0[b]	0[b]
[Condition=taVNS]	0[b]	0[b]	0[b]	0[b]
[Time=T0]	0[b]	-,000196	0[b]	0[b]
[Time=T2]	0[b]	0[b]	0[b]	0[b]
[Condition=Sham] * [Time=T0]	0[b]	,000392	0[b]	0[b]
[Condition=Sham] * [Time=T2]	0[b]	0[b]	0[b]	0[b]
[Condition=taVNS] * [Time=T0]	0[b]	0[b]	0[b]	0[b]
[Condition=taVNS] * [Time=T2]	0[b]	0[b]	0[b]	0[b]

Matrice de covariance pour les estimations des effets fixes[a]

Paramètre	[Condition=taVNS] * [Time=T2]
Constante	0[b]
[Condition=Sham]	0[b]
[Condition=taVNS]	0[b]
[Time=T0]	0[b]
[Time=T2]	0[b]
[Condition=Sham] * [Time=T0]	0[b]
[Condition=Sham] * [Time=T2]	0[b]
[Condition=taVNS] * [Time=T0]	0[b]
[Condition=taVNS] * [Time=T2]	0[b]

a. Variable dépendante : Vibrotactile N2 Latency .

b. La covariance est définie sur 0, car elle est associée à un paramètre redondant.

Paramètres de covariance

Estimations des paramètres de covariance[a]

Paramètre	Estimation	Erreur standard	Z de Wald	Sig.	Intervalle de confiance à 95 %	
					Borne inférieure	Borne supérieure
Résidu	,002054	,000325	6,325	,000	,001506	,002800

a. Variable dépendante : Vibrotactile N2 Latency .

Matrice de corrélation pour les estimations des paramètres de covariance[a]

Paramètre	Résidu
Résidu	1

a. Variable dépendante : Vibrotactile N2 Latency .

Matrice de covariance pour les estimations des paramètres de covariance[a]

Paramètre	Résidu
Résidu	1,054296E-7

a. Variable dépendante : Vibrotactile N2 Latency .

Moyenne marginale estimée

1. Grand Mean[a]

Moyenne	Erreur standard	ddl	Intervalle de confiance à 95 %	
			Borne inférieure	Borne supérieure
,170	,005	80	,160	,180

a. Variable dépendante : Vibrotactile N2 Latency .

2. Condition

Estimations[a]

Condition	Moyenne	Erreur standard	ddl	Intervalle de confiance à 95 % Borne inférieure	Borne supérieure
Sham	,168	,007	80	,154	,182
taVNS	,172	,007	80	,158	,186

a. Variable dépendante : Vibrotactile N2 Latency .

Comparaisons appariées[a]

(I) Condition	(J) Condition	Différence moyenne (I-J)	Erreur standard	ddl	Sig.[b]
Sham	taVNS	-,004	,010	80	,697
taVNS	Sham	,004	,010	80	,697

Comparaisons appariées[a]

(I) Condition	(J) Condition	Intervalle de confiance à 95 % pour la différence[b] Borne inférieure	Borne supérieure
Sham	taVNS	-,024	,016
taVNS	Sham	-,016	,024

Basées sur les moyennes marginales estimées[a]

a. Variable dépendante : Vibrotactile N2 Latency .

b. Ajustement pour les comparaisons multiples : Bonferroni.

Tests univariés[a]

Ddl du numérateur	Ddl du dénominateur	F	Sig.
1	80	,153	,697

Le test de F permet de tester l'effet de Condition. Il s'appuie sur les comparaisons appariées (indépendantes) linéaires parmi les moyennes marginales estimées.[a]

a. Variable dépendante : Vibrotactile N2 Latency .

3. Time

Estimations[a]

Time	Moyenne	Erreur standard	ddl	Intervalle de confiance à 95 % Borne inférieure	Borne supérieure
T0	,166	,007	80	,151	,180
T2	,175	,007	80	,161	,188

a. Variable dépendante : Vibrotactile N2 Latency .

Comparaisons appariées[a]

(I) Time	(J) Time	Différence moyenne (I-J)	Erreur standard	ddl	Sig.[b]	Intervalle de confiance à 95 % pour la différence[b] Borne inférieure
T0	T2	-,009	,010	80	,352	-,029
T2	T0	,009	,010	80	,352	-,010

Comparaisons appariées[a]

(I) Time	(J) Time	Intervalle de confiance à 95 % pour la différence Borne supérieure
T0	T2	,010
T2	T0	,029

Basées sur les moyennes marginales estimées[a]

a. Variable dépendante : Vibrotactile N2 Latency .

b. Ajustement pour les comparaisons multiples : Bonferroni.

Tests univariés[a]

Ddl du numérateur	Ddl du dénominateur	F	Sig.
1	80	,876	,352

Le test de F permet de tester l'effet de Time. Il s'appuie sur les comparaisons appariées (indépendantes) linéaires parmi les moyennes marginales estimées.[a]

a. Variable dépendante : Vibrotactile N2 Latency .

4. Condition * Time[a]

Condition	Time	Moyenne	Erreur standard	ddl	Intervalle de confiance à 95 % Borne inférieure	Intervalle de confiance à 95 % Borne supérieure
Sham	T0	,165	,010	80	,145	,185
	T2	,172	,010	80	,152	,191
taVNS	T0	,166	,010	80	,146	,186
	T2	,178	,010	80	,159	,197

a. Variable dépendante : Vibrotactile N2 Latency .

1.2.4. Vibrotactile P2 Amplitude

```
MIXED VibrotactileP2Amplitude BY Condition Time
  /CRITERIA=CIN(95) MXITER(100) MXSTEP(10) SCORING(1)
SINGULAR(0.000000000001) HCONVERGE(0,
    ABSOLUTE) LCONVERGE(0, ABSOLUTE) PCONVERGE(0.000001, ABSOLUTE)
  /FIXED=Condition Time Condition*Time | SSTYPE(3)
  /METHOD=REML
  /PRINT=CPS CORB COVB DESCRIPTIVES G  SOLUTION TESTCOV
  /EMMEANS=TABLES(OVERALL)
  /EMMEANS=TABLES(Condition) COMPARE ADJ(BONFERRONI)
  /EMMEANS=TABLES(Time) COMPARE ADJ(BONFERRONI)
  /EMMEANS=TABLES(Condition*Time) .
```

Remarques

Sortie obtenue		05-MAY-2021 12:17:17
Commentaires		
Entrée	Jeu de données actif	Jeu_de_données1
	Filtre	<sans>
	Pondération	<sans>
	Fichier scindé	<sans>
	N de lignes dans le fichier de travail	1048530
Gestion des valeurs manquantes	Définition de la valeur manquante	Les valeurs manquantes définies par l'utilisateur sont traitées comme étant manquantes.
	Observations utilisées	Les statistiques sont basées sur toutes les observations comportant des données valides pour toutes les variables du modèle.

Syntaxe		MIXED VibrotactileP2Amplitude BY Condition Time /CRITERIA=CIN(95) MXITER(100) MXSTEP(10) SCORING(1) SINGULAR(0.000000000001) HCONVERGE(0, ABSOLUTE) LCONVERGE(0, ABSOLUTE) PCONVERGE(0.000001, ABSOLUTE) /FIXED=Condition Time Condition*Time | SSTYPE(3) /METHOD=REML /PRINT=CPS CORB COVB DESCRIPTIVES G SOLUTION TESTCOV /EMMEANS=TABLES(OVERALL) /EMMEANS=TABLES(Condition) COMPARE ADJ(BONFERRONI) /EMMEANS=TABLES(Time) COMPARE ADJ(BONFERRONI) /EMMEANS=TABLES(Condition*Time) .
Ressources	Temps de processeur	00:00:00,58
	Temps écoulé	00:00:00,58

Récapitulatif de traitement des observations

		Effectif	Pourcentage marginal
Condition	Sham	43	50,0%
	taVNS	43	50,0%

Time	T0	42	48,8%
	T2	44	51,2%
Valide		86	100,0%
Exclues		1048444	
Total		1048530	

Statistiques descriptives

Vibrotactile P2 Amplitude

Condition	Time	Effectif	Moyenne	Ecart type	Coefficient de variation
Sham	T0	21	8,195904761904762	4,648777308225917	56,7%
	T2	22	6,238027409090909	5,127307612963029	82,2%
	Total	43	7,194200069767441	4,941281579702848	68,7%
taVNS	T0	21	7,675347619047619	3,044581801761787	39,7%
	T2	22	7,116061863636364	4,243118008219027	59,6%
	Total	43	7,389201418604652	3,673701304020610	49,7%
Total	T0	42	7,935626190476190	3,890129671928595	49,0%
	T2	44	6,677044636363636	4,672135110540143	70,0%
	Total	86	7,291700744186046	4,329292885469625	59,4%

Dimension du modèle[a]

		Nombre de niveaux	Nombre de paramètres
Effets fixes	Constante	1	1
	Condition	2	1
	Time	2	1
	Condition * Time	4	1
Résidu			1
Total		9	5

a. Variable dépendante : Vibrotactile P2 Amplitude.

Critères d'information[a]

Log de vraisemblance restreint -2	485,881
Critère d'information d'Akaike (AIC)	487,881
Critère de Hurvich et Tsai (AICC)	487,931
Critère de Bozdogan (CAIC)	491,288
Critère bayésien de Schwartz (BIC)	490,288

Les critères d'informations sont présentés en plus petit, disposant d'un meilleur format.[a]

a. Variable dépendante : Vibrotactile P2 Amplitude.

Effets fixes

Tests des effets fixes de type III[a]

Source	Ddl du numérateur	Ddl du dénominateur	F	Sig.
Constante	1	82	243,091	,000
Condition	1	82	,036	,849
Time	1	82	1,803	,183
Condition * Time	1	82	,557	,458

a. Variable dépendante : Vibrotactile P2 Amplitude.

Estimations des effets fixes[a]

Paramètre	Estimation	Erreur standard	ddl	t	Sig.
Constante	7,116062	,926265	82	7,683	,000
[Condition=Sham]	-,878034	1,309937	82	-,670	,505
[Condition=taVNS]	0[b]	0	.	.	.
[Time=T0]	,559286	1,325439	82	,422	,674
[Time=T2]	0[b]	0	.	.	.
[Condition=Sham] * [Time=T0]	1,398592	1,874454	82	,746	,458
[Condition=Sham] * [Time=T2]	0[b]	0	.	.	.
[Condition=taVNS] * [Time=T0]	0[b]	0	.	.	.

[Condition=taVNS] * [Time=T2]	0[b]	0	.	.	.

Estimations des effets fixes[a]

Paramètre	Intervalle de confiance à 95 % Borne inférieure	Borne supérieure
Constante	5,273426	8,958698
[Condition=Sham]	-3,483915	1,727847
[Condition=taVNS]	.	.
[Time=T0]	-2,077435	3,196007
[Time=T2]	.	.
[Condition=Sham] * [Time=T0]	-2,330295	5,127478
[Condition=Sham] * [Time=T2]	.	.
[Condition=taVNS] * [Time=T0]	.	.
[Condition=taVNS] * [Time=T2]	.	.

a. Variable dépendante : Vibrotactile P2 Amplitude.

b. Ce paramètre est défini sur 0, car il est redondant.

Matrice de corrélation pour les estimations des effets fixes[a]

Paramètre	Constante	[Condition=Sham]	[Condition=taVNS]	[Time=T0]	[Time=T2]
Constante	1	-,707	.[b]	-,699	.[b]
[Condition=Sham]	-,707	1	.[b]	,494	.[b]
[Condition=taVNS]	.[b]	.[b]	.[b]	.[b]	.[b]
[Time=T0]	-,699	,494	.[b]	1	.[b]
[Time=T2]	.[b]	.[b]	.[b]	.[b]	.[b]
[Condition=Sham] * [Time=T0]	,494	-,699	.[b]	-,707	.[b]
[Condition=Sham] * [Time=T2]	.[b]	.[b]	.[b]	.[b]	.[b]
[Condition=taVNS] * [Time=T0]	.[b]	.[b]	.[b]	.[b]	.[b]
[Condition=taVNS] * [Time=T2]	.[b]	.[b]	.[b]	.[b]	.[b]

Matrice de corrélation pour les estimations des effets fixes[a]

Paramètre	[Condition=Sham] * [Time=T0]	[Condition=Sham] * [Time=T2]	[Condition=taVNS] * [Time=T0]	[Condition=taVNS] * [Time=T2]
Constante	,494	.[b]	.[b]	.[b]
[Condition=Sham]	-,699	.[b]	.[b]	.[b]
[Condition=taVNS]	.[b]	.[b]	.[b]	.[b]

[Time=T0]	-,707	.[b]	.[b]	.[b]
[Time=T2]	.[b]	.[b]	.[b]	.[b]
[Condition=Sham] * [Time=T0]	1	.[b]	.[b]	.[b]
[Condition=Sham] * [Time=T2]	.[b]	.[b]	.[b]	.[b]
[Condition=taVNS] * [Time=T0]	.[b]	.[b]	.[b]	.[b]
[Condition=taVNS] * [Time=T2]	.[b]	.[b]	.[b]	.[b]

a. Variable dépendante : Vibrotactile P2 Amplitude.

b. La corrélation est manquante par défaut, car elle est associée à un paramètre redondant.

Matrice de covariance pour les estimations des effets fixes[a]

Paramètre	Constante	[Condition=Sham]	[Condition=taVNS]	[Time=T0]	[Time=T2]
Constante	,857967	-,857967	0[b]	-,857967	0[b]
[Condition=Sham]	-,857967	1,715934	0[b]	,857967	0[b]
[Condition=taVNS]	0[b]	0[b]	0[b]	0[b]	0[b]
[Time=T0]	-,857967	,857967	0[b]	1,756789	0[b]
[Time=T2]	0[b]	0[b]	0[b]	0[b]	0[b]
[Condition=Sham] * [Time=T0]	,857967	-1,715934	0[b]	-1,756789	0[b]
[Condition=Sham] * [Time=T2]	0[b]	0[b]	0[b]	0[b]	0[b]
[Condition=taVNS] * [Time=T0]	0[b]	0[b]	0[b]	0[b]	0[b]
[Condition=taVNS] * [Time=T2]	0[b]	0[b]	0[b]	0[b]	0[b]

Matrice de covariance pour les estimations des effets fixes[a]

Paramètre	[Condition=Sham] * [Time=T0]	[Condition=Sham] * [Time=T2]	[Condition=taVNS] * [Time=T0]	[Condition=taVNS] * [Time=T2]
Constante	,857967	0[b]	0[b]	0[b]
[Condition=Sham]	-1,715934	0[b]	0[b]	0[b]
[Condition=taVNS]	0[b]	0[b]	0[b]	0[b]
[Time=T0]	-1,756789	0[b]	0[b]	0[b]
[Time=T2]	0[b]	0[b]	0[b]	0[b]
[Condition=Sham] * [Time=T0]	3,513579	0[b]	0[b]	0[b]
[Condition=Sham] * [Time=T2]	0[b]	0[b]	0[b]	0[b]
[Condition=taVNS] * [Time=T0]	0[b]	0[b]	0[b]	0[b]
[Condition=taVNS] * [Time=T2]	0[b]	0[b]	0[b]	0[b]

a. Variable dépendante : Vibrotactile P2 Amplitude.

b. La covariance est définie sur 0, car elle est associée à un paramètre redondant.

Paramètres de covariance

Estimations des paramètres de covariance[a]

Paramètre	Estimation	Erreur standard	Z de Wald	Sig.	Intervalle de confiance à 95 %	
					Borne inférieure	Borne supérieure
Résidu	18,875271	2,947822	6,403	,000	13,898177	25,634717

a. Variable dépendante : Vibrotactile P2 Amplitude.

Matrice de corrélation pour les estimations des paramètres de covariance[a]

Paramètre	Résidu
Résidu	1

a. Variable dépendante : Vibrotactile P2 Amplitude.

Matrice de covariance pour les estimations des paramètres de covariance[a]

Paramètre	Résidu
Résidu	8,689655

a. Variable dépendante : Vibrotactile P2 Amplitude.

Moyenne marginale estimée

1. Grand Mean[a]

Moyenne	Erreur standard	ddl	Intervalle de confiance à 95 %	
			Borne inférieure	Borne supérieure
7,306	,469	82	6,374	8,239

a. Variable dépendante : Vibrotactile P2 Amplitude.

2. Condition

Estimations[a]

Condition	Moyenne	Erreur standard	ddl	Intervalle de confiance à 95 % Borne inférieure	Borne supérieure
Sham	7,217	,663	82	5,899	8,535
taVNS	7,396	,663	82	6,077	8,714

a. Variable dépendante : Vibrotactile P2 Amplitude.

Comparaisons appariées[a]

(I) Condition	(J) Condition	Différence moyenne (I-J)	Erreur standard	ddl	Sig.[b]
Sham	taVNS	-,179	,937	82	,849
taVNS	Sham	,179	,937	82	,849

Comparaisons appariées[a]

(I) Condition	(J) Condition	Intervalle de confiance à 95 % pour la différence[b] Borne inférieure	Borne supérieure
Sham	taVNS	-2,043	1,686
taVNS	Sham	-1,686	2,043

Basées sur les moyennes marginales estimées[a]

a. Variable dépendante : Vibrotactile P2 Amplitude.

b. Ajustement pour les comparaisons multiples : Bonferroni.

Tests univariés[a]

Ddl du numérateur	Ddl du dénominateur	F	Sig.
1	82	,036	,849

Le test de F permet de tester l'effet de Condition. Il s'appuie sur les comparaisons appariées (indépendantes) linéaires parmi les moyennes marginales estimées.[a]

a. Variable dépendante : Vibrotactile P2 Amplitude.

3. Time

Estimations[a]

Time	Moyenne	Erreur standard	ddl	Intervalle de confiance à 95 % Borne inférieure	Borne supérieure
T0	7,936	,670	82	6,602	9,269
T2	6,677	,655	82	5,374	7,980

a. Variable dépendante : Vibrotactile P2 Amplitude.

Comparaisons appariées[a]

(I) Time	(J) Time	Différence moyenne (I-J)	Erreur standard	ddl	Sig.[b]	Intervalle de confiance à 95 % pour la différence[b] Borne inférieure
T0	T2	1,259	,937	82	,183	-,606
T2	T0	-1,259	,937	82	,183	-3,123

Comparaisons appariées[a]

(I) Time	(J) Time	Intervalle de confiance à 95 % pour la différence Borne supérieure
T0	T2	3,123
T2	T0	,606

Basées sur les moyennes marginales estimées[a]

a. Variable dépendante : Vibrotactile P2 Amplitude.

b. Ajustement pour les comparaisons multiples : Bonferroni.

Tests univariés[a]

Ddl du numérateur	Ddl du dénominateur	F	Sig.
1	82	1,803	,183

Le test de F permet de tester l'effet de Time. Il s'appuie sur les comparaisons appariées (indépendantes) linéaires parmi les moyennes marginales estimées.[a]

a. Variable dépendante : Vibrotactile P2 Amplitude.

4. Condition * Time[a]

Condition	Time	Moyenne	Erreur standard	ddl	Intervalle de confiance à 95 % Borne inférieure	Intervalle de confiance à 95 % Borne supérieure
Sham	T0	8,196	,948	82	6,310	10,082
	T2	6,238	,926	82	4,395	8,081
taVNS	T0	7,675	,948	82	5,789	9,561
	T2	7,116	,926	82	5,273	8,959

a. Variable dépendante : Vibrotactile P2 Amplitude.

1.2.5. Vibrotactile P2 Latency.

```
MIXED VibrotactileP2Latency BY Condition Time
  /CRITERIA=CIN(95) MXITER(100) MXSTEP(10) SCORING(1)
SINGULAR(0.000000000001) HCONVERGE(0,
    ABSOLUTE) LCONVERGE(0, ABSOLUTE) PCONVERGE(0.000001, ABSOLUTE)
  /FIXED=Condition Time Condition*Time | SSTYPE(3)
  /METHOD=REML
  /PRINT=CPS CORB COVB DESCRIPTIVES G  SOLUTION TESTCOV
  /EMMEANS=TABLES(OVERALL)
  /EMMEANS=TABLES(Condition) COMPARE ADJ(BONFERRONI)
  /EMMEANS=TABLES(Time) COMPARE ADJ(BONFERRONI)
  /EMMEANS=TABLES(Condition*Time) .
```

Remarques

Sortie obtenue		05-MAY-2021 12:17:51
Commentaires		
Entrée	Jeu de données actif	Jeu_de_données1
	Filtre	<sans>
	Pondération	<sans>
	Fichier scindé	<sans>
	N de lignes dans le fichier de travail	1048530
Gestion des valeurs manquantes	Définition de la valeur manquante	Les valeurs manquantes définies par l'utilisateur sont traitées comme étant manquantes.
	Observations utilisées	Les statistiques sont basées sur toutes les observations comportant des données valides pour toutes les variables du modèle.

Syntaxe		MIXED VibrotactileP2Latency BY Condition Time<br>/CRITERIA=CIN(95) MXITER(100) MXSTEP(10) SCORING(1) SINGULAR(0.000000000001) HCONVERGE(0, ABSOLUTE) LCONVERGE(0, ABSOLUTE) PCONVERGE(0.000001, ABSOLUTE)<br>/FIXED=Condition Time Condition*Time | SSTYPE(3)<br>/METHOD=REML<br>/PRINT=CPS CORB COVB DESCRIPTIVES G SOLUTION TESTCOV<br>/EMMEANS=TABLES(OVERALL)<br>/EMMEANS=TABLES(Condition) COMPARE ADJ(BONFERRONI)<br>/EMMEANS=TABLES(Time) COMPARE ADJ(BONFERRONI)<br>/EMMEANS=TABLES(Condition*Time) .
Ressources	Temps de processeur	00:00:00,56
	Temps écoulé	00:00:00,57

Récapitulatif de traitement des observations

		Effectif	Pourcentage marginal
Condition	Sham	42	50,0%
	taVNS	42	50,0%
Time	T0	40	47,6%

	T2	44	52,4%
Valide		84	100,0%
Exclues		1048446	
Total		1048530	

Statistiques descriptives

Vibrotactile P2 Latency

Condition	Time	Effectif	Moyenne	Ecart type	Coefficient de variation
Sham	T0	20	,308701500000000	,087205293252851	28,2%
	T2	22	,299687272727273	,081201952485429	27,1%
	Total	42	,303979761904762	,083199826380216	27,4%
taVNS	T0	20	,333453500000000	,070829801880802	21,2%
	T2	22	,300097272727273	,086288835462887	28,8%
	Total	42	,315981190476191	,080142797561523	25,4%
Total	T0	40	,321077500000000	,079410887187124	24,7%
	T2	44	,299892272727273	,082804304681486	27,6%
	Total	84	,309980476190476	,081416152713464	26,3%

Dimension du modèle[a]

		Nombre de niveaux	Nombre de paramètres
Effets fixes	Constante	1	1
	Condition	2	1
	Time	2	1
	Condition * Time	4	1
Résidu			1
Total		9	5

a. Variable dépendante : Vibrotactile P2 Latency .

Critères d'information[a]

Log de vraisemblance restreint -2	-161,451
Critère d'information d'Akaike (AIC)	-159,451
Critère de Hurvich et Tsai (AICC)	-159,400
Critère de Bozdogan (CAIC)	-156,069
Critère bayésien de Schwartz (BIC)	-157,069

Les critères d'informations sont présentés en plus petit, disposant d'un meilleur format.[a]

a. Variable dépendante : Vibrotactile P2 Latency .

Effets fixes

Tests des effets fixes de type III[a]

Source	Ddl du numérateur	Ddl du dénominateur	F	Sig.
Constante	1	80	1208,933	,000
Condition	1	80	,496	,483
Time	1	80	1,407	,239
Condition * Time	1	80	,464	,498

a. Variable dépendante : Vibrotactile P2 Latency .

Estimations des effets fixes[a]

Paramètre	Estimation	Erreur standard	ddl	t	Sig.
Constante	,300097	,017429	80	17,218	,000
[Condition=Sham]	-,000410	,024648	80	-,017	,987
[Condition=taVNS]	0[b]	0	.	.	.
[Time=T0]	,033356	,025257	80	1,321	,190
[Time=T2]	0[b]	0	.	.	.
[Condition=Sham] * [Time=T0]	-,024342	,035719	80	-,681	,498
[Condition=Sham] * [Time=T2]	0[b]	0	.	.	.
[Condition=taVNS] * [Time=T0]	0[b]	0	.	.	.

[Condition=taVNS] * [Time=T2]	0[b]	0	.	.	.

Estimations des effets fixes[a]

Paramètre	Intervalle de confiance à 95 % Borne inférieure	Borne supérieure
Constante	,265412	,334782
[Condition=Sham]	-,049462	,048642
[Condition=taVNS]	.	.
[Time=T0]	-,016907	,083620
[Time=T2]	.	.
[Condition=Sham] * [Time=T0]	-,095425	,046741
[Condition=Sham] * [Time=T2]	.	.
[Condition=taVNS] * [Time=T0]	.	.
[Condition=taVNS] * [Time=T2]	.	.

a. Variable dépendante : Vibrotactile P2 Latency .

b. Ce paramètre est défini sur 0, car il est redondant.

Matrice de corrélation pour les estimations des effets fixes[a]

Paramètre	Constante	[Condition=Sham]	[Condition=taVNS]	[Time=T0]	[Time=T2]
Constante	1	-,707	.[b]	-,690	.[b]
[Condition=Sham]	-,707	1	.[b]	,488	.[b]
[Condition=taVNS]	.[b]	.[b]	.[b]	.[b]	.[b]
[Time=T0]	-,690	,488	.[b]	1	.[b]
[Time=T2]	.[b]	.[b]	.[b]	.[b]	.[b]
[Condition=Sham] * [Time=T0]	,488	-,690	.[b]	-,707	.[b]
[Condition=Sham] * [Time=T2]	.[b]	.[b]	.[b]	.[b]	.[b]
[Condition=taVNS] * [Time=T0]	.[b]	.[b]	.[b]	.[b]	.[b]
[Condition=taVNS] * [Time=T2]	.[b]	.[b]	.[b]	.[b]	.[b]

Matrice de corrélation pour les estimations des effets fixes[a]

Paramètre	[Condition=Sham] * [Time=T0]	[Condition=Sham] * [Time=T2]	[Condition=taVNS] * [Time=T0]	[Condition=taVNS] * [Time=T2]
Constante	,488	.[b]	.[b]	.[b]
[Condition=Sham]	-,690	.[b]	.[b]	.[b]
[Condition=taVNS]	.[b]	.[b]	.[b]	.[b]

[Time=T0]	-,707	.[b]	.[b]	.[b]
[Time=T2]	.[b]	.[b]	.[b]	.[b]
[Condition=Sham] * [Time=T0]	1	.[b]	.[b]	.[b]
[Condition=Sham] * [Time=T2]	.[b]	.[b]	.[b]	.[b]
[Condition=taVNS] * [Time=T0]	.[b]	.[b]	.[b]	.[b]
[Condition=taVNS] * [Time=T2]	.[b]	.[b]	.[b]	.[b]

a. Variable dépendante : Vibrotactile P2 Latency .

b. La corrélation est manquante par défaut, car elle est associée à un paramètre redondant.

Matrice de covariance pour les estimations des effets fixes[a]

Paramètre	Constante	[Condition=Sham]	[Condition=taVNS]	[Time=T0]	[Time=T2]
Constante	,000304	-,000304	0[b]	-,000304	0[b]
[Condition=Sham]	-,000304	,000608	0[b]	,000304	0[b]
[Condition=taVNS]	0[b]	0[b]	0[b]	0[b]	0[b]
[Time=T0]	-,000304	,000304	0[b]	,000638	0[b]
[Time=T2]	0[b]	0[b]	0[b]	0[b]	0[b]
[Condition=Sham] * [Time=T0]	,000304	-,000608	0[b]	-,000638	0[b]
[Condition=Sham] * [Time=T2]	0[b]	0[b]	0[b]	0[b]	0[b]
[Condition=taVNS] * [Time=T0]	0[b]	0[b]	0[b]	0[b]	0[b]
[Condition=taVNS] * [Time=T2]	0[b]	0[b]	0[b]	0[b]	0[b]

Matrice de covariance pour les estimations des effets fixes[a]

Paramètre	[Condition=Sham] * [Time=T0]	[Condition=Sham] * [Time=T2]	[Condition=taVNS] * [Time=T0]	[Condition=taVNS] * [Time=T2]
Constante	,000304	0[b]	0[b]	0[b]
[Condition=Sham]	-,000608	0[b]	0[b]	0[b]
[Condition=taVNS]	0[b]	0[b]	0[b]	0[b]
[Time=T0]	-,000638	0[b]	0[b]	0[b]
[Time=T2]	0[b]	0[b]	0[b]	0[b]
[Condition=Sham] * [Time=T0]	,001276	0[b]	0[b]	0[b]
[Condition=Sham] * [Time=T2]	0[b]	0[b]	0[b]	0[b]
[Condition=taVNS] * [Time=T0]	0[b]	0[b]	0[b]	0[b]
[Condition=taVNS] * [Time=T2]	0[b]	0[b]	0[b]	0[b]

a. Variable dépendante : Vibrotactile P2 Latency .

b. La covariance est définie sur 0, car elle est associée à un paramètre redondant.

Paramètres de covariance

Estimations des paramètres de covariance[a]

Paramètre	Estimation	Erreur standard	Z de Wald	Sig.	Intervalle de confiance à 95 % Borne inférieure	Borne supérieure
Résidu	,006683	,001057	6,325	,000	,004902	,009111

a. Variable dépendante : Vibrotactile P2 Latency .

Matrice de corrélation pour les estimations des paramètres de covariance[a]

Paramètre	Résidu
Résidu	1

a. Variable dépendante : Vibrotactile P2 Latency .

Matrice de covariance pour les estimations des paramètres de covariance[a]

Paramètre	Résidu
Résidu	1,116565E-6

a. Variable dépendante : Vibrotactile P2 Latency .

Moyenne marginale estimée

1. Grand Mean[a]

Moyenne	Erreur standard	ddl	Intervalle de confiance à 95 % Borne inférieure	Borne supérieure
,310	,009	80	,293	,328

a. Variable dépendante : Vibrotactile P2 Latency .

2. Condition

Estimations[a]

Condition	Moyenne	Erreur standard	ddl	Intervalle de confiance à 95 % Borne inférieure	Borne supérieure
Sham	,304	,013	80	,279	,329
taVNS	,317	,013	80	,292	,342

a. Variable dépendante : Vibrotactile P2 Latency .

Comparaisons appariées[a]

(I) Condition	(J) Condition	Différence moyenne (I-J)	Erreur standard	ddl	Sig.[b]
Sham	taVNS	-,013	,018	80	,483
taVNS	Sham	,013	,018	80	,483

Comparaisons appariées[a]

(I) Condition	(J) Condition	Intervalle de confiance à 95 % pour la différence[b] Borne inférieure	Borne supérieure
Sham	taVNS	-,048	,023
taVNS	Sham	-,023	,048

Basées sur les moyennes marginales estimées[a]

a. Variable dépendante : Vibrotactile P2 Latency .

b. Ajustement pour les comparaisons multiples : Bonferroni.

Tests univariés[a]

Ddl du numérateur	Ddl du dénominateur	F	Sig.
1	80	,496	,483

Le test de F permet de tester l'effet de Condition. Il s'appuie sur les comparaisons appariées (indépendantes) linéaires parmi les moyennes marginales estimées.[a]

a. Variable dépendante : Vibrotactile P2 Latency .

3. Time

Estimations[a]

Time	Moyenne	Erreur standard	ddl	Intervalle de confiance à 95 % Borne inférieure	Borne supérieure
T0	,321	,013	80	,295	,347
T2	,300	,012	80	,275	,324

a. Variable dépendante : Vibrotactile P2 Latency .

Comparaisons appariées[a]

(I) Time	(J) Time	Différence moyenne (I-J)	Erreur standard	ddl	Sig.[b]	Intervalle de confiance à 95 % pour la différence[b] Borne inférieure
T0	T2	,021	,018	80	,239	-,014
T2	T0	-,021	,018	80	,239	-,057

Comparaisons appariées[a]

(I) Time	(J) Time	Intervalle de confiance à 95 % pour la différence Borne supérieure
T0	T2	,057
T2	T0	,014

Basées sur les moyennes marginales estimées[a]

a. Variable dépendante : Vibrotactile P2 Latency .

b. Ajustement pour les comparaisons multiples : Bonferroni.

Tests univariés[a]

Ddl du numérateur	Ddl du dénominateur	F	Sig.
1	80	1,407	,239

Le test de F permet de tester l'effet de Time. Il s'appuie sur les comparaisons appariées (indépendantes) linéaires parmi les moyennes marginales estimées.[a]

a. Variable dépendante : Vibrotactile P2 Latency .

4. Condition * Time[a]

Condition	Time	Moyenne	Erreur standard	ddl	Intervalle de confiance à 95 % Borne inférieure	Intervalle de confiance à 95 % Borne supérieure
Sham	T0	,309	,018	80	,272	,345
	T2	,300	,017	80	,265	,334
taVNS	T0	,333	,018	80	,297	,370
	T2	,300	,017	80	,265	,335

a. Variable dépendante : Vibrotactile P2 Latency .

1.3 Cool-evoked potentials.

1.3.1 Cool N2P2 Amplitude.

```
MIXED CoolN2P2Amplitude BY Condition Time
  /CRITERIA=CIN(95) MXITER(100) MXSTEP(10) SCORING(1)
SINGULAR(0.000000000001) HCONVERGE(0,
    ABSOLUTE) LCONVERGE(0, ABSOLUTE) PCONVERGE(0.000001, ABSOLUTE)
  /FIXED=Condition Time Condition*Time | SSTYPE(3)
  /METHOD=REML
  /PRINT=CPS CORB COVB DESCRIPTIVES G  SOLUTION TESTCOV
  /EMMEANS=TABLES(OVERALL)
  /EMMEANS=TABLES(Condition) COMPARE ADJ(BONFERRONI)
  /EMMEANS=TABLES(Time) COMPARE ADJ(BONFERRONI)
  /EMMEANS=TABLES(Condition*Time) .
```

Remarques

Sortie obtenue		05-MAY-2021 12:19:03
Commentaires		
Entrée	Jeu de données actif	Jeu_de_données1
	Filtre	<sans>
	Pondération	<sans>
	Fichier scindé	<sans>
	N de lignes dans le fichier de travail	1048530
Gestion des valeurs manquantes	Définition de la valeur manquante	Les valeurs manquantes définies par l'utilisateur sont traitées comme étant manquantes.
	Observations utilisées	Les statistiques sont basées sur toutes les observations comportant des données valides pour toutes les variables du modèle.

Syntaxe		MIXED CoolN2P2Amplitude BY Condition Time<br>/CRITERIA=CIN(95) MXITER(100) MXSTEP(10) SCORING(1) SINGULAR(0.000000000001) HCONVERGE(0, ABSOLUTE) LCONVERGE(0, ABSOLUTE) PCONVERGE(0.000001, ABSOLUTE)<br>/FIXED=Condition Time Condition*Time | SSTYPE(3)<br>/METHOD=REML<br>/PRINT=CPS CORB COVB DESCRIPTIVES G SOLUTION TESTCOV<br>/EMMEANS=TABLES(OVERALL)<br>/EMMEANS=TABLES(Condition) COMPARE ADJ(BONFERRONI)<br>/EMMEANS=TABLES(Time) COMPARE ADJ(BONFERRONI)<br>/EMMEANS=TABLES(Condition*Time) .
Ressources	Temps de processeur	00:00:00,58
	Temps écoulé	00:00:00,57

Récapitulatif de traitement des observations

		Effectif	Pourcentage marginal
Condition	Sham	44	50,6%
	taVNS	43	49,4%
Time	T0	44	50,6%

	T2	43	49,4%
Valide		87	100,0%
Exclues		1048443	
Total		1048530	

Statistiques descriptives

Cool N2P2 Amplitude

Condition	Time	Effectif	Moyenne	Ecart type	Coefficient de variation
Sham	T0	22	10,753159090909090	4,304707184296333	40,0%
	T2	22	10,485426363636364	4,391612156648654	41,9%
	Total	44	10,619292727272727	4,299649730898512	40,5%
taVNS	T0	22	11,160510454545454	6,023261852915369	54,0%
	T2	21	11,311207619047620	5,132348441243936	45,4%
	Total	43	11,234106744186050	5,539763956891131	49,3%
Total	T0	44	10,956834772727273	5,177858090848929	47,3%
	T2	43	10,888714883720931	4,728731916694838	43,4%
	Total	87	10,923166321839085	4,932212775114727	45,2%

Dimension du modèle[a]

		Nombre de niveaux	Nombre de paramètres
Effets fixes	Constante	1	1
	Condition	2	1
	Time	2	1
	Condition * Time	4	1
Résidu			1
Total		9	5

a. Variable dépendante : Cool N2P2 Amplitude.

Critères d'information[a]

Log de vraisemblance restreint -2	515,341
Critère d'information d'Akaike (AIC)	517,341
Critère de Hurvich et Tsai (AICC)	517,391
Critère de Bozdogan (CAIC)	520,760
Critère bayésien de Schwartz (BIC)	519,760

Les critères d'informations sont présentés en plus petit, disposant d'un meilleur format.[a]

a. Variable dépendante : Cool N2P2 Amplitude.

Effets fixes

Tests des effets fixes de type III[a]

Source	Ddl du numérateur	Ddl du dénominateur	F	Sig.
Constante	1	83	413,820	,000
Condition	1	83	,329	,568
Time	1	83	,003	,957
Condition * Time	1	83	,038	,846

a. Variable dépendante : Cool N2P2 Amplitude.

Estimations des effets fixes[a]

Paramètre	Estimation	Erreur standard	ddl	t	Sig.
Constante	11,311208	1,093150	83	10,347	,000
[Condition=Sham]	-,825781	1,528279	83	-,540	,590
[Condition=taVNS]	0[b]	0	.	.	.
[Time=T0]	-,150697	1,528279	83	-,099	,922
[Time=T2]	0[b]	0	.	.	.
[Condition=Sham] * [Time=T0]	,418430	2,148711	83	,195	,846
[Condition=Sham] * [Time=T2]	0[b]	0	.	.	.
[Condition=taVNS] * [Time=T0]	0[b]	0	.	.	.

[Condition=taVNS] * [Time=T2]	0[b]	0	.	.	.

Estimations des effets fixes[a]

Paramètre	Intervalle de confiance à 95 % Borne inférieure	Borne supérieure
Constante	9,136976	13,485439
[Condition=Sham]	-3,865467	2,213905
[Condition=taVNS]	.	.
[Time=T0]	-3,190383	2,888989
[Time=T2]	.	.
[Condition=Sham] * [Time=T0]	-3,855269	4,692129
[Condition=Sham] * [Time=T2]	.	.
[Condition=taVNS] * [Time=T0]	.	.
[Condition=taVNS] * [Time=T2]	.	.

a. Variable dépendante : Cool N2P2 Amplitude.

b. Ce paramètre est défini sur 0, car il est redondant.

Matrice de corrélation pour les estimations des effets fixes[a]

Paramètre	Constante	[Condition=Sham]	[Condition=taVNS]	[Time=T0]	[Time=T2]
Constante	1	-,715	.[b]	-,715	.[b]
[Condition=Sham]	-,715	1	.[b]	,512	.[b]
[Condition=taVNS]	.[b]	.[b]	.[b]	.[b]	.[b]
[Time=T0]	-,715	,512	.[b]	1	.[b]
[Time=T2]	.[b]	.[b]	.[b]	.[b]	.[b]
[Condition=Sham] * [Time=T0]	,509	-,711	.[b]	-,711	.[b]
[Condition=Sham] * [Time=T2]	.[b]	.[b]	.[b]	.[b]	.[b]
[Condition=taVNS] * [Time=T0]	.[b]	.[b]	.[b]	.[b]	.[b]
[Condition=taVNS] * [Time=T2]	.[b]	.[b]	.[b]	.[b]	.[b]

Matrice de corrélation pour les estimations des effets fixes[a]

Paramètre	[Condition=Sham] * [Time=T0]	[Condition=Sham] * [Time=T2]	[Condition=taVNS] * [Time=T0]	[Condition=taVNS] * [Time=T2]
Constante	,509	.[b]	.[b]	.[b]
[Condition=Sham]	-,711	.[b]	.[b]	.[b]
[Condition=taVNS]	.[b]	.[b]	.[b]	.[b]

[Time=T0]	-,711	.[b]	.[b]	.[b]
[Time=T2]	.[b]	.[b]	.[b]	.[b]
[Condition=Sham] * [Time=T0]	1	.[b]	.[b]	.[b]
[Condition=Sham] * [Time=T2]	.[b]	.[b]	.[b]	.[b]
[Condition=taVNS] * [Time=T0]	.[b]	.[b]	.[b]	.[b]
[Condition=taVNS] * [Time=T2]	.[b]	.[b]	.[b]	.[b]

a. Variable dépendante : Cool N2P2 Amplitude.

b. La corrélation est manquante par défaut, car elle est associée à un paramètre redondant.

Matrice de covariance pour les estimations des effets fixes[a]

Paramètre	Constante	[Condition=Sham]	[Condition=taVNS]	[Time=T0]	[Time=T2]
Constante	1,194977	-1,194977	0[b]	-1,194977	0[b]
[Condition=Sham]	-1,194977	2,335637	0[b]	1,194977	0[b]
[Condition=taVNS]	0[b]	0[b]	0[b]	0[b]	0[b]
[Time=T0]	-1,194977	1,194977	0[b]	2,335637	0[b]
[Time=T2]	0[b]	0[b]	0[b]	0[b]	0[b]
[Condition=Sham] * [Time=T0]	1,194977	-2,335637	0[b]	-2,335637	0[b]
[Condition=Sham] * [Time=T2]	0[b]	0[b]	0[b]	0[b]	0[b]
[Condition=taVNS] * [Time=T0]	0[b]	0[b]	0[b]	0[b]	0[b]
[Condition=taVNS] * [Time=T2]	0[b]	0[b]	0[b]	0[b]	0[b]

Matrice de covariance pour les estimations des effets fixes[a]

Paramètre	[Condition=Sham] * [Time=T0]	[Condition=Sham] * [Time=T2]	[Condition=taVNS] * [Time=T0]	[Condition=taVNS] * [Time=T2]
Constante	1,194977	0[b]	0[b]	0[b]
[Condition=Sham]	-2,335637	0[b]	0[b]	0[b]
[Condition=taVNS]	0[b]	0[b]	0[b]	0[b]
[Time=T0]	-2,335637	0[b]	0[b]	0[b]
[Time=T2]	0[b]	0[b]	0[b]	0[b]
[Condition=Sham] * [Time=T0]	4,616958	0[b]	0[b]	0[b]
[Condition=Sham] * [Time=T2]	0[b]	0[b]	0[b]	0[b]
[Condition=taVNS] * [Time=T0]	0[b]	0[b]	0[b]	0[b]
[Condition=taVNS] * [Time=T2]	0[b]	0[b]	0[b]	0[b]

a. Variable dépendante : Cool N2P2 Amplitude.

b. La covariance est définie sur 0, car elle est associée à un paramètre redondant.

Paramètres de covariance

Estimations des paramètres de covariance[a]

Paramètre	Estimation	Erreur standard	Z de Wald	Sig.	Intervalle de confiance à 95 %	
					Borne inférieure	Borne supérieure
Résidu	25,094522	3,895425	6,442	,000	18,511722	34,018177

a. Variable dépendante : Cool N2P2 Amplitude.

Matrice de corrélation pour les estimations des paramètres de covariance[a]

Paramètre	Résidu
Résidu	1

a. Variable dépendante : Cool N2P2 Amplitude.

Matrice de covariance pour les estimations des paramètres de covariance[a]

Paramètre	Résidu
Résidu	15,174338

a. Variable dépendante : Cool N2P2 Amplitude.

Moyenne marginale estimée

1. Grand Mean[a]

Moyenne	Erreur standard	ddl	Intervalle de confiance à 95 %	
			Borne inférieure	Borne supérieure
10,928	,537	83	9,859	11,996

a. Variable dépendante : Cool N2P2 Amplitude.

2. Condition

Estimations[a]

Condition	Moyenne	Erreur standard	ddl	Intervalle de confiance à 95 % Borne inférieure	Borne supérieure
Sham	10,619	,755	83	9,117	12,121
taVNS	11,236	,764	83	9,716	12,756

a. Variable dépendante : Cool N2P2 Amplitude.

Comparaisons appariées[a]

(I) Condition	(J) Condition	Différence moyenne (I-J)	Erreur standard	ddl	Sig.[b]
Sham	taVNS	-,617	1,074	83	,568
taVNS	Sham	,617	1,074	83	,568

Comparaisons appariées[a]

(I) Condition	(J) Condition	Intervalle de confiance à 95 % pour la différence[b] Borne inférieure	Borne supérieure
Sham	taVNS	-2,753	1,520
taVNS	Sham	-1,520	2,753

Basées sur les moyennes marginales estimées[a]

a. Variable dépendante : Cool N2P2 Amplitude.

b. Ajustement pour les comparaisons multiples : Bonferroni.

Tests univariés[a]

Ddl du numérateur	Ddl du dénominateur	F	Sig.
1	83	,329	,568

Le test de F permet de tester l'effet de Condition. Il s'appuie sur les comparaisons appariées (indépendantes) linéaires parmi les moyennes marginales estimées.[a]

a. Variable dépendante : Cool N2P2 Amplitude.

3. Time

Estimations[a]

Time	Moyenne	Erreur standard	ddl	Intervalle de confiance à 95 % Borne inférieure	Borne supérieure
T0	10,957	,755	83	9,455	12,459
T2	10,898	,764	83	9,378	12,418

a. Variable dépendante : Cool N2P2 Amplitude.

Comparaisons appariées[a]

(I) Time	(J) Time	Différence moyenne (I-J)	Erreur standard	ddl	Sig.[b]	Intervalle de confiance à 95 % pour la différence[b] Borne inférieure
T0	T2	,059	1,074	83	,957	-2,078
T2	T0	-,059	1,074	83	,957	-2,195

Comparaisons appariées[a]

(I) Time	(J) Time	Intervalle de confiance à 95 % pour la différence Borne supérieure
T0	T2	2,195
T2	T0	2,078

Basées sur les moyennes marginales estimées[a]

a. Variable dépendante : Cool N2P2 Amplitude.

b. Ajustement pour les comparaisons multiples : Bonferroni.

Tests univariés[a]

Ddl du numérateur	Ddl du dénominateur	F	Sig.
1	83	,003	,957

Le test de F permet de tester l'effet de Time. Il s'appuie sur les comparaisons appariées (indépendantes) linéaires parmi les moyennes marginales estimées.[a]

a. Variable dépendante : Cool N2P2 Amplitude.

4. Condition * Time[a]

Condition	Time	Moyenne	Erreur standard	ddl	Intervalle de confiance à 95 % Borne inférieure	Intervalle de confiance à 95 % Borne supérieure
Sham	T0	10,753	1,068	83	8,629	12,877
	T2	10,485	1,068	83	8,361	12,610
taVNS	T0	11,161	1,068	83	9,036	13,285
	T2	11,311	1,093	83	9,137	13,485

a. Variable dépendante : Cool N2P2 Amplitude.

1.3.2. Cool N2 Amplitude.

```
MIXED CoolN2Amplitude_A BY Condition Time
  /CRITERIA=CIN(95) MXITER(100) MXSTEP(10) SCORING(1)
SINGULAR(0.000000000001) HCONVERGE(0,
    ABSOLUTE) LCONVERGE(0, ABSOLUTE) PCONVERGE(0.000001, ABSOLUTE)
  /FIXED=Condition Time Condition*Time | SSTYPE(3)
  /METHOD=REML
  /PRINT=CPS CORB COVB DESCRIPTIVES G  SOLUTION TESTCOV
  /EMMEANS=TABLES(OVERALL)
  /EMMEANS=TABLES(Condition) COMPARE ADJ(BONFERRONI)
  /EMMEANS=TABLES(Time) COMPARE ADJ(BONFERRONI)
  /EMMEANS=TABLES(Condition*Time) .
```

Remarques

Sortie obtenue		05-MAY-2021 12:19:46
Commentaires		
Entrée	Jeu de données actif	Jeu_de_données1
	Filtre	<sans>
	Pondération	<sans>
	Fichier scindé	<sans>
	N de lignes dans le fichier de travail	1048530
Gestion des valeurs manquantes	Définition de la valeur manquante	Les valeurs manquantes définies par l'utilisateur sont traitées comme étant manquantes.
	Observations utilisées	Les statistiques sont basées sur toutes les observations comportant des données valides pour toutes les variables du modèle.

Syntaxe		MIXED CoolN2Amplitude_A BY Condition Time<br>/CRITERIA=CIN(95) MXITER(100) MXSTEP(10) SCORING(1) SINGULAR(0.000000000001) HCONVERGE(0, ABSOLUTE) LCONVERGE(0, ABSOLUTE) PCONVERGE(0.000001, ABSOLUTE)<br>/FIXED=Condition Time Condition*Time | SSTYPE(3)<br>/METHOD=REML<br>/PRINT=CPS CORB COVB DESCRIPTIVES G SOLUTION TESTCOV<br>/EMMEANS=TABLES(OVERALL)<br>/EMMEANS=TABLES(Condition) COMPARE ADJ(BONFERRONI)<br>/EMMEANS=TABLES(Time) COMPARE ADJ(BONFERRONI)<br>/EMMEANS=TABLES(Condition*Time) .
Ressources	Temps de processeur	00:00:00,58
	Temps écoulé	00:00:00,58

Récapitulatif de traitement des observations

		Effectif	Pourcentage marginal
Condition	Sham	44	51,2%
	taVNS	42	48,8%
Time	T0	43	50,0%

	T2	43	50,0%
Valide		86	100,0%
Exclues		1048444	
Total		1048530	

Statistiques descriptives

Cool N2 Amplitude

Condition	Time	Effectif	Moyenne	Ecart type	Coefficient de variation
Sham	T0	22	-3,9429818	3,23276448	-82,0%
	T2	22	-5,0735432	3,39669916	-66,9%
	Total	44	-4,5082625	3,32647875	-73,8%
taVNS	T0	21	-5,0451014	3,57065523	-70,8%
	T2	21	-5,5504400	3,20905287	-57,8%
	Total	42	-5,2977707	3,36275633	-63,5%
Total	T0	43	-4,4812263	3,40695465	-76,0%
	T2	43	-5,3064463	3,27578719	-61,7%
	Total	86	-4,8938363	3,34812038	-68,4%

Dimension du modèle[a]

		Nombre de niveaux	Nombre de paramètres
Effets fixes	Constante	1	1
	Condition	2	1
	Time	2	1
	Condition * Time	4	1
Résidu			1
Total		9	5

a. Variable dépendante : Cool N2 Amplitude.

Critères d'information[a]

Log de vraisemblance restreint -2	443,466
Critère d'information d'Akaike (AIC)	445,466
Critère de Hurvich et Tsai (AICC)	445,516
Critère de Bozdogan (CAIC)	448,872
Critère bayésien de Schwartz (BIC)	447,872

Les critères d'informations sont présentés en plus petit, disposant d'un meilleur format.[a]

a. Variable dépendante : Cool N2 Amplitude.

Effets fixes

Tests des effets fixes de type III[a]

Source	Ddl du numérateur	Ddl du dénominateur	F	Sig.
Constante	1	82	183,629	,000
Condition	1	82	1,190	,278
Time	1	82	1,278	,262
Condition * Time	1	82	,187	,667

a. Variable dépendante : Cool N2 Amplitude.

Estimations des effets fixes[a]

Paramètre	Estimation	Erreur standard	ddl	t	Sig.
Constante	-5,550440	,732007	82	-7,582	,000
[Condition=Sham]	,476897	1,023383	82	,466	,642
[Condition=taVNS]	0[b]	0	.	.	.
[Time=T0]	,505339	1,035214	82	,488	,627
[Time=T2]	0[b]	0	.	.	.
[Condition=Sham] * [Time=T0]	,625223	1,447282	82	,432	,667
[Condition=Sham] * [Time=T2]	0[b]	0	.	.	.
[Condition=taVNS] * [Time=T0]	0[b]	0	.	.	.
[Condition=taVNS] * [Time=T2]	0[b]	0	.	.	.

Estimations des effets fixes[a]

Paramètre	Intervalle de confiance à 95 %	
	Borne inférieure	Borne supérieure
Constante	-7,006635	-4,094245
[Condition=Sham]	-1,558938	2,512732
[Condition=taVNS]	.	.
[Time=T0]	-1,554033	2,564710

[Time=T2]	.	.
[Condition=Sham] * [Time=T0]	-2,253882	3,504328
[Condition=Sham] * [Time=T2]	.	.
[Condition=taVNS] * [Time=T0]	.	.
[Condition=taVNS] * [Time=T2]	.	.

a. Variable dépendante : Cool N2 Amplitude.

b. Ce paramètre est défini sur 0, car il est redondant.

Matrice de corrélation pour les estimations des effets fixes[a]

Paramètre	Constante	[Condition=Sham]	[Condition=taVNS]	[Time=T0]	[Time=T2]
Constante	1	-,715	.[b]	-,707	.[b]
[Condition=Sham]	-,715	1	.[b]	,506	.[b]
[Condition=taVNS]	.[b]	.[b]	.[b]	.[b]	.[b]
[Time=T0]	-,707	,506	.[b]	1	.[b]
[Time=T2]	.[b]	.[b]	.[b]	.[b]	.[b]
[Condition=Sham] * [Time=T0]	,506	-,707	.[b]	-,715	.[b]
[Condition=Sham] * [Time=T2]	.[b]	.[b]	.[b]	.[b]	.[b]
[Condition=taVNS] * [Time=T0]	.[b]	.[b]	.[b]	.[b]	.[b]
[Condition=taVNS] * [Time=T2]	.[b]	.[b]	.[b]	.[b]	.[b]

Matrice de corrélation pour les estimations des effets fixes[a]

Paramètre	[Condition=Sham] * [Time=T0]	[Condition=Sham] * [Time=T2]	[Condition=taVNS] * [Time=T0]	[Condition=taVNS] * [Time=T2]
Constante	,506	.[b]	.[b]	.[b]
[Condition=Sham]	-,707	.[b]	.[b]	.[b]
[Condition=taVNS]	.[b]	.[b]	.[b]	.[b]
[Time=T0]	-,715	.[b]	.[b]	.[b]
[Time=T2]	.[b]	.[b]	.[b]	.[b]
[Condition=Sham] * [Time=T0]	1	.[b]	.[b]	.[b]
[Condition=Sham] * [Time=T2]	.[b]	.[b]	.[b]	.[b]
[Condition=taVNS] * [Time=T0]	.[b]	.[b]	.[b]	.[b]
[Condition=taVNS] * [Time=T2]	.[b]	.[b]	.[b]	.[b]

a. Variable dépendante : Cool N2 Amplitude.

b. La corrélation est manquante par défaut, car elle est associée à un paramètre redondant.

Matrice de covariance pour les estimations des effets fixes[a]

Paramètre	Constante	[Condition=Sham]	[Condition=taVNS]	[Time=T0]	[Time=T2]
Constante	,535834	-,535834	0[b]	-,535834	0[b]
[Condition=Sham]	-,535834	1,047313	0[b]	,535834	0[b]
[Condition=taVNS]	0[b]	0[b]	0[b]	0[b]	0[b]
[Time=T0]	-,535834	,535834	0[b]	1,071669	0[b]
[Time=T2]	0[b]	0[b]	0[b]	0[b]	0[b]
[Condition=Sham] * [Time=T0]	,535834	-1,047313	0[b]	-1,071669	0[b]
[Condition=Sham] * [Time=T2]	0[b]	0[b]	0[b]	0[b]	0[b]
[Condition=taVNS] * [Time=T0]	0[b]	0[b]	0[b]	0[b]	0[b]
[Condition=taVNS] * [Time=T2]	0[b]	0[b]	0[b]	0[b]	0[b]

Matrice de covariance pour les estimations des effets fixes[a]

Paramètre	[Condition=Sham] * [Time=T0]	[Condition=Sham] * [Time=T2]	[Condition=taVNS] * [Time=T0]	[Condition=taVNS] * [Time=T2]
Constante	,535834	0[b]	0[b]	0[b]
[Condition=Sham]	-1,047313	0[b]	0[b]	0[b]
[Condition=taVNS]	0[b]	0[b]	0[b]	0[b]
[Time=T0]	-1,071669	0[b]	0[b]	0[b]
[Time=T2]	0[b]	0[b]	0[b]	0[b]
[Condition=Sham] * [Time=T0]	2,094626	0[b]	0[b]	0[b]
[Condition=Sham] * [Time=T2]	0[b]	0[b]	0[b]	0[b]
[Condition=taVNS] * [Time=T0]	0[b]	0[b]	0[b]	0[b]
[Condition=taVNS] * [Time=T2]	0[b]	0[b]	0[b]	0[b]

a. Variable dépendante : Cool N2 Amplitude.

b. La covariance est définie sur 0, car elle est associée à un paramètre redondant.

Paramètres de covariance

Estimations des paramètres de covariance[a]

Paramètre	Estimation	Erreur standard	Z de Wald	Sig.	Intervalle de confiance à 95 % Borne inférieure	Borne supérieure
Résidu	11,252524	1,757349	6,403	,000	8,285421	15,282179

a. Variable dépendante : Cool N2 Amplitude.

Matrice de corrélation pour les estimations des paramètres de covariance[a]

Paramètre	Résidu
Résidu	1

a. Variable dépendante : Cool N2 Amplitude.

Matrice de covariance pour les estimations des paramètres de covariance[a]

Paramètre	Résidu
Résidu	3,088275

a. Variable dépendante : Cool N2 Amplitude.

Moyenne marginale estimée

1. Grand Mean[a]

Moyenne	Erreur standard	ddl	Intervalle de confiance à 95 % Borne inférieure	Borne supérieure
-4,903	,362	82	-5,623	-4,183

a. Variable dépendante : Cool N2 Amplitude.

2. Condition

Estimations[a]

Condition	Moyenne	Erreur standard	ddl	Intervalle de confiance à 95 % Borne inférieure	Borne supérieure
Sham	-4,508	,506	82	-5,514	-3,502
taVNS	-5,298	,518	82	-6,327	-4,268

a. Variable dépendante : Cool N2 Amplitude.

Comparaisons appariées[a]

(I) Condition	(J) Condition	Différence moyenne (I-J)	Erreur standard	ddl	Sig.[b]
Sham	taVNS	,790	,724	82	,278
taVNS	Sham	-,790	,724	82	,278

Comparaisons appariées[a]

(I) Condition	(J) Condition	Intervalle de confiance à 95 % pour la différence[b] Borne inférieure	Borne supérieure
Sham	taVNS	-,650	2,229
taVNS	Sham	-2,229	,650

Basées sur les moyennes marginales estimées[a]

a. Variable dépendante : Cool N2 Amplitude.

b. Ajustement pour les comparaisons multiples : Bonferroni.

Tests univariés[a]

Ddl du numérateur	Ddl du dénominateur	F	Sig.
1	82	1,190	,278

Le test de F permet de tester l'effet de Condition. Il s'appuie sur les comparaisons appariées (indépendantes) linéaires parmi les moyennes marginales estimées.[a]

a. Variable dépendante : Cool N2 Amplitude.

3. Time

Estimations[a]

Time	Moyenne	Erreur standard	ddl	Intervalle de confiance à 95 % Borne inférieure	Borne supérieure
T0	-4,494	,512	82	-5,512	-3,476
T2	-5,312	,512	82	-6,330	-4,294

a. Variable dépendante : Cool N2 Amplitude.

Comparaisons appariées[a]

(I) Time	(J) Time	Différence moyenne (I-J)	Erreur standard	ddl	Sig.[b]	Intervalle de confiance à 95 % pour la différence[b] Borne inférieure
T0	T2	,818	,724	82	,262	-,622

T2	T0	-,818	,724	82	,262	-2,258

Comparaisons appariées[a]

(I) Time	(J) Time	Intervalle de confiance à 95 % pour la différence Borne supérieure
T0	T2	2,258
T2	T0	,622

Basées sur les moyennes marginales estimées[a]

a. Variable dépendante : Cool N2 Amplitude.

b. Ajustement pour les comparaisons multiples : Bonferroni.

Tests univariés[a]

Ddl du numérateur	Ddl du dénominateur	F	Sig.
1	82	1,278	,262

Le test de F permet de tester l'effet de Time. Il s'appuie sur les comparaisons appariées (indépendantes) linéaires parmi les moyennes marginales estimées.[a]

a. Variable dépendante : Cool N2 Amplitude.

4. Condition * Time[a]

Condition	Time	Moyenne	Erreur standard	ddl	Intervalle de confiance à 95 % Borne inférieure	Borne supérieure
Sham	T0	-3,943	,715	82	-5,366	-2,520
	T2	-5,074	,715	82	-6,496	-3,651
taVNS	T0	-5,045	,732	82	-6,501	-3,589
	T2	-5,550	,732	82	-7,007	-4,094

a. Variable dépendante : Cool N2 Amplitude.

1.3.3. Cool N2 Latency.

```
MIXED CoolN2Latency BY Condition Time
  /CRITERIA=CIN(95) MXITER(100) MXSTEP(10) SCORING(1)
SINGULAR(0.000000000001) HCONVERGE(0,
    ABSOLUTE) LCONVERGE(0, ABSOLUTE) PCONVERGE(0.000001, ABSOLUTE)
  /FIXED=Condition Time Condition*Time | SSTYPE(3)
  /METHOD=REML
  /PRINT=CPS CORB COVB DESCRIPTIVES G  SOLUTION TESTCOV
  /EMMEANS=TABLES(OVERALL)
  /EMMEANS=TABLES(Condition) COMPARE ADJ(BONFERRONI)
  /EMMEANS=TABLES(Time) COMPARE ADJ(BONFERRONI)
  /EMMEANS=TABLES(Condition*Time) .
```

Remarques

Sortie obtenue		05-MAY-2021 12:22:23
Commentaires		
Entrée	Jeu de données actif	Jeu_de_données1
	Filtre	<sans>
	Pondération	<sans>
	Fichier scindé	<sans>
	N de lignes dans le fichier de travail	1048530
Gestion des valeurs manquantes	Définition de la valeur manquante	Les valeurs manquantes définies par l'utilisateur sont traitées comme étant manquantes.
	Observations utilisées	Les statistiques sont basées sur toutes les observations comportant des données valides pour toutes les variables du modèle.

Syntaxe		MIXED CoolN2Latency BY Condition Time /CRITERIA=CIN(95) MXITER(100) MXSTEP(10) SCORING(1) SINGULAR(0.000000000001) HCONVERGE(0, ABSOLUTE) LCONVERGE(0, ABSOLUTE) PCONVERGE(0.000001, ABSOLUTE) /FIXED=Condition Time Condition*Time | SSTYPE(3) /METHOD=REML /PRINT=CPS CORB COVB DESCRIPTIVES G SOLUTION TESTCOV /EMMEANS=TABLES(OVERALL) /EMMEANS=TABLES(Condition) COMPARE ADJ(BONFERRONI) /EMMEANS=TABLES(Time) COMPARE ADJ(BONFERRONI) /EMMEANS=TABLES(Condition*Time) .
Ressources	Temps de processeur	00:00:00,75
	Temps écoulé	00:00:00,75

Récapitulatif de traitement des observations

		Effectif	Pourcentage marginal
Condition	Sham	43	50,6%
	taVNS	42	49,4%
Time	T0	42	49,4%

	T2	43	50,6%
Valide		85	100,0%
Exclues		1048445	
Total		1048530	

Statistiques descriptives

Cool N2 Latency

Condition	Time	Effectif	Moyenne	Ecart type	Coefficient de variation
Sham	T0	21	,19709138	,075007957	38,1%
	T2	22	,22127273	,089734316	40,6%
	Total	43	,20946323	,082794007	39,5%
taVNS	T0	21	,20475476	,094992283	46,4%
	T2	21	,18580476	,064019748	34,5%
	Total	42	,19527976	,080578975	41,3%
Total	T0	42	,20092307	,084624160	42,1%
	T2	43	,20395116	,079370094	38,9%
	Total	85	,20245493	,081532181	40,3%

Dimension du modèle[a]

		Nombre de niveaux	Nombre de paramètres
Effets fixes	Constante	1	1
	Condition	2	1
	Time	2	1
	Condition * Time	4	1
Résidu			1
Total		9	5

a. Variable dépendante : Cool N2 Latency.

Critères d'information[a]

Log de vraisemblance restreint -2	-163,162
Critère d'information d'Akaike (AIC)	-161,162
Critère de Hurvich et Tsai (AICC)	-161,111
Critère de Bozdogan (CAIC)	-157,767
Critère bayésien de Schwartz (BIC)	-158,767

Les critères d'informations sont présentés en plus petit, disposant d'un meilleur format.[a]

a. Variable dépendante : Cool N2 Latency.

Effets fixes

Tests des effets fixes de type III[a]

Source	Ddl du numérateur	Ddl du dénominateur	F	Sig.
Constante	1	81	517,338	,000
Condition	1	81	,611	,437
Time	1	81	,022	,883
Condition * Time	1	81	1,471	,229

a. Variable dépendante : Cool N2 Latency.

Estimations des effets fixes[a]

Paramètre	Estimation	Erreur standard	ddl	t	Sig.
Constante	,185805	,017884	81	10,389	,000
[Condition=Sham]	,035468	,025003	81	1,419	,160
[Condition=taVNS]	0[b]	0	.	.	.
[Time=T0]	,018950	,025292	81	,749	,456
[Time=T2]	0[b]	0	.	.	.
[Condition=Sham] * [Time=T0]	-,043131	,035565	81	-1,213	,229
[Condition=Sham] * [Time=T2]	0[b]	0	.	.	.
[Condition=taVNS] * [Time=T0]	0[b]	0	.	.	.
[Condition=taVNS] * [Time=T2]	0[b]	0	.	.	.

Estimations des effets fixes[a]

Paramètre	Intervalle de confiance à 95 %	
	Borne inférieure	Borne supérieure
Constante	,150221	,221389
[Condition=Sham]	-,014280	,085216
[Condition=taVNS]	.	.
[Time=T0]	-,031374	,069274
[Time=T2]	.	.

[Condition=Sham] * [Time=T0]	-,113894	,027631
[Condition=Sham] * [Time=T2]	.	.
[Condition=taVNS] * [Time=T0]	.	.
[Condition=taVNS] * [Time=T2]	.	.

a. Variable dépendante : Cool N2 Latency.

b. Ce paramètre est défini sur 0, car il est redondant.

Matrice de corrélation pour les estimations des effets fixes[a]

Paramètre	Constante	[Condition=Sham]	[Condition=taVNS]	[Time=T0]	[Time=T2]
Constante	1	-,715	.[b]	-,707	.[b]
[Condition=Sham]	-,715	1	.[b]	,506	.[b]
[Condition=taVNS]	.[b]	.[b]	.[b]	.[b]	.[b]
[Time=T0]	-,707	,506	.[b]	1	.[b]
[Time=T2]	.[b]	.[b]	.[b]	.[b]	.[b]
[Condition=Sham] * [Time=T0]	,503	-,703	.[b]	-,711	.[b]
[Condition=Sham] * [Time=T2]	.[b]	.[b]	.[b]	.[b]	.[b]
[Condition=taVNS] * [Time=T0]	.[b]	.[b]	.[b]	.[b]	.[b]
[Condition=taVNS] * [Time=T2]	.[b]	.[b]	.[b]	.[b]	.[b]

Matrice de corrélation pour les estimations des effets fixes[a]

Paramètre	[Condition=Sham] * [Time=T0]	[Condition=Sham] * [Time=T2]	[Condition=taVNS] * [Time=T0]	[Condition=taVNS] * [Time=T2]
Constante	,503	.[b]	.[b]	.[b]
[Condition=Sham]	-,703	.[b]	.[b]	.[b]
[Condition=taVNS]	.[b]	.[b]	.[b]	.[b]
[Time=T0]	-,711	.[b]	.[b]	.[b]
[Time=T2]	.[b]	.[b]	.[b]	.[b]
[Condition=Sham] * [Time=T0]	1	.[b]	.[b]	.[b]
[Condition=Sham] * [Time=T2]	.[b]	.[b]	.[b]	.[b]
[Condition=taVNS] * [Time=T0]	.[b]	.[b]	.[b]	.[b]
[Condition=taVNS] * [Time=T2]	.[b]	.[b]	.[b]	.[b]

a. Variable dépendante : Cool N2 Latency.

b. La corrélation est manquante par défaut, car elle est associée à un paramètre redondant.

Matrice de covariance pour les estimations des effets fixes[a]

Paramètre	Constante	[Condition=Sham]	[Condition=taVNS]	[Time=T0]	[Time=T2]
Constante	,000320	-,000320	0[b]	-,000320	0[b]
[Condition=Sham]	-,000320	,000625	0[b]	,000320	0[b]
[Condition=taVNS]	0[b]	0[b]	0[b]	0[b]	0[b]
[Time=T0]	-,000320	,000320	0[b]	,000640	0[b]
[Time=T2]	0[b]	0[b]	0[b]	0[b]	0[b]
[Condition=Sham] * [Time=T0]	,000320	-,000625	0[b]	-,000640	0[b]
[Condition=Sham] * [Time=T2]	0[b]	0[b]	0[b]	0[b]	0[b]
[Condition=taVNS] * [Time=T0]	0[b]	0[b]	0[b]	0[b]	0[b]
[Condition=taVNS] * [Time=T2]	0[b]	0[b]	0[b]	0[b]	0[b]

Matrice de covariance pour les estimations des effets fixes[a]

Paramètre	[Condition=Sham] * [Time=T0]	[Condition=Sham] * [Time=T2]	[Condition=taVNS] * [Time=T0]	[Condition=taVNS] * [Time=T2]
Constante	,000320	0[b]	0[b]	0[b]
[Condition=Sham]	-,000625	0[b]	0[b]	0[b]
[Condition=taVNS]	0[b]	0[b]	0[b]	0[b]
[Time=T0]	-,000640	0[b]	0[b]	0[b]
[Time=T2]	0[b]	0[b]	0[b]	0[b]
[Condition=Sham] * [Time=T0]	,001265	0[b]	0[b]	0[b]
[Condition=Sham] * [Time=T2]	0[b]	0[b]	0[b]	0[b]
[Condition=taVNS] * [Time=T0]	0[b]	0[b]	0[b]	0[b]
[Condition=taVNS] * [Time=T2]	0[b]	0[b]	0[b]	0[b]

a. Variable dépendante : Cool N2 Latency.

b. La covariance est définie sur 0, car elle est associée à un paramètre redondant.

Paramètres de covariance

Estimations des paramètres de covariance[a]

Paramètre	Estimation	Erreur standard	Z de Wald	Sig.	Intervalle de confiance à 95 % Borne inférieure	Intervalle de confiance à 95 % Borne supérieure
Résidu	,006717	,001055	6,364	,000	,004936	,009139

a. Variable dépendante : Cool N2 Latency.

Matrice de corrélation pour les estimations des paramètres de covariance[a]

Paramètre	Résidu
Résidu	1

a. Variable dépendante : Cool N2 Latency.

Matrice de covariance pour les estimations des paramètres de covariance[a]

Paramètre	Résidu
Résidu	1,113967E-6

a. Variable dépendante : Cool N2 Latency.

Moyenne marginale estimée

1. Grand Mean[a]

			Intervalle de confiance à 95 %	
Moyenne	Erreur standard	ddl	Borne inférieure	Borne supérieure
,202	,009	81	,185	,220

a. Variable dépendante : Cool N2 Latency.

2. Condition

Estimations[a]

				Intervalle de confiance à 95 %	
Condition	Moyenne	Erreur standard	ddl	Borne inférieure	Borne supérieure
Sham	,209	,013	81	,184	,234
taVNS	,195	,013	81	,170	,220

a. Variable dépendante : Cool N2 Latency.

Comparaisons appariées[a]

(I) Condition	(J) Condition	Différence moyenne (I-J)	Erreur standard	ddl	Sig.[b]
Sham	taVNS	,014	,018	81	,437
taVNS	Sham	-,014	,018	81	,437

Comparaisons appariées[a]

(I) Condition	(J) Condition	Intervalle de confiance à 95 % pour la différence[b] Borne inférieure	Borne supérieure
Sham	taVNS	-,021	,049
taVNS	Sham	-,049	,021

Basées sur les moyennes marginales estimées[a]

a. Variable dépendante : Cool N2 Latency.

b. Ajustement pour les comparaisons multiples : Bonferroni.

Tests univariés[a]

Ddl du numérateur	Ddl du dénominateur	F	Sig.
1	81	,611	,437

Le test de F permet de tester l'effet de Condition. Il s'appuie sur les comparaisons appariées (indépendantes) linéaires parmi les moyennes marginales estimées.[a]

a. Variable dépendante : Cool N2 Latency.

3. Time

Estimations[a]

Time	Moyenne	Erreur standard	ddl	Intervalle de confiance à 95 % Borne inférieure	Borne supérieure
T0	,201	,013	81	,176	,226
T2	,204	,013	81	,179	,228

a. Variable dépendante : Cool N2 Latency.

Comparaisons appariées[a]

(I) Time	(J) Time	Différence moyenne (I-J)	Erreur standard	ddl	Sig.[b]	Intervalle de confiance à 95 % pour la différence[b] Borne inférieure

T0	T2	-,003	,018	81	,883	-,038
T2	T0	,003	,018	81	,883	-,033

Comparaisons appariées[a]

(I) Time	(J) Time	Intervalle de confiance à 95 % pour la différence Borne supérieure
T0	T2	,033
T2	T0	,038

Basées sur les moyennes marginales estimées[a]

a. Variable dépendante : Cool N2 Latency.

b. Ajustement pour les comparaisons multiples : Bonferroni.

Tests univariés[a]

Ddl du numérateur	Ddl du dénominateur	F	Sig.
1	81	,022	,883

Le test de F permet de tester l'effet de Time. Il s'appuie sur les comparaisons appariées (indépendantes) linéaires parmi les moyennes marginales estimées.[a]

a. Variable dépendante : Cool N2 Latency.

4. Condition * Time[a]

Condition	Time	Moyenne	Erreur standard	ddl	Intervalle de confiance à 95 % Borne inférieure	Borne supérieure
Sham	T0	,197	,018	81	,162	,233
	T2	,221	,017	81	,187	,256
taVNS	T0	,205	,018	81	,169	,240
	T2	,186	,018	81	,150	,221

a. Variable dépendante : Cool N2 Latency.

1.3.4. Cool P2 Amplitude.

```
MIXED CoolP2Amplitude BY Condition Time
  /CRITERIA=CIN(95) MXITER(100) MXSTEP(10) SCORING(1)
SINGULAR(0.000000000001) HCONVERGE(0,
    ABSOLUTE) LCONVERGE(0, ABSOLUTE) PCONVERGE(0.000001, ABSOLUTE)
  /FIXED=Condition Time Condition*Time | SSTYPE(3)
  /METHOD=REML
  /PRINT=CPS CORB COVB DESCRIPTIVES G  SOLUTION TESTCOV
  /EMMEANS=TABLES(OVERALL)
  /EMMEANS=TABLES(Condition) COMPARE ADJ(BONFERRONI)
  /EMMEANS=TABLES(Time) COMPARE ADJ(BONFERRONI)
  /EMMEANS=TABLES(Condition*Time) .
```

Remarques

Sortie obtenue		05-MAY-2021 12:22:57
Commentaires		
Entrée	Jeu de données actif	Jeu_de_données1
	Filtre	<sans>
	Pondération	<sans>
	Fichier scindé	<sans>
	N de lignes dans le fichier de travail	1048530
Gestion des valeurs manquantes	Définition de la valeur manquante	Les valeurs manquantes définies par l'utilisateur sont traitées comme étant manquantes.
	Observations utilisées	Les statistiques sont basées sur toutes les observations comportant des données valides pour toutes les variables du modèle.

Syntaxe		MIXED CoolP2Amplitude BY Condition Time /CRITERIA=CIN(95) MXITER(100) MXSTEP(10) SCORING(1) SINGULAR(0.000000000001) HCONVERGE(0, ABSOLUTE) LCONVERGE(0, ABSOLUTE) PCONVERGE(0.000001, ABSOLUTE) /FIXED=Condition Time Condition*Time | SSTYPE(3) /METHOD=REML /PRINT=CPS CORB COVB DESCRIPTIVES G SOLUTION TESTCOV /EMMEANS=TABLES(OVERALL) /EMMEANS=TABLES(Condition) COMPARE ADJ(BONFERRONI) /EMMEANS=TABLES(Time) COMPARE ADJ(BONFERRONI) /EMMEANS=TABLES(Condition*Time) .
Ressources	Temps de processeur	00:00:00,75
	Temps écoulé	00:00:00,76

Récapitulatif de traitement des observations

		Effectif	Pourcentage marginal
Condition	Sham	44	51,2%
	taVNS	42	48,8%
Time	T0	43	50,0%

	T2	43	50,0%
Valide		86	100,0%
Exclues		1048444	
Total		1048530	

Statistiques descriptives

Cool P2 Amplitude

Condition	Time	Effectif	Moyenne	Ecart type	Coefficient de variation
Sham	T0	22	6,8101773	3,61907411	53,1%
	T2	22	5,4118832	4,27125371	78,9%
	Total	44	6,1110302	3,97572550	65,1%
taVNS	T0	21	6,6468619	3,77608293	56,8%
	T2	21	5,7607676	4,66234298	80,9%
	Total	42	6,2038148	4,21428783	67,9%
Total	T0	43	6,7304186	3,65315995	54,3%
	T2	43	5,5822686	4,41634518	79,1%
	Total	86	6,1563436	4,07001807	66,1%

Dimension du modèle[a]

		Nombre de niveaux	Nombre de paramètres
Effets fixes	Constante	1	1
	Condition	2	1
	Time	2	1
	Condition * Time	4	1
Résidu			1
Total		9	5

a. Variable dépendante : Cool P2 Amplitude.

Critères d'information[a]

Log de vraisemblance restreint -2	476,359
Critère d'information d'Akaike (AIC)	478,359
Critère de Hurvich et Tsai (AICC)	478,409
Critère de Bozdogan (CAIC)	481,766
Critère bayésien de Schwartz (BIC)	480,766

Les critères d'informations sont présentés en plus petit, disposant d'un meilleur format.[a]

a. Variable dépendante : Cool P2 Amplitude.

Effets fixes

Tests des effets fixes de type III[a]

Source	Ddl du numérateur	Ddl du dénominateur	F	Sig.
Constante	1	82	193,909	,000
Condition	1	82	,011	,917
Time	1	82	1,668	,200
Condition * Time	1	82	,084	,773

a. Variable dépendante : Cool P2 Amplitude.

Estimations des effets fixes[a]

Paramètre	Estimation	Erreur standard	ddl	t	Sig.
Constante	5,760768	,894587	82	6,440	,000
[Condition=Sham]	-,348884	1,250678	82	-,279	,781
[Condition=taVNS]	0[b]	0	.	.	.
[Time=T0]	,886094	1,265137	82	,700	,486
[Time=T2]	0[b]	0	.	.	.
[Condition=Sham] * [Time=T0]	,512200	1,768726	82	,290	,773
[Condition=Sham] * [Time=T2]	0[b]	0	.	.	.
[Condition=taVNS] * [Time=T0]	0[b]	0	.	.	.
[Condition=taVNS] * [Time=T2]	0[b]	0	.	.	.

Estimations des effets fixes[a]

Paramètre	Intervalle de confiance à 95 % Borne inférieure	Borne supérieure
Constante	3,981149	7,540386
[Condition=Sham]	-2,836881	2,139112
[Condition=taVNS]	.	.
[Time=T0]	-1,630666	3,402855

[Time=T2]	.	.
[Condition=Sham] * [Time=T0]	-3,006359	4,030758
[Condition=Sham] * [Time=T2]	.	.
[Condition=taVNS] * [Time=T0]	.	.
[Condition=taVNS] * [Time=T2]	.	.

a. Variable dépendante : Cool P2 Amplitude.

b. Ce paramètre est défini sur 0, car il est redondant.

Matrice de corrélation pour les estimations des effets fixes[a]

Paramètre	Constante	[Condition=Sham]	[Condition=taVNS]	[Time=T0]	[Time=T2]
Constante	1	-,715	.[b]	-,707	.[b]
[Condition=Sham]	-,715	1	.[b]	,506	.[b]
[Condition=taVNS]	.[b]	.[b]	.[b]	.[b]	.[b]
[Time=T0]	-,707	,506	.[b]	1	.[b]
[Time=T2]	.[b]	.[b]	.[b]	.[b]	.[b]
[Condition=Sham] * [Time=T0]	,506	-,707	.[b]	-,715	.[b]
[Condition=Sham] * [Time=T2]	.[b]	.[b]	.[b]	.[b]	.[b]
[Condition=taVNS] * [Time=T0]	.[b]	.[b]	.[b]	.[b]	.[b]
[Condition=taVNS] * [Time=T2]	.[b]	.[b]	.[b]	.[b]	.[b]

Matrice de corrélation pour les estimations des effets fixes[a]

Paramètre	[Condition=Sham] * [Time=T0]	[Condition=Sham] * [Time=T2]	[Condition=taVNS] * [Time=T0]	[Condition=taVNS] * [Time=T2]
Constante	,506	.[b]	.[b]	.[b]
[Condition=Sham]	-,707	.[b]	.[b]	.[b]
[Condition=taVNS]	.[b]	.[b]	.[b]	.[b]
[Time=T0]	-,715	.[b]	.[b]	.[b]
[Time=T2]	.[b]	.[b]	.[b]	.[b]
[Condition=Sham] * [Time=T0]	1	.[b]	.[b]	.[b]
[Condition=Sham] * [Time=T2]	.[b]	.[b]	.[b]	.[b]
[Condition=taVNS] * [Time=T0]	.[b]	.[b]	.[b]	.[b]
[Condition=taVNS] * [Time=T2]	.[b]	.[b]	.[b]	.[b]

a. Variable dépendante : Cool P2 Amplitude.

b. La corrélation est manquante par défaut, car elle est associée à un paramètre redondant.

Matrice de covariance pour les estimations des effets fixes[a]

Paramètre	Constante	[Condition=Sham]	[Condition=taVNS]	[Time=T0]	[Time=T2]
Constante	,800286	-,800286	0[b]	-,800286	0[b]
[Condition=Sham]	-,800286	1,564195	0[b]	,800286	0[b]
[Condition=taVNS]	0[b]	0[b]	0[b]	0[b]	0[b]
[Time=T0]	-,800286	,800286	0[b]	1,600572	0[b]
[Time=T2]	0[b]	0[b]	0[b]	0[b]	0[b]
[Condition=Sham] * [Time=T0]	,800286	-1,564195	0[b]	-1,600572	0[b]
[Condition=Sham] * [Time=T2]	0[b]	0[b]	0[b]	0[b]	0[b]
[Condition=taVNS] * [Time=T0]	0[b]	0[b]	0[b]	0[b]	0[b]
[Condition=taVNS] * [Time=T2]	0[b]	0[b]	0[b]	0[b]	0[b]

Matrice de covariance pour les estimations des effets fixes[a]

Paramètre	[Condition=Sham] * [Time=T0]	[Condition=Sham] * [Time=T2]	[Condition=taVNS] * [Time=T0]	[Condition=taVNS] * [Time=T2]
Constante	,800286	0[b]	0[b]	0[b]
[Condition=Sham]	-1,564195	0[b]	0[b]	0[b]
[Condition=taVNS]	0[b]	0[b]	0[b]	0[b]
[Time=T0]	-1,600572	0[b]	0[b]	0[b]
[Time=T2]	0[b]	0[b]	0[b]	0[b]
[Condition=Sham] * [Time=T0]	3,128390	0[b]	0[b]	0[b]
[Condition=Sham] * [Time=T2]	0[b]	0[b]	0[b]	0[b]
[Condition=taVNS] * [Time=T0]	0[b]	0[b]	0[b]	0[b]
[Condition=taVNS] * [Time=T2]	0[b]	0[b]	0[b]	0[b]

a. Variable dépendante : Cool P2 Amplitude.

b. La covariance est définie sur 0, car elle est associée à un paramètre redondant.

Paramètres de covariance

Estimations des paramètres de covariance[a]

Paramètre	Estimation	Erreur standard	Z de Wald	Sig.	Intervalle de confiance à 95 % Borne inférieure	Borne supérieure
Résidu	16,806004	2,624657	6,403	,000	12,374541	22,824423

a. Variable dépendante : Cool P2 Amplitude.

Matrice de corrélation pour les estimations des paramètres de covariance[a]

Paramètre	Résidu
Résidu	1

a. Variable dépendante : Cool P2 Amplitude.

Matrice de covariance pour les estimations des paramètres de covariance[a]

Paramètre	Résidu
Résidu	6,888823

a. Variable dépendante : Cool P2 Amplitude.

Moyenne marginale estimée

1. Grand Mean[a]

Moyenne	Erreur standard	ddl	Intervalle de confiance à 95 %	
			Borne inférieure	Borne supérieure
6,157	,442	82	5,278	7,037

a. Variable dépendante : Cool P2 Amplitude.

2. Condition

Estimations[a]

Condition	Moyenne	Erreur standard	ddl	Intervalle de confiance à 95 %	
				Borne inférieure	Borne supérieure
Sham	6,111	,618	82	4,882	7,340
taVNS	6,204	,633	82	4,945	7,462

a. Variable dépendante : Cool P2 Amplitude.

Comparaisons appariées[a]

(I) Condition	(J) Condition	Différence moyenne (I-J)	Erreur standard	ddl	Sig.[b]
Sham	taVNS	-,093	,884	82	,917
taVNS	Sham	,093	,884	82	,917

Comparaisons appariées[a]

(I) Condition	(J) Condition	Intervalle de confiance à 95 % pour la différence[b] Borne inférieure	Borne supérieure
Sham	taVNS	-1,852	1,666
taVNS	Sham	-1,666	1,852

Basées sur les moyennes marginales estimées[a]

a. Variable dépendante : Cool P2 Amplitude.

b. Ajustement pour les comparaisons multiples : Bonferroni.

Tests univariés[a]

Ddl du numérateur	Ddl du dénominateur	F	Sig.
1	82	,011	,917

Le test de F permet de tester l'effet de Condition. Il s'appuie sur les comparaisons appariées (indépendantes) linéaires parmi les moyennes marginales estimées.[a]

a. Variable dépendante : Cool P2 Amplitude.

3. Time

Estimations[a]

Time	Moyenne	Erreur standard	ddl	Intervalle de confiance à 95 % Borne inférieure	Borne supérieure
T0	6,729	,625	82	5,485	7,973
T2	5,586	,625	82	4,342	6,830

a. Variable dépendante : Cool P2 Amplitude.

Comparaisons appariées[a]

(I) Time	(J) Time	Différence moyenne (I-J)	Erreur standard	ddl	Sig.[b]	Intervalle de confiance à 95 % pour la différence[b] Borne inférieure
T0	T2	1,142	,884	82	,200	-,617
T2	T0	-1,142	,884	82	,200	-2,901

Comparaisons appariées[a]

(I) Time	(J) Time	Intervalle de confiance à 95 % pour la différence Borne supérieure
T0	T2	2,901
T2	T0	,617

Basées sur les moyennes marginales estimées[a]

a. Variable dépendante : Cool P2 Amplitude.

b. Ajustement pour les comparaisons multiples : Bonferroni.

Tests univariés[a]

Ddl du numérateur	Ddl du dénominateur	F	Sig.
1	82	1,668	,200

Le test de F permet de tester l'effet de Time. Il s'appuie sur les comparaisons appariées (indépendantes) linéaires parmi les moyennes marginales estimées.[a]

a. Variable dépendante : Cool P2 Amplitude.

4. Condition * Time[a]

Condition	Time	Moyenne	Erreur standard	ddl	Intervalle de confiance à 95 % Borne inférieure	Borne supérieure
Sham	T0	6,810	,874	82	5,071	8,549
	T2	5,412	,874	82	3,673	7,151
taVNS	T0	6,647	,895	82	4,867	8,426
	T2	5,761	,895	82	3,981	7,540

a. Variable dépendante : Cool P2 Amplitude.

1.3.5. Cool P2 Latency.

```
MIXED CoolP2Latency BY Condition Time
  /CRITERIA=CIN(95) MXITER(100) MXSTEP(10) SCORING(1)
SINGULAR(0.000000000001) HCONVERGE(0,
    ABSOLUTE) LCONVERGE(0, ABSOLUTE) PCONVERGE(0.000001, ABSOLUTE)
  /FIXED=Condition Time Condition*Time | SSTYPE(3)
  /METHOD=REML
  /PRINT=CPS CORB COVB DESCRIPTIVES G  SOLUTION TESTCOV
  /EMMEANS=TABLES(OVERALL)
  /EMMEANS=TABLES(Condition) COMPARE ADJ(BONFERRONI)
  /EMMEANS=TABLES(Time) COMPARE ADJ(BONFERRONI)
  /EMMEANS=TABLES(Condition*Time) .
```

Remarques

Sortie obtenue		05-MAY-2021 12:23:27
Commentaires		
Entrée	Jeu de données actif	Jeu_de_données1
	Filtre	<sans>
	Pondération	<sans>
	Fichier scindé	<sans>
	N de lignes dans le fichier de travail	1048530
Gestion des valeurs manquantes	Définition de la valeur manquante	Les valeurs manquantes définies par l'utilisateur sont traitées comme étant manquantes.
	Observations utilisées	Les statistiques sont basées sur toutes les observations comportant des données valides pour toutes les variables du modèle.

Syntaxe		MIXED CoolP2Latency BY Condition Time<br>/CRITERIA=CIN(95) MXITER(100) MXSTEP(10) SCORING(1) SINGULAR(0.000000000001) HCONVERGE(0, ABSOLUTE) LCONVERGE(0, ABSOLUTE) PCONVERGE(0.000001, ABSOLUTE)<br>/FIXED=Condition Time Condition*Time | SSTYPE(3)<br>/METHOD=REML<br>/PRINT=CPS CORB COVB DESCRIPTIVES G SOLUTION TESTCOV<br>/EMMEANS=TABLES(OVERALL)<br>/EMMEANS=TABLES(Condition) COMPARE ADJ(BONFERRONI)<br>/EMMEANS=TABLES(Time) COMPARE ADJ(BONFERRONI)<br>/EMMEANS=TABLES(Condition*Time) .
Ressources	Temps de processeur	00:00:00,77
	Temps écoulé	00:00:00,77

Récapitulatif de traitement des observations

		Effectif	Pourcentage marginal
Condition	Sham	43	50,6%
	taVNS	42	49,4%
Time	T0	42	49,4%

	T2	43	50,6%
Valide		85	100,0%
Exclues		1048445	
Total		1048530	

Statistiques descriptives

Cool P2 latency

Condition	Time	Effectif	Moyenne	Ecart type	Coefficient de variation
Sham	T0	21	,3709110	,05783397	15,6%
	T2	22	,3342814	,06554096	19,6%
	Total	43	,3521702	,06390452	18,1%
taVNS	T0	21	,3675257	,08869333	24,1%
	T2	21	,3553876	,08457030	23,8%
	Total	42	,3614567	,08581316	23,7%
Total	T0	42	,3692183	,07397195	20,0%
	T2	43	,3445891	,07528310	21,8%
	Total	85	,3567588	,07521962	21,1%

Dimension du modèle[a]

		Nombre de niveaux	Nombre de paramètres
Effets fixes	Constante	1	1
	Condition	2	1
	Time	2	1
	Condition * Time	4	1
Résidu			1
Total		9	5

a. Variable dépendante : Cool P2 latency.

Critères d'information[a]

Log de vraisemblance restreint -2	-177,202
Critère d'information d'Akaike (AIC)	-175,202
Critère de Hurvich et Tsai (AICC)	-175,152
Critère de Bozdogan (CAIC)	-171,808
Critère bayésien de Schwartz (BIC)	-172,808

Les critères d'informations sont présentés en plus petit, disposant d'un meilleur format.[a]

a. Variable dépendante : Cool P2 latency.

Effets fixes

Tests des effets fixes de type III[a]

Source	Ddl du numérateur	Ddl du dénominateur	F	Sig.
Constante	1	81	1917,608	,000
Condition	1	81	,295	,588
Time	1	81	2,236	,139
Condition * Time	1	81	,564	,455

a. Variable dépendante : Cool P2 latency.

Estimations des effets fixes[a]

Paramètre	Estimation	Erreur standard	ddl	t	Sig.
Constante	,355388	,016400	81	21,671	,000
[Condition=Sham]	-,021106	,022927	81	-,921	,360
[Condition=taVNS]	0[b]	0	.	.	.
[Time=T0]	,012138	,023192	81	,523	,602
[Time=T2]	0[b]	0	.	.	.
[Condition=Sham] * [Time=T0]	,024491	,032612	81	,751	,455
[Condition=Sham] * [Time=T2]	0[b]	0	.	.	.
[Condition=taVNS] * [Time=T0]	0[b]	0	.	.	.
[Condition=taVNS] * [Time=T2]	0[b]	0	.	.	.

Estimations des effets fixes[a]

Paramètre	Intervalle de confiance à 95 %	
	Borne inférieure	Borne supérieure
Constante	,322758	,388018
[Condition=Sham]	-,066725	,024512
[Condition=taVNS]	.	.
[Time=T0]	-,034008	,058284
[Time=T2]	.	.

[Condition=Sham] * [Time=T0]	-,040397	,089380
[Condition=Sham] * [Time=T2]	.	.
[Condition=taVNS] * [Time=T0]	.	.
[Condition=taVNS] * [Time=T2]	.	.

a. Variable dépendante : Cool P2 latency.

b. Ce paramètre est défini sur 0, car il est redondant.

Matrice de corrélation pour les estimations des effets fixes[a]

Paramètre	Constante	[Condition=Sham]	[Condition=taVNS]	[Time=T0]	[Time=T2]
Constante	1	-,715	.[b]	-,707	.[b]
[Condition=Sham]	-,715	1	.[b]	,506	.[b]
[Condition=taVNS]	.[b]	.[b]	.[b]	.[b]	.[b]
[Time=T0]	-,707	,506	.[b]	1	.[b]
[Time=T2]	.[b]	.[b]	.[b]	.[b]	.[b]
[Condition=Sham] * [Time=T0]	,503	-,703	.[b]	-,711	.[b]
[Condition=Sham] * [Time=T2]	.[b]	.[b]	.[b]	.[b]	.[b]
[Condition=taVNS] * [Time=T0]	.[b]	.[b]	.[b]	.[b]	.[b]
[Condition=taVNS] * [Time=T2]	.[b]	.[b]	.[b]	.[b]	.[b]

Matrice de corrélation pour les estimations des effets fixes[a]

Paramètre	[Condition=Sham] * [Time=T0]	[Condition=Sham] * [Time=T2]	[Condition=taVNS] * [Time=T0]	[Condition=taVNS] * [Time=T2]
Constante	,503	.[b]	.[b]	.[b]
[Condition=Sham]	-,703	.[b]	.[b]	.[b]
[Condition=taVNS]	.[b]	.[b]	.[b]	.[b]
[Time=T0]	-,711	.[b]	.[b]	.[b]
[Time=T2]	.[b]	.[b]	.[b]	.[b]
[Condition=Sham] * [Time=T0]	1	.[b]	.[b]	.[b]
[Condition=Sham] * [Time=T2]	.[b]	.[b]	.[b]	.[b]
[Condition=taVNS] * [Time=T0]	.[b]	.[b]	.[b]	.[b]
[Condition=taVNS] * [Time=T2]	.[b]	.[b]	.[b]	.[b]

a. Variable dépendante : Cool P2 latency.

b. La corrélation est manquante par défaut, car elle est associée à un paramètre redondant.

Matrice de covariance pour les estimations des effets fixes[a]

Paramètre	Constante	[Condition=Sham]	[Condition=taVNS]	[Time=T0]	[Time=T2]
Constante	,000269	-,000269	0[b]	-,000269	0[b]
[Condition=Sham]	-,000269	,000526	0[b]	,000269	0[b]
[Condition=taVNS]	0[b]	0[b]	0[b]	0[b]	0[b]
[Time=T0]	-,000269	,000269	0[b]	,000538	0[b]
[Time=T2]	0[b]	0[b]	0[b]	0[b]	0[b]
[Condition=Sham] * [Time=T0]	,000269	-,000526	0[b]	-,000538	0[b]
[Condition=Sham] * [Time=T2]	0[b]	0[b]	0[b]	0[b]	0[b]
[Condition=taVNS] * [Time=T0]	0[b]	0[b]	0[b]	0[b]	0[b]
[Condition=taVNS] * [Time=T2]	0[b]	0[b]	0[b]	0[b]	0[b]

Matrice de covariance pour les estimations des effets fixes[a]

Paramètre	[Condition=Sham] * [Time=T0]	[Condition=Sham] * [Time=T2]	[Condition=taVNS] * [Time=T0]	[Condition=taVNS] * [Time=T2]
Constante	,000269	0[b]	0[b]	0[b]
[Condition=Sham]	-,000526	0[b]	0[b]	0[b]
[Condition=taVNS]	0[b]	0[b]	0[b]	0[b]
[Time=T0]	-,000538	0[b]	0[b]	0[b]
[Time=T2]	0[b]	0[b]	0[b]	0[b]
[Condition=Sham] * [Time=T0]	,001064	0[b]	0[b]	0[b]
[Condition=Sham] * [Time=T2]	0[b]	0[b]	0[b]	0[b]
[Condition=taVNS] * [Time=T0]	0[b]	0[b]	0[b]	0[b]
[Condition=taVNS] * [Time=T2]	0[b]	0[b]	0[b]	0[b]

a. Variable dépendante : Cool P2 latency.

b. La covariance est définie sur 0, car elle est associée à un paramètre redondant.

Paramètres de covariance

Estimations des paramètres de covariance[a]

Paramètre	Estimation	Erreur standard	Z de Wald	Sig.	Intervalle de confiance à 95 %	
					Borne inférieure	Borne supérieure
Résidu	,005648	,000887	6,364	,000	,004151	,007685

a. Variable dépendante : Cool P2 latency.

Matrice de corrélation pour les estimations des paramètres de covariance[a]

Paramètre	Résidu
Résidu	1

a. Variable dépendante : Cool P2 latency.

Matrice de covariance pour les estimations des paramètres de covariance[a]

Paramètre	Résidu
Résidu	7,876112E-7

a. Variable dépendante : Cool P2 latency.

Moyenne marginale estimée

1. Grand Mean[a]

Moyenne	Erreur standard	ddl	Intervalle de confiance à 95 %	
			Borne inférieure	Borne supérieure
,357	,008	81	,341	,373

a. Variable dépendante : Cool P2 latency.

2. Condition

Estimations[a]

Condition	Moyenne	Erreur standard	ddl	Intervalle de confiance à 95 %	
				Borne inférieure	Borne supérieure
Sham	,353	,011	81	,330	,375
taVNS	,361	,012	81	,338	,385

a. Variable dépendante : Cool P2 latency.

Comparaisons appariées[a]

(I) Condition	(J) Condition	Différence moyenne (I-J)	Erreur standard	ddl	Sig.[b]
Sham	taVNS	-,009	,016	81	,588
taVNS	Sham	,009	,016	81	,588

Comparaisons appariées[a]

(I) Condition	(J) Condition	Intervalle de confiance à 95 % pour la différence[b] Borne inférieure	Borne supérieure
Sham	taVNS	-,041	,024
taVNS	Sham	-,024	,041

Basées sur les moyennes marginales estimées[a]

a. Variable dépendante : Cool P2 latency.

b. Ajustement pour les comparaisons multiples : Bonferroni.

Tests univariés[a]

Ddl du numérateur	Ddl du dénominateur	F	Sig.
1	81	,295	,588

Le test de F permet de tester l'effet de Condition. Il s'appuie sur les comparaisons appariées (indépendantes) linéaires parmi les moyennes marginales estimées.[a]

a. Variable dépendante : Cool P2 latency.

3. Time

Estimations[a]

Time	Moyenne	Erreur standard	ddl	Intervalle de confiance à 95 % Borne inférieure	Borne supérieure
T0	,369	,012	81	,346	,392
T2	,345	,011	81	,322	,368

a. Variable dépendante : Cool P2 latency.

Comparaisons appariées[a]

(I) Time	(J) Time	Différence moyenne (I-J)	Erreur standard	ddl	Sig.[b]	Intervalle de confiance à 95 % pour la différence[b] Borne inférieure
T0	T2	,024	,016	81	,139	-,008
T2	T0	-,024	,016	81	,139	-,057

Comparaisons appariées[a]

(I) Time	(J) Time	Intervalle de confiance à 95 % pour la différence<br>Borne supérieure
T0	T2	,057
T2	T0	,008

Basées sur les moyennes marginales estimées[a]

a. Variable dépendante : Cool P2 latency.

b. Ajustement pour les comparaisons multiples : Bonferroni.

Tests univariés[a]

Ddl du numérateur	Ddl du dénominateur	F	Sig.
1	81	2,236	,139

Le test de F permet de tester l'effet de Time. Il s'appuie sur les comparaisons appariées (indépendantes) linéaires parmi les moyennes marginales estimées.[a]

a. Variable dépendante : Cool P2 latency.

4. Condition * Time[a]

Condition	Time	Moyenne	Erreur standard	ddl	Intervalle de confiance à 95 %<br>Borne inférieure	 Borne supérieure
Sham	T0	,371	,016	81	,338	,404
	T2	,334	,016	81	,302	,366
taVNS	T0	,368	,016	81	,335	,400
	T2	,355	,016	81	,323	,388

a. Variable dépendante : Cool P2 latency.

2. Experiment 2

2.1. Laser-evoked potentials (LEPs)

2.1.1 LEPs N2P2 Amplitude.

```
MIXED LaserN2P2Amplitude BY Condition Phase
  /CRITERIA=CIN(95) MXITER(100) MXSTEP(10) SCORING(1)
SINGULAR(0.000000000001) HCONVERGE(0,
    ABSOLUTE) LCONVERGE(0, ABSOLUTE) PCONVERGE(0.000001, ABSOLUTE)
  /FIXED=Condition Phase Condition*Phase | SSTYPE(3)
  /METHOD=REML
  /PRINT=CPS CORB COVB DESCRIPTIVES G  SOLUTION TESTCOV
  /EMMEANS=TABLES(OVERALL)
  /EMMEANS=TABLES(Condition) COMPARE ADJ(BONFERRONI)
  /EMMEANS=TABLES(Phase) COMPARE ADJ(BONFERRONI)
  /EMMEANS=TABLES(Condition*Phase) .
```

Remarques

Sortie obtenue		05-MAY-2021 12:26:24
Commentaires		
Entrée	Jeu de données actif	Jeu_de_données2
	Filtre	<sans>
	Pondération	<sans>
	Fichier scindé	<sans>
	N de lignes dans le fichier de travail	60
Gestion des valeurs manquantes	Définition de la valeur manquante	Les valeurs manquantes définies par l'utilisateur sont traitées comme étant manquantes.
	Observations utilisées	Les statistiques sont basées sur toutes les observations comportant des données valides pour toutes les variables du modèle.

Syntaxe		MIXED LaserN2P2Amplitude BY Condition Phase /CRITERIA=CIN(95) MXITER(100) MXSTEP(10) SCORING(1) SINGULAR(0.000000000001) HCONVERGE(0, ABSOLUTE) LCONVERGE(0, ABSOLUTE) PCONVERGE(0.000001, ABSOLUTE) /FIXED=Condition Phase Condition*Phase | SSTYPE(3) /METHOD=REML /PRINT=CPS CORB COVB DESCRIPTIVES G SOLUTION TESTCOV /EMMEANS=TABLES(OVERALL) /EMMEANS=TABLES(Condition) COMPARE ADJ(BONFERRONI) /EMMEANS=TABLES(Phase) COMPARE ADJ(BONFERRONI) /EMMEANS=TABLES(Condition*Phase) .
Ressources	Temps de processeur	00:00:00,02
	Temps écoulé	00:00:00,02

Récapitulatif de traitement des observations

		Effectif	Pourcentage marginal
Condition	Sham	30	50,0%
	taVNS	30	50,0%

Phase	OFF	30	50,0%
	ON	30	50,0%
Valide		60	100,0%
Exclues		0	
Total		60	

Statistiques descriptives

Laser N2P2 Amplitude

Condition	Phase	Effectif	Moyenne	Ecart type	Coefficient de variation
Sham	OFF	15	24,287506666666670	11,963986447099517	49,3%
	ON	15	24,259046666666674	12,600303798881011	51,9%
	Total	30	24,273276666666670	12,072583445222095	49,7%
taVNS	OFF	15	23,580073333333330	11,000338887862025	46,7%
	ON	15	21,356833333333327	11,121686059719200	52,1%
	Total	30	22,468453333333333	10,927444035238265	48,6%
Total	OFF	30	23,933790000000005	11,298116180122761	47,2%
	ON	30	22,807940000000006	11,770221205597185	51,6%
	Total	60	23,370865000000000	11,452476329314113	49,0%

Dimension du modèle[a]

		Nombre de niveaux	Nombre de paramètres
Effets fixes	Constante	1	1
	Condition	2	1
	Phase	2	1
	Condition * Phase	4	1
Résidu			1
Total		9	5

a. Variable dépendante : Laser N2P2 Amplitude.

Critères d'information[a]

Log de vraisemblance restreint -2	445,129
Critère d'information d'Akaike (AIC)	447,129
Critère de Hurvich et Tsai (AICC)	447,203
Critère de Bozdogan (CAIC)	450,155
Critère bayésien de Schwartz (BIC)	449,155

Les critères d'informations sont présentés en plus petit, disposant d'un meilleur format.[a]

a. Variable dépendante : Laser N2P2 Amplitude.

Effets fixes

Tests des effets fixes de type III[a]

Source	Ddl du numérateur	Ddl du dénominateur	F	Sig.
Constante	1	56	239,821	,000
Condition	1	56	,358	,552
Phase	1	56	,139	,711
Condition * Phase	1	56	,132	,718

a. Variable dépendante : Laser N2P2 Amplitude.

Estimations des effets fixes[a]

Paramètre	Estimation	Erreur standard	ddl	t	Sig.
Constante	21,356833	3,018289	56	7,076	,000
[Condition=Sham]	2,902213	4,268505	56	,680	,499
[Condition=taVNS]	0[b]	0	.	.	.
[Phase=OFF]	2,223240	4,268505	56	,521	,605
[Phase=ON]	0[b]	0	.	.	.
[Condition=Sham] * [Phase=OFF]	-2,194780	6,036577	56	-,364	,718
[Condition=Sham] * [Phase=ON]	0[b]	0	.	.	.
[Condition=taVNS] * [Phase=OFF]	0[b]	0	.	.	.

[Condition=taVNS] * [Phase=ON]	0[b]	0	.	.	.

Estimations des effets fixes[a]

Paramètre	Intervalle de confiance à 95 % Borne inférieure	Borne supérieure
Constante	15,310475	27,403192
[Condition=Sham]	-5,648629	11,453056
[Condition=taVNS]	.	.
[Phase=OFF]	-6,327603	10,774083
[Phase=ON]	.	.
[Condition=Sham] * [Phase=OFF]	-14,287497	9,897937
[Condition=Sham] * [Phase=ON]	.	.
[Condition=taVNS] * [Phase=OFF]	.	.
[Condition=taVNS] * [Phase=ON]	.	.

a. Variable dépendante : Laser N2P2 Amplitude.

b. Ce paramètre est défini sur 0, car il est redondant.

Matrice de corrélation pour les estimations des effets fixes[a]

Paramètre	Constante	[Condition=Sham]	[Condition=taVNS]	[Phase=OFF]
Constante	1	-,707	.[b]	-,707
[Condition=Sham]	-,707	1	.[b]	,500
[Condition=taVNS]	.[b]	.[b]	.[b]	.[b]
[Phase=OFF]	-,707	,500	.[b]	1
[Phase=ON]	.[b]	.[b]	.[b]	.[b]
[Condition=Sham] * [Phase=OFF]	,500	-,707	.[b]	-,707
[Condition=Sham] * [Phase=ON]	.[b]	.[b]	.[b]	.[b]
[Condition=taVNS] * [Phase=OFF]	.[b]	.[b]	.[b]	.[b]
[Condition=taVNS] * [Phase=ON]	.[b]	.[b]	.[b]	.[b]

Matrice de corrélation pour les estimations des effets fixes[a]

Paramètre	[Phase=ON]	[Condition=Sham] * [Phase=OFF]	[Condition=Sham] * [Phase=ON]	[Condition=taVNS] * [Phase=OFF]
Constante	.[b]	,500	.[b]	.[b]
[Condition=Sham]	.[b]	-,707	.[b]	.[b]
[Condition=taVNS]	.[b]	.[b]	.[b]	.[b]

[Phase=OFF]	.[b]	-,707	.[b]	.[b]
[Phase=ON]	.[b]	.[b]	.[b]	.[b]
[Condition=Sham] * [Phase=OFF]	.[b]	1	.[b]	.[b]
[Condition=Sham] * [Phase=ON]	.[b]	.[b]	.[b]	.[b]
[Condition=taVNS] * [Phase=OFF]	.[b]	.[b]	.[b]	.[b]
[Condition=taVNS] * [Phase=ON]	.[b]	.[b]	.[b]	.[b]

Matrice de corrélation pour les estimations des effets fixes[a]

Paramètre	[Condition=taVNS] * [Phase=ON]
Constante	.[b]
[Condition=Sham]	.[b]
[Condition=taVNS]	.[b]
[Phase=OFF]	.[b]
[Phase=ON]	.[b]
[Condition=Sham] * [Phase=OFF]	.[b]
[Condition=Sham] * [Phase=ON]	.[b]
[Condition=taVNS] * [Phase=OFF]	.[b]
[Condition=taVNS] * [Phase=ON]	.[b]

a. Variable dépendante : Laser N2P2 Amplitude.

b. La corrélation est manquante par défaut, car elle est associée à un paramètre redondant.

Matrice de covariance pour les estimations des effets fixes[a]

Paramètre	Constante	[Condition=Sham]	[Condition=taVNS]	[Phase=OFF]
Constante	9,110066	-9,110066	0[b]	-9,110066
[Condition=Sham]	-9,110066	18,220133	0[b]	9,110066
[Condition=taVNS]	0[b]	0[b]	0[b]	0[b]
[Phase=OFF]	-9,110066	9,110066	0[b]	18,220133
[Phase=ON]	0[b]	0[b]	0[b]	0[b]
[Condition=Sham] * [Phase=OFF]	9,110066	-18,220133	0[b]	-18,220133
[Condition=Sham] * [Phase=ON]	0[b]	0[b]	0[b]	0[b]
[Condition=taVNS] * [Phase=OFF]	0[b]	0[b]	0[b]	0[b]
[Condition=taVNS] * [Phase=ON]	0[b]	0[b]	0[b]	0[b]

Matrice de covariance pour les estimations des effets fixes[a]

Paramètre	[Phase=ON]	[Condition=Sham] * [Phase=OFF]	[Condition=Sham] * [Phase=ON]	[Condition=taVNS] * [Phase=OFF]
Constante	0[b]	9,110066	0[b]	0[b]
[Condition=Sham]	0[b]	-18,220133	0[b]	0[b]
[Condition=taVNS]	0[b]	0[b]	0[b]	0[b]
[Phase=OFF]	0[b]	-18,220133	0[b]	0[b]
[Phase=ON]	0[b]	0[b]	0[b]	0[b]
[Condition=Sham] * [Phase=OFF]	0[b]	36,440266	0[b]	0[b]
[Condition=Sham] * [Phase=ON]	0[b]	0[b]	0[b]	0[b]
[Condition=taVNS] * [Phase=OFF]	0[b]	0[b]	0[b]	0[b]
[Condition=taVNS] * [Phase=ON]	0[b]	0[b]	0[b]	0[b]

Matrice de covariance pour les estimations des effets fixes[a]

Paramètre	[Condition=taVNS] * [Phase=ON]
Constante	0[b]
[Condition=Sham]	0[b]
[Condition=taVNS]	0[b]
[Phase=OFF]	0[b]
[Phase=ON]	0[b]
[Condition=Sham] * [Phase=OFF]	0[b]
[Condition=Sham] * [Phase=ON]	0[b]
[Condition=taVNS] * [Phase=OFF]	0[b]
[Condition=taVNS] * [Phase=ON]	0[b]

a. Variable dépendante : Laser N2P2 Amplitude.

b. La covariance est définie sur 0, car elle est associée à un paramètre redondant.

Paramètres de covariance

Estimations des paramètres de covariance[a]

Paramètre	Estimation	Erreur standard	Z de Wald	Sig.	Intervalle de confiance à 95 % Borne inférieure	Borne supérieure
Résidu	136,650996	25,824611	5,292	,000	94,351939	197,913205

a. Variable dépendante : Laser N2P2 Amplitude.

Matrice de corrélation pour les estimations des paramètres de covariance[a]

Paramètre	Résidu
Résidu	1

a. Variable dépendante : Laser N2P2 Amplitude.

Matrice de covariance pour les estimations des paramètres de covariance[a]

Paramètre	Résidu
Résidu	666,910525

a. Variable dépendante : Laser N2P2 Amplitude.

Moyenne marginale estimée

1. Grand Mean[a]

Moyenne	Erreur standard	ddl	Intervalle de confiance à 95 % Borne inférieure	Intervalle de confiance à 95 % Borne supérieure
23,371	1,509	56	20,348	26,394

a. Variable dépendante : Laser N2P2 Amplitude.

2. Condition

Estimations[a]

Condition	Moyenne	Erreur standard	ddl	Intervalle de confiance à 95 % Borne inférieure	Intervalle de confiance à 95 % Borne supérieure
Sham	24,273	2,134	56	19,998	28,549
taVNS	22,468	2,134	56	18,193	26,744

a. Variable dépendante : Laser N2P2 Amplitude.

Comparaisons appariées[a]

(I) Condition	(J) Condition	Différence moyenne (I-J)	Erreur standard	ddl	Sig.[b]
Sham	taVNS	1,805	3,018	56	,552
taVNS	Sham	-1,805	3,018	56	,552

Comparaisons appariées[a]

		Intervalle de confiance à 95 % pour la différence[b]	
(I) Condition	(J) Condition	Borne inférieure	Borne supérieure
Sham	taVNS	-4,242	7,851
taVNS	Sham	-7,851	4,242

Basées sur les moyennes marginales estimées[a]

a. Variable dépendante : Laser N2P2 Amplitude.

b. Ajustement pour les comparaisons multiples : Bonferroni.

Tests univariés[a]

Ddl du numérateur	Ddl du dénominateur	F	Sig.
1	56	,358	,552

Le test de F permet de tester l'effet de Condition. Il s'appuie sur les comparaisons appariées (indépendantes) linéaires parmi les moyennes marginales estimées.[a]

a. Variable dépendante : Laser N2P2 Amplitude.

3. Phase

Estimations[a]

				Intervalle de confiance à 95 %	
Phase	Moyenne	Erreur standard	ddl	Borne inférieure	Borne supérieure
OFF	23,934	2,134	56	19,658	28,209
ON	22,808	2,134	56	18,533	27,083

a. Variable dépendante : Laser N2P2 Amplitude.

Comparaisons appariées[a]

(I) Phase	(J) Phase	Différence moyenne (I-J)	Erreur standard	ddl	Sig.[b]	Intervalle de confiance à 95 % pour la différence[b] Borne inférieure
OFF	ON	1,126	3,018	56	,711	-4,921
ON	OFF	-1,126	3,018	56	,711	-7,172

Comparaisons appariées[a]

(I) Phase	(J) Phase	Intervalle de confiance à 95 % pour la différence
		Borne supérieure
OFF	ON	7,172
ON	OFF	4,921

Basées sur les moyennes marginales estimées[a]

a. Variable dépendante : Laser N2P2 Amplitude.

b. Ajustement pour les comparaisons multiples : Bonferroni.

Tests univariés[a]

Ddl du numérateur	Ddl du dénominateur	F	Sig.
1	56	,139	,711

Le test de F permet de tester l'effet de Phase. Il s'appuie sur les comparaisons appariées (indépendantes) linéaires parmi les moyennes marginales estimées.[a]

a. Variable dépendante : Laser N2P2 Amplitude.

4. Condition * Phase[a]

Condition	Phase	Moyenne	Erreur standard	ddl	Intervalle de confiance à 95 %	
					Borne inférieure	Borne supérieure
Sham	OFF	24,288	3,018	56	18,241	30,334
	ON	24,259	3,018	56	18,213	30,305
taVNS	OFF	23,580	3,018	56	17,534	29,626
	ON	21,357	3,018	56	15,310	27,403

a. Variable dépendante : Laser N2P2 Amplitude.

2.1.2 LEPs N2 Amplitude.

```
MIXED LaserN2Amplitude BY Condition Phase
  /CRITERIA=CIN(95) MXITER(100) MXSTEP(10) SCORING(1)
SINGULAR(0.000000000001) HCONVERGE(0,
    ABSOLUTE) LCONVERGE(0, ABSOLUTE) PCONVERGE(0.000001, ABSOLUTE)
  /FIXED=Condition Phase Condition*Phase | SSTYPE(3)
  /METHOD=REML
  /PRINT=CPS CORB COVB DESCRIPTIVES G  SOLUTION TESTCOV
  /EMMEANS=TABLES(OVERALL)
  /EMMEANS=TABLES(Condition) COMPARE ADJ(BONFERRONI)
  /EMMEANS=TABLES(Phase) COMPARE ADJ(BONFERRONI)
  /EMMEANS=TABLES(Condition*Phase) .
```

Remarques

Sortie obtenue		05-MAY-2021 12:27:32
Commentaires		
Entrée	Jeu de données actif	Jeu_de_données2
	Filtre	<sans>
	Pondération	<sans>
	Fichier scindé	<sans>
	N de lignes dans le fichier de travail	60
Gestion des valeurs manquantes	Définition de la valeur manquante	Les valeurs manquantes définies par l'utilisateur sont traitées comme étant manquantes.
	Observations utilisées	Les statistiques sont basées sur toutes les observations comportant des données valides pour toutes les variables du modèle.

Syntaxe		MIXED LaserN2Amplitude BY Condition Phase<br>/CRITERIA=CIN(95) MXITER(100) MXSTEP(10) SCORING(1) SINGULAR(0.000000000001) HCONVERGE(0, ABSOLUTE) LCONVERGE(0, ABSOLUTE) PCONVERGE(0.000001, ABSOLUTE)<br>/FIXED=Condition Phase Condition*Phase | SSTYPE(3)<br>/METHOD=REML<br>/PRINT=CPS CORB COVB DESCRIPTIVES G SOLUTION TESTCOV<br>/EMMEANS=TABLES(OVERALL)<br>/EMMEANS=TABLES(Condition) COMPARE ADJ(BONFERRONI)<br>/EMMEANS=TABLES(Phase) COMPARE ADJ(BONFERRONI)<br>/EMMEANS=TABLES(Condition*Phase) .
Ressources	Temps de processeur	00:00:00,02
	Temps écoulé	00:00:00,02

Récapitulatif de traitement des observations

		Effectif	Pourcentage marginal
Condition	Sham	30	50,0%
	taVNS	30	50,0%

Phase	OFF	30	50,0%
	ON	30	50,0%
Valide		60	100,0%
Exclues		0	
Total		60	

Statistiques descriptives

Laser N2 Amplitude

Condition	Phase	Effectif	Moyenne	Ecart type	Coefficient de variation
Sham	OFF	15	-12,03	6,266	-52,1%
	ON	15	-12,68	7,349	-58,0%
	Total	30	-12,35	6,718	-54,4%
taVNS	OFF	15	-13,85	8,409	-60,7%
	ON	15	-12,61	9,768	-77,4%
	Total	30	-13,23	8,977	-67,8%
Total	OFF	30	-12,94	7,345	-56,8%
	ON	30	-12,65	8,493	-67,2%
	Total	60	-12,79	7,874	-61,5%

Dimension du modèle[a]

		Nombre de niveaux	Nombre de paramètres
Effets fixes	Constante	1	1
	Condition	2	1
	Phase	2	1
	Condition * Phase	4	1
Résidu			1
Total		9	5

a. Variable dépendante : Laser N2 Amplitude.

Critères d'information[a]

Log de vraisemblance restreint -2	403,386
Critère d'information d'Akaike (AIC)	405,386
Critère de Hurvich et Tsai (AICC)	405,460
Critère de Bozdogan (CAIC)	408,412

Critère bayésien de Schwartz (BIC)	407,412

Les critères d'informations sont présentés en plus petit, disposant d'un meilleur format.[a]

a. Variable dépendante : Laser N2 Amplitude.

Effets fixes

Tests des effets fixes de type III[a]

Source	Ddl du numérateur	Ddl du dénominateur	F	Sig.
Constante	1	56	151,446	,000
Condition	1	56	,179	,674
Phase	1	56	,020	,887
Condition * Phase	1	56	,206	,652

a. Variable dépendante : Laser N2 Amplitude.

Estimations des effets fixes[a]

Paramètre	Estimation	Erreur standard	ddl	t	Sig.
Constante	-12,613360	2,079202	56	-6,066	,000
[Condition=Sham]	-,064820	2,940435	56	-,022	,982
[Condition=taVNS]	0[b]	0	.	.	.
[Phase=OFF]	-1,239573	2,940435	56	-,422	,675
[Phase=ON]	0[b]	0	.	.	.
[Condition=Sham] * [Phase=OFF]	1,887553	4,158404	56	,454	,652
[Condition=Sham] * [Phase=ON]	0[b]	0	.	.	.
[Condition=taVNS] * [Phase=OFF]	0[b]	0	.	.	.
[Condition=taVNS] * [Phase=ON]	0[b]	0	.	.	.

Estimations des effets fixes[a]

Paramètre	Intervalle de confiance à 95 % Borne inférieure	Borne supérieure
Constante	-16,778502	-8,448218
[Condition=Sham]	-5,955220	5,825580

[Condition=taVNS]	.	.
[Phase=OFF]	-7,129973	4,650827
[Phase=ON]	.	.
[Condition=Sham] * [Phase=OFF]	-6,442730	10,217837
[Condition=Sham] * [Phase=ON]	.	.
[Condition=taVNS] * [Phase=OFF]	.	.
[Condition=taVNS] * [Phase=ON]	.	.

a. Variable dépendante : Laser N2 Amplitude.

b. Ce paramètre est défini sur 0, car il est redondant.

Matrice de corrélation pour les estimations des effets fixes[a]

Paramètre	Constante	[Condition=Sham]	[Condition=taVNS]	[Phase=OFF]
Constante	1	-,707	.[b]	-,707
[Condition=Sham]	-,707	1	.[b]	,500
[Condition=taVNS]	.[b]	.[b]	.[b]	.[b]
[Phase=OFF]	-,707	,500	.[b]	1
[Phase=ON]	.[b]	.[b]	.[b]	.[b]
[Condition=Sham] * [Phase=OFF]	,500	-,707	.[b]	-,707
[Condition=Sham] * [Phase=ON]	.[b]	.[b]	.[b]	.[b]
[Condition=taVNS] * [Phase=OFF]	.[b]	.[b]	.[b]	.[b]
[Condition=taVNS] * [Phase=ON]	.[b]	.[b]	.[b]	.[b]

Matrice de corrélation pour les estimations des effets fixes[a]

Paramètre	[Phase=ON]	[Condition=Sham] * [Phase=OFF]	[Condition=Sham] * [Phase=ON]	[Condition=taVNS] * [Phase=OFF]
Constante	.[b]	,500	.[b]	.[b]
[Condition=Sham]	.[b]	-,707	.[b]	.[b]
[Condition=taVNS]	.[b]	.[b]	.[b]	.[b]
[Phase=OFF]	.[b]	-,707	.[b]	.[b]
[Phase=ON]	.[b]	.[b]	.[b]	.[b]
[Condition=Sham] * [Phase=OFF]	.[b]	1	.[b]	.[b]
[Condition=Sham] * [Phase=ON]	.[b]	.[b]	.[b]	.[b]
[Condition=taVNS] * [Phase=OFF]	.[b]	.[b]	.[b]	.[b]

[Condition=taVNS] * [Phase=ON]	.[b]	.[b]	.[b]	.[b]

Matrice de corrélation pour les estimations des effets fixes[a]

Paramètre	[Condition=taVNS] * [Phase=ON]
Constante	.[b]
[Condition=Sham]	.[b]
[Condition=taVNS]	.[b]
[Phase=OFF]	.[b]
[Phase=ON]	.[b]
[Condition=Sham] * [Phase=OFF]	.[b]
[Condition=Sham] * [Phase=ON]	.[b]
[Condition=taVNS] * [Phase=OFF]	.[b]
[Condition=taVNS] * [Phase=ON]	.[b]

a. Variable dépendante : Laser N2 Amplitude.

b. La corrélation est manquante par défaut, car elle est associée à un paramètre redondant.

Matrice de covariance pour les estimations des effets fixes[a]

Paramètre	Constante	[Condition=Sham]	[Condition=taVNS]	[Phase=OFF]
Constante	4,323080	-4,323080	0[b]	-4,323080
[Condition=Sham]	-4,323080	8,646160	0[b]	4,323080
[Condition=taVNS]	0[b]	0[b]	0[b]	0[b]
[Phase=OFF]	-4,323080	4,323080	0[b]	8,646160
[Phase=ON]	0[b]	0[b]	0[b]	0[b]
[Condition=Sham] * [Phase=OFF]	4,323080	-8,646160	0[b]	-8,646160
[Condition=Sham] * [Phase=ON]	0[b]	0[b]	0[b]	0[b]
[Condition=taVNS] * [Phase=OFF]	0[b]	0[b]	0[b]	0[b]
[Condition=taVNS] * [Phase=ON]	0[b]	0[b]	0[b]	0[b]

Matrice de covariance pour les estimations des effets fixes[a]

Paramètre	[Phase=ON]	[Condition=Sham] * [Phase=OFF]	[Condition=Sham] * [Phase=ON]	[Condition=taVNS] * [Phase=OFF]
Constante	0[b]	4,323080	0[b]	0[b]
[Condition=Sham]	0[b]	-8,646160	0[b]	0[b]
[Condition=taVNS]	0[b]	0[b]	0[b]	0[b]
[Phase=OFF]	0[b]	-8,646160	0[b]	0[b]

[Phase=ON]	0[b]	0[b]	0[b]	0[b]
[Condition=Sham] * [Phase=OFF]	0[b]	17,292320	0[b]	0[b]
[Condition=Sham] * [Phase=ON]	0[b]	0[b]	0[b]	0[b]
[Condition=taVNS] * [Phase=OFF]	0[b]	0[b]	0[b]	0[b]
[Condition=taVNS] * [Phase=ON]	0[b]	0[b]	0[b]	0[b]

Matrice de covariance pour les estimations des effets fixes[a]

Paramètre	[Condition=taVNS] * [Phase=ON]
Constante	0[b]
[Condition=Sham]	0[b]
[Condition=taVNS]	0[b]
[Phase=OFF]	0[b]
[Phase=ON]	0[b]
[Condition=Sham] * [Phase=OFF]	0[b]
[Condition=Sham] * [Phase=ON]	0[b]
[Condition=taVNS] * [Phase=OFF]	0[b]
[Condition=taVNS] * [Phase=ON]	0[b]

a. Variable dépendante : Laser N2 Amplitude.

b. La covariance est définie sur 0, car elle est associée à un paramètre redondant.

Paramètres de covariance

Estimations des paramètres de covariance[a]

Paramètre	Estimation	Erreur standard	Z de Wald	Sig.	Intervalle de confiance à 95 % Borne inférieure	Borne supérieure
Résidu	64,846201	12,254780	5,292	,000	44,773657	93,917497

a. Variable dépendante : Laser N2 Amplitude.

Matrice de corrélation pour les estimations des paramètres de covariance[a]

Paramètre	Résidu
Résidu	1

a. Variable dépendante :
Laser N2 Amplitude.

Matrice de covariance pour les estimations des paramètres de covariance[a]

Paramètre	Résidu
Résidu	150,179634

a. Variable dépendante :
Laser N2 Amplitude.

Moyenne marginale estimée

1. Grand Mean[a]

Moyenne	Erreur standard	ddl	Intervalle de confiance à 95 % Borne inférieure	Intervalle de confiance à 95 % Borne supérieure
-12,794	1,040	56	-14,876	-10,711

a. Variable dépendante : Laser N2 Amplitude.

2. Condition

Estimations[a]

Condition	Moyenne	Erreur standard	ddl	Intervalle de confiance à 95 % Borne inférieure	Intervalle de confiance à 95 % Borne supérieure
Sham	-12,354	1,470	56	-15,299	-9,409
taVNS	-13,233	1,470	56	-16,178	-10,288

a. Variable dépendante : Laser N2 Amplitude.

Comparaisons appariées[a]

(I) Condition	(J) Condition	Différence moyenne (I-J)	Erreur standard	ddl	Sig.[b]
Sham	taVNS	,879	2,079	56	,674
taVNS	Sham	-,879	2,079	56	,674

Comparaisons appariées[a]

(I) Condition	(J) Condition	Intervalle de confiance à 95 % pour la différence[b] Borne inférieure	Intervalle de confiance à 95 % pour la différence[b] Borne supérieure
Sham	taVNS	-3,286	5,044
taVNS	Sham	-5,044	3,286

Basées sur les moyennes marginales estimées[a]
a. Variable dépendante : Laser N2 Amplitude.
b. Ajustement pour les comparaisons multiples : Bonferroni.

Tests univariés[a]

Ddl du numérateur	Ddl du dénominateur	F	Sig.
1	56	,179	,674

Le test de F permet de tester l'effet de Condition. Il s'appuie sur les comparaisons appariées (indépendantes) linéaires parmi les moyennes marginales estimées.[a]
a. Variable dépendante : Laser N2 Amplitude.

3. Phase

Estimations[a]

Phase	Moyenne	Erreur standard	ddl	Intervalle de confiance à 95 % Borne inférieure	Intervalle de confiance à 95 % Borne supérieure
OFF	-12,942	1,470	56	-15,887	-9,996
ON	-12,646	1,470	56	-15,591	-9,701

a. Variable dépendante : Laser N2 Amplitude.

Comparaisons appariées[a]

(I) Phase	(J) Phase	Différence moyenne (I-J)	Erreur standard	ddl	Sig.[b]	Intervalle de confiance à 95 % pour la différence[b] Borne inférieure
OFF	ON	-,296	2,079	56	,887	-4,461
ON	OFF	,296	2,079	56	,887	-3,869

Comparaisons appariées[a]

(I) Phase	(J) Phase	Intervalle de confiance à 95 % pour la différence Borne supérieure
OFF	ON	3,869
ON	OFF	4,461

Basées sur les moyennes marginales estimées[a]
a. Variable dépendante : Laser N2 Amplitude.
b. Ajustement pour les comparaisons multiples : Bonferroni.

Tests univariés[a]

Ddl du numérateur	Ddl du dénominateur	F	Sig.
1	56	,020	,887

Le test de F permet de tester l'effet de Phase. Il s'appuie sur les comparaisons appariées (indépendantes) linéaires parmi les moyennes marginales estimées.[a]

a. Variable dépendante : Laser N2 Amplitude.

4. Condition * Phase[a]

Condition	Phase	Moyenne	Erreur standard	ddl	Intervalle de confiance à 95 % Borne inférieure	Borne supérieure
Sham	OFF	-12,030	2,079	56	-16,195	-7,865
	ON	-12,678	2,079	56	-16,843	-8,513
taVNS	OFF	-13,853	2,079	56	-18,018	-9,688
	ON	-12,613	2,079	56	-16,779	-8,448

a. Variable dépendante : Laser N2 Amplitude.

2.1.3. LEPs N2 Latency.

```
MIXED LaserN2Latency BY Condition Phase
  /CRITERIA=CIN(95) MXITER(100) MXSTEP(10) SCORING(1)
SINGULAR(0.000000000001) HCONVERGE(0,
    ABSOLUTE) LCONVERGE(0, ABSOLUTE) PCONVERGE(0.000001, ABSOLUTE)
  /FIXED=Condition Phase Condition*Phase | SSTYPE(3)
  /METHOD=REML
  /PRINT=CPS CORB COVB DESCRIPTIVES G  SOLUTION TESTCOV
  /EMMEANS=TABLES(OVERALL)
  /EMMEANS=TABLES(Condition) COMPARE ADJ(BONFERRONI)
  /EMMEANS=TABLES(Phase) COMPARE ADJ(BONFERRONI)
  /EMMEANS=TABLES(Condition*Phase) .
```

Remarques

Sortie obtenue		05-MAY-2021 12:28:13
Commentaires		
Entrée	Jeu de données actif	Jeu_de_données2
	Filtre	<sans>
	Pondération	<sans>
	Fichier scindé	<sans>
	N de lignes dans le fichier de travail	60

Gestion des valeurs manquantes	Définition de la valeur manquante	Les valeurs manquantes définies par l'utilisateur sont traitées comme étant manquantes.
	Observations utilisées	Les statistiques sont basées sur toutes les observations comportant des données valides pour toutes les variables du modèle.
Syntaxe		MIXED LaserN2Latency BY Condition Phase /CRITERIA=CIN(95) MXITER(100) MXSTEP(10) SCORING(1) SINGULAR(0.000000000001) HCONVERGE(0, ABSOLUTE) LCONVERGE(0, ABSOLUTE) PCONVERGE(0.000001, ABSOLUTE) /FIXED=Condition Phase Condition*Phase | SSTYPE(3) /METHOD=REML /PRINT=CPS CORB COVB DESCRIPTIVES G SOLUTION TESTCOV /EMMEANS=TABLES(OVERALL) /EMMEANS=TABLES(Condition) COMPARE ADJ(BONFERRONI) /EMMEANS=TABLES(Phase) COMPARE ADJ(BONFERRONI) /EMMEANS=TABLES(Condition*Phase) .
Ressources	Temps de processeur	00:00:00,00

Temps écoulé	00:00:00,02

Récapitulatif de traitement des observations

		Effectif	Pourcentage marginal
Condition	Sham	30	50,0%
	taVNS	30	50,0%
Phase	OFF	30	50,0%
	ON	30	50,0%
Valide		60	100,0%
Exclues		0	
Total		60	

Statistiques descriptives

Laser N2 Latency

Condition	Phase	Effectif	Moyenne	Ecart type	Coefficient de variation
Sham	OFF	15	,23	,032	14,1%
	ON	15	,23	,036	15,9%
	Total	30	,23	,034	14,8%
taVNS	OFF	15	,24	,023	9,4%
	ON	15	,23	,024	10,3%
	Total	30	,24	,024	9,9%
Total	OFF	30	,24	,028	12,0%
	ON	30	,23	,030	13,2%
	Total	60	,23	,029	12,6%

Dimension du modèle[a]

		Nombre de niveaux	Nombre de paramètres
Effets fixes	Constante	1	1
	Condition	2	1
	Phase	2	1
	Condition * Phase	4	1
Résidu			1
Total		9	5

a. Variable dépendante : Laser N2 Latency .

Critères d'information[a]

Log de vraisemblance restreint -2	-225,533
Critère d'information d'Akaike (AIC)	-223,533
Critère de Hurvich et Tsai (AICC)	-223,459
Critère de Bozdogan (CAIC)	-220,508
Critère bayésien de Schwartz (BIC)	-221,508

Les critères d'informations sont présentés en plus petit, disposant d'un meilleur format.[a]

a. Variable dépendante : Laser N2 Latency

.

Effets fixes

Tests des effets fixes de type III[a]

Source	Ddl du numérateur	Ddl du dénominateur	F	Sig.
Constante	1	56	3801,133	,000
Condition	1	56	1,958	,167
Phase	1	56	,938	,337
Condition * Phase	1	56	,084	,772

a. Variable dépendante : Laser N2 Latency .

Estimations des effets fixes[a]

Paramètre	Estimation	Erreur standard	ddl	t	Sig.
Constante	,233933	,007571	56	30,897	,000
[Condition=Sham]	-,008395	,010708	56	-,784	,436
[Condition=taVNS]	0[b]	0	.	.	.
[Phase=OFF]	,009533	,010708	56	,890	,377
[Phase=ON]	0[b]	0	.	.	.
[Condition=Sham] * [Phase=OFF]	-,004400	,015143	56	-,291	,772
[Condition=Sham] * [Phase=ON]	0[b]	0	.	.	.
[Condition=taVNS] * [Phase=OFF]	0[b]	0	.	.	.

[Condition=taVNS] * [Phase=ON]	0[b]	0	.	.	.

Estimations des effets fixes[a]

Paramètre	Intervalle de confiance à 95 % Borne inférieure	Borne supérieure
Constante	,218766	,249101
[Condition=Sham]	-,029845	,013055
[Condition=taVNS]	.	.
[Phase=OFF]	-,011917	,030983
[Phase=ON]	.	.
[Condition=Sham] * [Phase=OFF]	-,034735	,025935
[Condition=Sham] * [Phase=ON]	.	.
[Condition=taVNS] * [Phase=OFF]	.	.
[Condition=taVNS] * [Phase=ON]	.	.

a. Variable dépendante : Laser N2 Latency .

b. Ce paramètre est défini sur 0, car il est redondant.

Matrice de corrélation pour les estimations des effets fixes[a]

Paramètre	Constante	[Condition=Sham]	[Condition=taVNS]	[Phase=OFF]
Constante	1	-,707	.[b]	-,707
[Condition=Sham]	-,707	1	.[b]	,500
[Condition=taVNS]	.[b]	.[b]	.[b]	.[b]
[Phase=OFF]	-,707	,500	.[b]	1
[Phase=ON]	.[b]	.[b]	.[b]	.[b]
[Condition=Sham] * [Phase=OFF]	,500	-,707	.[b]	-,707
[Condition=Sham] * [Phase=ON]	.[b]	.[b]	.[b]	.[b]
[Condition=taVNS] * [Phase=OFF]	.[b]	.[b]	.[b]	.[b]
[Condition=taVNS] * [Phase=ON]	.[b]	.[b]	.[b]	.[b]

Matrice de corrélation pour les estimations des effets fixes[a]

Paramètre	[Phase=ON]	[Condition=Sham] * [Phase=OFF]	[Condition=Sham] * [Phase=ON]	[Condition=taVNS] * [Phase=OFF]
Constante	.[b]	,500	.[b]	.[b]
[Condition=Sham]	.[b]	-,707	.[b]	.[b]
[Condition=taVNS]	.[b]	.[b]	.[b]	.[b]

[Phase=OFF]	.[b]	-,707	.[b]	.[b]
[Phase=ON]	.[b]	.[b]	.[b]	.[b]
[Condition=Sham] * [Phase=OFF]	.[b]	1	.[b]	.[b]
[Condition=Sham] * [Phase=ON]	.[b]	.[b]	.[b]	.[b]
[Condition=taVNS] * [Phase=OFF]	.[b]	.[b]	.[b]	.[b]
[Condition=taVNS] * [Phase=ON]	.[b]	.[b]	.[b]	.[b]

Matrice de corrélation pour les estimations des effets fixes[a]

Paramètre	[Condition=taVNS] * [Phase=ON]
Constante	.[b]
[Condition=Sham]	.[b]
[Condition=taVNS]	.[b]
[Phase=OFF]	.[b]
[Phase=ON]	.[b]
[Condition=Sham] * [Phase=OFF]	.[b]
[Condition=Sham] * [Phase=ON]	.[b]
[Condition=taVNS] * [Phase=OFF]	.[b]
[Condition=taVNS] * [Phase=ON]	.[b]

a. Variable dépendante : Laser N2 Latency .

b. La corrélation est manquante par défaut, car elle est associée à un paramètre redondant.

Matrice de covariance pour les estimations des effets fixes[a]

Paramètre	Constante	[Condition=Sham]	[Condition=taVNS]	[Phase=OFF]
Constante	5,732676E-5	-5,732676E-5	0[b]	-5,732676E-5
[Condition=Sham]	-5,732676E-5	,000115	0[b]	5,732676E-5
[Condition=taVNS]	0[b]	0[b]	0[b]	0[b]
[Phase=OFF]	-5,732676E-5	5,732676E-5	0[b]	,000115
[Phase=ON]	0[b]	0[b]	0[b]	0[b]
[Condition=Sham] * [Phase=OFF]	5,732676E-5	-,000115	0[b]	-,000115
[Condition=Sham] * [Phase=ON]	0[b]	0[b]	0[b]	0[b]
[Condition=taVNS] * [Phase=OFF]	0[b]	0[b]	0[b]	0[b]
[Condition=taVNS] * [Phase=ON]	0[b]	0[b]	0[b]	0[b]

Matrice de covariance pour les estimations des effets fixes[a]

Paramètre	[Phase=ON]	[Condition=Sham] * [Phase=OFF]	[Condition=Sham] * [Phase=ON]	[Condition=taVNS] * [Phase=OFF]
Constante	0[b]	5,732676E-5	0[b]	0[b]
[Condition=Sham]	0[b]	-,000115	0[b]	0[b]
[Condition=taVNS]	0[b]	0[b]	0[b]	0[b]
[Phase=OFF]	0[b]	-,000115	0[b]	0[b]
[Phase=ON]	0[b]	0[b]	0[b]	0[b]
[Condition=Sham] * [Phase=OFF]	0[b]	,000229	0[b]	0[b]
[Condition=Sham] * [Phase=ON]	0[b]	0[b]	0[b]	0[b]
[Condition=taVNS] * [Phase=OFF]	0[b]	0[b]	0[b]	0[b]
[Condition=taVNS] * [Phase=ON]	0[b]	0[b]	0[b]	0[b]

Matrice de covariance pour les estimations des effets fixes[a]

Paramètre	[Condition=taVNS] * [Phase=ON]
Constante	0[b]
[Condition=Sham]	0[b]
[Condition=taVNS]	0[b]
[Phase=OFF]	0[b]
[Phase=ON]	0[b]
[Condition=Sham] * [Phase=OFF]	0[b]
[Condition=Sham] * [Phase=ON]	0[b]
[Condition=taVNS] * [Phase=OFF]	0[b]
[Condition=taVNS] * [Phase=ON]	0[b]

a. Variable dépendante : Laser N2 Latency .

b. La covariance est définie sur 0, car elle est associée à un paramètre redondant.

Paramètres de covariance

Estimations des paramètres de covariance[a]

Paramètre	Estimation	Erreur standard	Z de Wald	Sig.	Intervalle de confiance à 95 % Borne inférieure	Borne supérieure
Résidu	,000860	,000163	5,292	,000	,000594	,001245

a. Variable dépendante : Laser N2 Latency .

Matrice de corrélation pour les estimations des paramètres de covariance[a]

Paramètre	Résidu
Résidu	1

a. Variable dépendante : Laser N2 Latency .

Matrice de covariance pour les estimations des paramètres de covariance[a]

Paramètre	Résidu
Résidu	2,640822E-8

a. Variable dépendante : Laser N2 Latency .

Moyenne marginale estimée

1. Grand Mean[a]

Moyenne	Erreur standard	ddl	Intervalle de confiance à 95 %	
			Borne inférieure	Borne supérieure
,233	,004	56	,226	,241

a. Variable dépendante : Laser N2 Latency .

2. Condition

Estimations[a]

Condition	Moyenne	Erreur standard	ddl	Intervalle de confiance à 95 %	
				Borne inférieure	Borne supérieure
Sham	,228	,005	56	,217	,239
taVNS	,239	,005	56	,228	,249

a. Variable dépendante : Laser N2 Latency .

Comparaisons appariées[a]

(I) Condition	(J) Condition	Différence moyenne (I-J)	Erreur standard	ddl	Sig.[b]
Sham	taVNS	-,011	,008	56	,167
taVNS	Sham	,011	,008	56	,167

Comparaisons appariées[a]

		Intervalle de confiance à 95 % pour la différence[b]	
(I) Condition	(J) Condition	Borne inférieure	Borne supérieure
Sham	taVNS	-,026	,005
taVNS	Sham	-,005	,026

Basées sur les moyennes marginales estimées[a]

a. Variable dépendante : Laser N2 Latency .

b. Ajustement pour les comparaisons multiples : Bonferroni.

Tests univariés[a]

Ddl du numérateur	Ddl du dénominateur	F	Sig.
1	56	1,958	,167

Le test de F permet de tester l'effet de Condition. Il s'appuie sur les comparaisons appariées (indépendantes) linéaires parmi les moyennes marginales estimées.[a]

a. Variable dépendante : Laser N2 Latency .

3. Phase

Estimations[a]

				Intervalle de confiance à 95 %	
Phase	Moyenne	Erreur standard	ddl	Borne inférieure	Borne supérieure
OFF	,237	,005	56	,226	,248
ON	,230	,005	56	,219	,240

a. Variable dépendante : Laser N2 Latency .

Comparaisons appariées[a]

						Intervalle de confiance à 95 % pour la différence[b]
(I) Phase	(J) Phase	Différence moyenne (I-J)	Erreur standard	ddl	Sig.[b]	Borne inférieure
OFF	ON	,007	,008	56	,337	-,008
ON	OFF	-,007	,008	56	,337	-,023

Comparaisons appariées[a]

(I) Phase	(J) Phase	Intervalle de confiance à 95 % pour la différence<br>Borne supérieure
OFF	ON	,023
ON	OFF	,008

Basées sur les moyennes marginales estimées[a]

a. Variable dépendante : Laser N2 Latency .

b. Ajustement pour les comparaisons multiples : Bonferroni.

Tests univariés[a]

Ddl du numérateur	Ddl du dénominateur	F	Sig.
1	56	,938	,337

Le test de F permet de tester l'effet de Phase. Il s'appuie sur les comparaisons appariées (indépendantes) linéaires parmi les moyennes marginales estimées.[a]

a. Variable dépendante : Laser N2 Latency .

4. Condition * Phase[a]

Condition	Phase	Moyenne	Erreur standard	ddl	Intervalle de confiance à 95 %<br>Borne inférieure	 Borne supérieure
Sham	OFF	,231	,008	56	,216	,246
	ON	,226	,008	56	,210	,241
taVNS	OFF	,243	,008	56	,228	,259
	ON	,234	,008	56	,219	,249

a. Variable dépendante : Laser N2 Latency .

2.1.4. LEPs P2 Amplitude.

```
MIXED LaserP2Amplitude BY Condition Phase
  /CRITERIA=CIN(95) MXITER(100) MXSTEP(10) SCORING(1)
SINGULAR(0.000000000001) HCONVERGE(0,
    ABSOLUTE) LCONVERGE(0, ABSOLUTE) PCONVERGE(0.000001, ABSOLUTE)
  /FIXED=Condition Phase Condition*Phase | SSTYPE(3)
  /METHOD=REML
  /PRINT=CPS CORB COVB DESCRIPTIVES G  SOLUTION TESTCOV
  /EMMEANS=TABLES(OVERALL)
  /EMMEANS=TABLES(Condition) COMPARE ADJ(BONFERRONI)
  /EMMEANS=TABLES(Phase) COMPARE ADJ(BONFERRONI)
  /EMMEANS=TABLES(Condition*Phase) .
```

Remarques

Sortie obtenue		05-MAY-2021 12:28:41
Commentaires		
Entrée	Jeu de données actif	Jeu_de_données2
	Filtre	<sans>
	Pondération	<sans>
	Fichier scindé	<sans>
	N de lignes dans le fichier de travail	60
Gestion des valeurs manquantes	Définition de la valeur manquante	Les valeurs manquantes définies par l'utilisateur sont traitées comme étant manquantes.
	Observations utilisées	Les statistiques sont basées sur toutes les observations comportant des données valides pour toutes les variables du modèle.

Syntaxe		MIXED LaserP2Amplitude BY Condition Phase /CRITERIA=CIN(95) MXITER(100) MXSTEP(10) SCORING(1) SINGULAR(0.000000000001) HCONVERGE(0, ABSOLUTE) LCONVERGE(0, ABSOLUTE) PCONVERGE(0.000001, ABSOLUTE) /FIXED=Condition Phase Condition*Phase | SSTYPE(3) /METHOD=REML /PRINT=CPS CORB COVB DESCRIPTIVES G SOLUTION TESTCOV /EMMEANS=TABLES(OVERALL) /EMMEANS=TABLES(Condition) COMPARE ADJ(BONFERRONI) /EMMEANS=TABLES(Phase) COMPARE ADJ(BONFERRONI) /EMMEANS=TABLES(Condition*Phase) .
Ressources	Temps de processeur	00:00:00,02
	Temps écoulé	00:00:00,01

Récapitulatif de traitement des observations

		Effectif	Pourcentage marginal
Condition	Sham	30	50,0%
	taVNS	30	50,0%

Phase	OFF	30	50,0%
	ON	30	50,0%
Valide		60	100,0%
Exclues		0	
Total		60	

Statistiques descriptives

Laser P2 Amplitude

Condition	Phase	Effectif	Moyenne	Ecart type	Coefficient de variation
Sham	OFF	15	12,26	7,193	58,7%
	ON	15	11,58	6,911	59,7%
	Total	30	11,92	6,939	58,2%
taVNS	OFF	15	9,73	4,516	46,4%
	ON	15	8,74	6,314	72,2%
	Total	30	9,24	5,417	58,7%
Total	OFF	30	10,99	6,039	54,9%
	ON	30	10,16	6,662	65,6%
	Total	60	10,58	6,318	59,7%

Dimension du modèle[a]

		Nombre de niveaux	Nombre de paramètres
Effets fixes	Constante	1	1
	Condition	2	1
	Phase	2	1
	Condition * Phase	4	1
Résidu			1
Total		9	5

a. Variable dépendante : Laser P2 Amplitude.

Critères d'information[a]

Log de vraisemblance restreint -2	376,244
Critère d'information d'Akaike (AIC)	378,244
Critère de Hurvich et Tsai (AICC)	378,319
Critère de Bozdogan (CAIC)	381,270

Critère bayésien de Schwartz (BIC)	380,270

Les critères d'informations sont présentés en plus petit, disposant d'un meilleur format.[a]

a. Variable dépendante : Laser P2 Amplitude.

Effets fixes

Tests des effets fixes de type III[a]

Source	Ddl du numérateur	Ddl du dénominateur	F	Sig.
Constante	1	56	168,074	,000
Condition	1	56	2,705	,106
Phase	1	56	,259	,613
Condition * Phase	1	56	,009	,925

a. Variable dépendante : Laser P2 Amplitude.

Estimations des effets fixes[a]

Paramètre	Estimation	Erreur standard	ddl	t	Sig.
Constante	8,743473	1,631738	56	5,358	,000
[Condition=Sham]	2,837393	2,307627	56	1,230	,224
[Condition=taVNS]	0[b]	0	.	.	.
[Phase=OFF]	,983667	2,307627	56	,426	,672
[Phase=ON]	0[b]	0	.	.	.
[Condition=Sham] * [Phase=OFF]	-,307227	3,263477	56	-,094	,925
[Condition=Sham] * [Phase=ON]	0[b]	0	.	.	.
[Condition=taVNS] * [Phase=OFF]	0[b]	0	.	.	.
[Condition=taVNS] * [Phase=ON]	0[b]	0	.	.	.

Estimations des effets fixes[a]

Paramètre	Intervalle de confiance à 95 % Borne inférieure	Borne supérieure
Constante	5,474708	12,012238
[Condition=Sham]	-1,785338	7,460125

[Condition=taVNS]	.	.
[Phase=OFF]	-3,639065	5,606398
[Phase=ON]	.	.
[Condition=Sham] * [Phase=OFF]	-6,844756	6,230303
[Condition=Sham] * [Phase=ON]	.	.
[Condition=taVNS] * [Phase=OFF]	.	.
[Condition=taVNS] * [Phase=ON]	.	.

a. Variable dépendante : Laser P2 Amplitude.

b. Ce paramètre est défini sur 0, car il est redondant.

Matrice de corrélation pour les estimations des effets fixes[a]

Paramètre	Constante	[Condition=Sham]	[Condition=taVNS]	[Phase=OFF]
Constante	1	-,707	.[b]	-,707
[Condition=Sham]	-,707	1	.[b]	,500
[Condition=taVNS]	.[b]	.[b]	.[b]	.[b]
[Phase=OFF]	-,707	,500	.[b]	1
[Phase=ON]	.[b]	.[b]	.[b]	.[b]
[Condition=Sham] * [Phase=OFF]	,500	-,707	.[b]	-,707
[Condition=Sham] * [Phase=ON]	.[b]	.[b]	.[b]	.[b]
[Condition=taVNS] * [Phase=OFF]	.[b]	.[b]	.[b]	.[b]
[Condition=taVNS] * [Phase=ON]	.[b]	.[b]	.[b]	.[b]

Matrice de corrélation pour les estimations des effets fixes[a]

Paramètre	[Phase=ON]	[Condition=Sham] * [Phase=OFF]	[Condition=Sham] * [Phase=ON]	[Condition=taVNS] * [Phase=OFF]
Constante	.[b]	,500	.[b]	.[b]
[Condition=Sham]	.[b]	-,707	.[b]	.[b]
[Condition=taVNS]	.[b]	.[b]	.[b]	.[b]
[Phase=OFF]	.[b]	-,707	.[b]	.[b]
[Phase=ON]	.[b]	.[b]	.[b]	.[b]
[Condition=Sham] * [Phase=OFF]	.[b]	1	.[b]	.[b]
[Condition=Sham] * [Phase=ON]	.[b]	.[b]	.[b]	.[b]
[Condition=taVNS] * [Phase=OFF]	.[b]	.[b]	.[b]	.[b]

[Condition=taVNS] * [Phase=ON]	.[b]	.[b]	.[b]	.[b]

Matrice de corrélation pour les estimations des effets fixes[a]

Paramètre	[Condition=taVNS] * [Phase=ON]
Constante	.[b]
[Condition=Sham]	.[b]
[Condition=taVNS]	.[b]
[Phase=OFF]	.[b]
[Phase=ON]	.[b]
[Condition=Sham] * [Phase=OFF]	.[b]
[Condition=Sham] * [Phase=ON]	.[b]
[Condition=taVNS] * [Phase=OFF]	.[b]
[Condition=taVNS] * [Phase=ON]	.[b]

a. Variable dépendante : Laser P2 Amplitude.

b. La corrélation est manquante par défaut, car elle est associée à un paramètre redondant.

Matrice de covariance pour les estimations des effets fixes[a]

Paramètre	Constante	[Condition=Sham]	[Condition=taVNS]	[Phase=OFF]
Constante	2,662570	-2,662570	0[b]	-2,662570
[Condition=Sham]	-2,662570	5,325141	0[b]	2,662570
[Condition=taVNS]	0[b]	0[b]	0[b]	0[b]
[Phase=OFF]	-2,662570	2,662570	0[b]	5,325141
[Phase=ON]	0[b]	0[b]	0[b]	0[b]
[Condition=Sham] * [Phase=OFF]	2,662570	-5,325141	0[b]	-5,325141
[Condition=Sham] * [Phase=ON]	0[b]	0[b]	0[b]	0[b]
[Condition=taVNS] * [Phase=OFF]	0[b]	0[b]	0[b]	0[b]
[Condition=taVNS] * [Phase=ON]	0[b]	0[b]	0[b]	0[b]

Matrice de covariance pour les estimations des effets fixes[a]

Paramètre	[Phase=ON]	[Condition=Sham] * [Phase=OFF]	[Condition=Sham] * [Phase=ON]	[Condition=taVNS] * [Phase=OFF]
Constante	0[b]	2,662570	0[b]	0[b]
[Condition=Sham]	0[b]	-5,325141	0[b]	0[b]
[Condition=taVNS]	0[b]	0[b]	0[b]	0[b]
[Phase=OFF]	0[b]	-5,325141	0[b]	0[b]

[Phase=ON]	0[b]	0[b]	0[b]	0[b]
[Condition=Sham] * [Phase=OFF]	0[b]	10,650281	0[b]	0[b]
[Condition=Sham] * [Phase=ON]	0[b]	0[b]	0[b]	0[b]
[Condition=taVNS] * [Phase=OFF]	0[b]	0[b]	0[b]	0[b]
[Condition=taVNS] * [Phase=ON]	0[b]	0[b]	0[b]	0[b]

Matrice de covariance pour les estimations des effets fixes[a]

Paramètre	[Condition=taVNS] * [Phase=ON]
Constante	0[b]
[Condition=Sham]	0[b]
[Condition=taVNS]	0[b]
[Phase=OFF]	0[b]
[Phase=ON]	0[b]
[Condition=Sham] * [Phase=OFF]	0[b]
[Condition=Sham] * [Phase=ON]	0[b]
[Condition=taVNS] * [Phase=OFF]	0[b]
[Condition=taVNS] * [Phase=ON]	0[b]

a. Variable dépendante : Laser P2 Amplitude.

b. La covariance est définie sur 0, car elle est associée à un paramètre redondant.

Paramètres de covariance

Estimations des paramètres de covariance[a]

Paramètre	Estimation	Erreur standard	Z de Wald	Sig.	Intervalle de confiance à 95 % Borne inférieure	Borne supérieure
Résidu	39,938555	7,547677	5,292	,000	27,575943	57,843467

a. Variable dépendante : Laser P2 Amplitude.

Matrice de corrélation pour les estimations des paramètres de covariance[a]

Paramètre	Résidu
Résidu	1

a. Variable dépendante :
Laser P2 Amplitude.

Matrice de covariance pour les estimations des paramètres de covariance[a]

Paramètre	Résidu
Résidu	56,967435

a. Variable dépendante :
Laser P2 Amplitude.

Moyenne marginale estimée

1. Grand Mean[a]

Moyenne	Erreur standard	ddl	Intervalle de confiance à 95 % Borne inférieure	Borne supérieure
10,577	,816	56	8,943	12,212

a. Variable dépendante : Laser P2 Amplitude.

2. Condition

Estimations[a]

Condition	Moyenne	Erreur standard	ddl	Intervalle de confiance à 95 % Borne inférieure	Borne supérieure
Sham	11,919	1,154	56	9,608	14,230
taVNS	9,235	1,154	56	6,924	11,547

a. Variable dépendante : Laser P2 Amplitude.

Comparaisons appariées[a]

(I) Condition	(J) Condition	Différence moyenne (I-J)	Erreur standard	ddl	Sig.[b]
Sham	taVNS	2,684	1,632	56	,106
taVNS	Sham	-2,684	1,632	56	,106

Comparaisons appariées[a]

(I) Condition	(J) Condition	Intervalle de confiance à 95 % pour la différence[b] Borne inférieure	Borne supérieure
Sham	taVNS	-,585	5,953
taVNS	Sham	-5,953	,585

Basées sur les moyennes marginales estimées[a]

a. Variable dépendante : Laser P2 Amplitude.

b. Ajustement pour les comparaisons multiples : Bonferroni.

Tests univariés[a]

Ddl du numérateur	Ddl du dénominateur	F	Sig.
1	56	2,705	,106

Le test de F permet de tester l'effet de Condition. Il s'appuie sur les comparaisons appariées (indépendantes) linéaires parmi les moyennes marginales estimées.[a]

a. Variable dépendante : Laser P2 Amplitude.

3. Phase

Estimations[a]

Phase	Moyenne	Erreur standard	ddl	Intervalle de confiance à 95 % Borne inférieure	Borne supérieure
OFF	10,992	1,154	56	8,681	13,304
ON	10,162	1,154	56	7,851	12,474

a. Variable dépendante : Laser P2 Amplitude.

Comparaisons appariées[a]

(I) Phase	(J) Phase	Différence moyenne (I-J)	Erreur standard	ddl	Sig.[b]	Intervalle de confiance à 95 % pour la différence[b] Borne inférieure
OFF	ON	,830	1,632	56	,613	-2,439
ON	OFF	-,830	1,632	56	,613	-4,099

Comparaisons appariées[a]

(I) Phase	(J) Phase	Intervalle de confiance à 95 % pour la différence Borne supérieure
OFF	ON	4,099
ON	OFF	2,439

Basées sur les moyennes marginales estimées[a]

a. Variable dépendante : Laser P2 Amplitude.

b. Ajustement pour les comparaisons multiples : Bonferroni.

Tests univariés[a]

Ddl du numérateur	Ddl du dénominateur	F	Sig.
1	56	,259	,613

Le test de F permet de tester l'effet de Phase. Il s'appuie sur les comparaisons appariées (indépendantes) linéaires parmi les moyennes marginales estimées.[a]

a. Variable dépendante : Laser P2 Amplitude.

4. Condition * Phase[a]

Condition	Phase	Moyenne	Erreur standard	ddl	Intervalle de confiance à 95 % Borne inférieure	Borne supérieure
Sham	OFF	12,257	1,632	56	8,989	15,526
	ON	11,581	1,632	56	8,312	14,850
taVNS	OFF	9,727	1,632	56	6,458	12,996
	ON	8,743	1,632	56	5,475	12,012

a. Variable dépendante : Laser P2 Amplitude.

2.1.5. Laser P2 Latency.

```
MIXED LaserP2Latency BY Condition Phase
  /CRITERIA=CIN(95) MXITER(100) MXSTEP(10) SCORING(1)
SINGULAR(0.000000000001) HCONVERGE(0,
    ABSOLUTE) LCONVERGE(0, ABSOLUTE) PCONVERGE(0.000001, ABSOLUTE)
  /FIXED=Condition Phase Condition*Phase | SSTYPE(3)
  /METHOD=REML
  /PRINT=CPS CORB COVB DESCRIPTIVES G  SOLUTION TESTCOV
  /EMMEANS=TABLES(OVERALL)
  /EMMEANS=TABLES(Condition) COMPARE ADJ(BONFERRONI)
  /EMMEANS=TABLES(Phase) COMPARE ADJ(BONFERRONI)
  /EMMEANS=TABLES(Condition*Phase) .
```

Remarques

Sortie obtenue		05-MAY-2021 12:29:22
Commentaires		
Entrée	Jeu de données actif	Jeu_de_données2
	Filtre	<sans>
	Pondération	<sans>
	Fichier scindé	<sans>
	N de lignes dans le fichier de travail	60

Gestion des valeurs manquantes	Définition de la valeur manquante	Les valeurs manquantes définies par l'utilisateur sont traitées comme étant manquantes.
	Observations utilisées	Les statistiques sont basées sur toutes les observations comportant des données valides pour toutes les variables du modèle.
Syntaxe		MIXED LaserP2Latency BY Condition Phase /CRITERIA=CIN(95) MXITER(100) MXSTEP(10) SCORING(1) SINGULAR(0.000000000001) HCONVERGE(0, ABSOLUTE) LCONVERGE(0, ABSOLUTE) PCONVERGE(0.000001, ABSOLUTE) /FIXED=Condition Phase Condition*Phase | SSTYPE(3) /METHOD=REML /PRINT=CPS CORB COVB DESCRIPTIVES G SOLUTION TESTCOV /EMMEANS=TABLES(OVERALL) /EMMEANS=TABLES(Condition) COMPARE ADJ(BONFERRONI) /EMMEANS=TABLES(Phase) COMPARE ADJ(BONFERRONI) /EMMEANS=TABLES(Condition*Phase) .
Ressources	Temps de processeur	00:00:00,03

Temps écoulé	00:00:00,02

Récapitulatif de traitement des observations

		Effectif	Pourcentage marginal
Condition	Sham	30	50,0%
	taVNS	30	50,0%
Phase	OFF	30	50,0%
	ON	30	50,0%
Valide		60	100,0%
Exclues		0	
Total		60	

Statistiques descriptives

Laser P2 Latency

Condition	Phase	Effectif	Moyenne	Ecart type	Coefficient de variation
Sham	OFF	15	,37	,053	14,4%
	ON	15	,36	,047	13,3%
	Total	30	,36	,050	13,7%
taVNS	OFF	15	,37	,056	15,2%
	ON	15	,36	,049	13,6%
	Total	30	,37	,052	14,2%
Total	OFF	30	,37	,053	14,5%
	ON	30	,36	,047	13,2%
	Total	60	,36	,050	13,8%

Dimension du modèle[a]

		Nombre de niveaux	Nombre de paramètres
Effets fixes	Constante	1	1
	Condition	2	1
	Phase	2	1
	Condition * Phase	4	1
Résidu			1
Total		9	5

a. Variable dépendante : Laser P2 Latency .

Critères d'information[a]

Log de vraisemblance restreint -2	-162,649
Critère d'information d'Akaike (AIC)	-160,649
Critère de Hurvich et Tsai (AICC)	-160,575
Critère de Bozdogan (CAIC)	-157,623
Critère bayésien de Schwartz (BIC)	-158,623

Les critères d'informations sont présentés en plus petit, disposant d'un meilleur format.[a]
a. Variable dépendante : Laser P2 Latency
.

Effets fixes

Tests des effets fixes de type III[a]

Source	Ddl du numérateur	Ddl du dénominateur	F	Sig.
Constante	1	56	3001,853	,000
Condition	1	56	,062	,805
Phase	1	56	,498	,483
Condition * Phase	1	56	,043	,836

a. Variable dépendante : Laser P2 Latency .

Estimations des effets fixes[a]

Paramètre	Estimation	Erreur standard	ddl	t	Sig.
Constante	,362000	,013275	56	27,270	,000
[Condition=Sham]	-,006061	,018773	56	-,323	,748
[Condition=taVNS]	0[b]	0	.	.	.
[Phase=OFF]	,006600	,018773	56	,352	,726
[Phase=ON]	0[b]	0	.	.	.
[Condition=Sham] * [Phase=OFF]	,005533	,026549	56	,208	,836
[Condition=Sham] * [Phase=ON]	0[b]	0	.	.	.

[Condition=taVNS] * [Phase=OFF]	0[b]	0	.	.	.
[Condition=taVNS] * [Phase=ON]	0[b]	0	.	.	.

Estimations des effets fixes[a]

Paramètre	Intervalle de confiance à 95 % Borne inférieure	Borne supérieure
Constante	,335408	,388592
[Condition=Sham]	-,043668	,031546
[Condition=taVNS]	.	.
[Phase=OFF]	-,031007	,044207
[Phase=ON]	.	.
[Condition=Sham] * [Phase=OFF]	-,047652	,058717
[Condition=Sham] * [Phase=ON]	.	.
[Condition=taVNS] * [Phase=OFF]	.	.
[Condition=taVNS] * [Phase=ON]	.	.

a. Variable dépendante : Laser P2 Latency .

b. Ce paramètre est défini sur 0, car il est redondant.

Matrice de corrélation pour les estimations des effets fixes[a]

Paramètre	Constante	[Condition=Sham]	[Condition=taVNS]	[Phase=OFF]
Constante	1	-,707	.[b]	-,707
[Condition=Sham]	-,707	1	.[b]	,500
[Condition=taVNS]	.[b]	.[b]	.[b]	.[b]
[Phase=OFF]	-,707	,500	.[b]	1
[Phase=ON]	.[b]	.[b]	.[b]	.[b]
[Condition=Sham] * [Phase=OFF]	,500	-,707	.[b]	-,707
[Condition=Sham] * [Phase=ON]	.[b]	.[b]	.[b]	.[b]
[Condition=taVNS] * [Phase=OFF]	.[b]	.[b]	.[b]	.[b]
[Condition=taVNS] * [Phase=ON]	.[b]	.[b]	.[b]	.[b]

Matrice de corrélation pour les estimations des effets fixes[a]

Paramètre	[Phase=ON]	[Condition=Sham] * [Phase=OFF]	[Condition=Sham] * [Phase=ON]	[Condition=taVNS] * [Phase=OFF]
Constante	.[b]	,500	.[b]	.[b]

[Condition=Sham]	.[b]	-,707	.[b]	.[b]
[Condition=taVNS]	.[b]	.[b]	.[b]	.[b]
[Phase=OFF]	.[b]	-,707	.[b]	.[b]
[Phase=ON]	.[b]	.[b]	.[b]	.[b]
[Condition=Sham] * [Phase=OFF]	.[b]	1	.[b]	.[b]
[Condition=Sham] * [Phase=ON]	.[b]	.[b]	.[b]	.[b]
[Condition=taVNS] * [Phase=OFF]	.[b]	.[b]	.[b]	.[b]
[Condition=taVNS] * [Phase=ON]	.[b]	.[b]	.[b]	.[b]

Matrice de corrélation pour les estimations des effets fixes[a]

Paramètre	[Condition=taVNS] * [Phase=ON]
Constante	.[b]
[Condition=Sham]	.[b]
[Condition=taVNS]	.[b]
[Phase=OFF]	.[b]
[Phase=ON]	.[b]
[Condition=Sham] * [Phase=OFF]	.[b]
[Condition=Sham] * [Phase=ON]	.[b]
[Condition=taVNS] * [Phase=OFF]	.[b]
[Condition=taVNS] * [Phase=ON]	.[b]

a. Variable dépendante : Laser P2 Latency .

b. La corrélation est manquante par défaut, car elle est associée à un paramètre redondant.

Matrice de covariance pour les estimations des effets fixes[a]

Paramètre	Constante	[Condition=Sham]	[Condition=taVNS]	[Phase=OFF]
Constante	,000176	-,000176	0[b]	-,000176
[Condition=Sham]	-,000176	,000352	0[b]	,000176
[Condition=taVNS]	0[b]	0[b]	0[b]	0[b]
[Phase=OFF]	-,000176	,000176	0[b]	,000352
[Phase=ON]	0[b]	0[b]	0[b]	0[b]
[Condition=Sham] * [Phase=OFF]	,000176	-,000352	0[b]	-,000352
[Condition=Sham] * [Phase=ON]	0[b]	0[b]	0[b]	0[b]
[Condition=taVNS] * [Phase=OFF]	0[b]	0[b]	0[b]	0[b]

[Condition=taVNS] * [Phase=ON]	0[b]	0[b]	0[b]	0[b]

Matrice de covariance pour les estimations des effets fixes[a]

Paramètre	[Phase=ON]	[Condition=Sham] * [Phase=OFF]	[Condition=Sham] * [Phase=ON]	[Condition=taVNS] * [Phase=OFF]
Constante	0[b]	,000176	0[b]	0[b]
[Condition=Sham]	0[b]	-,000352	0[b]	0[b]
[Condition=taVNS]	0[b]	0[b]	0[b]	0[b]
[Phase=OFF]	0[b]	-,000352	0[b]	0[b]
[Phase=ON]	0[b]	0[b]	0[b]	0[b]
[Condition=Sham] * [Phase=OFF]	0[b]	,000705	0[b]	0[b]
[Condition=Sham] * [Phase=ON]	0[b]	0[b]	0[b]	0[b]
[Condition=taVNS] * [Phase=OFF]	0[b]	0[b]	0[b]	0[b]
[Condition=taVNS] * [Phase=ON]	0[b]	0[b]	0[b]	0[b]

Matrice de covariance pour les estimations des effets fixes[a]

Paramètre	[Condition=taVNS] * [Phase=ON]
Constante	0[b]
[Condition=Sham]	0[b]
[Condition=taVNS]	0[b]
[Phase=OFF]	0[b]
[Phase=ON]	0[b]
[Condition=Sham] * [Phase=OFF]	0[b]
[Condition=Sham] * [Phase=ON]	0[b]
[Condition=taVNS] * [Phase=OFF]	0[b]
[Condition=taVNS] * [Phase=ON]	0[b]

a. Variable dépendante : Laser P2 Latency .

b. La covariance est définie sur 0, car elle est associée à un paramètre redondant.

Paramètres de covariance

Estimations des paramètres de covariance[a]

Paramètre	Estimation	Erreur standard	Z de Wald	Sig.	Intervalle de confiance à 95 % Borne inférieure	Intervalle de confiance à 95 % Borne supérieure
Résidu	,002643	,000500	5,292	,000	,001825	,003828

a. Variable dépendante : Laser P2 Latency .

Matrice de corrélation pour les estimations des paramètres de covariance[a]

Paramètre	Résidu
Résidu	1

a. Variable dépendante : Laser P2 Latency .

Matrice de covariance pour les estimations des paramètres de covariance[a]

Paramètre	Résidu
Résidu	2,495248E-7

a. Variable dépendante : Laser P2 Latency .

Moyenne marginale estimée

1. Grand Mean[a]

			Intervalle de confiance à 95 %	
Moyenne	Erreur standard	ddl	Borne inférieure	Borne supérieure
,364	,007	56	,350	,377

a. Variable dépendante : Laser P2 Latency .

2. Condition

Estimations[a]

				Intervalle de confiance à 95 %	
Condition	Moyenne	Erreur standard	ddl	Borne inférieure	Borne supérieure
Sham	,362	,009	56	,343	,381
taVNS	,365	,009	56	,346	,384

a. Variable dépendante : Laser P2 Latency .

Comparaisons appariées[a]

(I) Condition	(J) Condition	Différence moyenne (I-J)	Erreur standard	ddl	Sig.[b]
Sham	taVNS	-,003	,013	56	,805
taVNS	Sham	,003	,013	56	,805

Comparaisons appariées[a]

		Intervalle de confiance à 95 % pour la différence[b]	
(I) Condition	(J) Condition	Borne inférieure	Borne supérieure
Sham	taVNS	-,030	,023
taVNS	Sham	-,023	,030

Basées sur les moyennes marginales estimées[a]

a. Variable dépendante : Laser P2 Latency .

b. Ajustement pour les comparaisons multiples : Bonferroni.

Tests univariés[a]

Ddl du numérateur	Ddl du dénominateur	F	Sig.
1	56	,062	,805

Le test de F permet de tester l'effet de Condition. Il s'appuie sur les comparaisons appariées (indépendantes) linéaires parmi les moyennes marginales estimées.[a]

a. Variable dépendante : Laser P2 Latency .

3. Phase

Estimations[a]

				Intervalle de confiance à 95 %	
Phase	Moyenne	Erreur standard	ddl	Borne inférieure	Borne supérieure
OFF	,368	,009	56	,350	,387
ON	,359	,009	56	,340	,378

a. Variable dépendante : Laser P2 Latency .

Comparaisons appariées[a]

(I) Phase	(J) Phase	Différence moyenne (I-J)	Erreur standard	ddl	Sig.[b]	Intervalle de confiance à 95 % pour la différence[b] Borne inférieure
OFF	ON	,009	,013	56	,483	-,017
ON	OFF	-,009	,013	56	,483	-,036

Comparaisons appariées[a]

(I) Phase	(J) Phase	Intervalle de confiance à 95 % pour la différence Borne supérieure
OFF	ON	,036
ON	OFF	,017

Basées sur les moyennes marginales estimées[a]

a. Variable dépendante : Laser P2 Latency .

b. Ajustement pour les comparaisons multiples : Bonferroni.

Tests univariés[a]

Ddl du numérateur	Ddl du dénominateur	F	Sig.
1	56	,498	,483

Le test de F permet de tester l'effet de Phase. Il s'appuie sur les comparaisons appariées (indépendantes) linéaires parmi les moyennes marginales estimées.[a]

a. Variable dépendante : Laser P2 Latency .

4. Condition * Phase[a]

Condition	Phase	Moyenne	Erreur standard	ddl	Intervalle de confiance à 95 % Borne inférieure	Intervalle de confiance à 95 % Borne supérieure
Sham	OFF	,368	,013	56	,341	,395
	ON	,356	,013	56	,329	,383
taVNS	OFF	,369	,013	56	,342	,395
	ON	,362	,013	56	,335	,389

a. Variable dépendante : Laser P2 Latency .

2.2. Vibrotactile-evoked potentials.

2.2.1 Vibrotactile N2P2 Amplitude.

```
MIXED VibrotactileN2P2Amplitude BY Condition Phase
  /CRITERIA=CIN(95) MXITER(100) MXSTEP(10) SCORING(1)
SINGULAR(0.000000000001) HCONVERGE(0,
    ABSOLUTE) LCONVERGE(0, ABSOLUTE) PCONVERGE(0.000001, ABSOLUTE)
  /FIXED=Condition Phase Condition*Phase | SSTYPE(3)
  /METHOD=REML
  /PRINT=CPS CORB COVB DESCRIPTIVES G  SOLUTION TESTCOV
  /EMMEANS=TABLES(OVERALL)
  /EMMEANS=TABLES(Condition) COMPARE ADJ(BONFERRONI)
  /EMMEANS=TABLES(Phase) COMPARE ADJ(BONFERRONI)
  /EMMEANS=TABLES(Condition*Phase) .
```

Remarques

Sortie obtenue		05-MAY-2021 12:30:04
Commentaires		
Entrée	Jeu de données actif	Jeu_de_données2
	Filtre	<sans>
	Pondération	<sans>
	Fichier scindé	<sans>
	N de lignes dans le fichier de travail	60
Gestion des valeurs manquantes	Définition de la valeur manquante	Les valeurs manquantes définies par l'utilisateur sont traitées comme étant manquantes.
	Observations utilisées	Les statistiques sont basées sur toutes les observations comportant des données valides pour toutes les variables du modèle.

Syntaxe		MIXED VibrotactileN2P2Amplitude BY Condition Phase<br>/CRITERIA=CIN(95) MXITER(100) MXSTEP(10) SCORING(1) SINGULAR(0.000000000001) HCONVERGE(0, ABSOLUTE) LCONVERGE(0, ABSOLUTE) PCONVERGE(0.000001, ABSOLUTE)<br>/FIXED=Condition Phase Condition*Phase | SSTYPE(3)<br>/METHOD=REML<br>/PRINT=CPS CORB COVB DESCRIPTIVES G SOLUTION TESTCOV<br>/EMMEANS=TABLES(OVERALL)<br>/EMMEANS=TABLES(Condition) COMPARE ADJ(BONFERRONI)<br>/EMMEANS=TABLES(Phase) COMPARE ADJ(BONFERRONI)<br>/EMMEANS=TABLES(Condition*Phase) .
Ressources	Temps de processeur	00:00:00,02
	Temps écoulé	00:00:00,01

Récapitulatif de traitement des observations

		Effectif	Pourcentage marginal
Condition	Sham	30	50,0%

	taVNS	30	50,0%
Phase	OFF	30	50,0%
	ON	30	50,0%
Valide		60	100,0%
Exclues		0	
Total		60	

Statistiques descriptives

Vibrotactile N2P2 Amplitude

Condition	Phase	Effectif	Moyenne	Ecart type	Coefficient de variation
Sham	OFF	15	20,080966666666672	6,568294173926598	32,7%
	ON	15	17,889520000000000	6,679932172688154	37,3%
	Total	30	18,985243333333337	6,603849554755968	34,8%
taVNS	OFF	15	18,535640000000000	5,708890220499452	30,8%
	ON	15	17,718766666666667	6,480924253824413	36,6%
	Total	30	18,127203333333330	6,015261938830852	33,2%
Total	OFF	30	19,308303333333335	6,097441751484562	31,6%
	ON	30	17,804143333333336	6,467299434992212	36,3%
	Total	60	18,556223333333335	6,277583793158343	33,8%

Dimension du modèle[a]

		Nombre de niveaux	Nombre de paramètres
Effets fixes	Constante	1	1
	Condition	2	1
	Phase	2	1
	Condition * Phase	4	1
Résidu			1
Total		9	5

a. Variable dépendante : Vibrotactile N2P2 Amplitude.

Critères d'information[a]

Log de vraisemblance restreint -2	377,150
Critère d'information d'Akaike (AIC)	379,150
Critère de Hurvich et Tsai (AICC)	379,224
Critère de Bozdogan (CAIC)	382,175
Critère bayésien de Schwartz (BIC)	381,175

Les critères d'informations sont présentés en plus petit, disposant d'un meilleur format.[a]

a. Variable dépendante : Vibrotactile N2P2 Amplitude.

Effets fixes

Tests des effets fixes de type III[a]

Source	Ddl du numérateur	Ddl du dénominateur	F	Sig.
Constante	1	56	508,999	,000
Condition	1	56	,272	,604
Phase	1	56	,836	,364
Condition * Phase	1	56	,175	,678

a. Variable dépendante : Vibrotactile N2P2 Amplitude.

Estimations des effets fixes[a]

Paramètre	Estimation	Erreur standard	ddl	t	Sig.
Constante	17,718767	1,644981	56	10,771	,000
[Condition=Sham]	,170753	2,326355	56	,073	,942
[Condition=taVNS]	0[b]	0	.	.	.
[Phase=OFF]	,816873	2,326355	56	,351	,727
[Phase=ON]	0[b]	0	.	.	.
[Condition=Sham] * [Phase=OFF]	1,374573	3,289962	56	,418	,678
[Condition=Sham] * [Phase=ON]	0[b]	0	.	.	.
[Condition=taVNS] * [Phase=OFF]	0[b]	0	.	.	.

[Condition=taVNS] * [Phase=ON]	0[b]	0	.	.	.

Estimations des effets fixes[a]

Paramètre	Intervalle de confiance à 95 % Borne inférieure	Borne supérieure
Constante	14,423473	21,014060
[Condition=Sham]	-4,489495	4,831002
[Condition=taVNS]	.	.
[Phase=OFF]	-3,843375	5,477122
[Phase=ON]	.	.
[Condition=Sham] * [Phase=OFF]	-5,216013	7,965160
[Condition=Sham] * [Phase=ON]	.	.
[Condition=taVNS] * [Phase=OFF]	.	.
[Condition=taVNS] * [Phase=ON]	.	.

a. Variable dépendante : Vibrotactile N2P2 Amplitude.

b. Ce paramètre est défini sur 0, car il est redondant.

Matrice de corrélation pour les estimations des effets fixes[a]

Paramètre	Constante	[Condition=Sham]	[Condition=taVNS]	[Phase=OFF]
Constante	1	-,707	.[b]	-,707
[Condition=Sham]	-,707	1	.[b]	,500
[Condition=taVNS]	.[b]	.[b]	.[b]	.[b]
[Phase=OFF]	-,707	,500	.[b]	1
[Phase=ON]	.[b]	.[b]	.[b]	.[b]
[Condition=Sham] * [Phase=OFF]	,500	-,707	.[b]	-,707
[Condition=Sham] * [Phase=ON]	.[b]	.[b]	.[b]	.[b]
[Condition=taVNS] * [Phase=OFF]	.[b]	.[b]	.[b]	.[b]
[Condition=taVNS] * [Phase=ON]	.[b]	.[b]	.[b]	.[b]

Matrice de corrélation pour les estimations des effets fixes[a]

Paramètre	[Phase=ON]	[Condition=Sham] * [Phase=OFF]	[Condition=Sham] * [Phase=ON]	[Condition=taVNS] * [Phase=OFF]
Constante	.[b]	,500	.[b]	.[b]
[Condition=Sham]	.[b]	-,707	.[b]	.[b]
[Condition=taVNS]	.[b]	.[b]	.[b]	.[b]

[Phase=OFF]	.[b]	-,707	.[b]	.[b]
[Phase=ON]	.[b]	.[b]	.[b]	.[b]
[Condition=Sham] * [Phase=OFF]	.[b]	1	.[b]	.[b]
[Condition=Sham] * [Phase=ON]	.[b]	.[b]	.[b]	.[b]
[Condition=taVNS] * [Phase=OFF]	.[b]	.[b]	.[b]	.[b]
[Condition=taVNS] * [Phase=ON]	.[b]	.[b]	.[b]	.[b]

Matrice de corrélation pour les estimations des effets fixes[a]

Paramètre	[Condition=taVNS] * [Phase=ON]
Constante	.[b]
[Condition=Sham]	.[b]
[Condition=taVNS]	.[b]
[Phase=OFF]	.[b]
[Phase=ON]	.[b]
[Condition=Sham] * [Phase=OFF]	.[b]
[Condition=Sham] * [Phase=ON]	.[b]
[Condition=taVNS] * [Phase=OFF]	.[b]
[Condition=taVNS] * [Phase=ON]	.[b]

a. Variable dépendante : Vibrotactile N2P2 Amplitude.

b. La corrélation est manquante par défaut, car elle est associée à un paramètre redondant.

Matrice de covariance pour les estimations des effets fixes[a]

Paramètre	Constante	[Condition=Sham]	[Condition=taVNS]	[Phase=OFF]
Constante	2,705963	-2,705963	0[b]	-2,705963
[Condition=Sham]	-2,705963	5,411926	0[b]	2,705963
[Condition=taVNS]	0[b]	0[b]	0[b]	0[b]
[Phase=OFF]	-2,705963	2,705963	0[b]	5,411926
[Phase=ON]	0[b]	0[b]	0[b]	0[b]
[Condition=Sham] * [Phase=OFF]	2,705963	-5,411926	0[b]	-5,411926
[Condition=Sham] * [Phase=ON]	0[b]	0[b]	0[b]	0[b]
[Condition=taVNS] * [Phase=OFF]	0[b]	0[b]	0[b]	0[b]
[Condition=taVNS] * [Phase=ON]	0[b]	0[b]	0[b]	0[b]

Matrice de covariance pour les estimations des effets fixes[a]

Paramètre	[Phase=ON]	[Condition=Sham] * [Phase=OFF]	[Condition=Sham] * [Phase=ON]	[Condition=taVNS] * [Phase=OFF]
Constante	0[b]	2,705963	0[b]	0[b]
[Condition=Sham]	0[b]	-5,411926	0[b]	0[b]
[Condition=taVNS]	0[b]	0[b]	0[b]	0[b]
[Phase=OFF]	0[b]	-5,411926	0[b]	0[b]
[Phase=ON]	0[b]	0[b]	0[b]	0[b]
[Condition=Sham] * [Phase=OFF]	0[b]	10,823853	0[b]	0[b]
[Condition=Sham] * [Phase=ON]	0[b]	0[b]	0[b]	0[b]
[Condition=taVNS] * [Phase=OFF]	0[b]	0[b]	0[b]	0[b]
[Condition=taVNS] * [Phase=ON]	0[b]	0[b]	0[b]	0[b]

Matrice de covariance pour les estimations des effets fixes[a]

Paramètre	[Condition=taVNS] * [Phase=ON]
Constante	0[b]
[Condition=Sham]	0[b]
[Condition=taVNS]	0[b]
[Phase=OFF]	0[b]
[Phase=ON]	0[b]
[Condition=Sham] * [Phase=OFF]	0[b]
[Condition=Sham] * [Phase=ON]	0[b]
[Condition=taVNS] * [Phase=OFF]	0[b]
[Condition=taVNS] * [Phase=ON]	0[b]

a. Variable dépendante : Vibrotactile N2P2 Amplitude.

b. La covariance est définie sur 0, car elle est associée à un paramètre redondant.

Paramètres de covariance

Estimations des paramètres de covariance[a]

Paramètre	Estimation	Erreur standard	Z de Wald	Sig.	Intervalle de confiance à 95 %	
					Borne inférieure	Borne supérieure
Résidu	40,589447	7,670685	5,292	,000	28,025358	58,786162

a. Variable dépendante : Vibrotactile N2P2 Amplitude.

Matrice de corrélation pour les estimations des paramètres de covariance[a]

Paramètre	Résidu
Résidu	1

a. Variable dépendante : Vibrotactile N2P2 Amplitude.

Matrice de covariance pour les estimations des paramètres de covariance[a]

Paramètre	Résidu
Résidu	58,839401

a. Variable dépendante : Vibrotactile N2P2 Amplitude.

Moyenne marginale estimée

1. Grand Mean[a]

Moyenne	Erreur standard	ddl	Intervalle de confiance à 95 %	
			Borne inférieure	Borne supérieure
18,556	,822	56	16,909	20,204

a. Variable dépendante : Vibrotactile N2P2 Amplitude.

2. Condition

Estimations[a]

Condition	Moyenne	Erreur standard	ddl	Intervalle de confiance à 95 %	
				Borne inférieure	Borne supérieure
Sham	18,985	1,163	56	16,655	21,315
taVNS	18,127	1,163	56	15,797	20,457

a. Variable dépendante : Vibrotactile N2P2 Amplitude.

Comparaisons appariées[a]

(I) Condition	(J) Condition	Différence moyenne (I-J)	Erreur standard	ddl	Sig.[b]
Sham	taVNS	,858	1,645	56	,604
taVNS	Sham	-,858	1,645	56	,604

Comparaisons appariées[a]

		Intervalle de confiance à 95 % pour la différence[b]	
(I) Condition	(J) Condition	Borne inférieure	Borne supérieure
Sham	taVNS	-2,437	4,153
taVNS	Sham	-4,153	2,437

Basées sur les moyennes marginales estimées[a]

a. Variable dépendante : Vibrotactile N2P2 Amplitude.

b. Ajustement pour les comparaisons multiples : Bonferroni.

Tests univariés[a]

Ddl du numérateur	Ddl du dénominateur	F	Sig.
1	56	,272	,604

Le test de F permet de tester l'effet de Condition. Il s'appuie sur les comparaisons appariées (indépendantes) linéaires parmi les moyennes marginales estimées.[a]

a. Variable dépendante : Vibrotactile N2P2 Amplitude.

3. Phase

Estimations[a]

				Intervalle de confiance à 95 %	
Phase	Moyenne	Erreur standard	ddl	Borne inférieure	Borne supérieure
OFF	19,308	1,163	56	16,978	21,638
ON	17,804	1,163	56	15,474	20,134

a. Variable dépendante : Vibrotactile N2P2 Amplitude.

Comparaisons appariées[a]

(I) Phase	(J) Phase	Différence moyenne (I-J)	Erreur standard	ddl	Sig.[b]	Intervalle de confiance à 95 % pour la différence[b] Borne inférieure
OFF	ON	1,504	1,645	56	,364	-1,791
ON	OFF	-1,504	1,645	56	,364	-4,799

Comparaisons appariées[a]

(I) Phase	(J) Phase	Intervalle de confiance à 95 % pour la différence Borne supérieure
OFF	ON	4,799
ON	OFF	1,791

Basées sur les moyennes marginales estimées[a]

a. Variable dépendante : Vibrotactile N2P2 Amplitude.

b. Ajustement pour les comparaisons multiples : Bonferroni.

Tests univariés[a]

Ddl du numérateur	Ddl du dénominateur	F	Sig.
1	56	,836	,364

Le test de F permet de tester l'effet de Phase. Il s'appuie sur les comparaisons appariées (indépendantes) linéaires parmi les moyennes marginales estimées.[a]

a. Variable dépendante : Vibrotactile N2P2 Amplitude.

4. Condition * Phase[a]

Condition	Phase	Moyenne	Erreur standard	ddl	Intervalle de confiance à 95 % Borne inférieure	Intervalle de confiance à 95 % Borne supérieure
Sham	OFF	20,081	1,645	56	16,786	23,376
	ON	17,890	1,645	56	14,594	21,185
taVNS	OFF	18,536	1,645	56	15,240	21,831
	ON	17,719	1,645	56	14,423	21,014

a. Variable dépendante : Vibrotactile N2P2 Amplitude.

2.2.2. Vibrotactile N2 Amplitude.

```
MIXED VibrotactileN2Amplitude BY Condition Phase
  /CRITERIA=CIN(95) MXITER(100) MXSTEP(10) SCORING(1)
SINGULAR(0.000000000001) HCONVERGE(0,
    ABSOLUTE) LCONVERGE(0, ABSOLUTE) PCONVERGE(0.000001, ABSOLUTE)
  /FIXED=Condition Phase Condition*Phase | SSTYPE(3)
  /METHOD=REML
  /PRINT=CPS CORB COVB DESCRIPTIVES G  SOLUTION TESTCOV
  /EMMEANS=TABLES(OVERALL)
  /EMMEANS=TABLES(Condition) COMPARE ADJ(BONFERRONI)
  /EMMEANS=TABLES(Phase) COMPARE ADJ(BONFERRONI)
  /EMMEANS=TABLES(Condition*Phase) .
```

Remarques

Sortie obtenue		05-MAY-2021 12:30:40
Commentaires		
Entrée	Jeu de données actif	Jeu_de_données2
	Filtre	<sans>
	Pondération	<sans>
	Fichier scindé	<sans>
	N de lignes dans le fichier de travail	60
Gestion des valeurs manquantes	Définition de la valeur manquante	Les valeurs manquantes définies par l'utilisateur sont traitées comme étant manquantes.
	Observations utilisées	Les statistiques sont basées sur toutes les observations comportant des données valides pour toutes les variables du modèle.

Syntaxe		MIXED VibrotactileN2Amplitude BY Condition Phase<br>/CRITERIA=CIN(95) MXITER(100) MXSTEP(10) SCORING(1) SINGULAR(0.000000000001) HCONVERGE(0,<br>ABSOLUTE) LCONVERGE(0, ABSOLUTE) PCONVERGE(0.000001, ABSOLUTE)<br>/FIXED=Condition Phase Condition*Phase | SSTYPE(3)<br>/METHOD=REML<br>/PRINT=CPS CORB COVB DESCRIPTIVES G SOLUTION TESTCOV<br><br>/EMMEANS=TABLES(OVERALL)<br><br>/EMMEANS=TABLES(Condition) COMPARE ADJ(BONFERRONI)<br><br>/EMMEANS=TABLES(Phase) COMPARE ADJ(BONFERRONI)<br><br>/EMMEANS=TABLES(Condition*Phase) .
Ressources	Temps de processeur	00:00:00,03
	Temps écoulé	00:00:00,02

Récapitulatif de traitement des observations

		Effectif	Pourcentage marginal
Condition	Sham	30	50,0%

	taVNS	30	50,0%
Phase	OFF	30	50,0%
	ON	30	50,0%
Valide		60	100,0%
Exclues		0	
Total		60	

Statistiques descriptives

Vibrotactile N2 Amplitude

Condition	Phase	Effectif	Moyenne	Ecart type	Coefficient de variation
Sham	OFF	15	-9,12	3,211	-35,2%
	ON	15	-7,45	3,518	-47,2%
	Total	30	-8,28	3,418	-41,3%
taVNS	OFF	15	-8,41	4,305	-51,2%
	ON	15	-8,47	4,460	-52,7%
	Total	30	-8,44	4,307	-51,0%
Total	OFF	30	-8,76	3,749	-42,8%
	ON	30	-7,96	3,981	-50,0%
	Total	60	-8,36	3,855	-46,1%

Dimension du modèle[a]

		Nombre de niveaux	Nombre de paramètres
Effets fixes	Constante	1	1
	Condition	2	1
	Phase	2	1
	Condition * Phase	4	1
Résidu			1
Total		9	5

a. Variable dépendante : Vibrotactile N2 Amplitude.

Critères d'information[a]

Log de vraisemblance restreint -2	322,431
Critère d'information d'Akaike (AIC)	324,431
Critère de Hurvich et Tsai (AICC)	324,505
Critère de Bozdogan (CAIC)	327,456

Critère bayésien de Schwartz (BIC)	326,456

Les critères d'informations sont présentés en plus petit, disposant d'un meilleur format.[a]

a. Variable dépendante : Vibrotactile N2 Amplitude.

Effets fixes

Tests des effets fixes de type III[a]

Source	Ddl du numérateur	Ddl du dénominateur	F	Sig.
Constante	1	56	274,551	,000
Condition	1	56	,023	,879
Phase	1	56	,640	,427
Condition * Phase	1	56	,738	,394

a. Variable dépendante : Vibrotactile N2 Amplitude.

Estimations des effets fixes[a]

Paramètre	Estimation	Erreur standard	ddl	t	Sig.
Constante	-8,468080	1,009207	56	-8,391	,000
[Condition=Sham]	1,021293	1,427234	56	,716	,477
[Condition=taVNS]	0[b]	0	.	.	.
[Phase=OFF]	,059880	1,427234	56	,042	,967
[Phase=ON]	0[b]	0	.	.	.
[Condition=Sham] * [Phase=OFF]	-1,734293	2,018414	56	-,859	,394
[Condition=Sham] * [Phase=ON]	0[b]	0	.	.	.
[Condition=taVNS] * [Phase=OFF]	0[b]	0	.	.	.
[Condition=taVNS] * [Phase=ON]	0[b]	0	.	.	.

Estimations des effets fixes[a]

Paramètre	Intervalle de confiance à 95 %	
	Borne inférieure	Borne supérieure
Constante	-10,489764	-6,446396
[Condition=Sham]	-1,837800	3,880387

[Condition=taVNS]	.	.
[Phase=OFF]	-2,799213	2,918973
[Phase=ON]	.	.
[Condition=Sham] * [Phase=OFF]	-5,777662	2,309075
[Condition=Sham] * [Phase=ON]	.	.
[Condition=taVNS] * [Phase=OFF]	.	.
[Condition=taVNS] * [Phase=ON]	.	.

a. Variable dépendante : Vibrotactile N2 Amplitude.

b. Ce paramètre est défini sur 0, car il est redondant.

Matrice de corrélation pour les estimations des effets fixes[a]

Paramètre	Constante	[Condition=Sham]	[Condition=taVNS]	[Phase=OFF]
Constante	1	-,707	.[b]	-,707
[Condition=Sham]	-,707	1	.[b]	,500
[Condition=taVNS]	.[b]	.[b]	.[b]	.[b]
[Phase=OFF]	-,707	,500	.[b]	1
[Phase=ON]	.[b]	.[b]	.[b]	.[b]
[Condition=Sham] * [Phase=OFF]	,500	-,707	.[b]	-,707
[Condition=Sham] * [Phase=ON]	.[b]	.[b]	.[b]	.[b]
[Condition=taVNS] * [Phase=OFF]	.[b]	.[b]	.[b]	.[b]
[Condition=taVNS] * [Phase=ON]	.[b]	.[b]	.[b]	.[b]

Matrice de corrélation pour les estimations des effets fixes[a]

Paramètre	[Phase=ON]	[Condition=Sham] * [Phase=OFF]	[Condition=Sham] * [Phase=ON]	[Condition=taVNS] * [Phase=OFF]
Constante	.[b]	,500	.[b]	.[b]
[Condition=Sham]	.[b]	-,707	.[b]	.[b]
[Condition=taVNS]	.[b]	.[b]	.[b]	.[b]
[Phase=OFF]	.[b]	-,707	.[b]	.[b]
[Phase=ON]	.[b]	.[b]	.[b]	.[b]
[Condition=Sham] * [Phase=OFF]	.[b]	1	.[b]	.[b]
[Condition=Sham] * [Phase=ON]	.[b]	.[b]	.[b]	.[b]
[Condition=taVNS] * [Phase=OFF]	.[b]	.[b]	.[b]	.[b]

[Condition=taVNS] * [Phase=ON]	.[b]	.[b]	.[b]	.[b]

Matrice de corrélation pour les estimations des effets fixes[a]

Paramètre	[Condition=taVNS] * [Phase=ON]
Constante	.[b]
[Condition=Sham]	.[b]
[Condition=taVNS]	.[b]
[Phase=OFF]	.[b]
[Phase=ON]	.[b]
[Condition=Sham] * [Phase=OFF]	.[b]
[Condition=Sham] * [Phase=ON]	.[b]
[Condition=taVNS] * [Phase=OFF]	.[b]
[Condition=taVNS] * [Phase=ON]	.[b]

a. Variable dépendante : Vibrotactile N2 Amplitude.

b. La corrélation est manquante par défaut, car elle est associée à un paramètre redondant.

Matrice de covariance pour les estimations des effets fixes[a]

Paramètre	Constante	[Condition=Sham]	[Condition=taVNS]	[Phase=OFF]
Constante	1,018498	-1,018498	0[b]	-1,018498
[Condition=Sham]	-1,018498	2,036997	0[b]	1,018498
[Condition=taVNS]	0[b]	0[b]	0[b]	0[b]
[Phase=OFF]	-1,018498	1,018498	0[b]	2,036997
[Phase=ON]	0[b]	0[b]	0[b]	0[b]
[Condition=Sham] * [Phase=OFF]	1,018498	-2,036997	0[b]	-2,036997
[Condition=Sham] * [Phase=ON]	0[b]	0[b]	0[b]	0[b]
[Condition=taVNS] * [Phase=OFF]	0[b]	0[b]	0[b]	0[b]
[Condition=taVNS] * [Phase=ON]	0[b]	0[b]	0[b]	0[b]

Matrice de covariance pour les estimations des effets fixes[a]

Paramètre	[Phase=ON]	[Condition=Sham] * [Phase=OFF]	[Condition=Sham] * [Phase=ON]	[Condition=taVNS] * [Phase=OFF]
Constante	0[b]	1,018498	0[b]	0[b]
[Condition=Sham]	0[b]	-2,036997	0[b]	0[b]
[Condition=taVNS]	0[b]	0[b]	0[b]	0[b]
[Phase=OFF]	0[b]	-2,036997	0[b]	0[b]

[Phase=ON]	0[b]	0[b]	0[b]	0[b]
[Condition=Sham] * [Phase=OFF]	0[b]	4,073994	0[b]	0[b]
[Condition=Sham] * [Phase=ON]	0[b]	0[b]	0[b]	0[b]
[Condition=taVNS] * [Phase=OFF]	0[b]	0[b]	0[b]	0[b]
[Condition=taVNS] * [Phase=ON]	0[b]	0[b]	0[b]	0[b]

Matrice de covariance pour les estimations des effets fixes[a]

Paramètre	[Condition=taVNS] * [Phase=ON]
Constante	0[b]
[Condition=Sham]	0[b]
[Condition=taVNS]	0[b]
[Phase=OFF]	0[b]
[Phase=ON]	0[b]
[Condition=Sham] * [Phase=OFF]	0[b]
[Condition=Sham] * [Phase=ON]	0[b]
[Condition=taVNS] * [Phase=OFF]	0[b]
[Condition=taVNS] * [Phase=ON]	0[b]

a. Variable dépendante : Vibrotactile N2 Amplitude.

b. La covariance est définie sur 0, car elle est associée à un paramètre redondant.

Paramètres de covariance

Estimations des paramètres de covariance[a]

					Intervalle de confiance à 95 %	
Paramètre	Estimation	Erreur standard	Z de Wald	Sig.	Borne inférieure	Borne supérieure
Résidu	15,277477	2,887172	5,292	,000	10,548475	22,126545

a. Variable dépendante : Vibrotactile N2 Amplitude.

Matrice de corrélation pour les estimations des paramètres de covariance[a]

Paramètre	Résidu
Résidu	1

a. Variable dépendante : Vibrotactile N2 Amplitude.

Matrice de covariance pour les estimations des paramètres de covariance[a]

Paramètre	Résidu
Résidu	8,335761

a. Variable dépendante : Vibrotactile N2 Amplitude.

Moyenne marginale estimée

1. Grand Mean[a]

Moyenne	Erreur standard	ddl	Intervalle de confiance à 95 % Borne inférieure	Borne supérieure
-8,361	,505	56	-9,372	-7,350

a. Variable dépendante : Vibrotactile N2 Amplitude.

2. Condition

Estimations[a]

Condition	Moyenne	Erreur standard	ddl	Intervalle de confiance à 95 % Borne inférieure	Borne supérieure
Sham	-8,284	,714	56	-9,714	-6,854
taVNS	-8,438	,714	56	-9,868	-7,009

a. Variable dépendante : Vibrotactile N2 Amplitude.

Comparaisons appariées[a]

(I) Condition	(J) Condition	Différence moyenne (I-J)	Erreur standard	ddl	Sig.[b]
Sham	taVNS	,154	1,009	56	,879
taVNS	Sham	-,154	1,009	56	,879

Comparaisons appariées[a]

		Intervalle de confiance à 95 % pour la différence[b]	
(I) Condition	(J) Condition	Borne inférieure	Borne supérieure
Sham	taVNS	-1,868	2,176
taVNS	Sham	-2,176	1,868

Basées sur les moyennes marginales estimées[a]

a. Variable dépendante : Vibrotactile N2 Amplitude.

b. Ajustement pour les comparaisons multiples : Bonferroni.

Tests univariés[a]

Ddl du numérateur	Ddl du dénominateur	F	Sig.
1	56	,023	,879

Le test de F permet de tester l'effet de Condition. Il s'appuie sur les comparaisons appariées (indépendantes) linéaires parmi les moyennes marginales estimées.[a]

a. Variable dépendante : Vibrotactile N2 Amplitude.

3. Phase

Estimations[a]

				Intervalle de confiance à 95 %	
Phase	Moyenne	Erreur standard	ddl	Borne inférieure	Borne supérieure
OFF	-8,765	,714	56	-10,194	-7,335
ON	-7,957	,714	56	-9,387	-6,528

a. Variable dépendante : Vibrotactile N2 Amplitude.

Comparaisons appariées[a]

(I) Phase	(J) Phase	Différence moyenne (I-J)	Erreur standard	ddl	Sig.[b]	Intervalle de confiance à 95 % pour la différence[b] Borne inférieure
OFF	ON	-,807	1,009	56	,427	-2,829
ON	OFF	,807	1,009	56	,427	-1,214

Comparaisons appariées[a]

(I) Phase	(J) Phase	Intervalle de confiance à 95 % pour la différence Borne supérieure
OFF	ON	1,214
ON	OFF	2,829

Basées sur les moyennes marginales estimées[a]

a. Variable dépendante : Vibrotactile N2 Amplitude.

b. Ajustement pour les comparaisons multiples : Bonferroni.

Tests univariés[a]

Ddl du numérateur	Ddl du dénominateur	F	Sig.
1	56	,640	,427

Le test de F permet de tester l'effet de Phase. Il s'appuie sur les comparaisons appariées (indépendantes) linéaires parmi les moyennes marginales estimées.[a]

a. Variable dépendante : Vibrotactile N2 Amplitude.

4. Condition * Phase[a]

Condition	Phase	Moyenne	Erreur standard	ddl	Intervalle de confiance à 95 % Borne inférieure	Intervalle de confiance à 95 % Borne supérieure
Sham	OFF	-9,121	1,009	56	-11,143	-7,100
	ON	-7,447	1,009	56	-9,468	-5,425
taVNS	OFF	-8,408	1,009	56	-10,430	-6,387
	ON	-8,468	1,009	56	-10,490	-6,446

a. Variable dépendante : Vibrotactile N2 Amplitude.

2.2.3. Vibrotactile N2 Latency.

```
MIXED VibrotactileN2Latency BY Condition Phase
  /CRITERIA=CIN(95) MXITER(100) MXSTEP(10) SCORING(1)
SINGULAR(0.000000000001) HCONVERGE(0,
    ABSOLUTE) LCONVERGE(0, ABSOLUTE) PCONVERGE(0.000001, ABSOLUTE)
  /FIXED=Condition Phase Condition*Phase | SSTYPE(3)
  /METHOD=REML
  /PRINT=CPS CORB COVB DESCRIPTIVES G  SOLUTION TESTCOV
  /EMMEANS=TABLES(OVERALL)
  /EMMEANS=TABLES(Condition) COMPARE ADJ(BONFERRONI)
  /EMMEANS=TABLES(Phase) COMPARE ADJ(BONFERRONI)
  /EMMEANS=TABLES(Condition*Phase) .
```

Remarques

Sortie obtenue		05-MAY-2021 12:31:15
Commentaires		
Entrée	Jeu de données actif	Jeu_de_données2
	Filtre	<sans>
	Pondération	<sans>
	Fichier scindé	<sans>
	N de lignes dans le fichier de travail	60
Gestion des valeurs manquantes	Définition de la valeur manquante	Les valeurs manquantes définies par l'utilisateur sont traitées comme étant manquantes.
	Observations utilisées	Les statistiques sont basées sur toutes les observations comportant des données valides pour toutes les variables du modèle.

Syntaxe		MIXED VibrotactileN2Latency BY Condition Phase<br>/CRITERIA=CIN(95) MXITER(100) MXSTEP(10) SCORING(1) SINGULAR(0.000000000001) HCONVERGE(0, ABSOLUTE) LCONVERGE(0, ABSOLUTE) PCONVERGE(0.000001, ABSOLUTE)<br>/FIXED=Condition Phase Condition*Phase | SSTYPE(3)<br>/METHOD=REML<br>/PRINT=CPS CORB COVB DESCRIPTIVES G SOLUTION TESTCOV<br>/EMMEANS=TABLES(OVERALL)<br>/EMMEANS=TABLES(Condition) COMPARE ADJ(BONFERRONI)<br>/EMMEANS=TABLES(Phase) COMPARE ADJ(BONFERRONI)<br>/EMMEANS=TABLES(Condition*Phase) .
Ressources	Temps de processeur	00:00:00,00
	Temps écoulé	00:00:00,01

Récapitulatif de traitement des observations

		Effectif	Pourcentage marginal
Condition	Sham	30	50,0%

	taVNS	30	50,0%
Phase	OFF	30	50,0%
	ON	30	50,0%
Valide		60	100,0%
Exclues		0	
Total		60	

Statistiques descriptives

Vibrotactile N2 Latency

Condition	Phase	Effectif	Moyenne	Ecart type	Coefficient de variation
Sham	OFF	15	,14	,015	10,9%
	ON	15	,14	,013	9,4%
	Total	30	,14	,014	10,0%
taVNS	OFF	15	,14	,017	12,4%
	ON	15	,13	,029	22,0%
	Total	30	,13	,024	17,5%
Total	OFF	30	,14	,016	11,5%
	ON	30	,14	,023	16,6%
	Total	60	,14	,020	14,2%

Dimension du modèle[a]

		Nombre de niveaux	Nombre de paramètres
Effets fixes	Constante	1	1
	Condition	2	1
	Phase	2	1
	Condition * Phase	4	1
Résidu			1
Total		9	5

a. Variable dépendante : Vibrotactile N2 Latency .

Critères d'information[a]

Log de vraisemblance restreint -2	-270,468
Critère d'information d'Akaike (AIC)	-268,468
Critère de Hurvich et Tsai (AICC)	-268,394
Critère de Bozdogan (CAIC)	-265,443

Critère bayésien de Schwartz (BIC)	-266,443

Les critères d'informations sont présentés en plus petit, disposant d'un meilleur format.[a]

a. Variable dépendante : Vibrotactile N2 Latency .

Effets fixes

Tests des effets fixes de type III[a]

Source	Ddl du numérateur	Ddl du dénominateur	F	Sig.
Constante	1	56	2953,790	,000
Condition	1	56	1,784	,187
Phase	1	56	,151	,699
Condition * Phase	1	56	,358	,552

a. Variable dépendante : Vibrotactile N2 Latency .

Estimations des effets fixes[a]

Paramètre	Estimation	Erreur standard	ddl	t	Sig.
Constante	,131867	,005069	56	26,013	,000
[Condition=Sham]	,009804	,007169	56	1,368	,177
[Condition=taVNS]	0[b]	0	.	.	.
[Phase=OFF]	,005000	,007169	56	,697	,488
[Phase=ON]	0[b]	0	.	.	.
[Condition=Sham] * [Phase=OFF]	-,006066	,010138	56	-,598	,552
[Condition=Sham] * [Phase=ON]	0[b]	0	.	.	.
[Condition=taVNS] * [Phase=OFF]	0[b]	0	.	.	.
[Condition=taVNS] * [Phase=ON]	0[b]	0	.	.	.

Estimations des effets fixes[a]

Paramètre	Intervalle de confiance à 95 %	
	Borne inférieure	Borne supérieure
Constante	,121712	,142021
[Condition=Sham]	-,004557	,024165
[Condition=taVNS]	.	.

[Phase=OFF]	-,009361	,019361
[Phase=ON]	.	.
[Condition=Sham] * [Phase=OFF]	-,026376	,014244
[Condition=Sham] * [Phase=ON]	.	.
[Condition=taVNS] * [Phase=OFF]	.	.
[Condition=taVNS] * [Phase=ON]	.	.

a. Variable dépendante : Vibrotactile N2 Latency .

b. Ce paramètre est défini sur 0, car il est redondant.

Matrice de corrélation pour les estimations des effets fixes[a]

Paramètre	Constante	[Condition=Sham]	[Condition=taVNS]	[Phase=OFF]
Constante	1	-,707	.[b]	-,707
[Condition=Sham]	-,707	1	.[b]	,500
[Condition=taVNS]	.[b]	.[b]	.[b]	.[b]
[Phase=OFF]	-,707	,500	.[b]	1
[Phase=ON]	.[b]	.[b]	.[b]	.[b]
[Condition=Sham] * [Phase=OFF]	,500	-,707	.[b]	-,707
[Condition=Sham] * [Phase=ON]	.[b]	.[b]	.[b]	.[b]
[Condition=taVNS] * [Phase=OFF]	.[b]	.[b]	.[b]	.[b]
[Condition=taVNS] * [Phase=ON]	.[b]	.[b]	.[b]	.[b]

Matrice de corrélation pour les estimations des effets fixes[a]

Paramètre	[Phase=ON]	[Condition=Sham] * [Phase=OFF]	[Condition=Sham] * [Phase=ON]	[Condition=taVNS] * [Phase=OFF]
Constante	.[b]	,500	.[b]	.[b]
[Condition=Sham]	.[b]	-,707	.[b]	.[b]
[Condition=taVNS]	.[b]	.[b]	.[b]	.[b]
[Phase=OFF]	.[b]	-,707	.[b]	.[b]
[Phase=ON]	.[b]	.[b]	.[b]	.[b]
[Condition=Sham] * [Phase=OFF]	.[b]	1	.[b]	.[b]
[Condition=Sham] * [Phase=ON]	.[b]	.[b]	.[b]	.[b]
[Condition=taVNS] * [Phase=OFF]	.[b]	.[b]	.[b]	.[b]
[Condition=taVNS] * [Phase=ON]	.[b]	.[b]	.[b]	.[b]

Matrice de corrélation pour les estimations des effets fixes[a]

Paramètre	[Condition=taVNS] * [Phase=ON]
Constante	.[b]
[Condition=Sham]	.[b]
[Condition=taVNS]	.[b]
[Phase=OFF]	.[b]
[Phase=ON]	.[b]
[Condition=Sham] * [Phase=OFF]	.[b]
[Condition=Sham] * [Phase=ON]	.[b]
[Condition=taVNS] * [Phase=OFF]	.[b]
[Condition=taVNS] * [Phase=ON]	.[b]

a. Variable dépendante : Vibrotactile N2 Latency .

b. La corrélation est manquante par défaut, car elle est associée à un paramètre redondant.

Matrice de covariance pour les estimations des effets fixes[a]

Paramètre	Constante	[Condition=Sham]	[Condition=taVNS]	[Phase=OFF]
Constante	2,569670E-5	-2,569670E-5	0[b]	-2,569670E-5
[Condition=Sham]	-2,569670E-5	5,139339E-5	0[b]	2,569670E-5
[Condition=taVNS]	0[b]	0[b]	0[b]	0[b]
[Phase=OFF]	-2,569670E-5	2,569670E-5	0[b]	5,139339E-5
[Phase=ON]	0[b]	0[b]	0[b]	0[b]
[Condition=Sham] * [Phase=OFF]	2,569670E-5	-5,139339E-5	0[b]	-5,139339E-5
[Condition=Sham] * [Phase=ON]	0[b]	0[b]	0[b]	0[b]
[Condition=taVNS] * [Phase=OFF]	0[b]	0[b]	0[b]	0[b]
[Condition=taVNS] * [Phase=ON]	0[b]	0[b]	0[b]	0[b]

Matrice de covariance pour les estimations des effets fixes[a]

Paramètre	[Phase=ON]	[Condition=Sham] * [Phase=OFF]	[Condition=Sham] * [Phase=ON]	[Condition=taVNS] * [Phase=OFF]
Constante	0[b]	2,569670E-5	0[b]	0[b]
[Condition=Sham]	0[b]	-5,139339E-5	0[b]	0[b]
[Condition=taVNS]	0[b]	0[b]	0[b]	0[b]
[Phase=OFF]	0[b]	-5,139339E-5	0[b]	0[b]
[Phase=ON]	0[b]	0[b]	0[b]	0[b]
[Condition=Sham] * [Phase=OFF]	0[b]	,000103	0[b]	0[b]

[Condition=Sham] * [Phase=ON]	0[b]	0[b]	0[b]	0[b]
[Condition=taVNS] * [Phase=OFF]	0[b]	0[b]	0[b]	0[b]
[Condition=taVNS] * [Phase=ON]	0[b]	0[b]	0[b]	0[b]

Matrice de covariance pour les estimations des effets fixes[a]

Paramètre	[Condition=taVNS] * [Phase=ON]
Constante	0[b]
[Condition=Sham]	0[b]
[Condition=taVNS]	0[b]
[Phase=OFF]	0[b]
[Phase=ON]	0[b]
[Condition=Sham] * [Phase=OFF]	0[b]
[Condition=Sham] * [Phase=ON]	0[b]
[Condition=taVNS] * [Phase=OFF]	0[b]
[Condition=taVNS] * [Phase=ON]	0[b]

a. Variable dépendante : Vibrotactile N2 Latency .

b. La covariance est définie sur 0, car elle est associée à un paramètre redondant.

Paramètres de covariance

Estimations des paramètres de covariance[a]

Paramètre	Estimation	Erreur standard	Z de Wald	Sig.	Intervalle de confiance à 95 % Borne inférieure	Borne supérieure
Résidu	,000385	7,284328E-5	5,292	,000	,000266	,000558

a. Variable dépendante : Vibrotactile N2 Latency .

Matrice de corrélation pour les estimations des paramètres de covariance[a]

Paramètre	Résidu
Résidu	1

a. Variable dépendante : Vibrotactile N2 Latency .

Matrice de covariance pour les estimations des paramètres de covariance[a]

Paramètre	Résidu
Résidu	5,306144E-9

a. Variable dépendante : Vibrotactile N2 Latency .

Moyenne marginale estimée

1. Grand Mean[a]

Moyenne	Erreur standard	ddl	Intervalle de confiance à 95 % Borne inférieure	Intervalle de confiance à 95 % Borne supérieure
,138	,003	56	,133	,143

a. Variable dépendante : Vibrotactile N2 Latency .

2. Condition

Estimations[a]

Condition	Moyenne	Erreur standard	ddl	Intervalle de confiance à 95 % Borne inférieure	Intervalle de confiance à 95 % Borne supérieure
Sham	,141	,004	56	,134	,148
taVNS	,134	,004	56	,127	,142

a. Variable dépendante : Vibrotactile N2 Latency .

Comparaisons appariées[a]

(I) Condition	(J) Condition	Différence moyenne (I-J)	Erreur standard	ddl	Sig.[b]
Sham	taVNS	,007	,005	56	,187
taVNS	Sham	-,007	,005	56	,187

Comparaisons appariées[a]

(I) Condition	(J) Condition	Intervalle de confiance à 95 % pour la différence[b] Borne inférieure	Intervalle de confiance à 95 % pour la différence[b] Borne supérieure
Sham	taVNS	-,003	,017
taVNS	Sham	-,017	,003

Basées sur les moyennes marginales estimées[a]

a. Variable dépendante : Vibrotactile N2 Latency .

b. Ajustement pour les comparaisons multiples : Bonferroni.

Tests univariés[a]

Ddl du numérateur	Ddl du dénominateur	F	Sig.
1	56	1,784	,187

Le test de F permet de tester l'effet de Condition. Il s'appuie sur les comparaisons appariées (indépendantes) linéaires parmi les moyennes marginales estimées.[a]

a. Variable dépendante : Vibrotactile N2 Latency .

3. Phase

Estimations[a]

Phase	Moyenne	Erreur standard	ddl	Intervalle de confiance à 95 %	
				Borne inférieure	Borne supérieure
OFF	,139	,004	56	,132	,146
ON	,137	,004	56	,130	,144

a. Variable dépendante : Vibrotactile N2 Latency .

Comparaisons appariées[a]

(I) Phase	(J) Phase	Différence moyenne (I-J)	Erreur standard	ddl	Sig.[b]	Intervalle de confiance à 95 % pour la différence[b] Borne inférieure
OFF	ON	,002	,005	56	,699	-,008
ON	OFF	-,002	,005	56	,699	-,012

Comparaisons appariées[a]

(I) Phase	(J) Phase	Intervalle de confiance à 95 % pour la différence Borne supérieure
OFF	ON	,012
ON	OFF	,008

Basées sur les moyennes marginales estimées[a]

a. Variable dépendante : Vibrotactile N2 Latency .

b. Ajustement pour les comparaisons multiples : Bonferroni.

Tests univariés[a]

Ddl du numérateur	Ddl du dénominateur	F	Sig.
1	56	,151	,699

Le test de F permet de tester l'effet de Phase. Il s'appuie sur les comparaisons appariées (indépendantes) linéaires parmi les moyennes marginales estimées.[a]

a. Variable dépendante : Vibrotactile N2 Latency .

4. Condition * Phase[a]

Condition	Phase	Moyenne	Erreur standard	ddl	Intervalle de confiance à 95 % Borne inférieure	Borne supérieure
Sham	OFF	,141	,005	56	,130	,151
	ON	,142	,005	56	,132	,152
taVNS	OFF	,137	,005	56	,127	,147
	ON	,132	,005	56	,122	,142

a. Variable dépendante : Vibrotactile N2 Latency .

2.2.4. Vibrotactile P2 Amplitude.

```
MIXED VibrotactileP2Amplitude BY Condition Phase
  /CRITERIA=CIN(95) MXITER(100) MXSTEP(10) SCORING(1)
SINGULAR(0.000000000001) HCONVERGE(0,
    ABSOLUTE) LCONVERGE(0, ABSOLUTE) PCONVERGE(0.000001, ABSOLUTE)
  /FIXED=Condition Phase Condition*Phase | SSTYPE(3)
  /METHOD=REML
  /PRINT=CPS CORB COVB DESCRIPTIVES G  SOLUTION TESTCOV
  /EMMEANS=TABLES(OVERALL)
  /EMMEANS=TABLES(Condition) COMPARE ADJ(BONFERRONI)
  /EMMEANS=TABLES(Phase) COMPARE ADJ(BONFERRONI)
  /EMMEANS=TABLES(Condition*Phase) .
```

Remarques

Sortie obtenue		05-MAY-2021 12:31:53
Commentaires		
Entrée	Jeu de données actif	Jeu_de_données2
	Filtre	<sans>
	Pondération	<sans>
	Fichier scindé	<sans>
	N de lignes dans le fichier de travail	60
Gestion des valeurs manquantes	Définition de la valeur manquante	Les valeurs manquantes définies par l'utilisateur sont traitées comme étant manquantes.

	Observations utilisées	Les statistiques sont basées sur toutes les observations comportant des données valides pour toutes les variables du modèle.
Syntaxe		MIXED VibrotactileP2Amplitude BY Condition Phase<br>/CRITERIA=CIN(95) MXITER(100) MXSTEP(10) SCORING(1) SINGULAR(0.000000000001) HCONVERGE(0, ABSOLUTE) LCONVERGE(0, ABSOLUTE) PCONVERGE(0.000001, ABSOLUTE)<br>/FIXED=Condition Phase Condition*Phase | SSTYPE(3)<br>/METHOD=REML<br>/PRINT=CPS CORB COVB DESCRIPTIVES G SOLUTION TESTCOV<br>/EMMEANS=TABLES(OVERALL)<br>/EMMEANS=TABLES(Condition) COMPARE ADJ(BONFERRONI)<br>/EMMEANS=TABLES(Phase) COMPARE ADJ(BONFERRONI)<br>/EMMEANS=TABLES(Condition*Phase) .
Ressources	Temps de processeur	00:00:00,03
	Temps écoulé	00:00:00,02

Récapitulatif de traitement des observations

		Effectif	Pourcentage marginal
Condition	Sham	30	50,0%
	taVNS	30	50,0%
Phase	OFF	30	50,0%
	ON	30	50,0%
Valide		60	100,0%
Exclues		0	
Total		60	

Statistiques descriptives

Vibrotactile P2 Amplitude

Condition	Phase	Effectif	Moyenne	Ecart type	Coefficient de variation
Sham	OFF	15	10,96	4,514	41,2%
	ON	15	10,44	4,352	41,7%
	Total	30	10,70	4,365	40,8%
taVNS	OFF	15	10,13	4,768	47,1%
	ON	15	9,25	5,093	55,1%
	Total	30	9,69	4,868	50,2%
Total	OFF	30	10,54	4,581	43,5%
	ON	30	9,85	4,694	47,7%
	Total	60	10,20	4,612	45,2%

Dimension du modèle[a]

		Nombre de niveaux	Nombre de paramètres
Effets fixes	Constante	1	1
	Condition	2	1
	Phase	2	1
	Condition * Phase	4	1
Résidu			1
Total		9	5

a. Variable dépendante : Vibrotactile P2 Amplitude.

Critères d'information[a]

Log de vraisemblance restreint -2	342,848
Critère d'information d'Akaike (AIC)	344,848
Critère de Hurvich et Tsai (AICC)	344,922
Critère de Bozdogan (CAIC)	347,874
Critère bayésien de Schwartz (BIC)	346,874

Les critères d'informations sont présentés en plus petit, disposant d'un meilleur format.[a]

a. Variable dépendante : Vibrotactile P2 Amplitude.

Effets fixes

Tests des effets fixes de type III[a]

Source	Ddl du numérateur	Ddl du dénominateur	F	Sig.
Constante	1	56	283,494	,000
Condition	1	56	,699	,407
Phase	1	56	,331	,567
Condition * Phase	1	56	,022	,882

a. Variable dépendante : Vibrotactile P2 Amplitude.

Estimations des effets fixes[a]

Paramètre	Estimation	Erreur standard	ddl	t	Sig.
Constante	9,250687	1,211022	56	7,639	,000
[Condition=Sham]	1,192047	1,712644	56	,696	,489
[Condition=taVNS]	0[b]	0	.	.	.
[Phase=OFF]	,876753	1,712644	56	,512	,611
[Phase=ON]	0[b]	0	.	.	.
[Condition=Sham] * [Phase=OFF]	-,359720	2,422044	56	-,149	,882
[Condition=Sham] * [Phase=ON]	0[b]	0	.	.	.

[Condition=taVNS] * [Phase=OFF]	0[b]	0	.	.	.
[Condition=taVNS] * [Phase=ON]	0[b]	0	.	.	.

Estimations des effets fixes[a]

Paramètre	Intervalle de confiance à 95 % Borne inférieure	Borne supérieure
Constante	6,824718	11,676656
[Condition=Sham]	-2,238792	4,622885
[Condition=taVNS]	.	.
[Phase=OFF]	-2,554085	4,307592
[Phase=ON]	.	.
[Condition=Sham] * [Phase=OFF]	-5,211658	4,492218
[Condition=Sham] * [Phase=ON]	.	.
[Condition=taVNS] * [Phase=OFF]	.	.
[Condition=taVNS] * [Phase=ON]	.	.

a. Variable dépendante : Vibrotactile P2 Amplitude.

b. Ce paramètre est défini sur 0, car il est redondant.

Matrice de corrélation pour les estimations des effets fixes[a]

Paramètre	Constante	[Condition=Sham]	[Condition=taVNS]	[Phase=OFF]
Constante	1	-,707	.[b]	-,707
[Condition=Sham]	-,707	1	.[b]	,500
[Condition=taVNS]	.[b]	.[b]	.[b]	.[b]
[Phase=OFF]	-,707	,500	.[b]	1
[Phase=ON]	.[b]	.[b]	.[b]	.[b]
[Condition=Sham] * [Phase=OFF]	,500	-,707	.[b]	-,707
[Condition=Sham] * [Phase=ON]	.[b]	.[b]	.[b]	.[b]
[Condition=taVNS] * [Phase=OFF]	.[b]	.[b]	.[b]	.[b]
[Condition=taVNS] * [Phase=ON]	.[b]	.[b]	.[b]	.[b]

Matrice de corrélation pour les estimations des effets fixes[a]

Paramètre	[Phase=ON]	[Condition=Sham] * [Phase=OFF]	[Condition=Sham] * [Phase=ON]	[Condition=taVNS] * [Phase=OFF]
Constante	.[b]	,500	.[b]	.[b]

[Condition=Sham]	.[b]	-,707	.[b]	.[b]
[Condition=taVNS]	.[b]	.[b]	.[b]	.[b]
[Phase=OFF]	.[b]	-,707	.[b]	.[b]
[Phase=ON]	.[b]	.[b]	.[b]	.[b]
[Condition=Sham] * [Phase=OFF]	.[b]	1	.[b]	.[b]
[Condition=Sham] * [Phase=ON]	.[b]	.[b]	.[b]	.[b]
[Condition=taVNS] * [Phase=OFF]	.[b]	.[b]	.[b]	.[b]
[Condition=taVNS] * [Phase=ON]	.[b]	.[b]	.[b]	.[b]

Matrice de corrélation pour les estimations des effets fixes[a]

Paramètre	[Condition=taVNS] * [Phase=ON]
Constante	.[b]
[Condition=Sham]	.[b]
[Condition=taVNS]	.[b]
[Phase=OFF]	.[b]
[Phase=ON]	.[b]
[Condition=Sham] * [Phase=OFF]	.[b]
[Condition=Sham] * [Phase=ON]	.[b]
[Condition=taVNS] * [Phase=OFF]	.[b]
[Condition=taVNS] * [Phase=ON]	.[b]

a. Variable dépendante : Vibrotactile P2 Amplitude.

b. La corrélation est manquante par défaut, car elle est associée à un paramètre redondant.

Matrice de covariance pour les estimations des effets fixes[a]

Paramètre	Constante	[Condition=Sham]	[Condition=taVNS]	[Phase=OFF]
Constante	1,466575	-1,466575	0[b]	-1,466575
[Condition=Sham]	-1,466575	2,933150	0[b]	1,466575
[Condition=taVNS]	0[b]	0[b]	0[b]	0[b]
[Phase=OFF]	-1,466575	1,466575	0[b]	2,933150
[Phase=ON]	0[b]	0[b]	0[b]	0[b]
[Condition=Sham] * [Phase=OFF]	1,466575	-2,933150	0[b]	-2,933150
[Condition=Sham] * [Phase=ON]	0[b]	0[b]	0[b]	0[b]
[Condition=taVNS] * [Phase=OFF]	0[b]	0[b]	0[b]	0[b]

[Condition=taVNS] * [Phase=ON]	0[b]	0[b]	0[b]	0[b]

Matrice de covariance pour les estimations des effets fixes[a]

Paramètre	[Phase=ON]	[Condition=Sham] * [Phase=OFF]	[Condition=Sham] * [Phase=ON]	[Condition=taVNS] * [Phase=OFF]
Constante	0[b]	1,466575	0[b]	0[b]
[Condition=Sham]	0[b]	-2,933150	0[b]	0[b]
[Condition=taVNS]	0[b]	0[b]	0[b]	0[b]
[Phase=OFF]	0[b]	-2,933150	0[b]	0[b]
[Phase=ON]	0[b]	0[b]	0[b]	0[b]
[Condition=Sham] * [Phase=OFF]	0[b]	5,866299	0[b]	0[b]
[Condition=Sham] * [Phase=ON]	0[b]	0[b]	0[b]	0[b]
[Condition=taVNS] * [Phase=OFF]	0[b]	0[b]	0[b]	0[b]
[Condition=taVNS] * [Phase=ON]	0[b]	0[b]	0[b]	0[b]

Matrice de covariance pour les estimations des effets fixes[a]

Paramètre	[Condition=taVNS] * [Phase=ON]
Constante	0[b]
[Condition=Sham]	0[b]
[Condition=taVNS]	0[b]
[Phase=OFF]	0[b]
[Phase=ON]	0[b]
[Condition=Sham] * [Phase=OFF]	0[b]
[Condition=Sham] * [Phase=ON]	0[b]
[Condition=taVNS] * [Phase=OFF]	0[b]
[Condition=taVNS] * [Phase=ON]	0[b]

a. Variable dépendante : Vibrotactile P2 Amplitude.

b. La covariance est définie sur 0, car elle est associée à un paramètre redondant.

Paramètres de covariance

Estimations des paramètres de covariance[a]

Paramètre	Estimation	Erreur standard	Z de Wald	Sig.	Intervalle de confiance à 95 %	
					Borne inférieure	Borne supérieure
Résidu	21,998623	4,157349	5,292	,000	15,189152	31,860858

a. Variable dépendante : Vibrotactile P2 Amplitude.

Matrice de corrélation pour les estimations des paramètres de covariance[a]

Paramètre	Résidu
Résidu	1

a. Variable dépendante : Vibrotactile P2 Amplitude.

Matrice de covariance pour les estimations des paramètres de covariance[a]

Paramètre	Résidu
Résidu	17,283550

a. Variable dépendante : Vibrotactile P2 Amplitude.

Moyenne marginale estimée

1. Grand Mean[a]

			Intervalle de confiance à 95 %	
Moyenne	Erreur standard	ddl	Borne inférieure	Borne supérieure
10,195	,606	56	8,982	11,408

a. Variable dépendante : Vibrotactile P2 Amplitude.

2. Condition

Estimations[a]

				Intervalle de confiance à 95 %	
Condition	Moyenne	Erreur standard	ddl	Borne inférieure	Borne supérieure
Sham	10,701	,856	56	8,986	12,417
taVNS	9,689	,856	56	7,974	11,404

a. Variable dépendante : Vibrotactile P2 Amplitude.

Comparaisons appariées[a]

(I) Condition	(J) Condition	Différence moyenne (I-J)	Erreur standard	ddl	Sig.[b]
Sham	taVNS	1,012	1,211	56	,407
taVNS	Sham	-1,012	1,211	56	,407

Comparaisons appariées[a]

(I) Condition	(J) Condition	Intervalle de confiance à 95 % pour la différence[b] Borne inférieure	Borne supérieure
Sham	taVNS	-1,414	3,438
taVNS	Sham	-3,438	1,414

Basées sur les moyennes marginales estimées[a]

a. Variable dépendante : Vibrotactile P2 Amplitude.

b. Ajustement pour les comparaisons multiples : Bonferroni.

Tests univariés[a]

Ddl du numérateur	Ddl du dénominateur	F	Sig.
1	56	,699	,407

Le test de F permet de tester l'effet de Condition. Il s'appuie sur les comparaisons appariées (indépendantes) linéaires parmi les moyennes marginales estimées.[a]

a. Variable dépendante : Vibrotactile P2 Amplitude.

3. Phase

Estimations[a]

Phase	Moyenne	Erreur standard	ddl	Intervalle de confiance à 95 % Borne inférieure	Borne supérieure
OFF	10,544	,856	56	8,828	12,259
ON	9,847	,856	56	8,131	11,562

a. Variable dépendante : Vibrotactile P2 Amplitude.

Comparaisons appariées[a]

(I) Phase	(J) Phase	Différence moyenne (I-J)	Erreur standard	ddl	Sig.[b]	Intervalle de confiance à 95 % pour la différence[b] Borne inférieure
OFF	ON	,697	1,211	56	,567	-1,729
ON	OFF	-,697	1,211	56	,567	-3,123

Comparaisons appariées[a]

(I) Phase	(J) Phase	Intervalle de confiance à 95 % pour la différence Borne supérieure
OFF	ON	3,123
ON	OFF	1,729

Basées sur les moyennes marginales estimées[a]

a. Variable dépendante : Vibrotactile P2 Amplitude.

b. Ajustement pour les comparaisons multiples : Bonferroni.

Tests univariés[a]

Ddl du numérateur	Ddl du dénominateur	F	Sig.
1	56	,331	,567

Le test de F permet de tester l'effet de Phase. Il s'appuie sur les comparaisons appariées (indépendantes) linéaires parmi les moyennes marginales estimées.[a]

a. Variable dépendante : Vibrotactile P2 Amplitude.

4. Condition * Phase[a]

Condition	Phase	Moyenne	Erreur standard	ddl	Intervalle de confiance à 95 % Borne inférieure	Intervalle de confiance à 95 % Borne supérieure
Sham	OFF	10,960	1,211	56	8,534	13,386
	ON	10,443	1,211	56	8,017	12,869
taVNS	OFF	10,127	1,211	56	7,701	12,553
	ON	9,251	1,211	56	6,825	11,677

a. Variable dépendante : Vibrotactile P2 Amplitude.

2.2.5. Vibrotactile P2 Latency.

```
MIXED VibrotactileP2Latency BY Condition Phase
  /CRITERIA=CIN(95) MXITER(100) MXSTEP(10) SCORING(1)
SINGULAR(0.000000000001) HCONVERGE(0,
    ABSOLUTE) LCONVERGE(0, ABSOLUTE) PCONVERGE(0.000001, ABSOLUTE)
  /FIXED=Condition Phase Condition*Phase | SSTYPE(3)
  /METHOD=REML
  /PRINT=CPS CORB COVB DESCRIPTIVES G  SOLUTION TESTCOV
  /EMMEANS=TABLES(OVERALL)
  /EMMEANS=TABLES(Condition) COMPARE ADJ(BONFERRONI)
  /EMMEANS=TABLES(Phase) COMPARE ADJ(BONFERRONI)
  /EMMEANS=TABLES(Condition*Phase) .
```

Remarques

Sortie obtenue		05-MAY-2021 12:32:19
Commentaires		
Entrée	Jeu de données actif	Jeu_de_données2
	Filtre	<sans>
	Pondération	<sans>
	Fichier scindé	<sans>

	N de lignes dans le fichier de travail	60
Gestion des valeurs manquantes	Définition de la valeur manquante	Les valeurs manquantes définies par l'utilisateur sont traitées comme étant manquantes.
	Observations utilisées	Les statistiques sont basées sur toutes les observations comportant des données valides pour toutes les variables du modèle.

Syntaxe		MIXED VibrotactileP2Latency BY Condition Phase<br>/CRITERIA=CIN(95) MXITER(100) MXSTEP(10) SCORING(1) SINGULAR(0.000000000001) HCONVERGE(0, ABSOLUTE) LCONVERGE(0, ABSOLUTE) PCONVERGE(0.000001, ABSOLUTE)<br>/FIXED=Condition Phase Condition*Phase | SSTYPE(3)<br>/METHOD=REML<br>/PRINT=CPS CORB COVB DESCRIPTIVES G SOLUTION TESTCOV<br>/EMMEANS=TABLES(OVERALL)<br>/EMMEANS=TABLES(Condition) COMPARE ADJ(BONFERRONI)<br>/EMMEANS=TABLES(Phase) COMPARE ADJ(BONFERRONI)<br>/EMMEANS=TABLES(Condition*Phase) .
Ressources	Temps de processeur	00:00:00,02
	Temps écoulé	00:00:00,02

Récapitulatif de traitement des observations

		Effectif	Pourcentage marginal
Condition	Sham	30	50,0%
	taVNS	30	50,0%

Phase	OFF	30	50,0%
	ON	30	50,0%
Valide		60	100,0%
Exclues		0	
Total		60	

Statistiques descriptives

Vibrotactile P2 Latency

Condition	Phase	Effectif	Moyenne	Ecart type	Coefficient de variation
Sham	OFF	15	,29	,060	21,1%
	ON	15	,28	,063	22,8%
	Total	30	,28	,061	21,6%
taVNS	OFF	15	,28	,049	17,6%
	ON	15	,29	,043	15,2%
	Total	30	,28	,046	16,2%
Total	OFF	30	,28	,054	19,2%
	ON	30	,28	,054	19,0%
	Total	60	,28	,053	18,9%

Dimension du modèle[a]

		Nombre de niveaux	Nombre de paramètres
Effets fixes	Constante	1	1
	Condition	2	1
	Phase	2	1
	Condition * Phase	4	1
Résidu			1
Total		9	5

a. Variable dépendante : Vibrotactile P2 Latency .

Critères d'information[a]

Log de vraisemblance restreint -2	-155,883
Critère d'information d'Akaike (AIC)	-153,883
Critère de Hurvich et Tsai (AICC)	-153,809
Critère de Bozdogan (CAIC)	-150,858

Critère bayésien de Schwartz (BIC)	-151,858

Les critères d'informations sont présentés en plus petit, disposant d'un meilleur format.[a]

a. Variable dépendante : Vibrotactile P2 Latency .

Effets fixes

Tests des effets fixes de type III[a]

Source	Ddl du numérateur	Ddl du dénominateur	F	Sig.
Constante	1	56	1602,595	,000
Condition	1	56	,006	,938
Phase	1	56	,001	,979
Condition * Phase	1	56	,352	,555

a. Variable dépendante : Vibrotactile P2 Latency .

Estimations des effets fixes[a]

Paramètre	Estimation	Erreur standard	ddl	t	Sig.
Constante	,286800	,014101	56	20,339	,000
[Condition=Sham]	-,009461	,019942	56	-,474	,637
[Condition=taVNS]	0[b]	0	.	.	.
[Phase=OFF]	-,008000	,019942	56	-,401	,690
[Phase=ON]	0[b]	0	.	.	.
[Condition=Sham] * [Phase=OFF]	,016733	,028202	56	,593	,555
[Condition=Sham] * [Phase=ON]	0[b]	0	.	.	.
[Condition=taVNS] * [Phase=OFF]	0[b]	0	.	.	.
[Condition=taVNS] * [Phase=ON]	0[b]	0	.	.	.

Estimations des effets fixes[a]

Paramètre	Intervalle de confiance à 95 % Borne inférieure	Borne supérieure
Constante	,258552	,315048
[Condition=Sham]	-,049410	,030488

[Condition=taVNS]	.	.
[Phase=OFF]	-,047949	,031949
[Phase=ON]	.	.
[Condition=Sham] * [Phase=OFF]	-,039763	,073230
[Condition=Sham] * [Phase=ON]	.	.
[Condition=taVNS] * [Phase=OFF]	.	.
[Condition=taVNS] * [Phase=ON]	.	.

a. Variable dépendante : Vibrotactile P2 Latency .

b. Ce paramètre est défini sur 0, car il est redondant.

Matrice de corrélation pour les estimations des effets fixes[a]

Paramètre	Constante	[Condition=Sham]	[Condition=taVNS]	[Phase=OFF]
Constante	1	-,707	.[b]	-,707
[Condition=Sham]	-,707	1	.[b]	,500
[Condition=taVNS]	.[b]	.[b]	.[b]	.[b]
[Phase=OFF]	-,707	,500	.[b]	1
[Phase=ON]	.[b]	.[b]	.[b]	.[b]
[Condition=Sham] * [Phase=OFF]	,500	-,707	.[b]	-,707
[Condition=Sham] * [Phase=ON]	.[b]	.[b]	.[b]	.[b]
[Condition=taVNS] * [Phase=OFF]	.[b]	.[b]	.[b]	.[b]
[Condition=taVNS] * [Phase=ON]	.[b]	.[b]	.[b]	.[b]

Matrice de corrélation pour les estimations des effets fixes[a]

Paramètre	[Phase=ON]	[Condition=Sham] * [Phase=OFF]	[Condition=Sham] * [Phase=ON]	[Condition=taVNS] * [Phase=OFF]
Constante	.[b]	,500	.[b]	.[b]
[Condition=Sham]	.[b]	-,707	.[b]	.[b]
[Condition=taVNS]	.[b]	.[b]	.[b]	.[b]
[Phase=OFF]	.[b]	-,707	.[b]	.[b]
[Phase=ON]	.[b]	.[b]	.[b]	.[b]
[Condition=Sham] * [Phase=OFF]	.[b]	1	.[b]	.[b]
[Condition=Sham] * [Phase=ON]	.[b]	.[b]	.[b]	.[b]
[Condition=taVNS] * [Phase=OFF]	.[b]	.[b]	.[b]	.[b]

[Condition=taVNS] * [Phase=ON]	.[b]	.[b]	.[b]	.[b]

Matrice de corrélation pour les estimations des effets fixes[a]

Paramètre	[Condition=taVNS] * [Phase=ON]
Constante	.[b]
[Condition=Sham]	.[b]
[Condition=taVNS]	.[b]
[Phase=OFF]	.[b]
[Phase=ON]	.[b]
[Condition=Sham] * [Phase=OFF]	.[b]
[Condition=Sham] * [Phase=ON]	.[b]
[Condition=taVNS] * [Phase=OFF]	.[b]
[Condition=taVNS] * [Phase=ON]	.[b]

a. Variable dépendante : Vibrotactile P2 Latency .

b. La corrélation est manquante par défaut, car elle est associée à un paramètre redondant.

Matrice de covariance pour les estimations des effets fixes[a]

Paramètre	Constante	[Condition=Sham]	[Condition=taVNS]	[Phase=OFF]
Constante	,000199	-,000199	0[b]	-,000199
[Condition=Sham]	-,000199	,000398	0[b]	,000199
[Condition=taVNS]	0[b]	0[b]	0[b]	0[b]
[Phase=OFF]	-,000199	,000199	0[b]	,000398
[Phase=ON]	0[b]	0[b]	0[b]	0[b]
[Condition=Sham] * [Phase=OFF]	,000199	-,000398	0[b]	-,000398
[Condition=Sham] * [Phase=ON]	0[b]	0[b]	0[b]	0[b]
[Condition=taVNS] * [Phase=OFF]	0[b]	0[b]	0[b]	0[b]
[Condition=taVNS] * [Phase=ON]	0[b]	0[b]	0[b]	0[b]

Matrice de covariance pour les estimations des effets fixes[a]

Paramètre	[Phase=ON]	[Condition=Sham] * [Phase=OFF]	[Condition=Sham] * [Phase=ON]	[Condition=taVNS] * [Phase=OFF]
Constante	0[b]	,000199	0[b]	0[b]
[Condition=Sham]	0[b]	-,000398	0[b]	0[b]
[Condition=taVNS]	0[b]	0[b]	0[b]	0[b]
[Phase=OFF]	0[b]	-,000398	0[b]	0[b]
[Phase=ON]	0[b]	0[b]	0[b]	0[b]

[Condition=Sham] * [Phase=OFF]	0[b]	,000795	0[b]	0[b]
[Condition=Sham] * [Phase=ON]	0[b]	0[b]	0[b]	0[b]
[Condition=taVNS] * [Phase=OFF]	0[b]	0[b]	0[b]	0[b]
[Condition=taVNS] * [Phase=ON]	0[b]	0[b]	0[b]	0[b]

Matrice de covariance pour les estimations des effets fixes[a]

Paramètre	[Condition=taVNS] * [Phase=ON]
Constante	0[b]
[Condition=Sham]	0[b]
[Condition=taVNS]	0[b]
[Phase=OFF]	0[b]
[Phase=ON]	0[b]
[Condition=Sham] * [Phase=OFF]	0[b]
[Condition=Sham] * [Phase=ON]	0[b]
[Condition=taVNS] * [Phase=OFF]	0[b]
[Condition=taVNS] * [Phase=ON]	0[b]

a. Variable dépendante : Vibrotactile P2 Latency .

b. La covariance est définie sur 0, car elle est associée à un paramètre redondant.

Paramètres de covariance

Estimations des paramètres de covariance[a]

					Intervalle de confiance à 95 %	
Paramètre	Estimation	Erreur standard	Z de Wald	Sig.	Borne inférieure	Borne supérieure
Résidu	,002983	,000564	5,292	,000	,002059	,004320

a. Variable dépendante : Vibrotactile P2 Latency .

Matrice de corrélation pour les estimations des paramètres de covariance[a]

Paramètre	Résidu
Résidu	1

a. Variable dépendante : Vibrotactile P2 Latency .

Matrice de covariance pour les estimations des paramètres de covariance[a]

Paramètre	Résidu
Résidu	3,177235E-7

a. Variable dépendante : Vibrotactile P2 Latency .

Moyenne marginale estimée

1. Grand Mean[a]

Moyenne	Erreur standard	ddl	Intervalle de confiance à 95 % Borne inférieure	Intervalle de confiance à 95 % Borne supérieure
,282	,007	56	,268	,296

a. Variable dépendante : Vibrotactile P2 Latency .

2. Condition

Estimations[a]

Condition	Moyenne	Erreur standard	ddl	Intervalle de confiance à 95 % Borne inférieure	Intervalle de confiance à 95 % Borne supérieure
Sham	,282	,010	56	,262	,302
taVNS	,283	,010	56	,263	,303

a. Variable dépendante : Vibrotactile P2 Latency .

Comparaisons appariées[a]

(I) Condition	(J) Condition	Différence moyenne (I-J)	Erreur standard	ddl	Sig.[b]
Sham	taVNS	-,001	,014	56	,938
taVNS	Sham	,001	,014	56	,938

Comparaisons appariées[a]

(I) Condition	(J) Condition	Intervalle de confiance à 95 % pour la différence[b] Borne inférieure	Intervalle de confiance à 95 % pour la différence[b] Borne supérieure
Sham	taVNS	-,029	,027
taVNS	Sham	-,027	,029

Basées sur les moyennes marginales estimées[a]

a. Variable dépendante : Vibrotactile P2 Latency .

b. Ajustement pour les comparaisons multiples : Bonferroni.

Tests univariés[a]

Ddl du numérateur	Ddl du dénominateur	F	Sig.
1	56	,006	,938

Le test de F permet de tester l'effet de Condition. Il s'appuie sur les comparaisons appariées (indépendantes) linéaires parmi les moyennes marginales estimées.[a]

a. Variable dépendante : Vibrotactile P2 Latency .

3. Phase

Estimations[a]

Phase	Moyenne	Erreur standard	ddl	Intervalle de confiance à 95 % Borne inférieure	Intervalle de confiance à 95 % Borne supérieure
OFF	,282	,010	56	,262	,302
ON	,282	,010	56	,262	,302

a. Variable dépendante : Vibrotactile P2 Latency .

Comparaisons appariées[a]

(I) Phase	(J) Phase	Différence moyenne (I-J)	Erreur standard	ddl	Sig.[b]	Intervalle de confiance à 95 % pour la différence[b] Borne inférieure
OFF	ON	,000	,014	56	,979	-,028
ON	OFF	,000	,014	56	,979	-,029

Comparaisons appariées[a]

(I) Phase	(J) Phase	Intervalle de confiance à 95 % pour la différence Borne supérieure
OFF	ON	,029
ON	OFF	,028

Basées sur les moyennes marginales estimées[a]

a. Variable dépendante : Vibrotactile P2 Latency .

b. Ajustement pour les comparaisons multiples : Bonferroni.

Tests univariés[a]

Ddl du numérateur	Ddl du dénominateur	F	Sig.
1	56	,001	,979

Le test de F permet de tester l'effet de Phase. Il s'appuie sur les comparaisons appariées (indépendantes) linéaires parmi les moyennes marginales estimées.[a]

a. Variable dépendante : Vibrotactile P2 Latency .

4. Condition * Phase[a]

					Intervalle de confiance à 95 %	
Condition	Phase	Moyenne	Erreur standard	ddl	Borne inférieure	Borne supérieure
Sham	OFF	,286	,014	56	,258	,314
	ON	,277	,014	56	,249	,306
taVNS	OFF	,279	,014	56	,251	,307
	ON	,287	,014	56	,259	,315

a. Variable dépendante : Vibrotactile P2 Latency .

3. Experiment 3

3.1. Laser-evoked potentials (LEPs)

3.1.1. LEPs N2P2 Amplitude.

```
MIXED LaserN2P2Amplitude BY Phase
  /CRITERIA=CIN(95) MXITER(100) MXSTEP(10) SCORING(1)
SINGULAR(0.000000000001) HCONVERGE(0,
    ABSOLUTE) LCONVERGE(0, ABSOLUTE) PCONVERGE(0.000001, ABSOLUTE)
  /FIXED=Phase | SSTYPE(3)
  /METHOD=REML
  /PRINT=CPS CORB COVB DESCRIPTIVES G  SOLUTION TESTCOV
  /EMMEANS=TABLES(OVERALL)
  /EMMEANS=TABLES(Phase) COMPARE ADJ(BONFERRONI).
```

Remarques

Sortie obtenue		05-MAY-2021 12:34:23
Commentaires		
Entrée	Jeu de données actif	Jeu_de_données3
	Filtre	<sans>
	Pondération	<sans>
	Fichier scindé	<sans>
	N de lignes dans le fichier de travail	26
Gestion des valeurs manquantes	Définition de la valeur manquante	Les valeurs manquantes définies par l'utilisateur sont traitées comme étant manquantes.
	Observations utilisées	Les statistiques sont basées sur toutes les observations comportant des données valides pour toutes les variables du modèle.

Syntaxe		MIXED LaserN2P2Amplitude BY Phase<br>/CRITERIA=CIN(95) MXITER(100) MXSTEP(10) SCORING(1) SINGULAR(0.000000000001) HCONVERGE(0, ABSOLUTE) LCONVERGE(0, ABSOLUTE) PCONVERGE(0.000001, ABSOLUTE)<br>/FIXED=Phase | SSTYPE(3)<br>/METHOD=REML<br>/PRINT=CPS CORB COVB DESCRIPTIVES G SOLUTION TESTCOV<br>/EMMEANS=TABLES(OVERALL)<br>/EMMEANS=TABLES(Phase) COMPARE ADJ(BONFERRONI).
Ressources	Temps de processeur	00:00:00,00
	Temps écoulé	00:00:00,01

Récapitulatif de traitement des observations

		Effectif	Pourcentage marginal
Phase	OFF	13	50,0%
	ON	13	50,0%
Valide		26	100,0%
Exclues		0	
Total		26	

Statistiques descriptives

Laser N2P2 Amplitude

Phase	Effectif	Moyenne	Ecart type	Coefficient de variation

OFF	13	22,933967461538458	15,618307938159639	68,1%
ON	13	26,847605384615385	12,773666731771929	47,6%
Total	26	24,890786423076920	14,120531019987647	56,7%

Dimension du modèle[a]

		Nombre de niveaux	Nombre de paramètres
Effets fixes	Constante	1	1
	Phase	2	1
Résidu			1
Total		3	3

a. Variable dépendante : Laser N2P2 Amplitude.

Critères d'information[a]

Log de vraisemblance restreint -2	200,821
Critère d'information d'Akaike (AIC)	202,821
Critère de Hurvich et Tsai (AICC)	203,003
Critère de Bozdogan (CAIC)	204,999
Critère bayésien de Schwartz (BIC)	203,999

Les critères d'informations sont présentés en plus petit, disposant d'un meilleur format.[a]

a. Variable dépendante : Laser N2P2 Amplitude.

Effets fixes

Tests des effets fixes de type III[a]

Source	Ddl du numérateur	Ddl du dénominateur	F	Sig.
Constante	1	24	79,137	,000
Phase	1	24	,489	,491

a. Variable dépendante : Laser N2P2 Amplitude.

Estimations des effets fixes[a]

Paramètre	Estimation	Erreur standard	ddl	t	Sig.	Intervalle de confiance à 95 % Borne inférieure
Constante	26,847605	3,956971	24	6,785	,000	18,680819
[Phase=OFF]	-3,913638	5,596002	24	-,699	,491	-15,463218
[Phase=ON]	0[b]	0	.	.	.	.

Estimations des effets fixes[a]

Paramètre	Intervalle de confiance à 95 % Borne supérieure
Constante	35,014392
[Phase=OFF]	7,635943
[Phase=ON]	.

a. Variable dépendante : Laser N2P2 Amplitude.

b. Ce paramètre est défini sur 0, car il est redondant.

Matrice de corrélation pour les estimations des effets fixes[a]

Paramètre	Constante	[Phase=OFF]	[Phase=ON]
Constante	1	-,707	.[b]
[Phase=OFF]	-,707	1	.[b]
[Phase=ON]	.[b]	.[b]	.[b]

a. Variable dépendante : Laser N2P2 Amplitude.

b. La corrélation est manquante par défaut, car elle est associée à un paramètre redondant.

Matrice de covariance pour les estimations des effets fixes[a]

Paramètre	Constante	[Phase=OFF]	[Phase=ON]
Constante	15,657619	-15,657619	0[b]
[Phase=OFF]	-15,657619	31,315239	0[b]
[Phase=ON]	0[b]	0[b]	0[b]

a. Variable dépendante : Laser N2P2 Amplitude.

b. La covariance est définie sur 0, car elle est associée à un paramètre redondant.

Paramètres de covariance

Estimations des paramètres de covariance[a]

Paramètre	Estimation	Erreur standard	Z de Wald	Sig.	Intervalle de confiance à 95 % Borne inférieure	Borne supérieure
Résidu	203,549052	58,759550	3,464	,001	115,597479	358,417994

a. Variable dépendante : Laser N2P2 Amplitude.

Matrice de corrélation pour les estimations des paramètres de covariance[a]

Paramètre	Résidu
Résidu	1

a. Variable dépendante : Laser N2P2 Amplitude.

Matrice de covariance pour les estimations des paramètres de covariance[a]

Paramètre	Résidu
Résidu	3452,684725

a. Variable dépendante : Laser N2P2 Amplitude.

Moyenne marginale estimée

1. Grand Mean[a]

Moyenne	Erreur standard	ddl	Intervalle de confiance à 95 % Borne inférieure	Borne supérieure
24,891	2,798	24	19,116	30,666

a. Variable dépendante : Laser N2P2 Amplitude.

2. Phase

Estimations[a]

Phase	Moyenne	Erreur standard	ddl	Intervalle de confiance à 95 % Borne inférieure	Borne supérieure
OFF	22,934	3,957	24	14,767	31,101

ON	26,848	3,957	24	18,681	35,014

a. Variable dépendante : Laser N2P2 Amplitude.

Comparaisons appariées[a]

(I) Phase	(J) Phase	Différence moyenne (I-J)	Erreur standard	ddl	Sig.[b]	Intervalle de confiance à 95 % pour la différence[b] Borne inférieure
OFF	ON	-3,914	5,596	24	,491	-15,463
ON	OFF	3,914	5,596	24	,491	-7,636

Comparaisons appariées[a]

(I) Phase	(J) Phase	Intervalle de confiance à 95 % pour la différence Borne supérieure
OFF	ON	7,636
ON	OFF	15,463

Basées sur les moyennes marginales estimées[a]

a. Variable dépendante : Laser N2P2 Amplitude.

b. Ajustement pour les comparaisons multiples : Bonferroni.

Tests univariés[a]

Ddl du numérateur	Ddl du dénominateur	F	Sig.
1	24	,489	,491

Le test de F permet de tester l'effet de Phase. Il s'appuie sur les comparaisons appariées (indépendantes) linéaires parmi les moyennes marginales estimées.[a]

a. Variable dépendante : Laser N2P2 Amplitude.

3.1.2 LEPs N2 Amplitude.

```
MIXED LaserN2Amplitude BY Phase
  /CRITERIA=CIN(95) MXITER(100) MXSTEP(10) SCORING(1)
SINGULAR(0.000000000001) HCONVERGE(0,
    ABSOLUTE) LCONVERGE(0, ABSOLUTE) PCONVERGE(0.000001, ABSOLUTE)
  /FIXED=Phase | SSTYPE(3)
  /METHOD=REML
  /PRINT=CPS CORB COVB DESCRIPTIVES G  SOLUTION TESTCOV
  /EMMEANS=TABLES(OVERALL)
  /EMMEANS=TABLES(Phase) COMPARE ADJ(BONFERRONI).
```

Remarques

Sortie obtenue		05-MAY-2021 12:35:13
Commentaires		
Entrée	Jeu de données actif	Jeu_de_données3
	Filtre	<sans>
	Pondération	<sans>
	Fichier scindé	<sans>
	N de lignes dans le fichier de travail	26
Gestion des valeurs manquantes	Définition de la valeur manquante	Les valeurs manquantes définies par l'utilisateur sont traitées comme étant manquantes.
	Observations utilisées	Les statistiques sont basées sur toutes les observations comportant des données valides pour toutes les variables du modèle.

Syntaxe		MIXED LaserN2Amplitude BY Phase /CRITERIA=CIN(95) MXITER(100) MXSTEP(10) SCORING(1) SINGULAR(0.000000000001) HCONVERGE(0, ABSOLUTE) LCONVERGE(0, ABSOLUTE) PCONVERGE(0.000001, ABSOLUTE) /FIXED=Phase | SSTYPE(3) /METHOD=REML /PRINT=CPS CORB COVB DESCRIPTIVES G SOLUTION TESTCOV /EMMEANS=TABLES(OVERALL) /EMMEANS=TABLES(Phase) COMPARE ADJ(BONFERRONI).
Ressources	Temps de processeur	00:00:00,00
	Temps écoulé	00:00:00,01

Récapitulatif de traitement des observations

		Effectif	Pourcentage marginal
Phase	OFF	13	50,0%
	ON	13	50,0%
Valide		26	100,0%
Exclues		0	
Total		26	

Statistiques descriptives

Laser N2 Amplitude

Phase	Effectif	Moyenne	Ecart type	Coefficient de variation

OFF	13	-15,08	14,692	-97,5%
ON	13	-17,66	13,346	-75,6%
Total	26	-16,37	13,814	-84,4%

Dimension du modèle[a]

		Nombre de niveaux	Nombre de paramètres
Effets fixes	Constante	1	1
	Phase	2	1
Résidu			1
Total		3	3

a. Variable dépendante : Laser N2 Amplitude.

Critères d'information[a]

Log de vraisemblance restreint -2	200,033
Critère d'information d'Akaike (AIC)	202,033
Critère de Hurvich et Tsai (AICC)	202,215
Critère de Bozdogan (CAIC)	204,212
Critère bayésien de Schwartz (BIC)	203,212

Les critères d'informations sont présentés en plus petit, disposant d'un meilleur format.[a]

a. Variable dépendante : Laser N2 Amplitude.

Effets fixes

Tests des effets fixes de type III[a]

Source	Ddl du numérateur	Ddl du dénominateur	F	Sig.
Constante	1	24	35,362	,000
Phase	1	24	,220	,643

a. Variable dépendante : Laser N2 Amplitude.

Estimations des effets fixes[a]

Paramètre	Estimation	Erreur standard	ddl	t	Sig.	Intervalle de confiance à 95 %
						Borne inférieure
Constante	-17,660444	3,892601	24	-4,537	,000	-25,694378
[Phase=OFF]	2,584959	5,504970	24	,470	,643	-8,776740
[Phase=ON]	0[b]	0	.	.	.	.

Estimations des effets fixes[a]

Paramètre	Intervalle de confiance à 95 %
	Borne supérieure
Constante	-9,626510
[Phase=OFF]	13,946658
[Phase=ON]	.

a. Variable dépendante : Laser N2 Amplitude.

b. Ce paramètre est défini sur 0, car il est redondant.

Matrice de corrélation pour les estimations des effets fixes[a]

Paramètre	Constante	[Phase=OFF]	[Phase=ON]
Constante	1	-,707	.[b]
[Phase=OFF]	-,707	1	.[b]
[Phase=ON]	.[b]	.[b]	.[b]

a. Variable dépendante : Laser N2 Amplitude.

b. La corrélation est manquante par défaut, car elle est associée à un paramètre redondant.

Matrice de covariance pour les estimations des effets fixes[a]

Paramètre	Constante	[Phase=OFF]	[Phase=ON]
Constante	15,152345	-15,152345	0[b]
[Phase=OFF]	-15,152345	30,304690	0[b]
[Phase=ON]	0[b]	0[b]	0[b]

a. Variable dépendante : Laser N2 Amplitude.

b. La covariance est définie sur 0, car elle est associée à un paramètre redondant.

Paramètres de covariance

Estimations des paramètres de covariance[a]

Paramètre	Estimation	Erreur standard	Z de Wald	Sig.	Intervalle de confiance à 95 %	
					Borne inférieure	Borne supérieure
Résidu	196,980487	56,863369	3,464	,001	111,867128	346,851780

a. Variable dépendante : Laser N2 Amplitude.

Matrice de corrélation pour les estimations des paramètres de covariance[a]

Paramètre	Résidu
Résidu	1

a. Variable dépendante : Laser N2 Amplitude.

Matrice de covariance pour les estimations des paramètres de covariance[a]

Paramètre	Résidu
Résidu	3233,442695

a. Variable dépendante : Laser N2 Amplitude.

Moyenne marginale estimée

1. Grand Mean[a]

Moyenne	Erreur standard	ddl	Intervalle de confiance à 95 %	
			Borne inférieure	Borne supérieure
-16,368	2,752	24	-22,049	-10,687

a. Variable dépendante : Laser N2 Amplitude.

2. Phase

Estimations[a]

Phase	Moyenne	Erreur standard	ddl	Intervalle de confiance à 95 %	
				Borne inférieure	Borne supérieure

OFF	-15,075	3,893	24	-23,109	-7,042
ON	-17,660	3,893	24	-25,694	-9,627

a. Variable dépendante : Laser N2 Amplitude.

Comparaisons appariées[a]

(I) Phase	(J) Phase	Différence moyenne (I-J)	Erreur standard	ddl	Sig.[b]	Intervalle de confiance à 95 % pour la différence[b] Borne inférieure
OFF	ON	2,585	5,505	24	,643	-8,777
ON	OFF	-2,585	5,505	24	,643	-13,947

Comparaisons appariées[a]

(I) Phase	(J) Phase	Intervalle de confiance à 95 % pour la différence Borne supérieure
OFF	ON	13,947
ON	OFF	8,777

Basées sur les moyennes marginales estimées[a]

a. Variable dépendante : Laser N2 Amplitude.

b. Ajustement pour les comparaisons multiples : Bonferroni.

Tests univariés[a]

Ddl du numérateur	Ddl du dénominateur	F	Sig.
1	24	,220	,643

Le test de F permet de tester l'effet de Phase. Il s'appuie sur les comparaisons appariées (indépendantes) linéaires parmi les moyennes marginales estimées.[a]

a. Variable dépendante : Laser N2 Amplitude.

3.1.3. LEPs N2 Latency.

```
MIXED LaserN2Latency BY Phase
  /CRITERIA=CIN(95) MXITER(100) MXSTEP(10) SCORING(1)
SINGULAR(0.000000000001) HCONVERGE(0,
    ABSOLUTE) LCONVERGE(0, ABSOLUTE) PCONVERGE(0.000001, ABSOLUTE)
  /FIXED=Phase | SSTYPE(3)
  /METHOD=REML
  /PRINT=CPS CORB COVB DESCRIPTIVES G  SOLUTION TESTCOV
  /EMMEANS=TABLES(OVERALL)
  /EMMEANS=TABLES(Phase) COMPARE ADJ(BONFERRONI).
```

Remarques

Sortie obtenue		05-MAY-2021 12:35:39
Commentaires		
Entrée	Jeu de données actif	Jeu_de_données3
	Filtre	<sans>
	Pondération	<sans>
	Fichier scindé	<sans>
	N de lignes dans le fichier de travail	26
Gestion des valeurs manquantes	Définition de la valeur manquante	Les valeurs manquantes définies par l'utilisateur sont traitées comme étant manquantes.
	Observations utilisées	Les statistiques sont basées sur toutes les observations comportant des données valides pour toutes les variables du modèle.

Syntaxe		MIXED LaserN2Latency BY Phase /CRITERIA=CIN(95) MXITER(100) MXSTEP(10) SCORING(1) SINGULAR(0.000000000001) HCONVERGE(0, ABSOLUTE) LCONVERGE(0, ABSOLUTE) PCONVERGE(0.000001, ABSOLUTE) /FIXED=Phase | SSTYPE(3) /METHOD=REML /PRINT=CPS CORB COVB DESCRIPTIVES G SOLUTION TESTCOV /EMMEANS=TABLES(OVERALL) /EMMEANS=TABLES(Phase) COMPARE ADJ(BONFERRONI).
Ressources	Temps de processeur	00:00:00,00
	Temps écoulé	00:00:00,01

Récapitulatif de traitement des observations

		Effectif	Pourcentage marginal
Phase	OFF	13	50,0%
	ON	13	50,0%
Valide		26	100,0%
Exclues		0	
Total		26	

Statistiques descriptives

Laser N2 Latency

Phase	Effectif	Moyenne	Ecart type	Coefficient de variation

OFF	13	,26	,037	14,5%
ON	13	,26	,072	27,9%
Total	26	,26	,056	21,8%

Dimension du modèle[a]

		Nombre de niveaux	Nombre de paramètres
Effets fixes	Constante	1	1
	Phase	2	1
Résidu			1
Total		3	3

a. Variable dépendante : Laser N2 Latency .

Critères d'information[a]

Log de vraisemblance restreint -2	-63,969
Critère d'information d'Akaike (AIC)	-61,969
Critère de Hurvich et Tsai (AICC)	-61,787
Critère de Bozdogan (CAIC)	-59,790
Critère bayésien de Schwartz (BIC)	-60,790

Les critères d'informations sont présentés en plus petit, disposant d'un meilleur format.[a]

a. Variable dépendante : Laser N2 Latency .

Effets fixes

Tests des effets fixes de type III[a]

Source	Ddl du numérateur	Ddl du dénominateur	F	Sig.
Constante	1	24	523,067	,000
Phase	1	24	,008	,931

a. Variable dépendante : Laser N2 Latency .

Estimations des effets fixes[a]

Paramètre	Estimation	Erreur standard	ddl	t	Sig.	Intervalle de confiance à 95 % Borne inférieure
Constante	,258237	,015908	24	16,234	,000	,225405
[Phase=OFF]	-,001962	,022497	24	-,087	,931	-,048392
[Phase=ON]	0[b]	0	.	.	.	.

Estimations des effets fixes[a]

Paramètre	Intervalle de confiance à 95 % Borne supérieure
Constante	,291068
[Phase=OFF]	,044469
[Phase=ON]	.

a. Variable dépendante : Laser N2 Latency .

b. Ce paramètre est défini sur 0, car il est redondant.

Matrice de corrélation pour les estimations des effets fixes[a]

Paramètre	Constante	[Phase=OFF]	[Phase=ON]
Constante	1	-,707	.[b]
[Phase=OFF]	-,707	1	.[b]
[Phase=ON]	.[b]	.[b]	.[b]

a. Variable dépendante : Laser N2 Latency .

b. La corrélation est manquante par défaut, car elle est associée à un paramètre redondant.

Matrice de covariance pour les estimations des effets fixes[a]

Paramètre	Constante	[Phase=OFF]	[Phase=ON]
Constante	,000253	-,000253	0[b]
[Phase=OFF]	-,000253	,000506	0[b]
[Phase=ON]	0[b]	0[b]	0[b]

a. Variable dépendante : Laser N2 Latency .

b. La covariance est définie sur 0, car elle est associée à un paramètre redondant.

Paramètres de covariance

Estimations des paramètres de covariance[a]

Paramètre	Estimation	Erreur standard	Z de Wald	Sig.	Intervalle de confiance à 95 % Borne inférieure	Borne supérieure
Résidu	,003290	,000950	3,464	,001	,001868	,005793

a. Variable dépendante : Laser N2 Latency .

Matrice de corrélation pour les estimations des paramètres de covariance[a]

Paramètre	Résidu
Résidu	1

a. Variable dépendante : Laser N2 Latency .

Matrice de covariance pour les estimations des paramètres de covariance[a]

Paramètre	Résidu
Résidu	9,018077E-7

a. Variable dépendante : Laser N2 Latency .

Moyenne marginale estimée

1. Grand Mean[a]

Moyenne	Erreur standard	ddl	Intervalle de confiance à 95 % Borne inférieure	Borne supérieure
,257	,011	24	,234	,280

a. Variable dépendante : Laser N2 Latency .

2. Phase

Estimations[a]

Phase	Moyenne	Erreur standard	ddl	Intervalle de confiance à 95 % Borne inférieure	Intervalle de confiance à 95 % Borne supérieure
OFF	,256	,016	24	,223	,289
ON	,258	,016	24	,225	,291

a. Variable dépendante : Laser N2 Latency .

Comparaisons appariées[a]

(I) Phase	(J) Phase	Différence moyenne (I-J)	Erreur standard	ddl	Sig.[b]	Intervalle de confiance à 95 % pour la différence[b] Borne inférieure
OFF	ON	-,002	,022	24	,931	-,048
ON	OFF	,002	,022	24	,931	-,044

Comparaisons appariées[a]

(I) Phase	(J) Phase	Intervalle de confiance à 95 % pour la différence Borne supérieure
OFF	ON	,044
ON	OFF	,048

Basées sur les moyennes marginales estimées[a]

a. Variable dépendante : Laser N2 Latency .

b. Ajustement pour les comparaisons multiples : Bonferroni.

Tests univariés[a]

Ddl du numérateur	Ddl du dénominateur	F	Sig.
1	24	,008	,931

Le test de F permet de tester l'effet de Phase. Il s'appuie sur les comparaisons appariées (indépendantes) linéaires parmi les moyennes marginales estimées.[a]

a. Variable dépendante : Laser N2 Latency .

3.1.4. LEPs P2 Amplitude.

```
MIXED LaserP2Amplitude BY Phase
  /CRITERIA=CIN(95) MXITER(100) MXSTEP(10) SCORING(1)
SINGULAR(0.000000000001) HCONVERGE(0,
    ABSOLUTE) LCONVERGE(0, ABSOLUTE) PCONVERGE(0.000001, ABSOLUTE)
  /FIXED=Phase | SSTYPE(3)
  /METHOD=REML
  /PRINT=CPS CORB COVB DESCRIPTIVES G  SOLUTION TESTCOV
  /EMMEANS=TABLES(OVERALL)
  /EMMEANS=TABLES(Phase) COMPARE ADJ(BONFERRONI).
```

Remarques

Sortie obtenue		05-MAY-2021 12:36:03
Commentaires		
Entrée	Jeu de données actif	Jeu_de_données3
	Filtre	<sans>
	Pondération	<sans>
	Fichier scindé	<sans>
	N de lignes dans le fichier de travail	26
Gestion des valeurs manquantes	Définition de la valeur manquante	Les valeurs manquantes définies par l'utilisateur sont traitées comme étant manquantes.
	Observations utilisées	Les statistiques sont basées sur toutes les observations comportant des données valides pour toutes les variables du modèle.

Syntaxe		MIXED LaserP2Amplitude BY Phase<br>/CRITERIA=CIN(95) MXITER(100) MXSTEP(10) SCORING(1) SINGULAR(0.000000000001) HCONVERGE(0, ABSOLUTE) LCONVERGE(0, ABSOLUTE) PCONVERGE(0.000001, ABSOLUTE)<br>/FIXED=Phase | SSTYPE(3)<br>/METHOD=REML<br>/PRINT=CPS CORB COVB DESCRIPTIVES G SOLUTION TESTCOV<br>/EMMEANS=TABLES(OVERALL)<br>/EMMEANS=TABLES(Phase) COMPARE ADJ(BONFERRONI).
Ressources	Temps de processeur	00:00:00,02
	Temps écoulé	00:00:00,01

Récapitulatif de traitement des observations

		Effectif	Pourcentage marginal
Phase	OFF	13	50,0%
	ON	13	50,0%
Valide		26	100,0%
Exclues		0	
Total		26	

Statistiques descriptives

Laser P2 Amplitude

Phase	Effectif	Moyenne	Ecart type	Coefficient de variation

OFF	13	7,86	8,164	103,9%
ON	13	9,19	4,912	53,5%
Total	26	8,52	6,635	77,9%

Dimension du modèle[a]

		Nombre de niveaux	Nombre de paramètres
Effets fixes	Constante	1	1
	Phase	2	1
Résidu			1
Total		3	3

a. Variable dépendante : Laser P2 Amplitude.

Critères d'information[a]

Log de vraisemblance restreint -2	164,803
Critère d'information d'Akaike (AIC)	166,803
Critère de Hurvich et Tsai (AICC)	166,985
Critère de Bozdogan (CAIC)	168,981
Critère bayésien de Schwartz (BIC)	167,981

Les critères d'informations sont présentés en plus petit, disposant d'un meilleur format.[a]

a. Variable dépendante : Laser P2 Amplitude.

Effets fixes

Tests des effets fixes de type III[a]

Source	Ddl du numérateur	Ddl du dénominateur	F	Sig.
Constante	1	24	41,613	,000
Phase	1	24	,253	,620

a. Variable dépendante : Laser P2 Amplitude.

Estimations des effets fixes[a]

Paramètre	Estimation	Erreur standard	ddl	t	Sig.	Intervalle de confiance à 95 % Borne inférieure
Constante	9,187162	1,868451	24	4,917	,000	5,330868
[Phase=OFF]	-1,328679	2,642389	24	-,503	,620	-6,782301
[Phase=ON]	0[b]	0	.	.	.	.

Estimations des effets fixes[a]

Paramètre	Intervalle de confiance à 95 % Borne supérieure
Constante	13,043455
[Phase=OFF]	4,124943
[Phase=ON]	.

a. Variable dépendante : Laser P2 Amplitude.

b. Ce paramètre est défini sur 0, car il est redondant.

Matrice de corrélation pour les estimations des effets fixes[a]

Paramètre	Constante	[Phase=OFF]	[Phase=ON]
Constante	1	-,707	.[b]
[Phase=OFF]	-,707	1	.[b]
[Phase=ON]	.[b]	.[b]	.[b]

a. Variable dépendante : Laser P2 Amplitude.

b. La corrélation est manquante par défaut, car elle est associée à un paramètre redondant.

Matrice de covariance pour les estimations des effets fixes[a]

Paramètre	Constante	[Phase=OFF]	[Phase=ON]
Constante	3,491109	-3,491109	0[b]
[Phase=OFF]	-3,491109	6,982218	0[b]
[Phase=ON]	0[b]	0[b]	0[b]

a. Variable dépendante : Laser P2 Amplitude.

b. La covariance est définie sur 0, car elle est associée à un paramètre redondant.

Paramètres de covariance

Estimations des paramètres de covariance[a]

Paramètre	Estimation	Erreur standard	Z de Wald	Sig.	Intervalle de confiance à 95 % Borne inférieure	Borne supérieure
Résidu	45,384415	13,101352	3,464	,001	25,774249	79,914845

a. Variable dépendante : Laser P2 Amplitude.

Matrice de corrélation pour les estimations des paramètres de covariance[a]

Paramètre	Résidu
Résidu	1

a. Variable dépendante : Laser P2 Amplitude.

Matrice de covariance pour les estimations des paramètres de covariance[a]

Paramètre	Résidu
Résidu	171,645428

a. Variable dépendante : Laser P2 Amplitude.

Moyenne marginale estimée

1. Grand Mean[a]

Moyenne	Erreur standard	ddl	Intervalle de confiance à 95 % Borne inférieure	Borne supérieure
8,523	1,321	24	5,796	11,250

a. Variable dépendante : Laser P2 Amplitude.

2. Phase

Estimations[a]

Phase	Moyenne	Erreur standard	ddl	Intervalle de confiance à 95 % Borne inférieure	Borne supérieure
OFF	7,858	1,868	24	4,002	11,715

ON	9,187	1,868	24	5,331	13,043

a. Variable dépendante : Laser P2 Amplitude.

Comparaisons appariées[a]

(I) Phase	(J) Phase	Différence moyenne (I-J)	Erreur standard	ddl	Sig.[b]	Intervalle de confiance à 95 % pour la différence[b] Borne inférieure
OFF	ON	-1,329	2,642	24	,620	-6,782
ON	OFF	1,329	2,642	24	,620	-4,125

Comparaisons appariées[a]

(I) Phase	(J) Phase	Intervalle de confiance à 95 % pour la différence Borne supérieure
OFF	ON	4,125
ON	OFF	6,782

Basées sur les moyennes marginales estimées[a]

a. Variable dépendante : Laser P2 Amplitude.

b. Ajustement pour les comparaisons multiples : Bonferroni.

Tests univariés[a]

Ddl du numérateur	Ddl du dénominateur	F	Sig.
1	24	,253	,620

Le test de F permet de tester l'effet de Phase. Il s'appuie sur les comparaisons appariées (indépendantes) linéaires parmi les moyennes marginales estimées.[a]

a. Variable dépendante : Laser P2 Amplitude.

3.1.5. LEPs P2 Latency.

```
MIXED LaserP2Latency BY Phase
  /CRITERIA=CIN(95) MXITER(100) MXSTEP(10) SCORING(1)
SINGULAR(0.000000000001) HCONVERGE(0,
    ABSOLUTE) LCONVERGE(0, ABSOLUTE) PCONVERGE(0.000001, ABSOLUTE)
  /FIXED=Phase | SSTYPE(3)
  /METHOD=REML
  /PRINT=CPS CORB COVB DESCRIPTIVES G  SOLUTION TESTCOV
  /EMMEANS=TABLES(OVERALL)
  /EMMEANS=TABLES(Phase) COMPARE ADJ(BONFERRONI).
```

Remarques

Sortie obtenue		05-MAY-2021 12:36:37
Commentaires		
Entrée	Jeu de données actif	Jeu_de_données3
	Filtre	<sans>
	Pondération	<sans>
	Fichier scindé	<sans>
	N de lignes dans le fichier de travail	26
Gestion des valeurs manquantes	Définition de la valeur manquante	Les valeurs manquantes définies par l'utilisateur sont traitées comme étant manquantes.
	Observations utilisées	Les statistiques sont basées sur toutes les observations comportant des données valides pour toutes les variables du modèle.

Syntaxe		MIXED LaserP2Latency BY Phase<br>/CRITERIA=CIN(95) MXITER(100) MXSTEP(10) SCORING(1) SINGULAR(0.000000000001) HCONVERGE(0, ABSOLUTE) LCONVERGE(0, ABSOLUTE) PCONVERGE(0.000001, ABSOLUTE)<br>/FIXED=Phase | SSTYPE(3)<br>/METHOD=REML<br>/PRINT=CPS CORB COVB DESCRIPTIVES G SOLUTION TESTCOV<br>/EMMEANS=TABLES(OVERALL)<br>/EMMEANS=TABLES(Phase) COMPARE ADJ(BONFERRONI).
Ressources	Temps de processeur	00:00:00,00
	Temps écoulé	00:00:00,01

Récapitulatif de traitement des observations

		Effectif	Pourcentage marginal
Phase	OFF	13	50,0%
	ON	13	50,0%
Valide		26	100,0%
Exclues		0	
Total		26	

Statistiques descriptives

Laser P2 Latency

Phase	Effectif	Moyenne	Ecart type	Coefficient de variation

OFF	13	,36	,088	24,5%
ON	13	,37	,064	17,3%
Total	26	,37	,076	20,7%

Dimension du modèle[a]

		Nombre de niveaux	Nombre de paramètres
Effets fixes	Constante	1	1
	Phase	2	1
Résidu			1
Total		3	3

a. Variable dépendante : Laser P2 Latency .

Critères d'information[a]

Log de vraisemblance restreint -2	-49,809
Critère d'information d'Akaike (AIC)	-47,809
Critère de Hurvich et Tsai (AICC)	-47,628
Critère de Bozdogan (CAIC)	-45,631
Critère bayésien de Schwartz (BIC)	-46,631

Les critères d'informations sont présentés en plus petit, disposant d'un meilleur format.[a]

a. Variable dépendante : Laser P2 Latency .

Effets fixes

Tests des effets fixes de type III[a]

Source	Ddl du numérateur	Ddl du dénominateur	F	Sig.
Constante	1	24	584,100	,000
Phase	1	24	,123	,729

a. Variable dépendante : Laser P2 Latency .

Estimations des effets fixes[a]

Paramètre	Estimation	Erreur standard	ddl	t	Sig.	Intervalle de confiance à 95 % Borne inférieure
Constante	,370412	,021365	24	17,337	,000	,326316
[Phase=OFF]	-,010577	,030215	24	-,350	,729	-,072938
[Phase=ON]	0[b]	0	.	.	.	.

Estimations des effets fixes[a]

Paramètre	Intervalle de confiance à 95 % Borne supérieure
Constante	,414508
[Phase=OFF]	,051784
[Phase=ON]	.

a. Variable dépendante : Laser P2 Latency .

b. Ce paramètre est défini sur 0, car il est redondant.

Matrice de corrélation pour les estimations des effets fixes[a]

Paramètre	Constante	[Phase=OFF]	[Phase=ON]
Constante	1	-,707	.[b]
[Phase=OFF]	-,707	1	.[b]
[Phase=ON]	.[b]	.[b]	.[b]

a. Variable dépendante : Laser P2 Latency .

b. La corrélation est manquante par défaut, car elle est associée à un paramètre redondant.

Matrice de covariance pour les estimations des effets fixes[a]

Paramètre	Constante	[Phase=OFF]	[Phase=ON]
Constante	,000456	-,000456	0[b]
[Phase=OFF]	-,000456	,000913	0[b]
[Phase=ON]	0[b]	0[b]	0[b]

a. Variable dépendante : Laser P2 Latency .

b. La covariance est définie sur 0, car elle est associée à un paramètre redondant.

Paramètres de covariance

Estimations des paramètres de covariance[a]

Paramètre	Estimation	Erreur standard	Z de Wald	Sig.	Intervalle de confiance à 95 %	
					Borne inférieure	Borne supérieure
Résidu	,005934	,001713	3,464	,001	,003370	,010449

a. Variable dépendante : Laser P2 Latency .

Matrice de corrélation pour les estimations des paramètres de covariance[a]

Paramètre	Résidu
Résidu	1

a. Variable dépendante : Laser P2 Latency .

Matrice de covariance pour les estimations des paramètres de covariance[a]

Paramètre	Résidu
Résidu	2,934592E-6

a. Variable dépendante : Laser P2 Latency .

Moyenne marginale estimée

1. Grand Mean[a]

Moyenne	Erreur standard	ddl	Intervalle de confiance à 95 %	
			Borne inférieure	Borne supérieure
,365	,015	24	,334	,396

a. Variable dépendante : Laser P2 Latency .

2. Phase

Estimations[a]

Phase	Moyenne	Erreur standard	ddl	Intervalle de confiance à 95 %	
				Borne inférieure	Borne supérieure
OFF	,360	,021	24	,316	,404

ON	,370	,021	24	,326	,415

a. Variable dépendante : Laser P2 Latency .

Comparaisons appariées[a]

(I) Phase	(J) Phase	Différence moyenne (I-J)	Erreur standard	ddl	Sig.[b]	Intervalle de confiance à 95 % pour la différence[b] Borne inférieure
OFF	ON	-,011	,030	24	,729	-,073
ON	OFF	,011	,030	24	,729	-,052

Comparaisons appariées[a]

(I) Phase	(J) Phase	Intervalle de confiance à 95 % pour la différence Borne supérieure
OFF	ON	,052
ON	OFF	,073

Basées sur les moyennes marginales estimées[a]

a. Variable dépendante : Laser P2 Latency .

b. Ajustement pour les comparaisons multiples : Bonferroni.

Tests univariés[a]

Ddl du numérateur	Ddl du dénominateur	F	Sig.
1	24	,123	,729

Le test de F permet de tester l'effet de Phase. Il s'appuie sur les comparaisons appariées (indépendantes) linéaires parmi les moyennes marginales estimées.[a]

a. Variable dépendante : Laser P2 Latency .

3.2. Vibrotactile-evoked potentials.

3.2.1. Vibrotactile N2P2 Amplitude.

```
MIXED VibrotactileN2P2Amplitude BY Phase
  /CRITERIA=CIN(95) MXITER(100) MXSTEP(10) SCORING(1)
SINGULAR(0.000000000001) HCONVERGE(0,
    ABSOLUTE) LCONVERGE(0, ABSOLUTE) PCONVERGE(0.000001, ABSOLUTE)
  /FIXED=Phase | SSTYPE(3)
  /METHOD=REML
  /PRINT=CPS CORB COVB DESCRIPTIVES G  SOLUTION TESTCOV
  /EMMEANS=TABLES(OVERALL)
  /EMMEANS=TABLES(Phase) COMPARE ADJ(BONFERRONI).
```

Remarques

Sortie obtenue		05-MAY-2021 12:37:26
Commentaires		
Entrée	Jeu de données actif	Jeu_de_données3
	Filtre	<sans>
	Pondération	<sans>
	Fichier scindé	<sans>
	N de lignes dans le fichier de travail	26
Gestion des valeurs manquantes	Définition de la valeur manquante	Les valeurs manquantes définies par l'utilisateur sont traitées comme étant manquantes.
	Observations utilisées	Les statistiques sont basées sur toutes les observations comportant des données valides pour toutes les variables du modèle.

Syntaxe		MIXED VibrotactileN2P2Amplitude BY Phase<br>/CRITERIA=CIN(95) MXITER(100) MXSTEP(10) SCORING(1) SINGULAR(0.000000000001) HCONVERGE(0, ABSOLUTE) LCONVERGE(0, ABSOLUTE) PCONVERGE(0.000001, ABSOLUTE)<br>/FIXED=Phase | SSTYPE(3)<br>/METHOD=REML<br>/PRINT=CPS CORB COVB DESCRIPTIVES G SOLUTION TESTCOV<br>/EMMEANS=TABLES(OVERALL)<br>/EMMEANS=TABLES(Phase) COMPARE ADJ(BONFERRONI).
Ressources	Temps de processeur	00:00:00,00
	Temps écoulé	00:00:00,01

Récapitulatif de traitement des observations

		Effectif	Pourcentage marginal
Phase	OFF	13	50,0%
	ON	13	50,0%
Valide		26	100,0%
Exclues		0	
Total		26	

Statistiques descriptives

Vibrotactile N2P2 Amplitude

Phase	Effectif	Moyenne	Ecart type	Coefficient de variation
OFF	13	15,731990769230768	5,539259908890449	35,2%
ON	13	19,684574615384612	7,986928449360883	40,6%
Total	26	17,708282692307690	7,029202203997580	39,7%

Dimension du modèle[a]

		Nombre de niveaux	Nombre de paramètres
Effets fixes	Constante	1	1
	Phase	2	1
Résidu			1
Total		3	3

a. Variable dépendante : Vibrotactile N2P2 Amplitude.

Critères d'information[a]

Log de vraisemblance restreint -2	165,763
Critère d'information d'Akaike (AIC)	167,763
Critère de Hurvich et Tsai (AICC)	167,945
Critère de Bozdogan (CAIC)	169,941
Critère bayésien de Schwartz (BIC)	168,941

Les critères d'informations sont présentés en plus petit, disposant d'un meilleur format.[a]

a. Variable dépendante : Vibrotactile N2P2 Amplitude.

Effets fixes

Tests des effets fixes de type III[a]

Source	Ddl du numérateur	Ddl du dénominateur	F	Sig.
Constante	1	24	172,600	,000
Phase	1	24	2,150	,156

a. Variable dépendante : Vibrotactile N2P2 Amplitude.

Estimations des effets fixes[a]

Paramètre	Estimation	Erreur standard	ddl	t	Sig.	Intervalle de confiance à 95 % Borne inférieure
Constante	19,684575	1,906209	24	10,327	,000	15,750353
[Phase=OFF]	-3,952584	2,695786	24	-1,466	,156	-9,516413
[Phase=ON]	0[b]	0	.	.	.	.

Estimations des effets fixes[a]

Paramètre	Intervalle de confiance à 95 % Borne supérieure
Constante	23,618796
[Phase=OFF]	1,611246
[Phase=ON]	.

a. Variable dépendante : Vibrotactile N2P2 Amplitude.

b. Ce paramètre est défini sur 0, car il est redondant.

Matrice de corrélation pour les estimations des effets fixes[a]

Paramètre	Constante	[Phase=OFF]	[Phase=ON]
Constante	1	-,707	.[b]
[Phase=OFF]	-,707	1	.[b]
[Phase=ON]	.[b]	.[b]	.[b]

a. Variable dépendante : Vibrotactile N2P2 Amplitude.

b. La corrélation est manquante par défaut, car elle est associée à un paramètre redondant.

Matrice de covariance pour les estimations des effets fixes[a]

Paramètre	Constante	[Phase=OFF]	[Phase=ON]
Constante	3,633632	-3,633632	0[b]
[Phase=OFF]	-3,633632	7,267264	0[b]

[Phase=ON]	0[b]	0[b]	0[b]

a. Variable dépendante : Vibrotactile N2P2 Amplitude.

b. La covariance est définie sur 0, car elle est associée à un paramètre redondant.

Paramètres de covariance

Estimations des paramètres de covariance[a]

Paramètre	Estimation	Erreur standard	Z de Wald	Sig.	Intervalle de confiance à 95 %	
					Borne inférieure	Borne supérieure
Résidu	47,237213	13,636209	3,464	,001	26,826471	83,177332

a. Variable dépendante : Vibrotactile N2P2 Amplitude.

Matrice de corrélation pour les estimations des paramètres de covariance[a]

Paramètre	Résidu
Résidu	1

a. Variable dépendante : Vibrotactile N2P2 Amplitude.

Matrice de covariance pour les estimations des paramètres de covariance[a]

Paramètre	Résidu
Résidu	185,946193

a. Variable dépendante : Vibrotactile N2P2 Amplitude.

Moyenne marginale estimée

1. Grand Mean[a]

Moyenne	Erreur standard	ddl	Intervalle de confiance à 95 %	
			Borne inférieure	Borne supérieure
17,708	1,348	24	14,926	20,490

a. Variable dépendante : Vibrotactile N2P2 Amplitude.

2. Phase

Estimations[a]

Phase	Moyenne	Erreur standard	ddl	Intervalle de confiance à 95 % Borne inférieure	Borne supérieure
OFF	15,732	1,906	24	11,798	19,666
ON	19,685	1,906	24	15,750	23,619

a. Variable dépendante : Vibrotactile N2P2 Amplitude.

Comparaisons appariées[a]

(I) Phase	(J) Phase	Différence moyenne (I-J)	Erreur standard	ddl	Sig.[b]	Intervalle de confiance à 95 % pour la différence[b] Borne inférieure
OFF	ON	-3,953	2,696	24	,156	-9,516
ON	OFF	3,953	2,696	24	,156	-1,611

Comparaisons appariées[a]

(I) Phase	(J) Phase	Intervalle de confiance à 95 % pour la différence Borne supérieure
OFF	ON	1,611
ON	OFF	9,516

Basées sur les moyennes marginales estimées[a]

a. Variable dépendante : Vibrotactile N2P2 Amplitude.

b. Ajustement pour les comparaisons multiples : Bonferroni.

Tests univariés[a]

Ddl du numérateur	Ddl du dénominateur	F	Sig.
1	24	2,150	,156

Le test de F permet de tester l'effet de Phase. Il s'appuie sur les comparaisons appariées (indépendantes) linéaires parmi les moyennes marginales estimées.[a]

a. Variable dépendante : Vibrotactile N2P2 Amplitude.

3.2.2. Vibrotactile N2 Amplitude.

```
MIXED VibrotactileN2Amplitude BY Phase
  /CRITERIA=CIN(95) MXITER(100) MXSTEP(10) SCORING(1)
SINGULAR(0.000000000001) HCONVERGE(0,
    ABSOLUTE) LCONVERGE(0, ABSOLUTE) PCONVERGE(0.000001, ABSOLUTE)
  /FIXED=Phase | SSTYPE(3)
  /METHOD=REML
  /PRINT=CPS CORB COVB DESCRIPTIVES G  SOLUTION TESTCOV
  /EMMEANS=TABLES(OVERALL)
  /EMMEANS=TABLES(Phase) COMPARE ADJ(BONFERRONI).
```

Remarques

Sortie obtenue		05-MAY-2021 12:38:11
Commentaires		
Entrée	Jeu de données actif	Jeu_de_données3
	Filtre	<sans>
	Pondération	<sans>
	Fichier scindé	<sans>
	N de lignes dans le fichier de travail	26
Gestion des valeurs manquantes	Définition de la valeur manquante	Les valeurs manquantes définies par l'utilisateur sont traitées comme étant manquantes.
	Observations utilisées	Les statistiques sont basées sur toutes les observations comportant des données valides pour toutes les variables du modèle.

Syntaxe		MIXED VibrotactileN2Amplitude BY Phase /CRITERIA=CIN(95) MXITER(100) MXSTEP(10) SCORING(1) SINGULAR(0.000000000001) HCONVERGE(0, ABSOLUTE) LCONVERGE(0, ABSOLUTE) PCONVERGE(0.000001, ABSOLUTE) /FIXED=Phase | SSTYPE(3) /METHOD=REML /PRINT=CPS CORB COVB DESCRIPTIVES G SOLUTION TESTCOV /EMMEANS=TABLES(OVERALL) /EMMEANS=TABLES(Phase) COMPARE ADJ(BONFERRONI).
Ressources	Temps de processeur	00:00:00,02
	Temps écoulé	00:00:00,01

Récapitulatif de traitement des observations

		Effectif	Pourcentage marginal
Phase	OFF	13	50,0%
	ON	13	50,0%
Valide		26	100,0%
Exclues		0	
Total		26	

Statistiques descriptives

Vibrotactile N2 Amplitude

Phase	Effectif	Moyenne	Ecart type	Coefficient de variation
OFF	13	-7,28	5,248	-72,1%
ON	13	-8,09	5,809	-71,8%
Total	26	-7,68	5,439	-70,8%

Dimension du modèle[a]

		Nombre de niveaux	Nombre de paramètres
Effets fixes	Constante	1	1
	Phase	2	1
Résidu			1
Total		3	3

a. Variable dépendante : Vibrotactile N2 Amplitude.

Critères d'information[a]

Log de vraisemblance restreint -2	155,377
Critère d'information d'Akaike (AIC)	157,377
Critère de Hurvich et Tsai (AICC)	157,558
Critère de Bozdogan (CAIC)	159,555
Critère bayésien de Schwartz (BIC)	158,555

Les critères d'informations sont présentés en plus petit, disposant d'un meilleur format.[a]

a. Variable dépendante : Vibrotactile N2 Amplitude.

Effets fixes

Tests des effets fixes de type III[a]

Source	Ddl du numérateur	Ddl du dénominateur	F	Sig.
Constante	1	24	50,090	,000
Phase	1	24	,139	,713

a. Variable dépendante : Vibrotactile N2 Amplitude.

Estimations des effets fixes[a]

Paramètre	Estimation	Erreur standard	ddl	t	Sig.	Intervalle de confiance à 95 % Borne inférieure
Constante	-8,088128	1,535302	24	-5,268	,000	-11,256836
[Phase=OFF]	,809488	2,171245	24	,373	,713	-3,671741
[Phase=ON]	0[b]	0	.	.	.	.

Estimations des effets fixes[a]

Paramètre	Intervalle de confiance à 95 % Borne supérieure
Constante	-4,919421
[Phase=OFF]	5,290718
[Phase=ON]	.

a. Variable dépendante : Vibrotactile N2 Amplitude.

b. Ce paramètre est défini sur 0, car il est redondant.

Matrice de corrélation pour les estimations des effets fixes[a]

Paramètre	Constante	[Phase=OFF]	[Phase=ON]
Constante	1	-,707	.[b]
[Phase=OFF]	-,707	1	.[b]
[Phase=ON]	.[b]	.[b]	.[b]

a. Variable dépendante : Vibrotactile N2 Amplitude.

b. La corrélation est manquante par défaut, car elle est associée à un paramètre redondant.

Matrice de covariance pour les estimations des effets fixes[a]

Paramètre	Constante	[Phase=OFF]	[Phase=ON]
Constante	2,357152	-2,357152	0[b]
[Phase=OFF]	-2,357152	4,714305	0[b]
[Phase=ON]	0[b]	0[b]	0[b]

a. Variable dépendante : Vibrotactile N2 Amplitude.

b. La covariance est définie sur 0, car elle est associée à un paramètre redondant.

Paramètres de covariance

Estimations des paramètres de covariance[a]

Paramètre	Estimation	Erreur standard	Z de Wald	Sig.	Intervalle de confiance à 95 % Borne inférieure	Borne supérieure
Résidu	30,642979	8,845866	3,464	,001	17,402445	53,957486

a. Variable dépendante : Vibrotactile N2 Amplitude.

Matrice de corrélation pour les estimations des paramètres de covariance[a]

Paramètre	Résidu
Résidu	1

a. Variable dépendante : Vibrotactile N2 Amplitude.

Matrice de covariance pour les estimations des paramètres de covariance[a]

Paramètre	Résidu
Résidu	78,249349

a. Variable dépendante : Vibrotactile N2 Amplitude.

Moyenne marginale estimée

1. Grand Mean[a]

Moyenne	Erreur standard	ddl	Intervalle de confiance à 95 % Borne inférieure	Borne supérieure
-7,683	1,086	24	-9,924	-5,443

a. Variable dépendante : Vibrotactile N2 Amplitude.

2. Phase

Estimations[a]

Phase	Moyenne	Erreur standard	ddl	Intervalle de confiance à 95 % Borne inférieure	Borne supérieure
OFF	-7,279	1,535	24	-10,447	-4,110
ON	-8,088	1,535	24	-11,257	-4,919

a. Variable dépendante : Vibrotactile N2 Amplitude.

Comparaisons appariées[a]

(I) Phase	(J) Phase	Différence moyenne (I-J)	Erreur standard	ddl	Sig.[b]	Intervalle de confiance à 95 % pour la différence[b] Borne inférieure
OFF	ON	,809	2,171	24	,713	-3,672
ON	OFF	-,809	2,171	24	,713	-5,291

Comparaisons appariées[a]

(I) Phase	(J) Phase	Intervalle de confiance à 95 % pour la différence Borne supérieure
OFF	ON	5,291
ON	OFF	3,672

Basées sur les moyennes marginales estimées[a]

a. Variable dépendante : Vibrotactile N2 Amplitude.

b. Ajustement pour les comparaisons multiples : Bonferroni.

Tests univariés[a]

Ddl du numérateur	Ddl du dénominateur	F	Sig.
1	24	,139	,713

Le test de F permet de tester l'effet de Phase. Il s'appuie sur les comparaisons appariées (indépendantes) linéaires parmi les moyennes marginales estimées.[a]

a. Variable dépendante : Vibrotactile N2 Amplitude.

3.2.3. Vibrotactile N2 Latency.

```
MIXED VibrotactileN2Latency BY Phase
  /CRITERIA=CIN(95) MXITER(100) MXSTEP(10) SCORING(1)
SINGULAR(0.000000000001) HCONVERGE(0,
    ABSOLUTE) LCONVERGE(0, ABSOLUTE) PCONVERGE(0.000001, ABSOLUTE)
  /FIXED=Phase | SSTYPE(3)
  /METHOD=REML
  /PRINT=CPS CORB COVB DESCRIPTIVES G  SOLUTION TESTCOV
  /EMMEANS=TABLES(OVERALL)
  /EMMEANS=TABLES(Phase) COMPARE ADJ(BONFERRONI).
```

Remarques

Sortie obtenue		05-MAY-2021 12:38:57
Commentaires		
Entrée	Jeu de données actif	Jeu_de_données3
	Filtre	<sans>
	Pondération	<sans>
	Fichier scindé	<sans>
	N de lignes dans le fichier de travail	26
Gestion des valeurs manquantes	Définition de la valeur manquante	Les valeurs manquantes définies par l'utilisateur sont traitées comme étant manquantes.
	Observations utilisées	Les statistiques sont basées sur toutes les observations comportant des données valides pour toutes les variables du modèle.

Syntaxe		MIXED VibrotactileN2Latency BY Phase<br>/CRITERIA=CIN(95) MXITER(100) MXSTEP(10) SCORING(1) SINGULAR(0.000000000001) HCONVERGE(0, ABSOLUTE) LCONVERGE(0, ABSOLUTE) PCONVERGE(0.000001, ABSOLUTE)<br>/FIXED=Phase | SSTYPE(3)<br>/METHOD=REML<br>/PRINT=CPS CORB COVB DESCRIPTIVES G SOLUTION TESTCOV<br>/EMMEANS=TABLES(OVERALL)<br>/EMMEANS=TABLES(Phase) COMPARE ADJ(BONFERRONI).
Ressources	Temps de processeur	00:00:00,02
	Temps écoulé	00:00:00,01

Récapitulatif de traitement des observations

		Effectif	Pourcentage marginal
Phase	OFF	13	50,0%
	ON	13	50,0%
Valide		26	100,0%
Exclues		0	
Total		26	

Statistiques descriptives

Vibrotactile N2 Latency

Phase	Effectif	Moyenne	Ecart type	Coefficient de variation
OFF	13	,18	,068	37,8%
ON	13	,18	,060	33,2%
Total	26	,18	,063	34,9%

Dimension du modèle[a]

		Nombre de niveaux	Nombre de paramètres
Effets fixes	Constante	1	1
	Phase	2	1
Résidu			1
Total		3	3

a. Variable dépendante : Vibrotactile N2 Latency .

Critères d'information[a]

Log de vraisemblance restreint -2	-58,434
Critère d'information d'Akaike (AIC)	-56,434
Critère de Hurvich et Tsai (AICC)	-56,252
Critère de Bozdogan (CAIC)	-54,256
Critère bayésien de Schwartz (BIC)	-55,256

Les critères d'informations sont présentés en plus petit, disposant d'un meilleur format.[a]

a. Variable dépendante : Vibrotactile N2 Latency .

Effets fixes

Tests des effets fixes de type III[a]

Source	Ddl du numérateur	Ddl du dénominateur	F	Sig.
Constante	1	24	205,506	,000
Phase	1	24	,000	,989

a. Variable dépendante : Vibrotactile N2 Latency .

Estimations des effets fixes[a]

Paramètre	Estimation	Erreur standard	ddl	t	Sig.	Intervalle de confiance à 95 % Borne inférieure
Constante	,181140	,017852	24	10,147	,000	,144296
[Phase=OFF]	-,000365	,025246	24	-,014	,989	-,052471
[Phase=ON]	0[b]	0	.	.	.	.

Estimations des effets fixes[a]

Paramètre	Intervalle de confiance à 95 % Borne supérieure
Constante	,217984
[Phase=OFF]	,051740
[Phase=ON]	.

a. Variable dépendante : Vibrotactile N2 Latency .

b. Ce paramètre est défini sur 0, car il est redondant.

Matrice de corrélation pour les estimations des effets fixes[a]

Paramètre	Constante	[Phase=OFF]	[Phase=ON]
Constante	1	-,707	.[b]
[Phase=OFF]	-,707	1	.[b]
[Phase=ON]	.[b]	.[b]	.[b]

a. Variable dépendante : Vibrotactile N2 Latency .

b. La corrélation est manquante par défaut, car elle est associée à un paramètre redondant.

Matrice de covariance pour les estimations des effets fixes[a]

Paramètre	Constante	[Phase=OFF]	[Phase=ON]
Constante	,000319	-,000319	0[b]
[Phase=OFF]	-,000319	,000637	0[b]
[Phase=ON]	0[b]	0[b]	0[b]

a. Variable dépendante : Vibrotactile N2 Latency .

b. La covariance est définie sur 0, car elle est associée à un paramètre redondant.

Paramètres de covariance

Estimations des paramètres de covariance[a]

Paramètre	Estimation	Erreur standard	Z de Wald	Sig.	Intervalle de confiance à 95 % Borne inférieure	Borne supérieure
Résidu	,004143	,001196	3,464	,001	,002353	,007295

a. Variable dépendante : Vibrotactile N2 Latency .

Matrice de corrélation pour les estimations des paramètres de covariance[a]

Paramètre	Résidu
Résidu	1

a. Variable dépendante : Vibrotactile N2 Latency .

Matrice de covariance pour les estimations des paramètres de covariance[a]

Paramètre	Résidu
Résidu	1,430280E-6

a. Variable dépendante : Vibrotactile N2 Latency .

Moyenne marginale estimée

1. Grand Mean[a]

Moyenne	Erreur standard	ddl	Intervalle de confiance à 95 % Borne inférieure	Borne supérieure
,181	,013	24	,155	,207

a. Variable dépendante : Vibrotactile N2 Latency .

2. Phase

Estimations[a]

Phase	Moyenne	Erreur standard	ddl	Intervalle de confiance à 95 % Borne inférieure	Borne supérieure
OFF	,181	,018	24	,144	,218
ON	,181	,018	24	,144	,218

a. Variable dépendante : Vibrotactile N2 Latency .

Comparaisons appariées[a]

(I) Phase	(J) Phase	Différence moyenne (I-J)	Erreur standard	ddl	Sig.[b]	Intervalle de confiance à 95 % pour la différence[b] Borne inférieure
OFF	ON	,000	,025	24	,989	-,052
ON	OFF	,000	,025	24	,989	-,052

Comparaisons appariées[a]

(I) Phase	(J) Phase	Intervalle de confiance à 95 % pour la différence Borne supérieure
OFF	ON	,052
ON	OFF	,052

Basées sur les moyennes marginales estimées[a]

a. Variable dépendante : Vibrotactile N2 Latency .

b. Ajustement pour les comparaisons multiples : Bonferroni.

Tests univariés[a]

Ddl du numérateur	Ddl du dénominateur	F	Sig.
1	24	,000	,989

Le test de F permet de tester l'effet de Phase. Il s'appuie sur les comparaisons appariées (indépendantes) linéaires parmi les moyennes marginales estimées.[a]

a. Variable dépendante : Vibrotactile N2 Latency .

3.2.4. Vibrotactile P2 Amplitude.

```
MIXED VibrotactileP2Amplitude BY Phase
  /CRITERIA=CIN(95) MXITER(100) MXSTEP(10) SCORING(1)
SINGULAR(0.000000000001) HCONVERGE(0,
    ABSOLUTE) LCONVERGE(0, ABSOLUTE) PCONVERGE(0.000001, ABSOLUTE)
  /FIXED=Phase | SSTYPE(3)
  /METHOD=REML
  /PRINT=CPS CORB COVB DESCRIPTIVES G  SOLUTION TESTCOV
  /EMMEANS=TABLES(OVERALL)
  /EMMEANS=TABLES(Phase) COMPARE ADJ(BONFERRONI).
```

Remarques

Sortie obtenue		05-MAY-2021 12:39:38
Commentaires		
Entrée	Jeu de données actif	Jeu_de_données3
	Filtre	<sans>
	Pondération	<sans>
	Fichier scindé	<sans>
	N de lignes dans le fichier de travail	26
Gestion des valeurs manquantes	Définition de la valeur manquante	Les valeurs manquantes définies par l'utilisateur sont traitées comme étant manquantes.
	Observations utilisées	Les statistiques sont basées sur toutes les observations comportant des données valides pour toutes les variables du modèle.

Syntaxe		MIXED VibrotactileP2Amplitude BY Phase<br>/CRITERIA=CIN(95) MXITER(100) MXSTEP(10) SCORING(1) SINGULAR(0.000000000001) HCONVERGE(0, ABSOLUTE) LCONVERGE(0, ABSOLUTE) PCONVERGE(0.000001, ABSOLUTE)<br>/FIXED=Phase | SSTYPE(3)<br>/METHOD=REML<br>/PRINT=CPS CORB COVB DESCRIPTIVES G SOLUTION TESTCOV<br>/EMMEANS=TABLES(OVERALL)<br>/EMMEANS=TABLES(Phase) COMPARE ADJ(BONFERRONI).
Ressources	Temps de processeur	00:00:00,02
	Temps écoulé	00:00:00,02

Récapitulatif de traitement des observations

		Effectif	Pourcentage marginal
Phase	OFF	13	50,0%
	ON	13	50,0%
Valide		26	100,0%
Exclues		0	
Total		26	

Statistiques descriptives

Vibrotactile P2 Amplitude

Phase	Effectif	Moyenne	Ecart type	Coefficient de variation
OFF	13	8,45	5,707	67,5%
ON	13	11,60	8,362	72,1%
Total	26	10,02	7,195	71,8%

Dimension du modèle[a]

		Nombre de niveaux	Nombre de paramètres
Effets fixes	Constante	1	1
	Phase	2	1
Résidu			1
Total		3	3

a. Variable dépendante : Vibrotactile P2 Amplitude.

Critères d'information[a]

Log de vraisemblance restreint -2	167,720
Critère d'information d'Akaike (AIC)	169,720
Critère de Hurvich et Tsai (AICC)	169,901
Critère de Bozdogan (CAIC)	171,898
Critère bayésien de Schwartz (BIC)	170,898

Les critères d'informations sont présentés en plus petit, disposant d'un meilleur format.[a]

a. Variable dépendante : Vibrotactile P2 Amplitude.

Effets fixes

Tests des effets fixes de type III[a]

Source	Ddl du numérateur	Ddl du dénominateur	F	Sig.
Constante	1	24	50,986	,000
Phase	1	24	1,253	,274

a. Variable dépendante : Vibrotactile P2 Amplitude.

Estimations des effets fixes[a]

Paramètre	Estimation	Erreur standard	ddl	t	Sig.	Intervalle de confiance à 95 % Borne inférieure
Constante	11,596446	1,985500	24	5,841	,000	7,498576
[Phase=OFF]	-3,143095	2,807921	24	-1,119	,274	-8,938359
[Phase=ON]	0[b]	0	.	.	.	.

Estimations des effets fixes[a]

Paramètre	Intervalle de confiance à 95 % Borne supérieure
Constante	15,694317
[Phase=OFF]	2,652169
[Phase=ON]	.

a. Variable dépendante : Vibrotactile P2 Amplitude.

b. Ce paramètre est défini sur 0, car il est redondant.

Matrice de corrélation pour les estimations des effets fixes[a]

Paramètre	Constante	[Phase=OFF]	[Phase=ON]
Constante	1	-,707	.[b]
[Phase=OFF]	-,707	1	.[b]
[Phase=ON]	.[b]	.[b]	.[b]

a. Variable dépendante : Vibrotactile P2 Amplitude.

b. La corrélation est manquante par défaut, car elle est associée à un paramètre redondant.

Matrice de covariance pour les estimations des effets fixes[a]

Paramètre	Constante	[Phase=OFF]	[Phase=ON]
Constante	3,942210	-3,942210	0[b]
[Phase=OFF]	-3,942210	7,884420	0[b]
[Phase=ON]	0[b]	0[b]	0[b]

a. Variable dépendante : Vibrotactile P2 Amplitude.

b. La covariance est définie sur 0, car elle est associée à un paramètre redondant.

Paramètres de covariance

Estimations des paramètres de covariance[a]

Paramètre	Estimation	Erreur standard	Z de Wald	Sig.	Intervalle de confiance à 95 % Borne inférieure	Borne supérieure
Résidu	51,248732	14,794235	3,464	,001	29,104652	90,240989

a. Variable dépendante : Vibrotactile P2 Amplitude.

Matrice de corrélation pour les estimations des paramètres de covariance[a]

Paramètre	Résidu
Résidu	1

a. Variable dépendante : Vibrotactile P2 Amplitude.

Matrice de covariance pour les estimations des paramètres de covariance[a]

Paramètre	Résidu
Résidu	218,869377

a. Variable dépendante : Vibrotactile P2 Amplitude.

Moyenne marginale estimée

1. Grand Mean[a]

Moyenne	Erreur standard	ddl	Intervalle de confiance à 95 % Borne inférieure	Borne supérieure
10,025	1,404	24	7,127	12,923

a. Variable dépendante : Vibrotactile P2 Amplitude.

2. Phase

Estimations[a]

Phase	Moyenne	Erreur standard	ddl	Intervalle de confiance à 95 % Borne inférieure	Borne supérieure
OFF	8,453	1,985	24	4,355	12,551
ON	11,596	1,985	24	7,499	15,694

a. Variable dépendante : Vibrotactile P2 Amplitude.

Comparaisons appariées[a]

(I) Phase	(J) Phase	Différence moyenne (I-J)	Erreur standard	ddl	Sig.[b]	Intervalle de confiance à 95 % pour la différence[b] Borne inférieure
OFF	ON	-3,143	2,808	24	,274	-8,938
ON	OFF	3,143	2,808	24	,274	-2,652

Comparaisons appariées[a]

(I) Phase	(J) Phase	Intervalle de confiance à 95 % pour la différence Borne supérieure
OFF	ON	2,652
ON	OFF	8,938

Basées sur les moyennes marginales estimées[a]

a. Variable dépendante : Vibrotactile P2 Amplitude.

b. Ajustement pour les comparaisons multiples : Bonferroni.

Tests univariés[a]

Ddl du numérateur	Ddl du dénominateur	F	Sig.
1	24	1,253	,274

Le test de F permet de tester l'effet de Phase. Il s'appuie sur les comparaisons appariées (indépendantes) linéaires parmi les moyennes marginales estimées.[a]

a. Variable dépendante : Vibrotactile P2 Amplitude.

2.3.5. Vibrotactile P2 Latency.

```
MIXED VibrotactileP2Latency BY Phase
  /CRITERIA=CIN(95) MXITER(100) MXSTEP(10) SCORING(1)
SINGULAR(0.000000000001) HCONVERGE(0,
    ABSOLUTE) LCONVERGE(0, ABSOLUTE) PCONVERGE(0.000001, ABSOLUTE)
  /FIXED=Phase | SSTYPE(3)
  /METHOD=REML
  /PRINT=CPS CORB COVB DESCRIPTIVES G  SOLUTION TESTCOV
  /EMMEANS=TABLES(OVERALL)
  /EMMEANS=TABLES(Phase) COMPARE ADJ(BONFERRONI).
```

Remarques

Sortie obtenue		05-MAY-2021 12:40:08
Commentaires		
Entrée	Jeu de données actif	Jeu_de_données3
	Filtre	<sans>
	Pondération	<sans>
	Fichier scindé	<sans>
	N de lignes dans le fichier de travail	26
Gestion des valeurs manquantes	Définition de la valeur manquante	Les valeurs manquantes définies par l'utilisateur sont traitées comme étant manquantes.
	Observations utilisées	Les statistiques sont basées sur toutes les observations comportant des données valides pour toutes les variables du modèle.

Syntaxe		MIXED VibrotactileP2Latency BY Phase<br>/CRITERIA=CIN(95) MXITER(100) MXSTEP(10) SCORING(1) SINGULAR(0.000000000001) HCONVERGE(0, ABSOLUTE) LCONVERGE(0, ABSOLUTE) PCONVERGE(0.000001, ABSOLUTE)<br>/FIXED=Phase | SSTYPE(3)<br>/METHOD=REML<br>/PRINT=CPS CORB COVB DESCRIPTIVES G SOLUTION TESTCOV<br>/EMMEANS=TABLES(OVERALL)<br>/EMMEANS=TABLES(Phase) COMPARE ADJ(BONFERRONI).
Ressources	Temps de processeur	00:00:00,00
	Temps écoulé	00:00:00,01

Récapitulatif de traitement des observations

		Effectif	Pourcentage marginal
Phase	OFF	13	50,0%
	ON	13	50,0%
Valide		26	100,0%
Exclues		0	
Total		26	

Statistiques descriptives

Vibrotactile P2 Latency

Phase	Effectif	Moyenne	Ecart type	Coefficient de variation

OFF	13	,30	,085	28,7%
ON	13	,33	,086	26,2%
Total	26	,31	,085	27,4%

Dimension du modèle[a]

		Nombre de niveaux	Nombre de paramètres
Effets fixes	Constante	1	1
	Phase	2	1
Résidu			1
Total		3	3

a. Variable dépendante : Vibrotactile P2 Latency .

Critères d'information[a]

Log de vraisemblance restreint -2	-44,748
Critère d'information d'Akaike (AIC)	-42,748
Critère de Hurvich et Tsai (AICC)	-42,566
Critère de Bozdogan (CAIC)	-40,570
Critère bayésien de Schwartz (BIC)	-41,570

Les critères d'informations sont présentés en plus petit, disposant d'un meilleur format.[a]

a. Variable dépendante : Vibrotactile P2 Latency .

Effets fixes

Tests des effets fixes de type III[a]

Source	Ddl du numérateur	Ddl du dénominateur	F	Sig.
Constante	1	24	345,692	,000
Phase	1	24	,890	,355

a. Variable dépendante : Vibrotactile P2 Latency .

Estimations des effets fixes[a]

Paramètre	Estimation	Erreur standard	ddl	t	Sig.	Intervalle de confiance à 95 % Borne inférieure
Constante	,327968	,023741	24	13,814	,000	,278968
[Phase=OFF]	-,031673	,033576	24	-,943	,355	-,100970
[Phase=ON]	0[b]	0	.	.	.	.

Estimations des effets fixes[a]

Paramètre	Intervalle de confiance à 95 % Borne supérieure
Constante	,376968
[Phase=OFF]	,037623
[Phase=ON]	.

a. Variable dépendante : Vibrotactile P2 Latency .

b. Ce paramètre est défini sur 0, car il est redondant.

Matrice de corrélation pour les estimations des effets fixes[a]

Paramètre	Constante	[Phase=OFF]	[Phase=ON]
Constante	1	-,707	.[b]
[Phase=OFF]	-,707	1	.[b]
[Phase=ON]	.[b]	.[b]	.[b]

a. Variable dépendante : Vibrotactile P2 Latency .

b. La corrélation est manquante par défaut, car elle est associée à un paramètre redondant.

Matrice de covariance pour les estimations des effets fixes[a]

Paramètre	Constante	[Phase=OFF]	[Phase=ON]
Constante	,000564	-,000564	0[b]
[Phase=OFF]	-,000564	,001127	0[b]
[Phase=ON]	0[b]	0[b]	0[b]

a. Variable dépendante : Vibrotactile P2 Latency .

b. La covariance est définie sur 0, car elle est associée à un paramètre redondant.

Paramètres de covariance

Estimations des paramètres de covariance[a]

Paramètre	Estimation	Erreur standard	Z de Wald	Sig.	Intervalle de confiance à 95 %	
					Borne inférieure	Borne supérieure
Résidu	,007328	,002115	3,464	,001	,004161	,012903

a. Variable dépendante : Vibrotactile P2 Latency .

Matrice de corrélation pour les estimations des paramètres de covariance[a]

Paramètre	Résidu
Résidu	1

a. Variable dépendante : Vibrotactile P2 Latency .

Matrice de covariance pour les estimations des paramètres de covariance[a]

Paramètre	Résidu
Résidu	4,474415E-6

a. Variable dépendante : Vibrotactile P2 Latency .

Moyenne marginale estimée

1. Grand Mean[a]

Moyenne	Erreur standard	ddl	Intervalle de confiance à 95 %	
			Borne inférieure	Borne supérieure
,312	,017	24	,277	,347

a. Variable dépendante : Vibrotactile P2 Latency .

2. Phase

Estimations[a]

Phase	Moyenne	Erreur standard	ddl	Intervalle de confiance à 95 %	
				Borne inférieure	Borne supérieure
OFF	,296	,024	24	,247	,345

ON	,328	,024	24	,279	,377

a. Variable dépendante : Vibrotactile P2 Latency .

Comparaisons appariées[a]

(I) Phase	(J) Phase	Différence moyenne (I-J)	Erreur standard	ddl	Sig.[b]	Intervalle de confiance à 95 % pour la différence[b] Borne inférieure
OFF	ON	-,032	,034	24	,355	-,101
ON	OFF	,032	,034	24	,355	-,038

Comparaisons appariées[a]

(I) Phase	(J) Phase	Intervalle de confiance à 95 % pour la différence Borne supérieure
OFF	ON	,038
ON	OFF	,101

Basées sur les moyennes marginales estimées[a]

a. Variable dépendante : Vibrotactile P2 Latency .

b. Ajustement pour les comparaisons multiples : Bonferroni.

Tests univariés[a]

Ddl du numérateur	Ddl du dénominateur	F	Sig.
1	24	,890	,355

Le test de F permet de tester l'effet de Phase. Il s'appuie sur les comparaisons appariées (indépendantes) linéaires parmi les moyennes marginales estimées.[a]

a. Variable dépendante : Vibrotactile P2 Latency .
