# Supplementary material for: Transcutaneous auricular VNS applied to experimental pain: A paired behavioral and EEG study using thermonociceptive CO2 laser
Source: PLoS One. 2021 Jul 12;16(7):e0254480. doi: 10.1371/journal.pone.0254480 (PMC8274876; doi:10.1371/journal.pone.0254480)

# Transcutaneous VNS applied to experimental pain: a paired behavioral and EEG study using thermonociceptive CO2 laser

# **Supplementary Appendix**

# Bayesian Repeated Measures ANOVA (conducted using Jasp 0.14.00)

### Experiment 1

**Behavioral responses**

#### **Detection thresholds**

#### **Heat sensitive C- fibers**

| **Model Comparison** | | | | | | | | | | | |
| --- | --- | --- | --- | --- | --- | --- | --- | --- | --- | --- | --- |
| **Models** | | **P(M)** | | **P(M\|data)** | | **BF _M_** | | **BF _01_** | | **error %** | |
| Null model (incl. subject) |  | 0.053 |  | 0.473 |  | 16.177 |  | 1.000 |  |  |  |
| Time |  | 0.053 |  | 0.158 |  | 3.373 |  | 2.999 |  | 1.205 |  |
| Condition |  | 0.053 |  | 0.148 |  | 3.133 |  | 3.193 |  | 1.798 |  |
| Condition + Time |  | 0.053 |  | 0.049 |  | 0.931 |  | 9.626 |  | 1.669 |  |
| Volunteers |  | 0.053 |  | 0.038 |  | 0.714 |  | 12.412 |  | 0.223 |  |
| Condition + Time + Volunteers + Condition  ✻  Time + Condition  ✻  Volunteers + Time  ✻  Volunteers + Condition  ✻  Time  ✻  Volunteers |  | 0.053 |  | 0.037 |  | 0.685 |  | 12.912 |  | 13.069 |  |
| Condition + Time + Condition  ✻  Time |  | 0.053 |  | 0.025 |  | 0.459 |  | 19.016 |  | 2.082 |  |
| Condition + Volunteers + Condition  ✻  Volunteers |  | 0.053 |  | 0.014 |  | 0.264 |  | 32.779 |  | 1.056 |  |
| Time + Volunteers |  | 0.053 |  | 0.013 |  | 0.233 |  | 36.967 |  | 1.207 |  |
| Condition + Volunteers |  | 0.053 |  | 0.012 |  | 0.222 |  | 38.904 |  | 1.168 |  |
| Time + Volunteers + Time  ✻  Volunteers |  | 0.053 |  | 0.006 |  | 0.114 |  | 75.083 |  | 1.026 |  |
| Condition + Time + Volunteers + Condition  ✻  Volunteers + Time  ✻  Volunteers |  | 0.053 |  | 0.005 |  | 0.096 |  | 89.512 |  | 2.362 |  |
| Condition + Time + Volunteers + Condition  ✻  Volunteers |  | 0.053 |  | 0.005 |  | 0.095 |  | 89.985 |  | 2.313 |  |
| Condition + Time + Volunteers |  | 0.053 |  | 0.004 |  | 0.073 |  | 116.954 |  | 1.348 |  |
| Condition + Time + Volunteers + Condition  ✻  Time + Condition  ✻  Volunteers + Time  ✻  Volunteers |  | 0.053 |  | 0.003 |  | 0.058 |  | 148.586 |  | 1.762 |  |
| Condition + Time + Volunteers + Condition  ✻  Time + Condition  ✻  Volunteers |  | 0.053 |  | 0.003 |  | 0.052 |  | 163.372 |  | 1.978 |  |
| Condition + Time + Volunteers + Condition  ✻  Time |  | 0.053 |  | 0.002 |  | 0.038 |  | 223.500 |  | 2.421 |  |
| Condition + Time + Volunteers + Time  ✻  Volunteers |  | 0.053 |  | 0.002 |  | 0.038 |  | 227.527 |  | 1.475 |  |
| Condition + Time + Volunteers + Condition  ✻  Time + Time  ✻  Volunteers |  | 0.053 |  | 0.001 |  | 0.021 |  | 410.447 |  | 4.793 |  |
|  | | | | | | | | | | | |
| Note.  All models include subject | | | | | | | | | | | |

| **Post Hoc Comparisons - Condition** | | | | | | | | | | | |
| --- | --- | --- | --- | --- | --- | --- | --- | --- | --- | --- | --- |
|  | |  | | **Prior Odds** | | **Posterior Odds** | | **BF _01, U_** | | **error %** | |
| taVNS |  | Sham |  | 1.000 |  | 4.493 |  | 4.493 |  | 1.141e -5 |  |
|  | | | | | | | | | | | |
|  | | | | | | | | | | | |

### Model Averaged Q-Q Plot

| **Post Hoc Comparisons - Time** | | | | | | | | | | | |
| --- | --- | --- | --- | --- | --- | --- | --- | --- | --- | --- | --- |
|  | |  | | **Prior Odds** | | **Posterior Odds** | | **BF _01, U_** | | **error %** | |
| T1-T0 |  | T2-T0 |  | 1.000 |  | 4.105 |  | 4.105 |  | 1.070e -5 |  |
|  | | | | | | | | | | | |
| Note.  The posterior odds have been corrected for multiple testing by fixing to 0.5 the prior probability that the null hypothesis holds across all comparisons (Westfall, Johnson, & Utts, 1997). Individual comparisons are based on the default t-test with a Cauchy (0, r = 1/sqrt(2)) prior. The "U" in the Bayes factor denotes that it is uncorrected. | | | | | | | | | | | |


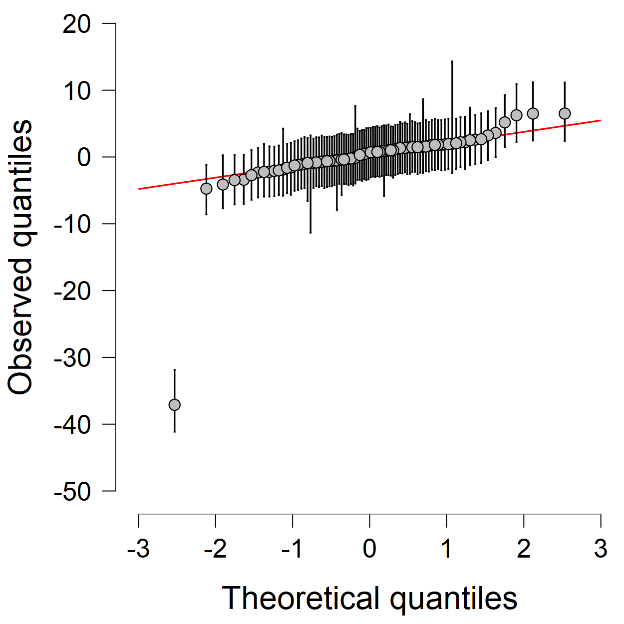


- ***Heat sensitive Aδ-fibers***

| **Model Comparison** | | | | | | | | | | | |
| --- | --- | --- | --- | --- | --- | --- | --- | --- | --- | --- | --- |
| **Models** | | **P(M)** | | **P(M\|data)** | | **BF _M_** | | **BF _01_** | | **error %** | |
| Null model (incl. subject) |  | 0.053 |  | 0.485 |  | 16.965 |  | 1.000 |  |  |  |
| Time |  | 0.053 |  | 0.198 |  | 4.431 |  | 2.456 |  | 0.976 |  |
| Condition |  | 0.053 |  | 0.118 |  | 2.401 |  | 4.123 |  | 3.042 |  |
| Volunteers |  | 0.053 |  | 0.048 |  | 0.911 |  | 10.076 |  | 0.191 |  |
| Condition + Time |  | 0.053 |  | 0.048 |  | 0.903 |  | 10.153 |  | 2.380 |  |
| Time + Volunteers |  | 0.053 |  | 0.020 |  | 0.359 |  | 24.837 |  | 0.897 |  |
| Condition + Time + Condition  ✻  Time |  | 0.053 |  | 0.019 |  | 0.348 |  | 25.580 |  | 4.528 |  |
| Condition + Time + Volunteers + Condition  ✻  Time + Condition  ✻  Volunteers + Time  ✻  Volunteers + Condition  ✻  Time  ✻  Volunteers |  | 0.053 |  | 0.013 |  | 0.230 |  | 38.523 |  | 21.512 |  |
| Condition + Volunteers |  | 0.053 |  | 0.012 |  | 0.212 |  | 41.592 |  | 1.175 |  |
| Time + Volunteers + Time  ✻  Volunteers |  | 0.053 |  | 0.011 |  | 0.194 |  | 45.514 |  | 0.871 |  |
| Condition + Volunteers + Condition  ✻  Volunteers |  | 0.053 |  | 0.009 |  | 0.161 |  | 54.820 |  | 2.382 |  |
| Condition + Time + Volunteers |  | 0.053 |  | 0.005 |  | 0.088 |  | 99.515 |  | 1.869 |  |
| Condition + Time + Volunteers + Condition  ✻  Volunteers |  | 0.053 |  | 0.004 |  | 0.069 |  | 126.791 |  | 1.490 |  |
| Condition + Time + Volunteers + Condition  ✻  Volunteers + Time  ✻  Volunteers |  | 0.053 |  | 0.003 |  | 0.063 |  | 138.732 |  | 1.759 |  |
| Condition + Time + Volunteers + Time  ✻  Volunteers |  | 0.053 |  | 0.003 |  | 0.047 |  | 187.984 |  | 1.364 |  |
| Condition + Time + Volunteers + Condition  ✻  Time + Condition  ✻  Volunteers + Time  ✻  Volunteers |  | 0.053 |  | 0.002 |  | 0.036 |  | 245.575 |  | 19.150 |  |
| Condition + Time + Volunteers + Condition  ✻  Time |  | 0.053 |  | 0.002 |  | 0.034 |  | 259.515 |  | 2.476 |  |
| Condition + Time + Volunteers + Condition  ✻  Time + Condition  ✻  Volunteers |  | 0.053 |  | 0.002 |  | 0.030 |  | 288.629 |  | 5.177 |  |
| Condition + Time + Volunteers + Condition  ✻  Time + Time  ✻  Volunteers |  | 0.053 |  | 0.001 |  | 0.019 |  | 470.455 |  | 2.078 |  |
|  | | | | | | | | | | | |
| Note.  All models include subject | | | | | | | | | | | |

###

| **Post Hoc Comparisons - Condition** | | | | | | | | | | | |
| --- | --- | --- | --- | --- | --- | --- | --- | --- | --- | --- | --- |
|  | |  | | **Prior Odds** | | **Posterior Odds** | | **BF _01, U_** | | **error %** | |
| taVNS |  | Sham |  | 1.000 |  | 5.617 |  | 5.617 |  | 1.327e -5 |  |
|  | | | | | | | | | | | |
| \| **Post Hoc Comparisons - Time** \| \| \| \| \| \| \| \| \| \| \| \| \| --- \| --- \| --- \| --- \| --- \| --- \| --- \| --- \| --- \| --- \| --- \| --- \| \|  \| \|  \| \| **Prior Odds** \| \| **Posterior Odds** \| \| **BF _01, U_** \| \| **error %** \| \| \| T1-T0 \|  \| T2-T0 \|  \| 1.000 \|  \| 3.435 \|  \| 3.435 \|  \| 9.383e -6 \|  \| \|  \| \| \| \| \| \| \| \| \| \| \| \| \| Note.  The posterior odds have been corrected for multiple testing by fixing to 0.5 the prior probability that the null hypothesis holds across all comparisons (Westfall, Johnson, & Utts, 1997). Individual comparisons are based on the default t-test with a Cauchy (0, r = 1/sqrt(2)) prior. The "U" in the Bayes factor denotes that it is uncorrected. \| \| \| \| \| \| \| \| \| \| \| \| | | | | | | | | | | | |

### Model Averaged Q-Q Plot


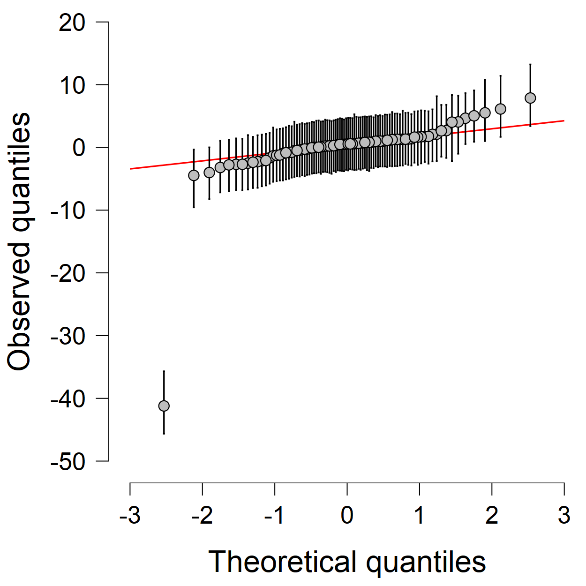


- ***Mechanosensitive Aβ-fibers***

| **Model Comparison** | | | | | | | | | | | |
| --- | --- | --- | --- | --- | --- | --- | --- | --- | --- | --- | --- |
| **Models** | | **P(M)** | | **P(M\|data)** | | **BF _M_** | | **BF _01_** | | **error %** | |
| Null model (incl. subject) |  | 0.053 |  | 0.263 |  | 6.434 |  | 1.000 |  |  |  |
| Condition |  | 0.053 |  | 0.191 |  | 4.249 |  | 1.379 |  | 0.878 |  |
| Volunteers |  | 0.053 |  | 0.123 |  | 2.517 |  | 2.146 |  | 0.974 |  |
| Condition + Volunteers |  | 0.053 |  | 0.099 |  | 1.968 |  | 2.672 |  | 4.500 |  |
| Time |  | 0.053 |  | 0.066 |  | 1.278 |  | 3.973 |  | 1.576 |  |
| Condition + Volunteers + Condition  ✻  Volunteers |  | 0.053 |  | 0.060 |  | 1.154 |  | 4.369 |  | 3.448 |  |
| Condition + Time |  | 0.053 |  | 0.047 |  | 0.891 |  | 5.582 |  | 1.337 |  |
| Time + Volunteers |  | 0.053 |  | 0.030 |  | 0.558 |  | 8.752 |  | 1.656 |  |
| Condition + Time + Volunteers |  | 0.053 |  | 0.024 |  | 0.443 |  | 10.959 |  | 5.057 |  |
| Condition + Time + Condition  ✻  Time |  | 0.053 |  | 0.019 |  | 0.358 |  | 13.518 |  | 2.167 |  |
| Condition + Time + Volunteers + Condition  ✻  Volunteers |  | 0.053 |  | 0.014 |  | 0.264 |  | 18.188 |  | 2.506 |  |
| Time + Volunteers + Time  ✻  Volunteers |  | 0.053 |  | 0.012 |  | 0.216 |  | 22.229 |  | 2.589 |  |
| Condition + Time + Volunteers + Condition  ✻  Time |  | 0.053 |  | 0.011 |  | 0.198 |  | 24.188 |  | 10.197 |  |
| Condition + Time + Volunteers + Time  ✻  Volunteers |  | 0.053 |  | 0.011 |  | 0.197 |  | 24.287 |  | 5.204 |  |
| Condition + Time + Volunteers + Condition  ✻  Volunteers + Time  ✻  Volunteers |  | 0.053 |  | 0.010 |  | 0.184 |  | 26.005 |  | 2.821 |  |
| Condition + Time + Volunteers + Condition  ✻  Time + Condition  ✻  Volunteers |  | 0.053 |  | 0.006 |  | 0.117 |  | 40.752 |  | 11.386 |  |
| Condition + Time + Volunteers + Condition  ✻  Time + Condition  ✻  Volunteers + Time  ✻  Volunteers |  | 0.053 |  | 0.005 |  | 0.082 |  | 58.279 |  | 8.521 |  |
| Condition + Time + Volunteers + Condition  ✻  Time + Condition  ✻  Volunteers + Time  ✻  Volunteers + Condition  ✻  Time  ✻  Volunteers |  | 0.053 |  | 0.004 |  | 0.074 |  | 64.150 |  | 4.477 |  |
| Condition + Time + Volunteers + Condition  ✻  Time + Time  ✻  Volunteers |  | 0.053 |  | 0.004 |  | 0.071 |  | 67.225 |  | 2.712 |  |
|  | | | | | | | | | | | |
| Note.  All models include subject | | | | | | | | | | | |

| **Post Hoc Comparisons - Condition** | | | | | | | | | | | |
| --- | --- | --- | --- | --- | --- | --- | --- | --- | --- | --- | --- |
|  | |  | | **Prior Odds** | | **Posterior Odds** | | **BF _01, U_** | | **error %** | |
| taVNS |  | Sham |  | 1.000 |  | 1.744 |  | 1.744 |  | 5.327e -6 |  |
|  | | | | | | | | | | | |

### Model Averaged Q-Q Plot

| **Post Hoc Comparisons - Time** | | | | | | | | | | | |
| --- | --- | --- | --- | --- | --- | --- | --- | --- | --- | --- | --- |
|  | |  | | **Prior Odds** | | **Posterior Odds** | | **BF _01, U_** | | **error %** | |
| T1-T0 |  | T2-T0 |  | 1.000 |  | 5.362 |  | 5.362 |  | 1.287e -5 |  |
|  | | | | | | | | | | | |
| Note.  The posterior odds have been corrected for multiple testing by fixing to 0.5 the prior probability that the null hypothesis holds across all comparisons (Westfall, Johnson, & Utts, 1997). Individual comparisons are based on the default t-test with a Cauchy (0, r = 1/sqrt(2)) prior. The "U" in the Bayes factor denotes that it is uncorrected. | | | | | | | | | | | |


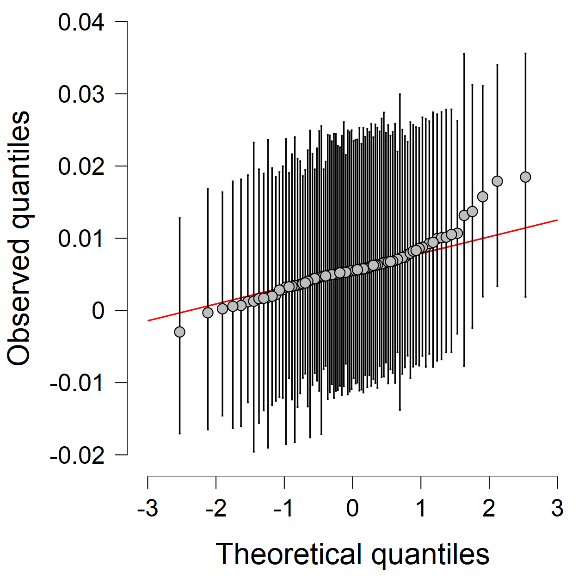


- ***Cool sensitive Aδ-fibers***

| **Model Comparison** | | | | | | | | | | | |
| --- | --- | --- | --- | --- | --- | --- | --- | --- | --- | --- | --- |
| **Models** | **P(M)** | | | **P(M\|data)** | | **BF _M_** | | **BF _01_** | | **error %** | |
| Null model (incl. subject) |  | 0.053 |  | 0.463 |  | 15.537 |  | 1.000 |  |  |  |
| Time |  | 0.053 |  | 0.155 |  | 3.313 |  | 2.980 |  | 2.355 |  |
| Condition |  | 0.053 |  | 0.150 |  | 3.189 |  | 3.078 |  | 1.022 |  |
| Volunteers |  | 0.053 |  | 0.055 |  | 1.043 |  | 8.459 |  | 0.173 |  |
| Condition + Time |  | 0.053 |  | 0.050 |  | 0.948 |  | 9.264 |  | 1.442 |  |
| Condition + Time + Condition  ✻  Time |  | 0.053 |  | 0.026 |  | 0.488 |  | 17.561 |  | 14.077 |  |
| Condition + Volunteers |  | 0.053 |  | 0.018 |  | 0.338 |  | 25.123 |  | 2.429 |  |
| Time + Volunteers |  | 0.053 |  | 0.018 |  | 0.335 |  | 25.376 |  | 1.250 |  |
| Condition + Volunteers + Condition  ✻  Volunteers |  | 0.053 |  | 0.017 |  | 0.306 |  | 27.720 |  | 1.629 |  |
| Condition + Time + Volunteers + Condition  ✻  Time + Condition  ✻  Volunteers + Time  ✻  Volunteers + Condition  ✻  Time  ✻  Volunteers |  | 0.053 |  | 0.009 |  | 0.157 |  | 53.477 |  | 25.915 |  |
| Time + Volunteers + Time  ✻  Volunteers |  | 0.053 |  | 0.008 |  | 0.148 |  | 56.769 |  | 3.076 |  |
| Condition + Time + Volunteers |  | 0.053 |  | 0.007 |  | 0.123 |  | 68.214 |  | 9.548 |  |
| Condition + Time + Volunteers + Condition  ✻  Volunteers |  | 0.053 |  | 0.006 |  | 0.117 |  | 71.992 |  | 6.875 |  |
| Condition + Time + Volunteers + Condition  ✻  Volunteers + Time  ✻  Volunteers |  | 0.053 |  | 0.004 |  | 0.080 |  | 105.309 |  | 2.423 |  |
| Condition + Time + Volunteers + Condition  ✻  Time |  | 0.053 |  | 0.003 |  | 0.051 |  | 165.446 |  | 4.700 |  |
| Condition + Time + Volunteers + Time  ✻  Volunteers |  | 0.053 |  | 0.003 |  | 0.050 |  | 168.309 |  | 1.591 |  |
| Condition + Time + Volunteers + Condition  ✻  Time + Condition  ✻  Volunteers |  | 0.053 |  | 0.003 |  | 0.049 |  | 171.250 |  | 2.292 |  |
| Condition + Time + Volunteers + Condition  ✻  Time + Condition  ✻  Volunteers + Time  ✻  Volunteers |  | 0.053 |  | 0.002 |  | 0.041 |  | 205.425 |  | 2.704 |  |
| Condition + Time + Volunteers + Condition  ✻  Time + Time  ✻  Volunteers |  | 0.053 |  | 0.001 |  | 0.022 |  | 374.635 |  | 2.179 |  |
|  | | | | | | | | | | | |
| Note.  All models include subject | | | | | | | | | | | |

| **Post Hoc Comparisons - Condition** | | | | | | | | | | | |
| --- | --- | --- | --- | --- | --- | --- | --- | --- | --- | --- | --- |
|  | |  | | **Prior Odds** | | **Posterior Odds** | | **BF _01, U_** | | **error %** | |
| taVNS |  | Sham |  | 1.000 |  | 4.275 |  | 4.275 |  | 1.101e -5 |  |
|  | | | | | | | | | | | |
| \| **Post Hoc Comparisons - Time** \| \| \| \| \| \| \| \| \| \| \| \| \| --- \| --- \| --- \| --- \| --- \| --- \| --- \| --- \| --- \| --- \| --- \| --- \| \|  \| \|  \| \| **Prior Odds** \| \| **Posterior Odds** \| \| **BF _01, U_** \| \| **error %** \| \| \| T1-T0 \|  \| T2-T0 \|  \| 1.000 \|  \| 4.080 \|  \| 4.080 \|  \| 1.065e -5 \|  \| \|  \| \| \| \| \| \| \| \| \| \| \| \| \| Note.  The posterior odds have been corrected for multiple testing by fixing to 0.5 the prior probability that the null hypothesis holds across all comparisons (Westfall, Johnson, & Utts, 1997). Individual comparisons are based on the default t-test with a Cauchy (0, r = 1/sqrt(2)) prior. The "U" in the Bayes factor denotes that it is uncorrected. \| \| \| \| \| \| \| \| \| \| \| \| | | | | | | | | | | | |

### Model Averaged Q-Q Plots
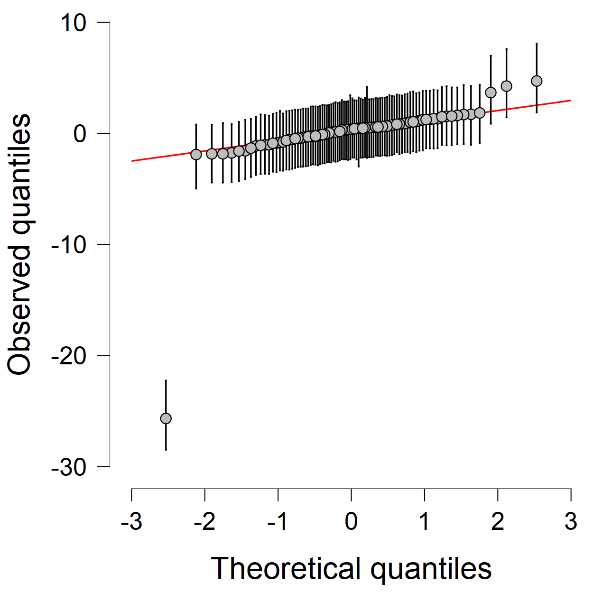


#### **Perception Intensity**

- ***Laser***

| **Model Comparison** | | | | | | | | | | | |
| --- | --- | --- | --- | --- | --- | --- | --- | --- | --- | --- | --- |
| **Models** | **P(M)** | | | **P(M\|data)** | | **BF _M_** | | **BF _01_** | | **error %** | |
| Null model (incl. subject) |  | 0.053 |  | 0.019 |  | 0.353 |  | 1.000 |  |  |  |
| Condition + Volunteers + Condition  ✻  Volunteers |  | 0.053 |  | 0.484 |  | 16.853 |  | 0.040 |  | 3.715 |  |
| Condition + Time + Volunteers + Condition  ✻  Volunteers + Time  ✻  Volunteers |  | 0.053 |  | 0.159 |  | 3.406 |  | 0.121 |  | 4.774 |  |
| Condition + Time + Volunteers + Condition  ✻  Volunteers |  | 0.053 |  | 0.153 |  | 3.261 |  | 0.125 |  | 7.267 |  |
| Condition + Time + Volunteers + Condition  ✻  Time + Condition  ✻  Volunteers + Time  ✻  Volunteers |  | 0.053 |  | 0.078 |  | 1.519 |  | 0.247 |  | 19.040 |  |
| Condition + Time + Volunteers + Condition  ✻  Time + Condition  ✻  Volunteers |  | 0.053 |  | 0.048 |  | 0.908 |  | 0.401 |  | 5.369 |  |
| Condition + Time + Volunteers + Condition  ✻  Time + Condition  ✻  Volunteers + Time  ✻  Volunteers + Condition  ✻  Time  ✻  Volunteers |  | 0.053 |  | 0.033 |  | 0.606 |  | 0.591 |  | 14.171 |  |
| Volunteers |  | 0.053 |  | 0.010 |  | 0.176 |  | 1.991 |  | 1.503 |  |
| Time |  | 0.053 |  | 0.005 |  | 0.086 |  | 4.034 |  | 1.344 |  |
| Condition |  | 0.053 |  | 0.005 |  | 0.084 |  | 4.151 |  | 2.132 |  |
| Time + Volunteers |  | 0.053 |  | 0.002 |  | 0.041 |  | 8.454 |  | 1.535 |  |
| Condition + Volunteers |  | 0.053 |  | 0.002 |  | 0.039 |  | 8.908 |  | 1.597 |  |
| Condition + Time |  | 0.053 |  | 0.001 |  | 0.020 |  | 17.036 |  | 2.668 |  |
| Condition + Time + Volunteers |  | 0.053 |  | 5.999e -4 |  | 0.011 |  | 32.069 |  | 3.218 |  |
| Time + Volunteers + Time  ✻  Volunteers |  | 0.053 |  | 4.411e -4 |  | 0.008 |  | 43.612 |  | 2.190 |  |
| Condition + Time + Condition  ✻  Time |  | 0.053 |  | 3.253e -4 |  | 0.006 |  | 59.141 |  | 1.780 |  |
| Condition + Time + Volunteers + Condition  ✻  Time |  | 0.053 |  | 1.750e -4 |  | 0.003 |  | 109.920 |  | 6.222 |  |
| Condition + Time + Volunteers + Time  ✻  Volunteers |  | 0.053 |  | 1.065e -4 |  | 0.002 |  | 180.717 |  | 4.981 |  |
| Condition + Time + Volunteers + Condition  ✻  Time + Time  ✻  Volunteers |  | 0.053 |  | 3.152e -5 |  | 5.674e -4 |  | 610.408 |  | 2.897 |  |
|  | | | | | | | | | | | |
| Note.  All models include subject | | | | | | | | | | | |

| **Post Hoc Comparisons - Time** | | | | | | | | | | | |
| --- | --- | --- | --- | --- | --- | --- | --- | --- | --- | --- | --- |
|  | |  | | **Prior Odds** | | **Posterior Odds** | | **BF _01, U_** | | **error %** | |
| T1-T0 |  | T2-T0 |  | 1.000 |  | 4.833 |  | 4.833 |  | 1.200e -5 |  |
|  | | | | | | | | | | | |
| \| **Post Hoc Comparisons - Condition** \| \| \| \| \| \| \| \| \| \| \| \| \| --- \| --- \| --- \| --- \| --- \| --- \| --- \| --- \| --- \| --- \| --- \| --- \| \|  \| \|  \| \| **Prior Odds** \| \| **Posterior Odds** \| \| **BF _01, U_** \| \| **error %** \| \| \| taVNS \|  \| Sham \|  \| 1.000 \|  \| 5.827 \|  \| 5.827 \|  \| 1.359e -5 \|  \| \|  \| \| \| \| \| \| \| \| \| \| \| \|   Note.  The posterior odds have been corrected for multiple testing by fixing to 0.5 the prior probability that the null hypothesis holds across all comparisons (Westfall, Johnson, & Utts, 1997). Individual comparisons are based on the default t-test with a Cauchy (0, r = 1/sqrt(2)) prior. The "U" in the Bayes factor denotes that it is uncorrected. | | | | | | | | | | | |

### Model Averaged Q-Q Plot


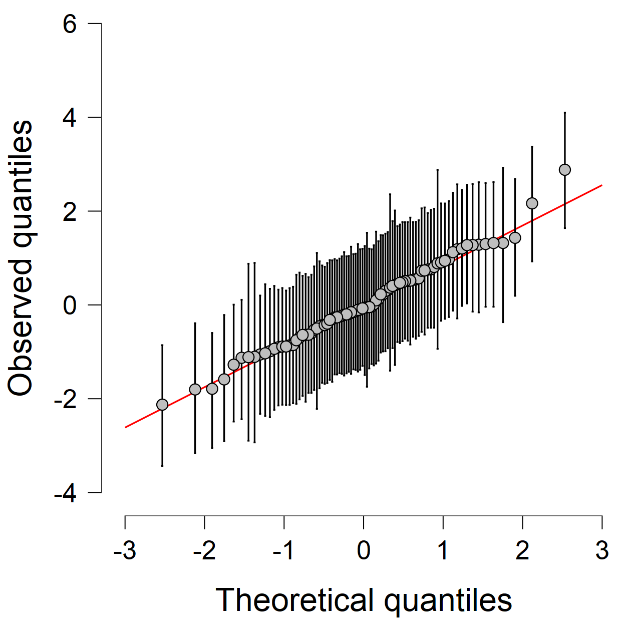


- ***Vibrotactile***

| **Model Comparison** | | | | | | | | | | | | | | | | |  |
| --- | --- | --- | --- | --- | --- | --- | --- | --- | --- | --- | --- | --- | --- | --- | --- | --- | --- |
| **Models** | | **P(M)** | | **P(M\|data)** | | | **BF _M_** | | | | **BF _01_** | | **error %** | | | |  |
| Null model (incl. subject) |  | | 0.053 | |  | 7.751e -7 | |  | 1.395e -5 |  | | 1.000 | |  |  |  | |
| Condition + Time + Volunteers + Condition  ✻  Time + Condition  ✻  Volunteers |  | | 0.053 | |  | 0.534 | |  | 20.636 |  | | 1.451e -6 | |  | 94.448 |  | |
| Condition + Volunteers + Condition  ✻  Volunteers |  | | 0.053 | |  | 0.359 | |  | 10.092 |  | | 2.158e -6 | |  | 15.563 |  | |
| Condition + Time + Volunteers + Condition  ✻  Volunteers |  | | 0.053 | |  | 0.081 | |  | 1.591 |  | | 9.543e -6 | |  | 5.168 |  | |
| Condition + Time + Volunteers + Condition  ✻  Volunteers + Time  ✻  Volunteers |  | | 0.053 | |  | 0.011 | |  | 0.194 |  | | 7.251e -5 | |  | 10.555 |  | |
| Condition + Time + Volunteers + Condition  ✻  Time + Condition  ✻  Volunteers + Time  ✻  Volunteers + Condition  ✻  Time  ✻  Volunteers |  | | 0.053 | |  | 0.009 | |  | 0.162 |  | | 8.689e -5 | |  | 7.579 |  | |
| Condition + Time + Volunteers + Condition  ✻  Time + Condition  ✻  Volunteers + Time  ✻  Volunteers |  | | 0.053 | |  | 0.006 | |  | 0.105 |  | | 1.336e -4 | |  | 18.894 |  | |
| Condition |  | | 0.053 | |  | 7.225e -6 | |  | 1.300e -4 |  | | 0.107 | |  | 1.107 |  | |
| Condition + Volunteers |  | | 0.053 | |  | 3.210e -6 | |  | 5.779e -5 |  | | 0.241 | |  | 1.435 |  | |
| Condition + Time |  | | 0.053 | |  | 1.560e -6 | |  | 2.808e -5 |  | | 0.497 | |  | 1.951 |  | |
| Condition + Time + Volunteers |  | | 0.053 | |  | 7.282e -7 | |  | 1.311e -5 |  | | 1.064 | |  | 4.082 |  | |
| Condition + Time + Condition  ✻  Time |  | | 0.053 | |  | 6.028e -7 | |  | 1.085e -5 |  | | 1.286 | |  | 10.091 |  | |
| Volunteers |  | | 0.053 | |  | 3.078e -7 | |  | 5.540e -6 |  | | 2.519 | |  | 0.733 |  | |
| Condition + Time + Volunteers + Condition  ✻  Time |  | | 0.053 | |  | 2.466e -7 | |  | 4.439e -6 |  | | 3.143 | |  | 2.466 |  | |
| Time |  | | 0.053 | |  | 1.711e -7 | |  | 3.079e -6 |  | | 4.531 | |  | 1.148 |  | |
| Time + Volunteers |  | | 0.053 | |  | 6.504e -8 | |  | 1.171e -6 |  | | 11.917 | |  | 1.281 |  | |
| Condition + Time + Volunteers + Time  ✻  Volunteers |  | | 0.053 | |  | 5.453e -8 | |  | 9.816e -7 |  | | 14.213 | |  | 4.145 |  | |
| Condition + Time + Volunteers + Condition  ✻  Time + Time  ✻  Volunteers |  | | 0.053 | |  | 1.814e -8 | |  | 3.265e -7 |  | | 42.729 | |  | 1.983 |  | |
| Time + Volunteers + Time  ✻  Volunteers |  | | 0.053 | |  | 4.550e -9 | |  | 8.190e -8 |  | | 170.355 | |  | 1.130 |  | |
|  | | | | | | | | | | | | | | | | |  |
| Note.  All models include subject | | | | | | | | | | | | | | | | |  |

| **Post Hoc Comparisons - Condition** | | | | | | | | | | | |
| --- | --- | --- | --- | --- | --- | --- | --- | --- | --- | --- | --- |
|  | |  | | **Prior Odds** | | **Posterior Odds** | | **BF _01, U_** | | **error %** | |
| taVNS |  | Sham |  | 1.000 |  | 0.378 |  | 0.378 |  | 9.801e -7 |  |
|  | | | | | | | | | | | |
| \| **Post Hoc Comparisons - Time** \| \| \| \| \| \| \| \| \| \| \| \| \| --- \| --- \| --- \| --- \| --- \| --- \| --- \| --- \| --- \| --- \| --- \| --- \| \|  \| \|  \| \| **Prior Odds** \| \| **Posterior Odds** \| \| **BF _01, U_** \| \| **error %** \| \| \| T1-T0 \|  \| T2-T0 \|  \| 1.000 \|  \| 6.071 \|  \| 6.071 \|  \| 1.394e -5 \|  \| \|  \| \| \| \| \| \| \| \| \| \| \| \| \| Note.  The posterior odds have been corrected for multiple testing by fixing to 0.5 the prior probability that the null hypothesis holds across all comparisons (Westfall, Johnson, & Utts, 1997). Individual comparisons are based on the default t-test with a Cauchy (0, r = 1/sqrt(2)) prior. The "U" in the Bayes factor denotes that it is uncorrected. \| \| \| \| \| \| \| \| \| \| \| \| | | | | | | | | | | | |

### Model Averaged Q-Q Plot


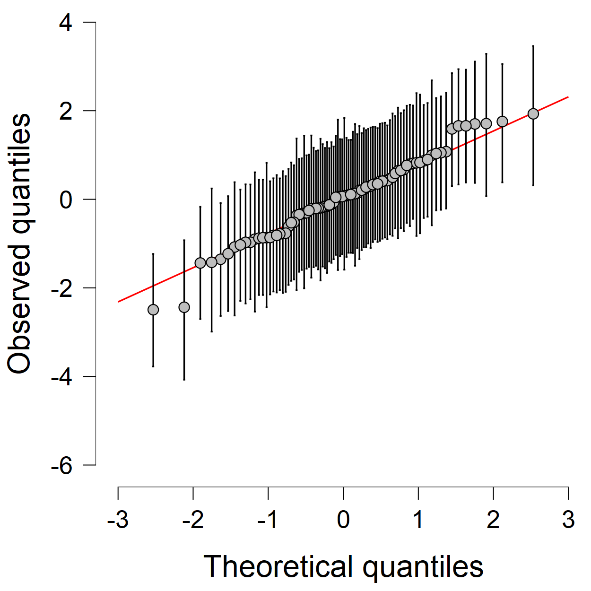


- ***Cool***

| **Model Comparison** | | | | | | | | | | | | |  |  |
| --- | --- | --- | --- | --- | --- | --- | --- | --- | --- | --- | --- | --- | --- | --- |
| **Models** | **P(M)** | | | **P(M\|data)** | | **BF _M_** | | **BF _01_** | | **error %** | | |  |  |
| Null model (incl. subject) |  | 0.053 |  | 1.592e -6 |  | | 2.866e -5 |  | 1.000 | |  |  | |  |
| Condition + Volunteers + Condition  ✻  Volunteers |  | 0.053 |  | 0.261 |  | | 6.362 |  | 6.097e -6 | |  | 16.894 | |  |
| Condition + Time + Volunteers + Condition  ✻  Volunteers + Time  ✻  Volunteers |  | 0.053 |  | 0.226 |  | | 5.253 |  | 7.049e -6 | |  | 11.009 | |  |
| Condition + Time + Volunteers + Condition  ✻  Volunteers |  | 0.053 |  | 0.216 |  | | 4.954 |  | 7.378e -6 | |  | 4.541 | |  |
| Condition + Time + Volunteers + Condition  ✻  Time + Condition  ✻  Volunteers |  | 0.053 |  | 0.127 |  | | 2.607 |  | 1.259e -5 | |  | 10.136 | |  |
| Condition + Time + Volunteers + Condition  ✻  Time + Condition  ✻  Volunteers + Time  ✻  Volunteers |  | 0.053 |  | 0.123 |  | | 2.522 |  | 1.296e -5 | |  | 7.915 | |  |
| Condition + Time + Volunteers + Condition  ✻  Time + Condition  ✻  Volunteers + Time  ✻  Volunteers + Condition  ✻  Time  ✻  Volunteers |  | 0.053 |  | 0.048 |  | | 0.902 |  | 3.336e -5 | |  | 7.665 | |  |
| Condition |  | 0.053 |  | 4.300e -6 |  | | 7.740e -5 |  | 0.370 | |  | 1.504 | |  |
| Condition + Volunteers |  | 0.053 |  | 2.321e -6 |  | | 4.178e -5 |  | 0.686 | |  | 2.330 | |  |
| Condition + Time |  | 0.053 |  | 1.800e -6 |  | | 3.241e -5 |  | 0.884 | |  | 2.351 | |  |
| Condition + Time + Volunteers |  | 0.053 |  | 9.214e -7 |  | | 1.658e -5 |  | 1.728 | |  | 1.908 | |  |
| Volunteers |  | 0.053 |  | 7.766e -7 |  | | 1.398e -5 |  | 2.050 | |  | 1.219 | |  |
| Condition + Time + Condition  ✻  Time |  | 0.053 |  | 6.278e -7 |  | | 1.130e -5 |  | 2.536 | |  | 2.423 | |  |
| Time |  | 0.053 |  | 6.262e -7 |  | | 1.127e -5 |  | 2.543 | |  | 1.256 | |  |
| Time + Volunteers |  | 0.053 |  | 3.342e -7 |  | | 6.016e -6 |  | 4.764 | |  | 4.515 | |  |
| Condition + Time + Volunteers + Condition  ✻  Time |  | 0.053 |  | 3.313e -7 |  | | 5.964e -6 |  | 4.806 | |  | 2.644 | |  |
| Condition + Time + Volunteers + Time  ✻  Volunteers |  | 0.053 |  | 9.782e -8 |  | | 1.761e -6 |  | 16.277 | |  | 2.903 | |  |
| Condition + Time + Volunteers + Condition  ✻  Time + Time  ✻  Volunteers |  | 0.053 |  | 3.477e -8 |  | | 6.259e -7 |  | 45.793 | |  | 3.316 | |  |
| Time + Volunteers + Time  ✻  Volunteers |  | 0.053 |  | 2.792e -8 |  | | 5.025e -7 |  | 57.032 | |  | 2.290 | |  |
|  | | | | | | | | | | | | |  |  |
| Note.  All models include subject | | | | | | | | | | | | |  |  |

| **Post Hoc Comparisons - Condition** | | | | | | | | | | | |
| --- | --- | --- | --- | --- | --- | --- | --- | --- | --- | --- | --- |
|  | |  | | **Prior Odds** | | **Posterior Odds** | | **BF _01, U_** | | **error %** | |
| taVNS |  | Sham |  | 1.000 |  | 0.775 |  | 0.775 |  | 2.346e -6 |  |
|  | | | | | | | | | | | |

### Model Averaged Q-Q Plot

| **Post Hoc Comparisons - Time** | | | | | | | | | | | |
| --- | --- | --- | --- | --- | --- | --- | --- | --- | --- | --- | --- |
|  | |  | | **Prior Odds** | | **Posterior Odds** | | **BF _01, U_** | | **error %** | |
| T1-T0 |  | T2-T0 |  | 1.000 |  | 0.954 |  | 0.954 |  | 2.943e -6 |  |
|  | | | | | | | | | | | |
| Note.  The posterior odds have been corrected for multiple testing by fixing to 0.5 the prior probability that the null hypothesis holds across all comparisons (Westfall, Johnson, & Utts, 1997). Individual comparisons are based on the default t-test with a Cauchy (0, r = 1/sqrt(2)) prior. The "U" in the Bayes factor denotes that it is uncorrected. | | | | | | | | | | | |


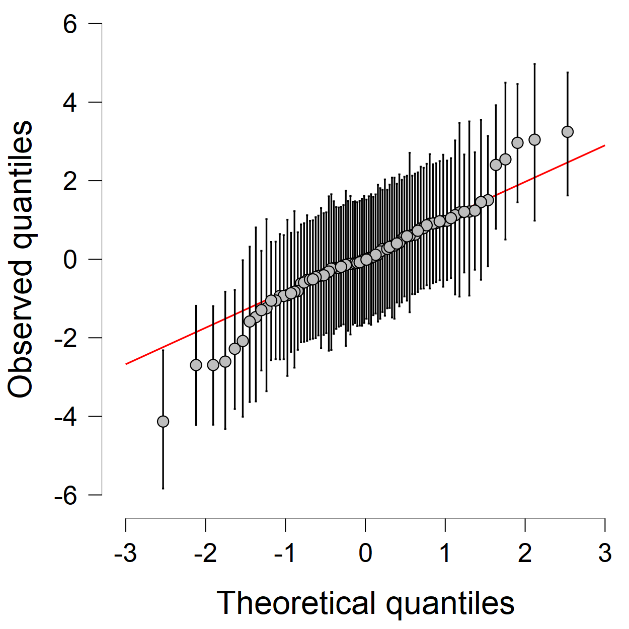


- ***Pinprick***

| **Model Comparison** | | | | | | | | | | | |
| --- | --- | --- | --- | --- | --- | --- | --- | --- | --- | --- | --- |
| **Models** | | **P(M)** | | **P(M\|data)** | | **BF _M_** | | **BF _01_** | | **error %** | |
| Null model (incl. subject) |  | 0.053 |  | 0.249 |  | 5.958 |  | 1.000 |  |  |  |
| Condition + Volunteers + Condition  ✻  Volunteers |  | 0.053 |  | 0.257 |  | 6.223 |  | 0.968 |  | 6.216 |  |
| Volunteers |  | 0.053 |  | 0.109 |  | 2.199 |  | 2.284 |  | 0.848 |  |
| Condition + Time + Volunteers + Condition  ✻  Volunteers |  | 0.053 |  | 0.076 |  | 1.488 |  | 3.257 |  | 21.462 |  |
| Condition |  | 0.053 |  | 0.074 |  | 1.438 |  | 3.362 |  | 3.491 |  |
| Time |  | 0.053 |  | 0.056 |  | 1.074 |  | 4.417 |  | 1.087 |  |
| Condition + Time + Volunteers + Condition  ✻  Time + Condition  ✻  Volunteers |  | 0.053 |  | 0.037 |  | 0.690 |  | 6.732 |  | 6.370 |  |
| Condition + Volunteers |  | 0.053 |  | 0.031 |  | 0.582 |  | 7.937 |  | 1.435 |  |
| Time + Volunteers |  | 0.053 |  | 0.025 |  | 0.468 |  | 9.803 |  | 1.588 |  |
| Condition + Time + Volunteers + Condition  ✻  Volunteers + Time  ✻  Volunteers |  | 0.053 |  | 0.018 |  | 0.338 |  | 13.483 |  | 2.767 |  |
| Condition + Time |  | 0.053 |  | 0.016 |  | 0.292 |  | 15.577 |  | 2.282 |  |
| Condition + Time + Volunteers + Condition  ✻  Time + Condition  ✻  Volunteers + Time  ✻  Volunteers + Condition  ✻  Time  ✻  Volunteers |  | 0.053 |  | 0.013 |  | 0.245 |  | 18.523 |  | 5.198 |  |
| Condition + Time + Volunteers + Condition  ✻  Time + Condition  ✻  Volunteers + Time  ✻  Volunteers |  | 0.053 |  | 0.013 |  | 0.236 |  | 19.225 |  | 3.883 |  |
| Condition + Time + Condition  ✻  Time |  | 0.053 |  | 0.008 |  | 0.142 |  | 31.879 |  | 1.673 |  |
| Condition + Time + Volunteers |  | 0.053 |  | 0.007 |  | 0.124 |  | 36.286 |  | 1.584 |  |
| Time + Volunteers + Time  ✻  Volunteers |  | 0.053 |  | 0.004 |  | 0.077 |  | 58.041 |  | 1.615 |  |
| Condition + Time + Volunteers + Condition  ✻  Time |  | 0.053 |  | 0.004 |  | 0.065 |  | 68.591 |  | 2.645 |  |
| Condition + Time + Volunteers + Time  ✻  Volunteers |  | 0.053 |  | 0.001 |  | 0.023 |  | 193.355 |  | 4.131 |  |
| Condition + Time + Volunteers + Condition  ✻  Time + Time  ✻  Volunteers |  | 0.053 |  | 6.406e -4 |  | 0.012 |  | 388.187 |  | 3.158 |  |
|  | | | | | | | | | | | |
| Note.  All models include subject | | | | | | | | | | | |

| **Post Hoc Comparisons - Condition** | | | | | | | | | | | |
| --- | --- | --- | --- | --- | --- | --- | --- | --- | --- | --- | --- |
|  | |  | | **Prior Odds** | | **Posterior Odds** | | **BF _01, U_** | | **error %** | |
| taVNS |  | Sham |  | 1.000 |  | 4.874 |  | 4.874 |  | 1.207e -5 |  |
|  | | | | | | | | | | | |
|  | | | | | | | | | | | |

### Model Averaged Q-Q Plot

| **Post Hoc Comparisons - Time** | | | | | | | | | | | |
| --- | --- | --- | --- | --- | --- | --- | --- | --- | --- | --- | --- |
|  | |  | | **Prior Odds** | | **Posterior Odds** | | **BF _01, U_** | | **error %** | |
| T1-T0 |  | T2-T0 |  | 1.000 |  | 5.748 |  | 5.748 |  | 1.346e -5 |  |
|  | | | | | | | | | | | |
| Note.  The posterior odds have been corrected for multiple testing by fixing to 0.5 the prior probability that the null hypothesis holds across all comparisons (Westfall, Johnson, & Utts, 1997). Individual comparisons are based on the default t-test with a Cauchy (0, r = 1/sqrt(2)) prior. The "U" in the Bayes factor denotes that it is uncorrected. | | | | | | | | | | | |


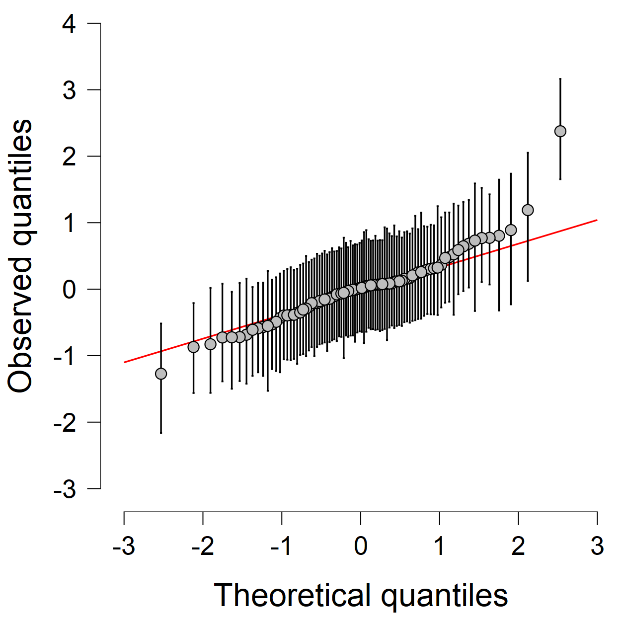


**Cerebral responses**

#### **Laser evoked ERPs : Δ(OFF-ON) P2 Latency**

| **Model Comparison** | | | | | | | | | | | |
| --- | --- | --- | --- | --- | --- | --- | --- | --- | --- | --- | --- |
| **Models** | | **P(M)** | | **P(M\|data)** | | **BF _M_** | | **BF _01_** | | **error %** | |
| Null model (incl. subject) |  | 0.200 |  | 0.436 |  | 3.095 |  | 1.000 |  |  |  |
| Volunteers |  | 0.200 |  | 0.211 |  | 1.069 |  | 2.068 |  | 0.657 |  |
| Condition + Volunteers + Condition  ✻  Volunteers |  | 0.200 |  | 0.145 |  | 0.679 |  | 3.006 |  | 1.973 |  |
| Condition |  | 0.200 |  | 0.137 |  | 0.635 |  | 3.185 |  | 1.208 |  |
| Condition + Volunteers |  | 0.200 |  | 0.071 |  | 0.305 |  | 6.159 |  | 7.189 |  |
|  | | | | | | | | | | | |
| Note.  All models include subject | | | | | | | | | | | |

| **Post Hoc Comparisons - Condition** | | | | | | | | | | | |
| --- | --- | --- | --- | --- | --- | --- | --- | --- | --- | --- | --- |
|  | |  | | **Prior Odds** | | **Posterior Odds** | | **BF _01, U_** | | **error %** | |
| taVNS |  | Sham |  | 1.000 |  | 4.171 |  | 4.171 |  | 0.031 |  |
|  | | | | | | | | | | | |
| Note.  The posterior odds have been corrected for multiple testing by fixing to 0.5 the prior probability that the null hypothesis holds across all comparisons (Westfall, Johnson, & Utts, 1997). Individual comparisons are based on the default t-test with a Cauchy (0, r = 1/sqrt(2)) prior. The "U" in the Bayes factor denotes that it is uncorrected. | | | | | | | | | | | |

###
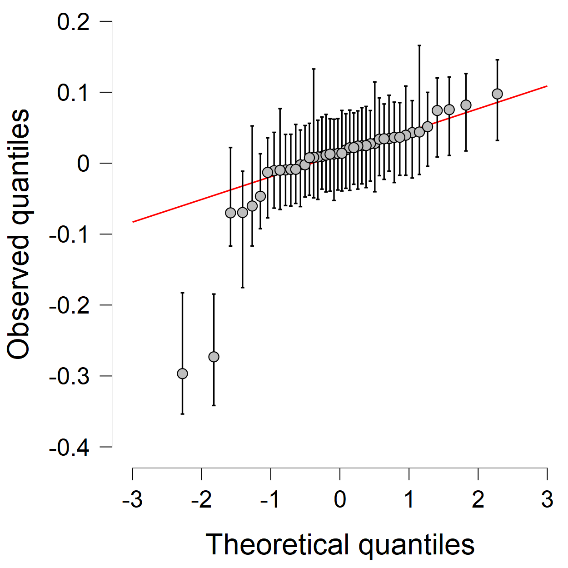
Model Averaged Q-Q Plot

#### **Laser evoked ERPs : Δ(OFF-ON) P2 amplitude:**

| **Model Comparison** | | | | | | | | | | | |
| --- | --- | --- | --- | --- | --- | --- | --- | --- | --- | --- | --- |
| **Models** | | **P(M)** | | **P(M\|data)** | | **BF _M_** | | **BF _01_** | | **error %** | |
| Null model (incl. subject) |  | 0.200 |  | 0.445 |  | 3.209 |  | 1.000 |  |  |  |
| Volunteers |  | 0.200 |  | 0.220 |  | 1.126 |  | 2.027 |  | 0.587 |  |
| Condition |  | 0.200 |  | 0.146 |  | 0.685 |  | 3.043 |  | 1.357 |  |
| Condition + Volunteers + Condition  ✻  Volunteers |  | 0.200 |  | 0.112 |  | 0.502 |  | **3.992** |  | 7.496 |  |
| Condition + Volunteers |  | 0.200 |  | 0.077 |  | 0.336 |  | **5.747** |  | 5.666 |  |
|  | | | | | | | | | | | |
| Note.  All models include subject | | | | | | | | | | | |

| **Post Hoc Comparisons - Condition** | | | | | | | | | | | |
| --- | --- | --- | --- | --- | --- | --- | --- | --- | --- | --- | --- |
|  | |  | | **Prior Odds** | | **Posterior Odds** | | **BF _01, U_** | | **error %** | |
| taVNS |  | Sham |  | 1.000 |  | 4.034 |  | 4.034 |  | 0.032 |  |
|  | | | | | | | | | | | |
| Note.  The posterior odds have been corrected for multiple testing by fixing to 0.5 the prior probability that the null hypothesis holds across all comparisons (Westfall, Johnson, & Utts, 1997). Individual comparisons are based on the default t-test with a Cauchy (0, r = 1/sqrt(2)) prior. The "U" in the Bayes factor denotes that it is uncorrected. | | | | | | | | | | | |

### Model Averaged Q-Q Plot


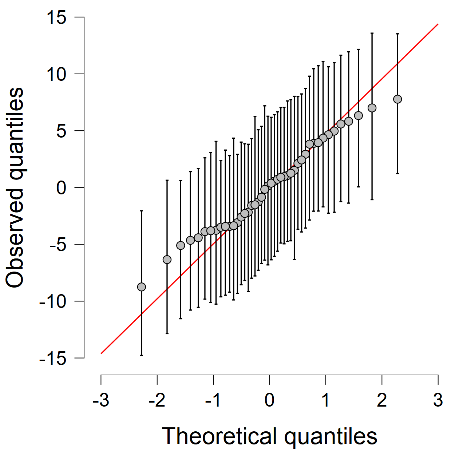


#### **Laser evoked ERPs : Δ(OFF-ON) N2 latency:**

| **Model Comparison** | | | | | | | | | | | |
| --- | --- | --- | --- | --- | --- | --- | --- | --- | --- | --- | --- |
| **Models** | | **P(M)** | | **P(M\|data)** | | **BF _M_** | | **BF _01_** | | **error %** | |
| Null model (incl. subject) |  | 0.200 |  | 0.403 |  | 2.705 |  | 1.000 |  |  |  |
| Condition + Volunteers + Condition  ✻  Volunteers |  | 0.200 |  | 0.298 |  | 1.697 |  | 1.355 |  | 13.501 |  |
| Condition |  | 0.200 |  | 0.158 |  | 0.750 |  | 2.555 |  | 2.107 |  |
| Volunteers |  | 0.200 |  | 0.100 |  | 0.447 |  | 4.016 |  | 0.166 |  |
| Condition + Volunteers |  | 0.200 |  | 0.040 |  | 0.168 |  | 9.998 |  | 1.988 |  |
|  | | | | | | | | | | | |
| Note.  All models include subject | | | | | | | | | | | |

| **Post Hoc Comparisons - Condition** | | | | | | | | | | | |
| --- | --- | --- | --- | --- | --- | --- | --- | --- | --- | --- | --- |
|  | |  | | **Prior Odds** | | **Posterior Odds** | | **BF _01, U_** | | **error %** | |
| taVNS |  | Sham |  | 1.000 |  | 3.551 |  | 3.551 |  | 0.032 |  |
|  | | | | | | | | | | | |
| Note.  The posterior odds have been corrected for multiple testing by fixing to 0.5 the prior probability that the null hypothesis holds across all comparisons (Westfall, Johnson, & Utts, 1997). Individual comparisons are based on the default t-test with a Cauchy (0, r = 1/sqrt(2)) prior. The "U" in the Bayes factor denotes that it is uncorrected. | | | | | | | | | | | |

### Model Averaged Q-Q Plot


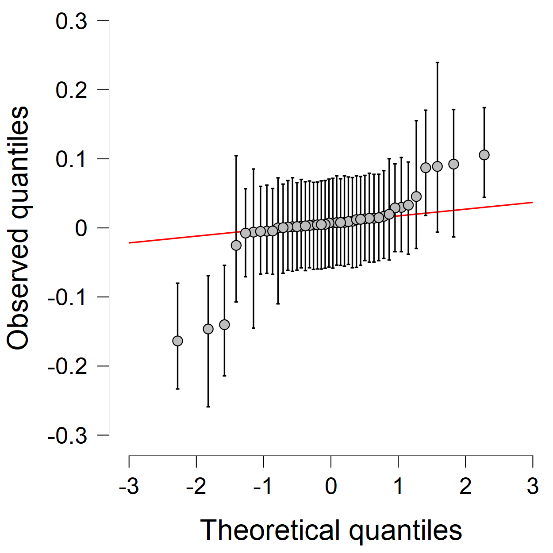


#### **Laser evoked ERPs : Δ(OFF-ON) N2 amplitude:**

| **Model Comparison** | | | | | | | | | | | |
| --- | --- | --- | --- | --- | --- | --- | --- | --- | --- | --- | --- |
| **Models** | | **P(M)** | | **P(M\|data)** | | **BF _M_** | | **BF _01_** | | **error %** | |
| Null model (incl. subject) |  | 0.200 |  | 0.446 |  | 3.221 |  | 1.000 |  |  |  |
| Condition + Volunteers + Condition  ✻  Volunteers |  | 0.200 |  | 0.226 |  | 1.171 |  | 1.970 |  | 3.624 |  |
| Volunteers |  | 0.200 |  | 0.153 |  | 0.720 |  | 2.924 |  | 0.245 |  |
| Condition |  | 0.200 |  | 0.131 |  | 0.604 |  | 3.399 |  | 1.032 |  |
| Condition + Volunteers |  | 0.200 |  | 0.044 |  | 0.183 |  | 10.183 |  | 0.829 |  |
|  | | | | | | | | | | | |
| Note.  All models include subject | | | | | | | | | | | |

| **Post Hoc Comparisons - Condition** | | | | | | | | | | | |
| --- | --- | --- | --- | --- | --- | --- | --- | --- | --- | --- | --- |
|  | |  | | **Prior Odds** | | **Posterior Odds** | | **BF _01, U_** | | **error %** | |
| taVNS |  | Sham |  | 1.000 |  | 4.483 |  | 4.483 |  | 0.031 |  |
|  | | | | | | | | | | | |
| Note.  The posterior odds have been corrected for multiple testing by fixing to 0.5 the prior probability that the null hypothesis holds across all comparisons (Westfall, Johnson, & Utts, 1997). Individual comparisons are based on the default t-test with a Cauchy (0, r = 1/sqrt(2)) prior. The "U" in the Bayes factor denotes that it is uncorrected. | | | | | | | | | | | |

### Model Averaged Q-Q Plot


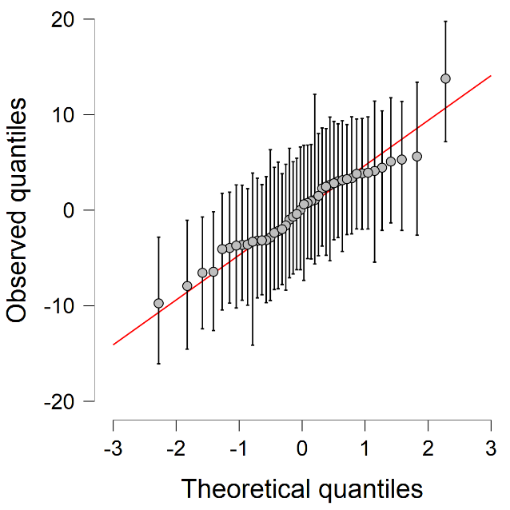


#### **Laser evoked ERPs : Δ(OFF-ON) N2P2 amplitude:**

| **Model Comparison** | | | | | | | | | | | |
| --- | --- | --- | --- | --- | --- | --- | --- | --- | --- | --- | --- |
| **Models** | | **P(M)** | | **P(M\|data)** | | **BF _M_** | | **BF _01_** | | **error %** | |
| Null model (incl. subject) |  | 0.200 |  | 0.428 |  | 2.995 |  | 1.000 |  |  |  |
| Volunteers |  | 0.200 |  | 0.217 |  | 1.109 |  | 1.973 |  | 0.705 |  |
| Condition + Volunteers + Condition  ✻  Volunteers |  | 0.200 |  | 0.159 |  | 0.757 |  | 2.692 |  | 33.979 |  |
| Condition |  | 0.200 |  | 0.130 |  | 0.600 |  | 3.283 |  | 1.551 |  |
| Condition + Volunteers |  | 0.200 |  | 0.065 |  | 0.279 |  | 6.564 |  | 1.192 |  |
|  | | | | | | | | | | | |
| Note.  All models include subject | | | | | | | | | | | |

| **Post Hoc Comparisons - Condition** | | | | | | | | | | | |
| --- | --- | --- | --- | --- | --- | --- | --- | --- | --- | --- | --- |
|  | |  | | **Prior Odds** | | **Posterior Odds** | | **BF _01, U_** | | **error %** | |
| taVNS |  | Sham |  | 1.000 |  | 4.283 |  | 4.283 |  | 0.031 |  |
|  | | | | | | | | | | | |
| Note.  The posterior odds have been corrected for multiple testing by fixing to 0.5 the prior probability that the null hypothesis holds across all comparisons (Westfall, Johnson, & Utts, 1997). Individual comparisons are based on the default t-test with a Cauchy (0, r = 1/sqrt(2)) prior. The "U" in the Bayes factor denotes that it is uncorrected. | | | | | | | | | | | |

###
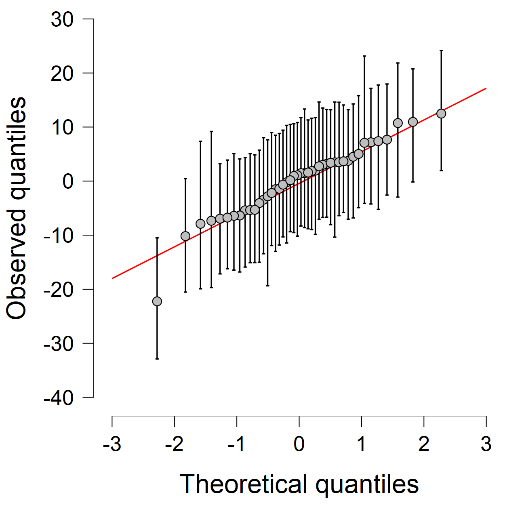
Model Averaged Q-Q Plot

###

#### **Vibrotactile-evoked ERPs : Δ(OFF-ON) P2 latency:**

| **Model Comparison** | | | | | | | | | | | |
| --- | --- | --- | --- | --- | --- | --- | --- | --- | --- | --- | --- |
| **Models** | | **P(M)** | | **P(M\|data)** | | **BF _M_** | | **BF _01_** | | **error %** | |
| Null model (incl. subject) |  | 0.200 |  | 0.349 |  | 2.147 |  | 1.000 |  |  |  |
| Volunteers |  | 0.200 |  | 0.257 |  | 1.384 |  | 1.358 |  | 2.779 |  |
| Condition |  | 0.200 |  | 0.153 |  | 0.723 |  | 2.280 |  | 10.049 |  |
| Condition + Volunteers + Condition  ✻  Volunteers |  | 0.200 |  | 0.127 |  | 0.581 |  | 2.755 |  | 1.655 |  |
| Condition + Volunteers |  | 0.200 |  | 0.114 |  | 0.513 |  | 3.072 |  | 5.695 |  |
|  | | | | | | | | | | | |
| Note.  All models include subject | | | | | | | | | | | |

| **Post Hoc Comparisons - Condition** | | | | | | | | | | | |
| --- | --- | --- | --- | --- | --- | --- | --- | --- | --- | --- | --- |
|  | |  | | **Prior Odds** | | **Posterior Odds** | | **BF _01, U_** | | **error %** | |
| taVNS |  | Sham |  | 1.000 |  | 3.156 |  | 3.156 |  | 0.031 |  |
|  | | | | | | | | | | | |
| Note.  The posterior odds have been corrected for multiple testing by fixing to 0.5 the prior probability that the null hypothesis holds across all comparisons (Westfall, Johnson, & Utts, 1997). Individual comparisons are based on the default t-test with a Cauchy (0, r = 1/sqrt(2)) prior. The "U" in the Bayes factor denotes that it is uncorrected. | | | | | | | | | | | |

### Model Averaged Q-Q Plot


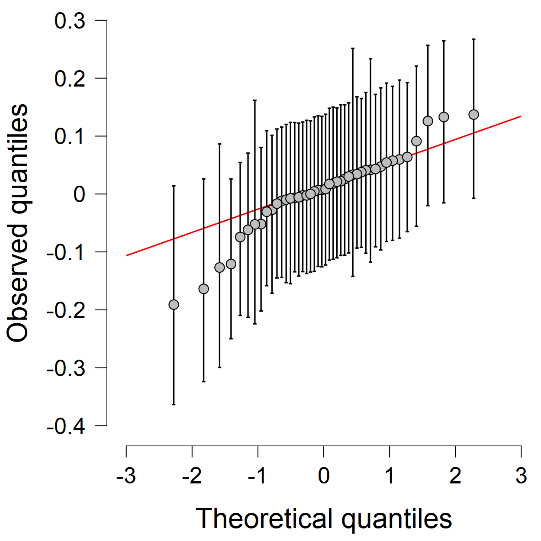


#### **Vibrotactile-evoked ERPs : Δ(OFF-ON) P2 amplitude:**

| **Model Comparison** | | | | | | | | | | | |
| --- | --- | --- | --- | --- | --- | --- | --- | --- | --- | --- | --- |
| **Models** | | **P(M)** | | **P(M\|data)** | | **BF _M_** | | **BF _01_** | | **error %** | |
| Null model (incl. subject) |  | 0.200 |  | 0.259 |  | 1.400 |  | 1.000 |  |  |  |
| Volunteers |  | 0.200 |  | 0.221 |  | 1.138 |  | 1.171 |  | 1.238 |  |
| Condition |  | 0.200 |  | 0.189 |  | 0.935 |  | 1.369 |  | 2.093 |  |
| Condition + Volunteers |  | 0.200 |  | 0.172 |  | 0.831 |  | 1.508 |  | 1.447 |  |
| Condition + Volunteers + Condition  ✻  Volunteers |  | 0.200 |  | 0.158 |  | 0.749 |  | 1.643 |  | 1.580 |  |
|  | | | | | | | | | | | |
| Note.  All models include subject | | | | | | | | | | | |

| **Post Hoc Comparisons - Condition** | | | | | | | | | | | |
| --- | --- | --- | --- | --- | --- | --- | --- | --- | --- | --- | --- |
|  | |  | | **Prior Odds** | | **Posterior Odds** | | **BF _01, U_** | | **error %** | |
| taVNS |  | Sham |  | 1.000 |  | 1.621 |  | 1.621 |  | 0.016 |  |
|  | | | | | | | | | | | |
| Note.  The posterior odds have been corrected for multiple testing by fixing to 0.5 the prior probability that the null hypothesis holds across all comparisons (Westfall, Johnson, & Utts, 1997). Individual comparisons are based on the default t-test with a Cauchy (0, r = 1/sqrt(2)) prior. The "U" in the Bayes factor denotes that it is uncorrected. | | | | | | | | | | | |

### Model Averaged Q-Q Plot


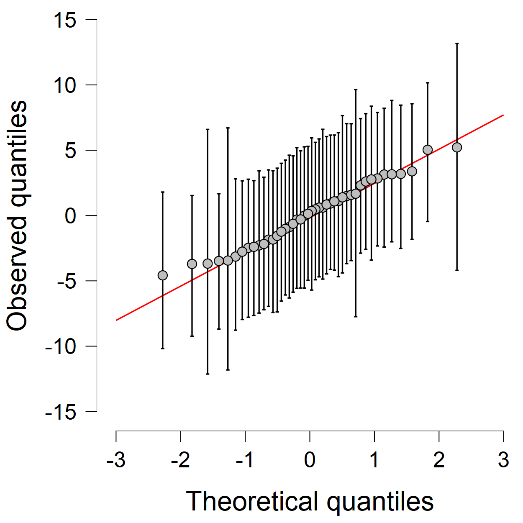


#### **Vibrotactile-evoked ERPs : Δ(OFF-ON) N2 latency:**

| **Model Comparison** | | | | | | | | | | | |
| --- | --- | --- | --- | --- | --- | --- | --- | --- | --- | --- | --- |
| **Models** | | **P(M)** | | **P(M\|data)** | | **BF _M_** | | **BF _01_** | | **error %** | |
| Null model (incl. subject) |  | 0.200 |  | 0.410 |  | 2.774 |  | 1.000 |  |  |  |
| Condition + Volunteers + Condition  ✻  Volunteers |  | 0.200 |  | 0.285 |  | 1.598 |  | 1.435 |  | 40.438 |  |
| Volunteers |  | 0.200 |  | 0.138 |  | 0.643 |  | 2.959 |  | 0.237 |  |
| Condition |  | 0.200 |  | 0.123 |  | 0.560 |  | 3.333 |  | 0.751 |  |
| Condition + Volunteers |  | 0.200 |  | 0.044 |  | 0.183 |  | 9.360 |  | 2.960 |  |
|  | | | | | | | | | | | |
| Note.  All models include subject | | | | | | | | | | | |

| **Post Hoc Comparisons - Condition** | | | | | | | | | | | |
| --- | --- | --- | --- | --- | --- | --- | --- | --- | --- | --- | --- |
|  | |  | | **Prior Odds** | | **Posterior Odds** | | **BF _01, U_** | | **error %** | |
| taVNS |  | Sham |  | 1.000 |  | 4.328 |  | 4.328 |  | 0.031 |  |
|  | | | | | | | | | | | |
| Note.  The posterior odds have been corrected for multiple testing by fixing to 0.5 the prior probability that the null hypothesis holds across all comparisons (Westfall, Johnson, & Utts, 1997). Individual comparisons are based on the default t-test with a Cauchy (0, r = 1/sqrt(2)) prior. The "U" in the Bayes factor denotes that it is uncorrected. | | | | | | | | | | | |

### Model Averaged Q-Q Plot


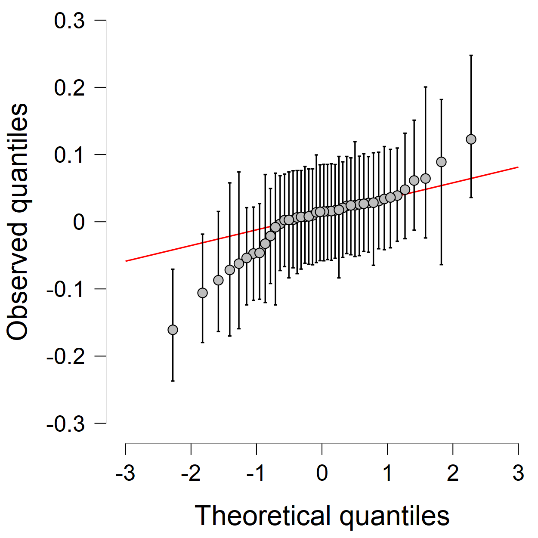


###

#### **Vibrotactile-evoked ERPs : Δ(OFF-ON) N2 amplitude:**

| **Model Comparison** | | | | | | | | | | | |
| --- | --- | --- | --- | --- | --- | --- | --- | --- | --- | --- | --- |
| **Models** | | **P(M)** | | **P(M\|data)** | | **BF _M_** | | **BF _01_** | | **error %** | |
| Null model (incl. subject) |  | 0.200 |  | 0.371 |  | 2.355 |  | 1.000 |  |  |  |
| Volunteers |  | 0.200 |  | 0.236 |  | 1.238 |  | 1.568 |  | 1.496 |  |
| Condition |  | 0.200 |  | 0.161 |  | 0.770 |  | 2.295 |  | 4.292 |  |
| Condition + Volunteers + Condition  ✻  Volunteers |  | 0.200 |  | 0.136 |  | 0.631 |  | 2.721 |  | 1.670 |  |
| Condition + Volunteers |  | 0.200 |  | 0.095 |  | 0.421 |  | 3.888 |  | 2.009 |  |
|  | | | | | | | | | | | |
| Note.  All models include subject | | | | | | | | | | | |

| **Post Hoc Comparisons - Condition** | | | | | | | | | | | |
| --- | --- | --- | --- | --- | --- | --- | --- | --- | --- | --- | --- |
|  | |  | | **Prior Odds** | | **Posterior Odds** | | **BF _01, U_** | | **error %** | |
| taVNS |  | Sham |  | 1.000 |  | 3.042 |  | 3.042 |  | 0.030 |  |
|  | | | | | | | | | | | |
| Note.  The posterior odds have been corrected for multiple testing by fixing to 0.5 the prior probability that the null hypothesis holds across all comparisons (Westfall, Johnson, & Utts, 1997). Individual comparisons are based on the default t-test with a Cauchy (0, r = 1/sqrt(2)) prior. The "U" in the Bayes factor denotes that it is uncorrected. | | | | | | | | | | | |

### Model Averaged Q-Q Plot


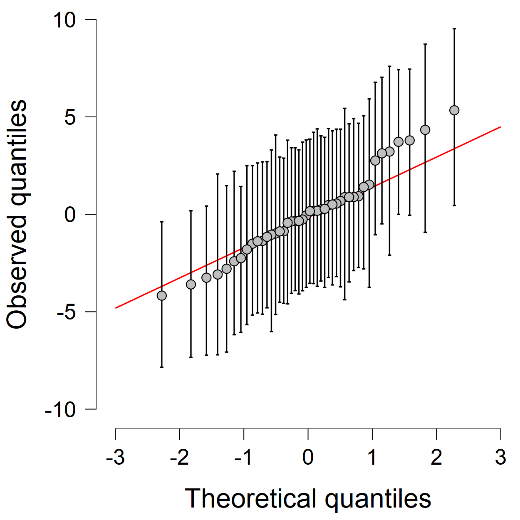


#### **Vibrotactile-evoked ERPs : Δ(OFF-ON) N2P2 amplitude:**

| **Model Comparison** | | | | | | | | | | | |
| --- | --- | --- | --- | --- | --- | --- | --- | --- | --- | --- | --- |
| **Models** | | **P(M)** | | **P(M\|data)** | | **BF _M_** | | **BF _01_** | | **error %** | |
| Null model (incl. subject) |  | 0.200 |  | 0.308 |  | 1.777 |  | 1.000 |  |  |  |
| Volunteers |  | 0.200 |  | 0.262 |  | 1.421 |  | 1.173 |  | 8.644 |  |
| Condition + Volunteers |  | 0.200 |  | 0.161 |  | 0.767 |  | 1.913 |  | 1.493 |  |
| Condition + Volunteers + Condition  ✻  Volunteers |  | 0.200 |  | 0.137 |  | 0.636 |  | 2.242 |  | 1.808 |  |
| Condition |  | 0.200 |  | 0.132 |  | 0.609 |  | 2.328 |  | 1.082 |  |
|  | | | | | | | | | | | |
| Note.  All models include subject | | | | | | | | | | | |

| **Post Hoc Comparisons - Condition** | | | | | | | | | | | |
| --- | --- | --- | --- | --- | --- | --- | --- | --- | --- | --- | --- |
|  | |  | | **Prior Odds** | | **Posterior Odds** | | **BF _01, U_** | | **error %** | |
| taVNS |  | Sham |  | 1.000 |  | 2.885 |  | 2.885 |  | 0.030 |  |
|  | | | | | | | | | | | |
| Note.  The posterior odds have been corrected for multiple testing by fixing to 0.5 the prior probability that the null hypothesis holds across all comparisons (Westfall, Johnson, & Utts, 1997). Individual comparisons are based on the default t-test with a Cauchy (0, r = 1/sqrt(2)) prior. The "U" in the Bayes factor denotes that it is uncorrected. | | | | | | | | | | | |

### Model Averaged Q-Q Plot


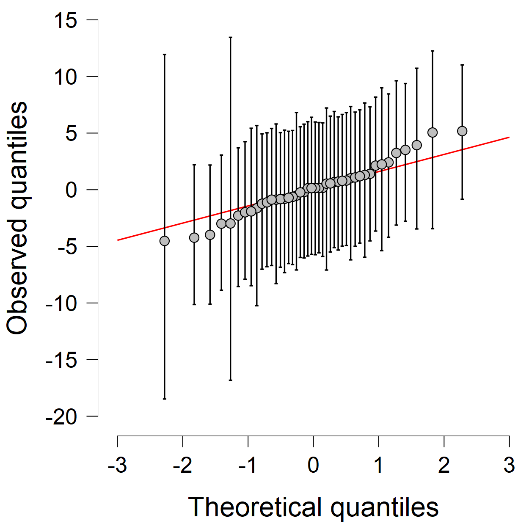


#### **Cool-evoked ERPs : Δ(OFF-ON) P2 latency:**

| **Model Comparison** | | | | | | | | | | | |
| --- | --- | --- | --- | --- | --- | --- | --- | --- | --- | --- | --- |
| **Models** | | **P(M)** | | **P(M\|data)** | | **BF _M_** | | **BF _01_** | | **error %** | |
| Null model (incl. subject) |  | 0.200 |  | 0.062 |  | 0.266 |  | 1.000 |  |  |  |
| Condition + Volunteers + Condition  ✻  Volunteers |  | 0.200 |  | 0.905 |  | 38.063 |  | 0.069 |  | 61.590 |  |
| Condition |  | 0.200 |  | 0.020 |  | 0.082 |  | 3.118 |  | 4.561 |  |
| Volunteers |  | 0.200 |  | 0.010 |  | 0.040 |  | 6.294 |  | 0.202 |  |
| Condition + Volunteers |  | 0.200 |  | 0.003 |  | 0.012 |  | 20.993 |  | 1.697 |  |
|  | | | | | | | | | | | |
| Note.  All models include subject | | | | | | | | | | | |

| **Post Hoc Comparisons - Condition** | | | | | | | | | | | |
| --- | --- | --- | --- | --- | --- | --- | --- | --- | --- | --- | --- |
|  | |  | | **Prior Odds** | | **Posterior Odds** | | **BF _01, U_** | | **error %** | |
| taVNS |  | Sham |  | 1.000 |  | 4.392 |  | 4.392 |  | 0.031 |  |
|  | | | | | | | | | | | |
| Note.  The posterior odds have been corrected for multiple testing by fixing to 0.5 the prior probability that the null hypothesis holds across all comparisons (Westfall, Johnson, & Utts, 1997). Individual comparisons are based on the default t-test with a Cauchy (0, r = 1/sqrt(2)) prior. The "U" in the Bayes factor denotes that it is uncorrected. | | | | | | | | | | | |

### Model Averaged Q-Q Plot


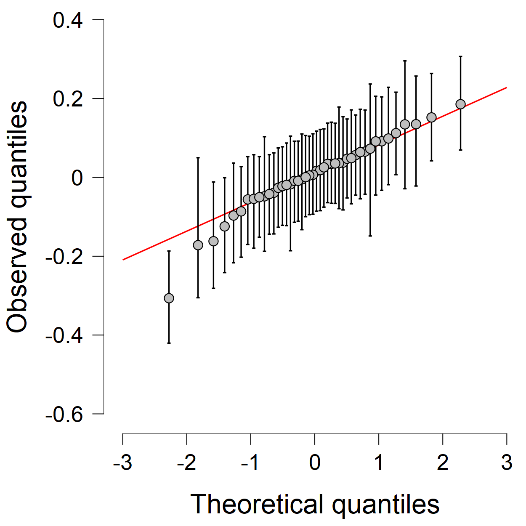


#### **Cool-evoked ERPs : Δ(OFF-ON) P2 amplitude:**

| **Model Comparison** | | | | | | | | | | | |
| --- | --- | --- | --- | --- | --- | --- | --- | --- | --- | --- | --- |
| **Models** | | **P(M)** | | **P(M\|data)** | | **BF _M_** | | **BF _01_** | | **error %** | |
| Null model (incl. subject) |  | 0.200 |  | 0.374 |  | 2.388 |  | 1.000 |  |  |  |
| Condition + Volunteers + Condition  ✻  Volunteers |  | 0.200 |  | 0.396 |  | 2.620 |  | 0.945 |  | 8.225 |  |
| Condition |  | 0.200 |  | 0.123 |  | 0.564 |  | 3.027 |  | 1.218 |  |
| Volunteers |  | 0.200 |  | 0.080 |  | 0.348 |  | 4.677 |  | 0.167 |  |
| Condition + Volunteers |  | 0.200 |  | 0.027 |  | 0.111 |  | 13.852 |  | 1.342 |  |
|  | | | | | | | | | | | |
| Note.  All models include subject | | | | | | | | | | | |

| **Post Hoc Comparisons - Condition** | | | | | | | | | | | |
| --- | --- | --- | --- | --- | --- | --- | --- | --- | --- | --- | --- |
|  | |  | | **Prior Odds** | | **Posterior Odds** | | **BF _01, U_** | | **error %** | |
| taVNS |  | Sham |  | 1.000 |  | 4.052 |  | 4.052 |  | 0.032 |  |
|  | | | | | | | | | | | |
| Note.  The posterior odds have been corrected for multiple testing by fixing to 0.5 the prior probability that the null hypothesis holds across all comparisons (Westfall, Johnson, & Utts, 1997). Individual comparisons are based on the default t-test with a Cauchy (0, r = 1/sqrt(2)) prior. The "U" in the Bayes factor denotes that it is uncorrected. | | | | | | | | | | | |

### Model Averaged Q-Q Plot


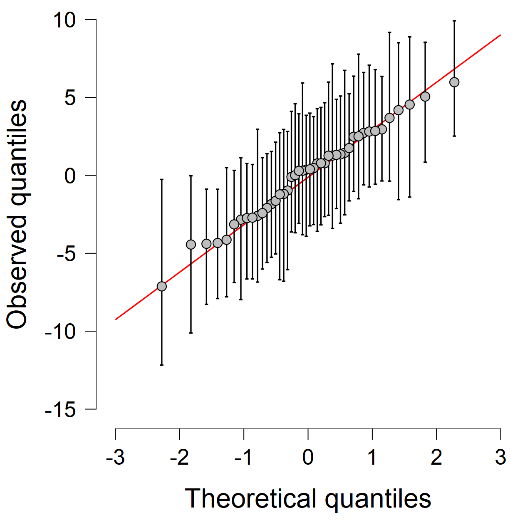


#### **Cool-evoked ERPs : Δ(OFF-ON) N2 latency:**

| **Model Comparison** | | | | | | | | | | | |
| --- | --- | --- | --- | --- | --- | --- | --- | --- | --- | --- | --- |
| **Models** | | **P(M)** | | **P(M\|data)** | | **BF _M_** | | **BF _01_** | | **error %** | |
| Null model (incl. subject) |  | 0.200 |  | 0.071 |  | 0.306 |  | 1.000 |  |  |  |
| Condition |  | 0.200 |  | 0.364 |  | 2.288 |  | 0.196 |  | 2.802 |  |
| Condition + Volunteers |  | 0.200 |  | 0.285 |  | 1.596 |  | 0.249 |  | 7.914 |  |
| Condition + Volunteers + Condition  ✻  Volunteers |  | 0.200 |  | 0.235 |  | 1.227 |  | 0.303 |  | 1.589 |  |
| Volunteers |  | 0.200 |  | 0.045 |  | 0.188 |  | 1.583 |  | 1.970 |  |
|  | | | | | | | | | | | |
| Note.  All models include subject | | | | | | | | | | | |

| **Post Hoc Comparisons - Condition** | | | | | | | | | | | |
| --- | --- | --- | --- | --- | --- | --- | --- | --- | --- | --- | --- |
|  | |  | | **Prior Odds** | | **Posterior Odds** | | **BF _01, U_** | | **error %** | |
| taVNS |  | Sham |  | 1.000 |  | 0.206 |  | 0.206 |  | 0.001 |  |
|  | | | | | | | | | | | |
| Note.  The posterior odds have been corrected for multiple testing by fixing to 0.5 the prior probability that the null hypothesis holds across all comparisons (Westfall, Johnson, & Utts, 1997). Individual comparisons are based on the default t-test with a Cauchy (0, r = 1/sqrt(2)) prior. The "U" in the Bayes factor denotes that it is uncorrected. | | | | | | | | | | | |

### Model Averaged Q-Q Plot


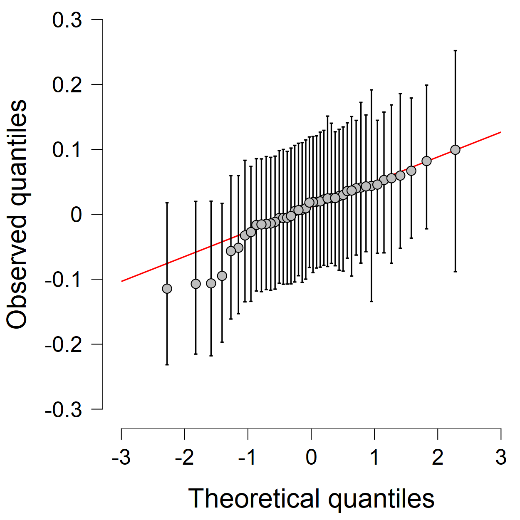


#### **Cool-evoked ERPs : Δ(OFF-ON) N2 amplitude:**

| **Model Comparison** | | | | | | | | | | | |
| --- | --- | --- | --- | --- | --- | --- | --- | --- | --- | --- | --- |
| **Models** | | **P(M)** | | **P(M\|data)** | | **BF _M_** | | **BF _01_** | | **error %** | |
| Null model (incl. subject) |  | 0.200 |  | 0.457 |  | 3.368 |  | 1.000 |  |  |  |
| Condition + Volunteers + Condition  ✻  Volunteers |  | 0.200 |  | 0.171 |  | 0.826 |  | 2.671 |  | 6.357 |  |
| Condition |  | 0.200 |  | 0.162 |  | 0.775 |  | 2.817 |  | 0.790 |  |
| Volunteers |  | 0.200 |  | 0.154 |  | 0.729 |  | 2.964 |  | 0.247 |  |
| Condition + Volunteers |  | 0.200 |  | 0.055 |  | 0.234 |  | 8.262 |  | 0.875 |  |
|  | | | | | | | | | | | |
| Note.  All models include subject | | | | | | | | | | | |

###

| **Post Hoc Comparisons - Condition** | | | | | | | | | | | |
| --- | --- | --- | --- | --- | --- | --- | --- | --- | --- | --- | --- |
|  | |  | | **Prior Odds** | | **Posterior Odds** | | **BF _01, U_** | | **error %** | |
| taVNS |  | Sham |  | 1.000 |  | 3.702 |  | 3.702 |  | 0.032 |  |
|  | | | | | | | | | | | |
| Note.  The posterior odds have been corrected for multiple testing by fixing to 0.5 the prior probability that the null hypothesis holds across all comparisons (Westfall, Johnson, & Utts, 1997). Individual comparisons are based on the default t-test with a Cauchy (0, r = 1/sqrt(2)) prior. The "U" in the Bayes factor denotes that it is uncorrected. | | | | | | | | | | | |

### Model Averaged Q-Q Plot


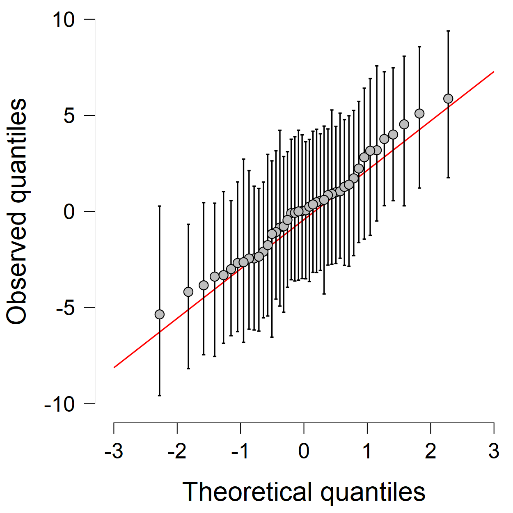


#### **Cool-evoked ERPs : Δ(OFF-ON) N2P2 amplitude:**

| **Model Comparison** | | | | | | | | | | | |
| --- | --- | --- | --- | --- | --- | --- | --- | --- | --- | --- | --- |
| **Models** | | **P(M)** | | **P(M\|data)** | | **BF _M_** | | **BF _01_** | | **error %** | |
| Null model (incl. subject) |  | 0.200 |  | 0.462 |  | 3.437 |  | 1.000 |  |  |  |
| Condition + Volunteers + Condition  ✻  Volunteers |  | 0.200 |  | 0.250 |  | 1.332 |  | 1.850 |  | 6.105 |  |
| Condition |  | 0.200 |  | 0.136 |  | 0.631 |  | 3.392 |  | 1.065 |  |
| Volunteers |  | 0.200 |  | 0.117 |  | 0.530 |  | 3.947 |  | 0.162 |  |
| Condition + Volunteers |  | 0.200 |  | 0.035 |  | 0.144 |  | 13.297 |  | 2.612 |  |
|  | | | | | | | | | | | |
| Note.  All models include subject | | | | | | | | | | | |

| **Post Hoc Comparisons - Condition** | | | | | | | | | | | |
| --- | --- | --- | --- | --- | --- | --- | --- | --- | --- | --- | --- |
|  | |  | | **Prior Odds** | | **Posterior Odds** | | **BF _01, U_** | | **error %** | |
| taVNS |  | Sham |  | 1.000 |  | 4.474 |  | 4.474 |  | 0.031 |  |
|  | | | | | | | | | | | |
| Note.  The posterior odds have been corrected for multiple testing by fixing to 0.5 the prior probability that the null hypothesis holds across all comparisons (Westfall, Johnson, & Utts, 1997). Individual comparisons are based on the default t-test with a Cauchy (0, r = 1/sqrt(2)) prior. The "U" in the Bayes factor denotes that it is uncorrected. | | | | | | | | | | | |

### Model Averaged Q-Q Plot


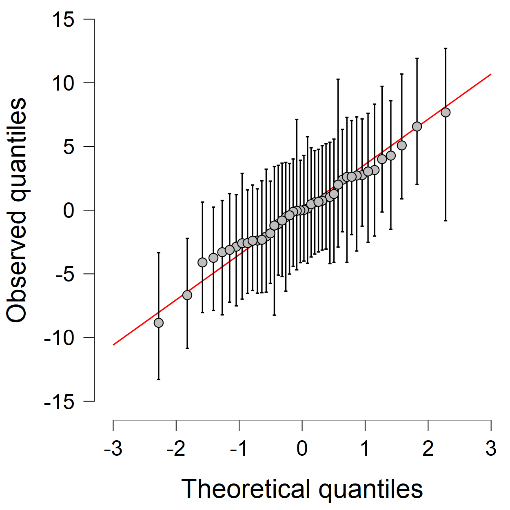


### Experiment 2

**Behavioral responses**

#### **Detection Thresholds (T1-T0)**

#### **Heat sensitive C-fibers**

| **Model Comparison** | | | | | | | | | | | |
| --- | --- | --- | --- | --- | --- | --- | --- | --- | --- | --- | --- |
| **Models** | | **P(M)** | | **P(M\|data)** | | **BF _M_** | | **BF _01_** | | **error %** | |
| Null model (incl. subject) |  | 0.500 |  | 0.746 |  | 2.933 |  | 1.000 |  |  |  |
| Condition |  | 0.500 |  | 0.254 |  | 0.341 |  | 2.933 |  | 0.992 |  |
|  | | | | | | | | | | | |
| Note.  All models include subject | | | | | | | | | | | |

| **Post Hoc Comparisons - Condition** | | | | | | | | | | | |
| --- | --- | --- | --- | --- | --- | --- | --- | --- | --- | --- | --- |
|  | |  | | **Prior Odds** | | **Posterior Odds** | | **BF _01, U_** | | **error %** | |
| taVNS |  | Sham |  | 1.000 |  | 3.779 |  | 3.779 |  | 0.003 |  |
|  | | | | | | | | | | | |
| Note.  The posterior odds have been corrected for multiple testing by fixing to 0.5 the prior probability that the null hypothesis holds across all comparisons (Westfall, Johnson, & Utts, 1997). Individual comparisons are based on the default t-test with a Cauchy (0, r = 1/sqrt(2)) prior. The "U" in the Bayes factor denotes that it is uncorrected. | | | | | | | | | | | |

### Model Averaged Q-Q Plot


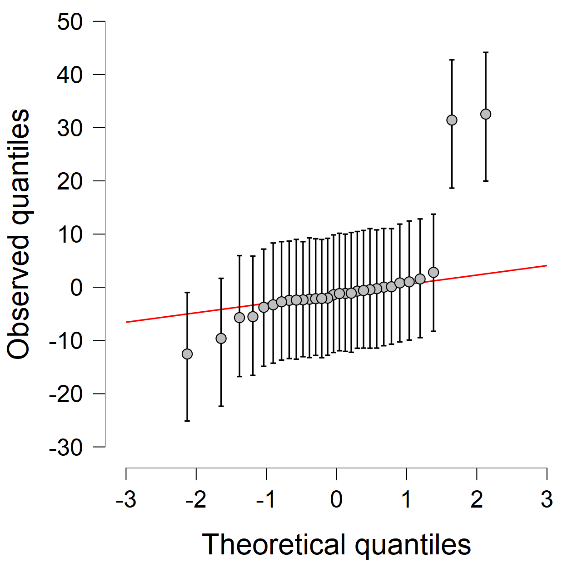


#### **Heat sensitive Aδ-fibers**

| **Model Comparison** | | | | | | | | | | | |
| --- | --- | --- | --- | --- | --- | --- | --- | --- | --- | --- | --- |
| **Models** | | **P(M)** | | **P(M\|data)** | | **BF _M_** | | **BF _01_** | | **error %** | |
| Null model (incl. subject) |  | 0.500 |  | 0.746 |  | 2.931 |  | 1.000 |  |  |  |
| Condition |  | 0.500 |  | 0.254 |  | 0.341 |  | 2.931 |  | 1.168 |  |
|  | | | | | | | | | | | |
| Note.  All models include subject | | | | | | | | | | | |

| **Post Hoc Comparisons - Condition** | | | | | | | | | | | |
| --- | --- | --- | --- | --- | --- | --- | --- | --- | --- | --- | --- |
|  | |  | | **Prior Odds** | | **Posterior Odds** | | **BF _01, U_** | | **error %** | |
| taVNS |  | Sham |  | 1.000 |  | 3.799 |  | 3.799 |  | 0.003 |  |
|  | | | | | | | | | | | |
| Note.  The posterior odds have been corrected for multiple testing by fixing to 0.5 the prior probability that the null hypothesis holds across all comparisons (Westfall, Johnson, & Utts, 1997). Individual comparisons are based on the default t-test with a Cauchy (0, r = 1/sqrt(2)) prior. The "U" in the Bayes factor denotes that it is uncorrected. | | | | | | | | | | | |

### Model Averaged Q-Q Plot


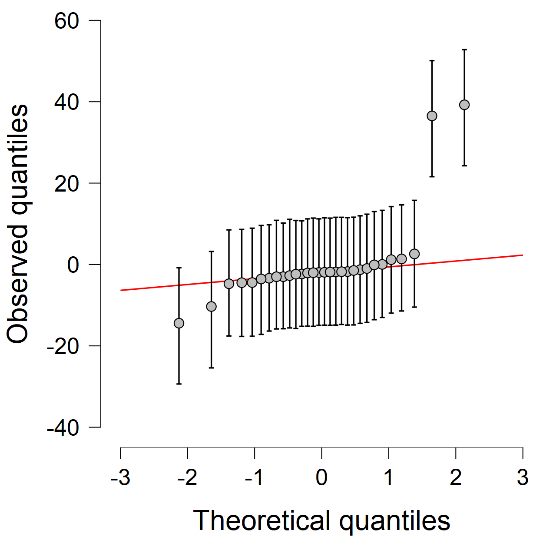


#### **Mechanosensitive Aβ-fibers**

| **Model Comparison** | | | | | | | | | | | |
| --- | --- | --- | --- | --- | --- | --- | --- | --- | --- | --- | --- |
| **Models** | | **P(M)** | | **P(M\|data)** | | **BF _M_** | | **BF _01_** | | **error %** | |
| Null model (incl. subject) |  | 0.500 |  | 0.550 |  | 1.224 |  | 1.000 |  |  |  |
| Condition |  | 0.500 |  | 0.450 |  | 0.817 |  | 1.224 |  | 1.006 |  |
|  | | | | | | | | | | | |
| Note.  All models include subject | | | | | | | | | | | |

### Model Averaged Q-Q Plot

| **Post Hoc Comparisons - Condition** | | | | | | | | | | | |
| --- | --- | --- | --- | --- | --- | --- | --- | --- | --- | --- | --- |
|  | |  | | **Prior Odds** | | **Posterior Odds** | | **BF _01, U_** | | **error %** | |
| taVNS |  | Sham |  | 1.000 |  | 1.591 |  | 1.591 |  | 0.012 |  |
|  | | | | | | | | | | | |
| Note.  The posterior odds have been corrected for multiple testing by fixing to 0.5 the prior probability that the null hypothesis holds across all comparisons (Westfall, Johnson, & Utts, 1997). Individual comparisons are based on the default t-test with a Cauchy (0, r = 1/sqrt(2)) prior. The "U" in the Bayes factor denotes that it is uncorrected. | | | | | | | | | | | |


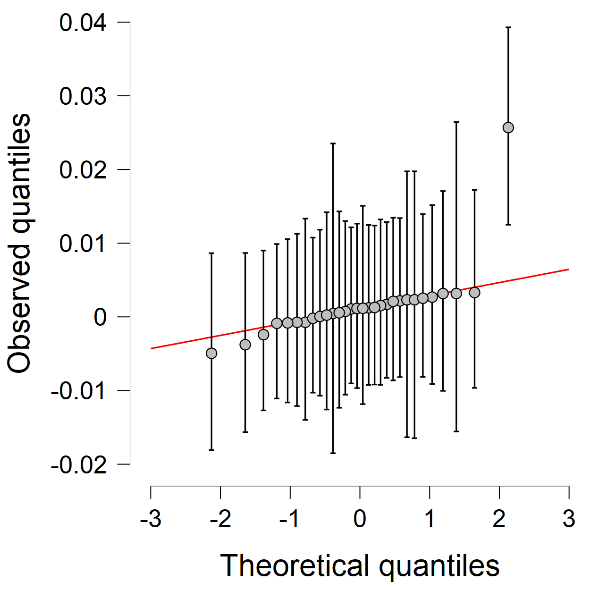


**Perception Intensity ( ΔOFF-ON)**

#### **Laser**

| **Model Comparison** | | | | | | | | | | | |
| --- | --- | --- | --- | --- | --- | --- | --- | --- | --- | --- | --- |
| **Models** | | **P(M)** | | **P(M\|data)** | | **BF _M_** | | **BF _01_** | | **error %** | |
| Null model (incl. subject) |  | 0.500 |  | 0.722 |  | 2.596 |  | 1.000 |  |  |  |
| Condition |  | 0.500 |  | 0.278 |  | 0.385 |  | 2.596 |  | 0.908 |  |
|  | | | | | | | | | | | |
| Note.  All models include subject | | | | | | | | | | | |

| **Post Hoc Comparisons - Condition** | | | | | | | | | | | |
| --- | --- | --- | --- | --- | --- | --- | --- | --- | --- | --- | --- |
|  | |  | | **Prior Odds** | | **Posterior Odds** | | **BF _01, U_** | | **error %** | |
| taVNS |  | Sham |  | 1.000 |  | 3.315 |  | 3.315 |  | 0.003 |  |
|  | | | | | | | | | | | |
| Note.  The posterior odds have been corrected for multiple testing by fixing to 0.5 the prior probability that the null hypothesis holds across all comparisons (Westfall, Johnson, & Utts, 1997). Individual comparisons are based on the default t-test with a Cauchy (0, r = 1/sqrt(2)) prior. The "U" in the Bayes factor denotes that it is uncorrected. | | | | | | | | | | | |

### Model Averaged Q-Q Plot


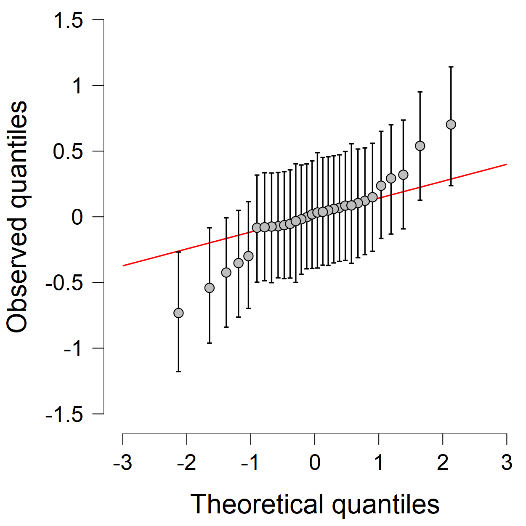


#### **Vibrotactile**

| **Model Comparison** | | | | | | | | | | | |
| --- | --- | --- | --- | --- | --- | --- | --- | --- | --- | --- | --- |
| **Models** | | **P(M)** | | **P(M\|data)** | | **BF _M_** | | **BF _01_** | | **error %** | |
| Null model (incl. subject) |  | 0.500 |  | 0.713 |  | 2.482 |  | 1.000 |  |  |  |
| Condition |  | 0.500 |  | 0.287 |  | 0.403 |  | 2.482 |  | 2.323 |  |
|  | | | | | | | | | | | |
| Note.  All models include subject | | | | | | | | | | | |

### Model Averaged Q-Q Plot

| **Post Hoc Comparisons - Condition** | | | | | | | | | | | |
| --- | --- | --- | --- | --- | --- | --- | --- | --- | --- | --- | --- |
|  | |  | | **Prior Odds** | | **Posterior Odds** | | **BF _01, U_** | | **error %** | |
| taVNS |  | Sham |  | 1.000 |  | 3.275 |  | 3.275 |  | 0.003 |  |
|  | | | | | | | | | | | |
| Note.  The posterior odds have been corrected for multiple testing by fixing to 0.5 the prior probability that the null hypothesis holds across all comparisons (Westfall, Johnson, & Utts, 1997). Individual comparisons are based on the default t-test with a Cauchy (0, r = 1/sqrt(2)) prior. The "U" in the Bayes factor denotes that it is uncorrected. | | | | | | | | | | | |


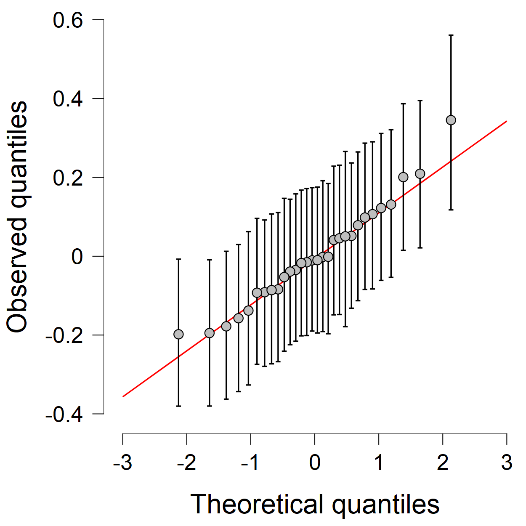


**Cerebral responses**

#### **Laser-evoked ERPs : Δ(OFF-ON) P2 latency:**

| **Model Comparison** | | | | | | | | | | | |
| --- | --- | --- | --- | --- | --- | --- | --- | --- | --- | --- | --- |
| **Models** | | **P(M)** | | **P(M\|data)** | | **BF _M_** | | **BF _01_** | | **error %** | |
| Null model (incl. subject) |  | 0.500 |  | 0.694 |  | 2.273 |  | 1.000 |  |  |  |
| Condition |  | 0.500 |  | 0.306 |  | 0.440 |  | 2.273 |  | 1.952 |  |
|  | | | | | | | | | | | |
| Note.  All models include subject | | | | | | | | | | | |

| **Post Hoc Comparisons - Condition** | | | | | | | | | | | |
| --- | --- | --- | --- | --- | --- | --- | --- | --- | --- | --- | --- |
|  | |  | | **Prior Odds** | | **Posterior Odds** | | **BF _01, U_** | | **error %** | |
| taVNS |  | Sham |  | 1.000 |  | 2.916 |  | 2.916 |  | 0.004 |  |
|  | | | | | | | | | | | |
| Note.  The posterior odds have been corrected for multiple testing by fixing to 0.5 the prior probability that the null hypothesis holds across all comparisons (Westfall, Johnson, & Utts, 1997). Individual comparisons are based on the default t-test with a Cauchy (0, r = 1/sqrt(2)) prior. The "U" in the Bayes factor denotes that it is uncorrected. | | | | | | | | | | | |

### Model Averaged Q-Q Plot


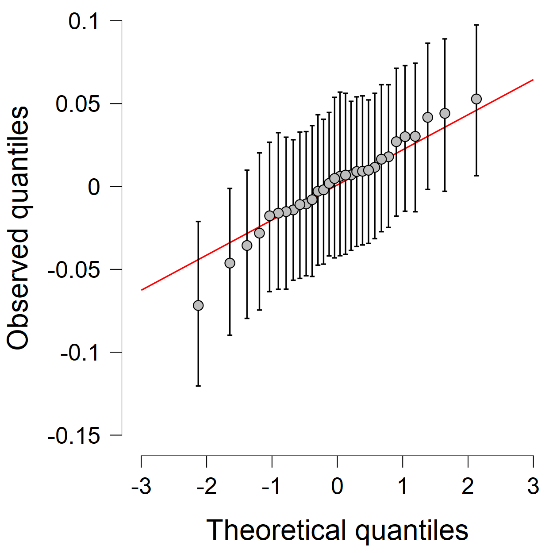


#### **Laser-evoked ERPs : Δ(OFF-ON) P2 amplitude:**

| **Model Comparison** | | | | | | | | | | | |
| --- | --- | --- | --- | --- | --- | --- | --- | --- | --- | --- | --- |
| **Models** | | **P(M)** | | **P(M\|data)** | | **BF _M_** | | **BF _01_** | | **error %** | |
| Null model (incl. subject) |  | 0.500 |  | 0.737 |  | 2.798 |  | 1.000 |  |  |  |
| Condition |  | 0.500 |  | 0.263 |  | 0.357 |  | 2.798 |  | 1.122 |  |
|  | | | | | | | | | | | |
| Note.  All models include subject | | | | | | | | | | | |

### Model Averaged Q-Q Plot

| **Post Hoc Comparisons - Condition** | | | | | | | | | | | |
| --- | --- | --- | --- | --- | --- | --- | --- | --- | --- | --- | --- |
|  | |  | | **Prior Odds** | | **Posterior Odds** | | **BF _01, U_** | | **error %** | |
| taVNS |  | Sham |  | 1.000 |  | 3.690 |  | 3.690 |  | 0.003 |  |
|  | | | | | | | | | | | |
| Note.  The posterior odds have been corrected for multiple testing by fixing to 0.5 the prior probability that the null hypothesis holds across all comparisons (Westfall, Johnson, & Utts, 1997). Individual comparisons are based on the default t-test with a Cauchy (0, r = 1/sqrt(2)) prior. The "U" in the Bayes factor denotes that it is uncorrected. | | | | | | | | | | | |


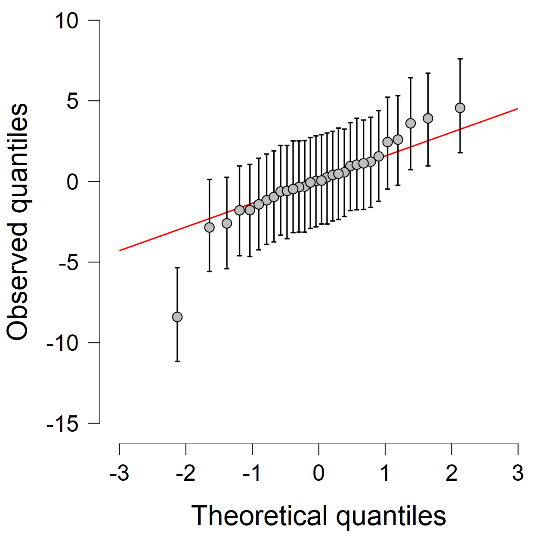


#### **Laser-evoked ERPs : Δ(OFF-ON) N2 latency:**

| **Model Comparison** | | | | | | | | | | | |
| --- | --- | --- | --- | --- | --- | --- | --- | --- | --- | --- | --- |
| **Models** | | **P(M)** | | **P(M\|data)** | | **BF _M_** | | **BF _01_** | | **error %** | |
| Null model (incl. subject) |  | 0.500 |  | 0.734 |  | 2.762 |  | 1.000 |  |  |  |
| Condition |  | 0.500 |  | 0.266 |  | 0.362 |  | 2.762 |  | 1.243 |  |
|  | | | | | | | | | | | |
| Note.  All models include subject | | | | | | | | | | | |

| **Post Hoc Comparisons - Condition** | | | | | | | | | | | |
| --- | --- | --- | --- | --- | --- | --- | --- | --- | --- | --- | --- |
|  | |  | | **Prior Odds** | | **Posterior Odds** | | **BF _01, U_** | | **error %** | |
| taVNS |  | Sham |  | 1.000 |  | 3.592 |  | 3.592 |  | 0.003 |  |
|  | | | | | | | | | | | |
| Note.  The posterior odds have been corrected for multiple testing by fixing to 0.5 the prior probability that the null hypothesis holds across all comparisons (Westfall, Johnson, & Utts, 1997). Individual comparisons are based on the default t-test with a Cauchy (0, r = 1/sqrt(2)) prior. The "U" in the Bayes factor denotes that it is uncorrected. | | | | | | | | | | | |

### Model Averaged Q-Q Plot


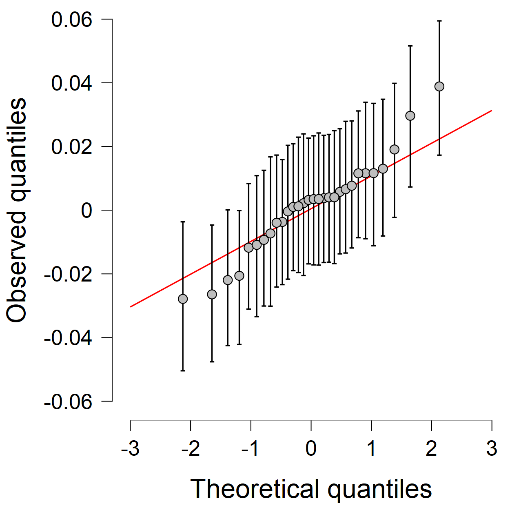


#### **Laser-evoked ERPs : Δ(OFF-ON) N2 amplitude:**

| **Model Comparison** | | | | | | | | | | | |
| --- | --- | --- | --- | --- | --- | --- | --- | --- | --- | --- | --- |
| **Models** | | **P(M)** | | **P(M\|data)** | | **BF _M_** | | **BF _01_** | | **error %** | |
| Null model (incl. subject) |  | 0.500 |  | 0.604 |  | 1.526 |  | 1.000 |  |  |  |
| Condition |  | 0.500 |  | 0.396 |  | 0.655 |  | 1.526 |  | 0.777 |  |
|  | | | | | | | | | | | |
| Note.  All models include subject | | | | | | | | | | | |

| **Post Hoc Comparisons - Condition** | | | | | | | | | | | |
| --- | --- | --- | --- | --- | --- | --- | --- | --- | --- | --- | --- |
|  | |  | | **Prior Odds** | | **Posterior Odds** | | **BF _01, U_** | | **error %** | |
| taVNS |  | Sham |  | 1.000 |  | 2.268 |  | 2.268 |  | 0.007 |  |
|  | | | | | | | | | | | |
| Note.  The posterior odds have been corrected for multiple testing by fixing to 0.5 the prior probability that the null hypothesis holds across all comparisons (Westfall, Johnson, & Utts, 1997). Individual comparisons are based on the default t-test with a Cauchy (0, r = 1/sqrt(2)) prior. The "U" in the Bayes factor denotes that it is uncorrected. | | | | | | | | | | | |

### Model Averaged Q-Q Plot


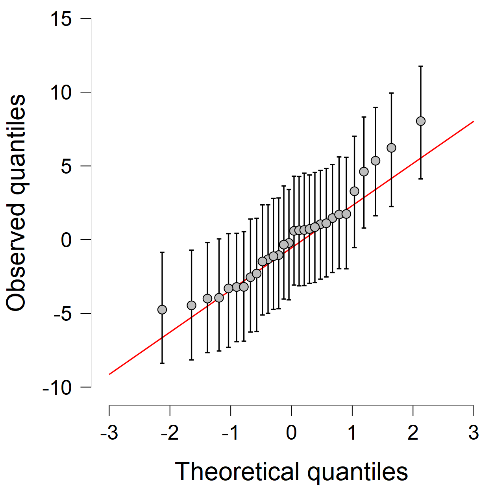


#### **Laser-evoked ERPs : Δ(OFF-ON) N2P2 amplitude:**

| **Model Comparison** | | | | | | | | | | | |
| --- | --- | --- | --- | --- | --- | --- | --- | --- | --- | --- | --- |
| **Models** | | **P(M)** | | **P(M\|data)** | | **BF _M_** | | **BF _01_** | | **error %** | |
| Null model (incl. subject) |  | 0.500 |  | 0.521 |  | 1.090 |  | 1.000 |  |  |  |
| Condition |  | 0.500 |  | 0.479 |  | 0.918 |  | 1.090 |  | 1.836 |  |
|  | | | | | | | | | | | |
| Note.  All models include subject | | | | | | | | | | | |

| **Post Hoc Comparisons - Condition** | | | | | | | | | | | |
| --- | --- | --- | --- | --- | --- | --- | --- | --- | --- | --- | --- |
|  | |  | | **Prior Odds** | | **Posterior Odds** | | **BF _01, U_** | | **error %** | |
| taVNS |  | Sham |  | 1.000 |  | 1.767 |  | 1.767 |  | 0.010 |  |
|  | | | | | | | | | | | |
| Note.  The posterior odds have been corrected for multiple testing by fixing to 0.5 the prior probability that the null hypothesis holds across all comparisons (Westfall, Johnson, & Utts, 1997). Individual comparisons are based on the default t-test with a Cauchy (0, r = 1/sqrt(2)) prior. The "U" in the Bayes factor denotes that it is uncorrected. | | | | | | | | | | | |

### Model Averaged Q-Q Plot


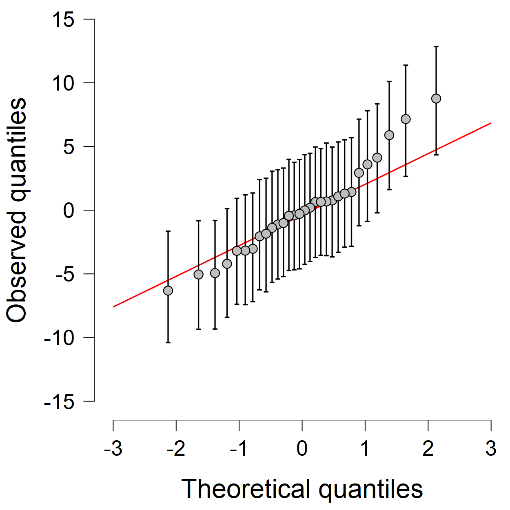


#### **Vibrotactile-evoked ERPs : Δ(OFF-ON) P2 latency:**

| **Model Comparison** | | | | | | | | | | | |
| --- | --- | --- | --- | --- | --- | --- | --- | --- | --- | --- | --- |
| **Models** | | **P(M)** | | **P(M\|data)** | | **BF _M_** | | **BF _01_** | | **error %** | |
| Condition |  | 0.500 |  | 0.618 |  | 1.618 |  | 1.000 |  |  |  |
| Null model (incl. subject) |  | 0.500 |  | 0.382 |  | 0.618 |  | 1.618 |  | 1.074 |  |
|  | | | | | | | | | | | |
| Note.  All models include subject | | | | | | | | | | | |

| **Post Hoc Comparisons - Condition** | | | | | | | | | | | |
| --- | --- | --- | --- | --- | --- | --- | --- | --- | --- | --- | --- |
|  | |  | | **Prior Odds** | | **Posterior Odds** | | **BF _01, U_** | | **error %** | |
| taVNS |  | Sham |  | 1.000 |  | 1.270 |  | 1.270 |  | 0.013 |  |
|  | | | | | | | | | | | |
| Note.  The posterior odds have been corrected for multiple testing by fixing to 0.5 the prior probability that the null hypothesis holds across all comparisons (Westfall, Johnson, & Utts, 1997). Individual comparisons are based on the default t-test with a Cauchy (0, r = 1/sqrt(2)) prior. The "U" in the Bayes factor denotes that it is uncorrected. | | | | | | | | | | | |

### Model Averaged Q-Q Plot


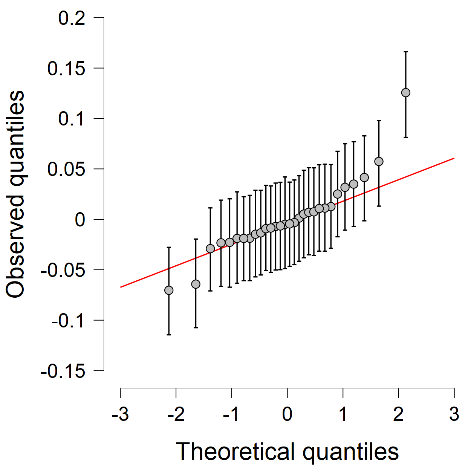


#### **Vibrotactile-evoked ERPs : Δ(OFF-ON) P2 amplitude:**

| **Model Comparison** | | | | | | | | | | | |
| --- | --- | --- | --- | --- | --- | --- | --- | --- | --- | --- | --- |
| **Models** | | **P(M)** | | **P(M\|data)** | | **BF _M_** | | **BF _01_** | | **error %** | |
| Null model (incl. subject) |  | 0.500 |  | 0.732 |  | 2.727 |  | 1.000 |  |  |  |
| Condition |  | 0.500 |  | 0.268 |  | 0.367 |  | 2.727 |  | 0.754 |  |
|  | | | | | | | | | | | |
| Note.  All models include subject | | | | | | | | | | | |

| **Post Hoc Comparisons - Condition** | | | | | | | | | | | |
| --- | --- | --- | --- | --- | --- | --- | --- | --- | --- | --- | --- |
|  | |  | | **Prior Odds** | | **Posterior Odds** | | **BF _01, U_** | | **error %** | |
| taVNS |  | Sham |  | 1.000 |  | 3.518 |  | 3.518 |  | 0.003 |  |
|  | | | | | | | | | | | |
| Note.  The posterior odds have been corrected for multiple testing by fixing to 0.5 the prior probability that the null hypothesis holds across all comparisons (Westfall, Johnson, & Utts, 1997). Individual comparisons are based on the default t-test with a Cauchy (0, r = 1/sqrt(2)) prior. The "U" in the Bayes factor denotes that it is uncorrected. | | | | | | | | | | | |

### Model Averaged Q-Q Plot


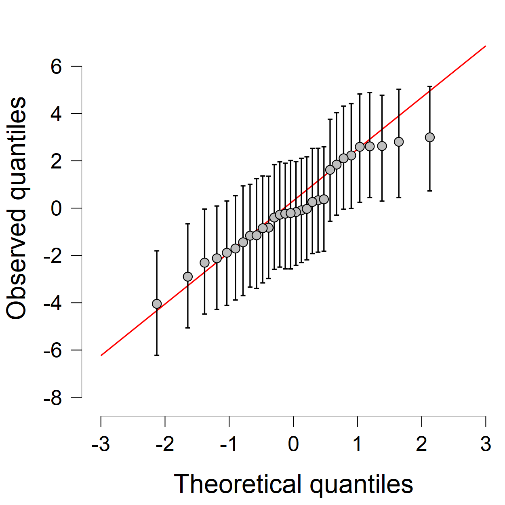


#### **Vibrotactile-evoked ERPs : Δ(OFF-ON) N2 latency:**

## Bayesian Repeated Measures ANOVA

| **Model Comparison** | | | | | | | | | | | |
| --- | --- | --- | --- | --- | --- | --- | --- | --- | --- | --- | --- |
| **Models** | | **P(M)** | | **P(M\|data)** | | **BF _M_** | | **BF _01_** | | **error %** | |
| Null model (incl. subject) |  | 0.500 |  | 0.661 |  | 1.948 |  | 1.000 |  |  |  |
| Condition |  | 0.500 |  | 0.339 |  | 0.513 |  | 1.948 |  | 1.010 |  |
|  | | | | | | | | | | | |
| Note.  All models include subject | | | | | | | | | | | |

| **Post Hoc Comparisons - Condition** | | | | | | | | | | | |
| --- | --- | --- | --- | --- | --- | --- | --- | --- | --- | --- | --- |
|  | |  | | **Prior Odds** | | **Posterior Odds** | | **BF _01, U_** | | **error %** | |
| taVNS |  | Sham |  | 1.000 |  | 2.625 |  | 2.625 |  | 0.005 |  |
|  | | | | | | | | | | | |
| Note.  The posterior odds have been corrected for multiple testing by fixing to 0.5 the prior probability that the null hypothesis holds across all comparisons (Westfall, Johnson, & Utts, 1997). Individual comparisons are based on the default t-test with a Cauchy (0, r = 1/sqrt(2)) prior. The "U" in the Bayes factor denotes that it is uncorrected. | | | | | | | | | | | |

### Model Averaged Q-Q Plot


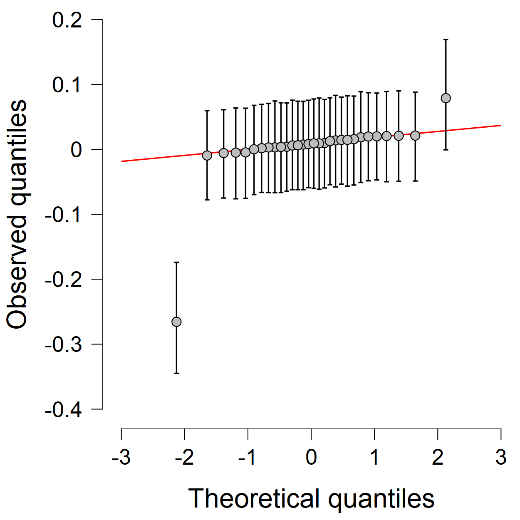


#### **Vibrotactile-evoked ERPs : Δ(OFF-ON) N2 amplitude:**

| **Model Comparison** | | | | | | | | | | | |
| --- | --- | --- | --- | --- | --- | --- | --- | --- | --- | --- | --- |
| **Models** | | **P(M)** | | **P(M\|data)** | | **BF _M_** | | **BF _01_** | | **error %** | |
| Null model (incl. subject) |  | 0.500 |  | 0.135 |  | 0.156 |  | 1.000 |  |  |  |
| Condition |  | 0.500 |  | 0.865 |  | 6.430 |  | 0.156 |  | 0.936 |  |
|  | | | | | | | | | | | |
| Note.  All models include subject | | | | | | | | | | | |

| **Post Hoc Comparisons - Condition** | | | | | | | | | | | |
| --- | --- | --- | --- | --- | --- | --- | --- | --- | --- | --- | --- |
|  | |  | | **Prior Odds** | | **Posterior Odds** | | **BF _01, U_** | | **error %** | |
| taVNS |  | Sham |  | 1.000 |  | 0.327 |  | 0.327 |  | 6.513e -5 |  |
|  | | | | | | | | | | | |
| Note.  The posterior odds have been corrected for multiple testing by fixing to 0.5 the prior probability that the null hypothesis holds across all comparisons (Westfall, Johnson, & Utts, 1997). Individual comparisons are based on the default t-test with a Cauchy (0, r = 1/sqrt(2)) prior. The "U" in the Bayes factor denotes that it is uncorrected. | | | | | | | | | | | |

### Model Averaged Q-Q Plot


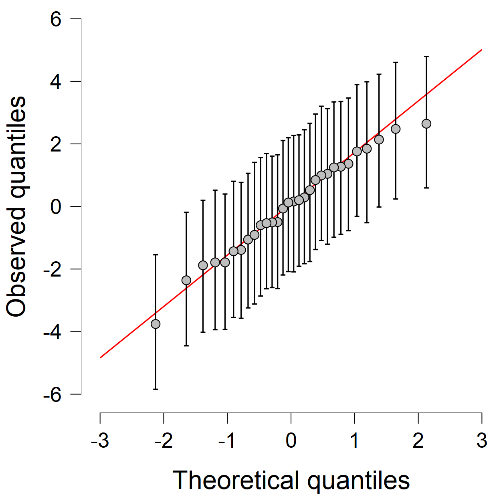


#### **Vibrotactile-evoked ERPs : Δ(OFF-ON) N2P2 amplitude:**

| **Model Comparison** | | | | | | | | | | | |
| --- | --- | --- | --- | --- | --- | --- | --- | --- | --- | --- | --- |
| **Models** | | **P(M)** | | **P(M\|data)** | | **BF _M_** | | **BF _01_** | | **error %** | |
| Null model (incl. subject) |  | 0.500 |  | 0.600 |  | 1.498 |  | 1.000 |  |  |  |
| Condition |  | 0.500 |  | 0.400 |  | 0.667 |  | 1.498 |  | 1.869 |  |
|  | | | | | | | | | | | |
| Note.  All models include subject | | | | | | | | | | | |

| **Post Hoc Comparisons - Condition** | | | | | | | | | | | |
| --- | --- | --- | --- | --- | --- | --- | --- | --- | --- | --- | --- |
|  | |  | | **Prior Odds** | | **Posterior Odds** | | **BF _01, U_** | | **error %** | |
| taVNS |  | Sham |  | 1.000 |  | 2.283 |  | 2.283 |  | 0.007 |  |
|  | | | | | | | | | | | |
| Note.  The posterior odds have been corrected for multiple testing by fixing to 0.5 the prior probability that the null hypothesis holds across all comparisons (Westfall, Johnson, & Utts, 1997). Individual comparisons are based on the default t-test with a Cauchy (0, r = 1/sqrt(2)) prior. The "U" in the Bayes factor denotes that it is uncorrected. | | | | | | | | | | | |

### Model Averaged Q-Q Plot


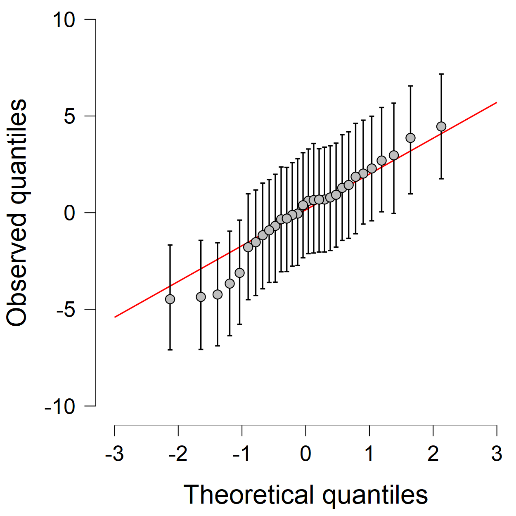


### Experiment 3

**Behavioral responses: Perception Intensity**

#### **Laser**

| **Model Comparison** | | | | | | | | | | | |
| --- | --- | --- | --- | --- | --- | --- | --- | --- | --- | --- | --- |
| **Models** | | **P(M)** | | **P(M\|data)** | | **BF _M_** | | **BF _01_** | | **error %** | |
| Null model (incl. subject) |  | 0.500 |  | 0.606 |  | 1.536 |  | 1.000 |  |  |  |
| Duty Cycle Phase |  | 0.500 |  | 0.394 |  | 0.651 |  | 1.536 |  | 1.355 |  |
|  | | | | | | | | | | | |
| Note.  All models include subject | | | | | | | | | | | |

| **Post Hoc Comparisons - Duty Cycle Phase** | | | | | | | | | | | |
| --- | --- | --- | --- | --- | --- | --- | --- | --- | --- | --- | --- |
|  | |  | | **Prior Odds** | | **Posterior Odds** | | **BF _01, U_** | | **error %** | |
| OFF |  | ON |  | 1.000 |  | 1.734 |  | 1.734 |  | 0.005 |  |
|  | | | | | | | | | | | |
| Note.  The posterior odds have been corrected for multiple testing by fixing to 0.5 the prior probability that the null hypothesis holds across all comparisons (Westfall, Johnson, & Utts, 1997). Individual comparisons are based on the default t-test with a Cauchy (0, r = 1/sqrt(2)) prior. The "U" in the Bayes factor denotes that it is uncorrected. | | | | | | | | | | | |

### Model Averaged Q-QPlot


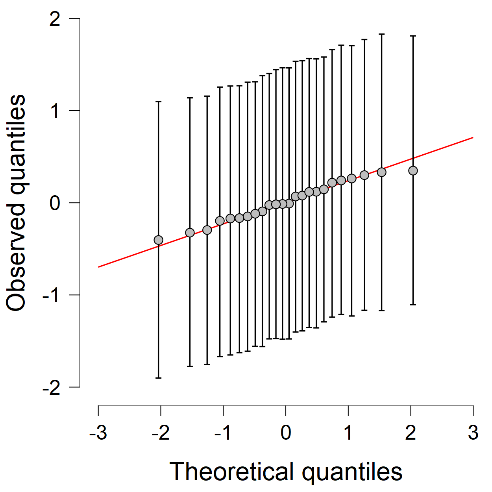


#### **Vibrotactile**

| **Model Comparison** | | | | | | | | | | | |
| --- | --- | --- | --- | --- | --- | --- | --- | --- | --- | --- | --- |
| **Models** | | **P(M)** | | **P(M\|data)** | | **BF _M_** | | **BF _01_** | | **error %** | |
| Null model (incl. subject) |  | 0.500 |  | 0.675 |  | 2.081 |  | 1.000 |  |  |  |
| Duty Cycle Phase |  | 0.500 |  | 0.325 |  | 0.481 |  | 2.081 |  | 0.930 |  |
|  | | | | | | | | | | | |
| Note.  All models include subject | | | | | | | | | | | |

| **Post Hoc Comparisons - Duty Cycle Phase** | | | | | | | | | | | |
| --- | --- | --- | --- | --- | --- | --- | --- | --- | --- | --- | --- |
|  | |  | | **Prior Odds** | | **Posterior Odds** | | **BF _01, U_** | | **error %** | |
| OFF |  | ON |  | 1.000 |  | 2.476 |  | 2.476 |  | 0.017 |  |
|  | | | | | | | | | | | |
| Note.  The posterior odds have been corrected for multiple testing by fixing to 0.5 the prior probability that the null hypothesis holds across all comparisons (Westfall, Johnson, & Utts, 1997). Individual comparisons are based on the default t-test with a Cauchy (0, r = 1/sqrt(2)) prior. The "U" in the Bayes factor denotes that it is uncorrected. | | | | | | | | | | | |

### Model Averaged Q-Q Plot


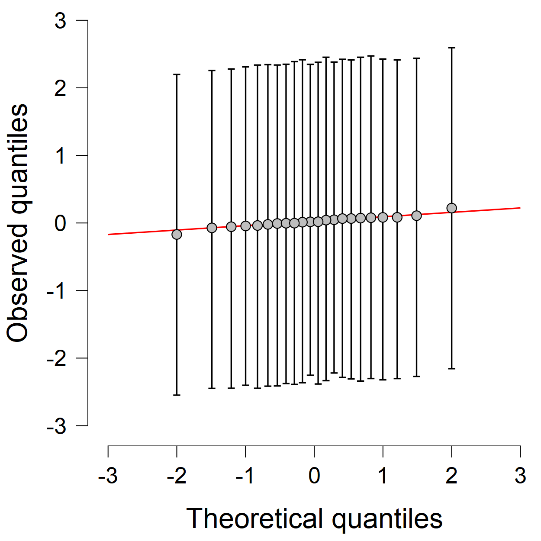


**Cerebral responses**

#### **Laser-evoked ERPs : P2 latency:**

| **Model Comparison** | | | | | | | | | | | |
| --- | --- | --- | --- | --- | --- | --- | --- | --- | --- | --- | --- |
| **Models** | | **P(M)** | | **P(M\|data)** | | **BF _M_** | | **BF _01_** | | **error %** | |
| Null model (incl. subject) |  | 0.500 |  | 0.707 |  | 2.415 |  | 1.000 |  |  |  |
| Duty Cycle Phase |  | 0.500 |  | 0.293 |  | 0.414 |  | 2.415 |  | 1.006 |  |
|  | | | | | | | | | | | |
| Note.  All models include subject | | | | | | | | | | | |

### Model Averaged Q-Q Plot

| **Post Hoc Comparisons - Duty Cycle Phase** | | | | | | | | | | | |
| --- | --- | --- | --- | --- | --- | --- | --- | --- | --- | --- | --- |
|  | |  | | **Prior Odds** | | **Posterior Odds** | | **BF _01, U_** | | **error %** | |
| OFF |  | ON |  | 1.000 |  | 2.984 |  | 2.984 |  | 0.017 |  |
|  | | | | | | | | | | | |
| Note.  The posterior odds have been corrected for multiple testing by fixing to 0.5 the prior probability that the null hypothesis holds across all comparisons (Westfall, Johnson, & Utts, 1997). Individual comparisons are based on the default t-test with a Cauchy (0, r = 1/sqrt(2)) prior. The "U" in the Bayes factor denotes that it is uncorrected. | | | | | | | | | | | |


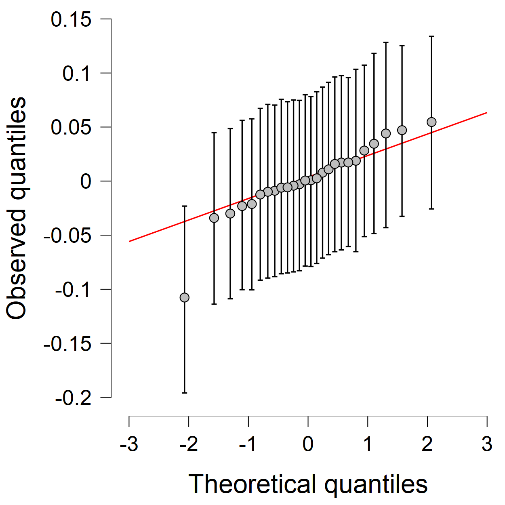


#### **Laser-evoked ERPs : P2 amplitude:**

| **Model Comparison** | | | | | | | | | | | |
| --- | --- | --- | --- | --- | --- | --- | --- | --- | --- | --- | --- |
| **Models** | | **P(M)** | | **P(M\|data)** | | **BF _M_** | | **BF _01_** | | **error %** | |
| Null model (incl. subject) |  | 0.500 |  | 0.704 |  | 2.376 |  | 1.000 |  |  |  |
| Duty Cycle Phase |  | 0.500 |  | 0.296 |  | 0.421 |  | 2.376 |  | 0.769 |  |
|  | | | | | | | | | | | |
| Note.  All models include subject | | | | | | | | | | | |

| **Post Hoc Comparisons - Duty Cycle Phase** | | | | | | | | | | | |
| --- | --- | --- | --- | --- | --- | --- | --- | --- | --- | --- | --- |
|  | |  | | **Prior Odds** | | **Posterior Odds** | | **BF _01, U_** | | **error %** | |
| OFF |  | ON |  | 1.000 |  | 2.966 |  | 2.966 |  | 0.017 |  |
|  | | | | | | | | | | | |
| Note.  The posterior odds have been corrected for multiple testing by fixing to 0.5 the prior probability that the null hypothesis holds across all comparisons (Westfall, Johnson, & Utts, 1997). Individual comparisons are based on the default t-test with a Cauchy (0, r = 1/sqrt(2)) prior. The "U" in the Bayes factor denotes that it is uncorrected. | | | | | | | | | | | |

### Model averaged Q-Q Plot


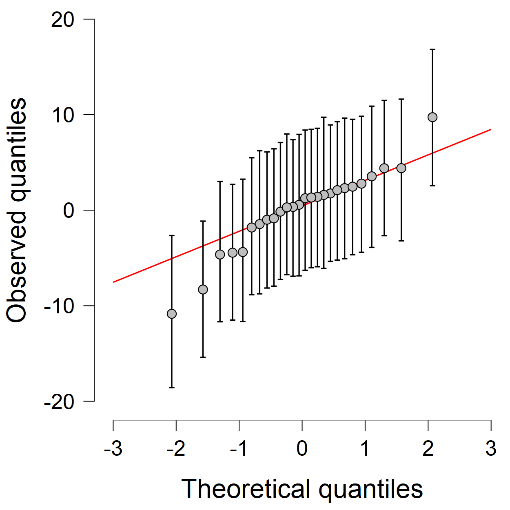


#### **Laser-evoked ERPs : N2 latency:**

| **Model Comparison** | | | | | | | | | | | |
| --- | --- | --- | --- | --- | --- | --- | --- | --- | --- | --- | --- |
| **Models** | | **P(M)** | | **P(M\|data)** | | **BF _M_** | | **BF _01_** | | **error %** | |
| Null model (incl. subject) |  | 0.500 |  | 0.734 |  | 2.758 |  | 1.000 |  |  |  |
| Duty Cycle Phase |  | 0.500 |  | 0.266 |  | 0.363 |  | 2.758 |  | 1.805 |  |
|  | | | | | | | | | | | |
| Note.  All models include subject | | | | | | | | | | | |

### Model Averaged Q-Q Plot

| **Post Hoc Comparisons - Duty Cycle Phase** | | | | | | | | | | | |
| --- | --- | --- | --- | --- | --- | --- | --- | --- | --- | --- | --- |
|  | |  | | **Prior Odds** | | **Posterior Odds** | | **BF _01, U_** | | **error %** | |
| OFF |  | ON |  | 1.000 |  | 3.571 |  | 3.571 |  | 0.016 |  |
|  | | | | | | | | | | | |
| Note.  The posterior odds have been corrected for multiple testing by fixing to 0.5 the prior probability that the null hypothesis holds across all comparisons (Westfall, Johnson, & Utts, 1997). Individual comparisons are based on the default t-test with a Cauchy (0, r = 1/sqrt(2)) prior. The "U" in the Bayes factor denotes that it is uncorrected. | | | | | | | | | | | |


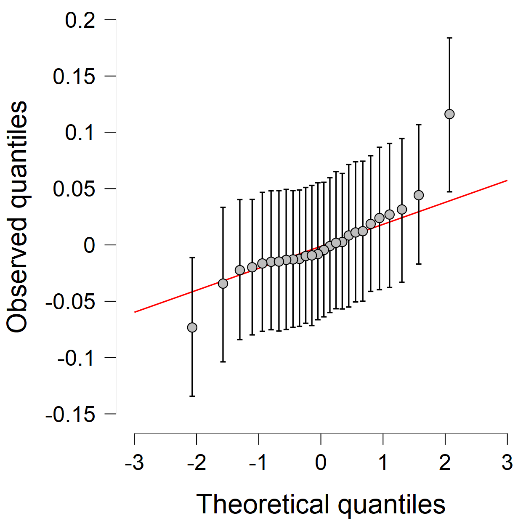


#### **Laser-evoked ERPs : N2 amplitude:**

| **Model Comparison** | | | | | | | | | | | |
| --- | --- | --- | --- | --- | --- | --- | --- | --- | --- | --- | --- |
| **Models** | | **P(M)** | | **P(M\|data)** | | **BF _M_** | | **BF _01_** | | **error %** | |
| Null model (incl. subject) |  | 0.500 |  | 0.645 |  | 1.817 |  | 1.000 |  |  |  |
| Duty Cycle Phase |  | 0.500 |  | 0.355 |  | 0.550 |  | 1.817 |  | 0.873 |  |
|  | | | | | | | | | | | |
| Note.  All models include subject | | | | | | | | | | | |

### Model Averaged Q-Q Plot

| **Post Hoc Comparisons - Duty Cycle Phase** | | | | | | | | | | | |
| --- | --- | --- | --- | --- | --- | --- | --- | --- | --- | --- | --- |
|  | |  | | **Prior Odds** | | **Posterior Odds** | | **BF _01, U_** | | **error %** | |
| OFF |  | ON |  | 1.000 |  | 2.090 |  | 2.090 |  | 0.021 |  |
|  | | | | | | | | | | | |
| Note.  The posterior odds have been corrected for multiple testing by fixing to 0.5 the prior probability that the null hypothesis holds across all comparisons (Westfall, Johnson, & Utts, 1997). Individual comparisons are based on the default t-test with a Cauchy (0, r = 1/sqrt(2)) prior. The "U" in the Bayes factor denotes that it is uncorrected. | | | | | | | | | | | |


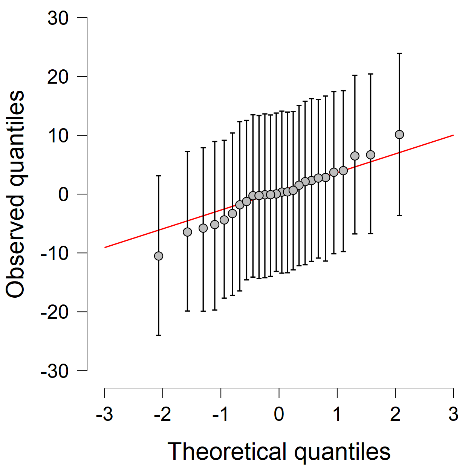


#### **Laser-evoked ERPs : N2P2 amplitude:**

| **Model Comparison** | | | | | | | | | | | |
| --- | --- | --- | --- | --- | --- | --- | --- | --- | --- | --- | --- |
| **Models** | | **P(M)** | | **P(M\|data)** | | **BF _M_** | | **BF _01_** | | **error %** | |
| Null model (incl. subject) |  | 0.500 |  | 0.376 |  | 0.603 |  | 1.000 |  |  |  |
| Duty Cycle Phase |  | 0.500 |  | 0.624 |  | 1.657 |  | 0.603 |  | 1.543 |  |
|  | | | | | | | | | | | |
| Note.  All models include subject | | | | | | | | | | | |

| **Post Hoc Comparisons - Duty Cycle Phase** | | | | | | | | | | | |
| --- | --- | --- | --- | --- | --- | --- | --- | --- | --- | --- | --- |
|  | |  | | **Prior Odds** | | **Posterior Odds** | | **BF _01, U_** | | **error %** | |
| OFF |  | ON |  | 1.000 |  | 0.556 |  | 0.556 |  | 7.889e -4 |  |
|  | | | | | | | | | | | |
| Note.  The posterior odds have been corrected for multiple testing by fixing to 0.5 the prior probability that the null hypothesis holds across all comparisons (Westfall, Johnson, & Utts, 1997). Individual comparisons are based on the default t-test with a Cauchy (0, r = 1/sqrt(2)) prior. The "U" in the Bayes factor denotes that it is uncorrected. | | | | | | | | | | | |

### Model Averaged Q-Q Plot


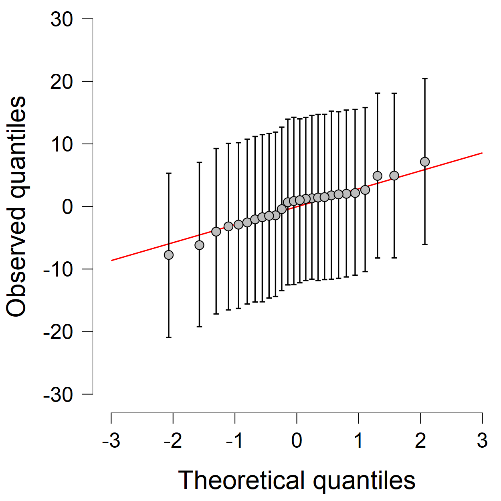


| **Post Hoc Comparisons - Duty Cycle Phase** | | | | | | | | | | | |
| --- | --- | --- | --- | --- | --- | --- | --- | --- | --- | --- | --- |
|  | | | | | | | | | | | |
|  | |  | | **Prior Odds** | | **Posterior Odds** | | **BF _01, U_** | | **error %** | |
| OFF |  | ON |  | 1.000 |  | 1.220 |  | 1.220 |  | 0.005 |  |
|  | | | | | | | | | | | |
| Note.  The posterior odds have been corrected for multiple testing by fixing to 0.5 the prior probability that the null hypothesis holds across all comparisons (Westfall, Johnson, & Utts, 1997). Individual comparisons are based on the default t-test with a Cauchy (0, r = 1/sqrt(2)) prior. The "U" in the Bayes factor denotes that it is uncorrected. | | | | | | | | | | | |

#### **Vibrotactile-evoked ERPs : P2 latency:**

| **Model Comparison** | | | | | | | | | | | |
| --- | --- | --- | --- | --- | --- | --- | --- | --- | --- | --- | --- |
| **Models** | | **P(M)** | | **P(M\|data)** | | **BF _M_** | | **BF _01_** | | **error %** | |
| Null model (incl. subject) |  | 0.500 |  | 0.529 |  | 1.125 |  | 1.000 |  |  |  |
| Duty Cycle Phase |  | 0.500 |  | 0.471 |  | 0.889 |  | 1.125 |  | 1.102 |  |
|  | | | | | | | | | | | |
| Note.  All models include subject | | | | | | | | | | | |

### Model Averaged Q-Q Plot


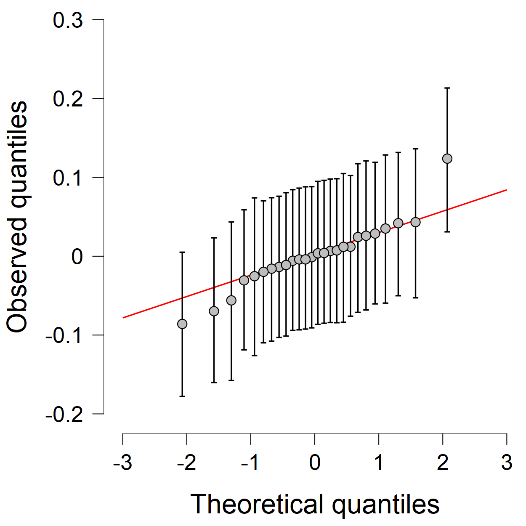


#### **Vibrotactile-evoked ERPs : P2 amplitude:**

| **Model Comparison** | | | | | | | | | | | |
| --- | --- | --- | --- | --- | --- | --- | --- | --- | --- | --- | --- |
| **Models** | | **P(M)** | | **P(M\|data)** | | **BF _M_** | | **BF _01_** | | **error %** | |
| Null model (incl. subject) |  | 0.500 |  | 0.520 |  | 1.084 |  | 1.000 |  |  |  |
| Duty Cycle Phase |  | 0.500 |  | 0.480 |  | 0.923 |  | 1.084 |  | 1.126 |  |
|  | | | | | | | | | | | |
| Note.  All models include subject | | | | | | | | | | | |

### Model Averaged Q-Q Plot

| **Post Hoc Comparisons - Duty Cycle Phase** | | | | | | | | | | | |
| --- | --- | --- | --- | --- | --- | --- | --- | --- | --- | --- | --- |
|  | |  | | **Prior Odds** | | **Posterior Odds** | | **BF _01, U_** | | **error %** | |
| OFF |  | ON |  | 1.000 |  | 1.256 |  | 1.256 |  | 0.005 |  |
|  | | | | | | | | | | | |
| Note.  The posterior odds have been corrected for multiple testing by fixing to 0.5 the prior probability that the null hypothesis holds across all comparisons (Westfall, Johnson, & Utts, 1997). Individual comparisons are based on the default t-test with a Cauchy (0, r = 1/sqrt(2)) prior. The "U" in the Bayes factor denotes that it is uncorrected. | | | | | | | | | | | |


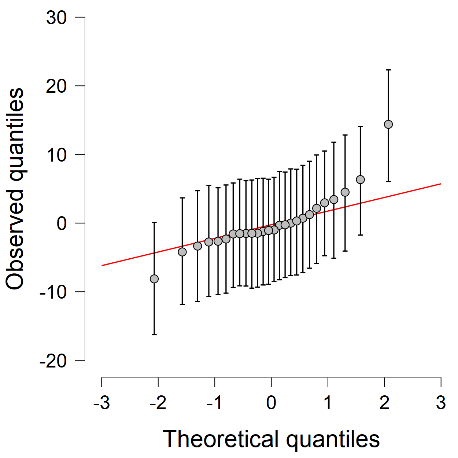


#### **Vibrotactile-evoked ERPs : N2 latency:**

| **Model Comparison** | | | | | | | | | | | |
| --- | --- | --- | --- | --- | --- | --- | --- | --- | --- | --- | --- |
| **Models** | | **P(M)** | | **P(M\|data)** | | **BF _M_** | | **BF _01_** | | **error %** | |
| Null model (incl. subject) |  | 0.500 |  | 0.738 |  | 2.811 |  | 1.000 |  |  |  |
| Duty Cycle Phase |  | 0.500 |  | 0.262 |  | 0.356 |  | 2.811 |  | 1.103 |  |
|  | | | | | | | | | | | |
| Note.  All models include subject | | | | | | | | | | | |

| **Post Hoc Comparisons - Duty Cycle Phase** | | | | | | | | | | | |
| --- | --- | --- | --- | --- | --- | --- | --- | --- | --- | --- | --- |
|  | |  | | **Prior Odds** | | **Posterior Odds** | | **BF _01, U_** | | **error %** | |
| OFF |  | ON |  | 1.000 |  | 3.593 |  | 3.593 |  | 0.016 |  |
|  | | | | | | | | | | | |
| Note.  The posterior odds have been corrected for multiple testing by fixing to 0.5 the prior probability that the null hypothesis holds across all comparisons (Westfall, Johnson, & Utts, 1997). Individual comparisons are based on the default t-test with a Cauchy (0, r = 1/sqrt(2)) prior. The "U" in the Bayes factor denotes that it is uncorrected. | | | | | | | | | | | |

### Model Averaged Q-Q Plot


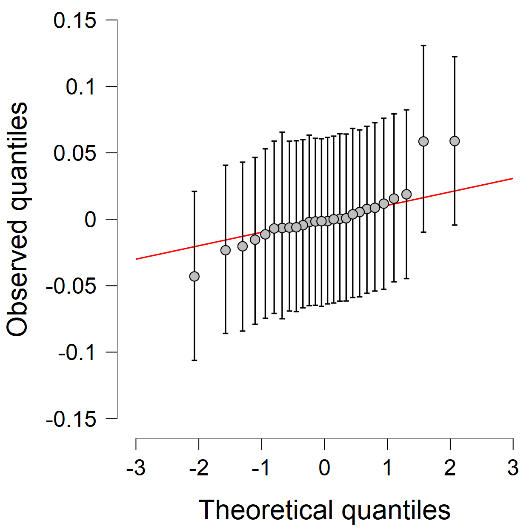


#### **Vibrotactile-evoked ERPs : N2 amplitude:**

| **Model Comparison** | | | | | | | | | | | |
| --- | --- | --- | --- | --- | --- | --- | --- | --- | --- | --- | --- |
| **Models** | | **P(M)** | | **P(M\|data)** | | **BF _M_** | | **BF _01_** | | **error %** | |
| Null model (incl. subject) |  | 0.500 |  | 0.712 |  | 2.474 |  | 1.000 |  |  |  |
| Duty Cycle Phase |  | 0.500 |  | 0.288 |  | 0.404 |  | 2.474 |  | 2.283 |  |
|  | | | | | | | | | | | |
| Note.  All models include subject | | | | | | | | | | | |

| **Post Hoc Comparisons - Duty Cycle Phase** | | | | | | | | | | | |
| --- | --- | --- | --- | --- | --- | --- | --- | --- | --- | --- | --- |
|  | |  | | **Prior Odds** | | **Posterior Odds** | | **BF _01, U_** | | **error %** | |
| OFF |  | ON |  | 1.000 |  | 3.223 |  | 3.223 |  | 0.017 |  |
|  | | | | | | | | | | | |
| Note.  The posterior odds have been corrected for multiple testing by fixing to 0.5 the prior probability that the null hypothesis holds across all comparisons (Westfall, Johnson, & Utts, 1997). Individual comparisons are based on the default t-test with a Cauchy (0, r = 1/sqrt(2)) prior. The "U" in the Bayes factor denotes that it is uncorrected. | | | | | | | | | | | |

### Model Averaged Q-Q Plot


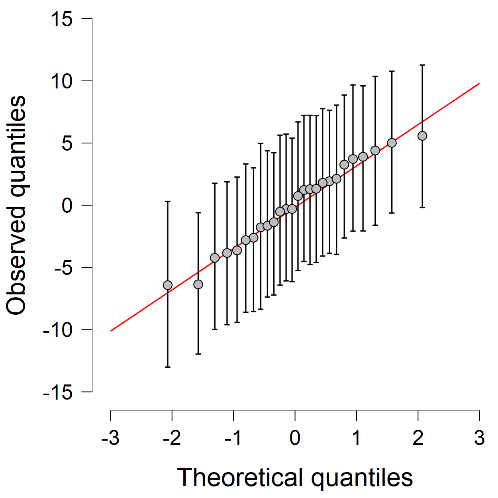


#### **Vibrotactile-evoked ERPs : N2P2 amplitude:**

| **Model Comparison** | | | | | | | | | | | |
| --- | --- | --- | --- | --- | --- | --- | --- | --- | --- | --- | --- |
| **Models** | | **P(M)** | | **P(M\|data)** | | **BF _M_** | | **BF _01_** | | **error %** | |
| Null model (incl. subject) |  | 0.500 |  | 0.330 |  | 0.493 |  | 1.000 |  |  |  |
| Duty Cycle Phase |  | 0.500 |  | 0.670 |  | 2.029 |  | 0.493 |  | 1.937 |  |
|  | | | | | | | | | | | |
| Note.  All models include subject | | | | | | | | | | | |

| **Post Hoc Comparisons - Duty Cycle Phase** | | | | | | | | | | | |
| --- | --- | --- | --- | --- | --- | --- | --- | --- | --- | --- | --- |
|  | |  | | **Prior Odds** | | **Posterior Odds** | | **BF _01, U_** | | **error %** | |
| OFF |  | ON |  | 1.000 |  | 0.511 |  | 0.511 |  | 0.001 |  |
|  | | | | | | | | | | | |
| Note.  The posterior odds have been corrected for multiple testing by fixing to 0.5 the prior probability that the null hypothesis holds across all comparisons (Westfall, Johnson, & Utts, 1997). Individual comparisons are based on the default t-test with a Cauchy (0, r = 1/sqrt(2)) prior. The "U" in the Bayes factor denotes that it is uncorrected. | | | | | | | | | | | |

### Model Averaged Q-Q Plot


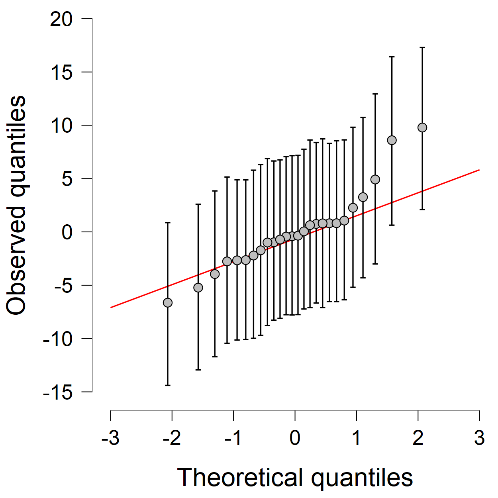

Supplement: S1 Appendix — (ZIP) [file pone.0254480.s001.zip › Supplementary Appendix_Bayesian statistics.docx]
