# Supplementary material for: Transcutaneous auricular VNS applied to experimental pain: A paired behavioral and EEG study using thermonociceptive CO2 laser
Source: PLoS One. 2021 Jul 12;16(7):e0254480. doi: 10.1371/journal.pone.0254480 (PMC8274876; doi:10.1371/journal.pone.0254480)
Supplement: S1 Appendix — (ZIP) [file pone.0254480.s001.zip › Supplementary Appendix_Bayesian statistics.pdf]

Transcutaneous VNS applied to experimental pain: a paired behavioral and EEG study using thermonociceptive CO2 laser

Supplementary Appendix

Bayesian Repeated Measures ANOVA (conducted using Jasp 0.14.00)

Experiment 1

Behavioral responses

Detection thresholds

- *Heat sensitive C- fibers*

Model Comparison

Models	P(M)	P(M|data)	BF_M	BF_{01}	error %
Null model (incl. subject)	0.053	0.473	16.177	1.000	
Time	0.053	0.158	3.373	2.999	1.205
Condition	0.053	0.148	3.133	3.193	1.798
Condition + Time	0.053	0.049	0.931	9.626	1.669
Volunteers	0.053	0.038	0.714	12.412	0.223
Condition + Time + Volunteers + Condition ✻ Time + Condition ✻ Volunteers + Time ✻ Volunteers + Condition ✻ Time ✻ Volunteers	0.053	0.037	0.685	12.912	13.069
Condition + Time + Condition ✻ Time	0.053	0.025	0.459	19.016	2.082
Condition + Volunteers + Condition ✻ Volunteers	0.053	0.014	0.264	32.779	1.056
Time + Volunteers	0.053	0.013	0.233	36.967	1.207
Condition + Volunteers	0.053	0.012	0.222	38.904	1.168
Time + Volunteers + Time ✻ Volunteers	0.053	0.006	0.114	75.083	1.026
Condition + Time + Volunteers + Condition ✻ Volunteers + Time ✻ Volunteers	0.053	0.005	0.096	89.512	2.362
Condition + Time + Volunteers + Condition ✻ Volunteers	0.053	0.005	0.095	89.985	2.313
Condition + Time + Volunteers	0.053	0.004	0.073	116.954	1.348
Condition + Time + Volunteers + Condition ✻ Time + Condition ✻ Volunteers + Time ✻ Volunteers	0.053	0.003	0.058	148.586	1.762

Model Comparison

Models	P(M)	P(M|data)	BF M	BF 01	error %
Condition + Time + Volunteers + Condition ✻ Time + Condition ✻ Volunteers	0.053	0.003	0.052	163.372	1.978
Condition + Time + Volunteers + Condition ✻ Time	0.053	0.002	0.038	223.500	2.421
Condition + Time + Volunteers + Time ✻ Volunteers	0.053	0.002	0.038	227.527	1.475
Condition + Time + Volunteers + Condition ✻ Time + Time ✻ Volunteers	0.053	0.001	0.021	410.447	4.793

Note. All models include subject

Model Averaged Q-Q Plot

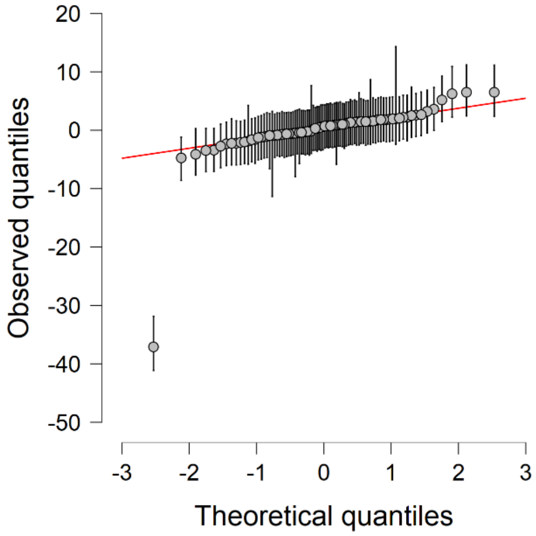

Post Hoc Comparisons - Condition

		Prior Odds	Posterior Odds	BF 01, U	error %
taVNS	Sham	1.000	4.493	4.493	1.141e -5

Post Hoc Comparisons - Time

		Prior Odds	Posterior Odds	BF 01, U	error %
T1-T0	T2-T0	1.000	4.105	4.105	1.070e -5

Note. The posterior odds have been corrected for multiple testing by fixing to 0.5 the prior probability that the null hypothesis holds across all comparisons (Westfall, Johnson, & Utts, 1997). Individual comparisons are based on the default t-test with a Cauchy (0, r = 1/sqrt(2)) prior. The "U" in the Bayes factor denotes that it is uncorrected.

- *Heat sensitive Aδ-fibers*

Model Comparison

Models	P(M)	P(M|data)	BF M	BF 01	error %
Null model (incl. subject)	0.053	0.485	16.965	1.000	
Time	0.053	0.198	4.431	2.456	0.976
Condition	0.053	0.118	2.401	4.123	3.042
Volunteers	0.053	0.048	0.911	10.076	0.191
Condition + Time	0.053	0.048	0.903	10.153	2.380

Model Comparison

Models	P(M)	P(M|data)	BF $_M$	BF $_{01}$	error %
Time + Volunteers	0.053	0.020	0.359	24.837	0.897
Condition + Time + Condition ✻ Time	0.053	0.019	0.348	25.580	4.528
Condition + Time + Volunteers + Condition ✻ Time + Condition ✻ Volunteers + Time ✻ Volunteers + Condition ✻ Time ✻ Volunteers	0.053	0.013	0.230	38.523	21.512
Condition + Volunteers	0.053	0.012	0.212	41.592	1.175
Time + Volunteers + Time ✻ Volunteers	0.053	0.011	0.194	45.514	0.871
Condition + Volunteers + Condition ✻ Volunteers	0.053	0.009	0.161	54.820	2.382
Condition + Time + Volunteers	0.053	0.005	0.088	99.515	1.869
Condition + Time + Volunteers + Condition ✻ Volunteers	0.053	0.004	0.069	126.791	1.490
Condition + Time + Volunteers + Condition ✻ Volunteers + Time ✻ Volunteers	0.053	0.003	0.063	138.732	1.759
Condition + Time + Volunteers + Time ✻ Volunteers	0.053	0.003	0.047	187.984	1.364
Condition + Time + Volunteers + Condition ✻ Time + Condition ✻ Volunteers + Time ✻ Volunteers	0.053	0.002	0.036	245.575	19.150
Condition + Time + Volunteers + Condition ✻ Time	0.053	0.002	0.034	259.515	2.476
Condition + Time + Volunteers + Condition ✻ Time + Condition ✻ Volunteers	0.053	0.002	0.030	288.629	5.177
Condition + Time + Volunteers + Condition ✻ Time + Time ✻ Volunteers	0.053	0.001	0.019	470.455	2.078

Note. All models include subject

Post Hoc Comparisons - Condition

		Prior Odds	Posterior Odds	BF $_{01, U}$	error %
taVNS	Sham	1.000	5.617	5.617	1.327e -5

Post Hoc Comparisons - Time

		Prior Odds	Posterior Odds	BF $_{01, U}$	error %
T1-T0	T2-T0	1.000	3.435	3.435	9.383e -6

Model Averaged Q-Q Plot

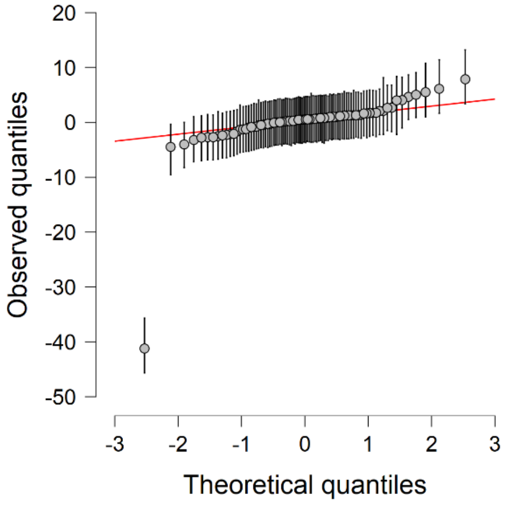

Note. The posterior odds have been corrected for multiple testing by fixing to 0.5 the prior probability that the null hypothesis holds across all comparisons (Westfall, Johnson, & Utts, 1997). Individual comparisons are based on the default t-test with a Cauchy (0, r = 1/sqrt(2)) prior. The "U" in the Bayes factor denotes that it is uncorrected.

- *Mechanosensitive Aβ-fibers*

Model Comparison

Models	P(M)	P(M|data)	BF_M	BF_{01}	error %
Null model (incl. subject)	0.053	0.263	6.434	1.000	
Condition	0.053	0.191	4.249	1.379	0.878
Volunteers	0.053	0.123	2.517	2.146	0.974
Condition + Volunteers	0.053	0.099	1.968	2.672	4.500
Time	0.053	0.066	1.278	3.973	1.576
Condition + Volunteers + Condition ✻ Volunteers	0.053	0.060	1.154	4.369	3.448
Condition + Time	0.053	0.047	0.891	5.582	1.337
Time + Volunteers	0.053	0.030	0.558	8.752	1.656
Condition + Time + Volunteers	0.053	0.024	0.443	10.959	5.057
Condition + Time + Condition ✻ Time	0.053	0.019	0.358	13.518	2.167
Condition + Time + Volunteers + Condition ✻ Volunteers	0.053	0.014	0.264	18.188	2.506
Time + Volunteers + Time ✻ Volunteers	0.053	0.012	0.216	22.229	2.589
Condition + Time + Volunteers + Condition ✻ Time	0.053	0.011	0.198	24.188	10.197
Condition + Time + Volunteers + Time ✻ Volunteers	0.053	0.011	0.197	24.287	5.204

Model Comparison

Models	P(M)	P(M|data)	BF M	BF 01	error %
Condition + Time + Volunteers + Condition ✻ Volunteers + Time ✻ Volunteers	0.053	0.010	0.184	26.005	2.821
Condition + Time + Volunteers + Condition ✻ Time + Condition ✻ Volunteers	0.053	0.006	0.117	40.752	11.386
Condition + Time + Volunteers + Condition ✻ Time + Condition ✻ Volunteers + Time ✻ Volunteers	0.053	0.005	0.082	58.279	8.521
Condition + Time + Volunteers + Condition ✻ Time + Condition ✻ Volunteers + Time ✻ Volunteers + Condition ✻ Time ✻ Volunteers	0.053	0.004	0.074	64.150	4.477
Condition + Time + Volunteers + Condition ✻ Time + Time ✻ Volunteers	0.053	0.004	0.071	67.225	2.712

Note. All models include subject

Model Averaged Q-Q Plot

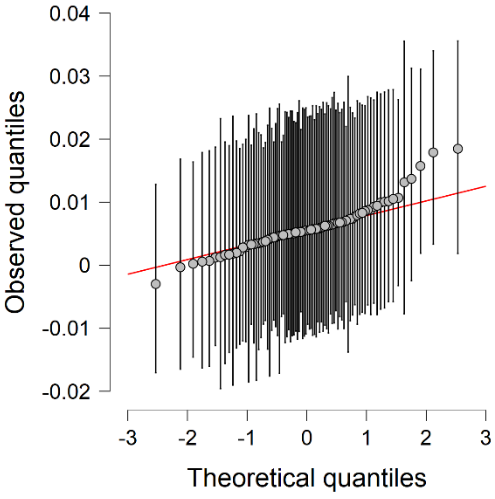

Post Hoc Comparisons - Condition

		Prior Odds	Posterior Odds	BF 01, U	error %
taVNS	Sham	1.000	1.744	1.744	5.327e -6

Post Hoc Comparisons - Time

		Prior Odds	Posterior Odds	BF 01, U	error %
T1-T0	T2-T0	1.000	5.362	5.362	1.287e -5

Note. The posterior odds have been corrected for multiple testing by fixing to 0.5 the prior probability that the null hypothesis holds across all comparisons (Westfall, Johnson, & Utts, 1997). Individual comparisons are based on the default t-test with a Cauchy (0, r = 1/sqrt(2)) prior. The "U" in the Bayes factor denotes that it is uncorrected.

- *Cool sensitive Aδ-fibers*

Model Comparison

Models	P(M)	P(M|data)	BF M	BF 01	error %
Null model (incl. subject)	0.053	0.463	15.537	1.000	
Time	0.053	0.155	3.313	2.980	2.355

Model Comparison

Models	P(M)	P(M|data)	BF_{M}	BF_{01}	error %
Condition	0.053	0.150	3.189	3.078	1.022
Volunteers	0.053	0.055	1.043	8.459	0.173
Condition + Time	0.053	0.050	0.948	9.264	1.442
Condition + Time + Condition ✻ Time	0.053	0.026	0.488	17.561	14.077
Condition + Volunteers	0.053	0.018	0.338	25.123	2.429
Time + Volunteers	0.053	0.018	0.335	25.376	1.250
Condition + Volunteers + Condition ✻ Volunteers	0.053	0.017	0.306	27.720	1.629
Condition + Time + Volunteers + Condition ✻ Time + Condition ✻ Volunteers + Time ✻ Volunteers + Condition ✻ Time ✻ Volunteers	0.053	0.009	0.157	53.477	25.915
Time + Volunteers + Time ✻ Volunteers	0.053	0.008	0.148	56.769	3.076
Condition + Time + Volunteers	0.053	0.007	0.123	68.214	9.548
Condition + Time + Volunteers + Condition ✻ Volunteers	0.053	0.006	0.117	71.992	6.875
Condition + Time + Volunteers + Condition ✻ Volunteers + Time ✻ Volunteers	0.053	0.004	0.080	105.309	2.423
Condition + Time + Volunteers + Condition ✻ Time	0.053	0.003	0.051	165.446	4.700
Condition + Time + Volunteers + Time ✻ Volunteers	0.053	0.003	0.050	168.309	1.591
Condition + Time + Volunteers + Condition ✻ Time + Condition ✻ Volunteers	0.053	0.003	0.049	171.250	2.292
Condition + Time + Volunteers + Condition ✻ Time + Condition ✻ Volunteers + Time ✻ Volunteers	0.053	0.002	0.041	205.425	2.704
Condition + Time + Volunteers + Condition ✻ Time + Time ✻ Volunteers	0.053	0.001	0.022	374.635	2.179

Note. All models include subject

Post Hoc Comparisons - Condition

		Prior Odds	Posterior Odds	$BF_{01, U}$	error %
taVNS	Sham	1.000	4.275	4.275	1.101e -5

Model Averaged Q-Q Plots

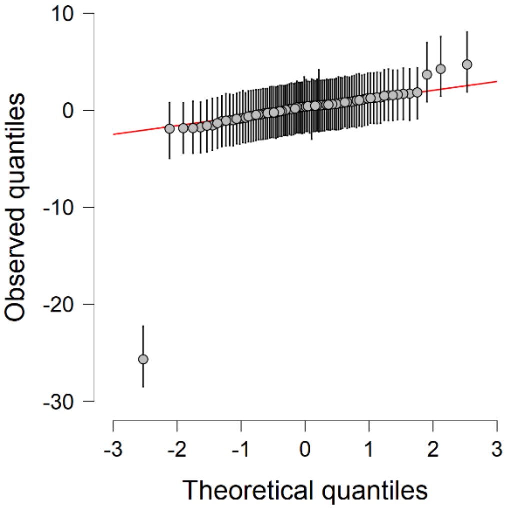

Post Hoc Comparisons - Time

		Prior Odds	Posterior Odds	BF $_{01, U}$	error %
T1-T0	T2-T0	1.000	4.080	4.080	1.065e -5

Note. The posterior odds have been corrected for multiple testing by fixing to 0.5 the prior probability that the null hypothesis holds across all comparisons (Westfall, Johnson, & Utts, 1997). Individual comparisons are based on the default t-test with a Cauchy (0, r = 1/sqrt(2)) prior. The "U" in the Bayes factor denotes that it is uncorrected.

Perception Intensity

- *Laser*

Model Comparison

Models	P(M)	P(M|data)	BF $_M$	BF $_{01}$	error %
Null model (incl. subject)	0.053	0.019	0.353	1.000	
Condition + Volunteers + Condition ✻ Volunteers	0.053	0.484	16.853	0.040	3.715
Condition + Time + Volunteers + Condition ✻ Volunteers + Time ✻ Volunteers	0.053	0.159	3.406	0.121	4.774
Condition + Time + Volunteers + Condition ✻ Volunteers	0.053	0.153	3.261	0.125	7.267
Condition + Time + Volunteers + Condition ✻ Time + Condition ✻ Volunteers + Time ✻ Volunteers	0.053	0.078	1.519	0.247	19.040
Condition + Time + Volunteers + Condition ✻ Time + Condition ✻ Volunteers	0.053	0.048	0.908	0.401	5.369

Model Comparison

Models	P(M)	P(M|data)	BF_M	BF_{01}	error %
Condition + Time + Volunteers + Condition ✻ Time + Condition ✻ Volunteers + Time ✻ Volunteers + Condition ✻ Time ✻ Volunteers	0.053	0.033	0.606	0.591	14.171
Volunteers	0.053	0.010	0.176	1.991	1.503
Time	0.053	0.005	0.086	4.034	1.344
Condition	0.053	0.005	0.084	4.151	2.132
Time + Volunteers	0.053	0.002	0.041	8.454	1.535
Condition + Volunteers	0.053	0.002	0.039	8.908	1.597
Condition + Time	0.053	0.001	0.020	17.036	2.668
Condition + Time + Volunteers	0.053	5.999e -4	0.011	32.069	3.218
Time + Volunteers + Time ✻ Volunteers	0.053	4.411e -4	0.008	43.612	2.190
Condition + Time + Condition ✻ Time	0.053	3.253e -4	0.006	59.141	1.780
Condition + Time + Volunteers + Condition ✻ Time	0.053	1.750e -4	0.003	109.920	6.222
Condition + Time + Volunteers + Time ✻ Volunteers	0.053	1.065e -4	0.002	180.717	4.981
Condition + Time + Volunteers + Condition ✻ Time + Time ✻ Volunteers	0.053	3.152e -5	5.674e -4	610.408	2.897

Note. All models include subject

Post Hoc Comparisons - Time

		Prior Odds	Posterior Odds	$BF_{01, U}$	error %
T1-T0	T2-T0	1.000	4.833	4.833	1.200e -5

Model Averaged Q-Q Plot

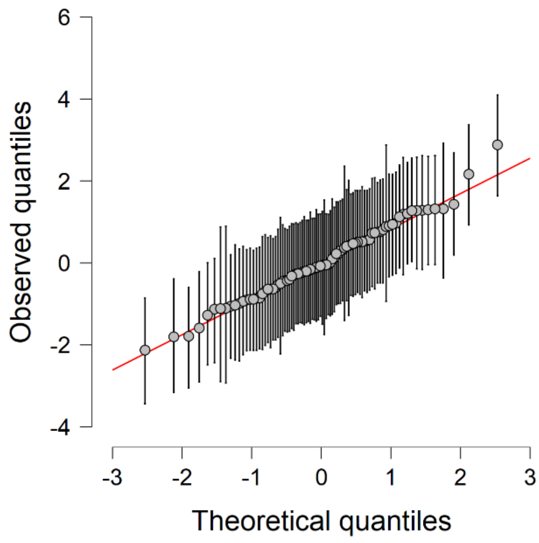

Post Hoc Comparisons - Condition

		Prior Odds	Posterior Odds	BF $_{01, U}$	error %
taVNS	Sham	1.000	5.827	5.827	1.359e -5

Note. The posterior odds have been corrected for multiple testing by fixing to 0.5 the prior probability that the null hypothesis holds across all comparisons (Westfall, Johnson, & Utts, 1997). Individual comparisons are based on the default t-test with a Cauchy (0, r = 1/sqrt(2)) prior. The "U" in the Bayes factor denotes that it is uncorrected.

- *Vibrotactile*

Model Comparison

Models	P(M)	P(M|data)	BF $_M$	BF $_{01}$	error %
Null model (incl. subject)	0.053	7.751e -7	1.395e -5	1.000	
Condition + Time + Volunteers + Condition ✻ Time + Condition ✻ Volunteers	0.053	0.534	20.636	1.451e -6	94.448
Condition + Volunteers + Condition ✻ Volunteers	0.053	0.359	10.092	2.158e -6	15.563
Condition + Time + Volunteers + Condition ✻ Volunteers	0.053	0.081	1.591	9.543e -6	5.168
Condition + Time + Volunteers + Condition ✻ Volunteers + Time ✻ Volunteers	0.053	0.011	0.194	7.251e -5	10.555
Condition + Time + Volunteers + Condition ✻ Time + Condition ✻ Volunteers + Time ✻ Volunteers + Condition ✻ Time ✻ Volunteers	0.053	0.009	0.162	8.689e -5	7.579
Condition + Time + Volunteers + Condition ✻ Time + Condition ✻ Volunteers + Time ✻ Volunteers	0.053	0.006	0.105	1.336e -4	18.894
Condition	0.053	7.225e -6	1.300e -4	0.107	1.107
Condition + Volunteers	0.053	3.210e -6	5.779e -5	0.241	1.435
Condition + Time	0.053	1.560e -6	2.808e -5	0.497	1.951
Condition + Time + Volunteers	0.053	7.282e -7	1.311e -5	1.064	4.082

Model Comparison

Models	P(M)	P(M|data)	BF $_M$	BF $_{01}$	error %
Condition + Time + Condition ✻ Time	0.053	6.028e -7	1.085e -5	1.286	10.091
Volunteers	0.053	3.078e -7	5.540e -6	2.519	0.733
Condition + Time + Volunteers + Condition ✻ Time	0.053	2.466e -7	4.439e -6	3.143	2.466
Time	0.053	1.711e -7	3.079e -6	4.531	1.148
Time + Volunteers	0.053	6.504e -8	1.171e -6	11.917	1.281
Condition + Time + Volunteers + Time ✻ Volunteers	0.053	5.453e -8	9.816e -7	14.213	4.145
Condition + Time + Volunteers + Condition ✻ Time + Time ✻ Volunteers	0.053	1.814e -8	3.265e -7	42.729	1.983
Time + Volunteers + Time ✻ Volunteers	0.053	4.550e -9	8.190e -8	170.355	1.130

Note. All models include subject

Model Averaged Q-Q Plot

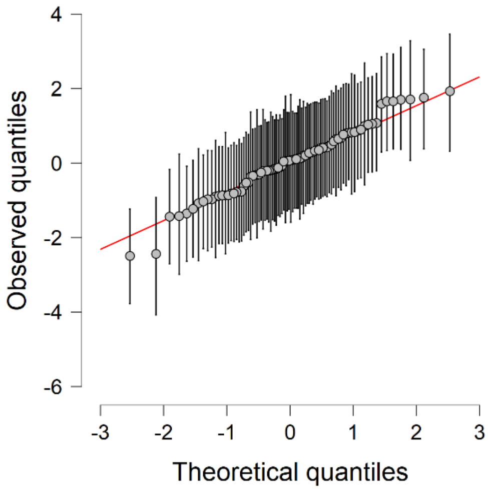

Post Hoc Comparisons - Condition

		Prior Odds	Posterior Odds	BF $_{01, U}$	error %
taVNS	Sham	1.000	0.378	0.378	9.801e -7

Post Hoc Comparisons - Time

		Prior Odds	Posterior Odds	BF $_{01, U}$	error %
T1-T0	T2-T0	1.000	6.071	6.071	1.394e -5

Note. The posterior odds have been corrected for multiple testing by fixing to 0.5 the prior probability that the null hypothesis holds across all comparisons (Westfall, Johnson, & Utts, 1997). Individual comparisons are based on the default t-test with a Cauchy (0, r = 1/sqrt(2)) prior. The "U" in the Bayes factor denotes that it is uncorrected.

- *Cool*

Model Comparison

Models	P(M)	P(M|data)	BF $_M$	BF $_{01}$	error %
Null model (incl. subject)	0.053	1.592e -6	2.866e -5	1.000	
Condition + Volunteers + Condition ✻ Volunteers	0.053	0.261	6.362	6.097e -6	16.894

Model Comparison

Models	P(M)	P(M|data)	BF $_M$	BF $_{01}$	error %
Condition + Time + Volunteers + Condition ✻ Volunteers + Time ✻ Volunteers	0.053	0.226	5.253	7.049e -6	11.009
Condition + Time + Volunteers + Condition ✻ Volunteers	0.053	0.216	4.954	7.378e -6	4.541
Condition + Time + Volunteers + Condition ✻ Time + Condition ✻ Volunteers	0.053	0.127	2.607	1.259e -5	10.136
Condition + Time + Volunteers + Condition ✻ Time + Condition ✻ Volunteers + Time ✻ Volunteers	0.053	0.123	2.522	1.296e -5	7.915
Condition + Time + Volunteers + Condition ✻ Time + Condition ✻ Volunteers + Time ✻ Volunteers + Condition ✻ Time ✻ Volunteers	0.053	0.048	0.902	3.336e -5	7.665
Condition	0.053	4.300e -6	7.740e -5	0.370	1.504
Condition + Volunteers	0.053	2.321e -6	4.178e -5	0.686	2.330
Condition + Time	0.053	1.800e -6	3.241e -5	0.884	2.351
Condition + Time + Volunteers	0.053	9.214e -7	1.658e -5	1.728	1.908
Volunteers	0.053	7.766e -7	1.398e -5	2.050	1.219
Condition + Time + Condition ✻ Time	0.053	6.278e -7	1.130e -5	2.536	2.423
Time	0.053	6.262e -7	1.127e -5	2.543	1.256
Time + Volunteers	0.053	3.342e -7	6.016e -6	4.764	4.515
Condition + Time + Volunteers + Condition ✻ Time	0.053	3.313e -7	5.964e -6	4.806	2.644
Condition + Time + Volunteers + Time ✻ Volunteers	0.053	9.782e -8	1.761e -6	16.277	2.903
Condition + Time + Volunteers + Condition ✻ Time + Time ✻ Volunteers	0.053	3.477e -8	6.259e -7	45.793	3.316
Time + Volunteers + Time ✻ Volunteers	0.053	2.792e -8	5.025e -7	57.032	2.290

Note. All models include subject

Model Averaged Q-Q Plot

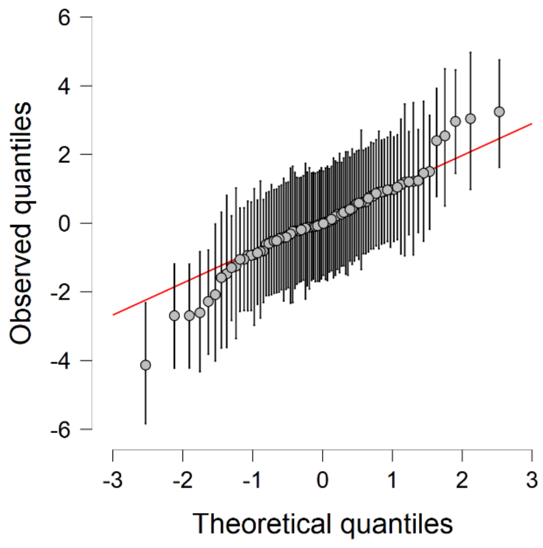

Post Hoc Comparisons - Condition

		Prior Odds	Posterior Odds	$BF_{01,U}$	error %
taVNS	Sham	1.000	0.775	0.775	2.346e -6

Post Hoc Comparisons - Time

		Prior Odds	Posterior Odds	$BF_{01,U}$	error %
T1-T0	T2-T0	1.000	0.954	0.954	2.943e -6

Note. The posterior odds have been corrected for multiple testing by fixing to 0.5 the prior probability that the null hypothesis holds across all comparisons (Westfall, Johnson, & Utts, 1997). Individual comparisons are based on the default t-test with a Cauchy (0, r = 1/sqrt(2)) prior. The "U" in the Bayes factor denotes that it is uncorrected.

- *Pinprick*

Model Comparison

Models	P(M)	P(M|data)	BF_M	BF_{01}	error %
Null model (incl. subject)	0.053	0.249	5.958	1.000	
Condition + Volunteers + Condition ✻ Volunteers	0.053	0.257	6.223	0.968	6.216
Volunteers	0.053	0.109	2.199	2.284	0.848
Condition + Time + Volunteers + Condition ✻ Volunteers	0.053	0.076	1.488	3.257	21.462
Condition	0.053	0.074	1.438	3.362	3.491
Time	0.053	0.056	1.074	4.417	1.087
Condition + Time + Volunteers + Condition ✻ Time + Condition ✻ Volunteers	0.053	0.037	0.690	6.732	6.370
Condition + Volunteers	0.053	0.031	0.582	7.937	1.435
Time + Volunteers	0.053	0.025	0.468	9.803	1.588
Condition + Time + Volunteers + Condition ✻ Volunteers + Time ✻ Volunteers	0.053	0.018	0.338	13.483	2.767
Condition + Time	0.053	0.016	0.292	15.577	2.282
Condition + Time + Volunteers + Condition ✻ Time + Condition ✻ Volunteers + Time ✻ Volunteers + Condition ✻ Time ✻ Volunteers	0.053	0.013	0.245	18.523	5.198

Model Comparison

Models	P(M)	P(M|data)	BF $_{M}$	BF $_{01}$	error %
Condition + Time + Volunteers + Condition ✻ Time + Condition ✻ Volunteers + Time ✻ Volunteers	0.053	0.013	0.236	19.225	3.883
Condition + Time + Condition ✻ Time	0.053	0.008	0.142	31.879	1.673
Condition + Time + Volunteers	0.053	0.007	0.124	36.286	1.584
Time + Volunteers + Time ✻ Volunteers	0.053	0.004	0.077	58.041	1.615
Condition + Time + Volunteers + Condition ✻ Time	0.053	0.004	0.065	68.591	2.645
Condition + Time + Volunteers + Time ✻ Volunteers	0.053	0.001	0.023	193.355	4.131
Condition + Time + Volunteers + Condition ✻ Time + Time ✻ Volunteers	0.053	6.406e -4	0.012	388.187	3.158

Note. All models include subject

Model Averaged Q-Q Plot

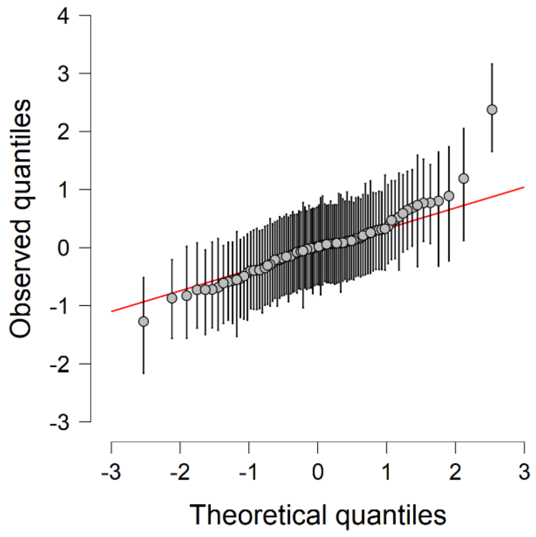

Post Hoc Comparisons - Condition

		Prior Odds	Posterior Odds	BF $_{01,\,U}$	error %
taVNS	Sham	1.000	4.874	4.874	1.207e -5

Post Hoc Comparisons - Time

		Prior Odds	Posterior Odds	BF $_{01,\,U}$	error %
T1-T0	T2-T0	1.000	5.748	5.748	1.346e -5

Note. The posterior odds have been corrected for multiple testing by fixing to 0.5 the prior probability that the null hypothesis holds across all comparisons (Westfall, Johnson, & Utts, 1997). Individual comparisons are based on the default t-test with a Cauchy (0, r = 1/sqrt(2)) prior. The "U" in the Bayes factor denotes that it is uncorrected.

<u>Cerebral responses</u>

- *Laser evoked ERPs : Δ(OFF-ON) P2 Latency*

Model Comparison

Models	P(M)	P(M|data)	BF_M	BF_{01}	error %
Null model (incl. subject)	0.200	0.436	3.095	1.000	
Volunteers	0.200	0.211	1.069	2.068	0.657
Condition + Volunteers + Condition ✻ Volunteers	0.200	0.145	0.679	3.006	1.973
Condition	0.200	0.137	0.635	3.185	1.208
Condition + Volunteers	0.200	0.071	0.305	6.159	7.189

Note. All models include subject

Model Averaged Q-Q Plot

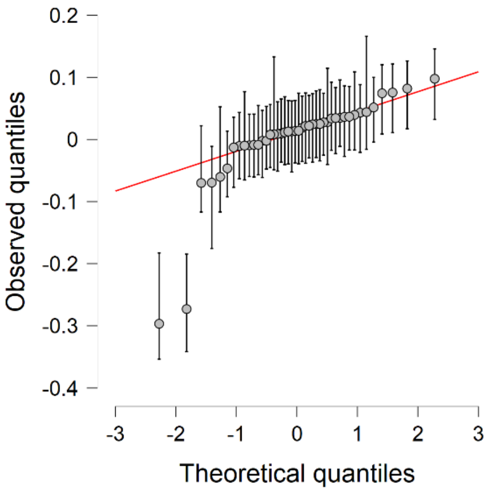

Post Hoc Comparisons - Condition

		Prior Odds	Posterior Odds	$BF_{01, U}$	error %
taVNS	Sham	1.000	4.171	4.171	0.031

Note. The posterior odds have been corrected for multiple testing by fixing to 0.5 the prior probability that the null hypothesis holds across all comparisons (Westfall, Johnson, & Utts, 1997). Individual comparisons are based on the default t-test with a Cauchy (0, r = 1/sqrt(2)) prior. The "U" in the Bayes factor denotes that it is uncorrected.

- *Laser evoked ERPs : Δ(OFF-ON) P2 amplitude:*

Model Comparison

Models	P(M)	P(M|data)	BF_M	BF_{01}	error %
Null model (incl. subject)	0.200	0.445	3.209	1.000	
Volunteers	0.200	0.220	1.126	2.027	0.587
Condition	0.200	0.146	0.685	3.043	1.357
Condition + Volunteers + Condition ✻ Volunteers	0.200	0.112	0.502	**3.992**	7.496
Condition + Volunteers	0.200	0.077	0.336	**5.747**	5.666

Note. All models include subject

Model Averaged Q-Q Plot

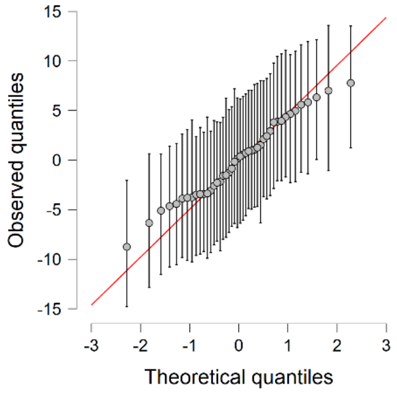

Post Hoc Comparisons - Condition

		Prior Odds	Posterior Odds	BF $_{01, U}$	error %
taVNS	Sham	1.000	4.034	4.034	0.032

Note. The posterior odds have been corrected for multiple testing by fixing to 0.5 the prior probability that the null hypothesis holds across all comparisons (Westfall, Johnson, & Utts, 1997). Individual comparisons are based on the default t-test with a Cauchy (0, r = 1/sqrt(2)) prior. The "U" in the Bayes factor denotes that it is uncorrected.

- *Laser evoked ERPs : Δ(OFF-ON) N2 latency:*

Model Comparison

Models	P(M)	P(M|data)	BF $_M$	BF $_{01}$	error %
Null model (incl. subject)	0.200	0.403	2.705	1.000	
Condition + Volunteers + Condition ✻ Volunteers	0.200	0.298	1.697	1.355	13.501
Condition	0.200	0.158	0.750	2.555	2.107
Volunteers	0.200	0.100	0.447	4.016	0.166
Condition + Volunteers	0.200	0.040	0.168	9.998	1.988

Note. All models include subject

Model Averaged Q-Q Plot

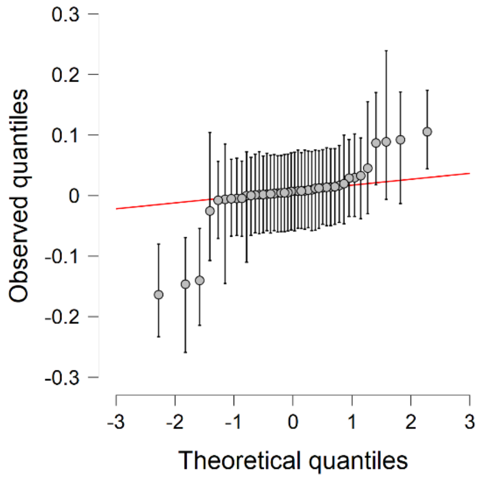

Post Hoc Comparisons - Condition

		Prior Odds	Posterior Odds	BF $_{01, U}$	error %
taVNS	Sham	1.000	3.551	3.551	0.032

Note. The posterior odds have been corrected for multiple testing by fixing to 0.5 the prior probability that the null hypothesis holds across all comparisons (Westfall, Johnson, & Utts, 1997). Individual comparisons are based on the default t-test with a Cauchy (0, r = 1/sqrt(2)) prior. The "U" in the Bayes factor denotes that it is uncorrected.

- *Laser evoked ERPs : **Δ**(OFF-ON) N2 amplitude:*

Model Comparison

Models	P(M)	P(M|data)	BF_{M}	BF_{01}	error %
Null model (incl. subject)	0.200	0.446	3.221	1.000	
Condition + Volunteers + Condition ✻ Volunteers	0.200	0.226	1.171	1.970	3.624
Volunteers	0.200	0.153	0.720	2.924	0.245
Condition	0.200	0.131	0.604	3.399	1.032
Condition + Volunteers	0.200	0.044	0.183	10.183	0.829

Note. All models include subject

Model Averaged Q-Q Plot

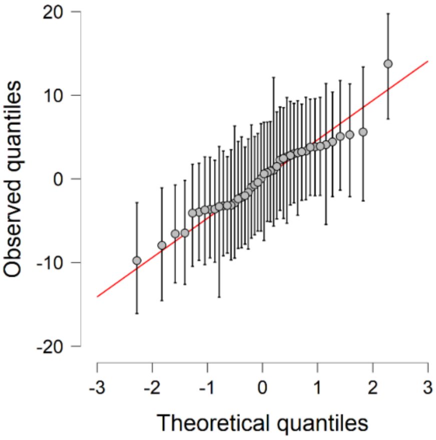

Post Hoc Comparisons - Condition

		Prior Odds	Posterior Odds	$BF_{01,U}$	error %
taVNS	Sham	1.000	4.483	4.483	0.031

Note. The posterior odds have been corrected for multiple testing by fixing to 0.5 the prior probability that the null hypothesis holds across all comparisons (Westfall, Johnson, & Utts, 1997). Individual comparisons are based on the default t-test with a Cauchy (0, r = 1/sqrt(2)) prior. The "U" in the Bayes factor denotes that it is uncorrected.

- *Laser evoked ERPs : **Δ**(OFF-ON) N2P2 amplitude:*

Model Comparison

Models	P(M)	P(M|data)	BF_{M}	BF_{01}	error %
Null model (incl. subject)	0.200	0.428	2.995	1.000	
Volunteers	0.200	0.217	1.109	1.973	0.705
Condition + Volunteers + Condition ✻ Volunteers	0.200	0.159	0.757	2.692	33.979
Condition	0.200	0.130	0.600	3.283	1.551
Condition + Volunteers	0.200	0.065	0.279	6.564	1.192

Note. All models include subject

Model Averaged Q-Q Plot

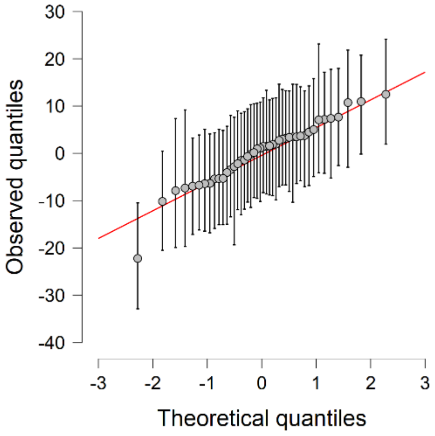

Post Hoc Comparisons - Condition

		Prior Odds	Posterior Odds	BF $_{01, U}$	error %
taVNS	Sham	1.000	4.283	4.283	0.031

Note. The posterior odds have been corrected for multiple testing by fixing to 0.5 the prior probability that the null hypothesis holds across all comparisons (Westfall, Johnson, & Utts, 1997). Individual comparisons are based on the default t-test with a Cauchy (0, r = 1/sqrt(2)) prior. The "U" in the Bayes factor denotes that it is uncorrected.

- *Vibrotactile-evoked ERPs : Δ(OFF-ON) P2 latency:*

Model Comparison

Models	P(M)	P(M|data)	BF $_M$	BF $_{01}$	error %
Null model (incl. subject)	0.200	0.349	2.147	1.000	
Volunteers	0.200	0.257	1.384	1.358	2.779
Condition	0.200	0.153	0.723	2.280	10.049
Condition + Volunteers + Condition ✻ Volunteers	0.200	0.127	0.581	2.755	1.655
Condition + Volunteers	0.200	0.114	0.513	3.072	5.695

Note. All models include subject

Model Averaged Q-Q Plot

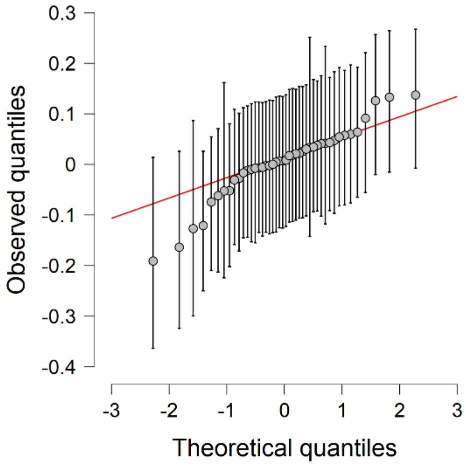

Post Hoc Comparisons - Condition

		Prior Odds	Posterior Odds	BF $_{01, U}$	error %
taVNS	Sham	1.000	3.156	3.156	0.031

Note. The posterior odds have been corrected for multiple testing by fixing to 0.5 the prior probability that the null hypothesis holds across all comparisons (Westfall, Johnson, & Utts, 1997). Individual comparisons are based on the default t-test with a Cauchy (0, r = 1/sqrt(2)) prior. The "U" in the Bayes factor denotes that it is uncorrected.

- *Vibrotactile-evoked ERPs : Δ(OFF-ON) P2 amplitude:*

Model Comparison

Models	P(M)	P(M|data)	BF_M	BF_{01}	error %
Null model (incl. subject)	0.200	0.259	1.400	1.000	
Volunteers	0.200	0.221	1.138	1.171	1.238
Condition	0.200	0.189	0.935	1.369	2.093
Condition + Volunteers	0.200	0.172	0.831	1.508	1.447
Condition + Volunteers + Condition ✻ Volunteers	0.200	0.158	0.749	1.643	1.580

Note. All models include subject

Model Averaged Q-Q Plot

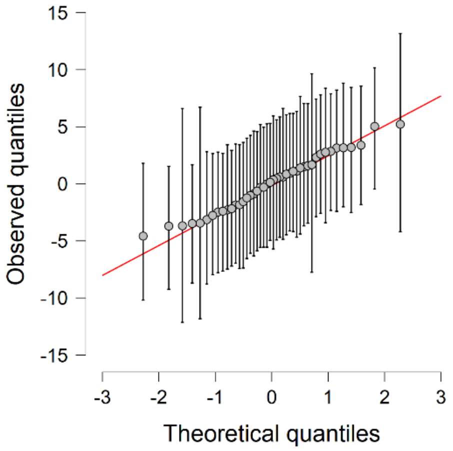

Post Hoc Comparisons - Condition

		Prior Odds	Posterior Odds	$BF_{01, U}$	error %
taVNS	Sham	1.000	1.621	1.621	0.016

Note. The posterior odds have been corrected for multiple testing by fixing to 0.5 the prior probability that the null hypothesis holds across all comparisons (Westfall, Johnson, & Utts, 1997). Individual comparisons are based on the default t-test with a Cauchy (0, r = 1/sqrt(2)) prior. The "U" in the Bayes factor denotes that it is uncorrected.

- *Vibrotactile-evoked ERPs : Δ(OFF-ON) N2 latency:*

Model Comparison

Models	P(M)	P(M|data)	BF_M	BF_{01}	error %
Null model (incl. subject)	0.200	0.410	2.774	1.000	
Condition + Volunteers + Condition ✻ Volunteers	0.200	0.285	1.598	1.435	40.438
Volunteers	0.200	0.138	0.643	2.959	0.237
Condition	0.200	0.123	0.560	3.333	0.751
Condition + Volunteers	0.200	0.044	0.183	9.360	2.960

Note. All models include subject

Model Averaged Q-Q Plot

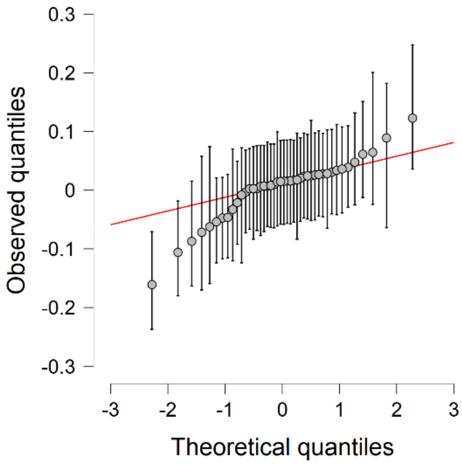

Post Hoc Comparisons - Condition

		Prior Odds	Posterior Odds	BF $_{01, U}$	error %
taVNS	Sham	1.000	4.328	4.328	0.031

Note. The posterior odds have been corrected for multiple testing by fixing to 0.5 the prior probability that the null hypothesis holds across all comparisons (Westfall, Johnson, & Utts, 1997). Individual comparisons are based on the default t-test with a Cauchy (0, r = 1/sqrt(2)) prior. The "U" in the Bayes factor denotes that it is uncorrected.

- *Vibrotactile-evoked ERPs : Δ(OFF-ON) N2 amplitude:*

Model Comparison

Models	P(M)	P(M|data)	BF $_M$	BF $_{01}$	error %
Null model (incl. subject)	0.200	0.371	2.355	1.000	
Volunteers	0.200	0.236	1.238	1.568	1.496
Condition	0.200	0.161	0.770	2.295	4.292
Condition + Volunteers + Condition ✻ Volunteers	0.200	0.136	0.631	2.721	1.670
Condition + Volunteers	0.200	0.095	0.421	3.888	2.009

Note. All models include subject

Model Averaged Q-Q Plot

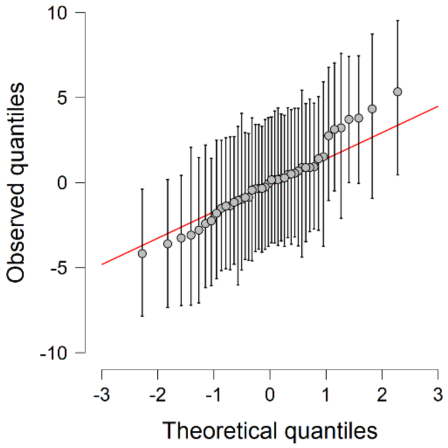

Post Hoc Comparisons - Condition

		Prior Odds	Posterior Odds	BF $_{01, U}$	error %
taVNS	Sham	1.000	3.042	3.042	0.030

Note. The posterior odds have been corrected for multiple testing by fixing to 0.5 the prior probability that the null hypothesis holds across all comparisons (Westfall, Johnson, & Utts, 1997). Individual comparisons are based on the default t-test with a Cauchy (0, r = 1/sqrt(2)) prior. The "U" in the Bayes factor denotes that it is uncorrected.

- *Vibrotactile-evoked ERPs : Δ(OFF-ON) N2P2 amplitude:*

Model Comparison

Models	P(M)	P(M|data)	BF_M	BF_{01}	error %
Null model (incl. subject)	0.200	0.308	1.777	1.000	
Volunteers	0.200	0.262	1.421	1.173	8.644
Condition + Volunteers	0.200	0.161	0.767	1.913	1.493
Condition + Volunteers + Condition ✻ Volunteers	0.200	0.137	0.636	2.242	1.808
Condition	0.200	0.132	0.609	2.328	1.082

Note. All models include subject

Model Averaged Q-Q Plot

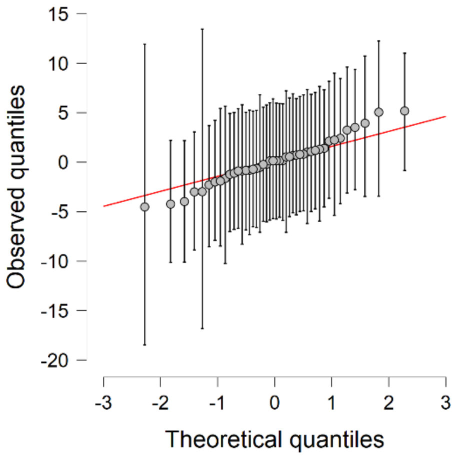

Post Hoc Comparisons - Condition

		Prior Odds	Posterior Odds	$BF_{01,U}$	error %
taVNS	Sham	1.000	2.885	2.885	0.030

Note. The posterior odds have been corrected for multiple testing by fixing to 0.5 the prior probability that the null hypothesis holds across all comparisons (Westfall, Johnson, & Utts, 1997). Individual comparisons are based on the default t-test with a Cauchy (0, r = 1/sqrt(2)) prior. The "U" in the Bayes factor denotes that it is uncorrected.

- *Cool-evoked ERPs : Δ(OFF-ON) P2 latency:*

Model Comparison

Models	P(M)	P(M|data)	BF_M	BF_{01}	error %
Null model (incl. subject)	0.200	0.062	0.266	1.000	
Condition + Volunteers + Condition ✻ Volunteers	0.200	0.905	38.063	0.069	61.590
Condition	0.200	0.020	0.082	3.118	4.561
Volunteers	0.200	0.010	0.040	6.294	0.202
Condition + Volunteers	0.200	0.003	0.012	20.993	1.697

Note. All models include subject

Model Averaged Q-Q Plot

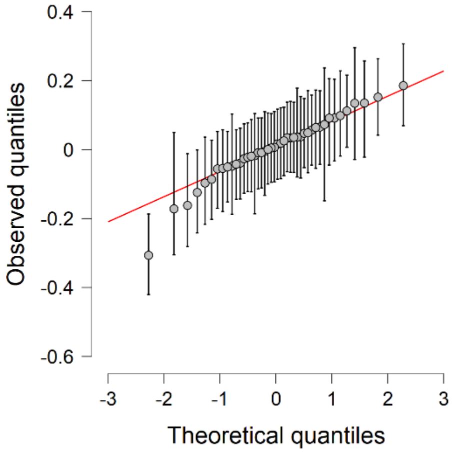

Post Hoc Comparisons - Condition

		Prior Odds	Posterior Odds	BF $_{01, U}$	error %
taVNS	Sham	1.000	4.392	4.392	0.031

Note. The posterior odds have been corrected for multiple testing by fixing to 0.5 the prior probability that the null hypothesis holds across all comparisons (Westfall, Johnson, & Utts, 1997). Individual comparisons are based on the default t-test with a Cauchy (0, r = 1/sqrt(2)) prior. The "U" in the Bayes factor denotes that it is uncorrected.

- *Cool-evoked ERPs : Δ(OFF-ON) P2 amplitude:*

Model Comparison

Models	P(M)	P(M|data)	BF $_{M}$	BF $_{01}$	error %
Null model (incl. subject)	0.200	0.374	2.388	1.000	
Condition + Volunteers + Condition ✻ Volunteers	0.200	0.396	2.620	0.945	8.225
Condition	0.200	0.123	0.564	3.027	1.218
Volunteers	0.200	0.080	0.348	4.677	0.167
Condition + Volunteers	0.200	0.027	0.111	13.852	1.342

Note. All models include subject

Model Averaged Q-Q Plot

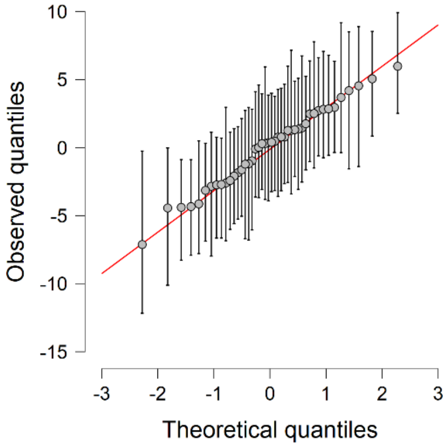

Post Hoc Comparisons - Condition

		Prior Odds	Posterior Odds	BF $_{01, U}$	error %
taVNS	Sham	1.000	4.052	4.052	0.032

Note. The posterior odds have been corrected for multiple testing by fixing to 0.5 the prior probability that the null hypothesis holds across all comparisons (Westfall, Johnson, & Utts, 1997). Individual comparisons are based on the default t-test with a Cauchy (0, r = 1/sqrt(2)) prior. The "U" in the Bayes factor denotes that it is uncorrected.

- *Cool-evoked ERPs : Δ(OFF-ON) N2 latency:*

Model Comparison

Models	P(M)	P(M|data)	BF $_M$	BF $_{01}$	error %
Null model (incl. subject)	0.200	0.071	0.306	1.000	
Condition	0.200	0.364	2.288	0.196	2.802
Condition + Volunteers	0.200	0.285	1.596	0.249	7.914
Condition + Volunteers + Condition ✻ Volunteers	0.200	0.235	1.227	0.303	1.589
Volunteers	0.200	0.045	0.188	1.583	1.970

Note. All models include subject

Model Averaged Q-Q Plot

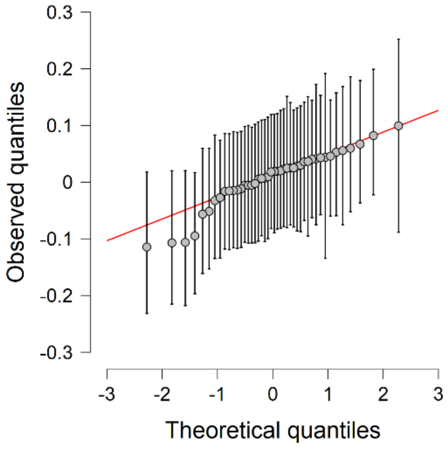

Post Hoc Comparisons - Condition

		Prior Odds	Posterior Odds	BF $_{01, U}$	error %
taVNS	Sham	1.000	0.206	0.206	0.001

Note. The posterior odds have been corrected for multiple testing by fixing to 0.5 the prior probability that the null hypothesis holds across all comparisons (Westfall, Johnson, & Utts, 1997). Individual comparisons are based on the default t-test with a Cauchy (0, r = 1/sqrt(2)) prior. The "U" in the Bayes factor denotes that it is uncorrected.

- *Cool-evoked ERPs : Δ(OFF-ON) N2 amplitude:*

Model Comparison

Models	P(M)	P(M|data)	BF $_M$	BF $_{01}$	error %
Null model (incl. subject)	0.200	0.457	3.368	1.000	
Condition + Volunteers + Condition ✻ Volunteers	0.200	0.171	0.826	2.671	6.357
Condition	0.200	0.162	0.775	2.817	0.790
Volunteers	0.200	0.154	0.729	2.964	0.247
Condition + Volunteers	0.200	0.055	0.234	8.262	0.875

Note. All models include subject

Model Averaged Q-Q Plot

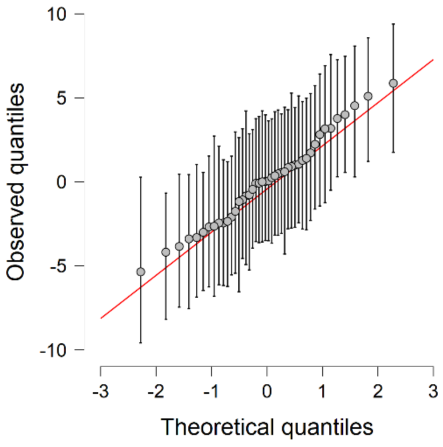

Post Hoc Comparisons - Condition

		Prior Odds	Posterior Odds	BF $_{01, U}$	error %
taVNS	Sham	1.000	3.702	3.702	0.032

Note. The posterior odds have been corrected for multiple testing by fixing to 0.5 the prior probability that the null hypothesis holds across all comparisons (Westfall, Johnson, & Utts, 1997). Individual comparisons are based on the default t-test with a Cauchy (0, r = 1/sqrt(2)) prior. The "U" in the Bayes factor denotes that it is uncorrected.

- *Cool-evoked ERPs : Δ(OFF-ON) N2P2 amplitude:*

Model Comparison

Models	P(M)	P(M|data)	BF $_M$	BF $_{01}$	error %
Null model (incl. subject)	0.200	0.462	3.437	1.000	
Condition + Volunteers + Condition ✻ Volunteers	0.200	0.250	1.332	1.850	6.105
Condition	0.200	0.136	0.631	3.392	1.065
Volunteers	0.200	0.117	0.530	3.947	0.162
Condition + Volunteers	0.200	0.035	0.144	13.297	2.612

Note. All models include subject

Model Averaged Q-Q Plot

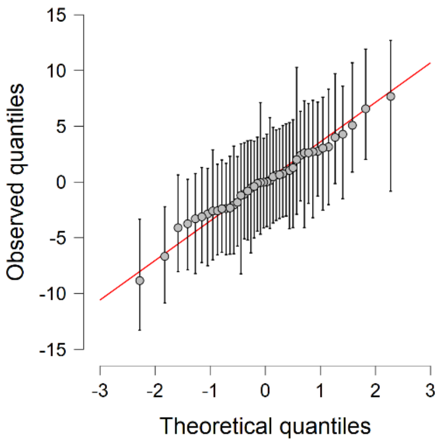

Post Hoc Comparisons - Condition

		Prior Odds	Posterior Odds	BF $_{01, U}$	error %
taVNS	Sham	1.000	4.474	4.474	0.031

Note. The posterior odds have been corrected for multiple testing by fixing to 0.5 the prior probability that the null hypothesis holds across all comparisons (Westfall, Johnson, & Utts, 1997). Individual comparisons are based on the default t-test with a Cauchy (0, r = 1/sqrt(2)) prior. The "U" in the Bayes factor denotes that it is uncorrected.

Experiment 2

Behavioral responses

Detection Thresholds (T1-T0)

- *Heat sensitive C-fibers*

Model Comparison

Models	P(M)	P(M|data)	BF_M	BF_{01}	error %
Null model (incl. subject)	0.500	0.746	2.933	1.000	
Condition	0.500	0.254	0.341	2.933	0.992

Note. All models include subject

Model Averaged Q-Q Plot

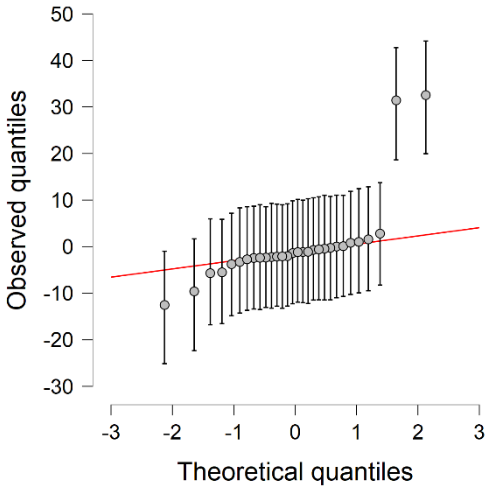

Post Hoc Comparisons - Condition

		Prior Odds	Posterior Odds	$BF_{01,U}$	error %
taVNS	Sham	1.000	3.779	3.779	0.003

Note. The posterior odds have been corrected for multiple testing by fixing to 0.5 the prior probability that the null hypothesis holds across all comparisons (Westfall, Johnson, & Utts, 1997). Individual comparisons are based on the default t-test with a Cauchy (0, r = 1/sqrt(2)) prior. The "U" in the Bayes factor denotes that it is uncorrected.

- *Heat sensitive Aδ-fibers*

Model Comparison

Models	P(M)	P(M|data)	BF_M	BF_{01}	error %
Null model (incl. subject)	0.500	0.746	2.931	1.000	
Condition	0.500	0.254	0.341	2.931	1.168

Note. All models include subject

Model Averaged Q-Q Plot

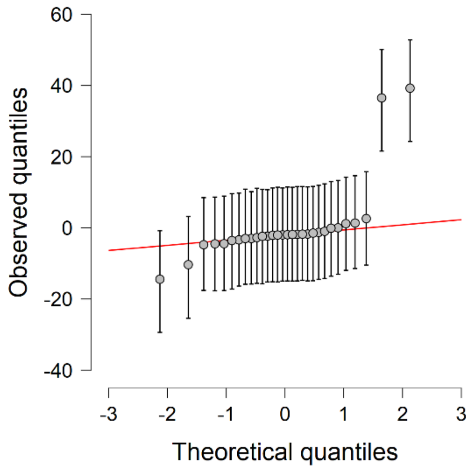

Post Hoc Comparisons - Condition

		Prior Odds	Posterior Odds	BF $_{01, U}$	error %
taVNS	Sham	1.000	3.799	3.799	0.003

Note. The posterior odds have been corrected for multiple testing by fixing to 0.5 the prior probability that the null hypothesis holds across all comparisons (Westfall, Johnson, & Utts, 1997). Individual comparisons are based on the default t-test with a Cauchy (0, r = 1/sqrt(2)) prior. The "U" in the Bayes factor denotes that it is uncorrected.

- *Mechanosensitive Aβ-fibers*

Model Comparison

Models	P(M)	P(M|data)	BF $_M$	BF $_{01}$	error %
Null model (incl. subject)	0.500	0.550	1.224	1.000	
Condition	0.500	0.450	0.817	1.224	1.006

Note. All models include subject

Model Averaged Q-Q Plot

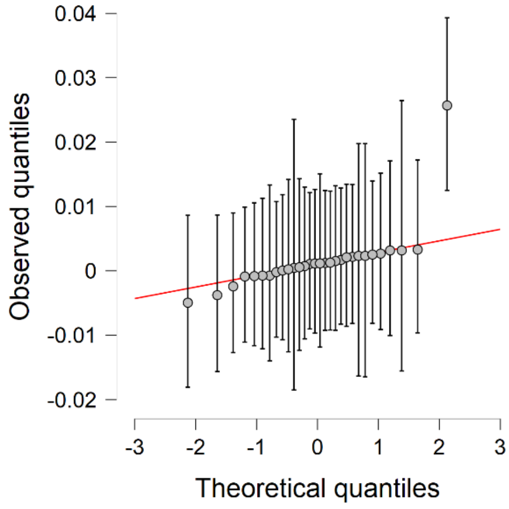

Post Hoc Comparisons - Condition

		Prior Odds	Posterior Odds	BF $_{01, U}$	error %
taVNS	Sham	1.000	1.591	1.591	0.012

Note. The posterior odds have been corrected for multiple testing by fixing to 0.5 the prior probability that the null hypothesis holds across all comparisons (Westfall, Johnson, & Utts, 1997). Individual comparisons are based on the default t-test with a Cauchy (0, r = 1/sqrt(2)) prior. The "U" in the Bayes factor denotes that it is uncorrected.

Perception Intensity (ΔOFF-ON)

- *Laser*

Model Comparison

Models	P(M)	P(M|data)	BF_M	BF_{01}	error %
Null model (incl. subject)	0.500	0.722	2.596	1.000	
Condition	0.500	0.278	0.385	2.596	0.908

Note. All models include subject

Model Averaged Q-Q Plot

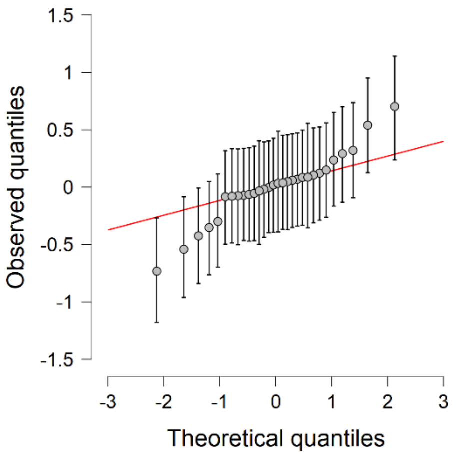

Post Hoc Comparisons - Condition

		Prior Odds	Posterior Odds	$BF_{01, U}$	error %
taVNS	Sham	1.000	3.315	3.315	0.003

Note. The posterior odds have been corrected for multiple testing by fixing to 0.5 the prior probability that the null hypothesis holds across all comparisons (Westfall, Johnson, & Utts, 1997). Individual comparisons are based on the default t-test with a Cauchy (0, r = 1/sqrt(2)) prior. The "U" in the Bayes factor denotes that it is uncorrected.

- *Vibrotactile*

Model Comparison

Models	P(M)	P(M|data)	BF_M	BF_{01}	error %
Null model (incl. subject)	0.500	0.713	2.482	1.000	
Condition	0.500	0.287	0.403	2.482	2.323

Note. All models include subject

Model Averaged Q-Q Plot

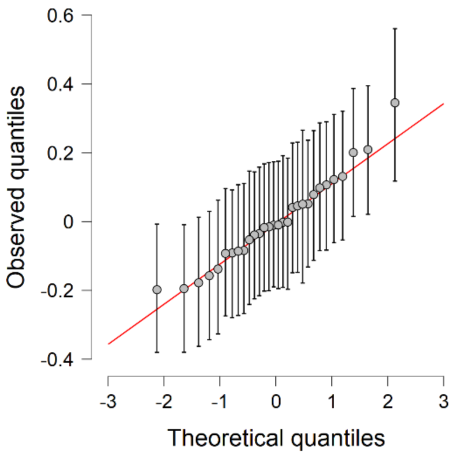

Post Hoc Comparisons - Condition

		Prior Odds	Posterior Odds	$BF_{01, U}$	error %
taVNS	Sham	1.000	3.275	3.275	0.003

Note. The posterior odds have been corrected for multiple testing by fixing to 0.5 the prior probability that the null hypothesis holds across all comparisons (Westfall, Johnson, & Utts, 1997). Individual comparisons are based on the default t-test with a Cauchy (0, r = 1/sqrt(2)) prior. The "U" in the Bayes factor denotes that it is uncorrected.

Cerebral responses

- *Laser-evoked ERPs : Δ(OFF-ON) P2 latency:*

Model Comparison

Models	P(M)	P(M|data)	BF $_M$	BF $_{01}$	error %
Null model (incl. subject)	0.500	0.694	2.273	1.000	
Condition	0.500	0.306	0.440	2.273	1.952

Note. All models include subject

Model Averaged Q-Q Plot

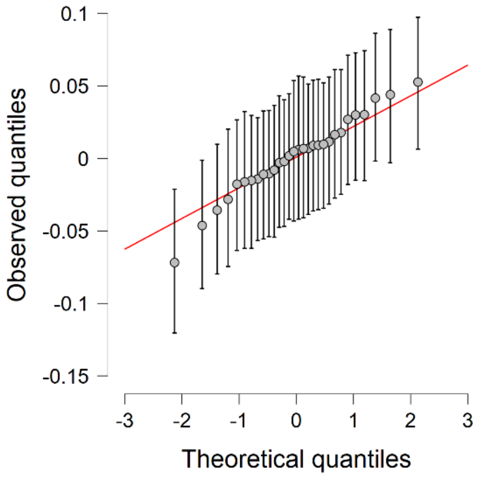

Post Hoc Comparisons - Condition

		Prior Odds	Posterior Odds	BF $_{01, U}$	error %
taVNS	Sham	1.000	2.916	2.916	0.004

Note. The posterior odds have been corrected for multiple testing by fixing to 0.5 the prior probability that the null hypothesis holds across all comparisons (Westfall, Johnson, & Utts, 1997). Individual comparisons are based on the default t-test with a Cauchy (0, r = 1/sqrt(2)) prior. The "U" in the Bayes factor denotes that it is uncorrected.

- *Laser-evoked ERPs : Δ(OFF-ON) P2 amplitude:*

Model Comparison

Models	P(M)	P(M|data)	BF $_M$	BF $_{01}$	error %
Null model (incl. subject)	0.500	0.737	2.798	1.000	
Condition	0.500	0.263	0.357	2.798	1.122

Note. All models include subject

Model Averaged Q-Q Plot

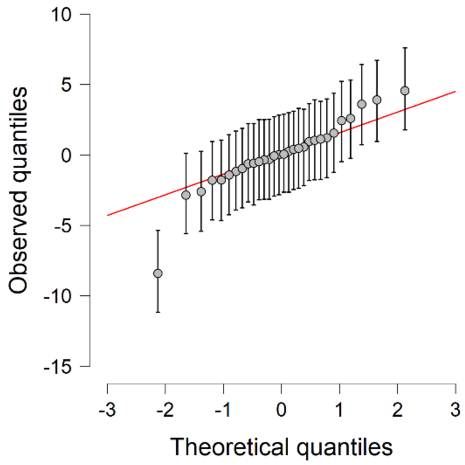

Post Hoc Comparisons - Condition

		Prior Odds	Posterior Odds	BF $_{01, U}$	error %
taVNS	Sham	1.000	3.690	3.690	0.003

Note. The posterior odds have been corrected for multiple testing by fixing to 0.5 the prior probability that the null hypothesis holds across all comparisons (Westfall, Johnson, & Utts, 1997). Individual comparisons are based on the default t-test with a Cauchy (0, r = 1/sqrt(2)) prior. The "U" in the Bayes factor denotes that it is uncorrected.

- *Laser-evoked ERPs : **Δ**(OFF-ON) N2 latency:*

Model Comparison

Models	P(M)	P(M|data)	BF $_M$	BF $_{01}$	error %
Null model (incl. subject)	0.500	0.734	2.762	1.000	
Condition	0.500	0.266	0.362	2.762	1.243

Note. All models include subject

Model Averaged Q-Q Plot

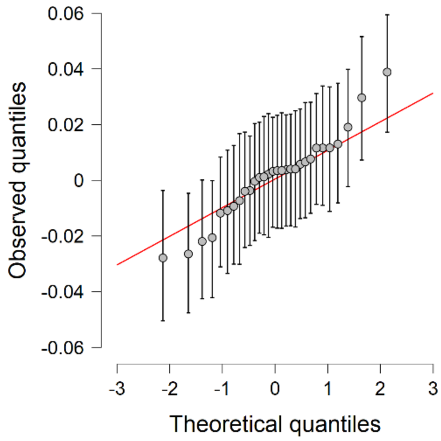

Post Hoc Comparisons - Condition

		Prior Odds	Posterior Odds	BF $_{01, U}$	error %
taVNS	Sham	1.000	3.592	3.592	0.003

Note. The posterior odds have been corrected for multiple testing by fixing to 0.5 the prior probability that the null hypothesis holds across all comparisons (Westfall, Johnson, & Utts, 1997). Individual comparisons are based on the default t-test with a Cauchy (0, r = 1/sqrt(2)) prior. The "U" in the Bayes factor denotes that it is uncorrected.

- *Laser-evoked ERPs : Δ(OFF-ON) N2 amplitude:*

Model Comparison

Models	P(M)	P(M|data)	BF $_M$	BF $_{01}$	error %
Null model (incl. subject)	0.500	0.604	1.526	1.000	
Condition	0.500	0.396	0.655	1.526	0.777

Note. All models include subject

Model Averaged Q-Q Plot

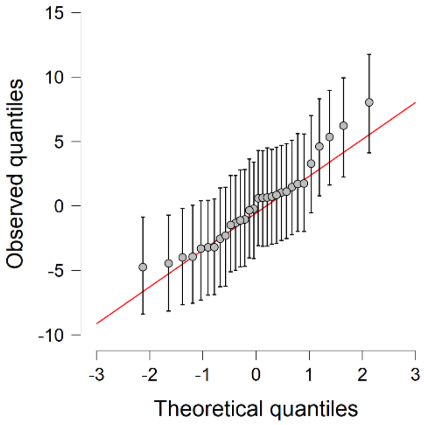

Post Hoc Comparisons - Condition

		Prior Odds	Posterior Odds	BF $_{01, U}$	error %
taVNS	Sham	1.000	2.268	2.268	0.007

Note. The posterior odds have been corrected for multiple testing by fixing to 0.5 the prior probability that the null hypothesis holds across all comparisons (Westfall, Johnson, & Utts, 1997). Individual comparisons are based on the default t-test with a Cauchy (0, r = 1/sqrt(2)) prior. The "U" in the Bayes factor denotes that it is uncorrected.

- *Laser-evoked ERPs : Δ(OFF-ON) N2P2 amplitude:*

Model Comparison

Models	P(M)	P(M|data)	BF $_M$	BF $_{01}$	error %
Null model (incl. subject)	0.500	0.521	1.090	1.000	
Condition	0.500	0.479	0.918	1.090	1.836

Note. All models include subject

Model Averaged Q-Q Plot

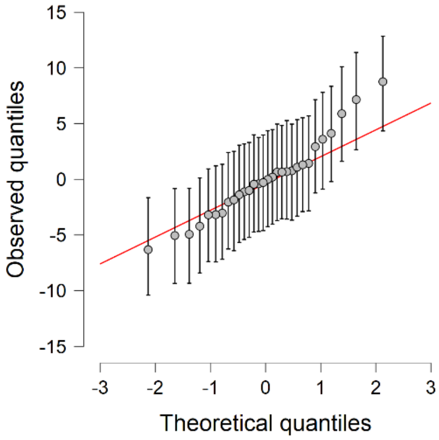

Post Hoc Comparisons - Condition

		Prior Odds	Posterior Odds	BF $_{01, U}$	error %
taVNS	Sham	1.000	1.767	1.767	0.010

Note. The posterior odds have been corrected for multiple testing by fixing to 0.5 the prior probability that the null hypothesis holds across all comparisons (Westfall, Johnson, & Utts, 1997). Individual comparisons are based on the default t-test with a Cauchy (0, r = 1/sqrt(2)) prior. The "U" in the Bayes factor denotes that it is uncorrected.

- *Vibrotactile-evoked ERPs : Δ(OFF-ON) P2 latency:*

Model Comparison

Models	P(M)	P(M|data)	BF $_M$	BF $_{01}$	error %
Condition	0.500	0.618	1.618	1.000	
Null model (incl. subject)	0.500	0.382	0.618	1.618	1.074

Note. All models include subject

Model Averaged Q-Q Plot

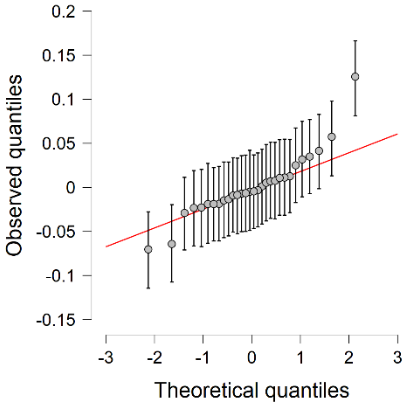

Post Hoc Comparisons - Condition

		Prior Odds	Posterior Odds	BF $_{01,U}$	error %
taVNS	Sham	1.000	1.270	1.270	0.013

Note. The posterior odds have been corrected for multiple testing by fixing to 0.5 the prior probability that the null hypothesis holds across all comparisons (Westfall, Johnson, & Utts, 1997). Individual comparisons are based on the default t-test with a Cauchy (0, r = 1/sqrt(2)) prior. The "U" in the Bayes factor denotes that it is uncorrected.

- *Vibrotactile-evoked ERPs : Δ(OFF-ON) P2 amplitude:*

Model Comparison

Models	P(M)	P(M|data)	BF $_M$	BF $_{01}$	error %
Null model (incl. subject)	0.500	0.732	2.727	1.000	
Condition	0.500	0.268	0.367	2.727	0.754

Note. All models include subject

Model Averaged Q-Q Plot

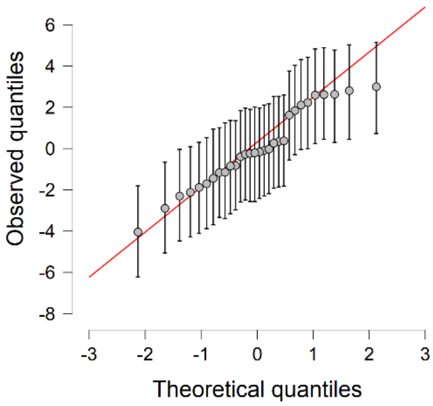

Post Hoc Comparisons - Condition

		Prior Odds	Posterior Odds	BF $_{01,U}$	error %
taVNS	Sham	1.000	3.518	3.518	0.003

Note. The posterior odds have been corrected for multiple testing by fixing to 0.5 the prior probability that the null hypothesis holds across all comparisons (Westfall, Johnson, & Utts, 1997). Individual comparisons are based on the default t-test with a Cauchy (0, r = 1/sqrt(2)) prior. The "U" in the Bayes factor denotes that it is uncorrected.

- *Vibrotactile-evoked ERPs : Δ(OFF-ON) N2 latency:*

Bayesian Repeated Measures ANOVA

Model Comparison

Models	P(M)	P(M|data)	BF $_M$	BF $_{01}$	error %
Null model (incl. subject)	0.500	0.661	1.948	1.000	
Condition	0.500	0.339	0.513	1.948	1.010

Note. All models include subject

Model Averaged Q-Q Plot

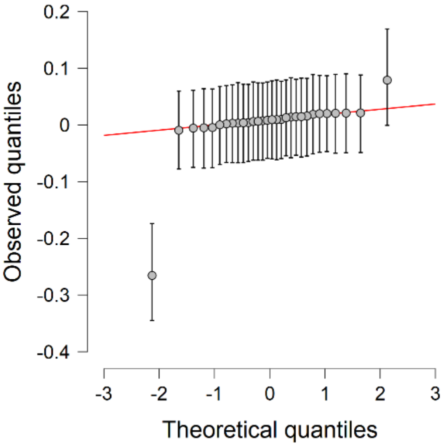

Post Hoc Comparisons - Condition

		Prior Odds	Posterior Odds	BF $_{01, U}$	error %
taVNS	Sham	1.000	2.625	2.625	0.005

Note. The posterior odds have been corrected for multiple testing by fixing to 0.5 the prior probability that the null hypothesis holds across all comparisons (Westfall, Johnson, & Utts, 1997). Individual comparisons are based on the default t-test with a Cauchy (0, r = 1/sqrt(2)) prior. The "U" in the Bayes factor denotes that it is uncorrected.

- *Vibrotactile-evoked ERPs : Δ(OFF-ON) N2 amplitude:*

Model Comparison

Models	P(M)	P(M|data)	BF $_M$	BF $_{01}$	error %
Null model (incl. subject)	0.500	0.135	0.156	1.000	
Condition	0.500	0.865	6.430	0.156	0.936

Note. All models include subject

Model Averaged Q-Q Plot

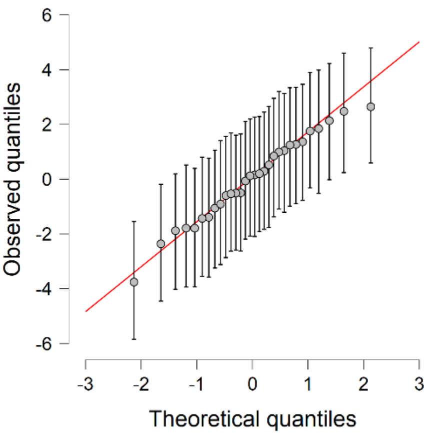

Post Hoc Comparisons - Condition

		Prior Odds	Posterior Odds	BF $_{01, U}$	error %
taVNS	Sham	1.000	0.327	0.327	6.513e -5

Note. The posterior odds have been corrected for multiple testing by fixing to 0.5 the prior probability that the null hypothesis holds across all comparisons (Westfall, Johnson, & Utts, 1997). Individual comparisons are based on the default t-test with a Cauchy (0, r = 1/sqrt(2)) prior. The "U" in the Bayes factor denotes that it is uncorrected.

- *Vibrotactile-evoked ERPs : Δ(OFF-ON) N2P2 amplitude:*

Model Comparison

Models	P(M)	P(M|data)	BF_M	BF_{01}	error %
Null model (incl. subject)	0.500	0.600	1.498	1.000	
Condition	0.500	0.400	0.667	1.498	1.869

Note. All models include subject

Model Averaged Q-Q Plot

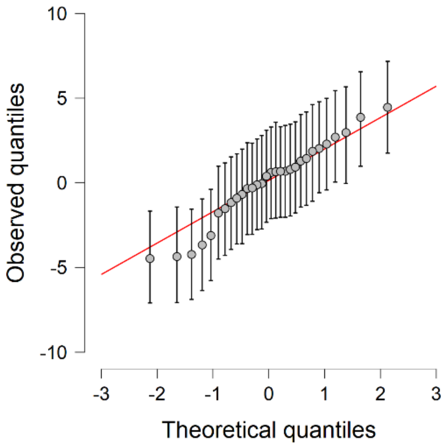

Post Hoc Comparisons - Condition

		Prior Odds	Posterior Odds	$BF_{01, U}$	error %
taVNS	Sham	1.000	2.283	2.283	0.007

Note. The posterior odds have been corrected for multiple testing by fixing to 0.5 the prior probability that the null hypothesis holds across all comparisons (Westfall, Johnson, & Utts, 1997). Individual comparisons are based on the default t-test with a Cauchy (0, r = 1/sqrt(2)) prior. The "U" in the Bayes factor denotes that it is uncorrected.

Experiment 3

<u>Behavioral responses:</u> Perception Intensity

- *Laser*

Model Comparison

Models	P(M)	P(M|data)	BF $_M$	BF $_{01}$	error %
Null model (incl. subject)	0.500	0.606	1.536	1.000	
Duty Cycle Phase	0.500	0.394	0.651	1.536	1.355

Note. All models include subject

Model Averaged Q-QPlot

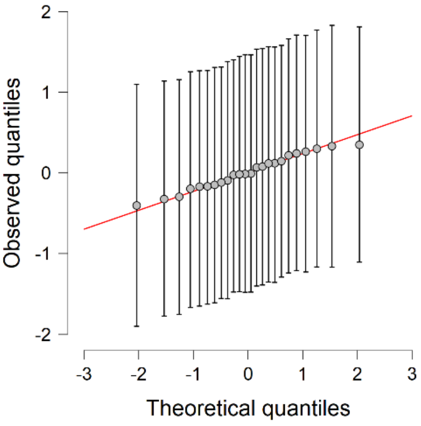

Post Hoc Comparisons - Duty Cycle Phase

		Prior Odds	Posterior Odds	BF $_{01, U}$	error %
OFF	ON	1.000	1.734	1.734	0.005

Note. The posterior odds have been corrected for multiple testing by fixing to 0.5 the prior probability that the null hypothesis holds across all comparisons (Westfall, Johnson, & Utts, 1997). Individual comparisons are based on the default t-test with a Cauchy (0, r = 1/sqrt(2)) prior. The "U" in the Bayes factor denotes that it is uncorrected.

- *Vibrotactile*

Model Comparison

Models	P(M)	P(M|data)	BF $_M$	BF $_{01}$	error %
Null model (incl. subject)	0.500	0.675	2.081	1.000	
Duty Cycle Phase	0.500	0.325	0.481	2.081	0.930

Note. All models include subject

Model Averaged Q-Q Plot

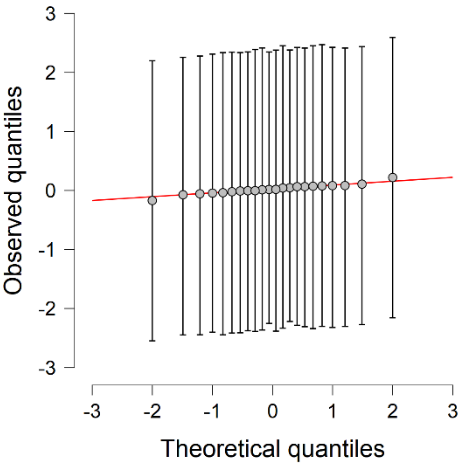

Post Hoc Comparisons - Duty Cycle Phase

		Prior Odds	Posterior Odds	BF $_{01, U}$	error %
OFF	ON	1.000	2.476	2.476	0.017

Note. The posterior odds have been corrected for multiple testing by fixing to 0.5 the prior probability that the null hypothesis holds across all comparisons (Westfall, Johnson, & Utts, 1997). Individual comparisons are based on the default t-test with a Cauchy (0, r = 1/sqrt(2)) prior. The "U" in the Bayes factor denotes that it is uncorrected.

Cerebral responses

- *Laser-evoked ERPs : P2 latency:*

Model Comparison

Models	P(M)	P(M|data)	BF $_M$	BF $_{01}$	error %
Null model (incl. subject)	0.500	0.707	2.415	1.000	
Duty Cycle Phase	0.500	0.293	0.414	2.415	1.006

Note. All models include subject

Model Averaged Q-Q Plot

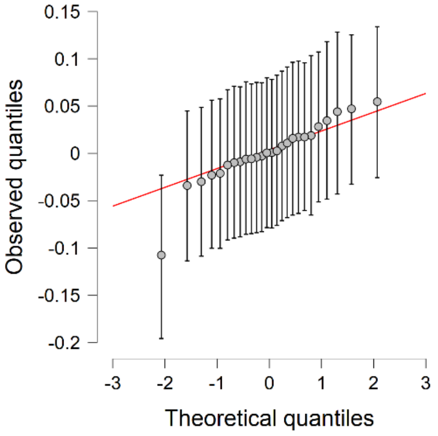

Post Hoc Comparisons - Duty Cycle Phase

		Prior Odds	Posterior Odds	BF $_{01, U}$	error %
OFF	ON	1.000	2.984	2.984	0.017

Note. The posterior odds have been corrected for multiple testing by fixing to 0.5 the prior probability that the null hypothesis holds across all comparisons (Westfall, Johnson, & Utts, 1997). Individual comparisons are based on the default t-test with a Cauchy (0, r = 1/sqrt(2)) prior. The "U" in the Bayes factor denotes that it is uncorrected.

- *Laser-evoked ERPs : P2 amplitude:*

Model Comparison

Models	P(M)	P(M|data)	BF $_M$	BF $_{01}$	error %
Null model (incl. subject)	0.500	0.704	2.376	1.000	
Duty Cycle Phase	0.500	0.296	0.421	2.376	0.769

Note. All models include subject

Model averaged Q-Q Plot

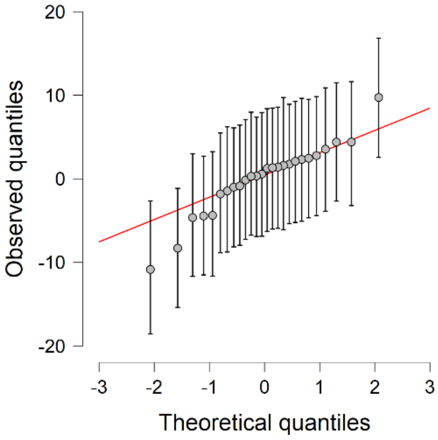

Post Hoc Comparisons - Duty Cycle Phase

		Prior Odds	Posterior Odds	BF $_{01, U}$	error %
OFF	ON	1.000	2.966	2.966	0.017

Note. The posterior odds have been corrected for multiple testing by fixing to 0.5 the prior probability that the null hypothesis holds across all comparisons (Westfall, Johnson, & Utts, 1997). Individual comparisons are based on the default t-test with a Cauchy (0, r = 1/sqrt(2)) prior. The "U" in the Bayes factor denotes that it is uncorrected.

- *Laser-evoked ERPs : N2 latency:*

Model Comparison

Models	P(M)	P(M|data)	BF $_M$	BF $_{01}$	error %
Null model (incl. subject)	0.500	0.734	2.758	1.000	
Duty Cycle Phase	0.500	0.266	0.363	2.758	1.805

Note. All models include subject

Model Averaged Q-Q Plot

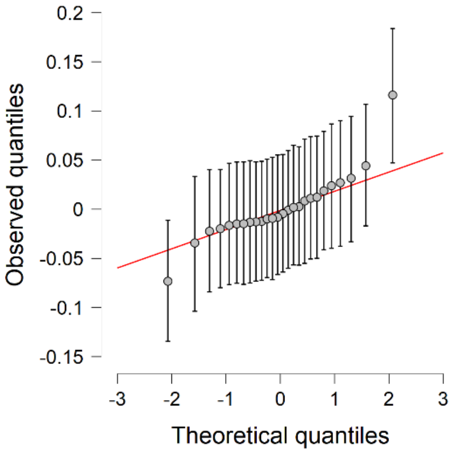

Post Hoc Comparisons - Duty Cycle Phase

		Prior Odds	Posterior Odds	BF $_{01, U}$	error %
OFF	ON	1.000	3.571	3.571	0.016

Note. The posterior odds have been corrected for multiple testing by fixing to 0.5 the prior probability that the null hypothesis holds across all comparisons (Westfall, Johnson, & Utts, 1997). Individual comparisons are based on the default t-test with a Cauchy (0, r = 1/sqrt(2)) prior. The "U" in the Bayes factor denotes that it is uncorrected.

- *Laser-evoked ERPs : N2 amplitude:*

Model Comparison

Models	P(M)	P(M|data)	BF $_M$	BF $_{01}$	error %
Null model (incl. subject)	0.500	0.645	1.817	1.000	
Duty Cycle Phase	0.500	0.355	0.550	1.817	0.873

Note. All models include subject

Model Averaged Q-Q Plot

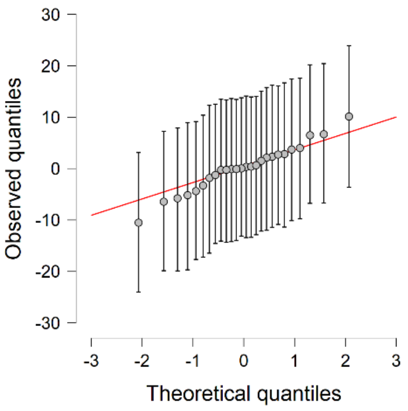

Post Hoc Comparisons - Duty Cycle Phase

		Prior Odds	Posterior Odds	BF $_{01, U}$	error %
OFF	ON	1.000	2.090	2.090	0.021

Note. The posterior odds have been corrected for multiple testing by fixing to 0.5 the prior probability that the null hypothesis holds across all comparisons (Westfall, Johnson, & Utts, 1997). Individual comparisons are based on the default t-test with a Cauchy (0, r = 1/sqrt(2)) prior. The "U" in the Bayes factor denotes that it is uncorrected.

- *Laser-evoked ERPs : N2P2 amplitude:*

Model Comparison

Models	P(M)	P(M|data)	BF_M	BF_{01}	error %
Null model (incl. subject)	0.500	0.376	0.603	1.000	
Duty Cycle Phase	0.500	0.624	1.657	0.603	1.543

Note. All models include subject

Model Averaged Q-Q Plot

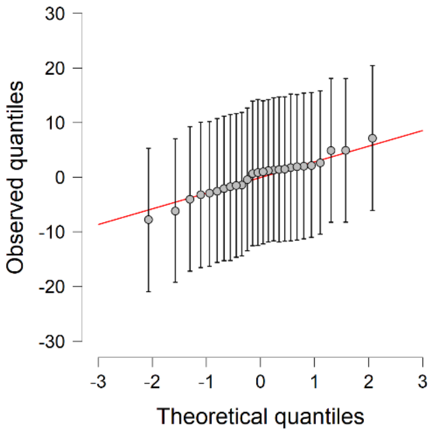

Post Hoc Comparisons - Duty Cycle Phase

		Prior Odds	Posterior Odds	$BF_{01,U}$	error %
OFF	ON	1.000	0.556	0.556	7.889e -4

Note. The posterior odds have been corrected for multiple testing by fixing to 0.5 the prior probability that the null hypothesis holds across all comparisons (Westfall, Johnson, & Utts, 1997). Individual comparisons are based on the default t-test with a Cauchy (0, r = 1/sqrt(2)) prior. The "U" in the Bayes factor denotes that it is uncorrected.

- *Vibrotactile-evoked ERPs : P2 latency:*

Model Comparison

Models	P(M)	P(M|data)	BF_M	BF_{01}	error %
Null model (incl. subject)	0.500	0.529	1.125	1.000	
Duty Cycle Phase	0.500	0.471	0.889	1.125	1.102

Note. All models include subject

Model Averaged Q-Q Plot

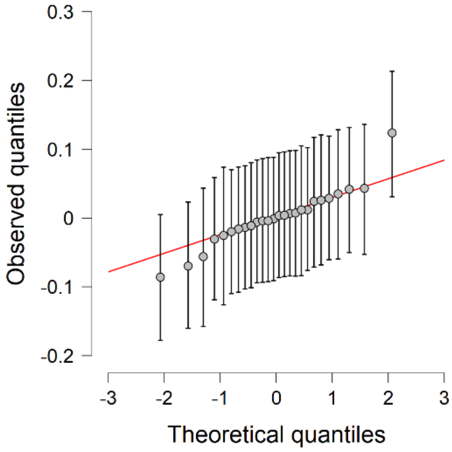

Post Hoc Comparisons - Duty Cycle Phase

		Prior Odds	Posterior Odds	$BF_{01,U}$	error %
OFF	ON	1.000	1.220	1.220	0.005

Note. The posterior odds have been corrected for multiple testing by fixing to 0.5 the prior probability that the null hypothesis holds across all comparisons (Westfall, Johnson, & Utts, 1997). Individual comparisons are based on the default t-test with a Cauchy (0, r = 1/sqrt(2)) prior. The "U" in the Bayes factor denotes that it is uncorrected.

- *Vibrotactile-evoked ERPs : P2 amplitude:*

Model Comparison

Models	P(M)	P(M|data)	BF M	BF 01	error %
Null model (incl. subject)	0.500	0.520	1.084	1.000	
Duty Cycle Phase	0.500	0.480	0.923	1.084	1.126

Note. All models include subject

Model Averaged Q-Q Plot

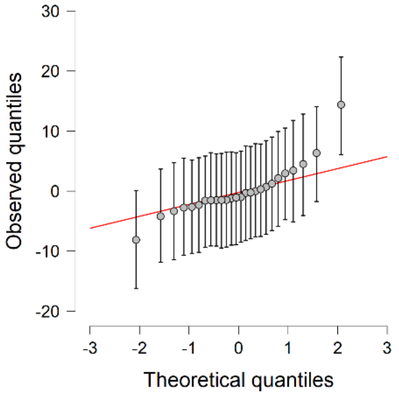

Post Hoc Comparisons - Duty Cycle Phase

		Prior Odds	Posterior Odds	BF 01, U	error %
OFF	ON	1.000	1.256	1.256	0.005

Note. The posterior odds have been corrected for multiple testing by fixing to 0.5 the prior probability that the null hypothesis holds across all comparisons (Westfall, Johnson, & Utts, 1997). Individual comparisons are based on the default t-test with a Cauchy (0, r = 1/sqrt(2)) prior. The "U" in the Bayes factor denotes that it is uncorrected.

- *Vibrotactile-evoked ERPs : N2 latency:*

Model Comparison

Models	P(M)	P(M|data)	BF M	BF 01	error %
Null model (incl. subject)	0.500	0.738	2.811	1.000	
Duty Cycle Phase	0.500	0.262	0.356	2.811	1.103

Note. All models include subject

Model Averaged Q-Q Plot

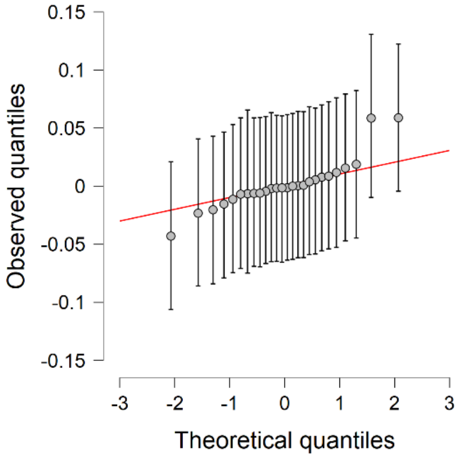

Post Hoc Comparisons - Duty Cycle Phase

		Prior Odds	Posterior Odds	BF 01, U	error %
OFF	ON	1.000	3.593	3.593	0.016

Note. The posterior odds have been corrected for multiple testing by fixing to 0.5 the prior probability that the null hypothesis holds across all comparisons (Westfall, Johnson, & Utts, 1997). Individual comparisons are based on the default t-test with a Cauchy (0, r = 1/sqrt(2)) prior. The "U" in the Bayes factor denotes that it is uncorrected.

- *Vibrotactile-evoked ERPs : N2 amplitude:*

Model Comparison

Models	P(M)	P(M|data)	BF_{M}	BF_{01}	error %
Null model (incl. subject)	0.500	0.712	2.474	1.000	
Duty Cycle Phase	0.500	0.288	0.404	2.474	2.283

Note. All models include subject

Model Averaged Q-Q Plot

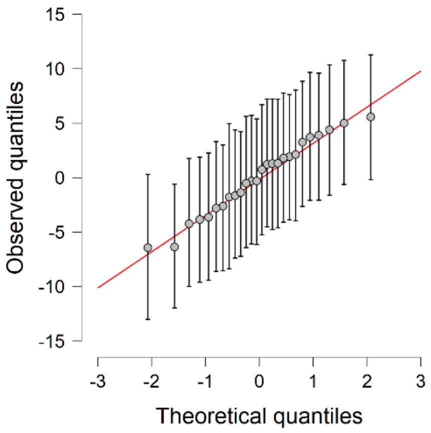

Post Hoc Comparisons - Duty Cycle Phase

		Prior Odds	Posterior Odds	$BF_{01, U}$	error %
OFF	ON	1.000	3.223	3.223	0.017

Note. The posterior odds have been corrected for multiple testing by fixing to 0.5 the prior probability that the null hypothesis holds across all comparisons (Westfall, Johnson, & Utts, 1997). Individual comparisons are based on the default t-test with a Cauchy (0, r = 1/sqrt(2)) prior. The "U" in the Bayes factor denotes that it is uncorrected.

- *Vibrotactile-evoked ERPs : N2P2 amplitude:*

Model Comparison

Models	P(M)	P(M|data)	BF_{M}	BF_{01}	error %
Null model (incl. subject)	0.500	0.330	0.493	1.000	
Duty Cycle Phase	0.500	0.670	2.029	0.493	1.937

Note. All models include subject

Model Averaged Q-Q Plot

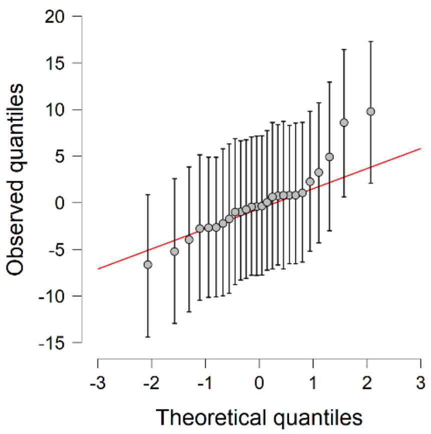

Post Hoc Comparisons - Duty Cycle Phase

		Prior Odds	Posterior Odds	$BF_{01, U}$	error %
OFF	ON	1.000	0.511	0.511	0.001

Note. The posterior odds have been corrected for multiple testing by fixing to 0.5 the prior probability that the null hypothesis holds across all comparisons (Westfall, Johnson, & Utts, 1997). Individual comparisons are based on the default t-test with a Cauchy (0, r = 1/sqrt(2)) prior. The "U" in the Bayes factor denotes that it is uncorrected.
